# Exercise therapy for adolescent idiopathic scoliosis rehabilitation: a bibliometric analysis (1999–2023)

**DOI:** 10.3389/fped.2023.1342327

**Published:** 2024-01-04

**Authors:** Run-Ting Ma, Qiang Wu, Zhen-Da Xu, Li Zhang, Yi-Xin Wei, Qiang Gao

**Affiliations:** ^1^ Department of Rehabilitation Medicine, West China Hospital, Sichuan University, Chengdu, Sichuan Province, China; ^2^ Key Laboratory of Rehabilitation Medicine of Sichuan Province, Chengdu, Sichuan Province, China; ^3^ Department of Computing, The Hong Kong Polytechnic University, Hong Kong, Hong Kong SAR, China

**Keywords:** adolescent idiopathic scoliosis, exercise therapy, rehabilitation, bibliometric, hotspots, research trends

## Abstract

**Background:**

Among the conservative treatments for rehabilitation of adolescent idiopathic scoliosis (AIS), exercise therapy has attracted a large number of studies as its advantages of good clinical effect, high operability, high compliance, few side effects and low cost. We conduct a bibliometric analysis of previous research to identify prevalent areas of study and inform research for the future directions in this paper.

**Methods:**

Relevant publications and reviews were collected using the Science Citation Index Expanded from the Web of Science Core Collection. Information from the included studies was analyzed systematically using VOSviewer and Citespace software to identify patterns regarding publications, keywords, authors, citations, countries, institutions and journals.

**Results:**

A total of 172 articles published from 1999 to 2023 were identified. Over the last decade, the number of publications has gradually increased, reaching a peak of 21 publications in 2021. China, North America and Western European countries and institutions are leading the way as far as the quantity of publications and the total number of citations are concerned. The current areas of focus are the efficacy of exercise therapy in relation to enhancing the quality of life of adolescents during rehabilitation.

**Conclusions:**

This is the first bibliometric analysis that provides a comprehensive review of the research trends and advances in exercise therapy for the rehabilitation of AIS. The study identifies latest research frontiers and hot directions, providing a valuable reference for scholars in the field of exercise therapy.

## 1 Introduction

Scoliosis refers to a three-dimensional spinal deformity (1). Adolescent idiopathic scoliosis (AIS) presents the most common manifestation of scoliosis. It typically presents between the ages of 10 and 16 (2). The initial manifestation of vertebral rotation is commonly reported by most patients. This is followed by asymmetry of the thoracic cage and unequal shoulder height, as well as other deformities including unequal length of lower limbs, razor back, pelvic tilt, lowering height, and in severe cases, movement imbalance and cardiopulmonary dysfunction can also occur (3, 4). Furthermore, individuals with AIS may experience psychological issues, including feelings of inferiority and potential development of psychological disorders, due to alterations in their physical appearance caused by the condition (5). Today, AIS has emerged as a prominent disorder that impedes the growth and development of young individuals. Research indicates that AIS has affected 0.47%–5.20% of adolescents globally (6).

According to the growing body of research, individuals with scoliosis and their families report that wearing a brace is a source of stress and hinders physical and social activities. Moreover, it is widely believed that using a brace can hinder the development of independence (7). Thus, it is imperative to explore other conservative treatment options.

Exercise therapy is widely acknowledged by researchers and the medical communities due to its specific curative effect, high operability, strong patient adherence, minimal side effects, and cost-effectiveness (8). Exercise therapy can decelerate scoliosis progression in AIS patients, improve the motor control of spine and enhance cardiopulmonary function. Furthermore, it helps regulate psychological problems such as depression and anxiety (9, 10). Studies have demonstrated that it is an essential treatment option for patients with mild AIS to progress to surgical criteria, and a supplementary technique for patients with severe scoliosis (11).

Exercise therapy has attracted a great deal of research on the conservative treatment of AIS (12). Common examples of exercise therapy in AIS are listed below: (1) The Schroth methodology, originating from Germany, is widely used and studied. The proprietary Schroth Rotational Angular Breathing (RAB) technique is credited with its success. The method is a three-dimensional scoliosis treatment that emphasises patterned postural correction following the Schroth classification (13). (2) Core stabilization exercises improves the stability of the vertebral column by working out core muscles and enhancing the strength of the deep and superficial muscles on either side of the spine. This ensures that patients with scoliosis can maintain spinal stability and balance of the torso, whether at rest or in motion, with the strength of the peripheral muscles of the spine (14). (3) The Lyon therapy, originating in France, is centered around maintaining proper spinal alignment during exercise. This approach involves lordosis of the lumbar spine and prioritising kyphosis of the thoracic region, alongside frontal segmental mobilisation, plane correction, proprioception, core stabilisation, stabilisation, and balance (15). (3) The Scientific Exercise Approach to Scoliosis (SEAS), developed in Italy, is focused on autocorrection and stabilization. Its exercises are designed to alleviate any identified impairments during the initial evaluation, such as weakness, muscle tightness, and poor motor coordination (16). (4) The technique of the Side Shift method in the UK is centered on intensive training in trunk flexion. This is an active self-correcting method, which involves instructing the patient to shift their trunk laterally over the pelvis in the direction contrary to the convexity of the principal curvature (17). (5) The DoboMed technique, which originated in Poland, concentrates on enhancing the thoracic kyphosis. This is achieved through closed kinematic chains, utilizing a pelvis and shoulder girdle placed symmetrically. Moreover, the corrected position is actively stabilized to establish it as habitual (18).

Physiotherapeutic Scoliosis-Specific Exercises (PSSE) and core stabilization exercises have been developed and utilised in multiple countries worldwide. Various other methodologies are also employed across several continents, but principles are akin to those of PSSE. Conservative treatment utilising exercise therapy has demonstrated the capacity to ameliorate quality of life and is deemed a crucial constituent of successful scoliosis rehabilitation with a view to favourable outcomes (19).

In the current situation of increasing demand for AIS rehabilitation, the increasing application of exercise therapy makes it a meaningful research field and show good prospects in the treatment of AIS. The focus of current research is primarily to address the clinical question of whether PSSE can effectively delay the progression or slow down the advancement of the curve. Preventing disease progression is essential to avoid the need for bracing, surgery or both (11). Numerous scholars have conducted bibliometric investigations on various aspects of AIS. Nevertheless, there has been no scientific research that has presented a comprehensive analysis of exercise therapy for rehabilitation of AIS through bibliometrics. A study presents an updated bibliometric analysis of scientific articles on AIS from 1985 to 2020. The analysis focuses on publication trends and the most influential articles in the field of AIS. It should be noted that the study did not examine the growing research on exercise therapy for scoliosis during this period (20). To address the lack of quantitative analysis in the research area, this bibliometric study seeks to gather worldwide scientific research on exercise therapy for scoliosis rehabilitation from inception to 2023. We conducted a comprehensive analysis of publications from the Web of Science Core Collection (WoSCC) database using Citespace and VOSviewer. The purpose of this article is to aid researchers and clinicians in understanding the prominent areas and emerging patterns within this field, which may enable high quality in clinical practice and future research.

## 2 Materials and methods

### 2.1 Source of bibliometric data and search strategy

**Figure 1:**
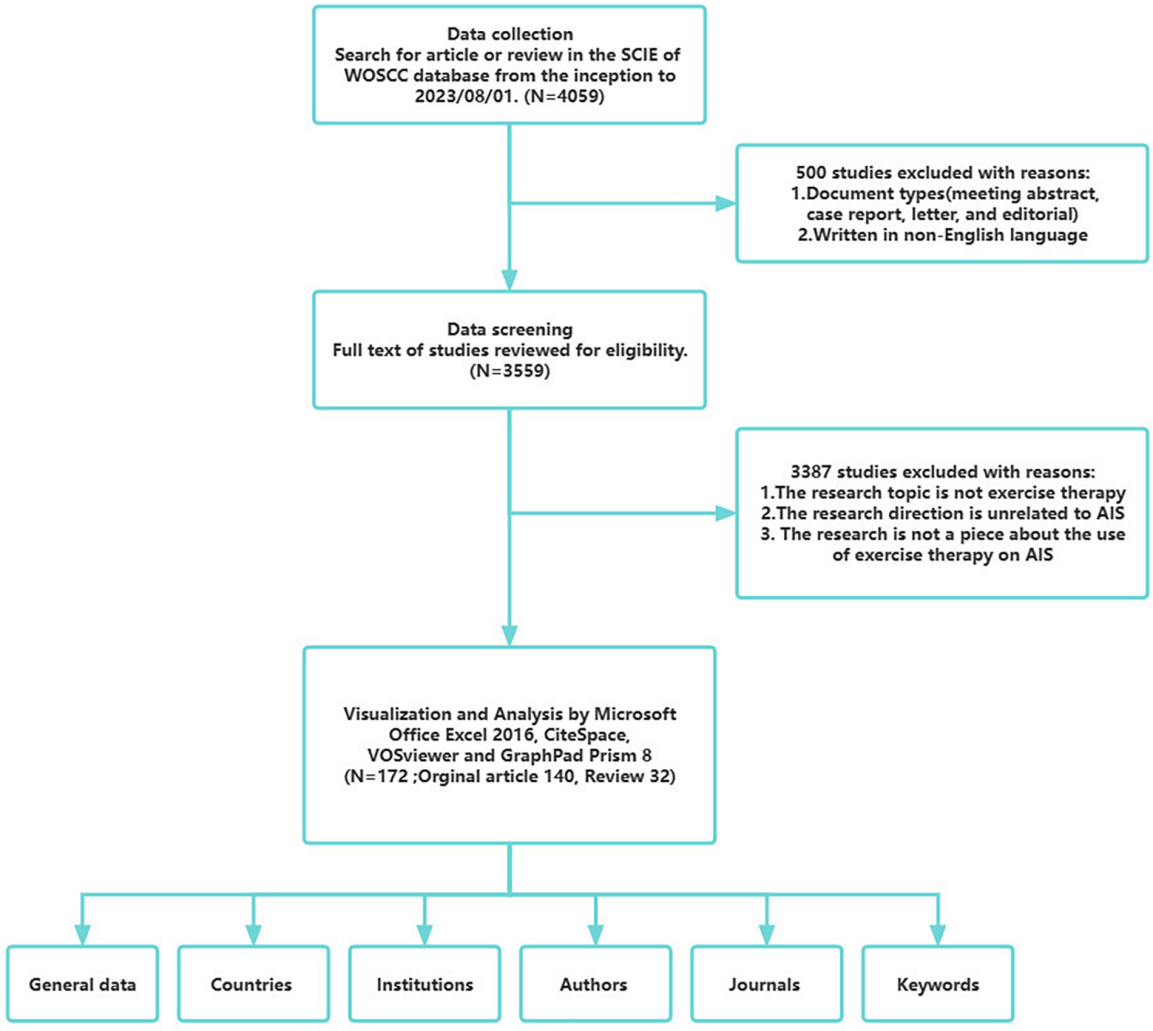
Flow chart of the bibliometric search and analysis process.

To ensure the coverage and authority of the data analyzed, Publications with related themes were searched from the Science Citation Index Expanded (SCIE) within the WoSCC,with a time span starting from the inception to 2023, a search time cutoff of 1st August 2023. Two authors searched independently, and Supplementary Material S1 showed the search strategy of the study. Only English language articles or reviews were selected. All documents included had to be peer-reviewed. Data must be pre-processed prior to analysis to avoid the results of the analysis being influenced by the quality of the data itself. Data from all articles relevant to the bibliometrics were imported into Zotero, and titles, abstracts, and entire texts of the included papers were then independently screened by two researchers to identify usable studies based on predetermined exclusion criteria. Exclusion criterias were including: (1) the intervention modality is not exercise therapy; (2) the target conditions are not associated with AIS; (3) the implementation of exercise therapy for AIS is not the subject of the paper. In the end, 172 valid documents were obtained. Figure 1 shows the process of searching and analysing bibliometric data.

### 2.2 Data extraction and analysis

Available documents were manually selected from the WOS database after screening and review using Zotero. Then, VOSviewer 1.6.19.0 and Citespace 6.2 R4 were used to analyse the plain text downloaded from the Web of Science database containing information from these files in order to map and visualise the bibliometric network of scientific publications (Supplementary Material S2). Basic data were gathered and co-occurrence and co-citation maps by country, institution, author and journal were created employing VOSviewer and Microsoft Excel. The number of published articles is represented by the size of the nodes in the VOSviewer co-occurrence graph, and the level of collaboration is indicated by the connecting lines between the nodes. The node cluster is depicted by the circle colour. Additionally, this study utilised Citespace to analyse the keyword co-occurrence network, burst keyword and cluster network analysis, revealing current innovative research tendencies. Furthermore, GraphPad Prism 8 was utilised to display the quantity of publications and the extent of collaboration amongst diverse nations.

## 3 Results

### 3.1 Publication outputs and study trend

**Figure 2:**
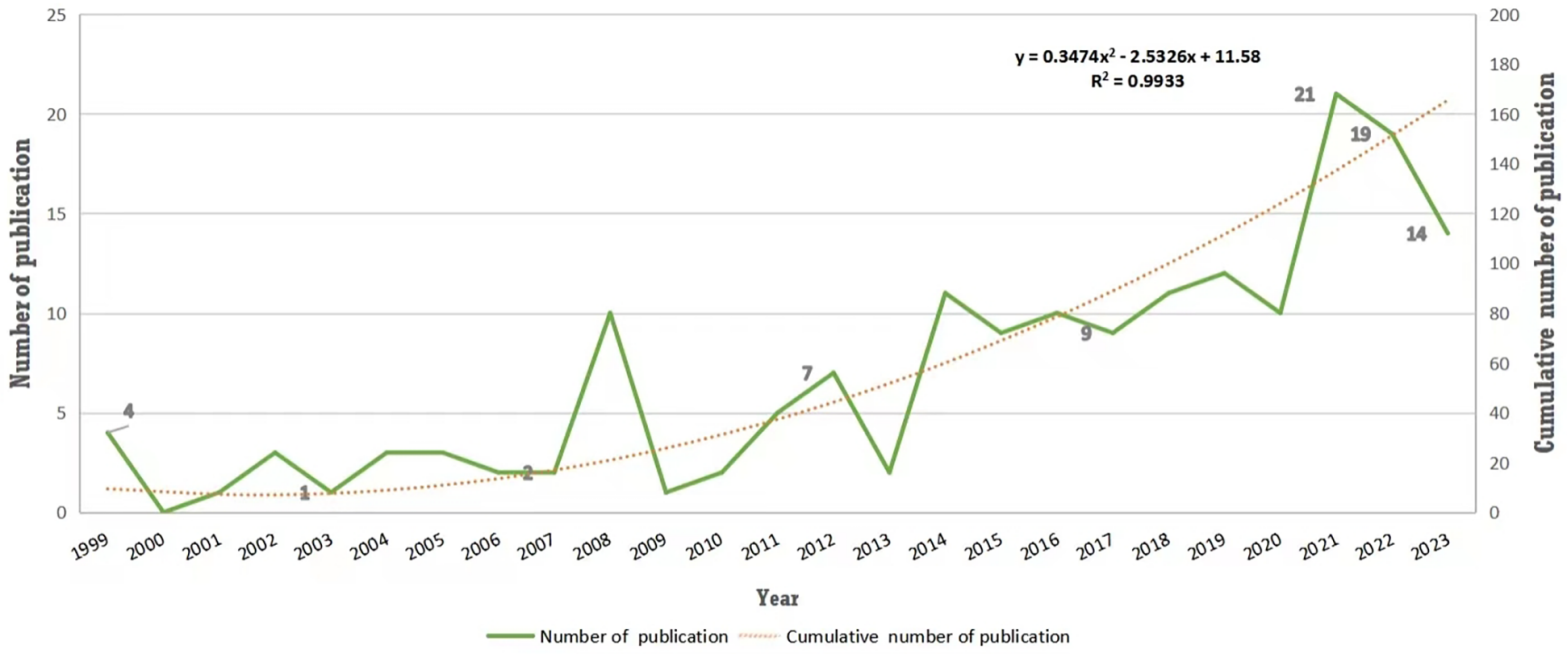
Trend of publication outputs from 1999 to 2023 on exercise therapy for AIS rehabilitation topic.

The initial search of the WoSCC database revealed 4,059 publications. After screening out non-relevant documents, those in languages other than English, and research topics that were unrelated, a total of 172 papers were selected for the analysis. These papers comprised of 140 articles, consisting of 29 randomized controlled trials (RCTs) and 111 non-RCTs, in addition to 32 reviews. These papers were published between 1999 and 2023. In 1999, Boer et al. published the initial study concerning exercise therapy for rehabilitation of AIS (17), which yielded clinical evidence that supports the employment of Side Shift method on patients with AIS. Figure 2 presents an analysis of the bibliometric trends and the annual numbers of the 172 publications, which can be classified into three phases: the infancy phase (1999–2007), the ups and downs phase (2008–2013), and the rapid growth phase (2014–2023). In its infancy, annual publication remained below 4 except 1999. Over the ups and downs phase, there were small peaks in 2008 and 2012, respectively, and declines the following year. During the rapid growth phase, the number of publications increased significantly, with over 13 papers being released every year. Almost 50% of the publications were published in the final phase, with a maximum in 2021 (*n* = 21). Moreover, according to the linear regression analysis, there was a positive correlation observed between the volume of publications and the year of publication (*R*^2^ = 0.9933, *p* < 0.001). It is anticipated that research in this field will persist in growing in the future.

### 3.2 Active countries

**Figure 3:**
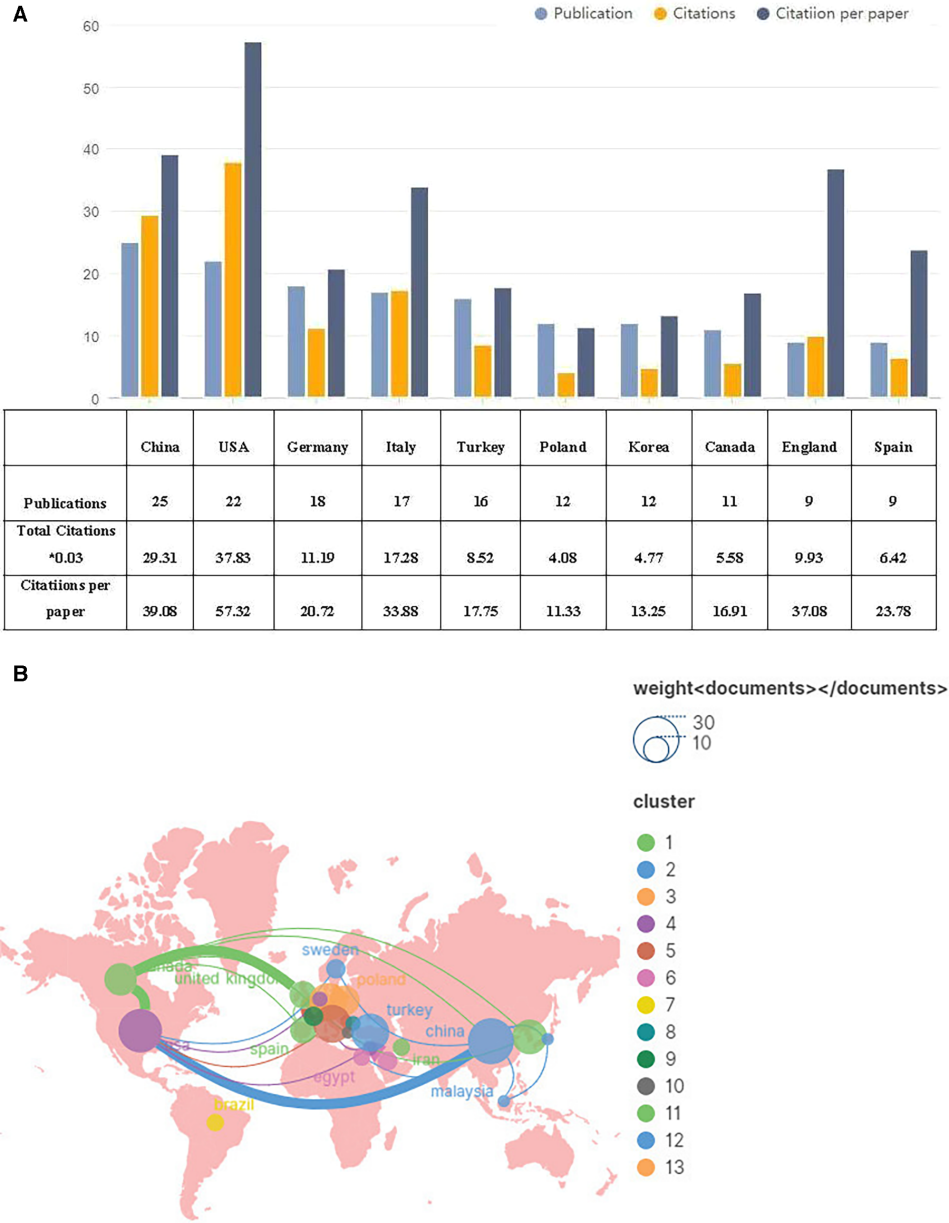
The top 10 prolific countries and international collaboration network on exercise therapy for AIS rehabilitation research. (**A**) The number of publications, total citations, and citations per paper in the top 10 countries. (**B**) The co-operative network visualization map of countries.

Thirty-three countries participated in publications on exercise therapy for AIS rehabilitation. Figure 3A displays the top 10 countries that produced nine or more papers. The five most engaged nations were China (25, 14.5%), USA (22, 12.8%), Germany (18, 10.5%), Italy (17, 10%), and Turkey (16, 9%). Of the 10 nations with the highest publication output, China and Turkey are the only classified as developing, while the remaining eight are classified as developed. The most cited research came from the USA, with 1,261 citations, followed by China (977) and Italy (576). Regarding citations per paper, the USA ranked first with 57.32 citations, followed by China (39.08) and England (37.08). Geographical information from the papers analyzed was extracted with the use of VOSviewer and imported into Scimago Graphica. This process resulted in the creation of a collaborative network between all countries depicted in Figure 3B. The results show that there are three main clusters of countries, namely Western Europe, Eastern Asia and North America. The closest collaboration was observed to be between China and the USA.

### 3.3 Institution distributions

**Figure 4:**
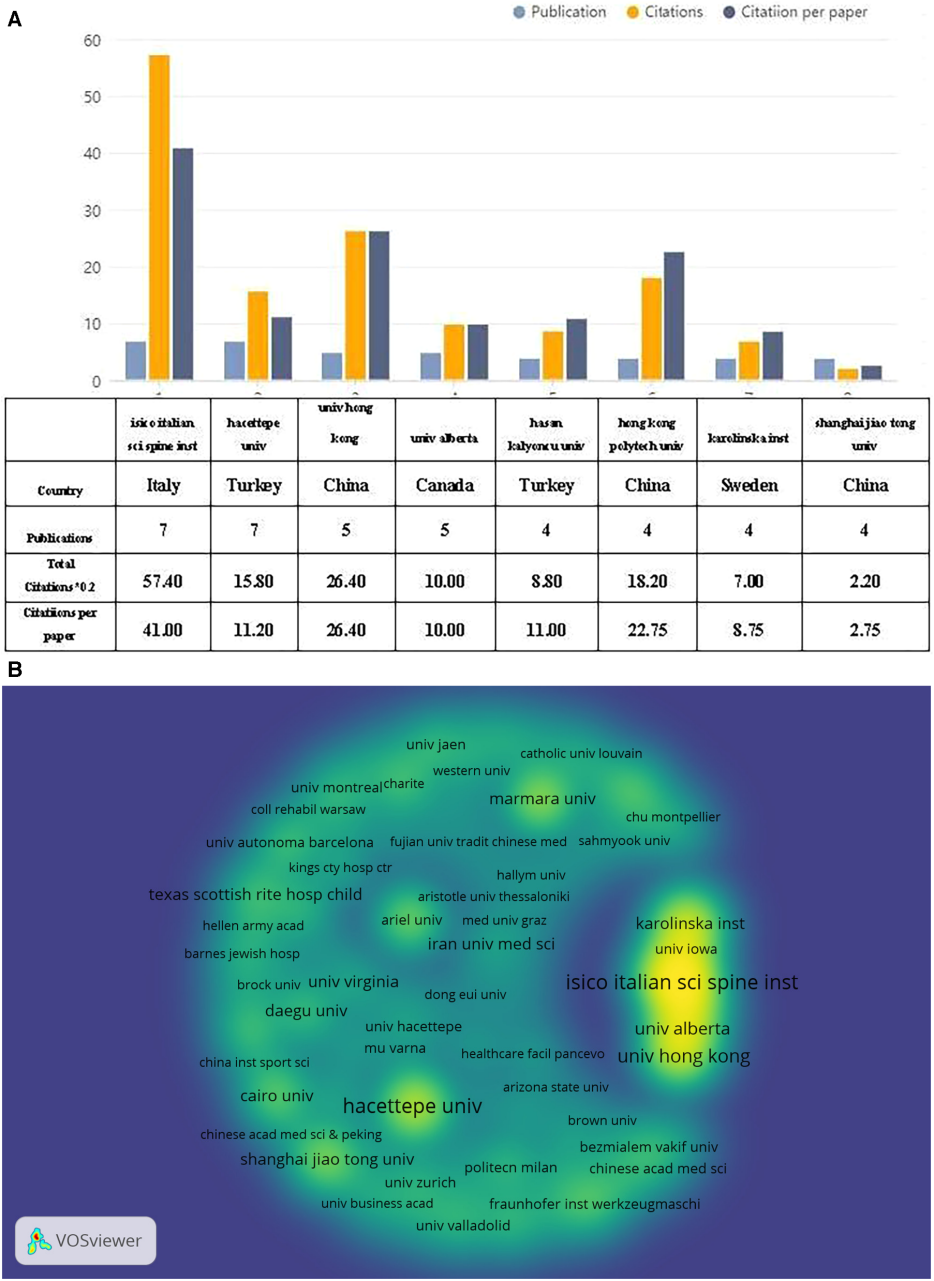
The top 8 active institutions and the inter-institutional collaboration network on exercise therapy for AIS rehabilitation research. (**A**) The number of publications, total citations, and citations per paper in the top 8 institutions. (**B**) The density visualization map of institutions.

According to the author's address, 313 institutions made contributions to the 172 publications. Figure 4A shows the 8 institutions that were the most productive in publishing over 4 papers. Hacettepe University (Turkey) and the Italian Scientific Spine Institute (Italy) were the most prolific, both publishing 7 papers. The University of Hong Kong (China) followed with 5 papers. The research papers from the Italian Scientific Spine Institute garnered the greatest number of citations (287) and citations per paper (48). Figure 4B displays the collaborative network between prominent institutions engaged in research on exercise therapy for AIS rehabilitation. The top organisations had significant links with remaining organisations, with the yellow block representing the density of collaboration between institutions. Closer collaboration was observed between various institutions, specifically the Italian Scientific Spine Institute, University of Alberta, University of Hong Kong, and Karolinska Institutet.

### 3.4 Author analysis

**Figure 5:**
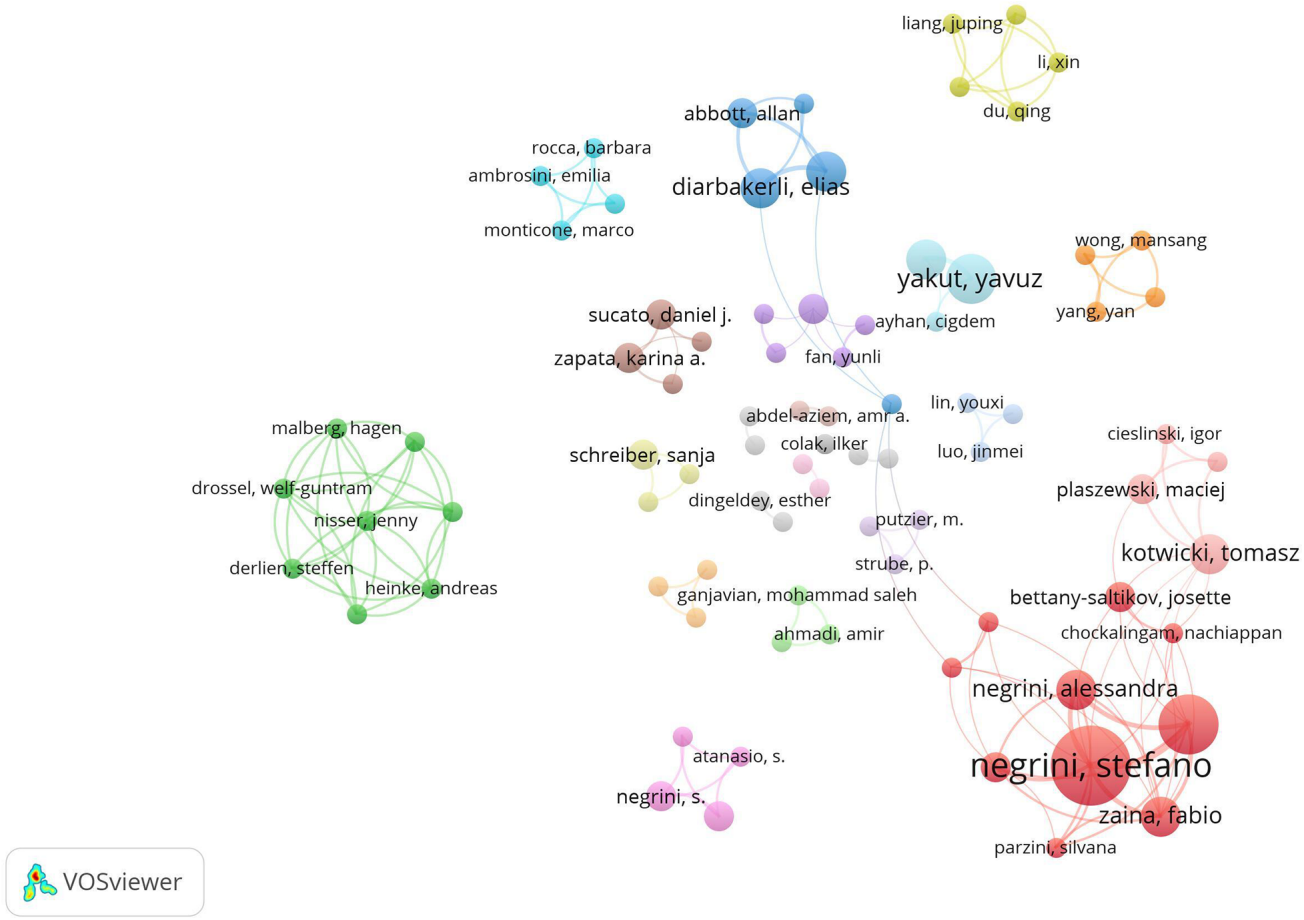
Network map of active authors contributed to exercise therapy for AIS rehabilitation research.

Overall, 735 authors contributed to the literature on rehabilitation exercise for AIS. Table 1 presents the details of the 10 most prolific authors. The top 3 authors with the highest number of publications were Negrini (11 papers), Romano (9 papers), and Yakut (5 papers). Five of the top ten authors were associated with the Italian Scientific Spine Institute, whereas the remaining authors worked in different research units. Table 1 demonstrates that the Italian Scientific Spine Institute ranked first in terms of the total number of citations and citations per paper, with 287 citations and an average of 47 citations per paper. Furthermore, the author's academic achievement could be accurately reflected by the H-index. Negrini Stefano is ranked first on the H-Index and has the most significant influence in this field (21). An overlay visualization map is exhibited in Figure 5, created by VOSviewer, which analyses author co-occurrence. The graph depicts a prominent cluster centered on Negrini Stefano with a strong collaboration between him and other authors. The remaining authors are situated in smaller groups.

**Table 1:** The top 10 active authors who published literature on exercise therapy for AIS rehabilitation.

Rank	Author	Institution	Country	Publications	Citations	Citations per paper	H-index
1	Negrini, Stefano	Italian Scientific Spine Institute	Italy	11	421	38.27	43
2	Romano, Michele	Italian Scientific Spine Institute	Italy	9	370	41,11	25
3	Yakut, Yavuz	Hasan Kalyoncu University	Turkey	5	76	15.20	19
4	Zaina, Fabio	Italian Scientific Spine Institute	Italy	4	185	46.25	33
5	Negrini, Alessandra	Italian Scientific Spine Institute	Italy	4	89	22.25	11
6	Diarbakerli, Elias	Karolinska University Hospital	Sweden	4	15	3.75	9
7	Gerdhem, Paul	Uppsala University Hospita	Sweden	4	15	3.75	33
8	Kotwicki, Tomasz	Poznan University of Medical Sciences	Poland	4	88	22.00	26
9	Yagci, Gozde	Hacettepe University	Turkey	4	50	12.50	8
10	Donzelli, Sabrina	Italian Scientific Spine Institute	Italy	3	40	13.33	15

### 3.5 Journal characteristic

**Figure 6:**
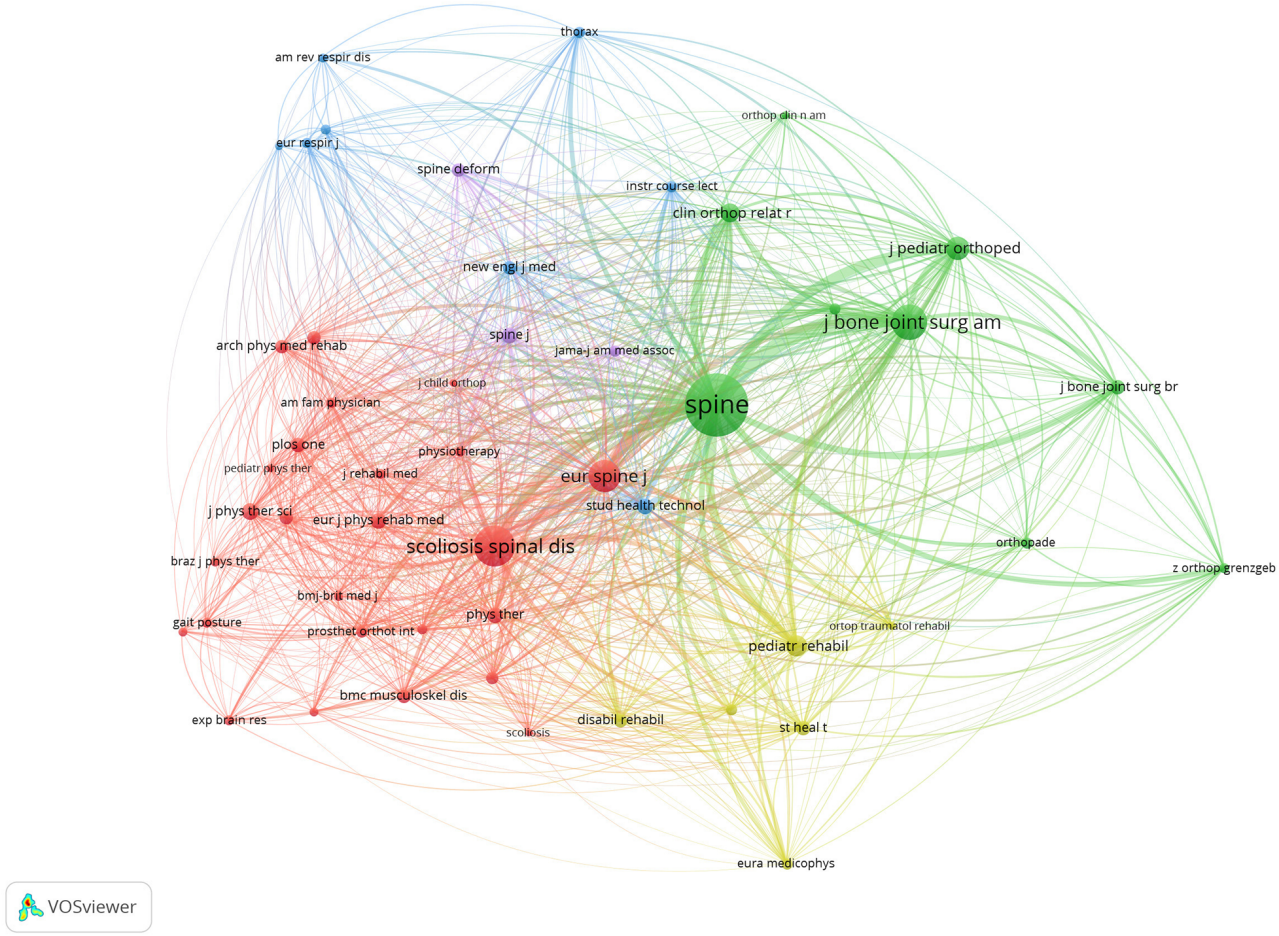
Co-citation network map of journals.

The publications included in this study were published in a total of 79 academic journals. Following Bradford's law, those journals that produced over a third of all related papers were identified as core journals, revealing a total of 73 non-core journals and 6 core journals of this field of research. As can be seen in Table 2, the top ten journals with the highest productivity comprised 44.77% (77 articles) of the entire number of research papers. Spine published the most papers (18 papers), followed by European Spine Journal (9 papers) and Journal Of Back And Musculoskeletal Rehabilitation (9 papers). As far as the impact factor (IF) of the journals is concerned, none of the top 10 journals had an IF > 5,000. Seven journals had an IF between 2,000 and 5,000, two journals had an IF < 2,000, and one journal had no current IF. VOSviewer generated a co-citation map of 21 journals that received a minimum of 50 citations. Among these, the most frequently co-cited publications were Spine (3.50), Scoliosis and Spinal Disorders (N/A), and Journal of Bone and Joint Surgery-American Volume (5.30). These journals are notable and respected in the field, as depicted in Figure 6.

**Table 2:** The top 10 most productive journals in the exercise therapy for AIS rehabilitation field.

Rank	Journal	Publications	Citations	Citations per paper	IF	JCR	OA
1	Spine	18	408	22.67	3.50	Q1	No
2	European Spine Journal	9	252	28.00	3.20	Q1	No
3	Journal of Back and Musculoskeletal Rehabilitation	9	84	9.33	1.70	Q2	No
4	Journal of Physical Therapy Science	8	91	11.38	/	/	No
5	PLoS One	7	86	12.29	3.80	Q1	Yes
6	European Journal of Physical and Rehabilitation Medicine	6	179	29.83	4.50	Q1	No
7	Prosthetics and Orthotics International	5	79	15.80	2.10	Q2	No
8	Physiotherapy Theory and Practice	5	23	4.60	2.00	Q2	No
9	Children-basel	5	4	0.80	2.60	Q3	Yes
10	Orthopade	5	26	5.20	1.00	Q3	No

### 3.6 Analysis of keywords

**Figure 7:**
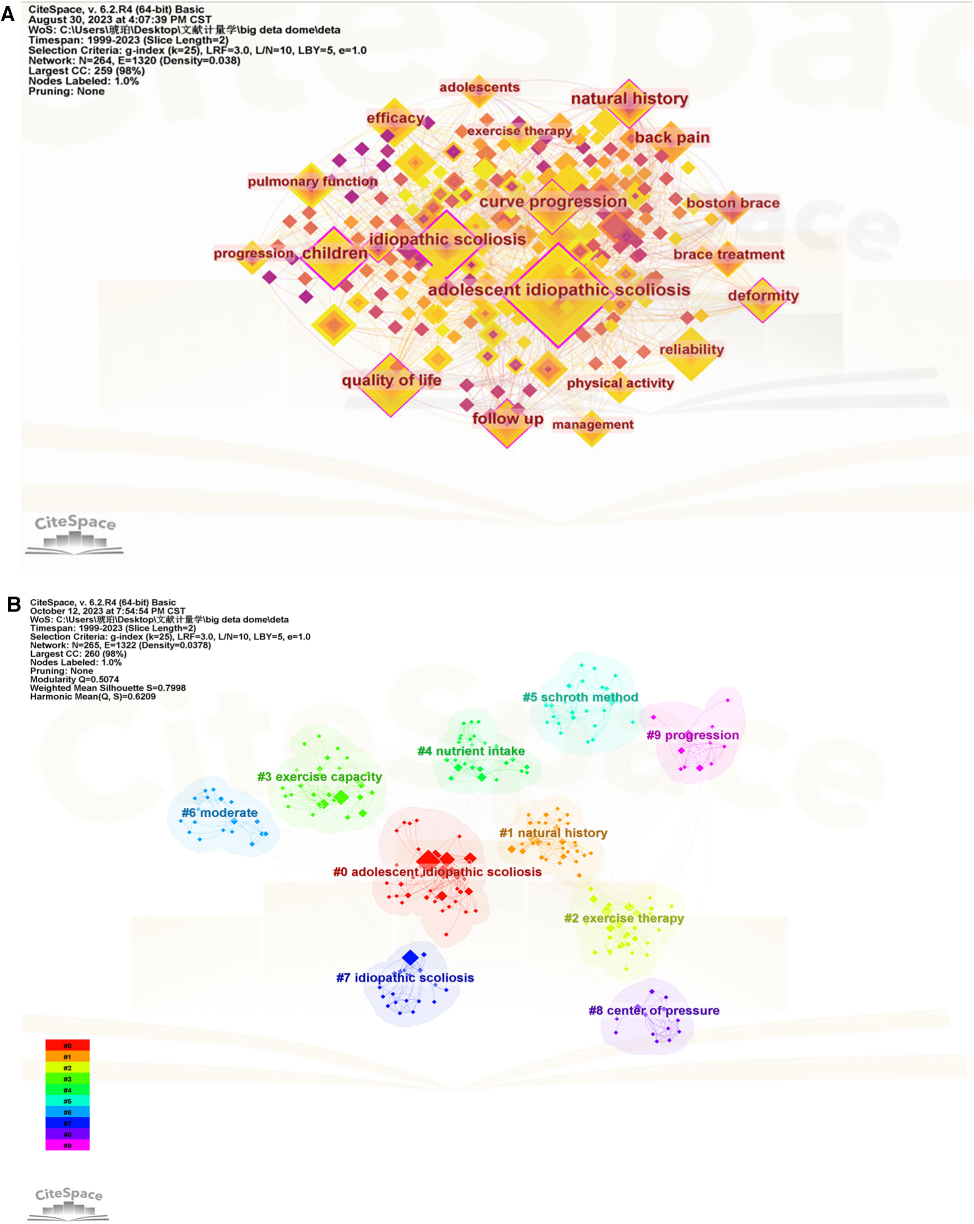
Analysis of keywords related to publications on exercise therapy for AIS rehabilitation field. (**A**) The keyword co-occurrence network map. (**B**) The keyword cluster map. (**C**) The top 25 keywords with the strongest citation bursts.

Keywords provide a concise summary of a paper by reflecting current themes and predicting future research frontiers through high frequency or burst keywords. As demonstrated in Figure 7A, the three keywords with the highest frequency were adolescent idiopathic scoliosis, idiopathic scoliosis, and curve progression. The keywords could be classified into eleven distinct clusters based on their type, as demonstrated in Figure 7B. Clusters #0, #7 mainly described the target patients. Clusters #2 and #5 indicated the different types of exercise therapy, including Schroth method. Clusters #4 shows another conservative treatment. Clusters #1, #3, #4, #8 and #9 primarily investigated the potential correlations between idiopathic scoliosis and various influencing factors, while other groups of factors were examined in relation to different stages of the disease's development. Furthermore, Citespace has generated the top 25 most significant keywords with the strongest burst, as illustrated in Figure 7C. In terms of burst time, the keywords have been segmented into three periods: 1999–2007, 2008–2013, and 2014–2023. Schroth exercises and reliability indicate the highest burst strength among these keywords. Spinal deformity, physical activity, Schroth exercises, and Schroth have been identified as emerging keywords, indicating potential research focuses in the coming future.

**Figure:**
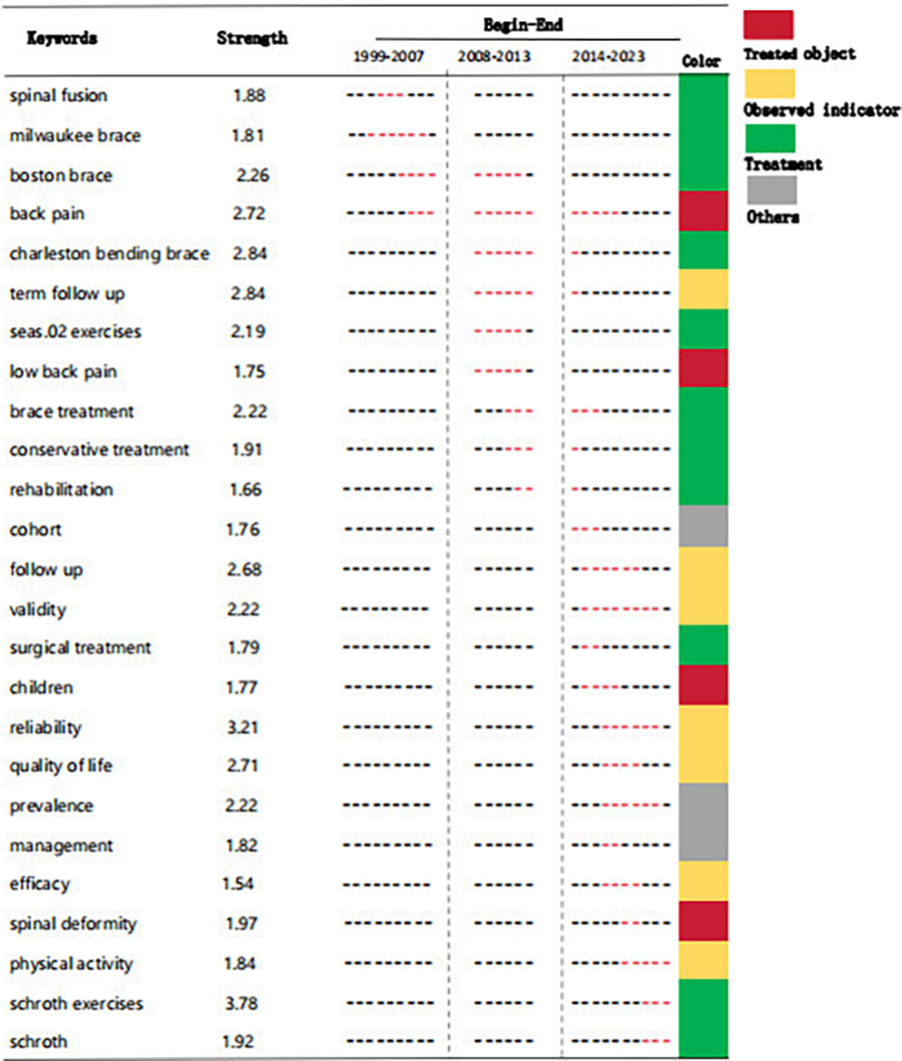


## 4 Discussion

### 4.1 General overview of the results

In this bibliometric analysis, we analysed 172 papers examining exercise therapy as a means of rehabilitating AIS, using Citespace and VOSviewer software to identify research priorities and trends in this area. The literature pertaining to this subject could exhibit fluctuations in research activity and output, divided into three discrete stages. Before 2007, a total papers obtained slightly changed. Exercise therapy has not been extensively utilized in the medical field because of research limitations. Previous studies have shown that surgeons have conducted the majority of research on scoliosis, with a primary focus on the effects of surgical treatments. As a result, there is not enough evidence for non-surgical interventions, and the limited number of available trials have a low level of quality (22). Throughout the 2010 s, numerous studies have highlighted limitations within brace therapy when compared with exercise therapy. As a result, the number of papers examining this issue has gradually gained momentum, drawing the interest of medical practitioners and researchers alike. Since 2020, there has been a significant increase in the number of annual publications, reaching 21 in 2021. This increase can be attributed to the intricate nature of scoliosis surgical treatment, which incurs a rather high expense, along with a complications risk. Therefore, the pursuit of viable alternatives remains active. Over the last decade (Phase III), the number of publications on exercise therapy for AIS has steadily increased, with a significant rise in total papers compared to the previous two phases. This suggests that the popularity of this area of research will continue to grow in the coming future.

In terms of researchers, more than one-fifth of countries globally have published on exercise therapy for AIS rehabilitation. China, the USA, Italy, Germany, and Turkey have dominated this field and have also been driving factors in other areas of scoliosis researches (23). It is likely due to the substantial national gross domestic product (GDP) that can offer ample resources on clinical study, and the parents of teenage patients prioritise a holistic treatment strategy aimed at conservative management (24). Around 30% of the top 10 research institutions are situated in China, with Italy (*n* = 2, 20%), Turkey (*n* = 2, 20%), Canada (*n* = 1, 10%), and Sweden (*n* = 1, 10%) following behind. We observed a close collaboration between five nations: the USA, China, Italy, Germany, and Canada. Additionally, Canada engages in active collaborations with Spain, China, Germany, and the USA. When it comes to research institutions, several of them maintain a productive and collaborative relationship, such as Italian Scientific Spine Institute, Karolinsika Institute, Alberta University, Hong Kong University, University of Lowa. However, we found that despite publishing the highest number of papers, Hacettepe University had limited collaboration with other institutions, which could hinder the long-term expansion of scholarly investigation. Although there are some cooperative relationships between specific nations, collaboration between institutions is lacking in extent and scope. Merely minimal cooperation has been observed between institutions located in China and Turkey, which may impede the advancement of the research field in the long term. As a result, we highly recommend that research institutions across different nations collaborate and communicate extensively to promote the growth of exercise therapy for the rehabilitation of AIS.

From the perspective of the author, Negrini stefano, Romano Michele, Yakut Yavuz, Zaina Fabio and Negrini Alessandra have been noted for publishing the most articles, with an average of 7 papers per person. Half of the top 10 researchers listed in Table 1 were associated with the Italian Scientific Spine Institute, Italy. However, their number of citations was lower than those affiliated with North American institutions. One possible explanation for this may be the fact that Italian researchers predominantly produced research papers in the post-2015 period, with limited citations. In addition to these findings, further insights into a particular field could be obtained by analyzing core journals that have a high publication rate. Over one-third of the total quantities of papers were published in the leading 10 journals, which implies that research articles on exercise therapy for AIS rehabilitation were concentrated in these selected journals. Moreover, the active journals in this study did not achieve a high impact factor, indicating that no journal reached a score of 5. Therefore, there is a need for enhancing the standard and quality of research in exercise therapy for AIS rehabilitation. This necessitates international collaboration among authors to conduct clinical studies of high quality.

### 4.2 Hotspots analysis

The author clusters could help to identify historical, current, and future focal points within a particular field. As illustrated in Figure 5, a cohort of authors have contributed to the publication of 172 papers, with Negrini Stefano assuming the primary leadership role. The team of Negrini Stefano is concentrated on rehabilitation treatment of idiopathic scoliosis during growth, especially exerise therapy (25, 26). According to previous research, although AIS is diagnosed and treated globally, there are international variations in conservative treatment approaches. The typical approach in North America is to initially monitor their condition before considering bracing if the curvature worsens beyond 25°, for patients with remaining growth (27). Exercise therapy, comprising of both outpatient and inpatient rehabilitation, is recognized as the foremost treatment approach for minor spinal curves and individuals with a low risk of progression, a recommendation consistently emphasized by a plethora of clinicians, primarily based in Europe (28). Exerise therapy has shown to be effective in the rehabilitation of AIS in that exercise can reduce scoliosis progression in AIS patients, enhance the control of spinal nerve movement, play a positive role in cardiopulmonary function, and regulate psychological problems such as depression and anxiety (9). Bettany-Saltikov (15) has reported on the controversial use of exercises as a means of treating AIS. While there is a shortage of high-quality research that supports the efficacy of PSSE in the treatment of AIS, current evidence suggests that PSSE aids in stabilising spinal deformities, enhancing patients' life quality, functionality, disability, and pain, aesthetically improving deformities, and delaying progression. Nevertheless, there is inadequate proof to confirm the superiority of one physiotherapy technique over another. Further robust research is required before advocating the use of PSSE in clinical settings.

References displaying citation bursts indicate emerging subjects in a specific research area, due to the fact that these references have been frequently cited by researchers over the last few years (29). It is apparent that the prevailing areas of interest in the field of exercise therapy for AIS rehabilitation include investigating the therapeutic effects of exercise therapy and the application of PSSE for treating AIS, based on the primary research outlined in highly cited references (Table 3). These PSSE physiotherapy contain Schroth, Side Shift, Barcelona Scoliosis Physical Therapy School (BSPTS), SEAS, Functional Individual Therapy of Scoliosis (FITS) and Lyon, Dobomed, respectively.

**Table 3:** The main research contents of the 15 references with strong citations bursts.

Rank	Strength	Main research content
1	3.57	The status quo of physical exercises as a treatment for adolescent idiopathic scoliosis.
2	4.6	Orthopaedic and Rehabilitation treatment of idiopathic scoliosis during growth guidelines.
3	4.4	The advances of physical exercises in the treatment of adolescent idiopathic scoliosis.
4	6.15	Discuss in detail seven major scoliosis schools and their approaches to Physiotherapy Scoliosis Specific Exercises to improve the conservative management of patients with idiopathic scoliosis.
5	5.36	A double-bilnded randomized controlled clinical trial study active self-correction and task-oriented exercises reduce spinal deformity and improve quality of life in subjects with mild adolescent idiopathic scoliosis.
6	4.57	The status quo of exercises for adolescent idiopathic scoliosis.
7	4.15	SEAS (Scientific Exercises Approach to Scoliosis): a modern and effective evidence based approach to physiotherapic specific scoliosis exercises
8	4.08	A single-bilnded randomized controlled clinical trial study the efficacy of three-dimensional Schroth exercises in adolescent idiopathic scoliosis.
9	4.06	A double-bilnded randomized controlled clinical trial study the effect of Schroth exercises improve the quality of life and muscle endurance in adolescents with idiopathic scoliosis.
10	3.64	A double-bilnded randomized controlled clinical trial study Schroth Physiotherapeutic Scoliosis-Specific Exercises lead to better cobb angle outcomes in adolescents with idiopathic scoliosis.
11	3.49	The current state of exercise protocols for adolescent idiopathic scoliosis.
12	2.9	Introduces the different “Schools” and approaches of physiotherapeutic scoliosis-specific exercises currently practiced and discusses their commonalities and differences.
13	5.68	Guidelines for the management of idiopathic scoliosis during growth in orthopaedic and rehabilitation settings.
14	4.22	A meta-analysis study effects of the Schroth exercise on idiopathic scoliosis
15	3.11	A single-bilnded randomized controlled clinical trial study core stabilization exercises versus scoliosis-specific exercises in moderate idiopathic scoliosis treatment.

### 4.3 Keywords and trend analysis

The analysis of keyword co-occurrence and burst clusters could aid in swiftly capturing the distribution and progression of hotspots within the domain of exercise therapy for AIS rehabilitation. Based on the results of the clustering analysis, it can be inferred that exercise therapy primarily targeted children who suffer from spinal deformities and low back pain. Furthermore, apart from the reliability and validity of stabilizing spinal deformity short-term and in long-term follow-up, further investigations have been carried out to examine the advantages of exercise therapy on the quality of life and children's performance in physical activities, and Schroth has been extensively researched and shown to be a successful treatment (15). As depicted by Figure 7C, the theme explored in the studies included in this research underwent three distinct stages of variation: Phase I (1999–2007), Phase II (2008–2013), and Phase III (2014–2023).

The literature from 2007 and before focussed primarily on brace rehabilitation for AIS, with less content directed toward exercise therapy. Since 2008, there has been a growing interest among researchers worldwide in adapting exercise therapy to enhance the condition of patients with AIS who are experiencing low back pain. In less severe cases, exercise can be the primary form of treatment while in more severe cases, it can be used as a supplementary treatment (30).

Following the emergence of exercise therapy in the 2014 s, various forms of exercise therapy (PSSE) and exercise-related proper nouns (Schroth exercises) have become available with an enhanced and more uniform research layout. In last 3 years, studies have predominantly confirmed the effectiveness of exercise therapy in treating patients with AIS through high-quality RCTs, exemplifying the current cutting-edge approach to AIS rehabilitation through exercise therapy.

### 4.4 Summary of exercise therapy intervention

The agreement that physical therapy can steady or decrease the magnitude of a spinal malformation or ameliorate functional levels in AIS has been established in recent decades (31, 32). Still, some barriers remain for exercise therapy to be successfully applied: (1) There remain several cognitive limitations to be addressed in order to comply with clinical standards and personalized treatment, particularly as many healthcare professionals lack expertise in distinguishing between generalized physiotherapy exercises and PSSE (33, 34). (2) Few studies on exercise therapy have prospectively registered their study protocols. This is expected to be replenished in future research in order to improve methodological standardization and rigor. Exercise therapy research should conform to standard reporting guidelines. This includes the prospective registration of detailed protocols and the use of suitable labelling for exercises, Schroth classification and therapists who are accredited. In addition, exercise names and descriptions should be provided according to their classification, along with details of therapy dosages, prescription methods and adherence (35, 36). (3) In the clinical practice of exercise therapy for the treatment of AIS, research has found that core stabilization exercises has a beneficial impact on the Cobb angle, trunk rotation, and quality of life for individuals with idiopathic scoliosis. The Schroth method has a bigger impact size than do core stabilization exercises. Additionally, these two approaches are compatible and can be combined to achieve better treatment outcomes for AIS patients in the future. While there appears to be clinically significant changes resulting from studies examining the effectiveness of certain PSSE exercise therapies and core stabilization exercises, the scientific validity of the present evidence remains inadequate. Studies of the DoboMed, FITS and Lyon methods have found similar limitations. The examined therapies were discovered to possess a poor overall standard of evidence for the Cobb angle, and an extremely low standard of proof for spine rotation angle and quality of life. To determine the real efficacy of PSSE physiotherapy as a treatment for mild to moderate AIS patients compared to no treatment, further randomised controlled trials are necessary. Furthermore, additional research is required to establish the optimum types of PSSE for varying curve types, as well as the most efficient methods (frequency and intensity) available (37–39). (4) Exercise compliance and follow-up investigation variables should also be considered when designing the subsequent study. Furthermore, future research should comprises methods for objectively monitoring exercise adherence and motor learning, such as group supervised exercise sessions, which is combined with telerehabilitation. A study has demonstrated that patients significantly favoured the weekly digital REDCap survey is preferred to the paper log (40, 41). Exercise therapy has been considered helpful in reducing scoliosis progression in AIS patients, enhancing the control of spinal nerve movement, and playing a positive role in cardiopulmonary function, even regulating psychological problems such as depression and anxiety (42). The results outlined in our paper offer valuable insights and encourage further investigation into the field of interest in order to facilitate more studies and clinical applications.

### 4.5 Strengths and limitations

Bibliometric studies of various aspects of AIS have been carried out by numerous scholars. One study summarised information published in scoliosis-related literature over a 10-year period from 2009 to 2018. A study summarized the publication information of scoliosis-related literature in the 10 years from 2009 to 2018. It analyzed former research hotspots in the field of scoliosis and predicted future areas of interest. Exercise therapy was not mentioned in the study, reflecting the fact that exercise therapy was not a hot topic in the overall scoliosis research field during this decade. Another bibliometric analysis includes the 100 most cited articles on idiopathic scoliosis (43). The results indicate that the evolution of the knowledge on idiopathic scoliosis has been through case reports and case series. The study did not address exercise therapy for scoliosis because of the relatively weak impact of exercise therapy-related articles in the overall field of scoliosis research (44, 45). Our study is the first review to summarise, from a bibliometric point of view, the ongoing publications and the forthcoming patterns of progression of exercise therapy for AIS, which can effectively guide researchers who express an interest in related research. To conduct a thorough and comprehensive evaluation, from the WoSCC database, this study gathered 172 relevant papers published in the last 24 years. Additionally, we utilised widely accepted bibliometric software, VOSviewer and Citespace, to conduct a quantitative assessment of exercise therapy in the area of AIS incorporating country, institution, author, journal, citation and keyword specific data.

Nevertheless, we need to acknowledge that we have failed to deliver. Firstly, the study's data was restricted solely to the WOS database. Thus, incorporating other databases such as PubMed and Scopus could yield a higher number of published documents and more comprehensive results. Secondly, we only included studies published in English. This may have resulted in an underestimation of papers published in non-English languages. Thirdly, the formulation of our study's strategy in this study was primarily based on a broad topic (TS), which may have resulted in the inclusion of irrelevant papers, ultimately causing some bias in the final results. It is possible that we have overlooked several studies on exercise therapy research if the authors did not explicitly state our inclusion criteria in the article topic. Finally, this study aims to provide an accurate representation of the current state of research in this field. Further systematic reviews or high-quality trials with a focused scope are warranted to refine both the specific therapeutic effects and applied techniques of exercise therapy that are necessary for rehabilitation following AIS, and to assist in the formation of clinical guidelines.

## 5 Conclusion

This bibliometric study offers a comprehensive examination of research pertaining to exercise therapy for AIS rehabilitation. Exercise therapy have important application prospects and research value in AIS rehabilitation. Over the last 24 years, there has been a notable increase in the quantity of research conducted in this field. China, Western Europe and North America have proved to be major contributors in terms of publications and overall citations received. However, there is still a need to improve the co-operation and communication between the different countries and institutions. Many journals possess a low Impact Factor, which calls for further consideration in the future. The co-occurrence analysis conducted by Negrini Stefano identified a predominant cluster focusing on the rehabilitation treatment of idiopathic scoliosis during the growing phase. Notably, in addition to expand study design, containing bigger numbers, multi-site institution studies, we should also pay attention to the restrict to factors that contribute to different risks of progression, that is, the inclusion of similar curves. These findings may enable future researchers to gain a deeper understanding of the current key areas and developments.
