# Supplementary material for: Exercise therapy for adolescent idiopathic scoliosis rehabilitation: a bibliometric analysis (1999–2023)
Source: Front Pediatr. 2024 Jan 4;11:1342327. doi: 10.3389/fped.2023.1342327 (PMC10794515; doi:10.3389/fped.2023.1342327)
Supplement: Supplementary file 1 [file Datasheet1.pdf]

Supplementary Material

Search Strategy:

1. Database: Web of Science core collection (to 31th July 2023).

#1 TS=((AIS) OR ("adolescent idiopathic scoliosis")OR(scoliosis)OR("idiopathic scoliosis")) (37040)

#2 TS=(((exercise*)OR("conservative treatment")OR("scoliosis specific exercise*")OR(physiotherapy)OR("Physical Activit*")OR("exercise therap*")OR("exercise movement technique*")OR("motor control exercise*")OR("motion therapy")OR("motor activity")OR(movement)OR(Training))OR((Schroth)OR((FITS)OR("Functional Individual Therapy of Scoliosis"))OR(DoboMed)OR(SEAS)OR("Side Shift")OR(FED)OR((BSPTS)OR("Barcelona Scoliosis Physical Therapy School"))OR(Lyon))) (4,063,053)

#3 #1 AND #2 (4059)
