# Supplementary material for: Exercise therapy for adolescent idiopathic scoliosis rehabilitation: a bibliometric analysis (1999–2023)
Source: Front Pediatr. 2024 Jan 4;11:1342327. doi: 10.3389/fped.2023.1342327 (PMC10794515; doi:10.3389/fped.2023.1342327)
Supplement: Supplementary file 2 [file Datasheet2.pdf]

Supplementary Material

FN Clarivate Analytics Web of Science

VR 1.0

PT J

AU Zapata, KA

McIntosh, AL

Jo, CH

Virostek, D

AF Zapata, Karina A.

McIntosh, Amy L.

Jo, Chan-Hee

Virostek, Donald

TI The Addition of Daytime Physiotherapeutic Scoliosis-specific Exercises

to Adolescent Idiopathic Scoliosis Nighttime Bracing Reduces Curve

Progression

SO JOURNAL OF PEDIATRIC ORTHOPAEDICS

LA English

DT Article

DE AIS; PSSE; Schroth; bracing

ID CRITERIA

AB Background:The effectiveness of physiotherapeutic scoliosis-specific exercises (PSSE) in addition to nighttime bracing compared with nighttime bracing alone is unknown. The purpose of this prospective comparative study was to evaluate the effectiveness of PSSE in skeletally immature patients with adolescent idiopathic scoliosis treated with nighttime bracing (PSSE group) compared with the standard of care of nighttime bracing alone (control group). Methods:Patients with adolescent idiopathic scoliosis thoracolumbar or lumbar primary curves <35 degrees at Risser stage 0 who wore a Providence brace were prospectively enrolled into the PSSE or control group. A

temperature sensor recorded the number of hours of brace wear. The PSSE group was instructed in the Schroth-based physical therapy method and a home exercise program for at least 15 minutes per day, 5 days per week, for 1 year. Results:Seventy-four patients (37 PSSE, 37 controls) were followed until the final visit of skeletal maturity or surgery. The PSSE and control groups had similar baseline Cobb angles (24 vs. 25 degrees) and average hours of brace wear (8.0 vs. 7.3 h). The PSSE group had no change in curve magnitude at the final visit compared with curve progression in the control group (1 vs. 7 degrees, P<0.01). Furthermore, the PSSE group had a lower rate of curve progression >5 degrees at the final visit (14% vs. 43%, P<0.01). The PSSE group also had less conversion to full-time bracing after 1 year (5% vs. 24%, P=0.046), but differences were no longer significant at the final visit (14% vs. 27%). Conclusions:In this prospective series of patients in nighttime Providence braces, the addition of Schroth-based physical therapy reduced curve progression after 1 year and at skeletal maturity. These findings can educate motivated families interested in PSSE.

C1 [Zapata, Karina A.; McIntosh, Amy L.; Jo, Chan-Hee; Virostek, Donald] Scottish Rite Children, Dallas, TX USA.

[Zapata, Karina A.] Scottish Rite Children, 2222 Welborn St, Dallas, TX 75219 USA.

RP Zapata, KA (通讯作者)， Scottish Rite Children, 2222 Welborn St, Dallas, TX 75219 USA.

EM karina.zapata@tsrh.org; amy.mcintosh@tsrh.org; chanhee.jo@tsrh.org;

don.virostek@tsrh.org

CR Buyuk AF, 2022, SPINE DEFORM, V10, P247, DOI 10.1007/s43390-021-00426-z

d'Amato CR, 2001, SPINE, V26, P2006

Gao A, 2021, CHINESE MED J-PEKING, V134, P2589, DOI 10.1097/CM9.0000000000001799

Harris PA, 2009, J BIOMED INFORM, V42, P377, DOI 10.1016/j.jbi.2008.08.010

Janicki JA, 2007, J PEDIATR ORTHOPED, V27, P369, DOI 10.1097/01.bpb.0000271331.71857.9a

Katz DE, 1997, SPINE, V22, P1302, DOI 10.1097/00007632-199706150-00005

Kwan KYH, 2017, SCOLIOSIS SPINAL DIS, V12, DOI 10.1186/s13013-017-0139-6

Ohrt-Nissen S, 2019, J ORTHOP SURG-HONG K, V27, DOI 10.1177/2309499019860017

Richards BS, 2005, SPINE, V30, P2068, DOI 10.1097/01.brs.0000178819.90239.d0

Roye BD, 2020, SPINE DEFORM, V8, P597, DOI 10.1007/s43390-020-00060-1

Ruffilli A, 2021, SPINE DEFORM, V9, P671, DOI 10.1007/s43390-020-00248-5

Tolo VT, 2020, SPINE DEFORM, V8, P149, DOI 10.1007/s43390-020-00036-1

Weinstein SL, 2013, NEW ENGL J MED, V369, P1512, DOI 10.1056/NEJMoa1307337

Wiemann JM, 2014, J PEDIATR ORTHOPED, V34, P603, DOI 10.1097/BPO.0000000000000221

Yagci G, 2019, PROSTHET ORTHOT INT, V43, P301, DOI 10.1177/0309364618820144

Yrjonen T, 2006, EUR SPINE J, V15, P1139, DOI 10.1007/s00586-005-0049-9

Zapata KA, 2019, SPINE DEFORM, V7, P870, DOI 10.1016/j.jspd.2019.02.012

Zapata KA, 2019, PEDIATR PHYS THER, V31, P280, DOI 10.1097/PEP.0000000000000621

Zapata KA, 2023, SPINE DEFORM, V11, P861, DOI 10.1007/s43390-023-00665-2

Zhou ZW, 2021, COMPLEMENT THER MED, V58, DOI 10.1016/j.ctim.2021.102697

NR 20

TC 0

Z9 0

U1 1

U2 1

PU LIPPINCOTT WILLIAMS & WILKINS

PI PHILADELPHIA

PA TWO COMMERCE SQ, 2001 MARKET ST, PHILADELPHIA, PA 19103 USA

SN 0271-6798

EI 1539-2570

J9 J PEDIATR ORTHOPED

JI J. Pediatr. Orthop.

PD JUL

PY 2023

VL 43

IS 6

BP 368

EP 372

DI 10.1097/BPO.0000000000002391

PG 5

WC Orthopedics; Pediatrics

WE Science Citation Index Expanded (SCI-EXPANDED)

SC Orthopedics; Pediatrics

GA I2SP1

UT WOS:001001335600005

PM 36922003

DA 2023-08-10

ER

PT J

AU Schreiber, S

Whibley, D

Somers, EC

AF Schreiber, Sanja

Whibley, Daniel

Somers, Emily C.

TI Schroth Physiotherapeutic Scoliosis-Specific Exercise (PSSE)

Trials-Systematic Review of Methods and Recommendations for Future

Research

SO CHILDREN-BASEL

LA English

DT Review

DE Schroth method; physiotherapeutic scoliosis-specific exercises (PSSE);

exercise therapy; research design; data reporting; exercise trials

ID ADOLESCENT IDIOPATHIC SCOLIOSIS; RANDOMIZED-TRIALS; CURVE PROGRESSION;

CONSORT STATEMENT; NATURAL-HISTORY; ADULT SCOLIOSIS; CLASSIFICATION;

GROWTH; EXPLANATION; MODULATION

AB The Schroth method is a non-operative treatment for scoliosis and kyphosis, used standalone or as an adjunct to bracing. While supporting evidence for its effectiveness is emerging, methodologic standardization and rigor are equivocal. Thus, we aimed to systematically review methods of published Schroth physiotherapeutic scoliosis-specific exercise (PSSE) trials and provide guidance for future research. We searched six databases for randomized controlled trials (RCT) and non-randomized studies of interventions (NRSIs) investigating the effect of Schroth in children and adults with scoliosis or kyphosis. General characteristics, methodological approaches, treatment protocols, and outcomes reporting were analyzed. Risk of bias (RoB) was assessed using an adapted Cochrane RoB2 tool for RCTs and ROBINS-I for NRSI. Eligible studies (n = 7) were conducted in six countries and included patients with Scheuermann's kyphosis (n = 1) and adolescent idiopathic scoliosis (n = 6). Though all seven studies used the term Schroth to describe their interventions, the Schroth method was used in four of seven studies, of which only one used Schroth classification, three used Schroth therapists, and none prospectively registered the study protocol. Overall, methodological rigor was suboptimal, potentially invalidating evidence synthesis. Authors should follow minimum standards for reporting, including prospectively registering detailed protocols; using appropriate exercise labeling, Schroth classification and certified therapists; naming and describing exercises per classification; and providing therapy dosages, prescription methods, and adherence.

C1 [Schreiber, Sanja] Univ Alberta, Fac Rehabil Med, Dept Phys Therapy, Edmonton, AB T6G 2G4, Canada.

[Schreiber, Sanja] Kyphosis & Other Spinal Disorders Ctr, Curvy Spine Specialized Scoliosis, Edmonton, AB T6E 1W7, Canada.

[Whibley, Daniel] Univ Michigan, Dept Phys Med & Rehabil, Ann Arbor, MI 48109 USA.

[Somers, Emily C.] Univ Michigan, Dept Internal Med, Environm Hlth Sci & Obstet & Gynecol, Ann Arbor, MI 48109 USA.

C3 University of Alberta; University of Michigan System; University of

Michigan; University of Michigan System; University of Michigan

RP Schreiber, S (通讯作者)， Univ Alberta, Fac Rehabil Med, Dept Phys Therapy, Edmonton, AB T6G 2G4, Canada.; Schreiber, S (通讯作者)， Kyphosis & Other Spinal Disorders Ctr, Curvy Spine Specialized Scoliosis, Edmonton, AB T6E 1W7, Canada.; Somers, EC (通讯作者)， Univ Michigan, Dept Internal Med, Environm Hlth Sci & Obstet & Gynecol, Ann Arbor, MI 48109 USA.

EM sanja.schreiber@ualberta.ca; emsomers@umich.edu

RI ; Whibley, Daniel/F-9812-2016

OI Schreiber, Sanja/0000-0002-8231-5131; Somers, Emily/0000-0001-5234-3978;

Whibley, Daniel/0000-0002-7131-7158

CR Altman DG, 2001, ANN INTERN MED, V134, P663, DOI 10.7326/0003-4819-134-8-200104170-00012

[Anonymous], 2008, SPINAL DISORDERS FUN

[Anonymous], 2005, THERAPEUTIC EXERCISE

Aronsson DD, 2011, J PEDIATR ORTHOPED, V31, pS99, DOI 10.1097/BPO.0b013e318203b141

Asher MA, 2006, SCOLIOSIS SPINAL DIS, V1, DOI 10.1186/1748-7161-1-2

Bagnall KM, 2009, SCOLIOSIS SPINAL DIS, V4, DOI 10.1186/1748-7161-4-28

Bezalel T, 2019, ASIAN SPINE J, V13, P490, DOI 10.31616/asj.2018.0097

Boutron I, 2008, ANN INTERN MED, V148, P295, DOI 10.7326/0003-4819-148-4-200802190-00008

Burwell R, 2000, ETIOLOGY ADOLESCENT

BYLUND P, 1987, CLIN ORTHOP RELAT R, P222

Cheng JC, 2015, NAT REV DIS PRIMERS, V1, DOI 10.1038/nrdp.2015.30

Cheung KMC, 2008, INT ORTHOP, V32, P729, DOI 10.1007/s00264-007-0393-y

Close C., 2021, DEV PERSON CENTRED C

Damborg F, 2014, DAN MED J, V61

Danielsson AJ, 2001, SPINE, V26, P1449, DOI 10.1097/00007632-200107010-00015

Danielsson AJ, 2003, SPINE, V28, P2078

De Kleuver M, 2017, ACTA ORTHOP, V88, P612, DOI 10.1080/17453674.2017.1371371

Diebo BG, 2019, LANCET, V394, P160, DOI 10.1016/S0140-6736(19)31125-0

Furlan AD, 2015, SPINE, V40, P1660, DOI 10.1097/BRS.0000000000001061

Fusco C., 2011, Physiotherapy Theory and Practice, V27, P80, DOI 10.3109/09593985.2010.533342

Garrido E, 2021, SPINE DEFORM, V9, P1633, DOI 10.1007/s43390-021-00354-y

Hallager DW, 2016, SPINE, V41, pE589, DOI 10.1097/BRS.0000000000001355

Hawes MC, 2006, SCOLIOSIS SPINAL DIS, V1, DOI 10.1186/1748-7161-1-3

Hennes A., 2023, INT SCHROTH 3D SCO 1, P63

Hennes A., 2023, INT SCHROTH 3D SCO 2, P104

Khalafallah A, 2010, MEDITERR J HEMATOL I, V2, DOI [10.4084/MJHID.2010.005, 10.1136/bmj.l4898]

Kim G, 2016, J PHYS THER SCI, V28, P1012, DOI 10.1589/jpts.28.1012

Kocaman H, 2021, PLOS ONE, V16, DOI 10.1371/journal.pone.0249492

Konieczny MR, 2013, J CHILD ORTHOP, V7, P3, DOI 10.1007/s11832-012-0457-4

Kotwicki T, 2009, SCOLIOSIS SPINAL DIS, V4, DOI 10.1186/1748-7161-4-26

Kruse LM, 2012, J BONE JOINT SURG AM, V94A, P1485, DOI 10.2106/JBJS.K.01450

Kuklo TR, 2005, J SPINAL DISORD TECH, V18, P139, DOI 10.1097/01.bsd.0000159033.89623.bc

Kuru T, 2016, CLIN REHABIL, V30, P181, DOI 10.1177/0269215515575745

Lehnert-Schroth C., 2007, 3 DIMENSIONAL TREATM, V7th ed.

Lenke LG, 2001, J BONE JOINT SURG AM, V83A, P1169, DOI 10.2106/00004623-200108000-00006

Martinez-Llorens J, 2010, EUR RESPIR J, V36, P393, DOI 10.1183/09031936.00025509

McGuinness L.A., 2021, DOING METAANALYSIS R

Miller NH, 1999, ORTHOP CLIN N AM, V30, P343, DOI 10.1016/S0030-5898(05)70091-2

Mohamed RA, 2021, EUR REV MED PHARMACO, V25, P7717, DOI 10.26355/eurrev_202112_27618

Moher David, 2012, Int J Surg, V10, P28, DOI 10.1016/j.ijsu.2011.10.001

MURRAY PM, 1993, J BONE JOINT SURG AM, V75A, P236, DOI 10.2106/00004623-199302000-00011

Negrini S, 2015, SCOLIOSIS SPINAL DIS, V10, DOI 10.1186/s13013-014-0025-4

Negrini S, 2009, SCOLIOSIS SPINAL DIS, V4, DOI 10.1186/1748-7161-4-19

Parent EC, 2023, CHILDREN-BASEL, V10, DOI 10.3390/children10020239

Poncet P, 2001, SPINE, V26, P2235, DOI 10.1097/00007632-200110150-00015

Richards BS, 2005, SPINE, V30, P2068, DOI 10.1097/01.brs.0000178819.90239.d0

Rowe DE, 1997, J BONE JOINT SURG AM, V79A, P664, DOI 10.2106/00004623-199705000-00005

Sato T, 2011, EUR SPINE J, V20, P274, DOI 10.1007/s00586-010-1657-6

Schreiber S, 2016, PLOS ONE, V11, DOI 10.1371/journal.pone.0168746

Schreiber S, 2015, SCOLIOSIS SPINAL DIS, V10, DOI 10.1186/s13013-015-0048-5

Schwab F, 2005, SPINE, V30, P1082, DOI 10.1097/01.brs.0000160842.43482.cd

Schwab F, 2003, SPINE, V28, P602, DOI 10.1097/00007632-200303150-00016

Schwab F, 2006, SPINE, V31, P2109, DOI 10.1097/01.brs.0000231725.38943.ab

Schwab F, 2012, SPINE, V37, P1077, DOI 10.1097/BRS.0b013e31823e15e2

Scoliosis Research Society, 2016, AD ID SCOL

Smith JS, 2008, NEUROSURGERY, V63, pA16, DOI 10.1227/01.NEU.0000320447.61835.EA

Smith JS, 2019, J NEUROSURG-SPINE, V30, P551, DOI 10.3171/2019.1.SPINE181494

Sterne JAC, 2016, BMJ-BRIT MED J, V355, DOI 10.1136/bmj.i4919

Stokes IAF, 2004, SPINE, V29, P2103, DOI 10.1097/01.brs.0000141182.42544.1f

Stokes Ian, 2002, Stud Health Technol Inform, V91, P314

Stokes IAF, 2006, SCOLIOSIS SPINAL DIS, V1, DOI 10.1186/1748-7161-1-16

Stokes IAF, 2007, EUR SPINE J, V16, P1621, DOI 10.1007/s00586-007-0442-7

Stokes IAF, 2009, STUD HEALTH TECHNOL, V135, P75

Stokes IAF, 2009, SPINE, V34, P584, DOI 10.1097/BRS.0b013e318190b914

Tones M, 2006, SPINE, V31, P3027, DOI 10.1097/01.brs.0000249555.87601.fc

Ueno M, 2011, J ORTHOP SCI, V16, P1, DOI 10.1007/s00776-010-0009-z

Wang WJ, 2011, J PEDIATR ORTHOPED, V31, pS14, DOI 10.1097/BPO.0b013e3181f73c12

Weinstein SL, 2008, LANCET, V371, P1527, DOI 10.1016/S0140-6736(08)60658-3

Yelin E, 2016, SEMIN ARTHRITIS RHEU, V46, P259, DOI 10.1016/j.semarthrit.2016.07.013

Zapata KA, 2019, PEDIATR PHYS THER, V31, P280, DOI 10.1097/PEP.0000000000000621

NR 70

TC 0

Z9 0

U1 1

U2 1

PU MDPI

PI BASEL

PA ST ALBAN-ANLAGE 66, CH-4052 BASEL, SWITZERLAND

EI 2227-9067

J9 CHILDREN-BASEL

JI Children-Basel

PD JUN

PY 2023

VL 10

IS 6

AR 954

DI 10.3390/children10060954

PG 14

WC Pediatrics

WE Science Citation Index Expanded (SCI-EXPANDED)

SC Pediatrics

GA K1LK6

UT WOS:001014127100001

PM 37371186

OA gold, Green Published

DA 2023-08-10

ER

PT J

AU Diarbakerli, E

Abbott, A

Gerdhem, P

AF Diarbakerli, Elias

Abbott, Allan

Gerdhem, Paul

TI PREventing Mild Idiopathic SCOliosis PROgression (PREMISCOPRO): A

protocol for a randomized controlled trial comparing scoliosis-specific

exercises with observation in mild idiopathic scoliosis

SO PLOS ONE

LA English

DT Article

ID QUALITY-OF-LIFE; ADOLESCENT; BRACE

AB BackgroundIdiopathic scoliosis is the most common spinal deformity in children. Treatment strategies aim to halt progression of the curve. Mild scoliosis is in many cases observed or, in some cases, treated with scoliosis-specific exercises. More severe curves are treated mainly with a brace. The aim of this study is to investigate the effectiveness of scoliosis-specific exercises compared to observation in adolescents with mild idiopathic scoliosis. MethodsSubjects. Previously untreated and skeletally immature children aged 9-15 years of age with idiopathic scoliosis (curve magnitude Cobb 15-24 degrees) will be included. A total of 90 subjects will be included to receive one of two possible interventions.Interventions. Both groups will receive a physical activity prescription according to the World Health Organization recommendations. The intervention group will receive an additional active self-correction treatment strategy for curve correction and will have outpatient sessions once every two weeks for the first three months. They will be prescribed to do the exercises at least three times per week. The intervention will be performed until skeletal maturity or progression of the curve.Outcome. The subjects will participate in the study until curve progression or until skeletal maturity (defined as less than 1 cm growth for six months). The primary outcome variable is failure of treatment, defined as progression of the Cobb angle more than 6 degrees on two consecutive x-

rays compared to the baseline x-ray. Secondary outcome measures include patient-reported outcomes, clinical characteristics (i.e. angle of trunk rotation and trunk asymmetry) and number requiring brace treatment. Clinical follow-ups will be performed every six months and radiographs will be taken annually. DiscussionThis study will compare effectiveness of an active self-corrective exercise strategy in mild idiopathic scoliosis with observation in terms of halting curve progression.

C1 [Diarbakerli, Elias; Gerdhem, Paul] Karolinska Inst, Dept Clin Sci Intervent & Technol, Stockholm, Sweden.

[Diarbakerli, Elias; Gerdhem, Paul] Karolinska Univ Hosp, Dept Reconstruct Orthopaed, Stockholm, Sweden.

[Abbott, Allan] Linkoping Univ, Dept Hlth Med & Caring Sci, Div Prevent Rehabil & Community Med, Unit Physiotherapy, Linkoping, Sweden.

[Abbott, Allan] Linkoping Univ Hosp, Dept Orthopaed, Linkoping, Sweden.

[Gerdhem, Paul] Uppsala Univ, Uppsala Univ Hosp, Dept Orthopaed, Dept Surg Sci, Uppsala, Sweden.

C3 Karolinska Institutet; Karolinska Institutet; Karolinska University

Hospital; Linkoping University; Linkoping University; Uppsala

University; Uppsala University Hospital

RP Diarbakerli, E (通讯作者)， Karolinska Inst, Dept Clin Sci Intervent & Technol, Stockholm, Sweden.; Diarbakerli, E (通讯作者)， Karolinska Univ Hosp, Dept Reconstruct Orthopaed, Stockholm, Sweden.

EM elias.diarbakerli@regionstockholm.se

CR Abbott A, 2013, BMC MUSCULOSKEL DIS, V14, DOI 10.1186/1471-2474-14-261

Bian ZX, 2011, ANN INTERN MED, V154, P290, DOI [10.1136/bmj.c332, 10.7326/0003-4819-154-4-201102150-00016]

BUNNELL WP, 1984, J BONE JOINT SURG AM, V66A, P1381, DOI 10.2106/00004623-198466090-00010

Cobb JR., 1948, INSTR COURSE LECT, V5, P261

Danielsson AJ, 2001, EUR SPINE J, V10, P278, DOI 10.1007/s005860100309

Diarbakerli E, 2018, J BONE JOINT SURG AM, V100, P811, DOI 10.2106/JBJS.17.00822

Dolan LA, 2007, SPINE, V32, pS91, DOI 10.1097/BRS.0b013e318134ead9

Enders CK, 2011, REHABIL PSYCHOL, V56, P267, DOI 10.1037/a0025579

Fan YL, 2020, BMC MUSCULOSKEL DIS, V21, DOI 10.1186/s12891-020-03517-6

Hollis S, 1999, BRIT MED J, V319, P670, DOI 10.1136/bmj.319.7211.670

Kuru T, 2016, CLIN REHABIL, V30, P181, DOI 10.1177/0269215515575745

Michie S, 2011, IMPLEMENT SCI, V6, DOI 10.1186/1748-5908-6-42

Monticone M, 2014, EUR SPINE J, V23, P1204, DOI 10.1007/s00586-014-3241-y

Murray E.J., 2020, RES METHODS MED HLTH, V2, P39, DOI DOI 10.1177/2632084320961043

Negrini S, 2008, J REHABIL MED, V40, P451, DOI 10.2340/16501977-0195

Negrini S, 2018, SCOLIOSIS SPINAL DIS, V13, DOI 10.1186/s13013-017-0145-8

Negrini S, 2015, COCHRANE DB SYST REV, DOI 10.1002/14651858.CD006850.pub3

Sanders JO, 2008, J BONE JOINT SURG AM, V90A, P540, DOI 10.2106/JBJS.G.00004

Wang H, 2021, QUAL LIFE RES, V30, P703, DOI 10.1007/s11136-020-02671-7

Weinstein SL, 2013, NEW ENGL J MED, V369, P1512, DOI 10.1056/NEJMoa1307337

Williams TC, 2018, PEDIATR RES, V84, P487, DOI 10.1038/s41390-018-0071-3

WILLNER S, 1982, ACTA ORTHOP SCAND, V53, P233, DOI 10.3109/17453678208992208

NR 22

TC 0

Z9 0

U1 1

U2 1

PU PUBLIC LIBRARY SCIENCE

PI SAN FRANCISCO

PA 1160 BATTERY STREET, STE 100, SAN FRANCISCO, CA 94111 USA

SN 1932-6203

J9 PLOS ONE

JI PLoS One

PD MAY 8

PY 2023

VL 18

IS 5

DI 10.1371/journal.pone.0285246

PG 10

WC Multidisciplinary Sciences

WE Science Citation Index Expanded (SCI-EXPANDED)

SC Science & Technology - Other Topics

GA G4YI5

UT WOS:000989224700005

PM 37155607

OA gold, Green Published

DA 2023-08-10

ER

PT J

AU Basbug, G

Gurses, HN

Zeren, M

Elmadag, NM

AF Basbug, Gozde

Gurses, Hulya Nilgun

Zeren, Melih

Elmadag, Nuh Mehmet

TI Effects of inspiratory muscle training on respiratory muscle strength,

respiratory function and functional capacity in adolescents with

idiopathic scoliosis A randomized, controlled trial

SO WIENER KLINISCHE WOCHENSCHRIFT

LA English

DT Article

DE Spine deformity; Children; Respiratory muscle training; Pulmonary

function; Exercise capacity

ID 6-MINUTE WALK TEST; PULMONARY-FUNCTION; STATEMENT; MILD; LIMITATION;

CHILDREN; PROGRAM; IMPACT

AB BackgroundAdolescent Idiopathic Scoliosis (AIS) may impair respiratory dynamics and affect the performance of inspiratory and expiratory muscles. The benefit of inspiratory muscle training (IMT) is not well investigated in AIS. We aimed to investigate the effects of IMT on respiratory muscle strength, respiratory function and functional capacity in adolescents with mild to moderate AIS.MethodsThirty-six adolescents were randomized into control or IMT groups. Forced vital capacity (FVC), forced expiratory volume in 1 second (FEV1) and peak expiratory flow (PEF) were measured by spirometry; respiratory muscle strength by maximum inspiratory pressure (MIP) and maximum expiratory pressure (MEP); and functional capacity by 6-min walk test (6MWT) before and after the 8-week-long home-based exercise program. Both groups received conventional exercise program including diaphragmatic breathing exercises, resistive local expansion exercise on the collapsed areas in concave sides of scoliosis, spinal stabilization, strengthening of interscapular muscles and stretching exercises. IMT group also trained with Threshold IMT device for 15 minutes, twice a day for 8 weeks at the intensity of 30% of initial MIP value in addition to conventional exercise program.ResultsFEV1, PEF, MIP, MEP and 6MWT distance significantly improved in both groups. IMT group also showed significant improvement in FVC. The increases in FVC, MIP, MEP and 6MWT distance of IMT group were significantly higher compared to control group.ConclusionIMT is found to be beneficial for patients with AIS for achieving further improvements in respiratory function, respiratory muscle strength and functional capacity compared to conventional exercise program alone.

C1 [Basbug, Gozde; Gurses, Hulya Nilgun] Bezmialem Vakif Univ, Inst Hlth Sci, Dept Cardiopulm Physiotherapy & Rehabil, Istanbul, Turkiye.

[Basbug, Gozde] Istanbul Kent Univ, Fac Hlth Sci, Div Physiotherapy & Rehabil, Istanbul, Turkiye.

[Gurses, Hulya Nilgun] Bezmialem Vakif Univ, Fac Hlth Sci, Div Physiotherapy & Rehabil, Silahtaraga Cd 186 Eyup, Istanbul, Turkiye.

[Zeren, Melih] Izmir Bakircay Univ, Fac Hlth Sci, Div Physiotherapy & Rehabil, Izmir, Turkiye.

[Elmadag, Nuh Mehmet] Bezmialem Vakif Univ, Fac Med, Dept Orthoped & Traumatol, Istanbul, Turkiye.

C3 Bezmialem Vakif University; Istanbul Kent University; Bezmialem Vakif

University; Izmir University of Bakircay; Bezmialem Vakif University

RP Gurses, HN (通讯作者)，Bezmialem Vakif Univ, Inst Hlth Sci, Dept Cardiopulm Physiotherapy & Rehabil, Istanbul, Turkiye.

EM gursesnil@yahoo.com

RI Gurses, H. Nilgun/AAD-3070-2020; zeren, Melih/HNR-7817-2023

OI Gurses, H. Nilgun/0000-0002-5846-6781; zeren, Melih/0000-0002-9749-315X

FU Scientific Project Unit of Bezmialem Vakif University [12.2016/29]

FX This study is supported by the Scientific Project Unit of Bezmialem

Vakif University (Project Number: 12.2016/29). This study is

prospectively registered to ClinicalTrial.gov website (identification

number: NCT03391895).

CR Abdelaal AAM, 2018, J INT MED RES, V46, P381, DOI 10.1177/0300060517715375

Aldrich TK, 2002, AM J RESP CRIT CARE, V166, P548, DOI 10.1164/rccm.166.4.518

American Thoracic Society; European Respiratory Society, 2002, AM J RESP CRIT CARE, V165, P277, DOI [DOI 10.1164/AJRCCM.165.2.ATS01, 10.1164/ajrccm.165.2.ats01]

Aslan GK, 2014, CLIN REHABIL, V28, P573, DOI 10.1177/0269215513512215

Barrios C, 2005, SPINE, V30, P1610, DOI 10.1097/01.brs.0000169447.55556.01

Bartels B, 2013, PHYS THER, V93, P529, DOI 10.2522/ptj.20120210

Crapo RO, 2002, AM J RESP CRIT CARE, V166, P111, DOI 10.1164/rccm.166/1/111

Dimitriadis Z, 2011, RESP CARE, V56, P776, DOI 10.4187/respcare.00783

Alves VLD, 2006, CHEST, V130, P500, DOI 10.1378/chest.130.2.500

Alves VLD, 2016, ACTA ORTOP BRAS, V24, P296, DOI 10.1590/1413-785220162406120752

Alves VLD, 2009, SPINE, V34, pE926, DOI 10.1097/BRS.0b013e3181afd1b2

Durmala J, 2009, STUD HEALTH TECHNOL, V135, P237

El-Hawary R, 2014, PEDIATR CLIN N AM, V61, P1223, DOI 10.1016/j.pcl.2014.08.007

Geiger R, 2007, J PEDIATR-US, V150, P395, DOI 10.1016/j.jpeds.2006.12.052

Gosselink R, 2011, EUR RESPIR J, V37, P416, DOI 10.1183/09031936.00031810

Grivas TB, 2008, STUD HEALTH TECHNOL, V140, P33, DOI 10.3233/978-1-58603-888-5-33

Grivas TB, 2006, SCOLIOSIS SPINAL DIS, V1, DOI 10.1186/1748-7161-1-17

Guidetti L, 2018, PLOS ONE, V13, DOI 10.1371/journal.pone.0209925

HORNSTEIN S, 1987, SPINE, V12, P859, DOI 10.1097/00007632-198711000-00006

Johnston CE, 2011, SPINE, V36, P1096, DOI 10.1097/BRS.0b013e3181f8c931

Kim Min-Jae, 2017, Physical therapy rehabilitation science, V6, P113, DOI 10.14474/ptrs.2017.6.3.113

Korbel Krzysztof, 2014, Pol Orthop Traumatol, V79, P118

Kraemer WJ, 2002, MED SCI SPORT EXER, V34, P364, DOI 10.1097/00005768-200202000-00027

Kumar A, 2017, J CLIN DIAGN RES, V11, pYC1, DOI 10.7860/JCDR/2017/27497.10335

Lanza FC, 2015, PLOS ONE, V10, DOI 10.1371/journal.pone.0146089

Lonstein JE, 2006, CLIN ORTHOP RELAT R, P248, DOI 10.1097/01.blo.0000198725.54891.73

Martinez-Llorens J, 2010, EUR RESPIR J, V36, P393, DOI 10.1183/09031936.00025509

Plentz RDM, 2012, ARQ BRAS CARDIOL, V99, P762, DOI 10.1590/S0066-782X2012001100011

Miller MR, 2005, EUR RESPIR J, V26, P319, DOI 10.1183/09031936.05.00034805

Monticone M, 2014, EUR SPINE J, V23, P1204, DOI 10.1007/s00586-014-3241-y

Negrini Stefano, 2003, Pediatr Rehabil, V6, P227, DOI: 10.1080/13638490310001636781

Negrini S, 2012, SCOLIOSIS SPINAL DIS, V7, DOI 10.1186/1748-7161-7-3

Parent Stefan, 2005, Instr Course Lect, V54, P529

QUANJER PH, 1993, EUR RESPIR J, V6, P5, DOI 10.1183/09041950.005s1693

Smart NA, 2013, INT J CARDIOL, V167, P1502, DOI 10.1016/j.ijcard.2012.04.029

Souza H, 2014, J GERONTOL A-BIOL, V69, P1545, DOI 10.1093/gerona/glu182

Sperandio EF, 2014, SPINE J, V14, P2366, DOI 10.1016/j.spinee.2014.01.041

Spruit MA, 2013, AM J RESP CRIT CARE, V188, pE13, DOI 10.1164/rccm.201309-1634ST

Szopa A, 2017, MEDICINE, V96, DOI 10.1097/MD.0000000000007032

Tsiligiannis T, 2012, SCOLIOSIS SPINAL DIS, V7, DOI 10.1186/1748-7161-7-7

Weinstein SL, 2003, JAMA-J AM MED ASSOC, V289, P559, DOI 10.1001/jama.289.5.559

Weinstein SL, 2008, LANCET, V371, P1527, DOI 10.1016/S0140-6736(08)60658-3

Weiss HR, 2008, EUR J PHYS REHAB MED, V44, P177

WINTER RB, 1986, NEW ENGL J MED, V314, P1379, DOI 10.1056/NEJM198605223142108

Zeren M, 2019, RESP MED, V148, P24, DOI 10.1016/j.rmed.2019.01.013

Zeren M, 2016, CLIN REHABIL, V30, P1165, DOI 10.1177/0269215515628038

NR 46

TC 0

Z9 0

U1 4

U2 4

PU SPRINGER WIEN

PI Vienna

PA Prinz-Eugen-Strasse 8-10, A-1040 Vienna, AUSTRIA

SN 0043-5325

EI 1613-7671

J9 WIEN KLIN WOCHENSCHR

JI Wien. Klin. Wochen.

PD JUN

PY 2023

VL 135

IS 11-12

BP 282

EP 290

DI 10.1007/s00508-023-02197-1

EA APR 2023

PG 9

WC Medicine, General & Internal

WE Science Citation Index Expanded (SCI-EXPANDED)

SC General & Internal Medicine

GA K8OD4

UT WOS:000971477500001

PM 37071202

DA 2023-08-10

ER

PT J

AU Ceballos-Laita, L

Carrasco-Uribarren, A

Cabanillas-Barea, S

Perez-Guillen, S

Pardos-Aguilella, P

Jimenez Del Barrio, S

AF Ceballos-Laita, Luis

Carrasco-Uribarren, Andoni

Cabanillas-Barea, Sara

Perez-Guillen, Silvia

Pardos-aguilella, Pilar

Jimenez Del Barrio, Sandra

TI The effectiveness of Schroth method in Cobb angle, quality of life and

trunk rotation angle in adolescent idiopathic scoliosis: a systematic

review and meta-analysis

SO EUROPEAN JOURNAL OF PHYSICAL AND REHABILITATION MEDICINE

LA English

DT Review

DE Scoliosis; Exercise; Systematic review; Meta-analysis

ID EXERCISES; IMPACT

AB INTRODUCTION: The Schroth method is one of the most common physiotherapeutic scoliosis-specific exercises intervention applied in adolescent idiopathic scoliosis (AIS). This method consists of three-dimensional correction of the specific curve pattern of the patient using a combination of sensorimotor, postural, and corrective breathing exercises. The aim of this systematic review and meta-analysis was to analyse the effects of the Schroth method in isolation on Cobb angle, quality of life, and trunk rotation angle compared to no intervention or other conservative treatments in patients with AIS.

EVIDENCE ACQUISITION: PubMed, Physiotherapy Evidence Database, Scopus, Cochrane Library, and Web of Science databases were searched. Studies were included if they were randomized controlled trials that compared the effects of the Schroth method in isolation to conservative interventions or no intervention. The quality of the studies was assessed with the PEDro Scale, and the risk of bias with the Cochrane Collaboration tool. Two independent assessors extracted data through a standardized form. Meta-analyses were conducted using fixed or random effects models according to the heterogeneity assessed with I-2 coefficient. Data on outcomes of interest were extracted by a researcher using RevMan 5.4 software.

EVIDENCE SYNTHESIS: A total of 317 studies were screened. Six were included in the meta-analysis involving 144 patients with AIS. The methodological quality of the included studies ranged from high to low. Schroth method in isolation showed significant improvements in Cobb angle (mean difference [MD] =-3.18 degrees; 95% CI: -4.30,-2.07; I-2: 0%), quality of life (MD=0.28; 95% CI: 0.18, 0.38; I-2: 0%) and trunk rotation angle (MD=-2.12 degrees; 95% CI:-3.44, -0.80; I-2: 71%) in the short-term.

CONCLUSIONS: The Schroth method in isolation is effective for reducing the Cobb angle and the trunk rotation angle and for improving the QoL in the short-term compared to no intervention or other conservative therapies in AIS, but the improvement in Cobb angle did not exceed the minimum clinically important difference.

C1 [Ceballos-Laita, Luis; Jimenez Del Barrio, Sandra] Univ Valladolid, Dept Surg Ophthalmol Otorhinolaryngol & Physiother, Clin Res Hlth Sci Grp, Soria, Spain.

[Carrasco-Uribarren, Andoni; Cabanillas-Barea, Sara; Perez-Guillen, Silvia] Univ Int Catalunya UIC, Dept Physiotherapy, Barcelona, Spain.

[Pardos-aguilella, Pilar] Univ Zaragoza, Dept Physiatry & Nursery, Zaragoza, Spain.

C3 Universidad de Valladolid; Universitat Internacional de Catalunya (UIC);

University of Zaragoza

RP Cabanillas-Barea, S (通讯作者)，Univ Int Catalunya UIC, Dept Physiotherapy, Barcelona, Spain.

EM scabanillas@uic.es

OI Pardos Aguilella, Pilar/0000-0001-7732-3914

CR [Anonymous], 2021, J PHYSIOTHER, V67, P66, DOI 10.1016/j.jphys.2020.07.003

Bettany-Saltikov J, 2014, EUR J PHYS REHAB MED, V50, P111

Borysov M, 2016, CURR PEDIATR REV, V12, P12, DOI 10.2174/1573396312666151117120313

Burger M, 2019, SOUTH AFR J PHYSIOTH, V75, DOI 10.4102/sajp.v75i1.904

Carreon LY, 2010, SPINE, V35, P2079, DOI 10.1097/BRS.0b013e3181c61fd7

Laita LC, 2018, ARCH ARGENT PEDIATR, V116, pE582, DOI [10.5546/aap.2018.e582, 10.5546/aap.2018.eng.e582]

Cheung JPY, 2020, CLIN ORTHOP RELAT R, V478, P334, DOI 10.1097/CORR.0000000000000989

Alves VLD, 2006, CHEST, V130, P500, DOI 10.1378/chest.130.2.500

Freidel K, 2002, SPINE, V27, pE87, DOI 10.1097/00007632-200202150-00013

Furlan AD, 2015, SPINE, V40, P1660, DOI 10.1097/BRS.0000000000001061

Gao A, 2021, CHINESE MED J-PEKING, V134, P2589, DOI 10.1097/CM9.0000000000001799

Higgins JPT, 2011, BMJ-BRIT MED J, V343, DOI 10.1136/bmj.d5928

Horne JP, 2014, AM FAM PHYSICIAN, V89, P193

Keenan BE, 2014, SCOLIOSIS SPINAL DIS, V9, DOI 10.1186/1748-7161-9-16

Kim G, 2016, J PHYS THER SCI, V28, P1012, DOI 10.1589/jpts.28.1012

Kocaman H, 2021, PLOS ONE, V16, DOI 10.1371/journal.pone.0249492

Kuru T, 2016, CLIN REHABIL, V30, P181, DOI 10.1177/0269215515575745

Li KP, 2021, EUR J MED RES, V26, DOI 10.1186/s40001-021-00526-6

Miller NH, 1999, ORTHOP CLIN N AM, V30, P343, DOI 10.1016/S0030-5898(05)70091-2

Mohamed RA, 2021, EUR REV MED PHARMACO, V25, P7717, DOI 10.26355/eurrev_202112_27618

Negrini S, 2016, SCOLIOSIS SPINAL DIS, V13

Negrini S, 2008, DISABIL REHABIL, V30, P731, DOI 10.1080/09638280801889485

Negrini S, 2006, SCOLIOSIS SPINAL DIS, V1, DOI 10.1186/1748-7161-1-14

Negrini S, 2006, SCOLIOSIS SPINAL DIS, V1, DOI 10.1186/1748-7161-1-4

Negrini S, 2012, SCOLIOSIS SPINAL DIS, V7, DOI 10.1186/1748-7161-7-3

Otman Saadet, 2005, Neurosciences (Riyadh), V10, P277

Page MJ, 2021, INT J SURG, V88, DOI [10.1016/j.ijsu.2021.105906, 10.1186/s13643-021-01626-4, 10.1016/j.rec.2021.07.010, 10.1016/j.recesp.2021.06.016, 10.1016/j.jclinepi.2021.03.001, 10.1136/bmj.n71]

Parent EC, 2010, SPINE, V35, P315, DOI 10.1097/BRS.0b013e3181cabe75

Park JH, 2018, EUR J PHYS REHAB MED, V54, P440, DOI 10.23736/S1973-9087.17.04461-6

Reichel Dagmar, 2003, Pediatr Rehabil, V6, P221

Rigo M, 2003, Pediatr Rehabil, V6, P209

Sapkas G, 2003, CLIN ORTHOP RELAT R, P32, DOI 10.1097/01.blo.0000068360.47147.30

Schlosser TPC, 2014, PLOS ONE, V9, DOI 10.1371/journal.pone.0097461

Schreiber S, 2019, BMC MUSCULOSKEL DIS, V20, DOI 10.1186/s12891-019-2695-9

Schreiber S, 2016, PLOS ONE, V11, DOI 10.1371/journal.pone.0168746

Schreiber S, 2015, SCOLIOSIS SPINAL DIS, V10, DOI 10.1186/s13013-015-0048-5

Schreiber Sanja, 2014, J Physiother, V60, P234, DOI 10.1016/j.jphys.2014.08.005

Shah J, 2019, ANN RHEUM DIS, V78, P2151, DOI 10.1136/annrheumdis-2019-eular.7716

Tan KJ, 2009, SPINE, V34, P697, DOI 10.1097/BRS.0b013e31819c9431

Verhagen AP, 1998, J CLIN EPIDEMIOL, V51, P1235, DOI 10.1016/S0895-4356(98)00131-0

Weinstein SL, 2008, LANCET, V371, P1527, DOI 10.1016/S0140-6736(08)60658-3

Weiss Hans-Rudolf, 2003, Pediatr Rehabil, V6, P23, DOI 10.1080/1363849031000095288

Weiss HR, SCHROTH THERAPY ADV

Young JL, 2018, BRAZ J PHYS THER, V22, P20, DOI 10.1016/j.bjpt.2017.10.001

NR 44

TC 0

Z9 0

U1 11

U2 11

PU EDIZIONI MINERVA MEDICA

PI TURIN

PA CORSO BRAMANTE 83-85 INT JOURNALS DEPT., 10126 TURIN, ITALY

SN 1973-9087

EI 1973-9095

J9 EUR J PHYS REHAB MED

JI Eur. J. Phys. Rehabil. Med.

PD APR

PY 2023

VL 59

IS 2

BP 228

EP 236

DI 10.23736/S1973-9087.23.07654-2

PG 9

WC Rehabilitation

WE Science Citation Index Expanded (SCI-EXPANDED)

SC Rehabilitation

GA F6BP6

UT WOS:000983180600011

PM 36692412

OA Green Published, hybrid

DA 2023-08-10

ER

PT J

AU Zhang, TY

Huang, ZF

Sui, WY

Wei, WQ

Shao, XX

Deng, YL

Yang, JL

Yang, JF

AF Zhang, Tianyuan

Huang, Zifang

Sui, Wenyuan

Wei, Wenqing

Shao, Xiexiang

Deng, Yaolong

Yang, Junlin

Yang, Jingfan

TI Intensive bracing management combined with physiotherapeutic

scoliosis-specific exercises for adolescent idiopathic scoliosis

patients with a major curve ranging from 40-60? who refused surgery: a

prospective cohort study

SO EUROPEAN JOURNAL OF PHYSICAL AND REHABILITATION MEDICINE

LA English

DT Article

DE Adolescent; Scoliosis; Physical therapy modalities

ID COBB ANGLE; PROGRESSION

AB BACKGROUND: Current guidelines for brace management of adolescent idiopathic scoliosis (AIS) are mostly recommended for curves be-tween 25 degrees to 40 degrees. For AIS patients with curves >40 degrees, surgery is often considered since bracing may be less effective; however, there are still some patients and families who refuse operation. Therefore, further research is necessary to determine optimal bracing management in this group. To date, few protocols for such have been reported in literature.AIM: The aim of this study was to introduce and evaluate the effectiveness of the treatment protocol comprising of intensive bracing manage-ment and physiotherapeutic scoliosis-specific exercises (PSSE) in AIS patients with a major curve of 40-60 degrees who refuse surgery. DESIGN: This is a prospective cohort study. SETTING: The study was carried out in an outpatient clinic. POPULATION: 10-18-year-old AIS patients having 40-60 degrees curves and a Risser grade of 0-3, but firmly refusing surgery were eligible. Patients who had a proximal thoracic curve or had undergone any other form of treatment previously were excluded from the study.METHODS: A total of 82 patients were recruited and received the treatment. The primary outcome was defined as "success" when the main curve was below 50 degrees upon reaching skeletal maturity, and "failure" if otherwise. The secondary outcome was defined as improved (>5 degrees reduc-tion), unchanged (<5 degrees change) or progressed (>5 degrees increase) based on the evolution of the main curve. The per protocol (PP) and intent to treat (ITT) analyses were performed to quantify success rates, while the dropouts were considered as failures. Risk factors associated with bracing failure were identified and a receiver operating characteristic (ROC) curve was used to determine the cut-off value.RESULTS: A total of 77 patients completed the treatment, while 5 dropped out. The average main curve was 47.40 +/- 5.93 degrees at baseline and 38.56 +/- 11.85 degrees at last follow-up (P<0.001). Our management was successful in 83% and 78% of patients based on the PP and ITT analyses, respectively. When compared with the curve magnitude at baseline, 65% patients improved, 30% remained unchanged, and 5% progressed when using a 5 degrees threshold. Univariate comparison and logistic regression analysis demonstrated that patients with successful outcomes had a significantly smaller baseline curve, larger Risser Stage, and larger in-brace correction (IBC) rate.CONCLUSIONS: For AIS patients with 40-60 degrees curves who refused

surgery, our intensive bracing management along with PSSE was practical and effective, achieving success in 78% of patients based on an ITT analysis. A larger baseline curve, smaller Risser Stage, and smaller IBC rate were associated with treatment failure.CLINICAL REHABILITATION IMPACT: Our intensive management provides new insights into improving the effectiveness of bracing in patients with AIS who refuse surgery. This is a promising option for patients with 40-60 degrees curves, since their scoliosis may be treated using a non-surgical technique instead of surgery in the future.

C1 [Zhang, Tianyuan; Sui, Wenyuan; Shao, Xiexiang; Deng, Yaolong; Yang, Junlin; Yang, Jingfan] Shanghai Jiao Tong Univ, Xinhua Hosp, Spine Ctr, Sch Med, Shanghai, china.

[Huang, Zifang] Sun Yat Sen Univ, Affiliated Hosp 3, Dept Spine Surg, Guangzhou, china.

[Sui, Wenyuan] Univ Shanghai Sci & Technol, Sch Med Instrument & Food Engn, Shanghai, china.

[Yang, Junlin; Yang, Jingfan] Shanghai Jiao Tong Univ, Xinhua Hosp, Sch Med, Dept Pediat Orthoped, Shanghai, china.

[Yang, Junlin] Shanghai Jiao Tong Univ, Xinhua Hosp, Spine Ctr, Sch Med, 1665 Kongjiang Rd, Shanghai 200092, china.

C3 Shanghai Jiao Tong University; Sun Yat Sen University; University of

Shanghai for Science & Technology; Shanghai Jiao Tong University;

Shanghai Jiao Tong University

RP Yang, JL (通讯作者)， Shanghai Jiao Tong Univ, Xinhua Hosp, Spine Ctr, Sch Med, 1665 Kongjiang Rd, Shanghai 200092, china.

EM yjunlin@126.com

OI Zhang, Tianyuan/0000-0001-7843-9249

FU National Natural Science Foundation of China [82072519]; Clinical

Science and Technology Innovation Project of Shanghai Shenkang Hospital

Development Center [SHDC22020206]; Shanghai Science and Technology

Innovation Program [22DZ2203300]

FX Funding.-This study was funded by the National Natural Science

Foundation of China (82072519) ; the Clinical Science and Technology

Innovation Project of Shanghai Shenkang Hospital Development Center

(SHDC22020206) ; and the Shanghai Science and Technology Innovation

Program (22DZ2203300) .

CR Altaf F, 2013, BMJ-BRIT MED J, V346, DOI 10.1136/bmj.f2508

ASCANI E, 1986, SPINE, V11, P784, DOI 10.1097/00007632-198610000-00007

Aubin CE, 1999, SPINE, V24, P349, DOI 10.1097/00007632-199902150-00010

Aulisa AG, 2019, EUR J PHYS REHAB MED, V55, P231, DOI 10.23736/S1973-9087.18.04782-2

Clin J, 2010, SPINE, V35, P1706, DOI 10.1097/BRS.0b013e3181cb46f6

Clin J, 2010, EUR SPINE J, V19, P1169, DOI 10.1007/s00586-009-1268-2

El Hawary R, 2019, SPINE J, V19, P1917, DOI 10.1016/j.spinee.2019.07.008

Gallant JN, 2018, WORLD NEUROSURG, V116, P421, DOI 10.1016/j.wneu.2018.05.104

Goodbody CM, 2016, J CHILD ORTHOP, V10, P395, DOI 10.1007/s11832-016-0763-3

Karam JA, 2019, ORTHOP TRAUMATOL-SUR, V105, P727, DOI 10.1016/j.otsr.2019.03.004

Karol LA, 2016, J BONE JOINT SURG AM, V98, P1253, DOI 10.2106/JBJS.15.01313

Liu DL, 2020, SPINE, V45, P1039, DOI 10.1097/BRS.0000000000003451

Lusini M, 2014, SPINE J, V14, P1951, DOI 10.1016/j.spinee.2013.11.040

Mac-Thiong JM, 2004, SPINE, V29, P26, DOI 10.1097/01.BRS.0000103943.25412.E9

Monticone M, 2014, EUR SPINE J, V23, P1204, DOI 10.1007/s00586-014-3241-y

Negrini S, 2014, EUR J PHYS REHAB MED, V50, P87

Negrini S, 2019, ANN PHYS REHABIL MED, V62, P69, DOI 10.1016/j.rehab.2018.07.010

Negrini S, 2018, SCOLIOSIS SPINAL DIS, V13, DOI 10.1186/s13013-017-0145-8

Negrini S, 2011, SPINE J, V11, P369, DOI 10.1016/j.spinee.2010.12.001

Negrini S, 2009, SCOLIOSIS SPINAL DIS, V4, DOI 10.1186/1748-7161-4-2

Razeghinezhad R, 2021, NEUROSPINE, V18, P437, DOI 10.14245/ns.2040654.327

Richards BS, 2005, SPINE, V30, P2068, DOI 10.1097/01.brs.0000178819.90239.d0

Rigo M, 2006, SCOLIOSIS SPINAL DIS, V1, DOI 10.1186/1748-7161-1-11

Schiller JR, 2010, CLIN ORTHOP RELAT R, V468, P670, DOI 10.1007/s11999-009-0884-9

Schreiber Sanja, 2014, J Physiother, V60, P234, DOI 10.1016/j.jphys.2014.08.005

Smith VA, 2021, JAMA-J AM MED ASSOC, V326, P433, DOI 10.1001/jama.2021.2825

Steen H, 2021, EUR J PHYS REHAB MED, V57, P101, DOI 10.23736/S1973-9087.20.06190-0

Verhofste BP, 2020, SPINE DEFORM, V8, P911, DOI 10.1007/s43390-020-00131-3

WEINSTEIN SL, 1983, J BONE JOINT SURG AM, V65, P447, DOI 10.2106/00004623-198365040-00004

WEINSTEIN SL, 1981, J BONE JOINT SURG AM, V63, P702, DOI 10.2106/00004623-198163050-00003

Weinstein SL, 2003, JAMA-J AM MED ASSOC, V289, P559, DOI 10.1001/jama.289.5.559

Weinstein SL, 2008, LANCET, V371, P1527, DOI 10.1016/S0140-6736(08)60658-3

Weinstein SL, 2013, SPINE, V38, P1832, DOI 10.1097/01.brs.0000435048.23726.3e

Weinstein SL, 2013, NEW ENGL J MED, V369, P1512, DOI 10.1056/NEJMoa1307337

Weiss HR, 2010, SCOLIOSIS SPINAL DIS, V5, DOI 10.1186/1748-7161-5-22

Wood G, 2014, SCOLIOSIS SPINAL DIS, V9, DOI 10.1186/1748-7161-9-2

Xu LL, 2017, CLIN SPINE SURG, V30, pE475, DOI 10.1097/BSD.0000000000000343

Zaina F, 2014, EUR J PHYS REHAB MED, V50, P93

Zaina F, 2022, CHILDREN-BASEL, V9, DOI 10.3390/children9111672

Zheng Y, 2018, SPINE, V43, pE494, DOI 10.1097/BRS.0000000000002412

Zhu ZZ, 2017, CLIN SPINE SURG, V30, P85, DOI 10.1097/BSD.0b013e3182a1de29

NR 41

TC 0

Z9 0

U1 1

U2 1

PU EDIZIONI MINERVA MEDICA

PI TURIN

PA CORSO BRAMANTE 83-85 INT JOURNALS DEPT., 10126 TURIN, ITALY

SN 1973-9087

EI 1973-9095

J9 EUR J PHYS REHAB MED

JI Eur. J. Phys. Rehabil. Med.

PD APR

PY 2023

VL 59

IS 2

BP 212

EP 221

DI 10.23736/S1973-9087.23.07605-0

PG 10

WC Rehabilitation

WE Science Citation Index Expanded (SCI-EXPANDED)

SC Rehabilitation

GA F6BP6

UT WOS:000983180600009

PM 36700244

OA Green Published, hybrid

DA 2023-08-10

ER

PT J

AU Muccio, M

Atun-Einy, O

Kafri, M

Kaplan, SL

AF Muccio, Marissa

Atun-Einy, Osnat

Kafri, Michal

Kaplan, Sandra L.

TI Bridging Motor Learning Principles with Physiotherapy Specific Scoliosis

Exercises: a Perspective Article

SO PHYSICAL & OCCUPATIONAL THERAPY IN PEDIATRICS

LA English

DT Article; Early Access

DE Motor learning and control; physiotherapeutic scoliosis specific

exercises; scoliosis; spine; therapeutic exercise

ID ADOLESCENT IDIOPATHIC SCOLIOSIS; PHYSICAL-THERAPY; REHABILITATION;

CHILDREN

AB Aim: This perspective paper illustrates the usefulness of explicitly integrating motor learning terminology with evolving therapeutic approaches. Physiotherapy specific scoliosis exercises (PSSEs) include a growing number of approaches to scoliosis management and serve as an example of this integration.Methods: Three quintessential patient cases (a young hypermobile adolescent, a post-pubescent teen, and an adult with childhood diagnosis of scoliosis) serve to contrast the clinical decision-making process for a PSSE plan of care when organized within a motor learning framework.Conclusions and implications: As intervention approaches evolve, aligning the unique terminologies from different schools of thought with motor learning constructs would provide a common language for clinicians, academics and researchers to facilitate comparison of approaches and organize intervention care plans. Linking a motor learning framework and terminology to PSSE may facilitate comparison of PSSE treatment approaches by clinicians, academics, and researchers, as well as advance the global quality of care for patients with scoliosis.

C1 [Muccio, Marissa] Scoliosis Specialty Ctr, Div PRNY PC, Totowa, NJ USA.

[Atun-Einy, Osnat; Kafri, Michal] Univ Haifa, Fac Social Welf & Hlth Sci, Dept Phys Therapy, Haifa, Israel.

[Kaplan, Sandra L.] Rutgers State Univ, Dept Rehabil & Movement Sci, Newark, NJ USA.

[Muccio, Marissa] Scoliosis Specialty Ctr, 265 Rte 46 Suite 102, Totowa, NJ 07512 USA.

C3 University of Haifa; Rutgers State University Newark; Rutgers State
University New Brunswick

RP Muccio, M (通讯作者)，Scoliosis Specialty Ctr, 265 Rte 46 Suite 102, Totowa, NJ 07512 USA.

EM mmuccio@pediatricrehab.net

CR [Anonymous], 2005, MOTOR CONTROL LEARNI

[Anonymous], 2018, MOTOR CONTROL LEARNI

APTA, 2015, PO6152524 APTA HOD

Armitano CN, 2018, J ATHL TRAINING, V53, P844, DOI 10.4085/1062-6050-320-17

Atun-Einy O, 2019, PHYSIOTHER THEOR PR, V35, P633, DOI 10.1080/09593985.2018.1456585

Barcelona Scoliosis Physical Therapy School and Schroth-Barcelona Institute, 2017, COURS HDB CERT LEV 2

Barker KL, 2009, BMC MUSCULOSKEL DIS, V10, DOI 10.1186/1471-2474-10-123

Berdishevsky H, 2016, SCOLIOSIS SPINAL DIS, V11, DOI 10.1186/s13013-016-0076-9

Bettany-Saltikov J, 2014, EUR J PHYS REHAB MED, V50, P111

Bezalel T, 2019, ASIAN SPINE J, V13, P490, DOI 10.31616/asj.2018.0097

Burger M, 2019, SOUTH AFR J PHYSIOTH, V75, DOI 10.4102/sajp.v75i1.904

Cano-de-la-Cuerda R, 2015, NEUROLOGIA, V30, P32, DOI 10.1016/j.nrl.2011.12.010

Charlton JM, 2021, PHYS THER, V101

Coker C., 2017, MOTOR LEARNING CONTR

D'Andrea CR, 2021, J ORTHOP RES, V39, P919, DOI 10.1002/jor.24992

D'Andrea CR, 2021, J ORTHOP RES, V39, P907, DOI 10.1002/jor.24976

Dufvenberg M, 2018, SCOLIOSIS SPINAL DIS, V13, DOI 10.1186/s13013-018-0163-1

Gao CF, 2019, AM J PHYS MED REHAB, V98, P642, DOI 10.1097/PHM.0000000000001160

Giordamni M.-A., 2021, SCOLIOSIS SPINAL DIS, V11, P88

Goh HT, 2012, RES Q EXERCISE SPORT, V83, P346, DOI 10.1080/02701367.2012.10599866

Gokeler A, 2019, SPORTS MED, V49, P853, DOI 10.1007/s40279-019-01058-0

Hoffmann TC, 2014, BMJ-BRIT MED J, V348, DOI [10.1136/bmj.g1687, 10.1055/s-0041-111066]

Jette AM, 2020, PHYS THER, V100, P883, DOI 10.1093/ptj/pzaa078

Kafri M, 2019, PHYS THER, V99, P1628, DOI 10.1093/ptj/pzz118

Kleynen M, 2020, PHYSIOTHER THEOR PR, V36, P1, DOI 10.1080/09593985.2018.1483987

Kuru T, 2016, CLIN REHABIL, V30, P181, DOI 10.1177/0269215515575745

Kwan KYH, 2017, SCOLIOSIS SPINAL DIS, V12, DOI 10.1186/s13013-017-0139-6

Lafrance S, 2021, PHYS THER, V101, DOI 10.1093/ptj/pzab072

Lanthier J, 2020, NEUROSCI LETT, V722, DOI 10.1016/j.neulet.2020.134836

Le Berre M, 2019, SPINE DEFORM, V7, P71, DOI 10.1016/j.jspd.2018.05.004

Leech K. A., 2022, SCOLIOSIS SPINAL DIS, V102, P1

Levac D, 2009, PEDIATR PHYS THER, V21, P345, DOI 10.1097/PEP.0b013e3181beb09d

Liu DL, 2020, SPINE, V45, P1039, DOI 10.1097/BRS.0000000000003451

Magill R.A., 2018, MOTOR LEARNING CONTR

Maier M, 2019, FRONT SYST NEUROSCI, V13, DOI 10.3389/fnsys.2019.00074

Mohamed RA, 2021, EUR REV MED PHARMACO, V25, P7717, DOI 10.26355/eurrev_202112_27618

Monticone M, 2014, EUR SPINE J, V23, P1204, DOI 10.1007/s00586-014-3241-y

Negrini S, 2015, SCOLIOSIS SPINAL DIS, V10, DOI 10.1186/s13013-014-0025-4

Newell KM, 2001, HUM MOVEMENT SCI, V20, P695, DOI 10.1016/S0167-9457(01)00073-2

NEWELL KM, 1991, ANNU REV PSYCHOL, V42, P213, DOI 10.1146/annurev.ps.42.020191.001241

Ng JY, 2021, MUSCULOSKEL SCI PRAC, V51, DOI 10.1016/j.msksp.2020.102295

Novak I, 2020, CURR NEUROL NEUROSCI, V20, DOI 10.1007/s11910-020-1022-z

Nunes MES, 2014, FRONT PSYCHOL, V5, DOI 10.3389/fpsyg.2014.01454

Pialasse JP, 2017, GAIT POSTURE, V57, P124, DOI 10.1016/j.gaitpost.2017.05.032

Sawers A, 2012, J REHABIL RES DEV, V49, P1431, DOI 10.1682/JRRD.2011.12.0235

Schmidt R.A., 2019, MOTOR LEARNING PERFO, V6th ed

Schmidt RA, 2003, RES Q EXERCISE SPORT, V74, P366, DOI 10.1080/02701367.2003.10609106

Schreiber S, 2016, PLOS ONE, V11, DOI 10.1371/journal.pone.0168746

Schreiber S, 2015, SCOLIOSIS SPINAL DIS, V10, DOI 10.1186/s13013-015-0048-5

Sidaway B, 2012, PHYS THER, V92, P948, DOI 10.2522/ptj.20110378

Sullivan KI, 2008, PHYS THER, V88, P720, DOI 10.2522/ptj.20070196

Ustinova KI, 2015, PHYSIOTHER THEOR PR, V31, P1, DOI 10.3109/09593985.2014.945674

van Dijk L, 2017, J MOTOR BEHAV, V49, P244, DOI 10.1080/00222895.2016.1191418

Vaz DV, 2021, BMC MED EDUC, V21, DOI 10.1186/s12909-021-02486-1

Verburgh L, 2016, J SPORT SCI, V34, P1782, DOI 10.1080/02640414.2015.1137344

Winstein C, 2014, J NEUROL PHYS THER, V38, P190, DOI 10.1097/NPT.0000000000000046

Wulf G, 2001, Q J EXP PSYCHOL-A, V54, P1143, DOI 10.1080/02724980143000118

Yagci G, 2019, PROSTHET ORTHOT INT, V43, P301, DOI 10.1177/0309364618820144

Yagci G, 2018, PHYSIOTHER THEOR PR, V34, P579, DOI 10.1080/09593985.2017.1423429

Zanca JM, 2019, ARCH PHYS MED REHAB, V100, P164, DOI 10.1016/j.apmr.2018.09.110

Zapata KA, 2019, PEDIATR PHYS THER, V31, P280, DOI 10.1097/PEP.0000000000000621

Zwicker JG, 2009, CAN J OCCUP THER, V76, P29, DOI 10.1177/000841740907600108

NR 62

TC 0

Z9 0

U1 2

U2 2

PU TAYLOR & FRANCIS INC

PI PHILADELPHIA

PA 530 WALNUT STREET, STE 850, PHILADELPHIA, PA 19106 USA

SN 0194-2638

EI 1541-3144

J9 PHYS OCCUP THER PEDI

JI Phys. Occup. Ther. Pediatr.

PD 2023 MAR 15

PY 2023

DI 10.1080/01942638.2023.2186198

EA MAR 2023

PG 18

WC Pediatrics; Rehabilitation

WE Science Citation Index Expanded (SCI-EXPANDED); Social Science Citation Index (SSCI)

SC Pediatrics; Rehabilitation

GA A5DA5

UT WOS:000955314800001

PM 36922700

DA 2023-08-10

ER

PT J

AU Bazancir, Z

Talu, B

Korkmaz, MF

AF Bazancir, Zilan

Talu, Burcu

Korkmaz, Mehmet Fatih

TI Postoperative rehabilitation versus early mobilization following

scoliosis surgery: A single-blind randomized clinical trial

SO JOURNAL OF ORTHOPAEDIC SCIENCE

LA English

DT Article

ID ADOLESCENT IDIOPATHIC SCOLIOSIS; POSTERIOR SPINAL-FUSION; EARLY

DISCHARGE; RELIABILITY; VALIDITY; BALANCE

AB Background: To compare the effect of five days of intensive postoperative rehabilitation and early mobilization following scoliosis surgery.Methods: Forty adolescent patients who had undergone scoliosis surgery were randomly allocated into a rehabilitation group (RG, n = 20) and a mobilization group (MG, n = 20). The RG received five days of intensive exercise program and early ambulation, the MG received five days of a standard gait training and early ambulation. The patients were evaluated for severity of pain using a visual analog scale, thorax mobility with the thoracic mobility index, balance with the functional reach test, walking distance with the 2-min walk test, and quality of life with the Scoliosis Research Society-22 questionnaire. The length of hospital stay was recorded.Results: The RG was favored over the MG for improvements from 0 to 1 week for pain. Improvements from 0 to 1 week were significantly better in the RG than the MG group for thorax mobility, balance, and walking distance. Quality of life scores improvements from 0 to 1 week were significantly better in the RG group than in the MG group. The length of hospital stay was significantly shorter in the RG group. Conclusion: Five days of intensive postoperative rehabilitation were superior to early mobilization in reducing the length of hospital stay, and in improving physical and functional outcomes following scoliosis surgery.(c) 2021 The Japanese Orthopaedic Association. Published by Elsevier B.V. All rights reserved.

C1 [Bazancir, Zilan] Hacettepe Univ, Fac Phys Therapy & Rehabil, Ankara, Turkiye.

[Talu, Burcu] Inonu Univ, Fac Hlth Sci, Dept Physiotherapy & Rehabil, Malatya, Turkiye.

[Korkmaz, Mehmet Fatih] Istanbul Medeniyet Univ, Fac Med, Dept Orthopaed & Traumatol, Istanbul, Turkiye.

[Bazancir, Zilan] Hacet tepe Univ, Fac Phys Therapy & Rehabil, TR-06100 Ankara, Turkiye.

C3 Hacettepe University; Inonu University; Istanbul Medeniyet University

RP Talu, B (通讯作者)， Inonu Univ, Fac Hlth Sci, Dept Physiotherapy & Rehabil, Malatya, Turkiye.; Korkmaz, MF (通讯作者)， Istanbul Medeniyet Univ, Fac Med, Dept Orthopaed & Traumatol, Istanbul, Turkiye.; Bazancir, Z (通讯作者)， Hacet tepe Univ, Fac Phys Therapy & Rehabil, TR-06100 Ankara, Turkiye.

EM zilanbazancir@hotmail.com; fzt.burcu@hotmail.com; dr_mfatih@yahoo.com

RI TALU, Burcu/F-1803-2016

OI TALU, Burcu/0000-0002-5623-8291

CR Akazawa Tsutomu, 2018, Eur J Orthop Surg Traumatol, V28, P177, DOI 10.1007/s00590-017-2027-4

Alanay A, 2005, SPINE, V30, P2464, DOI 10.1097/01.brs.0000184366.71761.84

Aresti N, 2017, BMJ-BRIT MED J, V359, DOI 10.1136/bmj.j4431

Asher M, 2003, SPINE, V28, P63, DOI 10.1097/00007632-200301010-00015

Bijur PE, 2001, ACAD EMERG MED, V8, P1153, DOI 10.1111/j.1553-2712.2001.tb01132.x

Borgeat A, 2008, CURR OPIN ANESTHESIO, V21, P313, DOI 10.1097/ACO.0b013e3282f82baa

Brooks D, 2001, ARCH PHYS MED REHAB, V82, P1478, DOI 10.1053/apmr.2001.25153

de Abreu DCC, 2012, GAIT POSTURE, V36, P586, DOI 10.1016/j.gaitpost.2012.05.019

Daffner SD, 2010, SPINE, V35, P1165, DOI 10.1097/BRS.0b013e3181d88e78

Danielsson AJ, 2001, EUR SPINE J, V10, P278, DOI 10.1007/s005860100309

de Santiago HAR, 2013, SPINE J, V13, P1470, DOI 10.1016/j.spinee.2013.03.027

DUNCAN PW, 1990, J GERONTOL, V45, pM192, DOI 10.1093/geronj/45.6.M192

Engsberg JR, 2003, SPINE, V28, P1993, DOI 10.1097/01.BRS.0000087209.34602.42

Erickson MA, 2013, J PEDIATR ORTHOPED, V33, P80, DOI 10.1097/BPO.0b013e318269c537

Fletcher ND, 2017, J PEDIATR ORTHOPED, V37, P92, DOI 10.1097/BPO.0000000000000601

Fletcher ND, 2014, J CHILD ORTHOP, V8, P257, DOI 10.1007/s11832-014-0587-y

Greenwood J, 2016, SPINE, V41, pE28, DOI 10.1097/BRS.0000000000001132

Kehlet H, 2002, AM J SURG, V183, P630, DOI 10.1016/S0002-9610(02)00866-8

Kehlet H, 2005, BRIT J SURG, V92, P3, DOI 10.1002/bjs.4841

Kernc D, 2018, J ORTHOP SURG RES, V13, DOI 10.1186/s13018-018-0853-7

Kim Min-Jae, 2017, Physical therapy rehabilitation science, V6, P113, DOI 10.14474/ptrs.2017.6.3.113

Kurapati Nikhil T, 2016, Spine Deform, V4, P432, DOI 10.1016/j.jspd.2016.08.004

Langelotz C, 2005, ACTA CHIR BELG, V105, P555, DOI 10.1080/00015458.2005.11679780

Lorente A, 2017, SPINE, V42, P1391, DOI 10.1097/BRS.0000000000002105

Martin CT, 2014, SPINE, V39, P1676, DOI 10.1097/BRS.0000000000000501

Maruyama T, 2008, SCOLIOSIS SPINAL DIS, V3, DOI 10.1186/1748-7161-3-6

Muhly WT, 2016, PEDIATRICS, V137, DOI 10.1542/peds.2015-1568

Negrini S, 2008, DISABIL REHABIL, V30, P731, DOI 10.1080/09638280801889485

Nielsen PR, 2010, CLIN REHABIL, V24, P137, DOI 10.1177/0269215509347432

Oestergaard LG, 2013, SPINE, V38, P1979, DOI 10.1097/BRS.0b013e3182a7902c

Ozalevli S, 2007, RESP MED, V101, P286, DOI 10.1016/j.rmed.2006.05.007

Schimmel JJP, 2015, SCOLIOSIS SPINAL DIS, V10, DOI 10.1186/s13013-015-0042-y

Thomas JJ, 2018, PEDIATR ANESTH, V28, P558, DOI 10.1111/pan.13398

WEISS HR, 1991, SPINE, V16, P88, DOI 10.1097/00007632-199101000-00016

Yen TC, 2016, J APPL BIOMECH, V32, P316, DOI 10.1123/jab.2015-0246

Yoshihara H, 2014, SPINE, V39, P1144, DOI 10.1097/BRS.0000000000000354

NR 36

TC 0

Z9 0

U1 1

U2 1

PU ELSEVIER

PI AMSTERDAM

PA RADARWEG 29, 1043 NX AMSTERDAM, NETHERLANDS

SN 0949-2658

EI 1436-2023

J9 J ORTHOP SCI

JI J. Orthop. Sci.

PD MAR

PY 2023

VL 28

IS 2

BP 308

EP 314

DI 10.1016/j.jos.2021.11.017

EA MAR 2023

PG 7

WC Orthopedics

WE Science Citation Index Expanded (SCI-EXPANDED)

SC Orthopedics

GA G9FX5

UT WOS:000992139900001

PM 34922807

DA 2023-08-10

ER

PT J

AU Schreiber, S

Parent, EC

Kawchuk, GN

Hedden, DM

AF Schreiber, Sanja

Parent, Eric C.

Kawchuk, Gregory N.

Hedden, Douglas M.

TI Algorithm for Schroth-Curve-Type Classification of Adolescent Idiopathic

Scoliosis: An Intra- and Inter-Rater Reliability Study

SO CHILDREN-BASEL

LA English

DT Article

DE scoliosis; algorithms; classification; posture; reliability;

reproducibility of results; Schroth; adolescent

ID INTEROBSERVER

AB Schroth exercises for scoliosis are prescribed based on curve types. This study aimed to determine the reliability of an algorithm for classifying Schroth curve types. Forty-four consecutive volunteers with adolescent idiopathic scoliosis, 10 to 18 years old, with curves 10 degrees to 50 degrees, were recruited from a scoliosis clinic. Their standing posture and Adam's bending test were videotaped. Ten consecutive Schroth therapist volunteers from an international registry independently classified the curve types using the proposed classification algorithm. Videos were rated twice at least seven days apart. Reliability was calculated using the Gwet's AC1 agreement coefficient for all the raters and for subgroups reporting full understanding (well-trained) and with prior algorithm experience. The intra-rater and weighted agreement coefficients for all the raters were 0.64 (95% CI: 0.53-0.73) and 0.75 (0.63-0.84), respectively. For the well-trained raters, they were 0.70 (0.60-0.78) and 0.82 (0.73-0.88), respectively; for the experienced raters, they were 0.81 (0.77-0.85) and 0.89 (0.80-0.94), respectively. The inter-rater versus weighted agreement coefficients for all the raters were 0.43 (0.28-0.58) versus 0.48 (0.29-0.67). For the well-trained raters, they were 0.50 (0.38-0.61) versus 0.61 (0.49-0.72), and for the experienced raters, they were 0.67 (0.50-0.85) versus 0.79 (0.64-0.94). Full understanding and experience led to higher reliability. Use of the algorithm can help standardize Schroth exercise treatment.

C1 [Schreiber, Sanja; Parent, Eric C.; Kawchuk, Gregory N.] Univ Alberta, Dept Phys Therapy, 8205 114 St,2-50 Corbett Hall, Edmonton, AB T6G 2G4, Canada.

[Hedden, Douglas M.] Univ Alberta, Dept Surg, 8440 112 St,2D2 24 WMC, Edmonton, AB T6G 2R7, Canada.

C3 University of Alberta; University of Alberta

RP Parent, EC (通讯作者)， Univ Alberta, Dept Phys Therapy, 8205 114 St,2-50 Corbett Hall, Edmonton, AB T6G 2G4, Canada.

EM eparent@ualberta.ca

OI Schreiber, Sanja/0000-0002-8231-5131; Parent, Eric/0000-0003-3835-0607

CR Asher MA, 2006, SCOLIOSIS SPINAL DIS, V1, DOI 10.1186/1748-7161-1-2

Coelho DM, 2013, BRAZ J PHYS THER, V17, P179, DOI 10.1590/S1413-35552012005000081

Cummings RJ, 1998, J BONE JOINT SURG AM, V80A, P1107

FEINSTEIN AR, 1990, J CLIN EPIDEMIOL, V43, P543, DOI 10.1016/0895-4356(90)90158-L

Grivas TB, 2010, SCOLIOSIS SPINAL DIS, V5, DOI 10.1186/1748-7161-5-23

Gwet K.L., 2012, HDB INTERRATER RELIA, P279

Hennes A., SCHROTH METHOD MANUA

Hennes A., 2023, INT SCHROTH 3D SCO 1

KING HA, 1983, J BONE JOINT SURG AM, V65, P1302, DOI 10.2106/00004623-198365090-00012

Kuru T, 2016, CLIN REHABIL, V30, P181, DOI 10.1177/0269215515575745

Lehnert-Schroth C., 2007, 3 DIMENSIONAL TREATM, V7th ed.

Lenke LG, 2002, SPINE, V27, P604, DOI 10.1097/00007632-200203150-00008

Lenke LG, 2001, J BONE JOINT SURG AM, V83A, P1169, DOI 10.2106/00004623-200108000-00006

Marti CL, 2015, SCOLIOSIS SPINAL DIS, V10, DOI 10.1186/s13013-015-0041-z

Minick KI, 2010, J STRENGTH COND RES, V24, P479, DOI 10.1519/JSC.0b013e3181c09c04

Munro A, 2012, J SPORT REHABIL, V21, P7, DOI 10.1123/jsr.21.1.7

Ogon M, 2002, SPINE, V27, P858, DOI 10.1097/00007632-200204150-00014

Phan P, 2010, SPINE, V35, P1054, DOI 10.1097/BRS.0b013e3181bf280e

Qiu GX, 2008, SPINE, V33, pE836, DOI 10.1097/BRS.0b013e318187bb10

Rigo MD, 2010, SCOLIOSIS SPINAL DIS, V5, DOI 10.1186/1748-7161-5-1

Ross DH, 2014, PHYSIOTHER THEOR PR, V30, P581, DOI 10.3109/09593985.2014.929767

Schreiber S, 2016, PLOS ONE, V11, DOI 10.1371/journal.pone.0168746

Schreiber S, 2015, SCOLIOSIS SPINAL DIS, V10, DOI 10.1186/s13013-015-0048-5

Schreiber Sanja, 2014, J Physiother, V60, P234, DOI 10.1016/j.jphys.2014.08.005

Stokes IAF, 2006, SPINE, V31, P665, DOI 10.1097/01.brs.0000203708.49972.ab

Weinstein SL, 2008, LANCET, V371, P1527, DOI 10.1016/S0140-6736(08)60658-3

Weiss HR, 2010, SCOLIOSIS SPINAL DIS, V5, DOI 10.1186/1748-7161-5-19

Zhou ZW, 2021, COMPLEMENT THER MED, V58, DOI 10.1016/j.ctim.2021.102697

NR 28

TC 0

Z9 0

U1 4

U2 4

PU MDPI

PI BASEL

PA ST ALBAN-ANLAGE 66, CH-4052 BASEL, SWITZERLAND

EI 2227-9067

J9 CHILDREN-BASEL

JI Children-Basel

PD MAR

PY 2023

VL 10

IS 3

AR 523

DI 10.3390/children10030523

PG 11

WC Pediatrics

WE Science Citation Index Expanded (SCI-EXPANDED)

SC Pediatrics

GA A6WZ1

UT WOS:000956518000001

PM 36980080

OA Green Published, gold

DA 2023-08-10

ER

PT J

AU Tucker, S

Heneghan, NR

Alamrani, S

Rushton, A

Gardner, A

Soundy, A

AF Tucker, Susanna

Heneghan, Nicola R.

Alamrani, Samia

Rushton, Alison

Gardner, Adrian

Soundy, Andrew

TI Barriers and facilitators of physical function, activity, sports and exercise in children and adolescents with spinal pain: a protocol for a systematic review and meta-ethnography

SO BMJ OPEN

LA English

DT Review

DE rehabilitation medicine; paediatric orthopaedics; sports medicine;

qualitative research

ID IDIOPATHIC SCOLIOSIS; SEARCH STRATEGIES; CORD-INJURY; PARTICIPATION;

HEALTH

AB IntroductionSpinal pain is one of the leading causes of disability, with the incidence of adolescent back pain estimated at 20%. Multiple barriers influence exercise participation in adolescents. However, there remains a lack of literature surrounding patients' choice to exercise, perceived barriers and facilitators of exercise, and their relationship to participant demographics. The aim of this systematic review with meta-ethnography will be to identify the barriers and facilitators of exercise participation among adolescents with thoracic or lower back pain (LBP). The secondary aim will be to identify any trends in barriers and facilitators of exercise between different demographic groups within children or adolescents under 18 years.Methods and analysisThe seven-phase process identified by Noblit and Hare's meta-ethnography approach will be used. A comprehensive electronic search of databases (AMED, CINAHLplus, EMBASE, MEDLINE, SCOPUS, Nursing & Allied Health, PubMed, PsycINFO, SPORTDiscus, Social Science Database) will be completed during April 2022. Grey literature using reference lists, websites and search engines will also be searched in accordance with Peer Review of Electronic Search Strategies (PRESS) guidelines. Inclusion criteria include: (A) qualitative studies, (B) participants under 18 years experiencing thoracic or LBP, (C) identification of barriers and facilitators of exercise participation in exercise, sports or physical activity and (D) primary research. This systematic review with meta-ethnography review aims to generate theories of behaviours and interpret significance across multiple studies. This process aims to develop future physiotherapeutic behavioural interventions, inform service provision and identify possible future research questions.Ethics and disseminationNo ethical approval was required due to the nature of using previously published work to form a systematic review paper. This systematic review and meta-ethnography will be disseminated through both conference presentations and journal publications. No funding was received for this review.PROSPERO registration numberCRD42022314796.

C1 [Tucker, Susanna; Heneghan, Nicola R.; Soundy, Andrew] Univ Birmingham, Sch Sport Exercise & Rehabil Sci, Birmingham, united kingdom.

[Alamrani, Samia] Univ Birmingham, Ctr Precis Rehabil Spinal Pain, Sch Sport Exercise & Rehabil Sci, Birmingham, united kingdom.

[Rushton, Alison] Western Univ, Sch Phys Therapy, Fac Hlth Sci, London, ON, Canada.

[Gardner, Adrian] Royal Orthopaed Hosp NHS Fdn Trust, Spinal Surg, Birmingham, united kingdom.

C3 University of Birmingham; University of Birmingham; Western University

(University of Western Ontario); Royal Orthopaedic Hospital

RP Tucker, S (通讯作者)，Univ Birmingham, Sch Sport Exercise & Rehabil Sci, Birmingham, united kingdom.

EM sxm1131@student.bham.ac.uk

RI R Heneghan, Nicola/B-2647-2016

OI R Heneghan, Nicola/0000-0001-7599-3674; Alamrani,

Samia/0000-0003-4099-8381

FU University of Birmingham (UoB) School of Sport Exercise and

Rehabilitation Sciences

FX Support was given from the University of Birmingham (UoB) School of

Sport Exercise and Rehabilitation Sciences.

CR Allender S, 2006, HEALTH EDUC RES, V21, P826, DOI 10.1093/her/cyl063

[Anonymous], 2000, PHYSIOTHERAPY, DOI DOI 10.1016/S0031-9406(05)61207-2

Anwer S, 2015, BIOMED RES INT-UK, V2015, DOI 10.1155/2015/123848

Aromataris E., 2020, JBI MANUAL EVIDENCE, DOI [DOI 10.46658/JBIMES-20-01, 10.46658/jbimes-20-01, 10.46658/JBIMES-20-01]

Atkins S, 2008, BMC MED RES METHODOL, V8, DOI 10.1186/1471-2288-8-21

Bettany-Saltikov J, 2015, COCHRANE DB SYST REV, DOI 10.1002/14651858.CD010663.pub2

Bloemen MAT, 2015, BMC NEUROL, V15, DOI 10.1186/s12883-015-0265-9

Bramer W, 2017, J MED LIBR ASSOC, V105, P285, DOI 10.5195/jmla.2017.183

Briggs AM, 2009, BMC MUSCULOSKEL DIS, V10, DOI 10.1186/1471-2474-10-77

Briggs The Joann, 2017, CRITICAL APPRAISAL T

CASPERSEN CJ, 1985, PUBLIC HEALTH REP, V100, P126

Choudhry Muhammad Naghman, 2016, Open Orthop J, V10, P143, DOI 10.2174/1874325001610010143

Diarbakerli E, 2016, SCOLIOSIS SPINAL DIS, V11, DOI 10.1186/s13013-016-0082-y

Du CP, 2016, J REHABIL MED, V48, P806, DOI 10.2340/16501977-2147

Duncan EAS., 2004, BR J OCCUPAT THER, V67, P453

Fayaz A, 2016, BMJ OPEN, V6, DOI 10.1136/bmjopen-2015-010364

France EF, 2019, BMC MED RES METHODOL, V19, DOI 10.1186/s12874-019-0670-7

France EF, 2014, BMC MED RES METHODOL, V14, DOI 10.1186/1471-2288-14-119

Geneen LJ, 2017, COCHRANE DB SYST REV, DOI [10.1002/14651858.CD011279.pub2, 10.1002/14651858.CD011279.pub3]

Gopalakrishnan S, 2013, J Family Med Prim Care, V2, P9, DOI 10.4103/2249-4863.109934

Hannes K, 2010, QUAL HEALTH RES, V20, P1736, DOI 10.1177/1049732310378656

Hayden JA, 2021, COCHRANE DB SYST REV, DOI 10.1002/14651858.CD009790.pub2

Higgins JPT., 2019, COCHRANE HDB SYSTEMA

JBI, 2022, CRITICAL APPRAISAL C

Jones GT, 2005, ARCH DIS CHILD, V90, P312, DOI 10.1136/adc.2004.056812

Joseph C, 2016, PHYS THER, V96, P1372, DOI 10.2522/ptj.20150258

Lasserson TJ., 2021, STARTING REV, DOI [10.1002/9781119536604, DOI 10.1002/9781119536604]

Lau R, 2015, BMJ OPEN, V5, DOI 10.1136/bmjopen-2015-009993

Levins SM, 2004, PHYS THER, V84, P496, DOI 10.1093/ptj/84.6.496

Lewin S, 2018, IMPLEMENT SCI, V13, DOI 10.1186/s13012-017-0688-3

Luben RN, 2020, BMC GERIATR, V20, DOI 10.1186/s12877-020-01573-0

Luchauer B, 2015, OTJR-OCCUP THER J RE, V35, P232, DOI 10.1177/1539449215601069

Mays N, 2000, BMJ-BRIT MED J, V320, P50, DOI 10.1136/bmj.320.7226.50

McGowan J, 2016, J CLIN EPIDEMIOL, V75, P40, DOI 10.1016/j.jclinepi.2016.01.021

Mckenzie G, 2021, DEV MED CHILD NEUROL, V63, P914, DOI 10.1111/dmcn.14830

McKenzie JE., 2021, SYNTH PRES FIND US O

Motyer GS, 2022, J PEDIATR PSYCHOL, V47, P225, DOI 10.1093/jpepsy/jsab095

Negrini S, 2018, SCOLIOSIS SPINAL DIS, V13, DOI 10.1186/s13013-017-0145-8

NHS, 2016, NAT BACK PAIN PATHW

NHS, 2021, EXERCISE

NHS, 2021, PHYS ACTIVITY GUIDEL

NICE, 2009, PHYS ACTIVITY CHILDR

Noblit GW, 1988, METAETHNOGRAPHY SYNT

Page MJ, 2021, INT J SURG, V88, DOI [10.1016/j.ijsu.2021.105906, 10.1186/s13643-021-01626-4, 10.1016/j.rec.2021.07.010, 10.1016/j.recesp.2021.06.016, 10.1016/j.jclinepi.2021.03.001, 10.1136/bmj.n71]

Porritt K, 2014, AM J NURS, V114, P47, DOI 10.1097/01.NAJ.0000450430.97383.64

Riff AJ, 2018, AM J SPORT MED, V46, P2594, DOI 10.1177/0363546518776638

Romano M, 2012, COCHRANE DB SYST REV, DOI 10.1002/14651858.CD007837.pub2

Sattar R, 2021, BMC HEALTH SERV RES, V21, DOI 10.1186/s12913-020-06049-w

Shamseer L, 2015, BMJ-BRIT MED J, V349, DOI [10.1136/bmj.i4086, 10.1186/2046-4053-4-1, 10.1136/bmj.g7647, 10.1136/bmj.b2535]

Sjostrom R, 2011, WORK, V39, P243, DOI 10.3233/WOR-2011-1172

Thomas J, 2008, BMC MED RES METHODOL, V8, DOI 10.1186/1471-2288-8-45

Volfson Z, 2020, DISABIL HEALTH J, V13, DOI 10.1016/j.dhjo.2020.100922

Wong G, 2013, BMC MED, V11, DOI [10.1186/1741-7015-11-20, 10.1186/1741-7015-11-21]

Zhou ZW, 2021, COMPLEMENT THER MED, V58, DOI 10.1016/j.ctim.2021.102697

Zimmer L, 2006, J ADV NURS, V53, P311, DOI 10.1111/j.1365-2648.2006.03721.x

NR 55

TC 0

Z9 0

U1 1

U2 1

PU BMJ PUBLISHING GROUP

PI LONDON

PA BRITISH MED ASSOC HOUSE, TAVISTOCK SQUARE, LONDON WC1H 9JR, united kingdom

SN 2044-6055

J9 BMJ OPEN

JI BMJ Open

PD MAR

PY 2023

VL 13

IS 3

DI 10.1136/bmjopen-2022-063946

PG 8

WC Medicine, General & Internal

WE Science Citation Index Expanded (SCI-EXPANDED)

SC General & Internal Medicine

GA I5OY6

UT WOS:001003285100081

PM 36927588

OA Green Published, gold

DA 2023-08-10

ER

PT J

AU Colak, TK

Akcay, B

Apti, A

Colak, I

AF Colak, Tugba Kuru

Akcay, Burcin

Apti, Adnan

Colak, Ilker

TI The Effectiveness of the Schroth Best Practice Program and Cheneau-Type

Brace Treatment in Adolescent Idiopathic Scoliosis: Long-Term Follow-Up

Evaluation Results

SO CHILDREN-BASEL

LA English

DT Article

DE adolescents; brace; exercise; scoliosis

ID RISSER SIGN; PUBERTAL CHANGES; INDICATORS; EXERCISES; PATTERN; CURVE

AB Background: Although the number of studies showing the efficacy of conservative treatment in adolescent idiopathic scoliosis has increased, studies with long-term follow-up are very limited. The aim of this study was to present the long-term effects of a conservative management method including exercise and brace in adolescent idiopathic scoliosis patients. Methods: This retrospective cohort study included patients with idiopathic scoliosis who presented at our department and were followed up for at least 2 years after completing the treatment. The main outcome measurements were the Cobb angle and angle of trunk rotation (ATR). Results: The cohort participants were 90.4% female, with a mean age of 11 years and the maximum Cobb angle was mean 32.1 degrees. The mean post-treatment follow-up period was 27.8 months (range 24-71 months). The improvements after treatment in mean maximum Cobb angle ($p < 0.001$) and ATR ($p = 0.001$) were statistically significant. At the end of treatment, the maximum Cobb angle was improved in 88.1% of the patients and worsened in 11.9% compared to baseline. In the long-term follow-up evaluations, 83.3% of the curvatures remained stable. Conclusions: The results of this study showed that moderate idiopathic scoliosis in growing adolescents can be successfully halted with appropriate conservative treatment and that long-term improvement is largely maintained.

C1 [Colak, Tugba Kuru] Marmara Univ, Fac Hlth Sci, Dept Physiotherapy & Rehabil, TR-34854 Istanbul, Turkiye.

[Akcay, Burcin] Bandirma Onyedi Eylul Univ, Fac Hlth Sci, Dept Physiotherapy & Rehabil, TR-10200 Bandirma, Turkiye.

[Apti, Adnan] Istanbul Kultur Univ, Fac Hlth Sci, Dept Physiotherapy & Rehabil, TR-34191 Istanbul, Turkiye.

[Colak, Ilker] VM Med Pk Maltepe Hosp, Dept Orthopaed & Traumatol, TR-34846 Istanbul, Turkiye.

C3 Marmara University; Bandirma Onyedi Eylul University; Istanbul Kultur University

RP Colak, TK (通讯作者)， Marmara Univ, Fac Hlth Sci, Dept Physiotherapy & Rehabil, TR-34854 Istanbul, Turkiye.

EM tugba.colak@marmara.edu.tr

RI Akçay, Burçin/IQS-2669-2023

OI Akçay, Burçin/0000-0002-0883-0311

CR Akcay B, 2021, SOUTH AFR J PHYSIOTH, V77, DOI 10.4102/sajp.v77i2.1568

AMENDT LE, 1990, PHYS THER, V70, P108, DOI 10.1093/ptj/70.2.108

Asher MA, 2006, SCOLIOSIS SPINAL DIS, V1, DOI 10.1186/1748-7161-1-2

Aulisa AG, 2019, EUR J PHYS REHAB MED, V55, P231, DOI 10.23736/S1973-9087.18.04782-2

Berdishevsky H, 2016, SCOLIOSIS SPINAL DIS, V11, DOI 10.1186/s13013-016-0076-9

Bettany-Saltikov J, 2015, COCHRANE DB SYST REV, DOI 10.1002/14651858.CD010663.pub2

Fadzan Maja, 2017, Open Orthop J, V11, P1466, DOI 10.2174/1874325001711011466

Fang MQ, 2022, DISABIL REHABIL, V44, P5060, DOI 10.1080/09638288.2021.1922521

Herring J.A., 2002, TACHDJIANS PEDIAT OR, V3rd ed., P213

Hoppenfeld S, 2004, SPINE, V29, P47, DOI 10.1097/01.BRS.0000103941.50129.66

Kotwicki T, 2008, STUD HEALTH TECHNOL, V140, P44, DOI 10.3233/978-1-58603-888-5-44

Kotwicki T, 2013, ADOLESC HEALTH MED T, V4, P59, DOI 10.2147/AHMT.S32088

Kotwicki T, 2008, EUR SPINE J, V17, P1676, DOI 10.1007/s00586-008-0794-7

Kuru T, 2016, CLIN REHABIL, V30, P181, DOI 10.1177/0269215515575745

Little DG, 2000, J BONE JOINT SURG AM, V82A, P685, DOI 10.2106/00004623-200005000-00009

LITTLE DG, 1994, J PEDIATR ORTHOPED, V14, P569, DOI 10.1097/01241398-199409000-00003

Lonstein J, 1995, MOES TXB SCOLIOSIS O, P219

LONSTEIN JE, 1984, J BONE JOINT SURG AM, V66A, P1061, DOI 10.2106/00004623-198466070-00013

Lonstein JE, 2006, CLIN ORTHOP RELAT R, P248, DOI 10.1097/01.blo.0000198725.54891.73

MACLEAN WE, 1989, J PEDIATR ORTHOPED, V9, P257

MARSHALL WA, 1969, ARCH DIS CHILD, V44, P291, DOI 10.1136/adc.44.235.291

MARSHALL WA, 1970, ARCH DIS CHILD, V45, P13, DOI 10.1136/adc.45.239.13

Moramarco M, 2020, SCHROTHS TXB SCOLIOS

Negrini S, 2018, SCOLIOSIS SPINAL DIS, V13, DOI 10.1186/s13013-017-0145-8

Negrini S, 2014, BMC MUSCULOSKEL DIS, V15, DOI 10.1186/1471-2474-15-263

Negrini S, 2009, SCOLIOSIS SPINAL DIS, V4, DOI 10.1186/1748-7161-4-19

Ng SY, 2016, CURR PEDIATR REV, V12, P6, DOI 10.2174/1573396312666151117120056

Richards BS, 2005, SPINE, V30, P2068, DOI 10.1097/01.brs.0000178819.90239.d0

Rigo M, 2017, SCOLIOSIS SPINAL DIS, V12, P1, DOI 10.1186/s13013-017-0114-2

RISSER J C, 1958, Clin Orthop, V11, P111

Weiss HR, 2021, SOUTH AFR J PHYSIOTH, V77, DOI 10.4102/sajp.v77i2.1573

Weiss Hans-Rudolf, 2019, J Phys Ther Sci, V31, P983, DOI 10.1589/jpts.31.983

Weiss Hans-Rudolf, 2002, Stud Health Technol Inform, V91, P342

Weiss Hans-Rudolf, 2017, Open Orthop J, V11, P1558, DOI 10.2174/1874325001711011558

Weiss HR, 2015, SCHROTH THERAPY ADV

Zaina F, 2009, SCOLIOSIS SPINAL DIS, V4, DOI 10.1186/1748-7161-4-8

NR 36

TC 0

Z9 0

U1 1

U2 1

PU MDPI

PI BASEL

PA ST ALBAN-ANLAGE 66, CH-4052 BASEL, SWITZERLAND

EI 2227-9067

J9 CHILDREN-BASEL

JI Children-Basel

PD FEB

PY 2023

VL 10

IS 2

AR 386

DI 10.3390/children10020386

PG 11

WC Pediatrics

WE Science Citation Index Expanded (SCI-EXPANDED)

SC Pediatrics

GA 9G9JK

UT WOS:000938460200001

PM 36832515

OA Green Published, gold

DA 2023-08-10

ER

PT J

AU Dimitrijevic, V

Viduka, D

Scepanovic, T

Maksimovic, N

Giustino, V

Bianco, A

Drid, P

AF Dimitrijevic, Vanja

Viduka, Dejan

Scepanovic, Tijana

Maksimovic, Nebojsa

Giustino, Valerio

Bianco, Antonino

Drid, Patrik

TI Effects of Schroth method and core stabilization exercises on idiopathic

scoliosis: a systematic review and meta-analysis

SO EUROPEAN SPINE JOURNAL

LA English

DT Review

DE Schroth exercise; Core exercise; Idiopathic scoliosis; Physical

exercise; Body posture

ID COBB ANGLE; ADOLESCENTS

AB Purpose Conservative approaches such as Schroth exercises and core stabilization exercises showed effective results in the treatment of idiopathic scoliosis. This study aimed to critically evaluate the magnitude effect of Schroth and core stabilization exercises using a systematic review and meta-analysis. Methods This study has been developed in accordance with the Preferred Reporting Items for Systematic Reviews and Meta-Analyses (PRISMA) Statement. The following three databases were searched for articles collection: Web of Science, PubMed, and Google Scholar. The key search terms were: Schroth exercise, core exercise, idiopathic scoliosis, Cobb angle, angle of trunk rotation, and quality of life. The articles included in our study was limited to original articles written only in English that met the following inclusion criteria: (1) Participants with idiopathic scoliosis; (2) Schroth exercises and core stabilization exercises used as interventions; (3) Cobb angle or angle of trunk rotation or quality of life used as outcome measures. Results Thirteen studies were

included. Depending on the outcome measured, the magnitude effect of Schroth and core stabilization exercises ranged from small to almost large as follows: Cobb angle (SMD = - 0.376, p < 0.001); angle of trunk rotation (SMD = - 0.268, p = 0.104); quality of life (SMD = 0.774, p = 0.002). By subgroup analysis, Schroth method showed a larger effect size than core stabilization exercises. Conclusion The current systematic review and meta-analysis indicates that both Schroth method and core stabilization exercises have a positive effect in subjects with idiopathic scoliosis. Subgroup analysis showed that the Schroth method had a larger effect size than the core stabilization exercises.

C1 [Dimitrijevic, Vanja; Scepanovic, Tijana; Maksimovic, Nebojsa; Drid, Patrik] Univ Novi Sad, Fac Sports & Phys Educ, Novi Sad, Serbia.

[Viduka, Dejan] Univ Business Acad, Fac Appl Management Econ & Finance Belgrade, Novi Sad, Serbia.

[Giustino, Valerio; Bianco, Antonino] Univ Palermo, Dept Psychol Educ Sci & Human Movement, Sport & Exercise Sci Res Unit, Via Giovanni Pascoli 6, I-90144 Palermo, Italy.

C3 University of Novi Sad; University of Palermo

RP Giustino, V (通讯作者)， Univ Palermo, Dept Psychol Educ Sci & Human Movement, Sport & Exercise Sci Res Unit, Via Giovanni Pascoli 6, I-90144 Palermo, Italy.

EM valerio.giustino@unipa.it

RI Drid, Patrik/P-5604-2015

OI Drid, Patrik/0000-0002-2075-6038; Viduka, Dejan/0000-0001-9147-8103;

Giustino, Valerio/0000-0002-4575-8021; Dimitrijevic,

Vanja/0000-0003-3180-336X

FU Universita degli Studi di Palermo within the CRUI-CARE Agreement

FX Open access funding provided by Universita degli Studi di Palermo within

the CRUI-CARE Agreement. No funding was received for conducting this

study.

CR Adamczewska K, 2019, INT J ENV RES PUB HE, V16, DOI 10.3390/ijerph16183426

Ayhan C, 2014, CLIN REHABIL, V28, P36, DOI 10.1177/0269215513492443

Bezalel T, 2019, ASIAN SPINE J, V13, P490, DOI 10.31616/asj.2018.0097

Day Joseph M, 2019, Arch Physiother, V9, P8, DOI 10.1186/s40945-019-0060-9

Duangkeaw R., 2019, WALAILAK J SCI TECHN, V16, P965, DOI [10.48048/wjst.2019.4255, DOI 10.48048/WJST.2019.4255]

Farooqui Sumaira Imran, 2018, Int J Health Sci (Qassim), V12, P44

Gur G, 2017, PROSTHET ORTHOT INT, V41, P303, DOI 10.1177/0309364616664151

Health N Council MR, 2009, NHMRC ADD LEV EV GRA

Higgins JPT., 2019, COCHRANE HDB SYSTEMA

Kim G, 2016, J PHYS THER SCI, V28, P1012, DOI 10.1589/jpts.28.1012

Kim Min-Jae, 2017, Physical therapy rehabilitation science, V6, P113, DOI 10.14474/ptrs.2017.6.3.113

Ko KJ, 2017, J EXERC REHABIL, V13, P244, DOI 10.12965/jer.1734952.476

Kocaman H, 2021, PLOS ONE, V16, DOI 10.1371/journal.pone.0249492

Kuru T, 2016, CLIN REHABIL, V30, P181, DOI 10.1177/0269215515575745

Lenhert-Schroth C., 1992, PHYSIOTHERAPY, V78, P810, DOI [10.1016/S0031-9406(10)60451-8, DOI 10.1016/S0031-9406(10)60451-8]

Liu DL, 2020, SPINE, V45, P1039, DOI 10.1097/BRS.0000000000003451

Moher D, 2015, SYST REV-LONDON, V4, DOI [10.1136/bmj.b2535, 10.1186/s13643-015-0087-2, 10.1016/j.ijsu.2010.02.007, 10.1016/j.ijsu.2010.07.299]

Monticone M, 2014, EUR SPINE J, V23, P1204, DOI 10.1007/s00586-014-3241-y

Muthukrishnan R, 2010, BMC SPORTS SCI MED R, V2, DOI 10.1186/1758-2555-2-13

Negrini A, 2016, SCOLIOSIS SPINAL DIS, V11, DOI 10.1186/s13013-016-0100-0

Negrini S, 2015, SCOLIOSIS SPINAL DIS, V10, DOI 10.1186/s13013-014-0025-4

Park JH, 2018, EUR J PHYS REHAB MED, V54, P440, DOI 10.23736/S1973-9087.17.04461-6

Park SW., 2021, ANNAL ROMANIAN SOC C, V25, P922

Park YH, 2016, J INT MED RES, V44, P728, DOI 10.1177/0300060516639750

Schreiber S, 2019, BMC MUSCULOSKEL DIS, V20, DOI 10.1186/s12891-019-2695-9

Schreiber S, 2016, PLOS ONE, V11, DOI 10.1371/journal.pone.0168746

Schreiber S, 2015, SCOLIOSIS SPINAL DIS, V10, DOI 10.1186/s13013-015-0048-5

Vrecic A., 2020, MEDICINSKI PODMLADAK, V71, P33, DOI [10.5937/mp71-22170, DOI 10.5937/MP71-22170]

WASSERTHEIL S, 1970, BIOMETRICS, V26, P588, DOI 10.2307/2529115

Weinstein SL, 2013, NEW ENGL J MED, V369, P1512, DOI 10.1056/NEJMoa1307337

Yagci G, 2019, PROSTHET ORTHOT INT, V43, P301, DOI 10.1177/0309364618820144

황보필녀, 2016, [The Journal of Korean Society of Physical Therapy, 대한물리치료학회지], V28, P364

NR 32

TC 0

Z9 0

U1 12

U2 21

PU SPRINGER

PI NEW YORK

PA ONE NEW YORK PLAZA, SUITE 4600, NEW YORK, NY, UNITED STATES

SN 0940-6719

EI 1432-0932

J9 EUR SPINE J

JI Eur. Spine J.

PD DEC

PY 2022

VL 31

IS 12

BP 3500

EP 3511

DI 10.1007/s00586-022-07407-4

EA OCT 2022

PG 12

WC Clinical Neurology; Orthopedics

WE Science Citation Index Expanded (SCI-EXPANDED)

SC Neurosciences & Neurology; Orthopedics

GA 8H0RE

UT WOS:000867557300001

PM 36229615

OA hybrid

DA 2023-08-10

ER

PT J

AU Gheitasi, M

Bayattork, M

Kolur, MK

AF Gheitasi, Mehdi

Bayattork, Mohammad

Kolur, Masoud Khorsandi

TI Adding corrective exercises along with bracing for postural

hyperkyphosis among adolescents: A randomized controlled trial

SO PM&R

LA English

DT Article

ID SPINE-STRENGTHENING EXERCISE; THORACIC KYPHOSIS; MILWAUKEE BRACE;

OLDER-ADULTS; SCOLIOSIS; REHABILITATION; PROGRAM

AB Background Although most attention has been paid to the effectiveness of corrective exercises and bracing to improve thoracic hyperkyphosis, no study, to the best of our knowledge, has compared the effects of exercises plus bracing to bracing alone. Objective The present study aims to compare the effect of corrective exercises and bracing and bracing alone on the Cobb angle of thoracic kyphosis. Design A parallel-group, blinded, randomized, controlled trial. Participants The participants included 180 adolescents with thoracic hyperkyphosis who were randomized into corrective exercises plus bracing (N = 60), only bracing (N = 60), and a waitlist control group (N = 60). Intervention Intervention groups received their programs for 24 weeks, whereas the control group was placed on a waitlist for 6 months before receiving a delayed intervention. Main Outcome Measurements The primary outcome changed from baseline to 6 months in Cobb angle measured from standing lateral spine radiographs. Results Significant differences were observed in the Cobb angle of thoracic kyphosis between all groups at the posttest (p = .0001). In post hoc analyses, we found significant differences between the corrective exercise plus bracing group (15.46_ decline) and the bracing only group (8.75_ decline), p = .0001; both groups had bigger improvements than the control group, which increased by 1.25_ (p = .00001). Conclusion The combination of corrective exercises and bracing demonstrated better overall outcomes in Cobb angle compared to only bracing, as well as waitlist control in adolescents with thoracic hyperkyphosis.

C1 [Gheitasi, Mehdi] Shahid Beheshti Univ, Fac Sport Sci & Hlth, Hlth & Sport Rehabil Dept, Tehran, Iran.

[Bayattork, Mohammad; Kolur, Masoud Khorsandi] Univ Hormozgan, Fac Humanities Sci, Sport Sci & Phys Educ, Bandar Abbas 7916193145, Iran.

C3 Shahid Beheshti University; University of Hormozgan

RP Bayattork, M (通讯作者)， Univ Hormozgan, Fac Humanities Sci, Sport Sci & Phys Educ, Bandar Abbas 7916193145, Iran.

EM mohammadbayat82@gmail.com

RI Bayattork, Mohammad/AAV-8567-2020

OI Bayattork, Mohammad/0000-0002-7588-0079; Khorsandi Kolur,

Masoud/0000-0003-2319-8489

CR Babaee T, 2017, ASIAN SPINE J, V11, P627, DOI 10.4184/asj.2017.11.4.627

Bansal S, 2014, ARCH PHYS MED REHAB, V95, P129, DOI 10.1016/j.apmr.2013.06.022

Barczyk-Pawelec K, 2015, J MANIP PHYSIOL THER, V38, P484, DOI 10.1016/j.jmpt.2015.06.010

Bettany-Saltikov J, 2016, ASIAN SPINE J, V10, P1170, DOI 10.4184/asj.2016.10.6.1170

CARMAN DL, 1990, J BONE JOINT SURG AM, V72A, P328, DOI 10.2106/00004623-199072030-00003

Coelho DM, 2013, BRAZ J PHYS THER, V17, P179, DOI 10.1590/S1413-35552012005000081

Czaprowski D, 2014, MANUAL THER, V19, P392, DOI 10.1016/j.math.2013.10.005

Feng Q, 2018, CLIN REHABIL, V32, P48, DOI 10.1177/0269215517714591

Gonzalez-Galvez N, 2019, PLOS ONE, V14, DOI 10.1371/journal.pone.0216180

Greendale GA, 2011, OSTEOPOROSIS INT, V22, P1897, DOI 10.1007/s00198-010-1422-z

Greendale GA, 2009, J AM GERIATR SOC, V57, P1569, DOI 10.1111/j.1532-5415.2009.02391.x

Hart ES, 2010, ORTHOP NURS, V29, P365, DOI 10.1097/NOR.0b013e3181f83761

Hodges PW., 2013, SPINAL CONTROL REHAB

Hrysomallis C, 2001, J STRENGTH COND RES, V15, P385

Katzman WB, 2017, OSTEOPOROSIS INT, V28, P2831, DOI 10.1007/s00198-017-4109-x

Katzman WB, 2007, ARCH PHYS MED REHAB, V88, P192, DOI 10.1016/j.apmr.2006.10.033

Katzman WB, 2016, PHYS THER, V96, P371, DOI 10.2522/ptj.20150171

Lederman E., 2010, NEUROMUSCULAR REHABI, P178, DOI DOI 10.1016/B978-0-443-06969-7.00014-0

Lowe TG, 2007, NEUROSURG CLIN N AM, V18, P305, DOI 10.1016/j.nec.2007.02.011

Marks M, 2009, SPINE, V34, P949, DOI 10.1097/BRS.0b013e318199650a

Mehdikhani M, 2016, J BACK MUSCULOSKELET, V29, P515, DOI 10.3233/BMR-150651

Mehdikhani M, 2014, PROSTHET ORTHOT INT, V38, P316, DOI 10.1177/0309364613499063

Papagelopoulos PJ, 2008, ORTHOPEDICS, V31, P52, DOI 10.3928/01477447-20080101-33

Schwanke NL, 2016, MANUAL THER, V22, P138, DOI 10.1016/j.math.2015.11.004

Seidi F, 2020, SCI REP-UK, V10, DOI 10.1038/s41598-020-77571-4

Seidi F, 2014, J BACK MUSCULOSKELET, V27, P7, DOI 10.3233/BMR-130411

Vedantam R, 2000, SPINE, V25, P2204, DOI 10.1097/00007632-200009010-00011

Weiss HR, 2006, ST HEAL T, V123, P594

Wu WF, 2014, BMC MUSCULOSKEL DIS, V15, DOI 10.1186/1471-2474-15-33

Zaina F, 2009, EUR J PHYS REHAB MED, V45, P595

Zmyslna A, 2019, INT J OCCUP MED ENV, V32, P25, DOI 10.13075/ijomeh.1896.01314

NR 31

TC 0

Z9 0

U1 6

U2 8

PU WILEY

PI HOBOKEN

PA 111 RIVER ST, HOBOKEN 07030-5774, NJ USA

SN 1934-1482

EI 1934-1563

J9 PM&R

JI PM&R

PD JUL

PY 2023

VL 15

IS 7

BP 872

EP 880

DI 10.1002/pmrj.12877

EA OCT 2022

PG 9

WC Rehabilitation; Sport Sciences

WE Science Citation Index Expanded (SCI-EXPANDED)

SC Rehabilitation; Sport Sciences

GA N3PN5

UT WOS:000863902300001

PM 36197728

DA 2023-08-10

ER

PT J

AU Akyurek, E

Alpozgen, AZ

Akgul, T

AF Akyurek, Elcin

Alpozgen, Ayse Zengin

Akgul, Turgut

TI The preliminary results of physiotherapy scoliosisspecific exercises on spine joint position sense in adolescent idiopathic scoliosis: A randomized controlled trial

SO PROSTHETICS AND ORTHOTICS INTERNATIONAL

LA English

DT Article

DE scoliosis; exercise; Schroth; proprioception; spine

ID SYMMETRY INDEX POTSI; PROPRIOCEPTIVE SYSTEM

AB Background: It has been observed that there are difficulties in maintaining spinal harmony in the absence of proprioceptive information, which has an important role in achieving postural control in scoliosis. One of the tests used to evaluate proprioceptive mechanisms is joint position reproduction.

Objective(s): The main aim of our study was to investigate the effect of physiotherapy scoliosis-specific exercise (PSSE) on spine joint reposition (JR) sense. The second aim was to determine the effects of PSSE on the angle of trunk rotation (ATR), posture, and deformity perception in adolescent idiopathic scoliosis.

Methods: Twenty-nine patients with adolescent idiopathic scoliosis (mean age 13.79 6 1.82 years) were randomly allocated to two groups. "Schroth" exercises were applied to the PSSE group for 8 weeks (16 sessions). The control group was placed on the waiting list. Patients' JR error (dual inclinometer), ATR (scoliometer), posture parameters (PostureScreen Mobile, Posterior Trunk Asymmetry Index, and Anterior Trunk Asymmetry Index), and deformity perception (Walter Reed Visual Assessment Scale [WRVAS]) were assessed at the first session and at the end of 8 weeks.

Results: The initial demographic characteristics, Cobb (thoracal: 19.41 6 7.03 degrees; lumbar: 20.76 6 7.24 degrees) and ATR (thoracal: 5.8663.98 degrees; lumbar: 5.6663.33 degrees) and clinical test parameters of the groups (JR, posture parameters, and WRVAS) were similar (P..05). According to the findings, thoracal and lumbar JR error values (P,.05), ATR (P #.001), posture parameters (P #.001), and WRVAS (P 5.048) were significantly improved in the PSSE group. Besides, when the groups were compared, a significant difference was found in favor of PSSE except for the deformity perception (P,.05).

Conclusions: Schroth exercises have a positive contribution for improving JR sense of the spine, vertebral rotation, and posture.

C1 [Akyurek, Elcin] Istanbul Univ Cerrahpasa, Inst Grad Studies, Dept Physiotherapy & Rehabil, Istanbul, Turkey.

[Alpozgen, Ayse Zengin] Istanbul Univ Cerrahpasa, Fac Hlth Sci, Dept Physiotherapy & Rehabil, Alkent 2000 Mah,Yigitturk Cad 5-9-1, TR-34500 Istanbul, Turkey.

[Akgul, Turgut] Istanbul Univ, Istanbul Fac Med, Dept Orthoped & Traumatol, Istanbul, Turkey.

C3 Istanbul University - Cerrahpasa; Istanbul University - Cerrahpasa;

Istanbul University

RP Alpozgen, AZ (通讯作者)， Istanbul Univ Cerrahpasa, Fac Hlth Sci, Dept Physiotherapy & Rehabil, Alkent 2000 Mah,Yigitturk Cad 5-9-1, TR-34500 Istanbul, Turkey.

EM azengin@istanbul.edu.tr

RI Zengin Alpozgen, Ayse/E-5682-2019

OI Zengin Alpozgen, Ayse/0000-0003-0436-1164

CR Abboud J, 2018, EUR J APPL PHYSIOL, V118, P133, DOI 10.1007/s00421-017-3754-2

Assaiante C, 2012, PLOS ONE, V7, DOI 10.1371/journal.pone.0040646

Berdishevsky H, 2016, SCOLIOSIS SPINAL DIS, V11, DOI 10.1186/s13013-016-0076-9

Blecher R, 2017, DEV CELL, V42, P388, DOI 10.1016/j.devcel.2017.07.022

Bogdani A, 2016, J HUM SPORT EXERC, V11, P245

Coelho DM, 2013, BRAZ J PHYS THER, V17, P179, DOI 10.1590/S1413-35552012005000081

Colak I, 2020, J TURK SPINAL SURG, V31, P125

de Seze M., 2012, Annals of Physical and Rehabilitation Medicine, V55, P128, DOI 10.1016/j.rehab.2012.01.003

Docherty CL, 1998, J ATHL TRAINING, V33, P310

Fletcher JP, 2021, J SPORT REHABIL, V30, P339, DOI 10.1123/jsr.2019-0368

Gubbels Christopher M, 2019, J Phys Ther Sci, V31, P159, DOI 10.1589/jpts.31.159

Gur G, 2017, PROSTHET ORTHOT INT, V41, P303, DOI 10.1177/0309364616664151

Han J, 2016, J SPORT HEALTH SCI, V5, P80, DOI 10.1016/j.jshs.2014.10.004

Inami K, 1999, ST HEAL T, V59, P85

Izatt MT, 2012, SCOLIOSIS SPINAL DIS, V7, DOI 10.1186/1748-7161-7-14

Knott P, 2014, SCOLIOSIS SPINAL DIS, V9, DOI 10.1186/1748-7161-9-4

Larson JE, 2018, J ORTHOP, V15, P563, DOI 10.1016/j.jor.2018.05.032

Le Berre M, 2017, EUR SPINE J, V26, P1638, DOI 10.1007/s00586-016-4802-z

Matlega A, 2020, PHYSIOTHER THEOR PR, V36, P1502, DOI 10.1080/09593985.2019.1570574

Minguez MF, 2007, EUR SPINE J, V16, P73, DOI 10.1007/s00586-006-0079-y

Moramarco M, 2016, CURR PEDIATR REV, V12, P17, DOI 10.2174/1573396312666151117120514

Negrini S, 2018, SCOLIOSIS SPINAL DIS, V13, DOI 10.1186/s13013-017-0145-8

Noh KH, 2015, J PHYS THER SCI, V27, P293, DOI 10.1589/jpts.27.293

Park JH, 2018, EUR J PHYS REHAB MED, V54, P440, DOI 10.23736/S1973-9087.17.04461-6

do Rosario JLP, 2014, J BODYW MOV THER, V18, P56, DOI 10.1016/j.jbmt.2013.05.008

Puntumetakul Rungthip, 2018, J Phys Ther Sci, V30, P1390, DOI 10.1589/jpts.30.1390

Roberts S, 1995, SPINE, V20, P2645, DOI 10.1097/00007632-199512150-00005

Roijezon U, 2015, MANUAL THER, V20, P368, DOI 10.1016/j.math.2015.01.008

Saur PMM, 1996, SPINE, V21, P1332, DOI 10.1097/00007632-199606010-00011

Simoneau M, 2006, EXP BRAIN RES, V170, P576, DOI 10.1007/s00221-005-0246-0

Stolinski L, 2017, SCOLIOSIS SPINAL DIS, V12, DOI 10.1186/s13013-017-0146-7

Stolinski L, 2013, SCOLIOSIS SPINAL DIS, V8, P1

Stolinski L, 2012, STUD HEALTH TECHNOL, V176, P242, DOI 10.3233/978-1-61499-067-3-242

Suzuki N, 1999, ST HEAL T, V59, P81

Swinkels A, 2000, SPINE, V25, P98, DOI 10.1097/00007632-200001010-00017

Szucs Kimberly A, 2018, J Phys Ther Sci, V30, P31, DOI 10.1589/jpts.30.31

Vidal C, 2013, EUR SPINE J, V22, P1362, DOI 10.1007/s00586-013-2752-2

Weinstein SL, 2008, LANCET, V371, P1527, DOI 10.1016/S0140-6736(08)60658-3

Weiss H, 2007, BEST PRACTISE CONSER

장우남, 2013, [The Journal of Korean Society of Physical Therapy, 대한물리치료학회지], V25, P210

NR 40

TC 0

Z9 0

U1 6

U2 9

PU WOLTERS KLUWER HEALTH

PI Philadelphia

PA 2001 Market St., Philadelphia, PA, UNITED STATES

SN 0309-3646

EI 1746-1553

J9 PROSTHET ORTHOT INT

JI Prosthet. Orthot. Int.

PD OCT

PY 2022

VL 46

IS 5

BP 510

EP 517

DI 10.1097/PXR.0000000000000136

PG 8

WC Orthopedics; Rehabilitation

WE Science Citation Index Expanded (SCI-EXPANDED)

SC Orthopedics; Rehabilitation

GA 8G6FY

UT WOS:000920440300015

PM 36215059

DA 2023-08-10

ER

PT J

AU Ghafar, MAA

Abdelraouf, OR

Abdel-Aziem, AA

Elnegamy, TE

Mohamed, ME

Yehia, AM

Mousa, GS

AF Ghafar, Mohamed A. Abdel

Abdelraouf, Osama R.

Abdel-Aziem, Amr A.

Elnegamy, Tamer E.

Mohamed, Mariam E.

Yehia, Amr M.

Mousa, Gihan Samir

TI PULMONARY FUNCTION AND AEROBIC CAPACITY RESPONSES TO EQUINE-ASSISTED

THERAPY IN ADOLESCENTS WITH IDIOPATHIC SCOLIOSIS: A RANDOMIZED

CONTROLLED TRIAL

SO JOURNAL OF REHABILITATION MEDICINE

LA English

DT Article

DE equine-assisted therapy; pulmonary function; Schroth method; idiopathic

scoliosis

ID RESPIRATORY-FUNCTION; CEREBRAL-PALSY; EXERCISE; HIPPOTHERAPY;

LIMITATION; SPIROMETRY; CHILDREN; IMPACT

AB Background: Idiopathic scoliosis is a common spinal malalignment that negatively impacts the respiratory system and physical conditioning in adolescents. Equine-assisted therapy comprises therapeutic horseback riding that optimizes physical performance and mobility in a range of contexts. However, the influence of equine-assisted therapy on pulmonary function remains unclear.

Objective: To examine the impact of 10 weeks of hippotherapy combined with Schroth exercises on pulmonary function and aerobic capacity in adolescents with idiopathic scoliosis.

Methods: A randomized controlled trial including 45 patients, randomly assigned to experimental and control groups, was performed. Patients in the experimental group received 15 30-min sessions of hippotherapy over a period of 10 weeks. The 2 groups attended a 60-min session of Schroth exercises 3 times/week for 10 weeks. Pulmonary function and functional capacity were assessed before and after the intervention.

Results: Pre- and post-intervention variables (FVC, FEV1, FEV1/FVC, MVV and 6MWT) revealed significant improvement in both groups ($p < 0.05$). The improvement in the experimental group was significantly higher than in the control group ($p < 0.05$).

Conclusion: The addition of hippotherapy to Schroth exercises resulted in improved pulmonary function and aerobic capacity in adolescents with idiopathic scoliosis.

C1 [Ghafar, Mohamed A. Abdel; Abdelraouf, Osama R.; Mohamed, Mariam E.] Batterjee Med Coll, Phys Therapy Program, Jeddah, Saudi Arabia.

[Abdelraouf, Osama R.; Abdel-Aziem, Amr A.] Cairo Univ, Fac Phys Therapy, Dept Biomech, Giza, Egypt.

[Elnegamy, Tamer E.] Prince Sattam Bin Abdelaziz Univ, Dept Phys Therapy & Hlth Rehabil, Coll Appl Med Sci, Al Kharj, Saudi Arabia.

[Mohamed, Mariam E.; Mousa, Gihan Samir] Cairo Univ, Fac Phys Therapy, Dept Phys Therapy Cardiovasc Resp Disorders & Ger, Giza, Egypt.

[Yehia, Amr M.] 6th October Univ, Fac Phys Therapy, Dept Musculoskeletal Disorders & Surg, Giza, Egypt.

[Mousa, Gihan Samir] Umm Al Qura Univ, Fac Appl Med Sci, Phys Therapy Dept, Mecca, Saudi Arabia.

C3 Egyptian Knowledge Bank (EKB); Cairo University; Prince Sattam Bin

Abdulaziz University; Egyptian Knowledge Bank (EKB); Cairo University;

Egyptian Knowledge Bank (EKB); October 6 University (O6U); Umm Al Qura

University

RP Ghafar, MAA (通讯作者)， Batterjee Med Coll, Phys Therapy Program, Jeddah, Saudi Arabia.

EM pt12.jed@bmc.edu.sa

RI Abdel-aziem, amr/GQA-5184-2022; ElSayed Mohamed, Dr.

Mariam/GWU-5119-2022; Salem, Mariam Elsayed Mohamed

AbdAlaal/GQA-3370-2022; Abdel Ghafar, Mohamed A./AAY-7831-2021;

Abdelraouf, Osama Ragaa/AGJ-0828-2022; mohamed, mariam/HTS-5332-2023;

Mousa, Gihan/C-1545-2019

OI Abdel Ghafar, Mohamed A./0000-0001-7907-3613; Abdelraouf, Osama

Ragaa/0000-0001-6888-5480; Yehia, Amr/0000-0002-5428-4286; Abdel-aziem,

Amr Almaz/0000-0001-8448-9218; Salem, Mariam/0000-0002-2169-9864; Mousa,

Gihan/0000-0001-8469-9207

CR Abdelaal AAM, 2018, J INT MED RES, V46, P381, DOI 10.1177/0300060517715375

Agrawal MB, 2015, J CLIN DIAGN RES, V9, pOC1, DOI 10.7860/JCDR/2015/13181.6311

Benda W, 2003, J ALTERN COMPLEM MED, V9, P817, DOI 10.1089/107555303771952163

Borel JC, 2009, RESP PHYSIOL NEUROBI, V167, P168, DOI 10.1016/j.resp.2009.03.014

Campbell RM, 2004, J BONE JOINT SURG AM, V86A, P1659, DOI 10.2106/00004623-200408000-00009

Charles YP, 2008, SPINE, V33, P1209, DOI 10.1097/BRS.0b013e3181715272

Chu ECP, 2020, CLINICS PRACT, V10, P70, DOI 10.4081/cp.2020.1258

Costa Valéria Sovat de Freitas, 2015, Fisioter. mov., V28, P373, DOI 10.1590/0103-5150.028.002.AO18

Crapo RO, 2002, AM J RESP CRIT CARE, V166, P111, DOI 10.1164/rccm.166/1/111

Deutz U, 2018, NEUROPEDIATRICS, V49, P185, DOI 10.1055/s-0038-1635121

Alves VLD, 2006, CHEST, V130, P500, DOI 10.1378/chest.130.2.500

Graham BL, 2019, AM J RESP CRIT CARE, V200, pE70, DOI 10.1164/rccm.201908-1590ST

Grivas T B, 2002, Stud Health Technol Inform, V91, P76

Han JY, 2012, ANN REHABIL MED-ARM, V36, P762, DOI 10.5535/arm.2012.36.6.762

Hilliere C, 2018, PM&R, V10, P1062, DOI 10.1016/j.pmrj.2018.03.019

Johari J, 2016, SINGAP MED J, V57, P33, DOI 10.11622/smedj.2016009

Johnston CE, 2011, SPINE, V36, P1096, DOI 10.1097/BRS.0b013e3181f8c931

Kim HS, 2020, HEALTHCARE-BASEL, V8, DOI 10.3390/healthcare8030276

Kim KD, 2016, J PHYS THER SCI, V28, P923, DOI 10.1589/jpts.28.923

Kim Min-Jae, 2017, Physical therapy rehabilitation science, V6, P113, DOI 10.14474/ptrs.2017.6.3.113

Kim Minjoo, 2015, [The Asian Journal of Kinesiology, 아시아 운동학 학술지], V17, P21, DOI 10.15758/jkak.2015.17.4.21

Kouwenhoven JWM, 2008, SPINE, V33, P2898, DOI 10.1097/BRS.0b013e3181891751

Kuru T, 2016, CLIN REHABIL, V30, P181, DOI 10.1177/0269215515575745

Lee JiHyun, 2017, J Phys Ther Sci, V29, P779, DOI 10.1589/jpts.29.779

Lim Jae-Heon, 2016, J Phys Ther Sci, V28, P1836, DOI 10.1589/jpts.28.1836

Martinez-Llorens J, 2010, EUR RESPIR J, V36, P393, DOI 10.1183/09031936.00025509

Matusiak-Wieczorek E, 2020, INT J ENV RES PUB HE, V17, DOI 10.3390/ijerph17186846

McIntire KL, 2007, SCOLIOSIS SPINAL DIS, V2, DOI 10.1186/1748-7161-2-9

Moraes Andrea Gomes, 2016, J Phys Ther Sci, V28, P2220, DOI 10.1589/jpts.28.2220

Parent S, 2002, SPINE, V27, P2305, DOI 10.1097/00007632-200211010-00002

Pyo Seo Jeong, 2020, [The Journal of Korean Society of Physical Therapy, 대한물리치료학회지], V32, P383, DOI 10.18857/jkpt.2020.32.6.383

Rigby BR, 2017, RES Q EXERCISE SPORT, V88, P26, DOI 10.1080/02701367.2016.1266458

Shin Doo Chul, 2016, J Phys Ther Sci, V28, P2547

Snezhina L, 2019, ANN PULMONOL, V3, P33

Souza FD, 2008, ISOKINET EXERC SCI, V16, P263

Sperandio EF, 2014, SPINE J, V14, P2366, DOI 10.1016/j.spinee.2014.01.041

Sperandio Evandro Fornias, 2015, Fisioter. mov., V28, P563, DOI 10.1590/0103-5150.028.003.AO15

Wang JS, 2015, J PHYS THER SCI, V27, P3329, DOI 10.1589/jpts.27.3329

Weiss HR, 2011, SCOLIOSIS SPINAL DIS, V6, DOI 10.1186/1748-7161-6-17

NR 39

TC 0

Z9 0

U1 2

U2 6

PU FOUNDATION REHABILITATION INFORMATION

PI UPPSALA

PA TRADGARDSGATAN 14, UPPSALA, SE-753 09, SWEDEN

SN 1650-1977

EI 1651-2081

J9 J REHABIL MED

JI J. Rehabil. Med.

PD OCT

PY 2022

VL 54

AR jrm00296

DI 10.2340/jrm.v54.1085

PG 8

WC Rehabilitation; Sport Sciences

WE Science Citation Index Expanded (SCI-EXPANDED)

SC Rehabilitation; Sport Sciences

GA 6P0YL

UT WOS:000890661700053

PM 35652929

OA gold, Green Published

DA 2023-08-10

ER

PT J

AU Trzcinska, S

Koszela, K

AF Trzcinska, Sandra

Koszela, Kamil

TI Retrospective Analysis of FED Method Treatment Results in 11-17-Year-Old

Children with Idiopathic Scoliosis

SO CHILDREN-BASEL

LA English

DT Article

DE scoliosis; musculoskeletal disorders; spine; conservative treatment;

rehabilitation

ID ULTRASOUND

AB (1) Background: Idiopathic scoliosis is a major treatment problem due to its unknown origin and its three-dimensional nature. Attempts to cure it and search for new methods of physiotherapeutic treatment that would lead to its correction are one of the key issues of modern medicine. One of them is the fixation, elongation, de-rotation method (FED), used in the conservative treatment of idiopathic scoliosis. The aim of the study was evaluation of the short-term effectiveness of the FED method in the treatment of patients with idiopathic scoliosis. (2) Methods: Each patient underwent therapy based on the guidelines of the FED method. Patients were tested with the Bunnell scoliometer and the Zebris computer system. The treatment period was three weeks, after which the examinations were repeated. (3) Results: The results appeared to be statistically significant for all tested variables. (4) Conclusions: The examinations showed that the FED method had a statistically significant effect on the improvement of all parameters of posture examination, regardless of the size of the scoliotic deformation angle and bone maturity.

C1 [Trzcinska, Sandra] Coll Rehabil Warsaw, Dept Physiotherapy, PL-01234 Warsaw, Poland.

[Koszela, Kamil] Natl Inst Geriatr Rheumatol & Rehabil, Neuroorthoped & Neurol Clin & Polyclin, PL-02637 Warsaw, Poland.

C3 National Institute of Geriatrics, Rheumatology & Rehabilitation

RP Koszela, K (通讯作者)， Natl Inst Geriatr Rheumatol & Rehabil, Neuroorthoped & Neurol Clin & Polyclin, PL-02637 Warsaw, Poland.

EM kamil.aikido@interia.pl

RI Koszela, Kamil/ABC-3000-2021; Trzcińska, Sandra Kamila/ADC-2552-2022;

Koszela, Kamil/IUM-9552-2023

OI Koszela, Kamil/0000-0003-3629-7523; Trzcińska, Sandra

Kamila/0000-0002-6085-3932; Koszela, Kamil/0000-0003-3629-7523

CR [Anonymous], 2007, ANN ACAD MED SILES

Cheung CWJ, 2015, IEEE T MED IMAGING, V34, P1760, DOI 10.1109/TMI.2015.2390233

Chowanska J, 2012, SCOLIOSIS SPINAL DIS, V7, DOI 10.1186/1748-7161-7-9

Goldberg C.J., 2001, SPINE, V26, P55, DOI [10.1097/00007632-200102150-00005, DOI 10.1097/00007632-200102150-00005]

Jiang WW, 2018, EUR SPINE J, V27, P2139, DOI 10.1007/s00586-018-5646-5

Kiebzak W., 2017, POL J PHYSIOTHER, V17, P140

Kotwicki T., 2008, FIZJOTERAPIA POLSKA, V8, P231

Lapuente JP, 2002, ST HEAL T, V88, P258

Latalski M, 2022, J CLIN MED, V11, DOI 10.3390/jcm11092669

Loughenbury PR, 2021, SPINE DEFORM, V9, P949, DOI 10.1007/s43390-021-00314-6

Nisser J, 2020, Z ORTHOP UNFALLCHIR, V158, P318, DOI 10.1055/a-0881-3430

Oakley PA, 2020, DOSE-RESPONSE, V18, DOI 10.1177/1559325820957797

Oakley PA, 2019, DOSE-RESPONSE, V17, DOI 10.1177/1559325819852810

Presciutti SM, 2014, SPINE J, V14, P1984, DOI 10.1016/j.spinee.2013.11.055

Ronckers CM, 2010, RADIAT RES, V174, P83, DOI 10.1667/RR2022.1

Sastre S, 1999, ST HEAL T, V59, P171

Sastre S., 2007, POL J PHYSIOTHER, V7, P223

Sastre S., BIOMECHANICS

Schmitz-Feuerhake I, 2011, RADIAT PROT DOSIM, V147, P310, DOI 10.1093/rpd/ncr348

Sliwinski Z, 2014, SCOLIOSIS SPINAL DIS, V9, P14

Trzcinska S., 2020, INT REV MED PRACT, V26, P42

Trzcinska S., 2017, POL J PHYSIOTHER, V17, P6

Trzcinska S, 2022, INT J ENV RES PUB HE, V19, DOI 10.3390/ijerph19084750

Trzcinska S, 2022, INT J ENV RES PUB HE, V19, DOI 10.3390/ijerph19010065

Trzcinska Sandra, 2020, Pol Merkur Lekarski, V48, P174

Zheng YP, 2016, SCOLIOSIS SPINAL DIS, V11, DOI 10.1186/s13013-016-0074-y

NR 26

TC 1

Z9 1

U1 2

U2 3

PU MDPI

PI BASEL

PA ST ALBAN-ANLAGE 66, CH-4052 BASEL, SWITZERLAND

EI 2227-9067

J9 CHILDREN-BASEL

JI Children-Basel

PD OCT

PY 2022

VL 9

IS 10

AR 1513

DI 10.3390/children9101513

PG 10

WC Pediatrics

WE Science Citation Index Expanded (SCI-EXPANDED)

SC Pediatrics

GA 5R0XO

UT WOS:000874243300001

PM 36291449

OA Green Published, gold

DA 2023-08-10

ER

PT J

AU Negrini, A

Poggio, M

Donzelli, S

Vanossi, M

Cordani, C

Romano, M

Negrini, S

AF Negrini, Alessandra

Poggio, Martina

Donzelli, Sabrina

Vanossi, Massimiliano

Cordani, Claudio

Romano, Michele

Negrini, Stefano

TI Sport improved medium-term results in a prospective cohort of 785 adolescents with idiopathic scoliosis braced full time. SOSORT 2018 award winner

SO EUROPEAN SPINE JOURNAL

LA English

DT Article

DE Sport; Idiopathic scoliosis; Full-time brace; Physical activity

ID EXERCISES; LIFE

AB Purpose The association between idiopathic scoliosis (IS) and sports activities remains vague. We aimed to analyse their effect on full-time braced adolescents with IS. Methods We retrospectively recruited all the consecutive patients of a tertiary referral Institute of age >= 10 (adolescents), with a juvenile (JIS) or adolescent (AIS) IS diagnosis, Risser 0-2, TLSO brace prescription and self-

reported adherence >= 20 h per day, and follow-up out-of-brace X-rays 18 months after brace prescription. We divided participants into two groups: SPORT (sport twice or more per week) and CONTROL (sport once per week or less). We calculated odds ratio (OR) to compare the outcome of subjects performing to those not performing sport. We ran a logistic regression with covariate adjustment to assess if sports frequency affected the outcomes. Results Out of 33,311 participants assessed for eligibility, 785 satisfied the inclusion criteria (693 females, age 12.7 +/- 1.3 and 40 +/- 11 degrees Cobb). The SPORT group consisted of 290 participants and the CONTROL group of 495. The SPORT group showed higher odds of improvement (OR = 1.59, 95%CI = 1.17-2.16, p = 0.0018). The odds of improving increased with the frequency of sports activity (OR = 1.20, 95%CI 1.08-1.34). Conclusion This study shows that sports activities increase the odds of improvement at 18-month follow-up in adolescents with IS treated with a full-time brace. The odds of improvement increase with sports week frequency.

C1 [Negrini, Alessandra; Poggio, Martina; Donzelli, Sabrina; Vanossi, Massimiliano; Romano, Michele] ISICO Italian Sci Spine Inst, Milan, Italy.

[Negrini, Stefano] Univ La Statale, Milan, Italy.

[Cordani, Claudio] IRCCS Ist Ortoped Galeazzi, Lab Evidence Based Rehabil, I-20161 Milan, Italy.

[Negrini, Stefano] IRCCS Ist Ortoped Galeazzi, Milan, Italy.

C3 IRCCS Istituto Ortopedico Galeazzi; IRCCS Istituto Ortopedico Galeazzi

RP Negrini, S (通讯作者)，Univ La Statale, Milan, Italy.; Negrini, S (通讯作者)，IRCCS Ist Ortoped Galeazzi, Milan, Italy.

EM Stefano.negrini@unimi.it

RI Cordani, Claudio/J-6590-2018; Negrini, Stefano/B-6667-2013; Donzelli,

Sabrina/IQU-0779-2023

OI Cordani, Claudio/0000-0002-9014-7887; Negrini,

Stefano/0000-0002-1878-2747;

FU Italian Ministry of Health - Ricerca Corrente 2022

FX This study was supported and funded by the Italian Ministry of Health -

Ricerca Corrente 2022. We thank Alberto Negrini and the ISICO software

team for the conception, development, and maintenance of the program for

data collection and extraction.

CR Atanasio S, 2008, DISABIL REHABIL-ASSI, V3, P154, DOI 10.1080/17483100801905843

BECKER TJ, 1986, CLIN SPORT MED, V5, P149

HELLSTROM M, 1990, ACTA RADIOL, V31, P127

Kenanidis EI, 2010, PHYSICIAN SPORTSMED, V38, P165, DOI 10.3810/psm.2010.06.1795

Longworth B, 2014, ARCH PHYS MED REHAB, V95, P1725, DOI 10.1016/j.apmr.2014.02.027

Monticone M, 2014, EUR SPINE J, V23, P1204, DOI 10.1007/s00586-014-3241-y

Negrini A, 2016, SCOLIOSIS SPINAL DIS, V11, DOI 10.1186/s13013-016-0100-0

Negrini S, 2019, ANN PHYS REHABIL MED, V62, P69, DOI 10.1016/j.rehab.2018.07.010

Negrini S, 2012, SCOLIOSIS SPINAL DIS, V7, DOI 10.1186/1748-7161-7-3

Negrini S, 2015, COCHRANE DB SYST REV, DOI 10.1002/14651858.CD006850.pub3

Negrini S, 2015, SCOLIOSIS SPINAL DIS, V10, DOI 10.1186/s13013-014-0025-4

Negrini S, 2014, BMC MUSCULOSKEL DIS, V15, DOI 10.1186/1471-2474-15-263

Negrini S, 2012, STUD HEALTH TECHNOL, V176, P437, DOI 10.3233/978-1-61499-067-3-437

Negrini S, 2011, SCOLIOSIS SPINAL DIS, V6, DOI 10.1186/1748-7161-6-8

Rivett L, 2009, BMC MUSCULOSKEL DIS, V10, DOI 10.1186/1471-2474-10-5

Romano M, 2015, SCOLIOSIS SPINAL DIS, V10, DOI 10.1186/s13013-014-0027-2

Romano M, 2013, SPINE, V38, pE883, DOI 10.1097/BRS.0b013e31829459f8

Tanchev PI, 2000, SPINE, V25, P1367, DOI 10.1097/00007632-200006010-00008

Tobias JH, 2019, INT J EPIDEMIOL, V48, P1152, DOI 10.1093/ije/dyy268

WARREN MP, 1986, NEW ENGL J MED, V314, P1348, DOI 10.1056/NEJM198605223142104

Watanabe K, 2017, J BONE JOINT SURG AM, V99, P284, DOI 10.2106/JBJS.16.00459

Weinstein SL, 2013, NEW ENGL J MED, V369, P1512, DOI 10.1056/NEJMoa1307337

Wu XY, 2017, PLOS ONE, V12, DOI 10.1371/journal.pone.0187668

Zaina F, 2015, J PEDIATR-US, V166, P163, DOI 10.1016/j.jpeds.2014.09.024

NR 24

TC 2

Z9 2

U1 2

U2 8

PU SPRINGER

PI NEW YORK

PA ONE NEW YORK PLAZA, SUITE 4600, NEW YORK, NY, UNITED STATES

SN 0940-6719

EI 1432-0932

J9 EUR SPINE J

JI Eur. Spine J.

PD NOV

PY 2022

VL 31

IS 11

BP 2994

EP 2999

DI 10.1007/s00586-022-07370-0

EA SEP 2022

PG 6

WC Clinical Neurology; Orthopedics

WE Science Citation Index Expanded (SCI-EXPANDED)

SC Neurosciences & Neurology; Orthopedics

GA 6N1PM

UT WOS:000852129300001

PM 36083351

OA Green Published, hybrid

DA 2023-08-10

ER

PT J

AU da Silveira, GE

Andrade, RM

Guilhermino, GG

Schmidt, AV

Neves, LM

Ribeiro, AP

AF da Silveira, Guilherme Erdmann

Andrade, Rodrigo Mantelatto

Guilhermino, Gean Gustavo

Schmidt, Ariane Verttu

Neves, Lucas Melo

Ribeiro, Ana Paula

TI The Effects of Short- and Long-Term Spinal Brace Use with and without

Exercise on Spine, Balance, and Gait in Adolescents with Idiopathic

Scoliosis

SO MEDICINA-LITHUANIA

LA English

DT Article

DE scoliosis; adolescents; brace; exercise; spine; gait; balance

ID PARAMETERS; ROTATION; CRITERIA; SERIES; SWAY

AB Background and Objectives: Adolescent idiopathic scoliosis (AIS) is a prevalent spinal disorder in adolescents. Previous studies have shown biomechanical changes of the gait in the lower limb of

AIS patients. To minimize the progression of scoliotic curvature, a spinal brace is used, which has been shown to be efficient. Usually, a brace is worn strictly for 20-22 h every day. To our knowledge, no study has assessed the short- and long-term effects of spinal brace use with or without an exercise program (6 months) to improve clinical and biomechanical parameters. The aim of our study was to verify the effects of short- and long-term spinal brace use, with or without an exercise program on the spine, body balance, and plantar load distribution during gait in AIS. Materials and Methods: A prospective randomized study was conducted with intention-to-treat analysis in forty-five adolescents diagnosed with AIS undergoing conservative treatment at a center specialized in spinal rehabilitation. Adolescents were evaluated at two stages of intervention: (1) spinal orthopedic brace, with acute use 24 h/day (n = 22) and (2) spinal orthopedic brace, with acute use between 15-18 h/day associated with a specific rehabilitation exercise protocol for six consecutive months (six months and 12 total sessions, n = 23). The evaluated parameters were: spine pain, using a visual analog scale (VAS); Cobb angle measurement using radiograph exams, as well as the Risser sign; and static balance and plantar pressure of the feet during gait, carried out using a pressure platform. Results: AIS patients showed significant improvements in the main scoliotic curvature, with a 12-degree reduction in Cobb angle pre- and post-short-term immediate use of spinal brace and a 5.3 degree correction after six months of spinal brace use in combination with specific exercises (long term). In addition, short- and long-term brace use with an exercise program showed a significant increase in anteroposterior and mediolateral balance and a reduction in plantar overload on the heel during gait, with an effect size between moderate and high. Conclusions: Intervention via the short- or long-term use of a spinal brace combined with specific exercises in adolescents with idiopathic scoliosis proved to be effective for correcting scoliotic curvature. In addition, intervention also showed improvements to the antero-posterior and mediolateral body balance and a reduction in the plantar load on the rearfoot region during gait, demonstrating effective mechanical action on the spine.

C1 [da Silveira, Guilherme Erdmann; Schmidt, Ariane Verttu; Neves, Lucas Melo; Ribeiro, Ana Paula] Univ Santo Amaro, Med Sch, Hlth Sci Postgrad Dept, Biomech & Musculoskeletal Rehabil Lab, BR-04829300 Sao Paulo, SP, Brazil.

[Andrade, Rodrigo Mantelatto; Guilhermino, Gean Gustavo] Rehabilitation Clin, Spine Grp, BR-13025270 Sao Paulo, SP, Brazil.

[Neves, Lucas Melo] Univ Sao Paulo, Dept Psychiat, BR-01246903 Sao Paulo, SP, Brazil.

[Ribeiro, Ana Paula] Univ Sao Paulo, Sch Med, Phys Therapy Dept, BR-01246903 Sao Paulo, SP, Brazil.

C3 Universidade de Santo Amaro (UNISA); Universidade de Sao Paulo;

Universidade de Sao Paulo

RP Ribeiro, AP (通讯作者)，Univ Santo Amaro, Med Sch, Hlth Sci Postgrad Dept, Biomech & Musculoskeletal Rehabil Lab, BR-04829300 Sao Paulo, SP, Brazil.; Ribeiro, AP (通讯作者)，Univ Sao Paulo, Sch Med, Phys Therapy Dept, BR-01246903 Sao Paulo, SP, Brazil.

EM gesilveira@prof.unisa.br; rodrigoandrade@usp.br;

gguilhermino99@gmail.com; ariane.schmidt@hotmail.com;

lmneves@prof.unisa.br; apribeiro@alumni.usp.br

RI Neves, Lucas Melo/K-6050-2016; Ribeiro, Ana Paula/J-6368-2013

OI Neves, Lucas Melo/0000-0003-2426-9736; Ribeiro, Ana

Paula/0000-0002-1061-3789

FU spine group of the Scientific Institute Specialized in Rehabilitation of

the city of Campinas, Sao Paulo/SP, Brazil

FX The authors acknowledge the help and support of the all the participants

and the spine group of the Scientific Institute Specialized in

Rehabilitation of the city of Campinas, Sao Paulo/SP, Brazil, during

this study.

CR Almansour H, 2019, J CLIN MED, V8, DOI 10.3390/jcm8111804

Aulisa AG, 2015, BMC MUSCULOSKEL DIS, V16, DOI 10.1186/s12891-015-0782-0

Aulisa AG, 2014, SCOLIOSIS SPINAL DIS, V9, DOI 10.1186/1748-7161-9-3

Aulisa AG, 2012, SPINE, V37, pE786, DOI 10.1097/BRS.0b013e31824b547d

Beaulieu M, 2009, EUR SPINE J, V18, P38, DOI 10.1007/s00586-008-0831-6

Belabbassi H., 2013, SCOLIOSIS SPINAL DIS, V8, pO6, DOI [10.1186/1748-7161-8-S2-O6, DOI 10.1186/1748-7161-8-S2-O6]

Bialek M, 2011, SCOLIOSIS SPINAL DIS, V6, DOI 10.1186/1748-7161-6-25

Bruyneel A. -V., 2010, Annals of Physical and Rehabilitation Medicine, V53, P372, DOI 10.1016/j.rehab.2010.06.005

Burger M, 2019, SOUTH AFR J PHYSIOTH, V75, DOI 10.4102/sajp.v75i1.904

Catan L, 2020, INT J ENV RES PUB HE, V17, DOI 10.3390/ijerph17062167

Charles YP, 2017, J PEDIATR ORTHOP B, V26, P240, DOI 10.1097/BPB.0000000000000410

Clin J, 2010, SPINE, V35, P1706, DOI 10.1097/BRS.0b013e3181cb46f6

Coillard C, 2007, J PEDIATR ORTHOPED, V27, P375, DOI 10.1097/01.bpb.0000271330.64234.db

Daryabor A, 2017, ANN PHYS REHABIL MED, V60, P107, DOI 10.1016/j.rehab.2016.10.008

Day Joseph M, 2019, Arch Physiother, V9, P8, DOI 10.1186/s40945-019-0060-9

de Mauroy JC, 2014, SCOLIOSIS SPINAL DIS, V9, DOI 10.1186/1748-7161-9-19

Alves VLD, 2009, SPINE, V34, pE926, DOI 10.1097/BRS.0b013e3181afd1b2

El Hawary R, 2019, SPINE J, V19, P1917, DOI 10.1016/j.spinee.2019.07.008

Freidel K, 2002, SPINE, V27, pE87, DOI 10.1097/00007632-200202150-00013

Gammon SR, 2010, J PEDIATR ORTHOPED, V30, P531, DOI 10.1097/BPO.0b013e3181e4f761

Gauchard GC, 2001, SPINE, V26, P1052, DOI 10.1097/00007632-200105010-00014

Giakas G, 1996, SPINE, V21, P2235, DOI 10.1097/00007632-199610010-00011

Barsotti CEG, 2021, J BACK MUSCULOSKELET, V34, P821, DOI 10.3233/BMR-200320

Haber Cassandra Kay, 2015, Arch Physiother, V5, P4, DOI 10.1186/s40945-015-0001-1

Kaelin AJ, 2020, ANN TRANSL MED, V8, DOI 10.21037/atm.2019.09.69

Kalichman L, 2016, J BODYW MOV THER, V20, P56, DOI 10.1016/j.jbmt.2015.04.007

Karavidas N, 2019, ADOLESC HEALTH MED T, V10, P153, DOI 10.2147/AHMT.S190565

Kim DS, 2020, J CLIN NEUROSCI, V81, P196, DOI 10.1016/j.jocn.2020.09.035

Kramers-de Quervain IA, 2004, EUR SPINE J, V13, P449, DOI 10.1007/s00586-003-0588-x

Lee CF, 2010, SPINE, V35, pE1492, DOI 10.1097/BRS.0b013e3181ecf3fe

Lee SW, 2014, GAIT POSTURE, V40, P676, DOI 10.1016/j.gaitpost.2014.07.023

Ma QC, 2020, FRONT PEDIATR, V7, DOI 10.3389/fped.2019.00523

Mahaudens Philippe, 2005, Spine J, V5, P427

Mccoy CE, 2017, WEST J EMERG MED, V18, P1075, DOI 10.5811/westjem.2017.8.35985

Meng ZD, 2017, MEDICINE, V96, DOI 10.1097/MD.0000000000006828

Minsk MK, 2017, SCOLIOSIS SPINAL DIS, V12, DOI 10.1186/s13013-017-0117-z

NACHEMSON AL, 1995, J BONE JOINT SURG AM, V77A, P815, DOI 10.2106/00004623-199506000-00001

Nault ML, 2002, SPINE, V27, P1911, DOI 10.1097/00007632-200209010-00018

Negrini S, 2007, Eura Medicophys, V43, P171

Negrini S, 2015, COCHRANE DB SYST REV, DOI 10.1002/14651858.CD006850.pub3

Negrini S, 2014, BMC MUSCULOSKEL DIS, V15, DOI 10.1186/1471-2474-15-263

Nishida M, 2017, GAIT POSTURE, V51, P142, DOI 10.1016/j.gaitpost.2016.10.004

Paolucci T, 2013, EUR J PHYS REHAB MED, V49, P649

Park HJ, 2016, EUR SPINE J, V25, P385, DOI 10.1007/s00586-015-3931-0

Pau M, 2018, GAIT POSTURE, V61, P301, DOI 10.1016/j.gaitpost.2018.01.031

Penha PJ, 2018, SPINE, V43, P1710, DOI 10.1097/BRS.0000000000002725

Ribeiro AP, 2015, PLOS ONE, V10, DOI 10.1371/journal.pone.0136971

Samadi B, 2020, COMPUT METHOD BIOMEC, V23, P664, DOI 10.1080/10255842.2020.1758075

Smania N, 2008, DISABIL REHABIL, V30, P763, DOI 10.1080/17483100801921311

Thompson RM, 2017, J BONE JOINT SURG AM, V99, P923, DOI 10.2106/JBJS.16.01050

Weinstein SL, 2013, NEW ENGL J MED, V369, P1512, DOI 10.1056/NEJMoa1307337

Weiss HR, 2021, SOUTH AFR J PHYSIOTH, V77, DOI 10.4102/sajp.v77i2.1573

Weiss Hans-Rudolf, 2003, Pediatr Rehabil, V6, P183

Weiss HR, 2003, ORTHOPADE, V32, P146, DOI 10.1007/s00132-002-0430-x

Wu KW, 2019, GAIT POSTURE, V68, P423, DOI 10.1016/j.gaitpost.2018.12.024

Yang JH, 2013, EUR SPINE J, V22, P2407, DOI 10.1007/s00586-013-2845-y

Yilmaz H, 2020, SPINE J, V20, P947, DOI 10.1016/j.spinee.2020.01.008

Zaborowska-Sapeta K, 2011, SCOLIOSIS SPINAL DIS, V6, DOI 10.1186/1748-7161-6-2

Zaina F, 2015, SCOLIOSIS SPINAL DIS, V10, DOI 10.1186/s13013-015-0046-7

Zhang Z, 2017, MEDICINE, V96, DOI 10.1097/MD.0000000000008799

Zhao J, 2018, MEDICINE, V97, DOI 10.1097/MD.0000000000011216

Zhu ZZ, 2015, ANN TRANSL MED, V3, DOI 10.3978/j.issn.2305-5839.2015.02.04

NR 62

TC 0

Z9 1

U1 10

U2 17

PU MDPI

PI BASEL

PA ST ALBAN-ANLAGE 66, CH-4052 BASEL, SWITZERLAND

SN 1010-660X

EI 1648-9144

J9 MEDICINA-LITHUANIA

JI Med. Lith.

PD AUG

PY 2022

VL 58

IS 8

AR 1024

DI 10.3390/medicina58081024

PG 14

WC Medicine, General & Internal

WE Science Citation Index Expanded (SCI-EXPANDED)

SC General & Internal Medicine

GA 4C8YY

UT WOS:000846732500001

PM 36013490

OA Green Published, gold

DA 2023-08-10

ER

PT J

AU Yildirim, S

Ozyilmaz, S

Elmadag, NM

Yabaci, A

AF Yildirim, Sefa

Ozyilmaz, Semiramis

Elmadag, Nuh Mehmet

Yabaci, Aysegul

TI Effects of Core Stabilization Exercises on Pulmonary Function,

Respiratory Muscle Strength, Peripheral Muscle Strength, Functional

Capacity, and Perceived Appearance in Children With Adolescent

Idiopathic Scoliosis A Randomized Controlled Trial

SO AMERICAN JOURNAL OF PHYSICAL MEDICINE & REHABILITATION

LA English

DT Article

DE Adolescent Idiopathic Scoliosis; Core Stabilization Exercises;

Respiratory Muscle Strength; Perceived Appearance

ID COBB ANGLE; DEFORMITY; VALIDITY; PROGRAM; SPINE

AB Objective: The aim of the study was to investigate the effects of core stabilization exercises on pulmonary function, respiratory muscle strength, peripheral muscle strength, walking capacity, and perceived appearance in children with adolescent idiopathic scoliosis.

Design: This is an evaluator-blinded, randomized controlled trial. A total of 30 patients were randomly allocated to either a training group (n = 15, age = 13.8 +/- 2.8 yrs, Cobb angle = 19.3 +/- 6 degrees, Risser sign: n((1)) = 1; n((2-4)) = 14) or a control group (n = 15, age = 15.8 +/- 3.4 yrs, Cobb angle = 20.8 +/- 7.9 degrees, Risser sign: n((1)) = 1; n((2-4)) = 14). The training group received

core stabilization exercises in addition to the traditional scoliosis exercises for 8 wks. The control group only received the traditional scoliosis exercises for 8 wks. Spirometry, maximal inspiratory and expiratory pressures, 6-min walking test, peripheral muscle strength, and the Walter Reed Visual Assessment Scale were assessed at the beginning and end of the study.

Results: The training group showed statistically significant improvements in maximal inspiratory pressure, maximum expiratory pressure, and Walter Reed Visual Assessment Scale score (respectively, mean changes = 17.4 +/- 5.2 cmH(2)O; 10.6 +/- 4.3 cmH(2)O; 2.4 +/- 1.6), which were significantly larger compared with the control group (P < 0.05).

Conclusions: Core stabilization exercises given in addition to the traditional scoliosis exercise can improve respiratory muscle strength and perceived appearance in patients with adolescent idiopathic scoliosis.

C1 [Yildirim, Sefa; Ozyilmaz, Semiramis] Bezmialem Vakif Univ, Dept Physiotherapy & Rehabil, Fac Hlth Sci, Silahtaraga St, TR-34050 Istanbul, Turkey.

[Yildirim, Sefa] Bezmialem Vakif Univ, Dept Physiotherapy & Rehabil, Inst Hlth Sci, Istanbul, Turkey.

[Elmadag, Nuh Mehmet] Bezmialem Vakif Univ, Dept Orthoped & Traumatol, Fac Med, Istanbul, Turkey.

[Yabaci, Aysegul] Bezmialem Vakif Univ, Dept Biostat, Fac Med, Istanbul, Turkey.

C3 Bezmialem Vakif University; Bezmialem Vakif University; Bezmialem Vakif

University; Bezmialem Vakif University

RP Yildirim, S (通讯作者)， Bezmialem Vakif Univ, Dept Physiotherapy & Rehabil, Fac Hlth Sci, Silahtaraga St, TR-34050 Istanbul, Turkey.

RI Ozyilmaz, Semiramis/AAD-4136-2020

OI Ozyilmaz, Semiramis/0000-0002-7788-9739

CR Aldrich TK, 2002, AM J RESP CRIT CARE, V166, P548, DOI 10.1164/rccm.166.4.518

Amaricai E, 2019, J INT MED RES, DOI 10.1177/0300060519895093

AMENDT LE, 1990, PHYS THER, V70, P108, DOI 10.1093/ptj/70.2.108

Bartels B, 2013, PHYS THER, V93, P529, DOI 10.2522/ptj.20120210

Beenakker EAC, 2001, NEUROMUSCULAR DISORD, V11, P441, DOI 10.1016/S0960-8966(01)00193-6

Chtara M, 2005, BRIT J SPORT MED, V39, P555, DOI 10.1136/bjsm.2004.015248

COOPER DM, 1984, AM REV RESPIR DIS, V130, P16

Crapo RO, 2002, AM J RESP CRIT CARE, V166, P111, DOI 10.1164/rccm.166/1/111

Alves VLD, 2006, CHEST, V130, P500, DOI 10.1378/chest.130.2.500

Alves VLD, 2016, ACTA ORTOP BRAS, V24, P296, DOI 10.1590/1413-785220162406120752

Fredericson Michael, 2005, Phys Med Rehabil Clin N Am, V16, P669, DOI 10.1016/j.pmr.2005.03.001

Greiner KA, 2002, AM FAM PHYSICIAN, V65, P1817

Hulzebos E, 2018, RESPIRATION, V95, DOI 10.1159/000485464

Ko KJ, 2017, J EXERC REHABIL, V13, P244, DOI 10.12965/jer.1734952.476

Koumbourlis AC, 2006, PAEDIATR RESPIR REV, V7, P152, DOI 10.1016/j.prrv.2006.04.009

Kuo FC, 2011, SPINE, V36, P810, DOI 10.1097/BRS.0b013e3181d0f80c

Lim SH, 2019, INT J ENV RES PUB HE, V16, DOI 10.3390/ijerph16122235

Marco E., 2012, SCOLIOSIS SPINAL DIS, V7, P1

Martinez-Llorens J, 2010, EUR RESPIR J, V36, P393, DOI 10.1183/09031936.00025509

Miller MR, 2005, EUR RESPIR J, V26, P319, DOI 10.1183/09031936.05.00034805

Negrini S, 2012, SCOLIOSIS SPINAL DIS, V7, DOI 10.1186/1748-7161-7-3

Park YH, 2016, J INT MED RES, V44, P728, DOI 10.1177/0300060516639750

Pereira Carlos Alberto de Castro, 2007, J. bras. pneumol., V33, P397, DOI 10.1590/S1806-37132007000400008

Pineda S, 2006, SCOLIOSIS SPINAL DIS, V1, DOI 10.1186/1748-7161-1-18

Schmidt R T, 1970, Arch Phys Med Rehabil, V51, P321

Shin SS, 2012, J PHYS THER SCI, V24, P211

Szopa A, 2017, MEDICINE, V96, DOI 10.1097/MD.0000000000007032

Tsiligiannis T, 2012, SCOLIOSIS SPINAL DIS, V7, DOI 10.1186/1748-7161-7-7

Wells C, 2012, COMPLEMENT THER MED, V20, P253, DOI 10.1016/j.ctim.2012.02.005

Yagci G, 2019, PROSTHET ORTHOT INT, V43, P301, DOI 10.1177/0309364618820144

NR 30

TC 0

Z9 0

U1 15

U2 27

PU LIPPINCOTT WILLIAMS & WILKINS

PI PHILADELPHIA

PA TWO COMMERCE SQ, 2001 MARKET ST, PHILADELPHIA, PA 19103 USA

SN 0894-9115

EI 1537-7385

J9 AM J PHYS MED REHAB

JI Am. J. Phys. Med. Rehabil.

PD AUG

PY 2022

VL 101

IS 8

BP 719

EP 725

DI 10.1097/PHM.0000000000001984

PG 7

WC Rehabilitation; Sport Sciences

WE Science Citation Index Expanded (SCI-EXPANDED)

SC Rehabilitation; Sport Sciences

GA 3B4CY

UT WOS:000827891800006

PM 35859288

DA 2023-08-10

ER

PT J

AU Qi, KX

Fu, HD

Yang, Z

Bao, LQ

Shao, YX

AF Qi, Kexin

Fu, Haidong

Yang, Zhen

Bao, Lingqi

Shao, Yinxin

TI Effects of Core Stabilization Training on the Cobb Angle and Pulmonary

Function in Adolescent Patients with Idiopathic Scoliosis

SO JOURNAL OF ENVIRONMENTAL AND PUBLIC HEALTH

LA English

DT Article

ID MUSCLE STRENGTH; EXERCISES; CAPACITY

AB Objective. To observe the effects of core stabilization training on the Cobb angle, respiratory muscle strength (maximum inspiratory pressure, MIP; maximal expiratory pressure, MEP), and pulmonary function (forced vital capacity, FVC; forced expiratory volume, FEV1.0; FEV1.0/FVC%) in adolescent patients with idiopathic scoliosis (AIS) and offer practical-based evidence for the rehabilitation treatment for AIS patients. Methods. 36 AIS patients were assigned to the core stability training (CST) group (n = 18) and control group (n = 18); the CST group participated in three sessions of core stabilization exercise per week for 12 weeks and the control group did not perform regular physical training during 12 weeks of study. Then, the Cobb angle, respiratory muscle strength (MIP and MEP), and pulmonary function (FVC, FEV1.0, and FEV1.0/FVC%) were measured before and after core stabilization training. Results. After 12 weeks of core stabilization training, compared with the pretest, the Cobb angle showed a significant decrease, FVC, FEV1, MIP, and MEP a significant increase ($P < 0.01$ respectively), and there was no statistical difference in FEV1/FVC in

the CST group; there was no significant difference (P > 0.05 respectively) before and after an experiment in the control group except MEP decreased significantly (P < 0.01, P < 0.05). After 12 weeks of core stabilization training, compared with the control group, the Cobb angle significantly decreased (P < 0.01), FVC, FEV1, MIP, and MEP significantly increased (P < 0.05 respectively) in the CST group, but there was no significant difference (P > 0.05, respectively) in FEV1/FVC between the control group and CST group. Conclusions. Core stabilization exercise can be considered to have a positive effect on the normal physiological curvature of the spine in AIS patients, as it decreases the Cobb angle and strengthens respiratory muscle strength and pulmonary function.

C1 [Qi, Kexin] Changchun Univ, Dept Phys Educ & Res, Changchun 130000, china.

[Fu, Haidong] Changchun Univ, Acad Affairs Off, Changchun 130000, china.

[Yang, Zhen] Luoyang Inst Sci & Technol, Luoyang 471023, Henan, china.

[Bao, Lingqi; Shao, Yinxin] Changchun Univ, Grad Sch, Changchun 130000, china.

C3 Changchun University; Changchun University; Luoyang Institute of Science & Technology; Changchun University

RP Yang, Z (通讯作者)，Luoyang Inst Sci & Technol, Luoyang 471023, Henan, china.

EM qikx@ccu.edu.cn; fuhd.ccu@163.com; yz@lit.edu.cn; sdtablq123@126.com; shoayixin@126.com

FU Scientific Research Topic of Education Department of Liaoning Province [WJC201913]

FX The study was supported by the Scientific Research Topic of Education Department of Liaoning Province in 2019, mechanism research on the deep integration of national fitness and national health (WJC201913).

CR Abdelaal AAM, 2018, J INT MED RES, V46, P381, DOI 10.1177/0300060517715375

Akodu A., 2016, PHYSIOTHERAPY, V102, pe243, DOI [10.1016/j.physio.2016.10.304, DOI 10.1016/J.PHYSIO.2016.10.304]

Amaricai E, 2019, J INT MED RES, DOI 10.1177/0300060519895093

Bettany-Saltikov J, 2014, EUR J PHYS REHAB MED, V50, P111

Bialek M, 2011, SCOLIOSIS SPINAL DIS, V6, DOI 10.1186/1748-7161-6-25

Fabian Krzysztof Marek, 2010, Ortop Traumatol Rehabil, V12, P301

Farrell J, 2021, EUR SPINE J, V30, P634, DOI 10.1007/s00586-020-06552-y

Horne JP, 2014, AM FAM PHYSICIAN, V89, P193

Jin Youngwan, 2014, [Korean society for Wellness, 한국웰니스학회], V9, P185

Johari J, 2016, SINGAP MED J, V57, P33, DOI 10.11622/smedj.2016009

Kato S, 2019, GLOB SPINE J, V9, P866, DOI 10.1177/2192568218811312

Ko KJ, 2017, J EXERC REHABIL, V13, P244, DOI 10.12965/jer.1734952.476

Konieczny MR, 2013, J CHILD ORTHOP, V7, P3, DOI 10.1007/s11832-012-0457-4

Misterska E, 2018, PLOS ONE, V13, DOI 10.1371/journal.pone.0193447

Mustafaoglu R, 2019, PEDIATR PULM, V54, P1002, DOI 10.1002/ppul.24330

Noh DK, 2014, J BACK MUSCULOSKELET, V27, P331, DOI 10.3233/BMR-130452

Park JH, 2018, EUR J PHYS REHAB MED, V54, P440, DOI 10.23736/S1973-9087.17.04461-6

Park Shin-Jun, 2017, J Phys Ther Sci, V29, P1144, DOI 10.1589/jpts.29.1144

Park YH, 2016, J INT MED RES, V44, P728, DOI 10.1177/0300060516639750

Ran H.A.O, 2011, PULMONARY FUNCTION P

Strongoli LM, 2010, J SPORT SCI MED, V9, P270

Villamor GA, 2019, SPINE DEFORM, V7, P729, DOI 10.1016/j.jspd.2019.02.007

Vitale MG, 2008, SPINE, V33, P1242, DOI 10.1097/BRS.0b013e3181714536

Wang X, 2018, INTERVENTION CORRECT

Wick Jane Maureen, 2009, AORN J, V90, P347

Yong Q.I.U, 2009, CHINESE J SPINE SPIN, V19, P174

NR 26

TC 2

Z9 2

U1 10

U2 25

PU HINDAWI LTD

PI LONDON

PA ADAM HOUSE, 3RD FLR, 1 FITZROY SQ, LONDON, W1T 5HF, united kingdom

SN 1687-9805

EI 1687-9813

J9 J ENVIRON PUBLIC HEA

JI J. Environ. Public Health

PD JUL 31

PY 2022

VL 2022

AR 4263393

DI 10.1155/2022/4263393

PG 6

WC Public, Environmental & Occupational Health

WE Science Citation Index Expanded (SCI-EXPANDED); Social Science Citation Index (SSCI)

SC Public, Environmental & Occupational Health

GA 3Q1SR

UT WOS:000838014600003

PM 35958375

OA Green Published, gold

DA 2023-08-10

ER

PT J

AU Fan, YL

To, MKT

Kuang, GM

Cheung, JPY

AF Fan, Yunli

To, Michael K. T.

Kuang, Guan-Ming

Cheung, Jason Pui Yin

TI The Relationship Between Compliance of Physiotherapeutic Scoliosis

Specific Exercises and Curve Regression With Mild to Moderate Adolescent

Idiopathic Scoliosis

SO GLOBAL SPINE JOURNAL

LA English

DT Article; Early Access

DE adolescent idiopathic scoliosis; physiotherapeutic scoliosis specific

exercise; exercise compliance; curve regression; cobb angle; apical

translation; apical wedging; apical rotation

ID QUALITY-OF-LIFE; SCHROTH EXERCISES; PROGRESSION; THERAPY

AB Study Design: Retrospective Case-control Study.

Objectives: To determine the requisite exercise compliance (EC) of physiotherapeutic scoliosis-specific exercise (PSSE) for achieving curve regression; to analyze whether the apical translation (AT), apical wedging (AW), and apical rotation (AR) of the major curve improve with regression effect.

Methods: Between 2019 and 2021, a total of 763 patients undertook a 6-month PSSE treatment. This resulted 426 compliable and 302 uncompliable patients remained available for analysis. For compliable patients, 213 with curve regression and 213 age-/sex-matched with curve stabilization/deterioration at the 6-month, were eligible for regression analysis to detect the relationship between EC and regression effect at the 6-month; receiver operating characteristic (ROC) curve analysis and Youden's index were applied to identify the threshold of EC leading to curve regression at the 6-month. The AT, AW, and AR of the major curve were compared before and after 6-month PSSE to investigate the radiographic parameters that improved with regression effect.

Results: EC was correlated with regression effect (odds ratio: 19.9, 95% confidence interval: 11.3-35.0, P < .001) and the cutoff threshold of EC was 4.4 h/week for 6 months to realize such an effect.

AT was improved by 47.6% with curve regression, in which 152 cases remained curve regression and no case progressed into the operative threshold at the 1.5- to 2-year.

Conclusions: A 6-month PSSE protocol of 4.4 hours per week was potentially leading to curve regression in treating mild to moderate scoliosis. An improvement in AT of the major curve was observed with the regression effect.

C1 [Fan, Yunli; To, Michael K. T.; Kuang, Guan-Ming; Cheung, Jason Pui Yin] Univ Hong Kong, Dept Orthopaed, Shenzhen Hosp, Shenzhen, china.

[Fan, Yunli; To, Michael K. T.; Cheung, Jason Pui Yin] Univ Hong Kong, Dept Orthopaed & Traumatol, Hong Kong, china.

[Fan, Yunli] Univ Hong Kong, Dept Physiotherapy, Shenzhen Hosp, Shenzhen, china.

C3 University of Hong Kong; University of Hong Kong; University of Hong

Kong

RP Cheung, JPY (通讯作者)， Univ Hong Kong, Dept Orthopaed & Traumatol, Hong Kong, china.

EM cheungjp@hku.hk

OI Fan, Yunli/0000-0001-9936-8857; Kuang, Guan-Ming/0000-0003-4976-4151

FU high level - hospital program of HKU - SZH in Shenzhen, Guangdong

Province, China [HKUSZH201902042]; Hong Kong Research Grant Council

Research Impact Fund [R5017-18]

FX The author(s) disclosed receipt of the following financial support for

the research, authorship, and/or publication of this article: This study

was financially supported by the high level - hospital program

(HKUSZH201902042) of the HKU - SZH in Shenzhen, Guangdong Province,

China. This study was partially supported by Hong Kong Research Grant

Council Research Impact Fund (R5017-18).

CR Berdishevsky H, 2016, SCOLIOSIS SPINAL DIS, V11, DOI 10.1186/s13013-016-0076-9

Cheung JPY, 2020, CLIN ORTHOP RELAT R, V478, P334, DOI 10.1097/CORR.0000000000000989

Cheung JPY, 2018, CLIN ORTHOP RELAT R, V476, P429, DOI 10.1007/s11999.0000000000000027

Czaprowski D, 2011, SCOLIOSIS SPINAL DIS, V6, DOI 10.1186/1748-7161-6-22

Day Joseph M, 2019, Arch Physiother, V9, P8, DOI 10.1186/s40945-019-0060-9

Fan YL, 2021, PLOS ONE, V16, DOI 10.1371/journal.pone.0245829

Fan YL, 2020, BMC MUSCULOSKEL DIS, V21, DOI 10.1186/s12891-020-03517-6

Gao A, 2021, CHINESE MED J-PEKING, V134, P2589, DOI 10.1097/CM9.0000000000001799

Kim S, 2017, STAT METHODS MED RES, V26, P1237, DOI 10.1177/0962280215572407

Korbel Krzysztof, 2014, Pol Orthop Traumatol, V79, P118

Kuru T, 2016, CLIN REHABIL, V30, P181, DOI 10.1177/0269215515575745

Kwan KYH, 2017, SCOLIOSIS SPINAL DIS, V12, DOI 10.1186/s13013-017-0139-6

La Maida GA, 2018, EUR SPINE J, V27, P157, DOI 10.1007/s00586-018-5626-9

Lehnert-Schroth C, 1979, ZFA (Stuttgart), V55, P1969

Liu DL, 2020, SPINE, V45, P1039, DOI 10.1097/BRS.0000000000003451

LONSTEIN JE, 1984, J BONE JOINT SURG AM, V66A, P1061, DOI 10.2106/00004623-198466070-00013

Malfair D, 2010, AM J ROENTGENOL, V194, pS8, DOI 10.2214/AJR.07.7145

Monticone M, 2014, EUR SPINE J, V23, P1204, DOI 10.1007/s00586-014-3241-y

NASH CL, 1969, J BONE JOINT SURG AM, VA 51, P223, DOI 10.2106/00004623-196951020-00002

Negrini S, 2018, SCOLIOSIS SPINAL DIS, V13, DOI 10.1186/s13013-017-0145-8

Negrini S, 2009, SCOLIOSIS SPINAL DIS, V4, DOI 10.1186/1748-7161-4-19

Noh DK, 2014, J BACK MUSCULOSKELET, V27, P331, DOI 10.3233/BMR-130452

Schreiber S, 2019, BMC MUSCULOSKEL DIS, V20, DOI 10.1186/s12891-019-2695-9

Schreiber S, 2016, PLOS ONE, V11, DOI 10.1371/journal.pone.0168746

Smits-Engelsman B, 2011, J PEDIATR-US, V158, P130, DOI 10.1016/j.jpeds.2010.07.021

Vandal S, 1999, Issues Compr Pediatr Nurs, V22, P59

Weinstein SL, 2008, LANCET, V371, P1527, DOI 10.1016/S0140-6736(08)60658-3

Weinstein SL, 2019, J PEDIATR ORTHOPED, V39, pS44, DOI 10.1097/BPO.0000000000001350

Weinstein SL, 2013, NEW ENGL J MED, V369, P1512, DOI 10.1056/NEJMoa1307337

Yagci G, 2019, PROSTHET ORTHOT INT, V43, P301, DOI 10.1177/0309364618820144

Yagci G, 2018, J BACK MUSCULOSKELET, V31, P693, DOI 10.3233/BMR-170868

Zapata KA, 2019, PEDIATR PHYS THER, V31, P280, DOI 10.1097/PEP.0000000000000621

Zheng Y, 2018, SPINE, V43, pE494, DOI 10.1097/BRS.0000000000002412

NR 33

TC 0

Z9 0

U1 2

U2 7

PU SAGE PUBLICATIONS LTD

PI LONDON

PA 1 OLIVERS YARD, 55 CITY ROAD, LONDON EC1Y 1SP, united kingdom

SN 2192-5682

EI 2192-5690

J9 GLOB SPINE J

JI Glob. Spine J.

PD 2022 JUN 28

PY 2022

DI 10.1177/21925682221109565

EA JUN 2022

PG 11

WC Clinical Neurology; Orthopedics

WE Science Citation Index Expanded (SCI-EXPANDED)

SC Neurosciences & Neurology; Orthopedics

GA 2T0MA

UT WOS:000822175900001

PM 35762385

OA gold

DA 2023-08-10

ER

PT J

AU Doucet, C

Rousseau, P

Page, I

AF Doucet, Chantal

Rousseau, Philippe

Page, Isabelle

TI Level of knowledge on conservative management of adolescent idiopathic

scoliosis among undergraduate students in healthcare: A scoping review

SO MUSCULOSKELETAL SCIENCE AND PRACTICE

LA English

DT Review

DE Adolescent idiopathic scoliosis; Knowledge; Education; Health

profession; Undergraduate students; Questionnaire

AB Background: Adolescent Idiopathic Scoliosis (AIS) is a three-dimensional deformity of the spine associated with pain and aesthetic changes. Various health care professionals may be led to evaluate and/or manage adolescents presenting AIS. There is no compiling of the studies evaluating the level of knowledge on AIS conservative management among undergraduate students in healthcare. Objective: To identify and map the current studies evaluating the level of knowledge of health profession students on AIS conservative management. Design: Scoping review. Methods: The search strategy was conducted in Scopus database. Three concepts were included: "Adolescent idiopathic scoliosis", "Knowledge" and "Undergraduate students studying in a healthcare field". Studies

identification included (1) duplicates removing, (2) title and abstract screening, and (3) full-text screening. The quality of the included studies was assessed. Studies' characteristics were extracted, and results were summarized. Results: Searches yielded the identification of 245 citations. After duplicates removal and abstract screening, three full-text articles were identified. Following full-text review, two full-text articles were finally included. Both studies evaluated students in physiotherapy, but using distinct questionnaires. Both studies reported an unsatisfactory level of knowledge. Conclusion: Knowledge of future health professionals about the conservative management of AIS has been barely evaluated. Therefore, no conclusion can be drawn regarding the level of knowledge of undergraduate health professions' students on AIS conservative management. The development a standardized questionnaire to adequately assess this knowledge across institutions and professions is required.

C1 [Doucet, Chantal; Rousseau, Philippe; Page, Isabelle] Univ Quebeca Trois Rivieres, Dept Chiropract, 3351 Boul Forges, Trois Rivieres, PQ G8Z 4M3, Canada.

[Page, Isabelle] Ctr Interdisciplinary Res Rehabil & Social Integra, Ctr Integre Univ St eet Serv Sociaux Capitale Natl, 525 Boul Wilfrid Hamel, Quebec City, PQ G1M 2S8, Canada.

RP Page, I (通讯作者)， Univ Quebeca Trois Rivieres, Dept Chiropract, 3351 Boul Forges, Trois Rivieres, PQ G8Z 4M3, Canada.

EM Chantale.Doucet@uqtr.ca; Philippe.Rousseau2@uqtr.ca;

Isabelle.Page1@uqtr.ca

OI Page, Isabelle/0000-0002-7175-0402

FU Fonds de Recherche du Quebec -Sante [288491]; Fondation Chiropratique du

Quebec

FX IP received a grant from the Fonds de Recherche du Quebec -Sante

(288491). PR received a scholarships from the Fondation Chiropratique du

Quebec. The funding bodies have no role in the design of the study, data

collection, analysis, and interpretation, and in writing the manuscript.

CR Arksey H., 2005, INT J SOC RES METHOD, V8, P19, DOI [10.1080/1364557032000119616, DOI 10.1080/1364557032000119616]

Berdishevsky H, 2016, SCOLIOSIS SPINAL DIS, V11, DOI 10.1186/s13013-016-0076-9

Black DAJ, 2017, SCOLIOSIS SPINAL DIS, V12, DOI 10.1186/s13013-017-0141-z

Bussieres AE, 2016, BMC COMPLEM ALTERN M, V16, DOI 10.1186/s12906-016-1175-0

Ciazynski D, 2008, STUD HEALTH TECHNOL, V140, P281, DOI 10.3233/978-1-58603-888-5-281

Drake S, 2014, SCOLIOSIS SPINAL DIS, V9

Freedman KB, 1998, J BONE JOINT SURG AM, V80A, P1421, DOI 10.2106/00004623-199810000-00003

Humphreys BK, 2007, J MANIP PHYSIOL THER, V30, P44, DOI 10.1016/j.jmpt.2006.11.006

Moola S., 2017, JBI MANUAL EVIDENCE, P219, DOI DOI 10.46658/JBIMES-20-08

Negrini S, 2018, SCOLIOSIS SPINAL DIS, V13, DOI 10.1186/s13013-017-0145-8

Negrini S, 2012, SCOLIOSIS SPINAL DIS, V7, DOI 10.1186/1748-7161-7-3

Theroux J, 2017, J MANIP PHYSIOL THER, V40, P452, DOI 10.1016/j.jmpt.2017.03.009

Theroux Jean, 2013, J Can Chiropr Assoc, V57, P251

Tricco AC, 2018, ANN INTERN MED, V169, P467, DOI 10.7326/M18-0850

Weiss HR, 2006, SCOLIOSIS SPINAL DIS, V1, DOI [10.1186/1748-7161-1-5, 10.1186/1748-7161-1-1]

NR 15

TC 0

Z9 0

U1 0

U2 1

PU ELSEVIER

PI AMSTERDAM

PA RADARWEG 29, 1043 NX AMSTERDAM, NETHERLANDS

SN 2468-7812

J9 MUSCULOSKEL SCI PRAC

JI Musculoskelet. Sci. Pract.

PD OCT

PY 2022

VL 61

AR 102595

DI 10.1016/j.msksp.2022.102595

EA JUN 2022

PG 5

WC Rehabilitation

WE Science Citation Index Expanded (SCI-EXPANDED)

SC Rehabilitation

GA 2F5EZ

UT WOS:000812933700005

PM 35688012

DA 2023-08-10

ER

PT J

AU Kozinoga, M

Stolinski, L

Korbel, K

Politarczyk, K

Janusz, P

Kotwicki, T

AF Kozinoga, Mateusz

Stolinski, Lukasz

Korbel, Krzysztof

Politarczyk, Katarzyna

Janusz, Piotr

Kotwicki, Tomasz

TI Regular School Sport versus Dedicated Physical Activities for Body

Posture-A Prospective Controlled Study Assessing the Sagittal Plane in

7-10-Year-Old Children

SO JOURNAL OF CLINICAL MEDICINE

LA English

DT Article

DE body posture; sports activity; corrective exercises; digital photography

ID BONE MASS; RELIABILITY

AB Body posture develops during the growing period and can be documented using trunk photography. The study aims to evaluate the body posture in children aged 7-10 years undergoing a dedicated physical activities program versus regular school sport. A total of 400 children, randomly chosen from a cohort of 9300 participating in a local scoliosis screening program, were evaluated twice at a one-year interval. A total of 167 children were involved in regular school sport (control group), while 233 received both school sport and a dedicated physical activities program (intervention group). Standardized photographic habitual body posture examination was performed at enrollment (T0) and one-year after (T1). Sacral slope (SS), lumbar lordosis (LL), thoracic kyphosis (TK), chest inclination (CI), and head protraction (HP) were measured. At T0, the body posture parameters did not differ between groups. At T1 in the controls, all five parameters tended to deteriorate (insignificant): SS p = 0.758, LL p = 0.38, TK p = 0.328, CI p = 0.081, and HP p = 0.106. At T1 in the intervention group, the SS decreased (p = 0.001), the LL tended to decrease (p = 0.0602), and the TK, CI, and HP remained unaltered. At T1, the SS and LL parameter differed between groups statistically (p = 0.0002 and p = 0.0064, respectively) and clinically (2.52 degrees and 2.58 degrees, respectively). In 7-10-year-old children, participation in dedicated physical activities tends to improve their body posture compared to regular school sport.

C1 [Kozinoga, Mateusz; Politarczyk, Katarzyna; Janusz, Piotr; Kotwicki, Tomasz] Univ Med Sci, Dept Spine Disorders & Pediat Orthoped, PL-61545 Poznan, Poland.

[Stolinski, Lukasz] Spine Disorders Ctr, PL-96100 Skierniewice, Poland.

[Korbel, Krzysztof] Univ Med Sci, Dept Physiotherapy, PL-61545 Poznan, Poland.

RP Kozinoga, M (通讯作者)， Univ Med Sci, Dept Spine Disorders & Pediat Orthoped, PL-61545 Poznan, Poland.

EM stolinskilukasz@op.pl; stolinskilukasz@op.pl; kkorbel@ump.edu.pl;

stolinskilukasz@op.pl; stolinskilukasz@op.pl; stolinskilukasz@op.pl

OI Kozinoga, Mateusz/0000-0003-1469-9388

FU Poznan University of Medical Sciences [502-14-01115157-41181]; EEA
Financial Mechanism
FX This study was supported by the Poznan University of Medical Sciences
(Young Medical Scientist grant number 502-14-01115157-41181). The
activities program was supported with a grant from Iceland,
Liechtenstein, and Norway through the EEA Financial Mechanism.
CR Angelakopoulos G., 2008, HELL J PHYS ED SPORT, V68, P35
[Anonymous], 2009, NUTR REV, V67, P114, DOI 10.1111/j.1753-4887.2008.00136.x
Bielemann RM, 2013, BMC MUSCULOSKEL DIS, V14, DOI 10.1186/1471-2474-14-77
Canales JZ, 2010, REV BRAS PSIQUIATR, V32, P375, DOI 10.1590/S1516-44462010000400010
Cerny P., 2016, LOCOMOT SYST J, V23, P32
Claus AP, 2009, MANUAL THER, V14, P404, DOI 10.1016/j.math.2008.06.001
Cosma G., 2015, PHYS ED SPORT KINETO, V11, P39
Czaprowski D, 2018, SCOLIOSIS SPINAL DIS, V13, DOI 10.1186/s13013-018-0151-5
Dewitte V, 2018, MUSCULOSKEL SCI PRAC, V34, P66, DOI 10.1016/j.msksp.2018.01.002
Dunk NM, 2005, J MANIP PHYSIOL THER, V28, P386, DOI 10.1016/j.jmpt.2005.06.006
Dutkiewicz R., 2010, SKUTECZNOSC ZAJEC KO
Hemming K, 2017, BMJ-BRIT MED J, V358, DOI 10.1136/bmj.j3064
Kulaga Z, 2015, STANDARDY MEDYCZNE, V12, P119
McEvoy MP, 2005, BMC MUSCULOSKEL DIS, V6, DOI 10.1186/1471-2474-6-35
Neinstein L. S., 2002, ADOLESCENT HLTH CARE
Shumway- Cook A, 2001, MOTOR CONTROL THEORY
Stolinski L, 2017, SCOLIOSIS SPINAL DIS, V12, DOI 10.1186/s13013-017-0146-7
Stolinski L, 2015, THESIS POZNAN U MED
Stolinski L., 2014, SCOLIOSIS SPINAL DIS, V9, P15, DOI [10.1186/1748-7161-9-S1-O15, DOI 10.1186/1748-7161-9-S1-O15, 10.1186/1748-7161-9-s1-o15]

Stolinski L, 2012, STUD HEALTH TECHNOL, V176, P242, DOI 10.3233/978-1-61499-067-3-242

Sundberg M, 2002, CALCIFIED TISSUE INT, V71, P406, DOI 10.1007/s00223-001-1105-z

Torlakovic A., 2013, Journal of Health Sciences, V3, P103

van Niekerk SM, 2008, BMC MUSCULOSKEL DIS, V9, DOI 10.1186/1471-2474-9-113

Weir JP, 2005, J STRENGTH COND RES, V19, P231, DOI 10.1519/15184.1

WELTEN DC, 1994, J BONE MINER RES, V9, P1089, DOI 10.1002/jbmr.5650090717

Young Stephen, 2002, J Audiov Media Med, V25, P94, DOI 10.1080/014051102320376799

NR 26

TC 0

Z9 0

U1 6

U2 13

PU MDPI

PI BASEL

PA ST ALBAN-ANLAGE 66, CH-4052 BASEL, SWITZERLAND

EI 2077-0383

J9 J CLIN MED

JI J. Clin. Med.

PD MAR

PY 2022

VL 11

IS 5

AR 1255

DI 10.3390/jcm11051255

PG 8

WC Medicine, General & Internal

WE Science Citation Index Expanded (SCI-EXPANDED)

SC General & Internal Medicine

GA ZW5ZO

UT WOS:000771291000001

PM 35268346

OA gold, Green Published

DA 2023-08-10

ER

PT J

AU Rafferty, A

Fleming, N

Kiely, P

Mockler, D

Dockrell, S

AF Rafferty, Anthony

Fleming, Neil

Kiely, Patrick

Mockler, David

Dockrell, Sara

TI Does exercise therapy improve pulmonary function in patients with Adolescent Idiopathic Scoliosis?

SO PHYSIOTHERAPY THEORY AND PRACTICE

LA English

DT Article

DE Adolescent idiopathic scoliosis; pulmonary function; exercise therapy;

physiotherapeutic scoliosis-specific exercises

ID SCHROTH EXERCISE; VITAL CAPACITY; COBBS ANGLE; REHABILITATION; PROGRAM;

EFFICACY; HEALTH

AB Introduction Exercise therapy is frequently used for treating patients with Adolescent Idiopathic Scoliosis (AIS) however no previous review has evaluated the effect of exercise therapy on pulmonary function in this population. Objective To systematically analyze the literature on the effect of exercise therapy on pulmonary function in patients with AIS. Methods A systematic electronic database search (CINAHL, Embase, Medline, Web of Science) was conducted. Manual searches of key reviews and studies were also conducted. Studies that included exercise-based interventions to improve pulmonary function in patients with AIS and reported pre- and post-intervention pulmonary function test scores were included. Test scores were compared using standardized mean difference (SMD) between intervention and control groups in randomized control trials (RCT) and mean +/- SD between pre- and post-intervention in prospective intervention studies (PI). Methodological quality was assessed using a modified Downs and Black checklist. Results Fifteen studies met the inclusion criteria (six RCTs and nine PIs). Results indicated the positive effect of exercise-based therapy on lung volumes (FVC/VC) and FEV1 in patients with AIS. Conclusion Exercise therapy has a positive effect on lung volumes in patients with AIS. The quality of many studies was only 'fair,' therefore more suitably powered higher level clinical trials are required.

C1 [Rafferty, Anthony; Dockrell, Sara] St James Hosp, Trinity Coll Dublin, Sch Med, Discipline Physiotherapy,Trinity Ctr Hlth Sci, Dublin D08 W9RT, Ireland.

[Fleming, Neil] Trinity Biomed Sci Inst, Sch Med, Trinity Coll, Trinity Biomed Sci Inst,Dept Anat, Level 1, Dublin, Ireland.

[Kiely, Patrick] Childrens Hlth Ireland, Dept Orthopaed, Dublin, Ireland.

[Mockler, David] St James Hosp, Trinity Coll Dublin, Sch Med, Trinity Ctr Hlth Sci,Trin Res, Dublin, Ireland.

C3 Trinity College Dublin; Trinity College Dublin; Trinity College Dublin

RP Rafferty, A (通讯作者)， St James Hosp, Trinity Coll Dublin, Sch Med, Discipline Physiotherapy,Trinity Ctr Hlth Sci, Dublin D08 W9RT, Ireland.

EM raffertt@tcd.ie

OI Mockler, David/0000-0001-6171-8751

CR Abdelaal AAM, 2018, J INT MED RES, V46, P381, DOI 10.1177/0300060517715375

Amaricai E, 2019, J INT MED RES, DOI 10.1177/0300060519895093

Athanasopoulos S, 1999, SCAND J MED SCI SPOR, V9, P36

Berdishevsky H, 2016, SCOLIOSIS SPINAL DIS, V11, DOI 10.1186/s13013-016-0076-9

Borysov M, 2012, SCOLIOSIS SPINAL DIS, V7, DOI 10.1186/1748-7161-7-1

Chopra S, 2020, PLOS ONE, V15, DOI 10.1371/journal.pone.0238181

Day Joseph M, 2019, Arch Physiother, V9, P8, DOI 10.1186/s40945-019-0060-9

Dobosiewicz Krystyna, 2002, Stud Health Technol Inform, V91, P336

Alves VLD, 2006, CHEST, V130, P500, DOI 10.1378/chest.130.2.500

Downs SH, 1998, J EPIDEMIOL COMMUN H, V52, P377, DOI 10.1136/jech.52.6.377

Fabian K., 2010, PHYSIOTHERAPY, V18, P212, DOI [10.2478/v10109-010-0072-1, DOI 10.2478/V10109-010-0072-1]

Fabian Krzysztof Marek, 2010, Ortop Traumatol Rehabil, V12, P301

Gao CF, 2019, AM J PHYS MED REHAB, V98, P642, DOI 10.1097/PHM.0000000000001160

Golshan M, 2007, RESPIROLOGY, V12, P361, DOI 10.1111/j.1440-1843.2007.01070.x

Graham BL, 2019, AM J RESP CRIT CARE, V200, pE70, DOI 10.1164/rccm.201908-1590ST

Grant J, 2006, J INTELL INF SYST, V27, P159, DOI 10.1007/s10844-006-2974-4

Grivas TB, 2006, SCOLIOSIS SPINAL DIS, V1, DOI 10.1186/1748-7161-1-9

Hancox RJ, 2018, EUR RESPIR J, V51, DOI 10.1183/13993003.01374-2017

Hooper P, 2008, CAN J OPHTHALMOL, V43, P180, DOI 10.3129/i08-001

KEARON C, 1993, AM REV RESPIR DIS, V148, P288, DOI 10.1164/ajrccm/148.2.288

Khosravi M, 2013, IRAN J BASIC MED SCI, V16, P628

Kim KD, 2016, J PHYS THER SCI, V28, P923, DOI 10.1589/jpts.28.923

Kim Min-Jae, 2017, Physical therapy rehabilitation science, V6, P113, DOI 10.14474/ptrs.2017.6.3.113

Kumar A, 2017, J CLIN DIAGN RES, V11, pYC1, DOI 10.7860/JCDR/2017/27497.10335

Kumar B, 2015, CLIN MED, V15, P267, DOI 10.7861/clinmedicine.15-3-267

Lenhert-Schroth C., 1992, PHYSIOTHERAPY, V78, P810, DOI [10.1016/S0031-9406(10)60451-8, DOI 10.1016/S0031-9406(10)60451-8]

Lenke LG, 2001, J BONE JOINT SURG AM, V83A, P1169, DOI 10.2106/00004623-200108000-00006

Lorente A, 2017, SPINE, V42, P1391, DOI 10.1097/BRS.0000000000002105

Moramarco M, 2016, CURR PEDIATR REV, V12, P17, DOI 10.2174/1573396312666151117120514

Negrini A, 2016, SCOLIOSIS SPINAL DIS, V11, DOI 10.1186/s13013-016-0100-0

Neve V, 2002, EUR RESPIR J, V20, P1292, DOI 10.1183/09031936.02.00208102

Otman S, 2005, SAUDI MED J, V26, P1429

Park JH, 2018, EUR J PHYS REHAB MED, V54, P440, DOI 10.23736/S1973-9087.17.04461-6

RAWASHDEH A, 2018, BIOMED PHARMACOL J, V11, P735, DOI DOI 10.13005/bpj/1427

Romano M, 2015, SCOLIOSIS SPINAL DIS, V10, DOI 10.1186/s13013-014-0027-2

Barroso AT, 2018, ARCH BRONCONEUMOL, V54, P327, DOI 10.1016/j.arbres.2018.01.030

Tsiligiannis T, 2012, SCOLIOSIS SPINAL DIS, V7, DOI 10.1186/1748-7161-7-7

Hui W, 2015, J TRADIT CHIN MED, V35, P514, DOI 10.1016/S0254-6272(15)30133-3

WEISS HR, 1991, SPINE, V16, P88, DOI 10.1097/00007632-199101000-00016

Wong HK, 2005, SPINE, V30, P1188, DOI 10.1097/01.brs.0000162280.95076.bb

Xavier VB, 2020, J PHYSIOTHER, V66, P33, DOI 10.1016/j.jphys.2019.11.012

Zhou ZW, 2021, COMPLEMENT THER MED, V58, DOI 10.1016/j.ctim.2021.102697

NR 42

TC 0

Z9 0

U1 8

U2 14

PU TAYLOR & FRANCIS INC

PI PHILADELPHIA

PA 530 WALNUT STREET, STE 850, PHILADELPHIA, PA 19106 USA

SN 0959-3985

EI 1532-5040

J9 PHYSIOTHER THEOR PR

JI Physiother. Theory Pract.

PD JUN 3

PY 2023

VL 39

IS 6

BP 1095

EP 1105

DI 10.1080/09593985.2022.2034198

EA FEB 2022

PG 11

WC Rehabilitation

WE Science Citation Index Expanded (SCI-EXPANDED)

SC Rehabilitation

GA H3UD3

UT WOS:000757693200001

PM 35176949

OA Green Published

DA 2023-08-10

ER

PT J

AU David, M

Raison, M

Paul, S

Cartiaux, O

Detrembleur, C

Mahaudens, P

AF David, Mercedes

Raison, Maxime

Paul, Stephanie

Cartiaux, Olivier

Detrembleur, Christine

Mahaudens, Philippe

TI Locoregional lung ventilation distribution in girls with adolescent

idiopathic scoliosis and healthy adolescents. The immediate effect of

Schroth 'derotational breathing' exercise in a controlled-trial

SO PHYSIOTHERAPY THEORY AND PRACTICE

LA English

DT Article

DE Schroth method; electrical impedance tomography (EIT); idiopathic

scoliosis; regional ventilation

ID ELECTRICAL-IMPEDANCE TOMOGRAPHY; DIAPHRAGM MOTIONS; VITAL CAPACITY;

TIDAL VOLUME; CHEST-WALL; ASYMMETRY; CHILDREN; SPINE; CAGE

AB Background Scoliosis curves present transverse plane deviations due to vertebral rotation. The Schroth method supports thoracic derotation by training patients to exert "derotational" breathing based on assumed enhanced ventilation in areas called "humps" in scoliosis and a patient's ability to voluntarily direct ventilation in less ventilated areas called "flats." Objective To assess the asymmetric ventilation distribution and the ability of patients to direct their ventilation to perform derotational breathing. Methods Twelve girls with adolescent idiopathic scoliosis and 12 healthy girls performed 3 x 3 min of rest, maximal, and derotational breathing. Electrical impedance tomography was used to record locoregional lung ventilation distribution (LLVD) within 4 thoracic regions of interest: anterior right (ROI 1), anterior left (ROI 2), posterior right (ROI 3), and posterior left (ROI

4) quadrants. Humps and flats were the sums of ROI '2 + 3' and ROI '1 + 4,' respectively. Results Overall, no difference in LLVD was observed in the flats and humps between groups. At rest, the LLVD in the humps was more elevated than that in the flats (51.5 +/- 8.1% versus 43.6 +/- 7.9%; p = .021) when considering both groups. Maximal and derotational breathing led to a more homogeneous LLVD between the humps and flats. Conclusion The postulated derotational breathing effect was not confirmed.

C1 [David, Mercedes; Paul, Stephanie; Cartiaux, Olivier; Detrembleur, Christine; Mahaudens, Philippe] Catholic Univ Louvain, Inst Rech Expt & Clin, Sect Sci Sante, Neuro Musculo Skeletal Lab NMSK, Ave Mounier 53, B-1200 Brussels, Belgium.

[David, Mercedes; Raison, Maxime] Ecole Polytech Montreal, Dept Mech Engn, Montreal, PQ, Canada.

[Raison, Maxime] CHU Ste Justine, Ctr Readaptat Marie Enfant, Dept Rehabil, Montreal, PQ, Canada.

[Paul, Stephanie; Mahaudens, Philippe] Clin Univ St Luc, Serv Orthopedie & Traumatol Appareil Locomoteur, Brussels, Belgium.

[Paul, Stephanie] Clin Univ St Luc, Serv Med Phys & Readaptat, Brussels, Belgium.

C3 Universite Catholique Louvain; Universite de Montreal; Polytechnique

Montreal; Universite de Montreal; Universite Catholique Louvain;

Cliniques Universitaires Saint-Luc; Universite Catholique Louvain;

Cliniques Universitaires Saint-Luc

RP David, M (通讯作者)，Catholic Univ Louvain, Inst Rech Expt & Clin, Sect Sci Sante, Neuro Musculo Skeletal Lab NMSK, Ave Mounier 53, B-1200 Brussels, Belgium.

EM mercedesdavid2018@gmail.com

OI David, Mercedes/0000-0002-3885-9262; Christine,

Detrembleur/0000-0003-0776-3820

CR Adam CJ, 2007, J PEDIATR ORTHOPED, V27, P677, DOI 10.1097/BPO.0b013e318425ee

Bikker IG, 2010, CRIT CARE, V14, DOI 10.1186/cc9036

Borysov M, 2012, SCOLIOSIS SPINAL DIS, V7, DOI 10.1186/1748-7161-7-1

Cheung J, 2006, SPINE, V31, P322, DOI 10.1097/01.brs.0000197155.68983.d8

Choudhry Muhammad Naghman, 2016, Open Orthop J, V10, P143, DOI 10.2174/1874325001610010143

Chu WCW, 2006, SPINE, V31, P2243, DOI 10.1097/01.brs.0000232822.74349.32

CLOSKEY RF, 1993, J ORTHOPAED RES, V11, P730, DOI 10.1002/jor.1100110515

Cote P, 1998, SPINE, V23, P796, DOI 10.1097/00007632-199804010-00011

Courvoisier A, 2013, EUR SPINE J, V22, P2427, DOI 10.1007/s00586-013-2862-x

Dayer R, 2013, J CHILD ORTHOP, V7, P11, DOI 10.1007/s11832-012-0458-3

Dolan LA, 2007, SPINE, V32, pS91, DOI 10.1097/BRS.0b013e318134ead9

Frerichs I, 1999, IEEE T MED IMAGING, V18, P764, DOI 10.1109/42.802754

Frerichs I, 2003, INTENS CARE MED, V29, P2312, DOI 10.1007/s00134-003-2029-z

Frerichs I, 2017, THORAX, V72, P83, DOI 10.1136/thoraxjnl-2016-208357

Gilroy AM., 2012, ATLAS ANATOMY

Grivas TB, 2014, SCOLIOSIS SPINAL DIS, V9, DOI 10.1186/s13013-014-0020-9

Grivas TB, 2010, SCOLIOSIS SPINAL DIS, V5, DOI 10.1186/1748-7161-5-23

Karsten J, 2016, CRIT CARE, V20, DOI 10.1186/s13054-015-1161-9

Kim KD, 2016, J PHYS THER SCI, V28, P923, DOI 10.1589/jpts.28.923

Kotani T, 2004, SPINE, V29, P298, DOI 10.1097/01.BRS.0000106490.82936.89

Kouwenhoven JWM, 2006, SPINE, V31, P1467, DOI 10.1097/01.brs.0000219938.14686.b3

Lehnert-Schroth C., 2012, TRAITEMENT TRIDIMENS

Lenhert-Schroth C., 1992, PHYSIOTHERAPY, V78, P810, DOI [10.1016/S0031-9406(10)60451-8, DOI 10.1016/S0031-9406(10)60451-8]

Leong JCY, 1999, SPINE, V24, P1310, DOI 10.1097/00007632-199907010-00007

LITTLER WA, 1972, THORAX, V27, P420, DOI 10.1136/thx.27.4.420

Lowe TG, 2000, J BONE JOINT SURG AM, V82A, P1157, DOI 10.2106/00004623-200008000-00014

Lundin S, 2012, CURR OPIN CRIT CARE, V18, P35, DOI 10.1097/MCC.0b013e32834eb462

MILICEMI.J, 1966, J APPL PHYSIOL, V21, P749, DOI 10.1152/jappl.1966.21.3.749

Murray J. F., 1986, NORMAL LUNG BASIS DI

Negrini S, 2012, SCOLIOSIS SPINAL DIS, V7, DOI 10.1186/1748-7161-7-3

Paulsen F., 2011, SOBOTTA

PERDRIOLLE R, 1985, SPINE, V10, P785, DOI 10.1097/00007632-198511000-00001

PONSETI IV, 1950, J BONE JOINT SURG AM, V32-A, P381, DOI 10.2106/00004623-195032020-00017

Pugacheva N, 2012, STUD HEALTH TECHNOL, V176, P365, DOI 10.3233/978-1-61499-067-3-365

Pulletz S, 2006, PHYSIOL MEAS, V27, pS115, DOI 10.1088/0967-3334/27/5/S10

Redding G, 2008, SPINE J, V8, P639, DOI 10.1016/j.spinee.2007.04.020

Rigo M, 2009, STUD HEALTH TECHNOL, V135, P208

Romano M, 2012, COCHRANE DB SYST REV, DOI 10.1002/14651858.CD007837.pub2

Sch?nke M., 2006, ATLAS DANATOMIE PROM

Schreiber S, 2016, PLOS ONE, V11, DOI 10.1371/journal.pone.0168746

Scoliosis-Research-Society, 2019, TREAT SCOL

Shah SA, 2007, NEUROSURG CLIN N AM, V18, P339, DOI 10.1016/j.nec.2007.02.003

Standring, 2005, GRAYS ANATOMY INT ED

Staubesand J., 1990, SOBOTTA ATLAS HUMAN

Szopa A, 2017, MEDICINE, V96, DOI 10.1097/MD.0000000000007032

Teschner E., 2015, ELECT IMPEDANCE TOMO

Trepte CJC, 2017, BRIT J ANAESTH, V118, P68, DOI 10.1093/bja/aew341

Tsiligiannis T, 2012, SCOLIOSIS SPINAL DIS, V7, DOI 10.1186/1748-7161-7-7

Victorino JA, 2004, AM J RESP CRIT CARE, V169, P791, DOI 10.1164/rccm.200301-133OC

Weinstein SL, 2008, LANCET, V371, P1527, DOI 10.1016/S0140-6736(08)60658-3

WEISS HR, 1991, SPINE, V16, P88, DOI 10.1097/00007632-199101000-00016

NR 51

TC 0

Z9 0

U1 3

U2 6

PU TAYLOR & FRANCIS INC

PI PHILADELPHIA

PA 530 WALNUT STREET, STE 850, PHILADELPHIA, PA 19106 USA

SN 0959-3985

EI 1532-5040

J9 PHYSIOTHER THEOR PR

JI Physiother. Theory Pract.

PD MAY 4

PY 2023

VL 39

IS 5

BP 938

EP 953

DI 10.1080/09593985.2022.2033896

EA FEB 2022

PG 16

WC Rehabilitation

WE Science Citation Index Expanded (SCI-EXPANDED)

SC Rehabilitation

GA J5ZY6

UT WOS:000750386200001

PM 35105251

DA 2023-08-10

ER

PT J

AU Lin, YX

Feng, EW

Shen, JX

Tan, HN

Jiao, Y

Rong, TH

Chen, LX

Yuan, WS

Cong, H

Liu, SF

Luo, JM

AF Lin, Youxi

Feng, Erwei

Shen, Jianxiong

Tan, Haining

Jiao, Yang

Rong, Tianhua

Chen, Lixia

Yuan, Wangshu

Cong, Hui

Liu, Shufen

Luo, Jinmei

TI Influences of Thoracic Spinal Deformity on Exercise Performance and

Pulmonary Function A Prospective Study of 168 Patients with Adolescent

Idiopathic Scoliosis

SO SPINE

LA English

DT Article

DE adolescent idiopathic scoliosis; cardiopulmonary exercise test; exercise

tolerance; pulmonary ventilation; spirometry

ID LUNG-FUNCTION; LIMITATION

AB Study Design. A prospective study. Objective. The aim of this study was to investigate the factors associated with cardiopulmonary exercise testing (CPET) measurements in patients with adolescent idiopathic scoliosis (AIS). Summary of Background Data. Patients with AIS display restrictive pulmonary dysfunction on pulmonary function testing (PFT). It remains unknown whether thoracic spinal deformity affects exercise performance. Methods. One hundred and sixty-eight patients with AIS from January 2014 to December 2019 were included. They underwent preoperative spinal radiological assessment, PFT, and CPET. The effects of the thoracic curve magnitude, body mass index, physical activity level and history of bracing on pulmonary function and exercise performance were analyzed. The Student t test and two-tailed Pearson test were used in data analysis. Results. We found significantly reduced forced expiratory volume in 1second (FEV1) in patients with a larger magnitude of the proximal thoracic curve ($P < 0.001$) and the main thoracic curve ($P < 0.001$). There was a negative correlation between forced vital capacity (FVC) and the magnitude of the main thoracic curve ($P < 0.001$) and thoracic hypokyphosis ($P < 0.001$). In CPET, exercise capacity indicators such as the work rate, peak oxygen intake, and heart rate were not affected by the thoracic curve magnitude. Patients with moderate or severe pulmonary dysfunction had decreased tidal volume ($P = 0.01$) and ventilatory reserve ($P < 0.001$), as well as increased respiratory frequency at maximal exercise ($P = 0.01$). Patients with a moderate or high physical activity level had better exercise capacity, which was reflected by a higher work rate ($P = 0.009$) and oxygen intake ($P < 0.001$). Conclusion. There was no significant correlation between radiographic parameters and exercise capacity indicators. When the thoracic curve increased, patients had restrictive ventilatory dysfunction, which led to a tachypneic breathing pattern and reduction of ventilatory reserve during exercise. A physiological change of improved peak oxygen intake was demonstrated in patients with a moderate or high physical activity level.

C1 [Lin, Youxi; Feng, Erwei; Shen, Jianxiong; Tan, Haining; Jiao, Yang; Rong, Tianhua] Chinese Acad Med Sci, Dept Orthoped Surg, Peking Union Med Coll Hosp, Peking Union Med Coll, 1 Shuai Fu Yuan,Wang Fu Jing St, Beijing 100730, china.

[Chen, Lixia; Yuan, Wangshu; Cong, Hui; Liu, Shufen] Chinese Acad Med Sci, Dept Phys Med & Rehabil, Peking Union Med Coll Hosp, Peking Union Med Coll, Beijing, china.

[Luo, Jinmei] Chinese Acad Med Sci, De Dept Resp Med, Peking Union Med Coll Hosp, Peking Union Med Coll, Beijing, china.

[Lin, Youxi] Capital Med Univ, Beijing Tiantan Hosp, Dept Orthoped Surg, Beijing, china.

C3 Chinese Academy of Medical Sciences - Peking Union Medical College;

Peking Union Medical College; Peking Union Medical College Hospital;

Chinese Academy of Medical Sciences - Peking Union Medical College;

Peking Union Medical College; Peking Union Medical College Hospital;

Chinese Academy of Medical Sciences - Peking Union Medical College;

Peking Union Medical College; Peking Union Medical College Hospital;

Capital Medical University

RP Shen, JX (通讯作者)， Chinese Acad Med Sci, Dept Orthoped Surg, Peking Union Med Coll Hosp, Peking Union Med Coll, 1 Shuai Fu Yuan,Wang Fu Jing St, Beijing 100730, china.

EM sjxpumch@163.com

RI Lin, Youxi/AEY-8541-2022; Rong, Tianhua/GNH-5575-2022; Luo,

jinmei/AFF-6995-2022; Luo, jinmei/AAE-9922-2022

OI Rong, Tianhua/0000-0002-7631-5418; Luo, jinmei/0000-0001-5519-6205; Luo,

jinmei/0000-0001-5519-6205

CR ATS, 2003, AM J RESP CRIT CARE, V167, P211, DOI 10.1164/rccm.167.2.211

BRANTHWAITE MA, 1986, BRIT J DIS CHEST, V80, P360, DOI 10.1016/0007-0971(86)90089-6

Cicoira M, 2001, J AM COLL CARDIOL, V37, P2080, DOI 10.1016/S0735-1097(01)01306-7

Czaprowski D, 2012, EUR SPINE J, V21, P1099, DOI 10.1007/s00586-011-2068-z

De Lorenzo A, 2017, CLIN CARDIOL, V40, P914, DOI 10.1002/clc.22747

Guenette JA, 2013, PULM MED, V2013, DOI 10.1155/2013/956081

IPAQ Research Committee, 2005, GUIDELINES DATA PROC

Johnston CE, 2011, SPINE, V36, P1096, DOI 10.1097/BRS.0b013e3181f8c931

Lin YX, 2019, J BONE JOINT SURG AM, V101, P1109, DOI 10.2106/JBJS.18.00935

Martinez-Llorens J, 2010, EUR RESPIR J, V36, P393, DOI 10.1183/09031936.00025509

Miller MR, 2005, EUR RESPIR J, V26, P319, DOI 10.1183/09031936.05.00034805

Newton PO, 2005, J BONE JOINT SURG AM, V87A, P1937, DOI 10.2106/JBJS.D.02209

PEHRSSON K, 1991, THORAX, V46, P474, DOI 10.1136/thx.46.7.474

Pellegrino R, 2005, EUR RESPIR J, V26, P948, DOI 10.1183/09031936.05.00035205

Shen JX, 2016, J BONE JOINT SURG AM, V98, P1614, DOI 10.2106/JBJS.15.01403

Sperandio EF, 2014, SPINE J, V14, P2366, DOI 10.1016/j.spinee.2014.01.041

Wagner PD, 1996, ANNU REV PHYSIOL, V58, P21, DOI 10.1146/annurev.physiol.58.1.21

Wasserman K, 2012, PRINCIPLES EXERCISE

WEINSTEIN SL, 1981, J BONE JOINT SURG AM, V63, P702, DOI 10.2106/00004623-198163050-00003

Weinstein SL, 2003, JAMA-J AM MED ASSOC, V289, P559, DOI 10.1001/jama.289.5.559

Yaszay B, 2017, EUR SPINE J, V26, P1658, DOI 10.1007/s00586-016-4694-y

NR 21

TC 1

Z9 1

U1 2

U2 7

PU LIPPINCOTT WILLIAMS & WILKINS

PI PHILADELPHIA

PA TWO COMMERCE SQ, 2001 MARKET ST, PHILADELPHIA, PA 19103 USA

SN 0362-2436

EI 1528-1159

J9 SPINE

JI SPINE

PD FEB 1

PY 2022

VL 47

IS 3

BP E107

EP E115

DI 10.1097/BRS.0000000000004161

PG 9

WC Clinical Neurology; Orthopedics

WE Science Citation Index Expanded (SCI-EXPANDED)

SC Neurosciences & Neurology; Orthopedics

GA YC3CG

UT WOS:000739571700003

PM 34265810

DA 2023-08-10

ER

PT J

AU Romano, A

Ippolito, E

Risoli, C

Malerba, E

Favetta, M

Sancesario, A

Lotan, M

Moran, DS

AF Romano, Alberto

Ippolito, Elena

Risoli, Camilla

Malerba, Edoardo

Favetta, Martina

Sancesario, Andrea

Lotan, Meir

Moran, Daniel Sender

TI Intensive Postural and Motor Activity Program Reduces Scoliosis

Progression in People with Rett Syndrome

SO JOURNAL OF CLINICAL MEDICINE

LA English

DT Article

DE Rett syndrome; scoliosis; motor skills; telerehabilitation; physical

therapy modalities; home exercise program

ID NEUROMUSCULAR SCOLIOSIS; NATURAL-HISTORY; SPINAL-FUSION; MANAGEMENT;

SURGERY; DISABILITIES; PREVALENCE; MUTATIONS; CHILDREN

AB Background: A scoliosis prevalence of 94% was reported in the population with Rett syndrome (RTT), with an annual progression rate of 14 to 21 degrees Cobb which may result in pain, loss of sitting balance, deterioration of motor skills, and lung disfunction. This paper describes the efficacy of an intensive conservative individualized physical and postural activity program in preventing scoliosis curvature progression in patients with RTT. Methods: Twenty subjects diagnosed with RTT and scoliosis were recruited, and an individualized intensive daily physical activity program was developed for each participant. Each program was conducted for six months by participants' primary caregivers in their daily living environment. Fortnightly remote supervision of the program implementation was provided by an expert therapist. Pre- and post-intervention radiographs and motor functioning were analyzed. Results: An averaged progression of +1.7 degrees +/- 8.7 degrees Cobb, over one year (12.3 +/- 3.5 months) was observed in our group, together with motor function improvements. A relation between curve progression and motor skill improvement was observed. Conclusions: The intervention prevented scoliosis progression in our group. The achievement of functional motor improvements could enable better body segment control and muscle balancing, with a protective effect on scoliosis progression. The intervention was effective for individuals with RTT across various ages and severity levels. Individual characteristics of each participant and the details of their activity program are described.

C1 [Romano, Alberto; Moran, Daniel Sender] Ariel Univ, Dept Hlth Syst Management, IL-4070000 Ariel, Israel.

[Romano, Alberto; Favetta, Martina; Sancesario, Andrea] Bambino Gesu Pediat Hosp, Intens Neurorehabil & Robot Dept, Movement Anal & Robot Lab, I-00165 Rome, Italy.

[Romano, Alberto] Res & Innovat AIRETT Ctr, CARI, I-37122 Verona, Italy.

[Ippolito, Elena] SMART Learning Ctr, I-20133 Milan, Italy.

[Risoli, Camilla] Guglielmo da Saliceto Hosp, Radiol Unit, Dept Radiol Funct, I-29121 Piacenza, Italy.

[Malerba, Edoardo] Poliambulatorio Hlth Med, I-29122 Piacenza, Italy.

[Lotan, Meir] Ariel Univ, Dept Physiotherapy, IL-4070000 Ariel, Israel.

[Lotan, Meir] Israeli Rett Syndrome Natl Evaluat Team, IL-5200100 Ramat Gan, Israel.

C3 Ariel University; IRCCS Bambino Gesu; Guglielmo da Saliceto Hospital;

Ariel University

RP Romano, A (通讯作者)，Ariel Univ, Dept Hlth Syst Management, IL-4070000 Ariel, Israel.; Romano, A (通讯作者)，Bambino Gesu Pediat Hosp, Intens Neurorehabil & Robot Dept, Movement Anal & Robot Lab, I-00165 Rome, Italy.; Romano, A (通讯作者)，Res & Innovat AIRETT Ctr, CARI, I-37122 Verona, Italy.

EM alberto.romano01@ateneopv.it; elena.ippolito@centrosmart.it;

camilla.risoli11@gmail.com; edoardo.malerba11@gmail.com;

martina.favetta@opbg.net; andrea.sancesario@opbg.net;

meirlo@ariel.ac.il; danielm@ariel.ac.il

OI Romano, Alberto/0000-0001-9774-2964; Risoli,

Camilla/0000-0003-0117-7011; Malerba, Edoardo/0000-0002-5851-9214

FU International Rett Syndrome Foundation [3610]

FX This research was funded by the International Rett Syndrome Foundation,

within the HeART grant number 3610.

CR Ager S, 2006, J CHILD NEUROL, V21, P809, DOI 10.1177/08830738060210091501

Ager S, 2009, DISABIL REHABIL, V31, P1917, DOI 10.1080/09638280902846392

Amir RE, 1999, NAT GENET, V23, P185, DOI 10.1038/13810

Armstrong RA, 2014, OPHTHAL PHYSL OPT, V34, P502, DOI 10.1111/opo.12131

Barney CC, 2017, PEDIATR ANESTH, V27, P290, DOI 10.1111/pan.13066

BASSETT GS, 1990, DEV MED CHILD NEUROL, V32, P963

Bisgaard AM, 2021, AM J MED GENET A, V185, P3683, DOI 10.1002/ajmg.a.62429

COHEN J, 1992, PSYCHOL BULL, V112, P155, DOI 10.1037/0033-2909.112.1.155

Cohen J., 2013, STAT POWER ANAL BEHA, V2nd

Downs J, 2016, SPINE, V41, P856, DOI 10.1097/BRS.0000000000001399

Downs J, 2016, DEV MED CHILD NEUROL, V58, P632, DOI 10.1111/dmcn.12984

Downs J, 2016, DEV NEUROREHABIL, V19, P31, DOI 10.3109/17518423.2014.898107

Downs J, 2009, J PEDIATR ORTHOPED, V29, P369, DOI 10.1097/BPO.0b013e3181a53b41

Downs J, 2009, SPINE, V34, pE607, DOI 10.1097/BRS.0b013e3181a95ca4

Fabio R.A., 2005, LIFE SPAN DISABIL, V8, P257

Ferrari A, 2010, EUR J PHYS REHAB MED, V46, P563

Fombonne E, 2003, INT REV PSYCHIATR, V15, P158, DOI 10.1080/0954026021000046119

HAGBERG B, 1986, AM J MED GENET, V24, P47

Hagberg B, 1993, RETT SYNDROME CLIN B

Haleem S., 2018, PAED CHILD HEALT-CAN, V28, P209, DOI [10.1016/j.paed.2018.03.007, DOI 10.1016/J.PAED.2018.03.007]

HANKS SB, 1990, BRAIN DEV-JPN, V12, P157, DOI 10.1016/S0387-7604(12)80201-4

HARRISON DJ, 1990, BRAIN DEV-JPN, V12, P154, DOI 10.1016/S0387-7604(12)80200-2

Horng MH, 2019, COMPUT MATH METHOD M, V2019, DOI 10.1155/2019/6357171

Huang T J, 1994, Orthop Rev, V23, P931

Karimi MT, 2018, J CRANIOVERTEBRAL JU, V9, P3, DOI 10.4103/jcvjs.JCVJS_39_17

KERET D, 1988, J PEDIATR ORTHOPED, V8, P138

Kerr AM, 2003, J CHILD NEUROL, V18, P703, DOI 10.1177/08830738030180101201

Killian JT, 2017, PEDIATR NEUROL, V70, P20, DOI 10.1016/j.pediatrneurol.2017.01.032

KING BM, 2008, STAT REASONING BEHAV

Kinney AR, 2020, ARCH PHYS MED REHAB, V101, P2219, DOI 10.1016/j.apmr.2020.02.017

Kotwicki T, 2008, DISABIL REHABIL, V30, P792, DOI 10.1080/09638280801889584

Larsson EL, 2009, EUR SPINE J, V18, P506, DOI 10.1007/s00586-008-0876-6

LIDSTROM J, 1994, SPINE, V19, P1632, DOI 10.1097/00007632-199407001-00013

LODER RT, 1989, J PEDIATR ORTHOPED, V9, P557, DOI 10.1097/01241398-198909010-00010

Logan SW, 2014, PEDIATR PHYS THER, V26, P418, DOI 10.1097/PEP.0000000000000070

Lor L, 2015, DEV MED CHILD NEUROL, V57, P1137, DOI 10.1111/dmcn.12838

Lotan M, 2004, J INTELL DISABIL RES, V48, P730, DOI 10.1111/j.1365-2788.2003.00589.x

Lotan M., 2011, RETT SYNDROME THERAP

Lotan M., 2022, ASSISTIVE TECHNOLOGI, P147

Lotan M, 2005, THESCIENTIFICWORLDJO, V5, P264, DOI 10.1100/tsw.2005.33

Lotan M, 2021, FRONT PSYCHOL, V12, DOI 10.3389/fpsyg.2021.720927

Lotan M, 2021, DEV NEUROREHABIL, V24, P429, DOI 10.1080/17518423.2021.1914762

Lotan M, 2010, THESCIENTIFICWORLDJO, V10, P778, DOI 10.1100/tsw.2010.79

McClure MK, 1998, AM J OCCUP THER, V52, P196, DOI 10.5014/ajot.52.3.196

Mehta JS, 2003, CURR ORTHOPAED, V17, P313, DOI 10.1016/S0268-0890(03)00002-1

Neul JL, 2008, NEUROLOGY, V70, P1313, DOI 10.1212/01.wnl.0000291011.54508.aa

Olafsson Y, 1999, ST HEAL T, V59, P332

OTTENBACHER KJ, 1995, ARCH PHYS MED REHAB, V76, P123, DOI 10.1016/S0003-9993(95)80021-2

Percy AK, 2010, PEDIATR RES, V67, P435, DOI 10.1203/PDR.0b013e3181d0187f

Pini G, 1996, CLIN GENET, V50, P486

Riise R, 2011, DEV MED CHILD NEUROL, V53, P653, DOI 10.1111/j.1469-8749.2011.03935.x

Roberts SB, 2016, J BACK MUSCULOSKELET, V29, P613, DOI 10.3233/BMR-160675

Rocos B, 2021, CUREUS J MED SCIENCE, V13, DOI 10.7759/cureus.15411

Roidi MLR, 2019, DEV MED CHILD NEUROL, V61, P957, DOI 10.1111/dmcn.14109

Roidi MLR, 2019, PEDIATR NEUROL, V100, P80, DOI 10.1016/j.pediatrneurol.2019.03.005

Romano A, 2022, DISABIL REHABIL, V44, P5898, DOI 10.1080/09638288.2021.1949398

Romano A, 2020, DEV NEUROREHABIL, V23, P485, DOI 10.1080/17518423.2019.1680761

Skjeldal OH, 1997, BRAIN DEV-JPN, V19, P258, DOI 10.1016/S0387-7604(97)00572-X

Stahlhut M, 2017, J CHILD NEUROL, V32, P1009, DOI 10.1177/0883073817728861

Thompson B, 2007, PSYCHOL SCHOOLS, V44, P423, DOI 10.1002/pits.20234

Tomczak M., 2014, TRENDS SPORT SCI, V1, P19, DOI DOI 10.1186/S13054-016-1208-6

Uyanik M, 2003, PEDIATR INT, V45, P68, DOI 10.1046/j.1442-200X.2003.01670.x

Weiss HR, 2006, SCOLIOSIS SPINAL DIS, V1, DOI [10.1186/1748-7161-1-5, 10.1186/1748-7161-1-1]

Westerlund LE, 2001, SPINE, V26, P1984, DOI 10.1097/00007632-200109150-00008

NR 64

TC 3

Z9 3

U1 4

U2 9

PU MDPI

PI BASEL

PA ST ALBAN-ANLAGE 66, CH-4052 BASEL, SWITZERLAND

EI 2077-0383

J9 J CLIN MED

JI J. Clin. Med.

PD FEB

PY 2022

VL 11

IS 3

AR 559

DI 10.3390/jcm11030559

PG 14

WC Medicine, General & Internal

WE Science Citation Index Expanded (SCI-EXPANDED); Social Science Citation Index (SSCI)

SC General & Internal Medicine

GA ZF2RC

UT WOS:000759417900001

PM 35160011

OA gold, Green Published

DA 2023-08-10

ER

PT J

AU Abdel-aziem, AA

Abdelraouf, OR

Ghally, SA

Dahlawi, HA

Radwan, RE

AF Abdel-aziem, Amr A.

Abdelraouf, Osama R.

Ghally, Shahesta A.

Dahlawi, Haytham A.

Radwan, Rafik E.

TI A 10-Week Program of Combined Hippotherapy and Scroth's Exercises

Improves Balance and Postural Asymmetries in Adolescence Idiopathic

Scoliosis: A Randomized Controlled Study

SO CHILDREN-BASEL

LA English

DT Article

DE balance; hippotherapy; posture; Schroth exercises; adolescent idiopathic

scoliosis

ID CEREBRAL-PALSY; OLDER-ADULTS; CHILDREN; STABILITY; IMMEDIATE; SIMULATOR;

EQUILIBRIUM; DEPRIVATION; SYMMETRY; THERAPY

AB Introduction: The most frequent type of spine abnormality throughout adolescence was adolescent idiopathic scoliosis (AIS). Hippotherapy improved posture, balance and gait of different musculoskeletal conditions. Therefore, this study aims to see how hippotherapy combined with Schroth exercises affected postural asymmetry and dynamic balance in AIS compared to traditional physiotherapy (Schroth exercises) alone. Materials and methods: In this randomized controlled trial, fifty-two patients with AIS (10-18 years, 37 girls and 15 boys) participated. They were arbitrarily allocated into two groups: experimental (19 female/8 male; aged 14.74 +/- 1.79 years; Cobb angle 18.59 +/- 2.66 degrees) and control (18 female/7 male; aged 15.04 +/- 1.81 years; Cobb angle 19.32 +/- 2.69 degrees) groups. Both groups received Schroth exercises for 10 weeks, three days/week. The experimental group additionally received hippotherapy training. Pre-treatment and post-treatment assessment for the scoliotic, kyphotic angle, pelvic obliquity, pelvic torsion and vertical spinal rotation and the anteroposterior, mediolateral and overall stability indices were assessed using the formetric system 4D and Biodex Balance System, respectively. Results: After intervention, both groups illustrated significant improvements in all examined variables ($p < 0.05$). The experimental group illustrated significant improvements in scoliotic angle, kyphotic angle, pelvic obliquity, pelvic torsion and vertical spinal rotation and the stability indices compared to the control group ($p < 0.05$). Conclusion: In adolescence idiopathic scoliosis, hippotherapy training combined with Schroth exercises improves posture asymmetry and balancing ability more effectively than Schroth exercises alone.

C1 [Abdel-aziem, Amr A.] Taif Univ, Dept Phys Therapy, Coll Appl Med Sci, POB 11099, At Taif 21944, Saudi Arabia.

[Abdelraouf, Osama R.; Radwan, Rafik E.] Cairo Univ, Fac Phys Therapy, Dept Biomech, Giza 12613, Egypt.

[Ghally, Shahesta A.] October 6 Univ, Fac Phys Therapy, Dept Musculoskeletal Disorders & Its Surg, Giza 12585, Egypt.

[Dahlawi, Haytham A.] Taif Univ, Coll Appl Med Sci, Clin Lab Sci Dept, POB 11099, At Taif 21944, Saudi Arabia.

C3 Taif University; Egyptian Knowledge Bank (EKB); Cairo University;

Egyptian Knowledge Bank (EKB); October 6 University (O6U); Taif

University

RP Abdelraouf, OR (通讯作者)， Cairo Univ, Fac Phys Therapy, Dept Biomech, Giza 12613, Egypt.

EM amralmaz@tu.edu.sa; osamaibrahim2006@yahoo.com;

Shahesta.Ahmed.PT@o6u.edu.eg; haytham.d@tu.edu.sa;

rafik_radwan@hotmail.com

RI Abdel-aziem, amr/GQA-5184-2022; Abdelraouf, Osama Ragaa/AGJ-0828-2022;

Abdel-aziem, Amr A./J-2817-2019

OI Abdelraouf, Osama Ragaa/0000-0001-6888-5480; Abdel-aziem, Amr

A./0000-0001-8448-9218; Ahmed osama, Shahesta/0000-0002-6120-4168;

Radwan, Rafik/0000-0001-5405-853X

FU Taif University Researchers supporting project number [TURSP-2020/291];

Taif University, Taif, Saudi Arabia

FX FundingThe author(s) disclosed receipt of the following financial

support for the research, authorship, and/or publication of this

article: This work was supported by Taif University Researchers

supporting project number [TURSP-2020/291], Taif University, Taif, Saudi

Arabia.

CR Allard P, 2004, AM J PHYS MED REHAB, V83, P689, DOI 10.1097/01.PHM.0000137344.95784.15

Aranda-Garcia S, 2015, J AGING PHYS ACTIV, V23, P78, DOI [10.1123/japa.2012-0326, 10.1123/JAPA.2012-0326]

Araujo TB, 2011, BRAZ J PHYS THER, V15, P414, DOI 10.1590/S1413-35552011005000027

Beaulieu M, 2009, EUR SPINE J, V18, P38, DOI 10.1007/s00586-008-0831-6

Behm DG, 2010, APPL PHYSIOL NUTR ME, V35, P91, DOI 10.1139/H09-127

Beinotti F, 2010, ARQ NEURO-PSIQUIAT, V68, P908, DOI 10.1590/S0004-282X2010000600015

Benda W, 2003, J ALTERN COMPLEM MED, V9, P817, DOI 10.1089/107555303771952163

BIERY MJ, 1989, ADAPTED PHYSICAL ACT, V6, P221, DOI DOI 10.1123/apaq.6.3.221

Bryant PR, 2004, ARCH PHYS MED REHAB, V85, pS21, DOI 10.1053/j.apmr.2003.12.007

Canavese F, 2020, ANN TRANSL MED, V8, DOI 10.21037/atm.2019.12.160

Canavese F, 2019, SURG RADIOL ANAT, V41, P287, DOI 10.1007/s00276-018-2164-4

Chen PQ, 1998, CLIN BIOMECH, V13, pS52, DOI 10.1016/S0268-0033(97)00075-2

Dalleau G, 2007, EUR SPINE J, V16, P1593, DOI 10.1007/s00586-007-0404-0

Dankerl P, 2016, PROSTHET ORTHOT INT, V40, P369, DOI 10.1177/0309364614554031

de Loeze MP, 1999, J BIOMECH, V32, P655, DOI 10.1016/S0021-9290(99)00061-5

Di Maria F, 2021, HEALTHCARE-BASEL, V9, DOI 10.3390/healthcare9101372

DRISCOLL DM, 1984, J PEDIATR ORTHOPED, V4, P677, DOI 10.1097/01241398-198411000-00004

El-Basatiny HMY, 2015, CLIN REHABIL, V29, P457, DOI 10.1177/0269215514547654

Funakoshi R, 2018, HELIYON, V4, DOI 10.1016/j.heliyon.2018.e00777

Garner BA, 2015, HUM MOVEMENT SCI, V39, P121, DOI 10.1016/j.humov.2014.06.011

Gauchard GC, 2001, SPINE, V26, P1052, DOI 10.1097/00007632-200105010-00014

Goldmann T, 2012, Comput Methods Biomech Biomed Engin, V15 Suppl 1, P203

GREGORIC M, 1981, ACTA ORTHOP SCAND, V52, P59, DOI 10.3109/17453678108991759

Guo X, 2003, J BONE JOINT SURG BR, V85B, P1026, DOI 10.1302/0301-620X.85B7.14046

Guo X, 2006, SPINE, V31, pE437, DOI 10.1097/01.brs.0000222048.47010.bf

Handzel T.M, 2014, NSCAS PERFORM TRAIN, V2, P26

Haumont T, 2011, SPINE, V36, pE847, DOI 10.1097/BRS.0b013e3181ff5837

Hibbs AE, 2011, J ELECTROMYOGR KINES, V21, P102, DOI 10.1016/j.jelekin.2010.06.001

Hilliere C, 2018, PM&R, V10, P1062, DOI 10.1016/j.pmrj.2018.03.019

Homnick DN, 2013, J ALTERN COMPLEM MED, V19, P622, DOI 10.1089/acm.2012.0642

Houghton KM, 2013, PEDIATR PHYS THER, V25, P150, DOI 10.1097/PEP.0b013e31828a2978

Janura M, 2015, NEUROENDOCRINOL LETT, V36, P481

Kang KY, 2015, J PHYS THER SCI, V27, P2499, DOI 10.1589/jpts.27.2499

Katsaris G, 1999, EUR SPINE J, V8, P2, DOI 10.1007/s005860050119

Kawashima H, 2012, PEDIATR INT, V54, DOI 10.1111/j.1442-200X.2011.03456.x

Kim KH, 2015, J PHYS THER SCI, V27, P1125, DOI 10.1589/jpts.27.1125

Kim MJ, 2018, PERCEPT MOTOR SKILL, V125, P93, DOI 10.1177/0031512517736463

Kouwenhoven JWM, 2008, SPINE, V33, P2898, DOI 10.1097/BRS.0b013e3181891751

Kuo FC, 2010, SPINE, V35, P2084, DOI 10.1097/BRS.0b013e3181cc8108

Kuru T, 2016, CLIN REHABIL, V30, P181, DOI 10.1177/0269215515575745

Lee DR, 2011, NEUROREHABILITATION, V29, P23, DOI 10.3233/NRE-2011-0673

McGibbon NH, 2009, ARCH PHYS MED REHAB, V90, P966, DOI 10.1016/j.apmr.2009.01.011

Munoz-Lasa S., 2012, G ITAL MED LAV ERGON, V33, P462

Nault ML, 2002, SPINE, V27, P1911, DOI 10.1097/00007632-200209010-00018

Negrini S, 2018, SCOLIOSIS SPINAL DIS, V13, DOI 10.1186/s13013-017-0145-8

O'Beirne J, 1989, J Spinal Disord, V2, P184

Penha PJ, 2018, SPINE, V43, P1710, DOI 10.1097/BRS.0000000000002725

Rahbar M, 2018, J BACK MUSCULOSKELET, V31, P1183, DOI 10.3233/BMR-170832

Richards BS, 2005, SPINE, V30, P2068, DOI 10.1097/01.brs.0000178819.90239.d0

Sahin F, 2019, TURK J PH MED REHAB, V65, P236, DOI 10.5606/tftrd.2019.2825

SAHLSTRAND T, 1978, ACTA ORTHOP SCAND, V49, P354, DOI 10.3109/17453677809050088

Schreiber S, 2019, BMC MUSCULOSKEL DIS, V20, DOI 10.1186/s12891-019-2695-9

Schreiber S, 2016, PLOS ONE, V11, DOI 10.1371/journal.pone.0168746

Schreiber Sanja, 2014, J Physiother, V60, P234, DOI 10.1016/j.jphys.2014.08.005

Shurtleff TL, 2009, ARCH PHYS MED REHAB, V90, P1185, DOI 10.1016/j.apmr.2009.01.026

Simoneau M, 2006, EXP BRAIN RES, V170, P576, DOI 10.1007/s00221-005-0246-0

Simoneau M, 2006, BMC NEUROSCI, V7, DOI 10.1186/1471-2202-7-68

Stergiou A, 2017, AM J PHYS MED REHAB, V96, P724, DOI 10.1097/PHM.0000000000000726

Tabard-Fougere A, 2017, SPINE, V42, P98, DOI 10.1097/BRS.0000000000001679

Terada K., 2004, Equine and Comparative Exercise Physiology, V1, P193

Weiss HR, 2011, SCOLIOSIS SPINAL DIS, V6, DOI 10.1186/1748-7161-6-17

Yagci G, 2019, PROSTHET ORTHOT INT, V43, P434, DOI 10.1177/0309364619839856

Yilmaz H, 2020, SPINE J, V20, P947, DOI 10.1016/j.spinee.2020.01.008

Yoo JH, 2014, INT J CLIN PRACT, V68, P941, DOI 10.1111/ijcp.12414

ZETTERBERG C, 1984, ACTA ORTHOP SCAND, V55, P304, DOI 10.3109/17453678408992362

NR 65

TC 3

Z9 3

U1 5

U2 29

PU MDPI

PI BASEL

PA ST ALBAN-ANLAGE 66, CH-4052 BASEL, SWITZERLAND

EI 2227-9067

J9 CHILDREN-BASEL

JI Children-Basel

PD JAN

PY 2022

VL 9

IS 1

AR 23

DI 10.3390/children9010023

PG 11

WC Pediatrics

WE Science Citation Index Expanded (SCI-EXPANDED); Social Science Citation Index (SSCI)

SC Pediatrics

GA YN9GM

UT WOS:000747559000001

PM 35053648

OA gold, Green Published

DA 2023-08-10

ER

PT J

AU Korkmaz, MD

Korkmaz, M

Capan, N

Sanli, G

Tatar, Y

Aydin, AR

AF Korkmaz, Merve Damla

Korkmaz, Murat

Capan, Nalan

Sanli, Goktug

Tatar, Yasar

Aydin, Ayse Resa

TI Seating system for scoliosis in nonambulatory children with cerebral

palsy: a randomized controlled trial

SO REVISTA DA ASSOCIACAO MEDICA BRASILEIRA

LA English

DT Article

DE Cerebral palsy; Sitting; Scoliosis; Spine; Pelvis; Sitting; Scoliosis;

Spine; Pelvis

ID SPINOPELVIC PARAMETERS; PELVIC INCIDENCE; MANAGEMENT; CLASSIFICATION

AB OBJECTIVE: This study aimed to investigate the effect of an adaptive seating system on pelvic obliquity and spinal coronal/sagittal balance in children with nonambulatory cerebral palsy and scoliosis. METHODS: This was a single-blind, prospective, randomized interventional study. Nonambulatory children aged 6???15 years with cerebral palsy and scoliosis were included. The seating system was used for 4 h/day, and exercises were performed 3 days/week for 12 weeks. The Cobb angle, spinopelvic parameters, pelvic obliquity, Reimer???s migration index, and Sitting Assessment Scale were measured before and after treatments. RESULTS: A total of 29 participants were randomized into two groups, namely, the seating system+exercise group (SSE-group; n=15) and the exercise group (E-group; n=14). There was no significant change in Cobb angle and Reimer???s migration index for both hips in SSE-group, but there was a significant increase in E-group (p=0.002, 0.049, and 0.003, respectively). The sagittal vertical axis, pelvic incidence, and pelvic obliquity decreased in SSE-group. However, there was no difference in the other sagittal parameters and Sitting Assessment Scale-total scores among groups. CONCLUSION: The adaptive seating system was found to be superior in reducing the progression of Cobb angle and hip subluxation/dislocation, decreasing pelvic obliquity, and improving the sagittal balance of the spine/pelvis compared with exercise therapy.

C1 [Korkmaz, Merve Damla] Univ Hlth Sci, Kanuni Sultan Suleyman Training & Res Hosp, Dept Phys Med & Rehabil, Istanbul, Turkey.

[Korkmaz, Murat] Istanbul Univ, Istanbul Fac Med, Dept Orthoped & Traumatol, Istanbul, Turkey.

[Capan, Nalan; Aydin, Ayse Resa] Istanbul Univ, Istanbul Fac Med, Dept Phys Med & Rehabil, Istanbul, Turkey.

[Sanli, Goktug] Marmara Univ, Fac Sports Sci, Istanbul, Turkey.

[Tatar, Yasar] Marmara Univ, Fac Med, Dept Phys Med & Rehabil, Istanbul, Turkey.

C3 Istanbul Kanuni Sultan Suleyman Training & Research Hospital; University
of Health Sciences Turkey; Istanbul University; Istanbul University;
Marmara University; Marmara University

RP Korkmaz, MD (通讯作者)， Univ Hlth Sci, Kanuni Sultan Suleyman Training & Res Hosp, Dept Phys Med & Rehabil, Istanbul, Turkey.

EM mervedml@gmail.com

RI Korkmaz, Merve Damla/ADK-9971-2022

OI Korkmaz, Merve Damla/0000-0003-2422-5709; Capan,
Nalan/0000-0002-9185-1809; Tatar, Yasar/0000-0001-6815-301X; Sanli,
Goktug/0000-0002-6524-9128

CR Angsupaisal M, 2015, DEV MED CHILD NEUROL, V57, P919, DOI 10.1111/dmcn.12762

Brooks JT, 2016, J PEDIATR ORTHOPED, V36, P627, DOI 10.1097/BPO.0000000000000497

Celestre PC, 2018, NEUROSURG CLIN N AM, V29, P323, DOI 10.1016/j.nec.2018.03.003

Cimolin V, 2009, DISABIL REHABIL-ASSI, V4, P422, DOI 10.3109/17483100903254553

Hayden AM, 2018, SPINE J, V18, P173, DOI 10.1016/j.spinee.2017.08.234

Holmes KJ, 2003, CLIN BIOMECH, V18, P480, DOI 10.1016/S0268-0033(03)00075-5

Kim D, 2021, STATPEARLS

Kim IS, 2019, ARCH PHYS MED REHAB, V100, P247, DOI 10.1016/j.apmr.2018.07.423

Koop SE, 2009, DEV MED CHILD NEUROL, V51, P92, DOI 10.1111/j.1469-8749.2009.03461.x

Le Huec JC, 2019, EUR SPINE J, V28, P1889, DOI 10.1007/s00586-019-06083-1

Mcdonald RL, 2007, DISABIL REHABIL, V29, P1041, DOI 10.1080/09638280600943087

Murphy RF, 2019, CURR REV MUSCULOSKE, V12, P220, DOI 10.1007/s12178-019-09552-8

Myhr U., 1993, MANUAL SITTING ASSES

Niedzwecki C.M., 2021, BRADDOMS PHYS MED RE

Novak I, 2017, JAMA PEDIATR, V171, P897, DOI 10.1001/jamapediatrics.2017.1689

Palisano RJ, 2008, DEV MED CHILD NEUROL, V50, P744, DOI 10.1111/j.1469-8749.2008.03089.x

Place HM, 2017, SPINE J, V17, P1565, DOI 10.1016/j.spinee.2017.06.037

Pountney T, 2002, CHILD CARE HLTH DEV, V28, P179, DOI 10.1046/j.1365-2214.2002.00254.x

Schroeder N., 2018, SPINE DEFORM, V6, P753, DOI [10.1016/j.jspd.2018.03.008, DOI 10.1016/J.JSPD.2018.03.008]

Suh SW, 2013, SPINE J, V13, P882, DOI 10.1016/j.spinee.2013.02.011

Toovey R, 2017, DEV MED CHILD NEUROL, V59, P690, DOI 10.1111/dmcn.13412

Vargus-Adams J, 2020, PEDIAT REHABILITATIO, V6th, P319

Vekerdy Z, 2007, DISABIL REHABIL, V29, P1434, DOI 10.1080/09638280601055691

Vialle R, 2013, ORTHOP TRAUMATOL-SUR, V99, pS124, DOI 10.1016/j.otsr.2012.11.002

Weigl DM, 2019, ISR MED ASSOC J, V21, P752

NR 25

TC 0

Z9 0

U1 2

U2 2

PU ASSOC MEDICA BRASILEIRA

PI SAO PAULO

PA RUA SAO CARLOS DO PINHAL 324, CAIXA POSTAL 8904, SAO PAULO, SP, BRAZIL

EI 1806-9282

J9 REV ASSOC MED BRAS

JI Rev. Assoc. Med. Bras.

PY 2022

VL 68

IS 5

BP 616

EP 621

DI 10.1590/1806-9282.20211260

PG 6

WC Medicine, General & Internal

WE Science Citation Index Expanded (SCI-EXPANDED)

SC General & Internal Medicine

GA 1N0PR

UT WOS:000800366600014

PM 35584484

OA gold

DA 2023-08-10

ER

PT J

AU Radwan, NL

Ibrahim, MM

Mahmoud, WS

AF Radwan, Nadia L.

Ibrahim, Marwa M.

Mahmoud, Waleed S.

TI Comparison of two periods of Schroth exercises for improving postural

stability indices and Cobb angle in adolescent idiopathic scoliosis

SO JOURNAL OF BACK AND MUSCULOSKELETAL REHABILITATION

LA English

DT Article

DE Adolescent idiopathic scoliosis; Biodex Balance System; postural

balance; Schroth exercises

ID DYNAMIC BALANCE; YOUNG-ADULTS; WEIGHT; BRACE

AB BACKGROUND: Adolescents with idiopathic scoliosis present postural instability when compared with healthy subjects. Although Schroth exercises therapy (SET) is broadly utilized, its effect on postural stability is still not clear.

OBJECTIVES: To compare the two treatment periods of the SET for improving the postural stability indices and Cobb angle, and to examine the correlation between the Cobb angle and stability indices in adolescent idiopathic scoliosis (AIS).

METHODS: Twenty girls aged 10-16 years with AIS (study group) and 20 age-matched girls without AIS (control group) were examined. The Biodex Balance System was used to evaluate the overall stability index (OSI), anteroposterior index (APSI), and mediolateral stability index (MLSI) in the study group before SET and one and three months after the therapy. A plain X-ray was used to measure the Cobb angle before and three months after SET. Stability indices and Cobb angle were measured only once for the control group.

RESULTS: One-way repeated-measures ANOVA revealed that the three-month duration of SET was the most effective for improving OSI, APSI, and MLSI ($p < 0.001$). The significant proximities of OSI, APSI, and MLSI to the normal values post three months of SET were 29.65%, 24.07, and 20% respectively. The MLSI was robust and correlated with the Cobb angle ($r = 0.85$) three months post intervention.

CONCLUSION: Stability indices and Cobb angles were highly improved after three months of SET compared to one month among AIS patients. The MLSI is the most substantial index correlated with the Cobb angle.

C1 [Radwan, Nadia L.] Cairo Univ, Fac Phys Therapy, Dept Biomech, Giza, Egypt.

[Ibrahim, Marwa M.] Cairo Univ, Fac Phys Therapy, Dept Phys Therapy Pediat, Giza, Egypt.

[Radwan, Nadia L.; Ibrahim, Marwa M.; Mahmoud, Waleed S.] Prince Sattam Bin Abdulaziz Univ, Coll Appl Med Sci, Dept Hlth & Rehabil Sci, Alkharj, Saudi Arabia.

[Mahmoud, Waleed S.] Cairo Univ, Fac Phys Therapy, Dept Basic Sci, Giza, Egypt.

C3 Egyptian Knowledge Bank (EKB); Cairo University; Egyptian Knowledge Bank

(EKB); Cairo University; Prince Sattam Bin Abdulaziz University;

Egyptian Knowledge Bank (EKB); Cairo University

RP Mahmoud, WS (通讯作者)， Prince Sattam Bin Abdulaziz Univ, Coll Appl Med Sci, Dept Phys Therapy & Hlth Rehabil, Riyadh, Alkharj, Saudi Arabia.

EM waleeds306@yahoo.com

RI Radwan, Nadia/GQQ-8419-2022

OI radwan, nadia/0000-0001-7324-6210; Ibrahim, Marwa/0000-0002-3665-3228

FU Deanship of Scientific Research at Prince Sattam Bin Abdulaziz
University, Alkharj, Saudi Arabia

FX This publication was supported by the Deanship of Scientific Research at
Prince Sattam Bin Abdulaziz University, Alkharj, Saudi Arabia. The
authors would like to thank all subjects who participated in this study
for their cooperation.

CR Allard P, 2004, AM J PHYS MED REHAB, V83, P689, DOI 10.1097/01.PHM.0000137344.95784.15

Beaulieu M, 2009, EUR SPINE J, V18, P38, DOI 10.1007/s00586-008-0831-6

Burger M, 2019, SOUTH AFR J PHYSIOTH, V75, DOI 10.4102/sajp.v75i1.904

Cachupe WJC., 2001, MEAS PHYS EDUC EXERC, V5, P97, DOI [DOI 10.1207/S15327841MPEE0502_3, 10.1207/S15327841MPEE0502_3]

Chow DHK, 2006, GAIT POSTURE, V24, P173, DOI 10.1016/j.gaitpost.2005.08.007

DRISCOLL DM, 1984, J PEDIATR ORTHOPED, V4, P677, DOI 10.1097/01241398-198411000-00004

Dupuis S, 2018, BMC MUSCULOSKEL DIS, V19, DOI 10.1186/s12891-018-2112-9

Gheitasi M, 2019, PHYS TREATMENTS, V9, P59, DOI [10.32598/PTJ.9.1.59, DOI 10.32598/PTJ.9.1.59]

Guo X, 2006, SPINE, V31, pE437, DOI 10.1097/01.brs.0000222048.47010.bf

Haumont T, 2011, SPINE, V36, pE847, DOI 10.1097/BRS.0b013e3181ff5837

Hawes MC, 2006, SCOLIOSIS SPINAL DIS, V1, DOI 10.1186/1748-7161-1-3

Horne JP, 2014, AM FAM PHYSICIAN, V89, P193

Jevtic N, 2014, EXERC QUAL LIFE, V6, P23

Jorgic B, 2018, PHYS ED SPORT, V16, P749

Karimi N, 2008, PAK J MED SCI, V24, P372

Kim G, 2016, J PHYS THER SCI, V28, P1012, DOI 10.1589/jpts.28.1012

Kuo FC, 2010, SPINE, V35, P2084, DOI 10.1097/BRS.0b013e3181cc8108

Kuru T, 2016, CLIN REHABIL, V30, P181, DOI 10.1177/0269215515575745

Langensiepen S, 2013, EUR SPINE J, V22, P2360, DOI 10.1007/s00586-013-2693-9

Lao MLM, 2008, J PEDIATR ORTHOPED, V28, P846, DOI 10.1097/BPO.0b013e31818e1bc9

Lee Hyung Joo, 2016, J Phys Ther Sci, V28, P2975

Lenhert-Schroth C., 2007, SCHROTH SCOLIOSIS 3

Liang JP, 2018, TRIALS, V19, DOI 10.1186/s13063-018-2834-x

Nault ML, 2002, SPINE, V27, P1911, DOI 10.1097/00007632-200209010-00018

Negrini S, 2008, J REHABIL MED, V40, P451, DOI 10.2340/16501977-0195

Negrini S, 2012, SCOLIOSIS SPINAL DIS, V7, DOI 10.1186/1748-7161-7-3

O'Beirne J, 1989, J Spinal Disord, V2, P184

Otman Saadet, 2005, Neurosciences (Riyadh), V10, P277

Paolucci T, 2013, EUR J PHYS REHAB MED, V49, P649

Parent EC, 2010, SPINE, V35, P315, DOI 10.1097/BRS.0b013e3181cabe75

Park JH, 2018, EUR J PHYS REHAB MED, V54, P440, DOI 10.23736/S1973-9087.17.04461-6

Pialasse JP, 2016, J MANIP PHYSIOL THER, V39, P473, DOI 10.1016/j.jmpt.2016.06.001

Rigo M, 2003, Pediatr Rehabil, V6, P209

Romano M, 2013, SPINE, V38, pE883, DOI 10.1097/BRS.0b013e31829459f8

Sahin F, 2019, TURK J PH MED REHAB, V65, P236, DOI 10.5606/tftrd.2019.2825

Schimmel JJP, 2015, SCOLIOSIS SPINAL DIS, V10, DOI 10.1186/s13013-015-0042-y

Schreiber S, 2019, BMC MUSCULOSKEL DIS, V20, DOI 10.1186/s12891-019-2695-9

Schreiber S, 2016, PLOS ONE, V11, DOI 10.1371/journal.pone.0168746

Strukcinskait e. V, 2017, BIOMEDICINE, V27, P71

Tolo VT, 2020, SPINE DEFORM, V8, P149, DOI 10.1007/s43390-020-00036-1

Watters H., 2012, J NOV PHYSIOTHER, V2, P113

Weinstein SL, 2008, LANCET, V371, P1527, DOI 10.1016/S0140-6736(08)60658-3

Yagci G, 2018, PHYSIOTHER THEOR PR, V34, P579, DOI 10.1080/09593985.2017.1423429

NR 43

TC 0

Z9 0

U1 4

U2 11

PU IOS PRESS

PI AMSTERDAM

PA NIEUWE HEMWEG 6B, 1013 BG AMSTERDAM, NETHERLANDS

SN 1053-8127

EI 1878-6324

J9 J BACK MUSCULOSKELET

JI J. Back Musculoskelet. Rehabil.

PY 2022

VL 35

IS 3

BP 573

EP 582

DI 10.3233/BMR-200342

PG 10

WC Orthopedics; Rehabilitation

WE Science Citation Index Expanded (SCI-EXPANDED)

SC Orthopedics; Rehabilitation

GA 1R9LZ

UT WOS:000803684600014

PM 34602457

DA 2023-08-10

ER

PT J

AU Yagci, G

Ozcan, DA

Kuru, CA

Yakut, Y

AF Yagci, Gozde

Ozcan, Damlagul Aydin

Kuru, Cigdem Ayhan

Yakut, Yavuz

TI Kinesiophobia and its association with treatment choice in individuals

with idiopathic scoliosis

SO JOURNAL OF BACK AND MUSCULOSKELETAL REHABILITATION

LA English

DT Article

DE Scoliosis; fear; movement; exercise therapy; brace

ID TAMPA SCALE; LOW-BACK; MUSCULOSKELETAL PAIN; FEAR; REHABILITATION;

MOVEMENT; VERSION

AB BACKGROUND: Previous studies have reported increased kinesiophobia in adults with degenerative scoliosis or adolescents who have undergone spinal surgery. However, little is known about the phenomenon of kinesiophobia among adolescents with idiopathic scoliosis (IS).

OBJECTIVES: The aim of this study was to investigate levels of kinesiophobia and its association with treatment choice in IS.

METHODS: The study included 98 IS patients with a mean age of 14 years and 20 healthy controls. Participants with IS were divided into groups based on treatment conditions, as follows: (1) untreated (n = 33); (2) treated with exercise (n = 32); and (3) treated with a brace (n = 33). Kinesiophobia was measured using the Tampa Scale for Kinesiophobia (TSK). Comparisons were made between four groups.

RESULTS: Untreated participants with IS were found to have greater kinesiophobia than healthy controls ($p < 0.001$). Participants treated with a brace ($p = 0.046$) and exercise ($p = 0.064$) had similar kinesiophobia levels as the healthy control group.

CONCLUSIONS: Kinesiophobia was found to be higher in adolescents with IS compared to healthy peers. Brace or exercise treatment both had a positive impact on kinesiophobia. These findings should be considered when organizing rehabilitation programs to achieve the best results for adolescents with IS.

C1 [Yagci, Gozde; Ozcan, Damlagul Aydin; Kuru, Cigdem Ayhan] Hacettepe Univ, Fac Phys Therapy & Rehabil, TR-06100 Ankara, Turkey.

[Yakut, Yavuz] Hasan Kalyoncu Univ, Fac Hlth Sci, Sch Phys Therapy & Rehabil, Gaziantep, Turkey.

C3 Hacettepe University; Hasan Kalyoncu University

RP Yagci, G (通讯作者)，Hacettepe Univ, Fac Phys Therapy & Rehabil, TR-06100 Ankara, Turkey.

EM gozdeygc8@gmail.com

RI Aydin Özcan, Damlagül/HZL-3555-2023; YAGCI, GOZDE/J-1185-2013; KURU,

CIGDEM AYHAN/J-1183-2013

OI YAGCI, GOZDE/0000-0002-4603-7162; KURU, CIGDEM AYHAN/0000-0002-4555-4563

CR [Anonymous], 1990, PAIN MANAG

Archer KR, 2012, ARCH PHYS MED REHAB, V93, P1460, DOI 10.1016/j.apmr.2012.03.024

Branstrom H, 2008, J REHABIL MED, V40, P375, DOI 10.2340/16501977-0186

Carrasco MIB, 2016, TEXTO CONTEXTO ENFER, V25

Chopra S, 2020, PLOS ONE, V15, DOI 10.1371/journal.pone.0238181

Coelho DM, 2013, BRAZ J PHYS THER, V17, P179, DOI 10.1590/S1413-35552012005000081

Day JM, 2019, ARCH PHYSIOTHERAPY, V9, P1

El Hawary R, 2019, SPINE J, V19, P1917, DOI 10.1016/j.spinee.2019.07.008

Falk B, 2015, SPINE J, V15, P1169, DOI 10.1016/j.spinee.2014.05.006

Flanigan DC, 2015, J AM ACAD ORTHOP SUR, V23, P563, DOI 10.5435/JAAOS-D-14-00225

Gur G, 2015, GAIT POSTURE, V41, P93, DOI 10.1016/j.gaitpost.2014.09.001

Haddas R, 2018, SPINE, V43, P647, DOI 10.1097/BRS.0000000000002381

Ilves O, 2017, J REHABIL MED, P49

Kernan T, 2007, J ORTHOP SPORT PHYS, V37, P679, DOI 10.2519/jospt.2007.2480

Lee WTK, 2005, OSTEOPOROSIS INT, V16, P1024, DOI 10.1007/s00198-004-1792-1

Leeuw M, 2007, J BEHAV MED, V30, P77, DOI 10.1007/s10865-006-9085-0

Liu DL, 2020, SPINE, V45, P1039, DOI 10.1097/BRS.0000000000003451

Neblett R, 2016, EUR J PAIN, V20, P701, DOI 10.1002/ejp.795

Negrini A, 2016, SCOLIOSIS SPINAL DIS, V11, DOI 10.1186/s13013-016-0100-0

O'Brien EM, 2008, EUR J PAIN, V12, P104, DOI 10.1016/j.ejpain.2007.04.001

Page MG, 2013, J PAIN RES, V6, P167, DOI 10.2147/JPR.S40846

Peterson GE, 2015, J MANIP PHYSIOL THER, V38, P465, DOI 10.1016/j.jmpt.2015.06.011

Roach JW, 1999, ORTHOP CLIN N AM, V30, P353, DOI 10.1016/S0030-5898(05)70092-4

Saraiva Bruna Marques de Almeida, 2017, Fisioter. mov., V30, P209, DOI 10.1590/1980-5918.030.s01.ao20

Tanure MC, 2010, SPINE J, V10, P769, DOI 10.1016/j.spinee.2010.02.020

Tobias JH, 2019, INT J EPIDEMIOL, V48, P1152, DOI 10.1093/ije/dyy268

Todd DD., 1991, CLIN J PAIN, V39, P51, DOI [DOI 10.1097/00002508-199103000-00053, 10.1097/00002508-199103000-00053]

Tunca Yilmaz O, 2011, FIZYOTERAPI REHABILI, V22, P44

Ulug N, 2016, J PHYS THER SCI, V28, P665, DOI 10.1589/jpts.28.665

VLAEYEN JWS, 1995, PAIN, V62, P363, DOI 10.1016/0304-3959(94)00279-N

Woby SR, 2005, PAIN, V117, P137, DOI 10.1016/j.pain.2005.05.029

Ye D, 2018, J PAIN, V19, pS40

Ye DL, 2020, PHYS OCCUP THER PEDI, V40, P546, DOI 10.1080/01942638.2020.1720054

Zelle DM, 2016, PLOS ONE, V11, DOI 10.1371/journal.pone.0147609

NR 34

TC 0

Z9 0

U1 1

U2 5

PU IOS PRESS

PI AMSTERDAM

PA NIEUWE HEMWEG 6B, 1013 BG AMSTERDAM, NETHERLANDS

SN 1053-8127

EI 1878-6324

J9 J BACK MUSCULOSKELET

JI J. Back Musculoskelet. Rehabil.

PY 2022

VL 35

IS 4

BP 881

EP 891

DI 10.3233/BMR-210111

PG 11

WC Orthopedics; Rehabilitation

WE Science Citation Index Expanded (SCI-EXPANDED)

SC Orthopedics; Rehabilitation

GA 2Z0MZ

UT WOS:000826280800023

PM 34744071

DA 2023-08-10

ER

PT J

AU Gamiz-Bermudez, F

Obrero-Gaitan, E

Zagalaz-Anula, N

Lomas-Vega, R

AF Gamiz-Bermudez, Francisca

Obrero-Gaitan, Esteban

Zagalaz-Anula, Noelia

Lomas-Vega, Rafael

TI Corrective exercise-based therapy for adolescent idiopathic scoliosis:

Systematic review and meta-analysis

SO CLINICAL REHABILITATION

LA English

DT Review

DE scoliosis; exercise therapy; exercise movement techniques; meta-analysis

ID QUALITY-OF-LIFE; SCHROTH; BIAS; QUESTIONNAIRE

AB Objective to analyze the efficacy of Corrective exercise-based therapy in the improvement of deformity and quality of life in adolescent idiopathic scoliosis. Data sources PubMed Medline, Scopus, Web of Science (WOS), Physiotherapy Evidence Database, CINAHL Complete and SciELO, until June 2021. Review methods Randomized controlled trials was selected, including participants diagnosed with adolescent idiopathic scoliosis, in which the experimental group received Corrective exercise-based therapy. Two authors independently searched the scientific literature in the data sources, extracted the data and assessed the risk of bias. A pairwise meta-analysis using the random-effects model was performed. Results Eight randomized controlled trials providing data from 279 adolescent idiopathic scoliosis patients were included. Seven randomized controlled trials including 236 patients showed moderate-quality evidence for a medium effect (SMD = -0.52, 95% CI

-0.96 to -0.1), favoring corrective exercise-based therapy for spinal deformity reduction. Corrective exercise-based therapy was better than no intervention (SMD = -0.59, 95% CI -1.18 to -0.01) but similar to other intervention (SMD = -0.2, 95% CI -0.67 to 0.27), and a medium effect was found (SMD = -0.51, 95% CI -0.89 to -0.13) when corrective exercise-based therapy was used with other therapies. Four studies including 151 patients showed low-quality evidence of a large effect of Corrective exercise-based therapy on Scoliosis Research Society measurement (SRS-22) total score improvement (SMD = 1.16, 95% CI 0.36 to 1.95). Conclusion In mild and moderate adolescent idiopathic scoliosis patients, corrective exercise-based therapy could be used to reduce spinal deformity and to improve quality of life as isolated treatment or as coadjuvant treatment combined with other therapeutic resources.

C1 [Gamiz-Bermudez, Francisca] Andalusian Hlth Serv, Poniente Almeria Hlth Dist, Adra, Spain.

[Obrero-Gaitan, Esteban; Zagalaz-Anula, Noelia; Lomas-Vega, Rafael] Univ Jaen, Dept Hlth Sci, Bldg B3,Off 214,Campus Lagunillas S-N, Jaen 23071, Spain.

C3 Universidad de Jaen

RP Zagalaz-Anula, N (通讯作者)， Univ Jaen, Dept Hlth Sci, Bldg B3,Off 214,Campus Lagunillas S-N, Jaen 23071, Spain.

EM nzagalaz@ujaen.es

RI Obrero-Gaitán, Esteban/HPF-9474-2023; Zagalaz-Anula,

Noelia/AAE-9027-2021

OI Zagalaz-Anula, Noelia/0000-0002-7105-9865; Obrero-Gaitan,

Esteban/0000-0002-8430-5500

CR [Anonymous], 2005, ZHONGGUO LINCHUANG K

Anwer S, 2015, BIOMED RES INT-UK, V2015, DOI 10.1155/2015/123848

Asher MA, 2006, SPINE, V31, P593, DOI 10.1097/01.brs.0000201331.50597.ea

Berdishevsky H, 2016, SCOLIOSIS SPINAL DIS, V11, DOI 10.1186/s13013-016-0076-9

Borenstein ML., COMPREHENSIVE META A

Burger M, 2019, SOUTH AFR J PHYSIOTH, V75, DOI 10.4102/sajp.v75i1.904

Coelho DM, 2013, BRAZ J PHYS THER, V17, P179, DOI 10.1590/S1413-35552012005000081

Cohen J., 2013, STAT POWER ANAL BEHA, V2nd

Delamothe T, 2004, BMJ-BRIT MED J, V328, P1, DOI 10.1136/bmj.328.7430.1

Diab AA, 2012, CLIN REHABIL, V26, P1123, DOI 10.1177/0269215512447085

DICKSON RA, 1978, ACTA ORTHOP SCAND, V49, P46, DOI 10.3109/17453677809005722

Duval S, 2000, BIOMETRICS, V56, P455, DOI 10.1111/j.0006-341X.2000.00455.x

Egger M, 1997, BMJ-BRIT MED J, V315, P629, DOI 10.1136/bmj.315.7109.629

Faraone Stephen V, 2008, P T, V33, P700

Gao CF, 2019, AM J PHYS MED REHAB, V98, P642, DOI 10.1097/PHM.0000000000001160

Glattes RC, 2007, SPINE, V32, P1778, DOI 10.1097/BRS.0b013e3180dc9bb2

Gur G, 2017, PROSTHET ORTHOT INT, V41, P303, DOI 10.1177/0309364616664151

Higgins Julian P T, 2011, BMJ, V343, pd5928, DOI 10.1136/bmj.d5928

Hozo S.P., 2005, BMC MED RES METHODOL, V5, DOI DOI 10.1186/1471-2288-5-13

Kim G, 2016, J PHYS THER SCI, V28, P1012, DOI 10.1589/jpts.28.1012

Kuru T, 2016, CLIN REHABIL, V30, P181, DOI 10.1177/0269215515575745

Li X, 2021, CLIN REHABIL, V35, P669, DOI 10.1177/0269215520975105

Macdonald G, 2012, COCHRANE DB SYST REV, DOI 10.1002/14651858.CD001930.pub3

Meng ZD, 2017, MEDICINE, V96, DOI 10.1097/MD.0000000000006828

Moher D, 2015, SYST REV-LONDON, V4, DOI [10.1136/bmj.b2535, 10.1186/s13643-015-0087-2, 10.1016/j.ijsu.2010.02.007, 10.1016/j.ijsu.2010.07.299]

Monticone M, 2014, EUR SPINE J, V23, P1204, DOI 10.1007/s00586-014-3241-y

Negrini A, 2016, SCOLIOSIS SPINAL DIS, V11, DOI 10.1186/s13013-016-0100-0

Negrini S, 2012, SCOLIOSIS SPINAL DIS, V7, DOI 10.1186/1748-7161-7-3

Obrero-Gaitan E., 2020, PROSPEROCRD420201971

Park JH, 2018, EUR J PHYS REHAB MED, V54, P440, DOI 10.23736/S1973-9087.17.04461-6

Rothman K., 2008, MODERN EPIDEMIOLOGY

Rucker G, 2021, RES SYNTH METHODS, V12, P13, DOI 10.1002/jrsm.1410

Schreiber S, 2016, PLOS ONE, V11, DOI 10.1371/journal.pone.0168746

Schreiber S, 2015, SCOLIOSIS SPINAL DIS, V10, DOI 10.1186/s13013-015-0048-5

Sterne JAC, 2001, J CLIN EPIDEMIOL, V54, P1046, DOI 10.1016/S0895-4356(01)00377-8

Thompson JY, 2019, PHYSIOTHERAPY, V105, P214, DOI 10.1016/j.physio.2018.10.004

Toledo Pollyana Coelho Vieira, 2011, Fisioter. Pesqui., V18, P329

Valentine, 2019, HDB RES SYNTHESIS ME

Zhang YH, 2019, EUR SPINE J, V28, P2012, DOI 10.1007/s00586-019-06075-1

Zheng Y, 2018, SPINE, V43, pE494, DOI 10.1097/BRS.0000000000002412

NR 40

TC 2

Z9 3

U1 6

U2 29

PU SAGE PUBLICATIONS LTD

PI LONDON

PA 1 OLIVERS YARD, 55 CITY ROAD, LONDON EC1Y 1SP, united kingdom

SN 0269-2155

EI 1477-0873

J9 CLIN REHABIL

JI Clin. Rehabil.

PD MAY

PY 2022

VL 36

IS 5

BP 597

EP 608

AR 02692155211070452

DI 10.1177/02692155211070452

EA DEC 2021

PG 12

WC Rehabilitation

WE Science Citation Index Expanded (SCI-EXPANDED)

SC Rehabilitation

GA ZX6KD

UT WOS:000737708300001

PM 34962437

DA 2023-08-10

ER

PT J

AU Gao, A

Li, JY

Shao, R

Wu, TX

Wang, YQ

Liu, XG

Yu, M

AF Gao, Ang

Li, Jun-Yu

Shao, Rui

Wu, Tong-Xuan

Wang, Yong-Qiang

Liu, Xiao-Guang

Yu, Miao

TI Schroth exercises improve health-related quality of life and

radiographic parameters in adolescent idiopathic scoliosis patients

SO CHINESE MEDICAL JOURNAL

LA English

DT Article

DE Adolescent idiopathic scoliosis; Schroth exercises; Health-related

quality of life; Curve progression; Cervical alignment

ID CURVE PROGRESSION; BRACE

AB Background: Finding an optimal treatment strategy for adolescent idiopathic scoliosis (AIS) patients remains challenging because of its intrinsic complexity. For mild to moderate scoliosis patients with lower skeletal growth potential (Risser 3-5), most clinicians agree with observation treatment; however, the curve progression that occurs during puberty, the adolescent period, and even in adulthood, remains a challenging issue for clinicians. The aim of the study is to investigate the efficacy of Schroth exercise in AIS patients with lower skeletal growth potential (Risser 3-5) and moderate scoliosis (Cobb angle 20 degrees-40 degrees). Methods: From 2015 to 2017, data of 64 patients diagnosed with AIS in Peking University Third Hospital were reviewed. Forty-three patients underwent Schroth exercise were classified as Schroth group, and 21 patients underwent observation were classified as observation group. Outcomes were measured by health-related quality of life (HRQOL) and radiographic parameters. HRQOL was assessed using the visual analog scale (VAS) scores for back, Scoliosis Research Society-22 (SRS-22) patient questionnaire. Radiographic spinopelvic parameters were obtained from anteroposterior and lateral X-rays. The pre-treatment and post-treatment HRQOL and radiographic parameters were tested to validate Schroth exercise efficacy. The inter-rater reliability of the radiographic parameters was tested using the interclass correlation coefficient (ICC). The paired t test was used to examine HRQOL and radiographic parameters. Clinical relevance between C2-C7 sagittal vertical axis (SVA) and thoracic kyphosis was analyzed using Spearman correlation. Results: In Schroth group, VAS back score, SRS-22 pain, and SRS-22 self-image domain were significantly improved from pre-treatment 3.0 +/- 0.8, 3.6 +/- 0.5, and 3.5 +/- 0.7 to post-treatment 1.6 +/- 0.6 (t = 5.578, P = 0.013), 4.0 +/- 0.3 (t = -3.918, P = 0.001), and 3.7 +/- 0.4 (t = -6.468, P < 0.001), respectively. No significant improvements of SRS-22 function domain (t = -2.825, P = 0.088) and mental health domain (t = -3.174, P = 0.061) were observed. The mean Cobb angle decreased from 28.9 +/- 5.5 degrees to 26.3 +/- 5.2 degrees at the final follow-up, despite no statistical significance was observed (t = 1.853, P = 0.102). The mean C2-C7 SVA value decreased from 21.7 +/- 8.4 mm to 17.0 +/- 8.0 mm (t = -1.224 P = 0.049) and mean T1 tilt decreased from 4.9 +/- 4.2 degrees to 3.5 +/- 3.1 degrees (t = 2.913, P = 0.011). No significant improvement of radiographic parameters and HRQOL were observed in observation group. Conclusions: For AIS patients with a Risser 3-5 and a Cobb angle 20 degrees-40 degrees, Schroth exercises improved HRQOL and halted curve progression during the follow-up period. Both cervical spine alignment and shoulder balance were also significantly improved after Schroth exercises. We recommend Schroth exercises for patients with AIS.

C1 [Gao, Ang; Li, Jun-Yu; Shao, Rui; Wang, Yong-Qiang; Liu, Xiao-Guang; Yu, Miao] Peking Univ Third Hosp, Dept Orthopaed, Beijing 100191, china.

[Wu, Tong-Xuan] Peking Univ Third Hosp, Dept Rehabil Med, Beijing 100191, china.

C3 Peking University

RP Yu, M (通讯作者)， Peking Univ Third Hosp, Dept Orthopaed, Beijing 100191, china.

EM miltonyupku@163.com

RI Yu, Miao/HRC-0342-2023

FU Major Programs of Peking University Third Hospital [Y77491-06]

FX This work was supported by a grant from the Major Programs of Peking

University Third Hospital (No. Y77491-06).

CR Akbar M, 2018, J NEUROSURG-SPINE, V29, P506, DOI 10.3171/2018.3.SPINE171263

Cheung JPY, 2018, CLIN ORTHOP RELAT R, V476, P429, DOI 10.1007/s11999.0000000000000027

Chiu CK, 2020, SPINE, V45, pE319, DOI 10.1097/BRS.0000000000003275

Danielsson AJ, 2012, SPINE, V37, P755, DOI 10.1097/BRS.0b013e318231493c

Danielsson AJ, 2010, SPINE, V35, P199, DOI 10.1097/BRS.0b013e3181c89f4a

Kuru T, 2016, CLIN REHABIL, V30, P181, DOI 10.1177/0269215515575745

Kwan MK, 2016, EUR SPINE J, V25, P401, DOI 10.1007/s00586-015-4016-9

Monticone M, 2014, EUR SPINE J, V23, P1204, DOI 10.1007/s00586-014-3241-y

Negrini S, 2019, ANN PHYS REHABIL MED, V62, P69, DOI 10.1016/j.rehab.2018.07.010

Negrini S, 2018, SCOLIOSIS SPINAL DIS, V13, DOI 10.1186/s13013-017-0145-8

Negrini S, 2015, SCOLIOSIS SPINAL DIS, V10, DOI 10.1186/s13013-014-0025-4

Neuman BJ, 2019, SPINE, V44, pE1311, DOI 10.1097/BRS.0000000000003159

Peng Y, 2020, CHINESE MED J-PEKING, V133, P483, DOI 10.1097/CM9.0000000000000652

Richards BS, 2005, SPINE, V30, P2068, DOI 10.1097/01.brs.0000178819.90239.d0

Schreiber S, 2019, BMC MUSCULOSKEL DIS, V20, DOI 10.1186/s12891-019-2695-9

Schreiber S, 2015, SCOLIOSIS SPINAL DIS, V10, DOI 10.1186/s13013-015-0048-5

Schwieger T, 2016, SPINE, V41, P311, DOI 10.1097/BRS.0000000000001210

Shan Z, 2014, SPINE, V39, pE276, DOI 10.1097/BRS.0000000000000124

Watanabe K, 2020, SPINE, V45, pE83, DOI 10.1097/BRS.0000000000003216

Zapata KA, 2019, PEDIATR PHYS THER, V31, P280, DOI 10.1097/PEP.0000000000000621

Zheng Y, 2018, SPINE, V43, pE494, DOI 10.1097/BRS.0000000000002412

NR 21

TC 6

Z9 6

U1 4

U2 15

PU LIPPINCOTT WILLIAMS & WILKINS

PI PHILADELPHIA

PA TWO COMMERCE SQ, 2001 MARKET ST, PHILADELPHIA, PA 19103 USA

SN 0366-6999

EI 2542-5641

J9 CHINESE MED J-PEKING

JI Chin. Med. J.

PD NOV 5

PY 2021

VL 134

IS 21

BP 2589

EP 2596

DI 10.1097/CM9.0000000000001799

PG 8

WC Medicine, General & Internal

WE Science Citation Index Expanded (SCI-EXPANDED)

SC General & Internal Medicine

GA WS9FQ

UT WOS:000715482100014

PM 34653079

OA Green Published, gold, Green Submitted

DA 2023-08-10

ER

PT J

AU Dufvenberg, M

Diarbakerli, E

Charalampidis, A

Oberg, B

Tropp, H

Ahl, AA

Moller, H

Gerdhem, P

Abbott, A

AF Dufvenberg, Marlene

Diarbakerli, Elias

Charalampidis, Anastasios

Oberg, Birgitta

Tropp, Hans

Ahl, Anna Aspberg

Moller, Hans

Gerdhem, Paul

Abbott, Allan

TI Six-Month Results on Treatment Adherence, Physical Activity, Spinal Appearance, Spinal Deformity, and Quality of Life in an Ongoing Randomised Trial on Conservative Treatment for Adolescent Idiopathic Scoliosis (CONTRAIS)

SO JOURNAL OF CLINICAL MEDICINE

LA English

DT Article

DE idiopathic scoliosis; bracing; physiotherapeutic scoliosis-specific exercise; physical activity; adherence; spinal appearance; health-related quality of life

ID POSTURAL ASYMMETRY MEASUREMENT; PATIENT QUESTIONNAIRE; MILD ADOLESCENT; VALIDITY; RELIABILITY; HEALTH; BRACE; RESPONSIVENESS; CHILDREN; INSTRUMENT

AB Adolescents with idiopathic scoliosis (AIS) often receive conservative treatments aiming to prevent progression of the spinal deformity during puberty. This study aimed to explore patient adherence and secondary outcomes during the first 6 months in an ongoing randomised controlled trial of three treatment interventions. Interventions consisted of physical activity combined with either hypercorrective Boston brace night shift (NB), scoliosis-specific exercise (SSE), or physical activity alone (PA). Measures at baseline and 6 months included angle of trunk rotation (ATR), Cobb angle, International Physical Activity Questionnaire short form (IPAQ-SF), pictorial Spinal Appearance Questionnaire (pSAQ), Scoliosis Research Society (SRS-22r), EuroQol 5-Dimensions Youth (EQ-5D-Y) and Visual Analogue Scale (EQ-VAS). Patient adherence, motivation, and capability in performing the intervention were reported at 6 months. The study included 135 patients (111 females) with AIS and >1-year estimated remaining growth, mean age 12.7 (1.4) years, and mean Cobb angle 31 (+/- 5.3). At 6 months, the proportion of patients in the groups reporting high to very high adherence ranged between 72 and 95%, while motivation ranged between 65 and 92%, with the highest proportion seen in the NB group (p = 0.014, p= 0.002). IPAQ-SF displayed significant between group main effects regarding moderate activity (F = 5.7; p = 0.004;eta(p)(2) =

0.10), with a medium-sized increase favouring the SSE group compared to NB. Walking showed significant between group main effects, as did metabolic equivalent (MET-min/week), with medium (F = 6.8, p = 0.002;eta(p)(2) = 0.11, and large (F = 8.3, p = < 0.001,eta(p)(2) = 0.14) increases, respectively, for the SSE and PA groups compared to NB. From baseline to 6 months, ATR showed significant between group medium-sized main effects (F = 1.2, p = 0.019, eta(p)(2) = 0.007) favouring the NB group compared to PA, but not reaching a clinically relevant level. In conclusion, patients reported high adherence and motivation to treatment, especially in the NB group. Patients in the SSE and PA groups increased their physical activity levels without other clinically relevant differences between groups in other clinical measures or patient-reported outcomes. The results suggest that the prescribed treatments are viable first-step options during the first 6 months.

C1 [Dufvenberg, Marlene; Oberg, Birgitta; Abbott, Allan] Linkoping Univ, Dept Hlth Med & Caring Sci, Unit Physiotherapy, SE-58183 Linkoping, Sweden.

[Diarbakerli, Elias; Charalampidis, Anastasios; Moller, Hans; Gerdhem, Paul] Karolinska Inst, Dept Clin Sci Intervent & Technol CLINTEC, Div Orthopaed & Biotechnol, SE-14186 Stockholm, Sweden.

[Diarbakerli, Elias; Charalampidis, Anastasios; Gerdhem, Paul] Karolinska Univ Hosp Huddinge, Dept Reconstruct Orthopaed, SE-14186 Stockholm, Sweden.

[Tropp, Hans] Linkoping Univ, Dept Biomed & Clin Sci, SE-58183 Linkoping, Sweden.

[Tropp, Hans] Linkoping Univ, Ctr Med Image Sci & Visualizat, SE-58183 Linkoping, Sweden.

[Tropp, Hans; Abbott, Allan] Linkoping Univ Hosp, Dept Orthopaed, SE-58183 Linkoping, Sweden.

[Ahl, Anna Aspberg] Ryhov Cty Hosp, Dept Orthopaed, SE-55185 Jonkoping, Sweden.

[Moller, Hans] Stockholm Ctr Spine Surg, SE-17164 Stockholm, Sweden.

C3 Linkoping University; Karolinska Institutet; Karolinska Institutet;

Karolinska University Hospital; Linkoping University; Linkoping

University; Linkoping University

RP Dufvenberg, M (通讯作者)， Linkoping Univ, Dept Hlth Med & Caring Sci, Unit Physiotherapy, SE-58183 Linkoping, Sweden.

EM marlene.dufvenberg@liu.se; elias.diarbakerli@sll.se;

anastasios.charalampidis@sll.se; birgitta.oberg@liu.se;

hans.tropp@regionostergotland.se; anna.aspberg.ahl@rjl.se;

hans.moller@rkc.se; paul.gerdhem@sll.se; allan.abbott@liu.se

OI Abbott, Allan/0000-0002-4318-9216; Charalampidis,
Anastasios/0000-0001-5228-738X; Oberg, Birgitta/0000-0001-8612-583X;
Dufvenberg, Marlene/0000-0002-3639-6380

FU Swedish Research Council [521-20121771]; Stockholm County Council;
Karolinska Institutet; Linkopings University; Swedish Society of Spinal
Surgeons; Region Stockholm

FX This research was financially supported by The Swedish Research Council
(Dnr 521-20121771); the regional agreement on medical training and
clinical research (ALF) between Stockholm County Council, Karolinska
Institutet, and Linkopings University; and the Swedish Society of Spinal
Surgeons. Paul Gerdhem was supported by Region Stockholm (clinical
research appointment).

CR Abbott A, 2013, BMC MUSCULOSKEL DIS, V14, DOI 10.1186/1471-2474-14-261
[Anonymous], 2014, ACES
Asher M, 2003, SPINE, V28, P74, DOI 10.1097/00007632-200301010-00017
Asher MA, 2006, SPINE, V31, P593, DOI 10.1097/01.brs.0000201331.50597.ea
Berdishevsky H, 2016, SCOLIOSIS SPINAL DIS, V11, DOI 10.1186/s13013-016-0076-9
Bergfors S, 2015, ACTA PAEDIATR, V104, P167, DOI 10.1111/apa.12863
Bonagamba GH, 2010, BRAZ J PHYS THER, V14, P432, DOI 10.1590/S1413-35552010005000025
Boston Orthotics and Prosthetics, BOST BRAC NIGHT SHIF
Burstrom K, 2014, ACTA PAEDIATR, V103, P426, DOI 10.1111/apa.12557
Burstrom K, 2011, EUR J PUBLIC HEALTH, V21, P171, DOI 10.1093/eurpub/ckq037
Carreon Leah Y, 2013, Spine Deform, V1, P328, DOI 10.1016/j.jspd.2013.06.001
Carreon LY, 2011, SPINE, V36, pE1240, DOI 10.1097/BRS.0b013e318204f987
Cheung PWH, 2018, EUR SPINE J, V27, P278, DOI 10.1007/s00586-017-5330-1

Cohen J., 2013, STAT POWER ANAL BEHA, V2nd

Costa L, 2021, J CLIN MED, V10, DOI 10.3390/jcm10102145

Craig CL, 2003, MED SCI SPORT EXER, V35, P1381, DOI 10.1249/01.MSS.0000078924.61453.FB

Danielsson AJ, 2013, SPINE, V38, P1875, DOI 10.1097/BRS.0b013e3182a211c0

Danielsson AJ, 2012, SPINE, V37, P755, DOI 10.1097/BRS.0b013e318231493c

Devlin N., USING EQ 5D PERFORMA

Diarbakerli E, 2020, BONE JOINT J, V102B, P268, DOI 10.1302/0301-620X.102B2.BJJ-2019-1016.R1

Diarbakerli E, 2019, SPINE, V44, P404, DOI 10.1097/BRS.0000000000002857

Diarbakerli E, 2018, J BONE JOINT SURG AM, V100, P811, DOI 10.2106/JBJS.17.00822

Diarbakerli E, 2017, EUR SPINE J, V26, P1631, DOI 10.1007/s00586-016-4854-0

Dolan LA, 2020, SPINE, V45, P1193, DOI 10.1097/BRS.0000000000003506

Ekelund U, 2006, PUBLIC HEALTH NUTR, V9, P258, DOI 10.1079/PHN2005840

George M, 2018, RESP CARE, V63, P818, DOI 10.4187/respcare.05905

International Physical Activity Questionnaire. IPAQ Research Committee, INT PHYS ACT QUEST

Janicki JA, 2007, J PEDIATR ORTHOPED, V27, P369, DOI 10.1097/01.bpb.0000271331.71857.9a

Janssen I, 2010, INT J BEHAV NUTR PHY, V7, DOI 10.1186/1479-5868-7-40

Kazdin AE, 2007, ANNU REV CLIN PSYCHO, V3, P1, DOI 10.1146/annurev.clinpsy.3.022806.091432

LANDIS JR, 1977, BIOMETRICS, V33, P159, DOI 10.2307/2529310

Langensiepen S, 2013, EUR SPINE J, V22, P2360, DOI 10.1007/s00586-013-2693-9

Makino T, 2019, SPINE, V44, pE833, DOI 10.1097/BRS.0000000000002996

McKay HA, 2000, J PEDIATR-US, V136, P156, DOI 10.1016/S0022-3476(00)70095-3

Michie S, 2011, IMPLEMENT SCI, V6, DOI 10.1186/1748-5908-6-42

Monticone M, 2017, SPINE, V42, pE672, DOI 10.1097/BRS.0000000000001923

Monticone M, 2014, EUR SPINE J, V23, P1204, DOI 10.1007/s00586-014-3241-y

NACHEMSON AL, 1995, J BONE JOINT SURG AM, V77A, P815, DOI 10.2106/00004623-199506000-00001

Negrini S, 2018, SCOLIOSIS SPINAL DIS, V13, DOI 10.1186/s13013-017-0145-8

Negrini S, 2012, SCOLIOSIS SPINAL DIS, V7, DOI 10.1186/1748-7161-7-3

Negrini S, 2015, COCHRANE DB SYST REV, DOI 10.1002/14651858.CD006850.pub3

Piantoni L, 2018, SCOLIOSIS SPINAL DIS, V13, DOI 10.1186/s13013-018-0172-0

Prowse A, 2017, WORLD J ORTHOP, V8, P68, DOI 10.5312/wjo.v8.i1.68

Prowse A, 2016, EUR SPINE J, V25, P450, DOI 10.1007/s00586-015-3961-7

Public Health Agency of Sweden, LIV COND LIF PHYS AC

Ramirez N, 1997, J BONE JOINT SURG AM, V79A, P364, DOI 10.2106/00004623-199703000-00007

Ravens-Sieberer U, 2010, QUAL LIFE RES, V19, P887, DOI 10.1007/s11136-010-9649-x

Ruffilli A, 2021, SPINE DEFORM, V9, P671, DOI 10.1007/s43390-020-00248-5

Sabatee Eduardo, 2003, ADHERENCE LONG TERM

Sanders JO, 2007, SPINE, V32, P2719, DOI 10.1097/BRS.0b013e31815a5959

Sanders JO, 2003, SPINE, V28, P2158, DOI 10.1097/01.BRS.0000084629.97042.0B

Sattout Amjad, 2016, Spine Deform, V4, P253, DOI 10.1016/j.jspd.2015.12.004

Savvides P, 2020, SPINE, V45, P522, DOI 10.1097/BRS.0000000000003308

Schreiber S, 2015, SCOLIOSIS SPINAL DIS, V10, DOI 10.1186/s13013-015-0048-5

Schwieger T, 2016, SPINE, V41, P311, DOI 10.1097/BRS.0000000000001210

Shephard RJ, 2016, SPRINGER SER EPIDEMI, P85, DOI 10.1007/978-3-319-29577-0_3

Simony A, 2019, EUR SPINE J, V28, P2020, DOI 10.1007/s00586-019-06077-z

Thompson JY, 2019, PHYSIOTHERAPY, V105, P214, DOI 10.1016/j.physio.2018.10.004

Weinstein SL, 2003, JAMA-J AM MED ASSOC, V289, P559, DOI 10.1001/jama.289.5.559

Weinstein SL, 2015, J BONE JOINT SURG AM, V97A, P1899, DOI 10.2106/JBJS.O.00330

Weiss H.-R., 2008, CONSERVATIVE SCOLIOS, V135, P173

Weiss H-R, 2006, Pediatr Rehabil, V9, P190, DOI 10.1080/13638490500079583

Wille N, 2010, QUAL LIFE RES, V19, P875, DOI 10.1007/s11136-010-9648-y

Williams MA, 2015, HEALTH TECHNOL ASSES, V19, DOI 10.3310/hta19550

World Health Organization, WHO GUID PHYS ACT SE

Yip BHK, 2016, SCI REP-UK, V6, DOI 10.1038/srep39220

NR 66

TC 4

Z9 4

U1 5

U2 12

PU MDPI

PI BASEL

PA ST ALBAN-ANLAGE 66, CH-4052 BASEL, SWITZERLAND

EI 2077-0383

J9 J CLIN MED

JI J. Clin. Med.

PD NOV

PY 2021

VL 10

IS 21

AR 4967

DI 10.3390/jcm10214967

PG 17

WC Medicine, General & Internal

WE Science Citation Index Expanded (SCI-EXPANDED); Social Science Citation Index (SSCI)

SC General & Internal Medicine

GA 0F2JC

UT WOS:000777190700011

PM 34768487

OA Green Published, gold

DA 2023-08-10

ER

PT J

AU Figueras, C

Matamalas, A

Pizones, J

Moreno-Manzanaro, L

Betegon, J

Bago, J

AF Figueras, Clara

Matamalas, Antonia

Pizones, Javier

Moreno-Manzanaro, Lucia

Betegon, Jesus

Bago, Juan

TI The Relationship of Kinesiophobia with Pain and Quality of Life in Idiopathic Scoliosis

SO SPINE

LA English

DT Article

DE adolescent idiopathic scoliosis; construct validity; kinesiophobia;

Tampa Scale of Kinesiophobia

ID LOW-BACK-PAIN; FEAR-AVOIDANCE BELIEFS; TAMPA SCALE; PSYCHOMETRIC

PROPERTIES; SPANISH VERSION; PATIENT QUESTIONNAIRE; PREVALENCE;

RELIABILITY; DEPRESSION; ANXIETY

AB Study Design. Prospective, multi-centric, cross-sectional study.

Objective. To analyze the construct validity of the Tampa Scale of Kinesiophobia (TSK) in a cohort of patients with adolescent idiopathic scoliosis (AIS).

Summary of the Background Data. Back pain is not uncommon in AIS. The fear of movement (kinesiophobia) in response to pain is related to back pain. TSK psychometric properties in AIS patients have not been properly analyzed.

Methods. Patients with AIS and no prior spine surgery were prospectively included. They fulfilled the Spanish version of the TSK-11 questionnaire, a pain intensity numerical rating scale (NRS), refined SRS-22 (SRS-22r), the Hospital Anxiety and Depression Scale (HADS), and item 7 of the Core Outcome Measurement Index (COMI). The sample was split into two groups for the statistical analysis: adolescents and young adults. Cronbach alpha was used to assess internal consistency. Discriminant and concurrent validity were obtained by computing Pearson correlation coefficients between the TSK score and several criterion measures.

Results. A total of 275 patients were included-198 adolescents (mean age of 14.6 yrs) and 77 young adults (mean age of 26.9 yrs). The Cobb of largest curve means were 44.9 degrees and 48.9 degrees, respectively. In the adolescent group, the TSK mean (+/- SD) was 21.5 (+/- 5.93), with a floor effect of 2.5%. In the adult group, the TSK mean was 24.2 (+/- 6.63), with a floor effect of 3.9%. The ceiling effect was 0% in both groups. Cronbach alphas for the adolescent and adult groups were 0.76 and 0.79, respectively. No correlation was found in any group between the TSK score and the curve magnitude or pattern (Lenke classification). The TSK was significantly correlated with HAD depression and SRS-22r in both groups. However, these correlations were weaker in adolescents. In adults, the TSK also significantly correlates with NRS and work/school absenteeism.

Conclusion. The Spanish version of TSK-11 is a reliable and valid instrument to analyze kinesiophobia in AIS. However, the weak correlation between kinesiophobia and pain intensity, disability, and emotional condition in adolescents requires further study.

C1 [Figueras, Clara] Vall DHebron Res Inst VHIR, Fundacio Inst Recerca Vall Hebron, Barcelona, Spain.

[Figueras, Clara] Univ Autonoma Barcelona, Dept Surg & Morphol Sci, Barcelona, Spain.

[Matamalas, Antonia; Bago, Juan] Hosp Valle De Hebron, Orthopaed Surg Dept, Spine Unit, Barcelona, Spain.

[Pizones, Javier; Moreno-Manzanaro, Lucia] Hosp Univ La Paz, Orthopaed Surg Dept, Spine Unit, Madrid, Spain.

[Betegon, Jesus] Complejo Asistencial Univ Leon CAULE, Orthopaed Surg Dept, Spine Unit, Leon, Spain.

C3 Autonomous University of Barcelona; Hospital Universitari Vall d'Hebron;

Vall d'Hebron Institut de Recerca (VHIR); Autonomous University of

Barcelona; Hospital Universitari Vall d'Hebron; Hospital Universitario

La Paz; Universidad de Leon

RP Figueras, C (通讯作者)， Passeig Vall dHebron 119-129, Barcelona 08035, Spain.

EM clarafiguerassanroman@gmail.com

RI Bagó, Joan/AAD-5072-2022

OI Bagó, Joan/0000-0001-9359-8088; Figueras, Clara/0000-0003-0543-7191;

Betegon Nicolas, Jesus/0000-0001-9863-9432

FU Spanish Spine Society (GEER: Sociedad para el Estudio de les

Enfermedades del Raquis); Eurospine; Nuvasive funds

FX The Spanish Spine Society (GEER: Sociedad para el Estudio de les

Enfermedades del Raquis), Eurospine, and Nuvasive funds were received in

support of this work.

CR Asher M, 2003, SPINE, V28, P63, DOI 10.1097/00007632-200301010-00015

Bago J, 2004, SPINE, V29, P1676, DOI 10.1097/01.BRS.0000132306.53942.10

Edwards RR, 2016, J PAIN, V17, pT70, DOI 10.1016/j.jpain.2016.01.001

Gomez-Perez L, 2011, J PAIN, V12, P425, DOI 10.1016/j.jpain.2010.08.004

Lenke LG, 2001, J BONE JOINT SURG AM, V83A, P1169, DOI 10.2106/00004623-200108000-00006

Makino T, 2015, SPRINGERPLUS, V4, DOI 10.1186/s40064-015-1189-y

Mannion AF, 2016, EUR SPINE J, V25, P2638, DOI 10.1007/s00586-015-4292-4

MAYO NE, 1994, SPINE, V19, P1573, DOI 10.1097/00007632-199407001-00005

Miller RP., 1991, CLIN J PAIN, V7, P51, DOI 10.1097/00002508-199103000-00053

Miro J, 2009, EUR J PAIN, V13, P1089, DOI 10.1016/j.ejpain.2009.07.002

Neblett R, 2016, EUR J PAIN, V20, P701, DOI 10.1002/ejp.795

Pellise F, 2009, ARCH PEDIAT ADOL MED, V163, P65, DOI 10.1001/archpediatrics.2008.512

Quintana JM, 2003, ACTA PSYCHIAT SCAND, V107, P216, DOI 10.1034/j.1600-0447.2003.00062.x

Ramirez N, 1997, J BONE JOINT SURG AM, V79A, P364, DOI 10.2106/00004623-199703000-00007

Ramirez N, 2020, SPINE DEFORM, V8, P663, DOI 10.1007/s43390-020-00065-w

Ranger TA, 2020, SPINE J, V20, P857, DOI 10.1016/j.spinee.2020.02.002

Sanchez-Mariscal F, 2012, SPINE, V37, P592, DOI 10.1097/BRS.0b013e318227336a

Sato T, 2011, EUR SPINE J, V20, P274, DOI 10.1007/s00586-010-1657-6

Schober P, 2018, ANESTH ANALG, V126, P1763, DOI 10.1213/ANE.0000000000002864

Teles AR, 2019, SPINE J, V19, P677, DOI 10.1016/j.spinee.2018.10.009

Theroux J, 2017, CHIROPR MAN THER, V25, DOI 10.1186/s12998-017-0143-1

Tkachuk GA, 2012, J PAIN, V13, P970, DOI 10.1016/j.jpain.2012.07.001

WADDELL G, 1993, PAIN, V52, P157, DOI 10.1016/0304-3959(93)90127-B

Wertli MM, 2014, SPINE J, V14, P816, DOI 10.1016/j.spinee.2013.09.036

Woby SR, 2005, PAIN, V117, P137, DOI 10.1016/j.pain.2005.05.029

Wong AYL, 2019, CLIN ORTHOP RELAT R, V477, P676, DOI 10.1097/CORR.0000000000000569

Ye DL, 2020, PHYS OCCUP THER PEDI, V40, P546, DOI 10.1080/01942638.2020.1720054

NR 27

TC 4

Z9 5

U1 3

U2 6

PU LIPPINCOTT WILLIAMS & WILKINS

PI PHILADELPHIA

PA TWO COMMERCE SQ, 2001 MARKET ST, PHILADELPHIA, PA 19103 USA

SN 0362-2436

EI 1528-1159

J9 SPINE

JI SPINE

PD NOV 1

PY 2021

VL 46

IS 21

BP 1455

EP 1460

DI 10.1097/BRS.0000000000004066

PG 6

WC Clinical Neurology; Orthopedics

WE Science Citation Index Expanded (SCI-EXPANDED)

SC Neurosciences & Neurology; Orthopedics

GA ZB5YP

UT WOS:000756917700010

PM 34618706

DA 2023-08-10

ER

PT J

AU Gou, YY

Lei, HW

Zeng, Y

Tao, J

Kong, WC

Wu, JS

AF Gou, Yanyun

Lei, Huangwei

Zeng, Yi

Tao, Jing

Kong, Weicheng

Wu, Jingsong

TI The effect of Pilates exercise training for scoliosis on improving

spinal deformity and quality of life Meta-analysis of randomized

controlled trials

SO MEDICINE

LA English

DT Review

DE meta-analysis; Pilates exercises training; scoliosis

ID LOW-BACK-PAIN; IDIOPATHIC SCOLIOSIS; PEDRO SCALE; DISABILITY; SCHROTH

AB Background: It remains unclear if Pilates is conducive to reducing spinal deformity and improving patients' quality of life (QOL) with scoliosis. The aim of this study was to systematically review the published evidence to determine whether Pilates exercise training is an efficacious therapy for scoliosis. Methods: Searches was conducted in Medline, Embase, PubMed, Scopus, CINAHL, Physiotherapy Evidence Database (PEDro), Cochrane library, Baidu Scholar, and Green Medical to identify randomized studies that tested the effect of Pilates exercise training on Cobb angle, pain level, trunk range of motion (ROM), angle of trunk rotation, and QOL in idiopathic scoliosis. Separate meta-analyses were performed on the endpoints of these outcome measures. The PEDro scale was used to assess the methodological quality of the included studies. Results: This review included 10 randomized controlled trials (n = 359). PEDro scores ranged from 3 to 10, with the mean score across all articles being 5.3/10 and judged to be of fair quality. The results indicated that Pilates exercises was effective in reducing Cobb angle (standardized mean difference [SMD] = 1.23, 95%

confidence interval [CI] = 0.11-2.35), angle of trunk rotation (SMD = 1.37, 95% CI = 1.01-1.73), and pain level (SMD = 2.78, 95% CI = 1.55-4.01), as well as improving trunk ROM (SMD = 1.23, 95% CI = 0.45-2.00), and QOL (SMD = 3.05, 95% CI = 2.59-3.51) in patients with scoliosis. Conclusion: Pilates exercise training may reduce the Cobb angle and trunk rotation, relieve pain, increase trunk ROM, and improve QOL for patients with scoliosis. Due to the poor quality of the evidence, however, these results should be interpreted with caution.

C1 [Gou, Yanyun; Lei, Huangwei; Zeng, Yi; Tao, Jing; Kong, Weicheng; Wu, Jingsong] Fujian Univ Tradit Chinese Med, Coll Rehabil Med, 1 Qiuyang Rd, Fuzhou 350122, Fujian, china.

C3 Fujian University of Traditional Chinese Medicine

RP Wu, JS (通讯作者)， Fujian Univ Tradit Chinese Med, Coll Rehabil Med, 1 Qiuyang Rd, Fuzhou 350122, Fujian, china.

EM jingsongwu01@163.com

RI Tao, Jing/GVS-6340-2022

OI Tao, Jing/0000-0002-4895-2065

FU Natural Science Foundation of Fujian Province, China [2019J01361];

Fujian Innovation and Entrepreneurship Training Program, China

[202010393039]

FX This study was supported by the Natural Science Foundation of Fujian

Province, China (Grant No. 2019J01361); Fujian Innovation and

Entrepreneurship Training Program, China (Grant No. 202010393039).

CR de Araujo MEA, 2012, J BODYW MOV THER, V16, P191, DOI 10.1016/j.jbmt.2011.04.002

Aly MIE., 2019, J ADV PHARM ED RES, V9, P155

Anwer S, 2015, BIOMED RES INT-UK, V2015, DOI 10.1155/2015/123848

Berdishevsky H, 2016, SCOLIOSIS SPINAL DIS, V11, DOI 10.1186/s13013-016-0076-9

Bipin Das UR., 2019, ISOLATED COMBINED EF

Bipin Das UR., 2018, ISOLATED COMBINED EF

Casonatto J, 2020, COMPLEMENT THER MED, V48, DOI 10.1016/j.ctim.2019.102232

de Araujo MEA., 2010, MOTRIZ RIO CLARO, V16

de Morton NA, 2009, AUST J PHYSIOTHER, V55, P129, DOI 10.1016/S0004-9514(09)70043-1

Dunn J, 2018, JAMA-J AM MED ASSOC, V319, P173, DOI 10.1001/jama.2017.11669

Emery K, 2010, CLIN BIOMECH, V25, P124, DOI 10.1016/j.clinbiomech.2009.10.003

Gonzalez-Galvez N, 2019, COMPLEMENT THER CLIN, V35, P1, DOI 10.1016/j.ctcp.2019.01.006

Gouveia Kmc GE., 2008, FISIOTER MOV, V21

Higgins JPT, 2003, BMJ-BRIT MED J, V327, P557, DOI 10.1136/bmj.327.7414.557

Hurer C, 2021, J COMP EFFECT RES, V10, P365, DOI 10.2217/cer-2020-0186

Hwangbo P-N., 2016, J KOREAN PHYS THER, V28

Im KC., 2014, EFFECT TRACK FIELD E

Jin XZ., 2015, EFFECT SPORTS MASSAG

Jin XZ., 2020, EFFECTS SPORTS MASSA

Kim G, 2016, J PHYS THER SCI, V28, P1012, DOI 10.1589/jpts.28.1012

Last RJ., 1978, ANATOMY REGIONAL APP

임관철, 2015, [The Korean Society of Sports Science, 한국체육과학회지], V24, P1161

Li X., 2020, J SHANXI DATONG U NA

Lim ECW, 2011, J ORTHOP SPORT PHYS, V41, P70, DOI 10.2519/jospt.2011.3393

Maher CG, 2003, PHYS THER, V83, P713, DOI 10.1093/ptj/83.8.713

Martin S, 2017, TOP GERIATR REHABIL, V33, P43, DOI 10.1097/TGR.0000000000000130

Meiying Li., 2018, FUJIAN SPORTS SCI TE, V37, P46

Melinda Bryan SH., 2003, TECH ORTHOP, V18, P126

Menger RP., 2021, ADOLESCENT IDIOPATHI

Miyamoto GC, 2013, BRAZ J PHYS THER, V17, P517, DOI 10.1590/S1413-35552012005000127

Monticone M, 2016, EUR SPINE J, V25, P3120, DOI 10.1007/s00586-016-4528-y

Moreno-Segura N, 2018, J AGING PHYS ACTIV, V26, P327, DOI 10.1123/japa.2017-0078

Moseley AM, 2020, BRAZ J PHYS THER, V24, P384, DOI 10.1016/j.bjpt.2019.11.002

Moseley AM, 2019, PLOS ONE, V14, DOI 10.1371/journal.pone.0222770

Mueller D, 2021, J BODYW MOV THER, V25, P80, DOI 10.1016/j.jbmt.2020.11.012

Nahid A., 2019, J REHABIL MED, V8, P221

Negrini S, 2006, SCOLIOSIS SPINAL DIS, V1, DOI 10.1186/1748-7161-1-4

Negrini S, 2018, SCOLIOSIS SPINAL DIS, V13, DOI 10.1186/s13013-017-0145-8

Negrini S, 2012, SCOLIOSIS SPINAL DIS, V7, DOI 10.1186/1748-7161-7-3

Oseph E., 2004, J BODYW MOV THER, V8, P122

Park JH, 2018, EUR J PHYS REHAB MED, V54, P440, DOI 10.23736/S1973-9087.17.04461-6

Patti A, 2021, MEDICINE, V100, DOI 10.1097/MD.0000000000025289

Roh SY, 2018, J EXERC REHABIL, V14, P944, DOI 10.12965/jer.1836436.218

Romano M, 2012, COCHRANE DB SYST REV, DOI 10.1002/14651858.CD007837.pub2

Rrecaj-Malaj S, 2020, MED SCI MONIT BASIC, V26, DOI 10.12659/MSMBR.920449

Rydeard R, 2006, J ORTHOP SPORT PHYS, V36, P472, DOI 10.2519/jospt.2006.2144

Saltan A, 2021, PERSPECT PSYCHIATR C, V57, P198, DOI 10.1111/ppc.12547

Segal NA, 2004, ARCH PHYS MED REHAB, V85, P1977, DOI 10.1016/j.apmr.2004.01.036

Sekendiz B, 2007, J BODYW MOV THER, V11, P318, DOI 10.1016/j.jbmt.2006.12.002

Tang R., 2019, CONT MED, V25, P176

Weinstein SL, 2008, LANCET, V371, P1527, DOI 10.1016/S0140-6736(08)60658-3

Weiss HR, 2011, SCOLIOSIS SPINAL DIS, V6, DOI 10.1186/1748-7161-6-17

Wells C, 2012, COMPLEMENT THER MED, V20, P253, DOI 10.1016/j.ctim.2012.02.005

Wick Jane Maureen, 2009, AORN J, V90, P347

황보필녀, 2018, [The Journal of Korean Society of Physical Therapy, 대한물리치료학회지], V30, P229, DOI 10.18857/jkpt.2018.30.6.229

NR 55

TC 4

Z9 4

U1 5

U2 20

PU LIPPINCOTT WILLIAMS & WILKINS

PI PHILADELPHIA

PA TWO COMMERCE SQ, 2001 MARKET ST, PHILADELPHIA, PA 19103 USA

SN 0025-7974

EI 1536-5964

J9 MEDICINE

JI Medicine (Baltimore)

PD OCT 1

PY 2021

VL 100

IS 39

AR e27254

DI 10.1097/MD.0000000000027254

PG 9

WC Medicine, General & Internal

WE Science Citation Index Expanded (SCI-EXPANDED); Social Science Citation Index (SSCI)

SC General & Internal Medicine

GA UX7KX

UT WOS:000701020600016

PM 34596121

OA gold, Green Published

DA 2023-08-10

ER

PT J

AU Diarbakerli, E

Charalampidis, A

Abbott, A

Gerdhem, P

AF Diarbakerli, Elias

Charalampidis, Anastasios

Abbott, Allan

Gerdhem, Paul

TI PReventing Idiopathic SCOliosis PROgression (PRISCOPRO): A protocol for

a quadruple-blinded, randomized controlled trial comparing 3D designed

Boston brace to standard Boston brace

SO PLOS ONE

LA English

DT Article

ID QUALITY-OF-LIFE; ADOLESCENT

AB Introduction

Idiopathic scoliosis is the most common spinal deformity in children. Treatment strategies aim to halt progression of the curve. Patients are treated mainly with thoracolumbosacral orthosis (TLSO) if indicated. This form of brace treatment has been shown to be cumbersome and tough on growing individuals. However, computer aided design and manufactured (CAD/CAM) braces might increase comfortability and ultimately outcome if compliance is improved. In a multicenter, randomized controlled trial, we aim to compare CAD/CAM designed Boston 3D-brace to standard Boston brace.

Methods

Subjects: 170 previously untreated and skeletally immature children diagnosed with idiopathic scoliosis, aged 9-17 years of age (curve magnitude Cobb 25-40 degrees) will be included. Interventions: Both groups will receive a physical activity prescription according to the World Health Organization recommendations. Randomization will be performed 1:1 to a 3D CAD/CAM designed Boston 3D-brace or a standard Boston brace, both with prescribed daily wear time of 20 hours. Outcome: The subjects will participate in the study until curve progression or until skeletal maturity.

The primary outcome variable is failure of treatment, defined as progression of the Cobb angle more than 6 degrees compared to the baseline x-ray. The progression is confirmed if seen on two consecutive standing spinal x-rays. Radiographs will be taken at each six-month follow-up. Secondary outcome measures include patient and clinical reported outcomes, including number of individuals requiring surgical intervention.

Discussion

This study will show if efficacy in brace treatment can be improved with new brace designs.

C1 [Diarbakerli, Elias; Charalampidis, Anastasios; Gerdhem, Paul] Karolinska Inst, Dept Clin Sci Intervent & Technol, Stockholm, Sweden.

[Diarbakerli, Elias; Charalampidis, Anastasios; Gerdhem, Paul] Karolinska Univ Hosp, Dept Reconstruct Orthopaed, Stockholm, Sweden.

[Abbott, Allan] Linkoping Univ, Div Prevent Rehabil & Community Med, Dept Hlth Med & Caring Sci, Unit Physiotherapy, Linkoping, Sweden.

[Abbott, Allan] Linkoping Univ Hosp, Dept Orthopaed, Linkoping, Sweden.

C3 Karolinska Institutet; Karolinska Institutet; Karolinska University

Hospital; Linkoping University; Linkoping University

RP Diarbakerli, E (通讯作者)， Karolinska Inst, Dept Clin Sci Intervent & Technol, Stockholm, Sweden.; Diarbakerli, E (通讯作者)， Karolinska Univ Hosp, Dept Reconstruct Orthopaed, Stockholm, Sweden.

EM elias.diarbakerli@sll.se

FU Stockholm County Council (ALF-funds); Sven Jerring Foundation

FX This study is financially supported by the Stockholm County Council

(ALF-funds) and the Sven Jerring Foundation. The funders had and will

not have a role in study design, data collection and analysis, decision

to publish, or preparation of the manuscript.

CR Abbott A, 2013, BMC MUSCULOSKEL DIS, V14, DOI 10.1186/1471-2474-14-261

BUNNELL WP, 1984, J BONE JOINT SURG AM, V66A, P1381, DOI 10.2106/00004623-198466090-00010

Cobb JR., 1948, INSTR COURSE LECT, V5, P261

Cobetto N, 2017, SCOLIOSIS SPINAL DIS, V12, DOI 10.1186/s13013-017-0128-9

Cobetto Nikita, 2014, Spine Deform, V2, P276, DOI 10.1016/j.jspd.2014.03.005

Cottalorda J, 2005, SPINE, V30, P399, DOI 10.1097/01.brs.0000153346.40391.3b

Danielsson AJ, 2001, EUR SPINE J, V10, P278, DOI 10.1007/s005860100309

Desbiens-Blais F, 2012, CLIN BIOMECH, V27, P999, DOI 10.1016/j.clinbiomech.2012.08.006

Diarbakerli E, 2018, J BONE JOINT SURG AM, V100, P811, DOI 10.2106/JBJS.17.00822

Dolan LA, 2020, SPINE, V45, P1193, DOI 10.1097/BRS.0000000000003506

El Hawary R, 2019, SPINE J, V19, P1917, DOI 10.1016/j.spinee.2019.07.008

Hollis S, 1999, BRIT MED J, V319, P670, DOI 10.1136/bmj.319.7211.670

Negrini S, 2016, SPINE, V41, P1813, DOI 10.1097/BRS.0000000000001887

RISSER J C, 1958, Clin Orthop, V11, P111

Sanders JO, 2008, J BONE JOINT SURG AM, V90A, P540, DOI 10.2106/JBJS.G.00004

Wang H, 2021, QUAL LIFE RES, V30, P703, DOI 10.1007/s11136-020-02671-7

Weinstein SL, 2013, NEW ENGL J MED, V369, P1512, DOI 10.1056/NEJMoa1307337

WILLNER S, 1982, ACTA ORTHOP SCAND, V53, P233, DOI 10.3109/17453678208992208

NR 18

TC 1

Z9 1

U1 0

U2 5

PU PUBLIC LIBRARY SCIENCE

PI SAN FRANCISCO

PA 1160 BATTERY STREET, STE 100, SAN FRANCISCO, CA 94111 USA

SN 1932-6203

J9 PLOS ONE

JI PLoS One

PD AUG 9

PY 2021

VL 16

IS 8

AR e0255264

DI 10.1371/journal.pone.0255264

PG 9

WC Multidisciplinary Sciences

WE Science Citation Index Expanded (SCI-EXPANDED)

SC Science & Technology - Other Topics

GA UA6JQ

UT WOS:000685266800048

PM 34370760

OA Green Published, gold

DA 2023-08-10

ER

PT J

AU Matussek, J

Dingeldey, E

Sauerlandt, B

AF Matussek, Jan

Dingeldey, Esther

Sauerlandt, Barbara

TI Physiotherapeutic and rehabilitative options for neuromuscular scolioses

Areas of physiotherapy techniques in the field of tension between

hypertonic and hypotonic forms of neuromuscular scoliosis

SO ORTHOPADE

LA German

DT Article

DE Life quality; Muscle tonus; Neurophysiotherapy; Orthosis; Sitting

position

AB Conservative treatment and physiotherapy (PT) of neuromuscular scoliosis and its underlying systemic causes is dominated by a pragmatic approach. In the choice of treatment, the observance of the underlying muscle tone sets the essential course to select an appropriate PT technique. Hypertonic forms need the reflex-modulation techniques included in Bobath or Vojta programmes. They sometimes necessitate tone-reducing medication and additional orthotics with a likely risk of pressure sores. In hypotonic forms of scoliosis on the other hand, gravity and spine collapse with pulmonal dysfunction are the key issues addressed by conservative treatment. Sustained quality of life and supported partaking in social life through maintenance of pulmonary function and upright sitting ability are of continuing concern. PT programmes with a neurophysiologic background such as those of Bobath and Vojta are of central significance.

C1 [Matussek, Jan; Sauerlandt, Barbara] Helios Klinikum Emil von Behring, Stiftung Oskar Helene Heim, Berlin, Germany.

[Dingeldey, Esther] Orthopad Univ Klin Regensburg, Asklepios Klinikum Bad Abbach, Bad Abbach, Germany.

C3 Helios Kliniken

RP Matussek, J (通讯作者)，Helios Klinikum Emil von Behring, Stiftung Oskar Helene Heim, Berlin, Germany.

EM jan.matussek@helios-gesundheit.de

CR Bobath K., 1998, MOTORISCHE ENTWICKLU

Doderlein L., 2007, INFANTILE ZEREBRALPA

Hirsch S., 2007, KLAPPSCHES KRIECHEN

Locher Hermann, LEITLINIEN DGMM DTSC

Schweizer, 2008, ENTDECKUNG IDEALEN M

Steding-Albrecht U., 2003, BOBATH KONZEPT ALLTA

Vialle R, 2013, ORTHOP TRAUMATOL-SUR, V99, pS124, DOI 10.1016/j.otsr.2012.11.002

Vojta, 2004, ZEREBRALEN BEWEGUNGS

Weiβ H-R., 1994, PRINZIPIEN ERGEBNISS

Weise S, 2011, NEURO DEV TREATM CON, DOI [10.21256/zhaw-355, DOI 10.21256/ZHAW-355]

NR 10

TC 1

Z9 2

U1 0

U2 6

PU SPRINGER

PI NEW YORK

PA ONE NEW YORK PLAZA, SUITE 4600, NEW YORK, NY, UNITED STATES

SN 0085-4530

EI 1433-0431

J9 ORTHOPADE

JI Orthopade

PD AUG

PY 2021

VL 50

IS 8

SI SI

BP 614

EP 621

DI 10.1007/s00132-021-04136-2

EA AUG 2021

PG 8

WC Orthopedics

WE Science Citation Index Expanded (SCI-EXPANDED)

SC Orthopedics

GA TZ1GR

UT WOS:000681143300002

PM 34347139

DA 2023-08-10

ER

PT J

AU Marin, L

Kawczynski, A

Pellino, VC

Febbi, M

Silvestri, D

Pedrotti, L

Lovecchio, N

Vandoni, M

AF Marin, Luca

Kawczynski, Adam

Carnevale Pellino, Vittoria

Febbi, Massimiliano

Silvestri, Dario

Pedrotti, Luisella

Lovecchio, Nicola

Vandoni, Matteo

TI Displacement of Centre of Pressure during Rehabilitation Exercise in

Adolescent Idiopathic Scoliosis Patients

SO JOURNAL OF CLINICAL MEDICINE

LA English

DT Article

DE adolescent idiopathic scoliosis; posture; balance; center of pressure;

self-elongation

ID POSTURAL CONTROL; BALANCE; RELIABILITY; ALIGNMENT; SYSTEM

AB Background. Adolescent idiopathic scoliosis (AIS) is included into the category of pathologies that could affect postural control. Rarely AIS shows symptoms but often compromises the normal positioning of the head, trunk and, more generally, of the limbs in the space. We used a stabilometric platform to evaluate the motor control outcomes during a self-elongation in girls with AIS. Methods. In 10 girls with AIS, we evaluated the center of pressure (COP) modifications on a baropodometric platform in a standing position and after a self-elongation correction. Results. All the outcomes (except the eccentricity) showed an increasement during the self-elongation exercise even if the significant differences were not found. Conclusion. SE correction contributes to ameliorate the COP symmetry with a better repercussion on the balance management. This highlights the importance of repetitions during all activities of daily life.

C1 [Marin, Luca; Febbi, Massimiliano] Lab Rehabil Med & Sport LARMS, I-00133 Rome, Italy.

[Marin, Luca; Febbi, Massimiliano; Silvestri, Dario] ASOMI Coll Sci, Dept Res, Marsa 2080, Malta.

[Kawczynski, Adam] Univ Sch Phys Educ, Dept Paralymp Sports, PL-51617 Wroclaw, Poland.

[Carnevale Pellino, Vittoria; Lovecchio, Nicola; Vandoni, Matteo] Univ Pavia, Dept Publ Hlth Expt & Forens Med, Lab Adapted Motor Act LAMA, I-27100 Pavia, Italy.

[Carnevale Pellino, Vittoria] Univ Tor Vergata, Dept Ind Engn, I-00133 Rome, Italy.

[Pedrotti, Luisella] Univ Pavia, Dept Pediat Diagnost Surg Clin Sci, Sect Pathol Musculoskeletal Syst, Orthoped Unit, I-27100 Pavia, Italy.

C3 University of Pavia; University of Rome Tor Vergata; University of Pavia

RP Lovecchio, N (通讯作者)， Univ Pavia, Dept Publ Hlth Expt & Forens Med, Lab Adapted Motor Act LAMA, I-27100 Pavia, Italy.

EM luca.marin@unipv.it; kawczynski.a@gmail.com;

vittoria.carnevalepellino@unipv.it; massimilianofebbi@gmail.com;
direttore@asomi-osteopatia.com; luisella.pedrotti@unipv.it;
Nicola.lovecchio@unipv.it; matteo.vandoni@unipv.it
RI Kawczynski, Adam/AAK-3031-2021; Pedrotti, Luisella/AFG-2359-2022;
Lovecchio, Nicola/GSM-6629-2022; vandoni, matteo/AAS-7299-2021; Marin,
Luca/K-2992-2018
OI Kawczynski, Adam/0000-0001-7840-3799; vandoni,
matteo/0000-0002-3405-9364; Marin, Luca/0000-0002-4185-7428; Silvestri,
Dario/0000-0002-8730-0107; lovecchio, nicola/0000-0003-4115-0833;
Carnevale Pellino, Vittoria/0000-0003-2299-6366
CR Amaral AP, 2013, BRAZ J PHYS THER, V17, P121, DOI 10.1590/S1413-35552012005000075
Anwer S, 2015, BIOMED RES INT-UK, V2015, DOI 10.1155/2015/123848
Baldini A, 2013, J ELECTROMYOGR KINES, V23, P1474, DOI 10.1016/j.jelekin.2013.08.003
Berdishevsky H, 2016, SCOLIOSIS SPINAL DIS, V11, DOI 10.1186/s13013-016-0076-9
Bruniera CAV, 2013, BRAZ J PHYS THER, V17, P464, DOI 10.1590/S1413-35552012005000119
Calcaterra V, 2013, INT J CARDIOL, V168, P1614, DOI 10.1016/j.ijcard.2013.01.024
COBB JR, 1958, J BONE JOINT SURG AM, V40, P507, DOI 10.2106/00004623-195840030-00002
Correale L, 2021, MOTOR CONTROL, V25, P89, DOI 10.1123/mc.2020-0060
Duarte M, 2010, BRAZ J PHYS THER, V14, P183, DOI 10.1590/S1413-35552010000300003
Dufvenberg M, 2018, SCOLIOSIS SPINAL DIS, V13, DOI 10.1186/s13013-018-0163-1
Freyler K, 2014, GAIT POSTURE, V40, P291, DOI 10.1016/j.gaitpost.2014.04.186
HERMAN R, 1985, SPINE, V10, P1, DOI 10.1097/00007632-198501000-00001
Konieczny MR, 2013, J CHILD ORTHOP, V7, P3, DOI 10.1007/s11832-012-0457-4
Kubat O, 2020, ANN TRANSL MED, V8, DOI 10.21037/atm.2019.10.49
Kuo FC, 2011, SPINE, V36, P810, DOI 10.1097/BRS.0b013e3181d0f80c

Le Berre M, 2017, EUR SPINE J, V26, P1638, DOI 10.1007/s00586-016-4802-z

Le Huec JC, 2019, EUR SPINE J, V28, P1889, DOI 10.1007/s00586-019-06083-1

LIDSTROM J, 1988, SPINE, V13, P1070, DOI 10.1097/00007632-198809000-00017

Lin DD, 2008, GAIT POSTURE, V28, P337, DOI 10.1016/j.gaitpost.2008.01.005

Lovecchio N, 2017, J MOTOR BEHAV, V49, P123, DOI 10.1080/00222895.2016.1152225

Masani K, 2014, GAIT POSTURE, V39, P946, DOI 10.1016/j.gaitpost.2013.12.008

Negrini S, 2018, SCOLIOSIS SPINAL DIS, V13, DOI 10.1186/s13013-017-0145-8

Pagnacco G, 2015, GAIT POSTURE, V41, P136, DOI 10.1016/j.gaitpost.2014.09.016

Paillard T, 2015, BIOMED RES INT, V2015, DOI 10.1155/2015/891390

Pasha S, 2016, EUR SPINE J, V25, P3234, DOI 10.1007/s00586-016-4661-7

Perinetti Giuseppe, 2012, Prog Orthod, V13, P273, DOI 10.1016/j.pio.2011.12.003

Risser JC, 2010, CLIN ORTHOP RELAT R, V468, P646, DOI 10.1007/s11999-009-1096-z

Ruhe A, 2010, GAIT POSTURE, V32, P436, DOI 10.1016/j.gaitpost.2010.09.012

Sanders A.E., 2018, SPINE DEFORM, V6, P435, DOI [10.1016/j.jspd.2017.12.014, DOI 10.1016/J.JSPD.2017.12.014]

Saripalle SK, 2014, HUM MOVEMENT SCI, V33, P238, DOI 10.1016/j.humov.2013.09.004

Sforza C, 2003, PERCEPT MOTOR SKILL, V96, P127, DOI 10.2466/PMS.96.1.127-136

Sim T, 2018, J NEUROENG REHABIL, V15, DOI 10.1186/s12984-018-0395-6

Taylor MR, 2015, J APPL BIOMECH, V31, P324, DOI 10.1123/jab.2014-0279

Tones M, 2006, SPINE, V31, P3027, DOI 10.1097/01.brs.0000249555.87601.fc

Vialle R, 2005, J BONE JOINT SURG AM, V87A, P260, DOI 10.2106/JBJS.D.02043

WINTER DA, 1990, MED PROG TECHNOL, V16, P31

NR 36

TC 3

Z9 3

U1 2

U2 4

PU MDPI

PI BASEL

PA ST ALBAN-ANLAGE 66, CH-4052 BASEL, SWITZERLAND

EI 2077-0383

J9 J CLIN MED

JI J. Clin. Med.

PD JUL

PY 2021

VL 10

IS 13

AR 2837

DI 10.3390/jcm10132837

PG 7

WC Medicine, General & Internal

WE Science Citation Index Expanded (SCI-EXPANDED)

SC General & Internal Medicine

GA TG0DA

UT WOS:000671082400001

PM 34198971

OA gold, Green Published

DA 2023-08-10

ER

PT J

AU Li, KP

Miao, J

Zhang, JG

AF Li, Kepeng

Miao, Jun

Zhang, Jingan

TI Network meta-analysis of short-term effects of different strategies in

the conservative treatment of AIS

SO EUROPEAN JOURNAL OF MEDICAL RESEARCH

LA English

DT Review

DE Adolescent idiopathic scoliosis; Brace; Scoliosis-specific exercises

ID ADOLESCENT IDIOPATHIC SCOLIOSIS; CURVE PROGRESSION; EXERCISES

AB Purpose To evaluate the short-term effects of different conservative treatments on in adolescent idiopathic scoliosis. Methods By searching the relevant literature of adolescent idiopathic scoliosis, the curative effects of the three regimens of bracing therapy combined with scoliosis-specific exercises, simple treatment with brace and simple scoliosis-specific exercises were compared. Review manager 5.3, Stata MP16 and Network software packages were used for Reticular Meta-analysis of Cobb's angles before and after treatment. Results A total of 364 patients were included in four clinical studies. Reticular meta-analysis showed that the short-term effect of bracing treatment combined with scoliosis-specific exercises was better than that of treatment with brace and scoliosis-specific exercises, with effects of 2.71(95% CI 0.83-4.58) and 3.67(95% CI 1.21-6.14), respectively. There was no statistical difference between simple bracing therapy and scoliosis-specific exercises. Conclusion Among the three common conservative treatments of adolescent idiopathic scoliosis, the short-term effect of bracing treatment combined with scoliosis-specific exercises is better than that of bracing treatment or scoliosis-specific exercises.

C1 [Li, Kepeng] Second Cent Hosp Baoding, 57 Fan Yang Middle Rd, Baoding, Hebei, china.

[Miao, Jun; Zhang, Jingan] Tianjin Hosp, 406 Jiefang South Rd, Tianjin, china.

RP Miao, J (通讯作者)， Tianjin Hosp, 406 Jiefang South Rd, Tianjin, china.

EM likepeng19801@163.com

CR Berdishevsky H, 2016, SCOLIOSIS SPINAL DIS, V11, DOI 10.1186/s13013-016-0076-9

Day Joseph M, 2019, Arch Physiother, V9, P8, DOI 10.1186/s40945-019-0060-9

Dolan LA, 2019, SPINE DEFORM, V7, P890, DOI 10.1016/j.jspd.2019.01.011

El Hawary R, 2019, SPINE J, V19, P1917, DOI 10.1016/j.spinee.2019.07.008

el-Sayyad M, 1994, Int J Rehabil Res, V17, P70, DOI 10.1097/00004356-199403000-00008

Gao CF, 2019, AM J PHYS MED REHAB, V98, P642, DOI 10.1097/PHM.0000000000001160

Kuru T, 2016, CLIN REHABIL, V30, P181, DOI 10.1177/0269215515575745

Mao SH, 2016, EUR SPINE J, V25, P500, DOI 10.1007/s00586-015-3937-7

Menger RP., 2020, ADOLESCENT IDIOPATHI

Negrini S, 2019, ANN PHYS REHABIL MED, V62, P69, DOI 10.1016/j.rehab.2018.07.010

Park JH, 2018, EUR J PHYS REHAB MED, V54, P440, DOI 10.23736/S1973-9087.17.04461-6

Roye BD, 2020, SPINE DEFORM, V8, P597, DOI 10.1007/s43390-020-00060-1

Schreiber S, 2017, SCOLIOSIS SPINAL DIS, V12, DOI 10.1186/s13013-017-0137-8

Schreiber S, 2016, PLOS ONE, V11, DOI 10.1371/journal.pone.0168746

Thompson JY, 2019, PHYSIOTHERAPY, V105, P214, DOI 10.1016/j.physio.2018.10.004

Trac S, 2019, SPINE DEFORM, V7, P18, DOI 10.1016/j.jspd.2018.06.015

van den Bogaart M, 2019, EUR SPINE J, V28, P511, DOI 10.1007/s00586-018-05870-6

Weniger CD, 2019, KLIN PADIATR, V231, P248, DOI 10.1055/a-0963-8996

Zapata KA, 2019, PEDIATR PHYS THER, V31, P280, DOI 10.1097/PEP.0000000000000621

Zheng Y, 2018, SPINE, V43, pE494, DOI 10.1097/BRS.0000000000002412

NR 20

TC 3

Z9 4

U1 3

U2 41

PU BMC

PI LONDON

PA CAMPUS, 4 CRINAN ST, LONDON N1 9XW, united kingdom

SN 0949-2321

EI 2047-783X

J9 EUR J MED RES

JI Eur. J. Med. Res.

PD JUN 13

PY 2021

VL 26

IS 1

AR 54

DI 10.1186/s40001-021-00526-6

PG 7

WC Medicine, Research & Experimental

WE Science Citation Index Expanded (SCI-EXPANDED)

SC Research & Experimental Medicine

GA SV4HK

UT WOS:000663781200002

PM 34120641

OA gold, Green Published

DA 2023-08-10

ER

PT J

AU Bachmann, KR

AF Bachmann, Keith R.

TI Spinal Deformities in the Adolescent Athlete

SO CLINICS IN SPORTS MEDICINE

LA English

DT Article

DE Scoliosis; Return to sport; Down syndrome; Posterior spinal fusion;

Adolescents

ID QUALITY-OF-LIFE; IDIOPATHIC SCOLIOSIS; FOLLOW-UP; PHYSICAL-ACTIVITIES;

RETURN; CHILDREN; SURGERY; FUSION; BRACE; INDIVIDUALS

C1 [Bachmann, Keith R.] Univ Virginia, Dept Orthopaed Surg, POB 800159, Charlottesville, VA 22908 USA.

C3 University of Virginia

RP Bachmann, KR (通讯作者)， Univ Virginia, Dept Orthopaed Surg, POB 800159, Charlottesville, VA 22908 USA.

EM kbachmann.uva@gmail.com

CR Altaf F, 2017, SPINE DEFORM, V5, P303, DOI [10.1016/j.jspd.2017.03.009, DOI 10.1016/J.JSPD.2017.03.009]

Bouchard M, 2019, SPINE DEFORM, V7, P950, DOI 10.1016/j.jspd.2019.01.012

Bull MJ, 2011, PEDIATRICS, V128, P393, DOI 10.1542/peds.2011-1605

Danielsson AJ, 2007, SPINE, V32, P2198, DOI 10.1097/BRS.0b013e31814b851f

Danielsson AJ, 2010, SPINE, V35, P199, DOI 10.1097/BRS.0b013e3181c89f4a

Danielsson AJ, 2001, EUR SPINE J, V10, P278, DOI 10.1007/s005860100309

Diarbakerli E, 2016, SCOLIOSIS SPINAL DIS, V11, DOI 10.1186/s13013-016-0082-y

Diaz MCG, 2019, AM J MED QUAL, V34, P182, DOI 10.1177/1062860618792667

Diebo BG, 2019, SPINE DEFORM, V7, P559, DOI 10.1016/j.jspd.2018.11.014

Dunn J, 2018, JAMA-J AM MED ASSOC, V319, P173, DOI 10.1001/jama.2017.11669

Fabricant PD, 2012, J PEDIATR ORTHOPED, V32, P259, DOI 10.1097/BPO.0b013e31824b285f

Fong DYT, 2015, SPINE J, V15, P825, DOI 10.1016/j.spinee.2015.01.019

Fong DYT, 2010, SPINE, V35, P1061, DOI 10.1097/BRS.0b013e3181bcc835

Gabos PG, 2004, J BONE JOINT SURG AM, V86A, P1891, DOI 10.2106/00004623-200409000-00006

Green BN, 2009, J CHIROPR MED, V8, P25, DOI 10.1016/j.jcm.2008.11.001

Grossman DC, 2018, JAMA-J AM MED ASSOC, V319, P165, DOI 10.1001/jama.2017.19342

Hines T, 2015, SPINE, V40, pE1135, DOI 10.1097/BRS.0000000000001040

Kadhim M, 2020, PUBLIC HEALTH, V178, P72, DOI 10.1016/j.puhe.2019.08.020

Kakar RS, 2019, SPINE DEFORM, V7, P254, DOI 10.1016/j.jspd.2018.08.015

Kakar RS, 2018, SPINE J, V18, P155, DOI 10.1016/j.spinee.2017.08.221

Kakar Rumit Singh, 2017, Int J Exerc Sci, V10, P166

Karachalios T, 1999, SPINE, V24, P2318, DOI 10.1097/00007632-199911150-00006

Lehman RA, 2015, SPINE J, V15, P951, DOI 10.1016/j.spinee.2013.06.035

Linker B, 2012, AM J PUBLIC HEALTH, V102, P606, DOI 10.2105/AJPH.2011.300531

Marks M, 2012, SPINE, V37, P826, DOI 10.1097/BRS.0b013e31823b4eab

Misterska E, 2017, PLOS ONE, V12, P1

Negrini S, 2012, STUD HEALTH TECHNOL, V176, P437, DOI 10.3233/978-1-61499-067-3-437

OrthoInfo, SURG TREATM SCOL

Piantoni L, 2018, SCOLIOSIS SPINAL DIS, V13, P1

Rubery PT, 2002, SPINE, V27, P423, DOI 10.1097/00007632-200202150-00019

Sarwahi V, 2018, SPINE, V43, P167, DOI 10.1097/BRS.0000000000002284

Segreto FA, 2019, J PEDIATR ORTHOP B, V28, P356, DOI 10.1097/BPB.0000000000000574

Society SR, SURG AD ID SCOL FAQ

Tarrant RC, 2014, SPINE, V39, P1471, DOI 10.1097/BRS.0000000000000452

Thomas JJ., 2018, SPINE DEFORM, V6, P403, DOI [10.1016/j.jspd.2017.12.007, DOI 10.1016/J.JSPD.2017.12.007]

Thompson JY, 2019, PHYSIOTHERAPY, V105, P214, DOI 10.1016/j.physio.2018.10.004

Watanabe K, 2017, J BONE JOINT SURG AM, V99, P284, DOI 10.2106/JBJS.16.00459

Weinstein SL, 2019, J PEDIATR ORTHOPED, V39, pS44, DOI 10.1097/BPO.0000000000001350

Weinstein SL, 2013, NEW ENGL J MED, V369, P1512, DOI 10.1056/NEJMoa1307337

Yawn BP, 1999, JAMA-J AM MED ASSOC, V282, P1427, DOI 10.1001/jama.282.15.1427

Yawn BP, 2000, SPINE, V25, P2387, DOI 10.1097/00007632-200009150-00019

Zapata KA, 2019, PEDIATR PHYS THER, V31, P280, DOI 10.1097/PEP.0000000000000621

NR 42

TC 1

Z9 1

U1 1

U2 4

PU W B SAUNDERS CO-ELSEVIER INC

PI PHILADELPHIA

PA 1600 JOHN F KENNEDY BOULEVARD, STE 1800, PHILADELPHIA, PA 19103-2899 USA

SN 0278-5919

EI 1556-228X

J9 CLIN SPORT MED

JI Clin. Sports Med.

PD JUL

PY 2021

VL 40

IS 3

BP 541

EP 554

DI 10.1016/j.csm.2021.03.007

EA MAY 2021

PG 14

WC Sport Sciences

WE Science Citation Index Expanded (SCI-EXPANDED); Social Science Citation Index (SSCI)

SC Sport Sciences

GA SJ1AZ

UT WOS:000655262800008

PM 34051945

DA 2023-08-10

ER

PT J

AU Fang, MQ

Huang, XL

Wang, W

Li, YA

Xiang, GH

Yan, GK

Ke, CR

Mao, CH

Wu, ZY

Pan, TL

Zhu, RB

Xiao, J

Yi, XH

AF Fang, Ming-Qiao

Huang, Xiao-Li

Wang, Wei

Li, Yu-An

Xiang, Guang-Heng

Yan, Guang-Kui

Ke, Chen-Rong

Mao, Cheng-Huang

Wu, Zong-Yi

Pan, Tian-Long

Zhu, Rui-Bo

Xiao, Jian

Yi, Xian-Hong

TI The efficacy of Schroth exercises combined with the Cheneau brace for

the treatment of adolescent idiopathic scoliosis: a retrospective

controlled study

SO DISABILITY AND REHABILITATION

LA English

DT Article

DE Adolescent idiopathic scoliosis; Chê neau brace; Schroth exercises;

coronal balance; sagittal alignment; health-related quality of life

ID QUALITY-OF-LIFE; RANDOMIZED CONTROLLED-TRIAL; DEFORMITY; FLATBACK;

CRITERIA; SRS

AB Purpose We aimed to determine the global effects of the Cheneau brace combined with Schroth exercises on adolescent idiopathic scoliosis (AIS). Methods We analyzed 192 patients with AIS who underwent the Cheneau brace treatment alone or combined with Schroth best practice (SBP) from June 2013 to October 2019. There were 138 patients in the Brace group and 54 patients in the Brace + SBP group. Radiographs were obtained at various treatment durations. Answers to the health-related quality of life (HRQoL) questionnaire were recorded before the intervention and at the time of treatment wean. Results The Cobb angle (-3.55 degrees; $p < 0.001$) and C7-CSVL (-3.03 mm; $p < 0.001$) significantly decreased in the Brace + SBP group. Thoracic kyphosis (TK) decreased in both the Brace + SBP group (-1.85 degrees; $p = 0.0152$) and the Brace group (-5.06; $p < 0.001$). Changes

before and after treatment of TK were significantly different between groups (p < 0.001). The 22-item Scoliosis Research Society function score, self-image, mental health, and EuroQol 5-Dimension scores were significantly higher in the Brace + SBP group. The satisfaction score was higher in the Brace + SBP group (3.77 +/- 0.63 vs. 3.13 +/- 0.79; p < 0.001). Conclusions Compared to bracing alone, the Schroth exercises plus bracing had a better effect on coronal balance. Schroth exercises improve flatback deformity caused by bracing and positively influence the HRQoL in AIS patients who received the Cheneau brace treatment.

C1 [Fang, Ming-Qiao; Wang, Wei; Li, Yu-An; Xiang, Guang-Heng; Yan, Guang-Kui; Ke, Chen-Rong; Mao, Cheng-Huang; Wu, Zong-Yi; Pan, Tian-Long; Zhu, Rui-Bo; Xiao, Jian; Yi, Xian-Hong] Wenzhou Med Univ, Dept Orthopaed Surg, Affiliated Hosp 2, Wenzhou 325027, Zhejiang, china.

[Fang, Ming-Qiao; Huang, Xiao-Li; Wang, Wei; Li, Yu-An; Xiang, Guang-Heng; Yan, Guang-Kui; Ke, Chen-Rong; Mao, Cheng-Huang; Wu, Zong-Yi; Pan, Tian-Long; Zhu, Rui-Bo; Xiao, Jian; Yi, Xian-Hong] Wenzhou Med Univ, Yuying Childrens Hosp, Wenzhou 325027, Zhejiang, china.

[Fang, Ming-Qiao; Xiang, Guang-Heng; Xiao, Jian] Wenzhou Med Univ, Sch Pharmaceut Sci, Mol Pharmacol Res Ctr, Wenzhou 325000, china.

[Huang, Xiao-Li] Wenzhou Med Univ, Dept Rehabil, Affiliated Hosp 2, Wenzhou, china.

C3 Wenzhou Medical University; Wenzhou Medical University; Wenzhou Medical

University; Wenzhou Medical University

RP Yi, XH (通讯作者)， Wenzhou Med Univ, Dept Orthopaed Surg, Affiliated Hosp 2, Wenzhou 325027, Zhejiang, china.; Yi, XH (通讯作者)， Wenzhou Med Univ, Yuying Childrens Hosp, Wenzhou 325027, Zhejiang, china.; Xiao, J (通讯作者)， Wenzhou Med Univ, Sch Pharmaceut Sci, Mol Pharmacol Res Ctr, Wenzhou 325000, china.

EM xfxj2000@126.com; wzfeyyxh@163.com

FU Wenzhou Municipal Science and Technology Bureau [Y2020389]

FX This research was funded by a grant from Wenzhou Municipal Science and

Technology Bureau (Y2020389).

CR [Anonymous], 1992, PHYSIOTHERAPY

Blondel B, 2012, NEUROSURGERY, V71, P341, DOI 10.1227/NEU.0b013e31825d20c0

Boody BS, 2017, CLIN SPINE SURG, V30, P142, DOI 10.1097/BSD.0000000000000522

Borysov M, 2016, CURR PEDIATR REV, V12, P31, DOI 10.2174/1573396312666151117120746

Canavese F, 2011, INDIAN J ORTHOP, V45, P7, DOI 10.4103/0019-5413.73655

Ch?neau J., 1994, CORSET CH NEAU MANUE

Cheung JPY, 2019, BONE JOINT J, V101B, P1370, DOI 10.1302/0301-620X.101B11.BJJ-2019-0515.R1

Cheung PWH, 2019, SPINE, V44, pE596, DOI 10.1097/BRS.0000000000002918

De Giorgi S, 2013, EUR SPINE J, V22, pS815, DOI 10.1007/s00586-013-3020-1

Fan HW, 2016, SPINE, V41, P259, DOI 10.1097/BRS.0000000000001197

Fang MQ, 2015, J NEUROSURG-SPINE, V23, P505, DOI 10.3171/2015.2.SPINE14970

Kotwicki T, 2008, DISABIL REHABIL-ASSI, V3, P146, DOI 10.1080/17483100801905744

Kuru T, 2016, CLIN REHABIL, V30, P181, DOI 10.1177/0269215515575745

Lenhert-Schroth C., 2007, SCHROTH SCOLIOSIS 3

Lenhert-Schroth C., 1992, PHYSIOTHERAPY, V78, P810, DOI [10.1016/S0031-9406(10)60451-8, DOI 10.1016/S0031-9406(10)60451-8]

Lonner BS, 2012, SPINE, V37, P1297, DOI 10.1097/BRS.0b013e318247e9a6

Makino T, 2019, SPINE, V44, pE833, DOI 10.1097/BRS.0000000000002996

Monticone M, 2014, EUR SPINE J, V23, P1204, DOI 10.1007/s00586-014-3241-y

NACHEMSON AL, 1995, J BONE JOINT SURG AM, V77A, P815, DOI 10.2106/00004623-199506000-00001

Negrini S, 2005, Eura Medicophys, V41, P183

Negrini Stefano, 2003, Pediatr Rehabil, V6, P227, DOI: 10.1080/13638490310001636781

Negrini S, 2014, BMC MUSCULOSKEL DIS, V15, DOI 10.1186/1471-2474-15-263

Noh DK, 2014, J BACK MUSCULOSKELET, V27, P331, DOI 10.3233/BMR-130452

Otman S, 2005, SAUDI MED J, V26, P1429

Park JH, 2018, EUR J PHYS REHAB MED, V54, P440, DOI 10.23736/S1973-9087.17.04461-6

Richards BS, 2005, SPINE, V30, P2068, DOI 10.1097/01.brs.0000178819.90239.d0

Rigo M, 2009, STUD HEALTH TECHNOL, V135, P303

Schmitz A, 2001, J Orthop Sci, V6, P316, DOI 10.1007/s007760100025

Schreiber S, 2017, SCOLIOSIS SPINAL DIS, V12, DOI 10.1186/s13013-017-0137-8

Schreiber S, 2016, PLOS ONE, V11, DOI 10.1371/journal.pone.0168746

Schreiber S, 2015, SCOLIOSIS SPINAL DIS, V10, DOI 10.1186/s13013-015-0048-5

VONDEIMLING U, 1995, Z ORTHOP GRENZGEB, V133, P270, DOI 10.1055/s-2008-1039447

Weinstein SL, 2008, LANCET, V371, P1527, DOI 10.1016/S0140-6736(08)60658-3

Weinstein SL, 2013, NEW ENGL J MED, V369, P1512, DOI 10.1056/NEJMoa1307337

Weiss Hans-Rudolf, 2003, Pediatr Rehabil, V6, P183

Weiss HR, 2009, STUD HEALTH TECHNOL, V135, P164

Wimmer C., 2003, MED ORTHOP TECH, V123, P33

Zaina F, 2009, SCOLIOSIS SPINAL DIS, V4, DOI 10.1186/1748-7161-4-8

NR 38

TC 3

Z9 3

U1 2

U2 19

PU TAYLOR & FRANCIS LTD

PI ABINGDON

PA 2-4 PARK SQUARE, MILTON PARK, ABINGDON OR14 4RN, OXON, united kingdom

SN 0963-8288

EI 1464-5165

J9 DISABIL REHABIL

JI Disabil. Rehabil.

PD AUG 28

PY 2022

VL 44

IS 18

BP 5060

EP 5068

DI 10.1080/09638288.2021.1922521

EA MAY 2021

PG 9

WC Rehabilitation

WE Science Citation Index Expanded (SCI-EXPANDED); Social Science Citation Index (SSCI)

SC Rehabilitation

GA 4K6SD

UT WOS:000650502600001

PM 33984249

DA 2023-08-10

ER

PT J

AU Zhou, ZW

Liu, F

Li, R

Chen, XR

AF Zhou, Ziwei

Liu, Fang

Li, Ru

Chen, Xiaorong

TI The effects of exercise therapy on adolescent idiopathic scoliosis: An

overview of systematic reviews and meta-analyses

SO COMPLEMENTARY THERAPIES IN MEDICINE

LA English

DT Article

DE Exercise; Adolescents; Scoliosis; Review; Meta-analysis

ID QUALITY-OF-LIFE; PROSPECTIVE-CONTROLLED-COHORT; CORE STABILIZATION EXERCISE; LOW-BACK-PAIN; SEAS.02 EXERCISES; SCHROTH EXERCISES; COBB ANGLE; STRENGTH; EFFICACY; REDUCE

AB Background: Exercise therapy was suggested as an appealing treatment option for Adolescent Idiopathic Scoliosis (AIS) patients with less side effects, lower economic costs, and more psychological benefits. Nevertheless, no unanimous findings about the efficacy of exercise therapy have been obtained from previous systematic reviews and/or meta-analyses.

Objective: To provide an overview of previous systematic reviews and/or meta-analyses on the effectiveness of exercise therapy on AIS treatment.

Methods: Systematic searches in Medline, Eric, CINAHL, Embase, SPORTDiscus, PsycINFO, and the Cochrane Library for systematic reviews and/or meta-analyses of randomized controlled trials (RCTs), non-randomized comparison studies (NRS) or observational studies using exercise as an intervention, and with outcome measures including Cobb angle, angle of trunk rotation (ATR), and quality of life. The methodological quality of the review articles was evaluated by A Measurement Tool to Assess Systematic Reviews (AMSTAR) checklist. Results: Ten systematic reviews and meta-analyses were included. The quality of most of the review articles is moderate with a mean score of 6/11 on the AMSTAR scale. Overall, there is increasing evidence showing the efficacy of exercise therapy on reducing the Cobb angle and angle of trunk rotation, and improving perceived quality of life.

Conclusion: Exercise therapy was found to have potential benefits to treat physiological and psychological aspects of AIS patients. However, the findings were not conclusive given that some reviews relied on data from the trials with potential risk of bias and significant heterogeneity. More high-quality research is still needed to verify these findings.

C1 [Zhou, Ziwei; Liu, Fang; Li, Ru; Chen, Xiaorong] Shenzhen Univ, Fac Phys Educ, Shenzhen 518060, china.

C3 Shenzhen University

RP Li, R (通讯作者)， Shenzhen Univ, Fac Phys Educ, Shenzhen 518060, china.

EM 1800371014@email.szu.edu.cn; 1800371006@email.szu.edu.cn; lirutracy@szu.edu.cn; cxr819@163.com

OI Li, Ru/0000-0002-9756-793X; zhou, zi wei/0000-0001-5836-2811

FU Natural Science Funds of Shenzhen University

FX This work was supported by the Natural Science Funds of Shenzhen University. The funders had no involvement in the design of the study, analysis and interpretation of data, decision to publish, or preparation of the manuscript.

CR Alayat MSM, 2017, J PHYS THER SCI, V29

Altaf F, 2013, BMJ-BRIT MED J, V346, DOI 10.1136/bmj.f2508

[Anonymous], 1991, P BOOK 11 INT C WORL

[Anonymous], 2005, ZHONGGUO LINCHUANG K

[Anonymous], 1985, P INT C PREV SCOL SC

[Anonymous], 2001, OXFORD CTR EVIDENCE

Anwer S, 2015, BIOMED RES INT-UK, V2015, DOI 10.1155/2015/123848

Berdishevsky H, 2016, SCOLIOSIS SPINAL DIS, V11, DOI 10.1186/s13013-016-0076-9

Burger M, 2019, SOUTH AFR J PHYSIOTH, V75, DOI 10.4102/sajp.v75i1.904

Laita LC, 2018, ARCH ARGENT PEDIATR, V116, pE582, DOI [10.5546/aap.2018.e582, 10.5546/aap.2018.eng.e582]

Cheon M, 2013, EURASIP J WIREL COMM, DOI 10.1186/1687-1499-2013-132

Day Joseph M, 2019, Arch Physiother, V9, P8, DOI 10.1186/s40945-019-0060-9

den Boer WA, 1999, EUR SPINE J, V8, P406, DOI 10.1007/s005860050195

Diab AA, 2012, CLIN REHABIL, V26, P1123, DOI 10.1177/0269215512447085

Dobosiewicz Krystyna, 2005, Ortop Traumatol Rehabil, V7, P49

Ducong P, 2002, RESONANCE EUROPEENNE, V10, P1229

Durmala Jacek, 2003, Ortop Traumatol Rehabil, V5, P80

Farooqui Sumaira Imran, 2018, Int J Health Sci (Qassim), V12, P44

Ferraro C., 1998, EUR MEDICOPHYS, V34, P25

Fusco C., 2011, Physiotherapy Theory and Practice, V27, P80, DOI 10.3109/09593985.2010.533342

Gao CF, 2019, AM J PHYS MED REHAB, V98, P642, DOI 10.1097/PHM.0000000000001160

Gur G, 2017, PROSTHET ORTHOT INT, V41, P303, DOI 10.1177/0309364616664151

jn lee woo, 2017, [The Journal of Korean Society of Physical Therapy, 대한물리치료학회지], V29, P276

Kim G, 2016, J PHYS THER SCI, V28, P1012, DOI 10.1589/jpts.28.1012

Kim M-J, 2017, PHYS THER REHABIL SC, V6

Ko KJ, 2017, J EXERC REHABIL, V13, P244, DOI 10.12965/jer.1734952.476

Kumar A, 2017, J CLIN DIAGN RES, V11, pYC1, DOI 10.7860/JCDR/2017/27497.10335

Kuru T, 2016, CLIN REHABIL, V30, P181, DOI 10.1177/0269215515575745

Kwan KYH, 2017, SCOLIOSIS SPINAL DIS, V12, DOI 10.1186/s13013-017-0139-6

Langensiepen S, 2017, J MUSCULOSKEL NEURON, V17, P259

Lewis C, 2012, PHYSICAL THERAPY PERSPECTIVES IN THE 21ST CENTURY - CHALLENGES AND POSSIBILITIES, P67

Liberati A, 2009, BMJ-BRIT MED J, V339, DOI [10.1136/bmj.b2700, 10.1136/bmj.b4037, 10.1371/journal.pmed.1000097, 10.7326/0003-4819-151-4-200908180-00136]

Liu DL, 2020, SPINE, V45, P1039, DOI 10.1097/BRS.0000000000003451

Lonstein JE, 2006, CLIN ORTHOP RELAT R, P248, DOI 10.1097/01.blo.0000198725.54891.73

Mamyama T, 2003, SIDE SHIFT EXERCISE

Mamyama Toni, 2002, Stud Health Technol Inform, V91, P361

Mcintire K, 2006, ST HEAL T, V123, P273

Mollon G, 1986, KINESITHERAPIE SCI, V244, P47

Monticone M, 2014, EUR SPINE J, V23, P1204, DOI 10.1007/s00586-014-3241-y

Mooney V, 2000, J SPINAL DISORD, V13, P102, DOI 10.1097/00002517-200004000-00002

Negrini S, 2008, DISABIL REHABIL, V30, P772, DOI 10.1080/09638280801889568

Negrini S, 2014, EUR J PHYS REHAB MED, V50, P83

Negrini S, 2008, J REHABIL MED, V40, P451, DOI 10.2340/16501977-0195

Negrini S, 2006, ST HEAL T, V123, P523

Negrini S, 2006, ST HEAL T, V123, P519

Negrini S, 2006, SCOLIOSIS SPINAL DIS, V1, DOI 10.1186/1748-7161-1-4

Negrini Stefano, 2003, Pediatr Rehabil, V6, P227, DOI: 10.1080/13638490310001636781

Negrini S, 2019, ANN PHYS REHABIL MED, V62, P69, DOI 10.1016/j.rehab.2018.07.010

Negrini S, 2018, SCOLIOSIS SPINAL DIS, V13, DOI 10.1186/s13013-017-0145-8

Negrini S, 2012, STUD HEALTH TECHNOL, V176, P433, DOI 10.3233/978-1-61499-067-3-433

Noh DK, 2014, J BACK MUSCULOSKELET, V27, P331, DOI 10.3233/BMR-130452

Otman Saadet, 2005, Neurosciences (Riyadh), V10, P277

Reamy BV, 2001, AM FAM PHYSICIAN, V64, P111

Romano M, 2012, COCHRANE DB SYST REV, DOI 10.1002/14651858.CD007837.pub2

Schreiber S, 2016, PLOS ONE, V11, DOI 10.1371/journal.pone.0168746

Schreiber S, 2015, SCOLIOSIS SPINAL DIS, V10, DOI 10.1186/s13013-015-0048-5

Scully D, 1998, BRIT J SPORT MED, V32, P111, DOI 10.1136/bjsm.32.2.111

Sharif Mohammad O, 2013, Oral Health Dent Manag, V12, P9

Shea BJ, 2009, J CLIN EPIDEMIOL, V62, P1013, DOI 10.1016/j.jclinepi.2008.10.009

STONE B, 1979, PHYS THER, V59, P759, DOI 10.1093/ptj/59.6.759

Thompson JY, 2019, PHYSIOTHERAPY, V105, P214, DOI 10.1016/j.physio.2018.10.004

To MKT., 2020, BMC MUSCULOSKEL DIS, V21, P1

Toledo Pollyana Coelho Vieira, 2011, Fisioter. Pesqui., V18, P329

Weinstein SL, 2003, JAMA-J AM MED ASSOC, V289, P559, DOI 10.1001/jama.289.5.559

Weinstein SL, 2008, LANCET, V371, P1527, DOI 10.1016/S0140-6736(08)60658-3

Weiss HR, 2008, EUR J PHYS REHAB MED, V44, P177

Weiss H R, 1992, Ital J Orthop Traumatol, V18, P395

Weiss H R, 1997, Pediatr Rehabil, V1, P35

Weiss H-R, 2006, Pediatr Rehabil, V9, P190, DOI 10.1080/13638490500079583

Weiss Hans-Rudolf, 2003, Pediatr Rehabil, V6, P23, DOI 10.1080/1363849031000095288

Weiss HR, 2002, ST HEAL T, V88, P304

Yagci G, 2019, PROSTHET ORTHOT INT, V43, P301, DOI 10.1177/0309364618820144

Yagci G, 2018, J BACK MUSCULOSKELET, V31, P693, DOI 10.3233/BMR-170868

Ying Huang, 2014, CHIN J REHABI MED, V29, P81

Zaina F, 2009, SCOLIOSIS SPINAL DIS, V4, DOI 10.1186/1748-7161-4-8

Zakaria A, 2012, J PHYS THER SCI, V24, P1127, DOI 10.1589/jpts.24.1127

Zapata KA, 2017, PEDIATR PHYS THER, V29, P62, DOI 10.1097/PEP.0000000000000325

Zapata KA, 2015, PEDIATR PHYS THER, V27, P396, DOI 10.1097/PEP.0000000000000174

Zheng Y, 2018, SPINE, V43, pE494, DOI 10.1097/BRS.0000000000002412

황보필녀, 2016, [The Journal of Korean Society of Physical Therapy, 대한물리치료학회지], V28, P364

NR 80

TC 10

Z9 10

U1 3

U2 31

PU CHURCHILL LIVINGSTONE

PI EDINBURGH

PA JOURNAL PRODUCTION DEPT, ROBERT STEVENSON HOUSE, 1-3 BAXTERS PLACE, LEITH WALK, EDINBURGH EH1 3AF, MIDLOTHIAN, SCOTLAND

SN 0965-2299

EI 1873-6963

J9 COMPLEMENT THER MED

JI Complement. Ther. Med.

PD MAY

PY 2021

VL 58

AR 102697

DI 10.1016/j.ctim.2021.102697

PG 11

WC Integrative & Complementary Medicine

WE Science Citation Index Expanded (SCI-EXPANDED)

SC Integrative & Complementary Medicine

GA RX2KA

UT WOS:000647049100028

PM 33636298

OA gold

DA 2023-08-10

ER

PT J

AU de Assis, SJC

Sanchis, GJB

de Souza, CG

Roncalli, AG

AF Costa de Assis, Sanderson Jose

Bouzas Sanchis, Geronimo Jose

de Souza, Clecio Gabriel

Roncalli, Angelo Giuseppe

TI Influence of physical activity and postural habits in schoolchildren

with scoliosis

SO ARCHIVES OF PUBLIC HEALTH

LA English

DT Article

DE Posture; Exercise; Risk factors

ID ADOLESCENT IDIOPATHIC SCOLIOSIS; PAIN; CHILDREN; MUSCULOSKELETAL;

ASSOCIATION; PREVALENCE; COMPUTER; RISK

AB BackgroundScoliosis is considered one of the main musculoskeletal changes in childhood, and is characterized by three-dimensional changes in the spine. Schoolchildren is a group who are directly exposed to this condition because they go through a rapid growth phase in adolescence, added to other external factors such as school environment and daily living habits such as little physical activity. This study aimed to identify the risk factors associated with scoliosis in schoolchildren.MethodsAn observational, retrospective case control study with a quantitative approach was carried out in the city of Santa Cruz/RN. The presence of scoliosis was assessed using the Adams test and physical activity by the Daily Physical Activity Index (IPAQ) and by a questionnaire on competitive sports practice, in addition to a questionnaire on postural habits in childhood and adolescence. Conditional multiple logistic regression was performed for statistical analysis, and the adjusted Odds Ratios (OR) and the respective confidence intervals (95%) of the outcome variable were estimated.ResultsA total of 156 schoolchildren participated in the study, with an average age of 13.9years, with 55.1% being female and 44.9% male, attending between the 6th grade of elementary school and the 3rd year of high school. Furthermore, 42.9% of these participants were considered irregularly active and only 33.3% practiced physical activity on a regular basis. After bivariate analysis and conditional logistic regression, little physical activity was shown to be a risk factor for scoliosis (p=0.041; OR: 2.81; 95% CI: 1.04-7.57), while the postural habits evaluated in this study did not show a statistical association with scoliosis.ConclusionLow practice of physical activity and schoolchildren being classified as irregularly active were considered as risk factors for scoliosis, however postural habits do not seem to be associated with this condition.

C1 [Costa de Assis, Sanderson Jose; Bouzas Sanchis, Geronimo Jose; Roncalli, Angelo Giuseppe] Univ Fed Rio Grande do Norte, Postgrad Program Publ Hlth, Natal, RN, Brazil.

[de Souza, Clecio Gabriel] Univ Fed Rio Grande do Norte, Postgrad Program Rehabil Sci, Santa Cruz, RN, Brazil.

C3 Universidade Federal do Rio Grande do Norte; Universidade Federal do Rio

Grande do Norte

RP de Assis, SJC (通讯作者)， Univ Fed Rio Grande do Norte, Postgrad Program Publ Hlth, Natal, RN, Brazil.

EM sanderson_assis@hotmail.com

OI Assis, Sanderson/0000-0002-7149-2335; Bouzas Sanchis, Geronimo

Jose/0000-0002-7320-5520

CR Baroni MP, 2015, J EPIDEMIOL, V25, P212, DOI 10.2188/jea.JE20140061

Bergmann GG, 2013, CAD SAUDE PUBLICA, V29, P2217, DOI [10.1590/0102-311X00077512, 10.1590/0102-311x00077512]

Bueno Rita de Cássia de S., 2013, Rev. paul. pediatr., V31, P237

Burwell RG, 2016, SCOLIOSIS SPINAL DIS, V11, DOI 10.1186/s13013-016-0063-1

Chen C, 2020, SPINE, V45, pE266, DOI 10.1097/BRS.0000000000003256

Coenen P, 2018, SCAND J WORK ENV HEA, V44, P521, DOI 10.5271/sjweh.3744

Craig CL, 2003, MED SCI SPORT EXER, V35, P1381, DOI 10.1249/01.MSS.0000078924.61453.FB

Damasceno GM, 2018, EUR SPINE J, V27, P1249, DOI 10.1007/s00586-017-5444-5

Espírito Santo Alcebíades do, 2011, Rev. bras. epidemiol., V14, P347, DOI 10.1590/S1415-790X2011000200015

Ee J, 2018, INT J ENV RES PUB HE, V15, DOI 10.3390/ijerph15081668

Fan HW, 2016, SPINE, V41, P259, DOI 10.1097/BRS.0000000000001197

Ferreira Dalva Minonroze Albuquerque, 2013, Fisioter. mov., V26, P337

Fornazari LP., 2008, CADERNO ESCOLA SAUDE, V1, P1

Grimmer K, 2002, BMC MUSCULOSKELET DI, V3, DOI 10.1186/1471-2474-3-10

Guedes Dartagnan Pinto, 2005, Rev Bras Med Esporte, V11, P151, DOI 10.1590/S1517-86922005000200011

Howie EK, 2017, APPL ERGON, V65, P41, DOI 10.1016/j.apergo.2017.05.011

Iunes DH, 2010, BRAZ J PHYS THER, V14, P133, DOI 10.1590/S1413-35552010005000009

Limon S, 2004, SPINE, V29, P697, DOI 10.1097/01.BRS.0000116695.09697.22

McMaster ME, 2015, SCOLIOSIS SPINAL DIS, V10, DOI 10.1186/s13013-015-0029-8

Milistetd Michel, 2014, Rev. Bras. Ciênc. Esporte, V36, P671, DOI 10.1590/2179-325520143630012

Mordecai SC, 2012, EUR SPINE J, V21, P382, DOI 10.1007/s00586-011-2063-4

Patias P, 2010, SCOLIOSIS SPINAL DIS, V5, DOI 10.1186/1748-7161-5-12

Preto Leonel São Romão, 2015, Rev. Enf. Ref., VserIV, P31, DOI 10.12707/RIV14051

Rebolho MCT., 2005, THESIS U SAO PAULO S

Silva GRR, 2016, J PEDIAT-BRAZIL, V92, P188, DOI 10.1016/j.jped.2015.06.006

Rodrigues PL., 2014, BRAZIL FISIOTER MOV, V27, P437, DOI [10.1590/0103-5150.027.003.AO15, DOI 10.1590/0103-5150.027.003.AO15]

Rosanova Giselle Cristina Lopes, 2013, Fisioter. mov., V26, P63

Sedrez Juliana Adami, 2015, Rev. paul. pediatr., V33, P72, DOI 10.1016/j.rpped.2014.11.012

Silva Jaqueline Santos, 2017, Fisioter. mov., V30, P297, DOI 10.1590/1980-5918.030.002.ao10

Souza Junior José Vitorino de, 2011, Fisioter. Pesqui., V18, P311

Straker L, 2018, ERGONOMICS, V61, P658, DOI 10.1080/00140139.2017.1401671

Straker L, 2016, J PHYS ACT HEALTH, V13, P177, DOI 10.1123/jpah.2015-0026

Toh SH, 2017, PLOS ONE, V12, DOI 10.1371/journal.pone.0181220

Vieira A., 2009, MOVIMENTO-PORTO ALEG, V1, P145

Yamato TP, 2018, BRIT J SPORT MED, V52, P1241, DOI 10.1136/bjsports-2017-098927

NR 35

TC 6

Z9 7

U1 6

U2 13

PU BMC

PI LONDON

PA CAMPUS, 4 CRINAN ST, LONDON N1 9XW, united kingdom

SN 0778-7367

EI 2049-3258

J9 ARCH PUBLIC HEALTH

JI Arch. PUblic Health

PD APR 29

PY 2021

VL 79

IS 1

AR 63

DI 10.1186/s13690-021-00584-6

PG 7

WC Public, Environmental & Occupational Health

WE Science Citation Index Expanded (SCI-EXPANDED); Social Science Citation Index (SSCI)

SC Public, Environmental & Occupational Health

GA SI3WB

UT WOS:000654757000002

PM 33926556

OA Green Published, gold

DA 2023-08-10

ER

PT J

AU Kocaman, H

Bek, N

Kaya, MH

Buyukturan, B

Yetis, M

Buyukturan, O

AF Kocaman, Hikmet

Bek, Nilgun

Kaya, Mehmet Hanifi

Buyukturan, Buket

Yetis, Mehmet

Buyukturan, Oznur

TI The effectiveness of two different exercise approaches in adolescent

idiopathic scoliosis: A single-blind, randomized-controlled trial

SO PLOS ONE

LA English

DT Article

ID CORE STABILIZATION EXERCISE; COBB ANGLE; RELIABILITY; VALIDITY; REDUCE;

THORACOLUMBAR; DEFORMITY; SCHROTH

AB Objectives

The purpose of this study was to compare the efficacy of two different types of exercise methods in patients with adolescent idiopathic scoliosis.

Methods

In total, 28 subjects with adolescent idiopathic scoliosis with a mild curve magnitude (10 degrees-26 degrees) were randomly divided into two groups: the Schroth group (n = 14) and the core group (n = 14). The patients in the Schroth group were treated with supervised Schroth exercises, and the patients in the core group were treated with supervised core stabilization exercises; both groups performed the exercises for three days per week for a total of 10 weeks, and both were given additional traditional exercises to perform. Assessment included Cobb angle (Radiography), trunk rotation (Adam's test), cosmetic trunk deformity (Walter Reed Visual Assessment Scale), spinal mobility (Spinal Mouse), peripheral muscle strength (Biodex System 4-Pro), and quality of life (Scoliosis Research Society-22 questionnaire).

Results

It was found that patients in the Schroth group showed greater improvement in Cobb angles, thoracic trunk rotation angle, cosmetic trunk deformity, spinal mobility, and quality of life than those in the core group ($p<0.05$), except for in lumbar trunk rotation angle. Peripheral muscle strength improvement was greater in the core group than in the Schroth group ($p<0.05$).

Conclusion

Schroth exercises are more effective than core stabilization exercises in the correction of scoliosis and related problems in mild adolescent idiopathic scoliosis, and core stabilization exercises are more effective than Schroth exercises in the improvement of peripheral muscle strength.

C1 [Kocaman, Hikmet] Karamanoglu Mehmetbey Univ, Dept Physiotherapy & Rehabil, Prosthet Orthot Physiotherapy, Karaman, Turkey.

[Bek, Nilgun] Lokman Hekim Univ, Fac Hlth Sci, Dept Physiotherapy & Rehabil, Ankara, Turkey.

[Kaya, Mehmet Hanifi] Ahi Evran Univ, Fac Med, Kirsehir, Turkey.

[Buyukturan, Buket; Buyukturan, Oznur] Ahi Evran Univ, Sch Phys Therapy & Rehabil, Kirsehir, Turkey.

[Yetis, Mehmet] Ahi Evran Univ, Fac Med, Dept Orthoped & Traumatol, Kirsehir, Turkey.

C3 Karamanoglu Mehmetbey University; Lokman Hekim University; Ahi Evran

University; Ahi Evran University; Ahi Evran University

RP Kocaman, H (通讯作者)， Karamanoglu Mehmetbey Univ, Dept Physiotherapy & Rehabil, Prosthet Orthot Physiotherapy, Karaman, Turkey.

EM kcmnhikmet@gmail.com

RI Kocaman, Hikmet/AAH-9689-2020

OI Kocaman, Hikmet/0000-0001-5971-7274; KAYA, MEHMET

HANIFI/0000-0003-4670-4794

CR Akuthota V, 2004, ARCH PHYS MED REHAB, V85, pS86, DOI 10.1053/j.apmr.2003.12.005

Akuthota V, 2008, CURR SPORT MED REP, V7, P39, DOI 10.1097/01.CSMR.0000308663.13278.69

Alanay A, 2005, SPINE, V30, P2464, DOI 10.1097/01.brs.0000184366.71761.84

AMENDT LE, 1990, PHYS THER, V70, P108, DOI 10.1093/ptj/70.2.108

Anwer S, 2015, BIOMED RES INT-UK, V2015, DOI 10.1155/2015/123848

Berdishevsky H, 2016, SCOLIOSIS SPINAL DIS, V11, DOI 10.1186/s13013-016-0076-9

Bialek M, 2011, SCOLIOSIS SPINAL DIS, V6, DOI 10.1186/1748-7161-6-25

Laita LC, 2018, ARCH ARGENT PEDIATR, V116, pE582, DOI [10.5546/aap.2018.e582, 10.5546/aap.2018.eng.e582]

Colak TK, 2020, J TURKISH SPINAL SUR, V31, P125

DEACON P, 1984, J BONE JOINT SURG BR, V66, P509, DOI 10.1302/0301-620X.66B4.6746683

Demir E., 2020, AGE, V16, P1

Deviren V, 2002, SPINE, V27, P2346, DOI 10.1097/00007632-200211010-00007

Faul F, 2007, BEHAV RES METHODS, V39, P175, DOI 10.3758/BF03193146

Fishman LM, 2017, TOP GERIATR REHABIL, V33, P231, DOI 10.1097/TGR.0000000000000159

Gur G, 2017, PROSTHET ORTHOT INT, V41, P303, DOI 10.1177/0309364616664151

Hawes Martha C, 2003, Pediatr Rehabil, V6, P171

Horne JP, 2014, AM FAM PHYSICIAN, V89, P193

Jiang H, 2017, MED SCI MONITOR, V23, P2089, DOI 10.12659/MSM.902455

Kao Feng-Chun, 2014, Biomed J, V37, P78, DOI 10.4103/2319-4170.113182

Kim G, 2016, J PHYS THER SCI, V28, P1012, DOI 10.1589/jpts.28.1012

Ko KJ, 2017, J EXERC REHABIL, V13, P244, DOI 10.12965/jer.1734952.476

Kuru T, 2016, CLIN REHABIL, V30, P181, DOI 10.1177/0269215515575745

Kwan KYH, 2017, SCOLIOSIS SPINAL DIS, V12, DOI 10.1186/s13013-017-0139-6

Le Blay G, 2007, ISOKINET EXERC SCI, V15, P23

Lenhert-Schroth C., 1992, PHYSIOTHERAPY, V78, P810, DOI [10.1016/S0031-9406(10)60451-8, DOI 10.1016/S0031-9406(10)60451-8]

Livanelioglu A, 2016, EUR SPINE J, V25, P476, DOI 10.1007/s00586-015-3945-7

Ma HH, 2017, BIOMED ENG ONLINE, V16, DOI 10.1186/s12938-017-0427-7

Maher JM, 2013, CBE-LIFE SCI EDUC, V12, P345, DOI 10.1187/cbe.13-04-0082

Martinez-Llorens J, 2010, EUR RESPIR J, V36, P393, DOI 10.1183/09031936.00025509

Miller NH, 1999, ORTHOP CLIN N AM, V30, P343, DOI 10.1016/S0030-5898(05)70091-2

Monticone M, 2014, EUR SPINE J, V23, P1204, DOI 10.1007/s00586-014-3241-y

NACHEMSON AL, 1995, J BONE JOINT SURG AM, V77A, P815, DOI 10.2106/00004623-199506000-00001

Negrini S, 2008, DISABIL REHABIL, V30, P772, DOI 10.1080/09638280801889568

Negrini S, 2006, SCOLIOSIS SPINAL DIS, V1, DOI 10.1186/1748-7161-1-4

Negrini S, 2012, SCOLIOSIS SPINAL DIS, V7, DOI 10.1186/1748-7161-7-3

Otman Saadet, 2005, Neurosciences (Riyadh), V10, P277

Parent EC, 2010, SPINE, V35, P315, DOI 10.1097/BRS.0b013e3181cabe75

Park JH, 2018, EUR J PHYS REHAB MED, V54, P440, DOI 10.23736/S1973-9087.17.04461-6

Polmang B, 2019, WALAILAK J SCI TECHN, V16, P965

Post RB, 2004, ARCH ORTHOP TRAUM SU, V124, P187, DOI 10.1007/s00402-004-0641-1

RISSER J C, 1958, Clin Orthop, V11, P111

Romano M, 2013, SPINE, V38, pE883, DOI 10.1097/BRS.0b013e31829459f8

Rrecaj-Malaj S, 2020, MED SCI MONIT BASIC, V26, DOI 10.12659/MSMBR.920449

Sanders JO, 2003, SPINE, V28, P2158, DOI 10.1097/01.BRS.0000084629.97042.0B

Schreiber S, 2015, SCOLIOSIS SPINAL DIS, V10, DOI 10.1186/s13013-015-0048-5

Shin SS, 2012, J PHYS THER SCI, V24, P211

Slattery C, 2018, CLIN ORTHOP RELAT R, V476, P2271, DOI 10.1097/CORR.0000000000000405

Tsai Yi-Ta, 2010, Chang Gung Med J, V33, P540

Yagci G, 2019, PROSTHET ORTHOT INT, V43, P301, DOI 10.1177/0309364618820144

김정현, 2015, [Journal of Korea Academia-Industrial cooperation Society, 한국산학기술학회논문지], V16, P4098, DOI 10.5762/KAIS.2015.16.6.4098

NR 50

TC 10

Z9 10

U1 11

U2 45

PU PUBLIC LIBRARY SCIENCE

PI SAN FRANCISCO

PA 1160 BATTERY STREET, STE 100, SAN FRANCISCO, CA 94111 USA

SN 1932-6203

J9 PLOS ONE

JI PLoS One

PD APR 15

PY 2021

VL 16

IS 4

AR e0249492

DI 10.1371/journal.pone.0249492

PG 15

WC Multidisciplinary Sciences

WE Science Citation Index Expanded (SCI-EXPANDED); Social Science Citation Index (SSCI)

SC Science & Technology - Other Topics

GA RP1DC

UT WOS:000641474900034

PM 33857180

OA Green Published, gold

DA 2023-08-10

ER

PT J

AU Cantele, F

Maghini, I

Tonellato, M

Meneguzzo, P

Favaro, A

Masiero, S

AF Cantele, Francesca

Maghini, Irene

Tonellato, Michele

Meneguzzo, Paolo

Favaro, Angela

Masiero, Stefano

TI An Analysis of Eating Disorders in Adolescent Idiopathic Scoliosis: A

Prospective Cross-sectional Study in a Female Population

SO SPINE

LA English

DT Article

DE adolescent idiopathic scoliosis; eating disorders; anorexia; quality of

life; self-image; physiotherapy scoliosis-specific exercises; orthotic

management; scoliosis brace; SRS-22r; adolescent health care

ID QUALITY-OF-LIFE; SCOLIOSIS-RESEARCH-SOCIETY-22 PATIENT QUESTIONNAIRE;

RELIABILITY; INSTRUMENT; VALIDITY; OUTCOMES; RISK

AB Study Design. Prospective cross-sectional study. Objective. We designed this study to investigate whether adolescent girls with idiopathic scoliosis show a predisposition for eating disorders (EDs) and alterations of the quality of life and body image self-perception, compared to same-age healthy females. Summary of Background Data. Idiopathic scoliosis is the most common spinal deformity of adolescence. Recent findings about the impairment of the self-body image in adolescents with idiopathic scoliosis provide a common trait of scoliosis and EDs and could lead to the suspicion of an association between these two pathological conditions. Despite this, current literature shows the lack of evident results about the impact of adolescent idiopathic scoliosis (AIS) on the possibility to develop of EDs. Methods. One hundred forty-four females with diagnosis of AIS (aged 10-18 years) formed the scoliosis group. One hundred forty-six same-age healthy girls were enrolled in the control group. For all subjects, we considered sport practice. Only for Scoliosis Group, we also considered

the severity of scoliosis, the use of brace and the practice of physiotherapy scoliosis-specific exercises. The participants were asked to answer to the Scoliosis Research Society-22 revised Patient Questionnaire (SRS-22r) and the Eating Disorders Inventory (EDI). Results. In the scoliosis group, significantly lower scores on the SRS-22r total and in the self-image domain were detected. The two groups showed differences in the total EDI score and in the body dissatisfaction EDI's domain. Severity of scoliosis was correlated with worse SRS-22r total score and SRS-22r self-image domain score. There were no differences in the scores of the SRS-22r and EDI between braced and nonbraced subjects. Lower scores in SRS-22r total and self-image domain were found in girls who practiced physiotherapy exercises. Subjects who practiced a sport showed higher SRS-22r total scores. Conclusion. The AIS cohort in our study demonstrated lower levels of eating psychopathology than healthy controls. Surprisingly, eating behavior does not seem to be affected by orthotic management. However, quality of life and self-body image could be impaired in scoliotic girls, especially when they practice physiotherapy exercises, whereas those who practice sport seem to be preserved in this regard.

C1 [Cantele, Francesca; Tonellato, Michele; Masiero, Stefano] Univ Padua, Phys Med & Rehabil Sch, Via Giustiniani 2, I-35128 Padua, Italy.

[Maghini, Irene] Univ Padua, Womans & Childs Dept, Pediat Pain & Palliat Care Serv, Padua, Italy.

[Meneguzzo, Paolo; Favaro, Angela] Univ Padua, Dept Neurosci, Padua, Italy.

[Favaro, Angela] Univ Padua, Padua Neurosci Ctr, Padua, Italy.

[Masiero, Stefano] Univ Padua, Sect Rehabil, Dept Neurosci, Via Giustiniani 2, Padua, Italy.

C3 University of Padua; University of Padua; University of Padua;

University of Padua; University of Padua

RP Cantele, F (通讯作者)， Univ Padua, Phys Med & Rehabil Sch, Via Giustiniani 2, I-35128 Padua, Italy.

EM francesca.cantele.11@gmail.com

RI Meneguzzo, Paolo/J-8425-2018; Favaro, Angela/J-2966-2012

OI Meneguzzo, Paolo/0000-0003-3323-6071; Masiero,

Stefano/0000-0002-0361-4898; Favaro, Angela/0000-0002-6540-5194

CR Alborghetti Amelia, 2008, Eat Disord, V16, P85, DOI 10.1080/10640260701773660

Asher M, 2003, SPINE, V28, P74, DOI 10.1097/00007632-200301010-00017

Asher M, 2003, SPINE, V28, P63, DOI 10.1097/00007632-200301010-00015

Bauer A, 2017, J ABNORM CHILD PSYCH, V45, P1647, DOI 10.1007/s10802-017-0263-z

Cheung KMC, 2007, INT ORTHOP, V31, P507, DOI 10.1007/s00264-006-0209-5

Ferraro C., 1998, EUR MEDICOPHYS, V34, P25

Ferraro C, 2017, EUR J PHYS REHAB MED, V53, P377, DOI 10.23736/S1973-9087.16.04227-1

Freidel K, 2002, SPINE, V27, pE87, DOI 10.1097/00007632-200202150-00013

Gallant JN, 2018, WORLD NEUROSURG, V116, P421, DOI 10.1016/j.wneu.2018.05.104

GARNER DM, 1983, INT J EAT DISORDER, V2, P15, DOI 10.1002/1098-108X(198321)2:2<15::AID-EAT2260020203>3.0.CO;2-6

Garner DM., 1997, HDB PSYCHOTHERAPY AN, V2

최지혜, 2011, Child Health Nursing Research, V17, P167

Giannini M., 2008, NUOVA VERSIONE EATIN

Janicki JA, 2007, PAED CHILD HEALT-CAN, V12, P771, DOI 10.1093/pch/12.9.771

Lee H, 2016, SCOLIOSIS SPINAL DIS, V11, DOI 10.1186/s13013-016-0071-1

Meland E, 2007, HEALTH EDUC RES, V22, P342, DOI 10.1093/her/cyl085

Meng ZD, 2017, MEDICINE, V96, DOI 10.1097/MD.0000000000006828

Monteleone AM, 2019, INT J EAT DISORDER, V52, P1263, DOI 10.1002/eat.23137

Monticone M, 2010, SPINE, V35, pE1412, DOI 10.1097/BRS.0b013e3181e88981

Negrini S, 2005, Eura Medicophys, V41, P183

Negrini S, 2018, SCOLIOSIS SPINAL DIS, V13, DOI 10.1186/s13013-017-0145-8

Rohde P, 2015, INT J EAT DISORDER, V48, P187, DOI 10.1002/eat.22270

Rushton PRP, 2013, SPINE, V38, P778, DOI 10.1097/BRS.0b013e31827db418

Sapountzi-Krepia DS, 2001, J ADV NURS, V35, P683, DOI 10.1046/j.1365-2648.2001.01900.x

Smith FM, 2008, J ADOLESCENT HEALTH, V42, P58, DOI 10.1016/j.jadohealth.2007.08.008

Solmi M, 2018, INT J EAT DISORDER, V51, P680, DOI 10.1002/eat.22884

Tam EMS, 2016, SPINE, V41, P940, DOI 10.1097/BRS.0000000000001376

te Velde SJ, 2018, SPORTS MED-OPEN, V4, DOI 10.1186/s40798-018-0152-1

Tones M, 2006, SPINE, V31, P3027, DOI 10.1097/01.brs.0000249555.87601.fc

Ugwonali Obinwanne F, 2004, Spine J, V4, P254

Verma K, 2014, J PEDIATR ORTHOPED, V34, P503, DOI 10.1097/BPO.0000000000000137

Watanabe K, 2005, SPINE, V30, P1202, DOI 10.1097/01.brs.0000162285.12045.81

Zaina F, 2013, RES DEV DISABIL, V34, P1119, DOI 10.1016/j.ridd.2013.01.001

NR 33

TC 4

Z9 5

U1 2

U2 12

PU LIPPINCOTT WILLIAMS & WILKINS

PI PHILADELPHIA

PA TWO COMMERCE SQ, 2001 MARKET ST, PHILADELPHIA, PA 19103 USA

SN 0362-2436

EI 1528-1159

J9 SPINE

JI SPINE

PD APR 1

PY 2021

VL 46

IS 7

BP 440

EP 446

DI 10.1097/BRS.0000000000003885

PG 7

WC Clinical Neurology; Orthopedics

WE Science Citation Index Expanded (SCI-EXPANDED)

SC Neurosciences & Neurology; Orthopedics

GA SW6KI

UT WOS:000664622400007

PM 33337684

DA 2023-08-10

ER

PT J

AU Romano, M

Negrini, A

Negrini, S

AF Romano, Michele

Negrini, Alessandra

Negrini, Stefano

TI Lessons learned in two months of exclusive application of telephysiotherapy instead of classical physiotherapy during the lockdown in Italy

SO SPINE JOURNAL

LA English

DT Article

DE Telephysiotherapy; Idiopathic scoliosis; Telemedicine; Rehabilitation; Exercise therapy

C1 [Romano, Michele; Negrini, Alessandra] ISICO Italian Sci Spine Inst, Milan, Italy.

[Negrini, Stefano] Univ La Statale, Dept Biomed Surg & Dent Sci, Milan, Italy.

[Negrini, Stefano] IRCCS Ist Ortoped Galeazzi, Milan, Italy.

C3 IRCCS Istituto Ortopedico Galeazzi

RP Negrini, S (通讯作者)，IRCCS Ist Ortoped Galeazzi, Milan, Italy.

EM stefano.negrini@unimi.it

RI Negrini, Stefano/B-6667-2013

OI Negrini, Stefano/0000-0002-1878-2747; Negrini,

Alessandra/0000-0002-9195-9019

CR [Anonymous], 2020, ARCH PHYS MED REHAB, DOI [10.1016/j.apmr.2020.08.001, DOI 10.1016/J.APMR.2020.08.001]

[Anonymous], 2012, SCOLIOSIS SPINAL DIS, V7, P17, DOI [10.1186/1748-7161-7-17, DOI 10.1186/1748-7161-7-17]

Bedford J, 2020, LANCET, V395, P1015, DOI 10.1016/S0140-6736(20)30673-5

Boldrini P, 2020, EUR J PHYS REHAB MED, V56, P316, DOI 10.23736/S1973-9087.20.06256-5

Dolan LA, AIS BRACING SUCCESS, P2020, DOI [10.1097/BRS.0000000000003506., DOI 10.1097/BRS.0000000000003506]

Donzelli S, 2012, SCOLIOSIS SPINAL DIS, V7, DOI 10.1186/1748-7161-7-12

Flodgren G, 2015, COCHRANE DB SYST REV, DOI 10.1002/14651858.CD002098.pub2

Grasselli G, 2020, JAMA-J AM MED ASSOC, V323, P1545, DOI 10.1001/jama.2020.4031

Nacoti M, 2020, NEJM CATALYST, V1, P1, DOI [DOI 10.1056/CAT.20.0080, 10.1056/CAT.20.0080]

Negrini A, 2019, PHYS THER, V99, P1712, DOI 10.1093/ptj/pzz123

Negrini S, FEASIBILITY ACCEPTAB

Negrini S, 2020, EUR J PHYS REHAB MED, V56, P361, DOI 10.23736/S1973-9087.20.06361-3

Negrini S, 2019, ANN PHYS REHABIL MED, V62, P69, DOI 10.1016/j.rehab.2018.07.010

Negrini S, 2018, SCOLIOSIS SPINAL DIS, V13, DOI 10.1186/s13013-017-0145-8

Negrini S, 2015, COCHRANE DB SYST REV, DOI 10.1002/14651858.CD006850.pub3

Negrini S, 2009, SCOLIOSIS SPINAL DIS, V4, DOI 10.1186/1748-7161-4-2

Roye BD, 2020, SPINE DEFORM, V8, P597, DOI 10.1007/s43390-020-00060-1

Tavernaro M, TEAM CARE CURE ADOLE

van Egmond MA, 2018, PHYSIOTHERAPY, V104, P277, DOI 10.1016/j.physio.2018.04.004

NR 19

TC 1

Z9 1

U1 0

U2 8

PU ELSEVIER SCIENCE INC

PI NEW YORK

PA STE 800, 230 PARK AVE, NEW YORK, NY 10169 USA

SN 1529-9430

EI 1878-1632

J9 SPINE J

JI Spine Journal

PD MAR

PY 2021

VL 21

IS 3

BP 366

EP 369

DI 10.1016/j.spinee.2020.10.023

EA FEB 2021

PG 4

WC Clinical Neurology; Orthopedics

WE Science Citation Index Expanded (SCI-EXPANDED)

SC Neurosciences & Neurology; Orthopedics

GA QI0PB

UT WOS:000618676400002

PM 33589094

OA Green Submitted

DA 2023-08-10

ER

PT J

AU Strube, P

Gunold, M

Muller, T

Leimert, M

Sachse, A

Pumberger, M

Putzier, M

Zippelius, T

AF Strube, P.

Gunold, M.

Mueller, T.

Leimert, M.

Sachse, A.

Pumberger, M.

Putzier, M.

Zippelius, T.

TI Influence of curve morphology and location on the efficacy of rigid

conservative treatment in patients with adolescent idiopathic scoliosis

SO BONE & JOINT JOURNAL

LA English

DT Article

ID BRACE TREATMENT; NATURAL-HISTORY; PROGRESSION; SUCCESS; MATURITY;

VALIDATION; SCHROTH; SOSORT; SYSTEM

AB Aims

The aim of the present study was to answer the question whether curve morphology and location have an influence on rigid conservative treatment in patients with adolescent idiopathic scoliosis (AIS).

Methods

We retrospectively analyzed AIS in 127 patients with single and double curves who had been treated with a Cheneau brace and physiotherapeutic specific exercises (B-PSE). The inclusion criteria were the presence of structural major curves >= 20 degrees and < 50 degrees (Risser stage 0 to 2) at the time when B-PSE was initiated. The patients were divided into two groups according to the outcome of treatment: failure (curve progression to >= 45 degrees or surgery) and success (curve progression < 45 degrees and no surgery). The main curve type (MCT), curve magnitude, and length (overall, above and below the apex), apical rotation, initial curve correction, flexibility, and derotation by the brace were compared between the two groups.

Results

In univariate analysis treatment failure depended significantly on: 1) MCT (p = 0.008); 2) the apical rotation of the major curve before (p = 0.007) and during brace treatment (p < 0.001); 3) the initial and in-brace Cobb angles of the major (p = 0.001 and p < 0.001, respectively) and minor curves (p = 0.015 and p = 0.002); 4) major curve flexibility (p = 0.005) and the in-brace curve correction rates (major p = 0.008, minor p = 0.034); and 5) the length of the major curve (LoC) above (p < 0.001) and below (p = 0.002) the apex. Furthermore, MCT (p = 0.043, p = 0.129, and p = 0.017 in MCT comparisons), LoC (upper length p = 0.003, lower length p = 0.005), and in-brace Cobb angles (major p = 0.002, minor p = 0.027) were significant in binary logistic regression analysis.

Conclusion

Curve size, location, and morphology were found to influence the outcome of rigid conservative treatment of AIS. These findings may improve future brace design and patient selection for conservative treatment.

C1 [Leimert, M.; Pumberger, M.; Putzier, M.] Jena Univ Hosp, Jena, Germany.

[Strube, P.] Jena Univ Hosp, Waldkliniken Eisenberg, Dept Orthopaed, Orthopaed Spine Dept, Jena, Germany.

[Gunold, M.; Mueller, T.; Zippelius, T.] Jena Univ Hosp, Waldkliniken Eisenberg, Dept Orthopaed, Jena, Germany.

[Sachse, A.] Jena Univ Hosp, Waldkliniken Eisenberg, Dept Orthopaed, Pediat Orthopaed Dept, Jena, Germany.

[Leimert, M.] Interdisciplinary Spine Ctr Neurosurg Spine Surg, Neurosurg Dept, Asklepios Sachs Schweiz Klin Sebnitz, Sebnitz, Germany.

[Pumberger, M.; Putzier, M.] Charite Univ Med Berlin, Ctr Musculoskeletal Surg, Dept Orthopaed, Charite Spine Ctr, Berlin, Germany.

C3 Friedrich Schiller University of Jena; Friedrich Schiller University of

Jena; Friedrich Schiller University of Jena; Friedrich Schiller

University of Jena; Free University of Berlin; Humboldt University of

Berlin; Charite Universitatsmedizin Berlin

RP Strube, P (通讯作者)， Jena Univ Hosp, Waldkliniken Eisenberg, Dept Orthopaed, Orthopaed Spine Dept, Jena, Germany.

EM patrick.strube@uni-jena.de

RI Strube, Patrick/AAH-5408-2019

OI Strube, Patrick/0000-0003-3210-5301; Pumberger,

Matthias/0000-0002-0885-7370

CR Abel MF, 2017, J BONE JOINT SURG AM, V99, DOI 10.2106/JBJS.17.00043

Aulisa AG, 2014, SCOLIOSIS SPINAL DIS, V9, DOI 10.1186/1748-7161-9-6

Benish BM, 2012, SPINE, V37, P309, DOI 10.1097/BRS.0b013e31821e1488

Cheung JPY, 2020, BONE JOINT J, V102B, P254, DOI 10.1302/0301-620X.102B2.BJJ-2019-0916.R1

Cheung JPY, 2019, BONE JOINT J, V101B, P1370, DOI 10.1302/0301-620X.101B11.BJJ-2019-0515.R1

Cobb JR., 1948, INSTR COURSE LECT, V5, P261

EMANS JB, 1986, SPINE, V11, P792, DOI 10.1097/00007632-198610000-00009

Karol LA, 2016, J BONE JOINT SURG AM, V98, P9, DOI 10.2106/JBJS.O.00359

Katz DE, 2001, SPINE, V26, P2354, DOI 10.1097/00007632-200111010-00012

Kwan KYH, 2017, SCOLIOSIS SPINAL DIS, V12, DOI 10.1186/s13013-017-0139-6

Lam GC, 2008, SCOLIOSIS SPINAL DIS, V3, DOI 10.1186/1748-7161-3-16

Landauer Franz, 2003, Pediatr Rehabil, V6, P201

Lenke LG, 2001, J BONE JOINT SURG AM, V83A, P1169, DOI 10.2106/00004623-200108000-00006

Li ZK, 2017, MEDICINE, V96, DOI 10.1097/MD.0000000000009425

LONSTEIN JE, 1988, ORTHOP CLIN N AM, V19, P227

Lusini M, 2014, SPINE J, V14, P1951, DOI 10.1016/j.spinee.2013.11.040

NACHEMSON AL, 1995, J BONE JOINT SURG AM, V77A, P815, DOI 10.2106/00004623-199506000-00001

NASH CL, 1969, J BONE JOINT SURG AM, VA 51, P223, DOI 10.2106/00004623-196951020-00002

Negrini S, 2014, EUR J PHYS REHAB MED, V50, P87

Negrini S, 2018, SCOLIOSIS SPINAL DIS, V13, DOI 10.1186/s13013-017-0145-8

Negrini S, 2015, SCOLIOSIS SPINAL DIS, V10, DOI 10.1186/s13013-014-0025-4

Nicholson GP, 2003, SPINE, V28, P2243, DOI 10.1097/01.BRS.0000085098.69522.52

Ovadia D, 2012, J CHILD ORTHOP, V6, P327, DOI 10.1007/s11832-012-0429-8

Rigo M, 2017, SCOLIOSIS SPINAL DIS, V12, P1, DOI 10.1186/s13013-017-0114-2

RISSER J C, 1958, Clin Orthop, V11, P111

Sanders A.E., 2018, SPINE DEFORM, V6, P435, DOI [10.1016/j.jspd.2017.12.014, DOI 10.1016/J.JSPD.2017.12.014]

Sanders JO, 2007, J BONE JOINT SURG AM, V89A, P64, DOI 10.2106/JBJS.F.00067

Seifert J, 2016, ORTHOPADE, V45, P509, DOI 10.1007/s00132-016-3274-5

Sitoula P, 2015, SPINE, V40, P1006, DOI 10.1097/BRS.0000000000000952

Sponseller PD, 2011, J PEDIATR ORTHOPED, V31, pS53, DOI 10.1097/BPO.0b013e3181f73e87

Thompson RM, 2017, J BONE JOINT SURG AM, V99, P923, DOI 10.2106/JBJS.16.01050

Weinstein SL, 2013, NEW ENGL J MED, V369, P1512, DOI 10.1056/NEJMoa1307337

Weiss HR, 2011, SCOLIOSIS SPINAL DIS, V6, DOI 10.1186/1748-7161-6-17

Wong HK, 2010, INDIAN J ORTHOP, V44, P9, DOI 10.4103/0019-5413.58601

Yamane K, 2016, ASIAN SPINE J, V10, P893, DOI 10.4184/asj.2016.10.5.893

NR 35

TC 4

Z9 4

U1 0

U2 8

PU BRITISH EDITORIAL SOC BONE & JOINT SURGERY

PI LONDON

PA 22 BUCKINGHAM STREET, LONDON WC2N 6ET, united kingdom

SN 2049-4394

J9 BONE JOINT J

JI Bone Joint J.

PD FEB

PY 2021

VL 103B

IS 2

BP 373

EP 381

DI 10.1302/0301-620X.103B2.BJJ-2020-1113.R2

PG 9

WC Orthopedics; Surgery

WE Science Citation Index Expanded (SCI-EXPANDED)

SC Orthopedics; Surgery

GA RM2BG

UT WOS:000639463200024

PM 33517722

DA 2023-08-10

ER

PT J

AU Fan, YL

To, MKT

Yeung, EHK

Wu, JB

He, R

Xu, ZM

Zhang, RW

Li, GS

Cheung, KMC

Cheung, JPY

AF Fan, Yunli

To, Michael K. T.

Yeung, Eric H. K.

Wu, Jianbin

He, Rong

Xu, Zhuoman

Zhang, Ruiwen

Li, Guangshuo

Cheung, Kenneth M. C.

Cheung, Jason P. Y.

TI Does curve pattern impact on the effects of physiotherapeutic scoliosis

specific exercises on Cobb angles of participants with adolescent

idiopathic scoliosis: A prospective clinical trial with two years

follow-up

SO PLOS ONE

LA English

DT Article

ID MODEL; BIOMECHANICS; BRACE

AB Background

Current clinical evidence suggests that a well-planned physiotherapeutic scoliosis specific exercise (PSSE) program is effective for scoliosis regression.

Objectives

We investigated the effect of curve patterns on Cobb angles with PSSE.

Methods

This was a non-randomized prospective clinical trial that recruited participants with adolescent idiopathic scoliosis between January and June 2017. Participants were grouped by curve pattern into major thoracic and major lumbar groups. An outpatient-based PSSE program was conducted with the following schedule of intensive exercise: >= 1 session of supervised PSSE per month and > 30min of home exercise 5 days/week in the first 6 months, after which exercise frequency was reduced to 1 session of supervised PSSE every three months and > 30min of home exercise 5 days/week until 2 years after study initiation. Radiographic Cobb angle progressions were identified at the 1, 1.5 and 2-year follow-ups. A mixed model analysis of variance (ANOVA) was performed to examine the differences in Cobb angles between groups at four testing time points. The two-tailed significance level was set to 0.05.

Results

In total, 40 participants were recruited, including 22 with major thoracic curves (5 males and 17 females; mean age 13.5 +/- 1.8 years; Cobb angle 18-45 degrees) and 18 with major lumbar curves (7 males and 11 females; mean age 12.7 +/- 1.7 years; Cobb angle 15-48 degrees). Curve regressions, namely the reduction of Cobb angles between 7 to 10 degrees were noted in 9.1% of participants in the major thoracic group; reductions of 6 to 13 degrees were noted in 33.3% of participants in the major lumbar group at the 2-year follow-up. Repeated measurements revealed a significant time effect (F-2.2,F-79.8 = 4.1, p = 0.02), but no group (F-2.2,F-79.8 = 2.3, p = 0.1) or time x group (F-

1,F-37 = 0.97, p = 0.3) effects in reducing Cobb angles after 2 years of PSSE. A logistic regression analysis revealed that no correlation was observed between curve pattern and curve regression or stabilization (OR: 0.2, 95% CI: 0.31-1.1, p = 0.068) at the 2-year follow-up.

Conclusion

This was the first study to investigate the long-term effects of PSSE in reducing Cobb angles on the basis of major curve location. No significant differences in correction were observed between major thoracic and major lumbar curves. A regression effect and no curve deterioration were noted in both groups at the 2-year follow-up.

C1 [Fan, Yunli; To, Michael K. T.; Wu, Jianbin; Cheung, Kenneth M. C.; Cheung, Jason P. Y.] Univ Hong Kong, Shenzhen Hosp, Dept Orthopaed, Shenzhen, Guangdong, china.

[Fan, Yunli; To, Michael K. T.; Cheung, Kenneth M. C.; Cheung, Jason P. Y.] Univ Hong Kong, Dept Orthpaed & Traumatol, Hong Kong, china.

[Fan, Yunli; Yeung, Eric H. K.; He, Rong; Xu, Zhuoman; Zhang, Ruiwen; Li, Guangshuo] Univ Hong Kong, Dept Physiotherapy, Shenzhen Hosp, Shenzhen, Guangdong, china.

C3 University of Hong Kong; University of Hong Kong; University of Hong

Kong

RP Cheung, JPY (通讯作者)， Univ Hong Kong, Shenzhen Hosp, Dept Orthopaed, Shenzhen, Guangdong, china.; Cheung, JPY (通讯作者)， Univ Hong Kong, Dept Orthpaed & Traumatol, Hong Kong, china.

EM cheungjp@hku.hk

RI Cheung, Jason Pui Yin/J-2214-2012; Cheung, Jason Pui Yin/AAJ-2016-2020

OI Cheung, Jason Pui Yin/0000-0002-7052-0875; Fan,

Yunli/0000-0001-9936-8857; Zhang, Ruiwen/0000-0001-5482-8899

FU Sanming Project of Medicine "Team of Excellence in Spinal Deformities

and Spinal Degeneration Diseases" in Shenzhen, Guangdong province, China

[SZSM201612055]; University of Hong Kong - Shenzhen Hospital Seeding

Project [HKUSZH201902042]

FX Funding Project: Sanming Project of Medicine (SZSM201612055) "Team of

Excellence in Spinal Deformities and Spinal Degeneration Diseases" in

Shenzhen, Guangdong province, China. The University of Hong Kong -

Shenzhen Hospital Seeding Project, HKUSZH201902042, Jason PY Cheung

Receiver: Kenneth MC Cheung, Jason PY Cheung.

CR Almansour H, 2019, J CLIN MED, V8, DOI 10.3390/jcm8111804

[Anonymous], 1984, NUREG, V3, P1061

[Anonymous], 2012, APPL LONGITUDINAL AN

[Anonymous], 2016, WHO DRUG INFO, V30, P181

BARNES PD, 1993, RADIOLOGY, V186, P247, DOI 10.1148/radiology.186.1.8416573

Berdishevsky H, 2016, SCOLIOSIS SPINAL DIS, V11, DOI 10.1186/s13013-016-0076-9

Burger M, 2019, SOUTH AFR J PHYSIOTH, V75, DOI 10.4102/sajp.v75i1.904

Chan SL, 2014, SCOLIOSIS SPINAL DIS, V9, DOI 10.1186/1748-7161-9-1

COBB JR, 1958, J BONE JOINT SURG AM, V40, P507, DOI 10.2106/00004623-195840030-00002

Coe JD, 2006, SPINE, V31, P345, DOI 10.1097/01.brs.0000197188.76369.13

Czaprowski D, 2011, SCOLIOSIS SPINAL DIS, V6, DOI 10.1186/1748-7161-6-22

Davids JR, 2004, J BONE JOINT SURG AM, V86A, P2187, DOI 10.2106/00004623-200410000-00009

De Giorgi S, 2013, EUR SPINE J, V22, pS815, DOI 10.1007/s00586-013-3020-1

Dolan LA, 2019, SPINE DEFORM, V7, P890, DOI 10.1016/j.jspd.2019.01.011

Dolgin E, 2019, CANCER DISCOV, V9, P8, DOI 10.1158/2159-8290.CD-NB2018-163

Eskandari AH, 2017, J BIOMECH, V57, P18, DOI 10.1016/j.jbiomech.2017.03.011

Fan YL, 2020, BMC MUSCULOSKEL DIS, V21, DOI 10.1186/s12891-020-03517-6

Gstoettner M, 2007, EUR SPINE J, V16, P1587, DOI 10.1007/s00586-007-0401-3

Hajihosseinali M, 2014, MED ENG PHYS, V36, P1296, DOI 10.1016/j.medengphy.2014.07.009

Kim S, 2017, STAT METHODS MED RES, V26, P1237, DOI 10.1177/0962280215572407

Korbel Krzysztof, 2014, Pol Orthop Traumatol, V79, P118

Lehnert-Schroth C, 1981, ZFA (Stuttgart), V57, P2227

Lehnert-Schroth C, 1979, ZFA (Stuttgart), V55, P1969

Li M, 2014, SPINE, V39, P1408, DOI 10.1097/BRS.0000000000000423

LITTLE DG, 1994, J PEDIATR ORTHOPED, V14, P569, DOI 10.1097/01241398-199409000-00003

Liu DL, 2020, SPINE, V45, P1039, DOI 10.1097/BRS.0000000000003451

Malfair D, 2010, AM J ROENTGENOL, V194, pS8, DOI 10.2214/AJR.07.7145

Mishra P, 2019, ANN CARD ANAESTH, V22, P67, DOI 10.4103/aca.ACA_157_18

Monticone M, 2014, EUR SPINE J, V23, P1204, DOI 10.1007/s00586-014-3241-y

Negrini S, 2006, SCOLIOSIS SPINAL DIS, V1, DOI 10.1186/1748-7161-1-14

Negrini S, 2009, SCOLIOSIS SPINAL DIS, V4, DOI 10.1186/1748-7161-4-19

Oxland TR, 2016, J BIOMECH, V49, P817, DOI 10.1016/j.jbiomech.2015.10.035

PANJABI MM, 1980, NEUROSURGERY, V7, P76, DOI 10.1227/00006123-198007000-00014

Rigo MD, 2010, SCOLIOSIS SPINAL DIS, V5, DOI 10.1186/1748-7161-5-1

Schreiber S, 2016, PLOS ONE, V11, DOI 10.1371/journal.pone.0168746

Smits-Engelsman B, 2011, J PEDIATR-US, V158, P130, DOI 10.1016/j.jpeds.2010.07.021

Thompson JY, 2019, PHYSIOTHERAPY, V105, P214, DOI 10.1016/j.physio.2018.10.004

Thompson RM, 2017, J BONE JOINT SURG AM, V99, P923, DOI 10.2106/JBJS.16.01050

van Ginkel JR, 2014, MULTIVAR BEHAV RES, V49, P78, DOI 10.1080/00273171.2013.855890

Wang WJ, 2012, SPINE, V37, P1586, DOI 10.1097/BRS.0b013e3182511d0c

Weinstein SL, 2008, LANCET, V371, P1527, DOI 10.1016/S0140-6736(08)60658-3

Weinstein SL, 2019, J PEDIATR ORTHOPED, V39, pS44, DOI 10.1097/BPO.0000000000001350

Weiss H R, 1997, Pediatr Rehabil, V1, P35

Weiss Hans-Rudolf, 2002, Stud Health Technol Inform, V91, P352

Weiss Hans-Rudolf, 2003, Pediatr Rehabil, V6, P23, DOI 10.1080/1363849031000095288

Yagci G, 2019, PROSTHET ORTHOT INT, V43, P301, DOI 10.1177/0309364618820144

Yagci G, 2018, J BACK MUSCULOSKELET, V31, P693, DOI 10.3233/BMR-170868

NR 47

TC 2

Z9 2

U1 1

U2 18

PU PUBLIC LIBRARY SCIENCE

PI SAN FRANCISCO

PA 1160 BATTERY STREET, STE 100, SAN FRANCISCO, CA 94111 USA

SN 1932-6203

J9 PLOS ONE

JI PLoS One

PD JAN 25

PY 2021

VL 16

IS 1

AR e0245829

DI 10.1371/journal.pone.0245829

PG 17

WC Multidisciplinary Sciences

WE Science Citation Index Expanded (SCI-EXPANDED)

SC Science & Technology - Other Topics

GA PZ9QF

UT WOS:000613081500068

PM 33493172

OA Green Published, gold

DA 2023-08-10

ER

PT J

AU Zagalaz-Anula, N

Leon-Morillas, F

Andradre-Ortega, JA

Ibanez-Vera, AJ

de Oliveira-Sousa, SL

Lomas-Vega, R

AF Zagalaz-Anula, Noelia

Leon-Morillas, Felipe

Andradre-Ortega, Juan Alfonso

Ibanez-Vera, Alfonso Javier

de Oliveira-Sousa, Silvana Loana

Lomas-Vega, Rafael

TI Case Report: Conservative Treatment of Adolescent Idiopathic Scoliosis

Can Alter the Perception of Verticality. A Preliminary Study

SO FRONTIERS IN PEDIATRICS

LA English

DT Article

DE idiopathic scoliosis; postural control; adolescent idiopathic scoliosis;

scoliosis physical therapy; sense of verticality; spinal diseases

AB Adolescent idiopathic scoliosis (AIS) is a lateral curvature of the spine of at least 10 degrees Cobb's angle of unknown etiology. Some studies have found that patients with AIS have a Visual Verticality (VV) perception similar to healthy controls. This study aimed to analyze VV perception and postural balance differences in patients with AIS depending on the management, either based on observation or conservative treatment. Eighteen patients with AIS were included in this study. Nine

patients were managed based on observation. The other nine underwent conservative treatment, such as bracing or exercise. Subjective Visual Vertical (SVV) and posturographic parameters were measured and analyzed. In the SVV test, patients who underwent treatment showed poor constant error in absolute values and mean absolute error, with statistically significant differences ($p < 0.05$). Only the Romberg Quotient for sway area was within the limits of statistical significance for posturographic parameters, with a lower value for patients under observation. This study found worse perception of verticality in patients receiving some type of conservative treatment than patients receiving only observation; whereas posturography showed similar values in both observation and treatment groups. Our results can be interpreted as the effect of treatment on the previous verticality perception adapted to the curvature.

C1 [Zagalaz-Anula, Noelia; Ibanez-Vera, Alfonso Javier; Lomas-Vega, Rafael] Univ Jaen, Dept Hlth Sci, Jaen, Spain.

[Leon-Morillas, Felipe] Catholic Univ Murcia UCAM, Hlth Sci PhD Program, Guadalupe, Spain.

[Andradre-Ortega, Juan Alfonso] Hosp Complex Jaen, Dept Phys Med & Rehabil, Jaen, Spain.

[de Oliveira-Sousa, Silvana Loana] Univ Murcia, Dept Physiotherapy, Murcia, Spain.

C3 Universidad de Jaen; Universidad Catolica de Murcia; University of

Murcia

RP Ibanez-Vera, AJ (通讯作者)， Univ Jaen, Dept Hlth Sci, Jaen, Spain.

EM ajibanez@ujaen.es

RI Zagalaz-Anula, Noelia/AAE-9027-2021; León-Morillas,

Felipe/AAD-5171-2022; Ibáñez-Vera, AJ/V-2681-2019; Oliveira-Sousa,

Silvana/AFQ-8593-2022

OI Zagalaz-Anula, Noelia/0000-0002-7105-9865; Ibáñez-Vera,

AJ/0000-0001-9103-9696; LEON-MORILLAS, FELIPE/0000-0001-9426-379X;

Oliveira-Sousa, Silvana/0000-0003-1842-2968

CR Catanzariti J. -F., 2014, Annals of Physical and Rehabilitation Medicine, V57, P465, DOI 10.1016/j.rehab.2014.04.003

COHEN J, 1992, PSYCHOL BULL, V112, P155, DOI 10.1037/0033-2909.112.1.155

Day Joseph M, 2019, Arch Physiother, V9, P8, DOI 10.1186/s40945-019-0060-9

Dufvenberg M, 2018, SCOLIOSIS SPINAL DIS, V13, DOI 10.1186/s13013-018-0163-1

Dunn J., 2018, SCREENING ADOLESCENT

Dupuis S, 2018, BMC MUSCULOSKEL DIS, V19, DOI 10.1186/s12891-018-2112-9

Fortin C, 2012, BMC MUSCULOSKEL DIS, V13, DOI 10.1186/1471-2474-13-80

Grossman DC, 2018, JAMA-J AM MED ASSOC, V319, P165, DOI 10.1001/jama.2017.19342

Gur G, 2015, GAIT POSTURE, V41, P93, DOI 10.1016/j.gaitpost.2014.09.001

Hita-Contreras F, 2013, CLIMACTERIC, V16, P584, DOI 10.3109/13697137.2012.733464

Jagger Fiona, 2020, J Clin Orthop Trauma, V11, P191, DOI 10.1016/j.jcot.2019.12.013

Kaelin AJ, 2020, ANN TRANSL MED, V8, DOI 10.21037/atm.2019.09.69

Kuznia AL, 2020, AM FAM PHYSICIAN, V101, P19

Le Berre M, 2019, SPINE DEFORM, V7, P71, DOI 10.1016/j.jspd.2018.05.004

Lomas-Vega R, 2017, AM J PHYS MED REHAB, V96, P124, DOI 10.1097/PHM.0000000000000575

Min KK, 2007, OTOL NEUROTOL, V28, P520, DOI 10.1097/01.mao.0000271674.41307.f2

Molina F, 2019, NEUROREHABILITATION, V44, P379, DOI 10.3233/NRE-182642

Negrillo-Cardenas J, 2018, J MED SYST, V42, DOI 10.1007/s10916-018-0981-y

Piatek E, 2019, PEERJ, V7, DOI 10.7717/peerj.7513

Piscicelli C, 2017, ANN PHYS REHABIL MED, V60, P208, DOI 10.1016/j.rehab.2016.02.004

Weniger CD, 2019, KLIN PADIATR, V231, P248, DOI 10.1055/a-0963-8996

Yagci G, 2018, PHYSIOTHER THEOR PR, V34, P579, DOI 10.1080/09593985.2017.1423429

NR 22

TC 1

Z9 2

U1 1

U2 12

PU FRONTIERS MEDIA SA

PI LAUSANNE

PA AVENUE DU TRIBUNAL FEDERAL 34, LAUSANNE, CH-1015, SWITZERLAND

SN 2296-2360

J9 FRONT PEDIATR

JI Front. Pediatr.

PD JAN 25

PY 2021

VL 8

AR 609555

DI 10.3389/fped.2020.609555

PG 6

WC Pediatrics

WE Science Citation Index Expanded (SCI-EXPANDED)

SC Pediatrics

GA QD9BS

UT WOS:000615805900001

PM 33569361

OA Green Published, gold

DA 2023-08-10

ER

PT J

AU Wang, L

Wang, C

Youssef, ASA

Xu, J

Huang, XL

Xia, N

AF Wang, Li

Wang, Chun

Youssef, Ahmed S. A.

Xu, Jiang

Huang, Xiaolin

Xia, Nan

TI Physiotherapeutic scoliosis-specific exercises performed immediately

after spinal manipulative therapy for the treatment of mild adolescent

idiopathic scoliosis: study protocol for a randomized controlled pilot

trial

SO TRIALS

LA English

DT Article

DE Spinal manipulative therapy; Physical exercise; Adolescent idiopathic

scoliosis; Somatosensory evoked potentials

ID QUALITY-OF-LIFE; SENSORIMOTOR INTEGRATION; PAIN; EFFICACY; ASSOCIATION;

RELIABILITY; PREVALENCE; DEFORMITY; FREQUENCY; CHILDREN

AB Background Spinal manipulative therapy is commonly used in the treatment of adolescent idiopathic scoliosis. Some therapists also rely on physiotherapeutic scoliosis-specific exercise (PSSE). Combining these two modalities seems reasonable, but the effectiveness of this combination has never been rigorously tested. Here, a protocol for a pilot study is proposed to determine the feasibility of conducting a larger randomized trial. The pilot study was designed to test the hypothesis that spinal manipulative therapy followed by PSSE is more effective than PSSE alone in improving the Cobb angle, sensorimotor integration, the angle of trunk rotation (ATR), body symmetry, and quality of life.

Methods The protocol describes a randomized controlled pilot trial with 40 subjects divided into study and control groups. Both groups will receive 8 weeks of PSSE, but the study group will also receive spinal manipulative therapy during the first 2 weeks before PSSE. The primary outcome will

be an estimate of the feasibility of conducting a full-scale experiment. The influencing factors will be the time to complete enrollment, the recruitment rate, subject retention, and adherence to the treatment allocations. The secondary outcomes that will be used to assess the efficacy of treatment will include the Cobb angle, somatosensory evoked potentials, ATR, three-dimensional postural parameters, and scores on the 22-item Scoliosis Research Society outcomes questionnaire. The Cobb angle will be measured at baseline and at the end of 8 weeks of training. The somatosensory evoked potentials will be measured at baseline and at the end of 2 weeks of training. The ATR, three-dimensional postural parameters, and scores on the 22-item Scoliosis Research Society outcomes questionnaire will be measured at baseline and at 2 weeks, 4 weeks, and 8 weeks of treatment.

Discussion This study will inform the design of a future full-scale trial. The outcomes will provide preliminary data about the efficacy of the combination of spinal manipulative therapy and exercise in treating scoliosis.

C1 [Wang, Li; Wang, Chun; Youssef, Ahmed S. A.; Xu, Jiang; Huang, Xiaolin; Xia, Nan] Huazhong Univ Sci & Technol, Tongji Hosp, Dept Rehabil Med, Tongji Med Coll, Wuhan, china.

[Wang, Chun] Shantou Cent Hosp, Dept Rehabil Med, Shantou, china.

[Youssef, Ahmed S. A.] Beni Suef Univ, Fac Phys Therapy, Dept Basic Sci, Bani Suwayf, Egypt.

C3 Huazhong University of Science & Technology; Egyptian Knowledge Bank

(EKB); Beni Suef University

RP Huang, XL; Xia, N (通讯作者)， Huazhong Univ Sci & Technol, Tongji Hosp, Dept Rehabil Med, Tongji Med Coll, Wuhan, china.

EM xiaolinh2006@126.com; xianan@tjh.tjmu.edu.cn

RI Xia, Nan/ABG-4600-2021; YOUSSEF, AHMED/Z-1800-2019

OI YOUSSEF, AHMED/0000-0003-3225-0189; Xia, Nan/0000-0002-9820-7127

FU Tongji Hospital Foundation [2201300754]

FX The Tongji Hospital Foundation supports this work (grant number

2201300754). The study funder is not playing a role in study design,

data collection, or analyses.

CR AMENDT LE, 1990, PHYS THER, V70, P108, DOI 10.1093/ptj/70.2.108

Berdishevsky H, 2016, SCOLIOSIS SPINAL DIS, V11, DOI 10.1186/s13013-016-0076-9

Canavese F, 2011, INDIAN J ORTHOP, V45, P7, DOI 10.4103/0019-5413.73655

Carnes D, 2010, MANUAL THER, V15, P355, DOI 10.1016/j.math.2009.12.006

Chen KC, 2008, J ALTERN COMPLEM MED, V14, P749, DOI 10.1089/acm.2008.0054

Christiansen TL, 2018, EUR J APPL PHYSIOL, V118, P737, DOI 10.1007/s00421-018-3799-x

Domenech J, 2011, EUR SPINE J, V20, P1069, DOI 10.1007/s00586-011-1776-8

Ernst E, 2007, J ROY SOC MED, V100, P330, DOI 10.1258/jrsm.100.7.330

Fan HW, 2016, SPINE, V41, P259, DOI 10.1097/BRS.0000000000001197

Fortin C, 2012, PHYSIOTHERAPY, V98, P64, DOI 10.1016/j.physio.2010.12.006

Freidel K, 2002, SPINE, V27, pE87, DOI 10.1097/00007632-200202150-00013

Gross A, 2015, COCHRANE DB SYST REV, DOI 10.1002/14651858.CD004249.pub4

Haavik H, 2018, J ELECTROMYOGR KINES, V42, P24, DOI 10.1016/j.jelekin.2018.06.010

Haavik H, 2017, J MANIP PHYSIOL THER, V40, P127, DOI 10.1016/j.jmpt.2016.10.002

Haavik H, 2017, BRAIN SCI, V7, DOI 10.3390/brainsci7010002

Haavik H, 2012, J ELECTROMYOGR KINES, V22, P768, DOI 10.1016/j.jelekin.2012.02.012

Haavik-Taylor H, 2007, CLIN NEUROPHYSIOL, V118, P391, DOI 10.1016/j.clinph.2006.09.014

Hasler C, 2010, J CHILD ORTHOP, V4, P219, DOI 10.1007/s11832-010-0258-6

Kuru T, 2016, CLIN REHABIL, V30, P181, DOI 10.1177/0269215515575745

LAN KKG, 1989, BIOMETRICS, V45, P1017, DOI 10.2307/2531701

Lascurain-Aguirrebena I, 2016, SPINE, V41, P159, DOI 10.1097/BRS.0000000000001151

Law D, 2017, ERGONOMICS, V60, P876, DOI 10.1080/00140139.2016.1227093

Le Berre M, 2017, EUR SPINE J, V26, P1638, DOI 10.1007/s00586-016-4802-z

Lelic D, 2016, NEURAL PLAST, V2016, DOI 10.1155/2016/3704964

Li M, 2009, SPINE, V34, P1321, DOI 10.1097/BRS.0b013e31819812b7

Liang JP, 2018, TRIALS, V19, DOI 10.1186/s13063-018-2834-x

Little RJ, 2012, NEW ENGL J MED, V367, P1355, DOI 10.1056/NEJMsr1203730

Makino T, 2019, SPINE, V44, pE833, DOI 10.1097/BRS.0000000000002996

Millan M, 2012, CHIROPR MAN THER, V20, DOI 10.1186/2045-709X-20-23

Miller JE, 2008, J MANIP PHYSIOL THER, V31, P419, DOI 10.1016/j.jmpt.2008.06.002

Monticone M, 2014, EUR SPINE J, V23, P1204, DOI 10.1007/s00586-014-3241-y

MORRISSY RT, 1990, J BONE JOINT SURG AM, V72A, P320, DOI 10.2106/00004623-199072030-00002

Negrini S, 2005, Eura Medicophys, V41, P183

Negrini S, 2018, SCOLIOSIS SPINAL DIS, V13, DOI 10.1186/s13013-017-0145-8

NUWER MR, 1994, ELECTROEN CLIN NEURO, V91, P6, DOI 10.1016/0013-4694(94)90012-4

OBRIEN PC, 1979, BIOMETRICS, V35, P549, DOI 10.2307/2530245

Ogura Y, 2018, SCI REP-UK, V8, DOI 10.1038/s41598-018-22552-x

Paige NM, 2017, JAMA-J AM MED ASSOC, V317, P1451, DOI 10.1001/jama.2017.3086

Parent Stefan, 2005, Instr Course Lect, V54, P529

Pasquier M, 2019, CHIROPR MAN THER, V27, DOI 10.1186/s12998-019-0244-0

Peterson DH., 2002, CHIROPRACTIC TECHNIQ, V3, P188

Pialasse JP, 2016, J MANIP PHYSIOL THER, V39, P473, DOI 10.1016/j.jmpt.2016.06.001

Qiu GX, 2011, SPINE, V36, pE545, DOI 10.1097/BRS.0b013e3181e0485e

Reichert B., 2015, PALPATION TECHNIQUES, V2, P223

RISSER J C, 1958, Clin Orthop, V11, P111

Romano M, 2015, SCOLIOSIS SPINAL DIS, V10, DOI 10.1186/s13013-014-0027-2

Rowe Dale E, 2006, Chiropr Osteopat, V14, P15, DOI 10.1186/1746-1340-14-15

Sato T, 2011, EUR SPINE J, V20, P274, DOI 10.1007/s00586-010-1657-6

Simoneau M, 2006, EXP BRAIN RES, V170, P576, DOI 10.1007/s00221-005-0246-0

Taylor HH, 2007, EXP BRAIN RES, V178, P488, DOI 10.1007/s00221-006-0755-5

Theroux J, 2017, SPINE, V42, pE914, DOI 10.1097/BRS.0000000000001986

Todd AJ, 2015, J MANIP PHYSIOL THER, V38, P699, DOI 10.1016/j.jmpt.2014.09.008

Triano JJ, 2013, CHIROPR MAN THER, V21, DOI 10.1186/2045-709X-21-36

Wang ZH., 2001, TUINA MANIPULATION, V1, P41

Weinstein SL, 2008, LANCET, V371, P1527, DOI 10.1016/S0140-6736(08)60658-3

Weinstein SL, 2013, NEW ENGL J MED, V369, P1512, DOI 10.1056/NEJMoa1307337

Weiss HR, 2011, SCOLIOSIS SPINAL DIS, V6, DOI 10.1186/1748-7161-6-17

Wnuk Bartosz, 2015, Ortop Traumatol Rehabil, V17, P343, DOI 10.5604/15093492.1173376

Wood TG, 2001, J MANIP PHYSIOL THER, V24, P260, DOI 10.1067/mmt.2001.114365

[夏楠 Xia Nan], 2019, [中国康复医学杂志, Chinese Journal of Rehabilitation Medicine], V34, P1168

Yaszay B, 2017, EUR SPINE J, V26, P1658, DOI 10.1007/s00586-016-4694-y

Yim APY, 2013, INT J MOL SCI, V14, P6345, DOI 10.3390/ijms14036345

Youssef ASA, 2019, TRIALS, V20, DOI 10.1186/s13063-019-3337-0

[郑倩 Zheng Qian], 2019, [中国康复医学杂志, Chinese Journal of Rehabilitation Medicine], V34, P1178

Zheng Y, 2018, SPINE, V43, pE494, DOI 10.1097/BRS.0000000000002412

Zheng Y, 2017, J REHABIL MED, V49, P512, DOI 10.2340/16501977-2240

NR 66

TC 1

Z9 1

U1 3

U2 19

PU BMC

PI LONDON

PA CAMPUS, 4 CRINAN ST, LONDON N1 9XW, united kingdom

EI 1745-6215

J9 TRIALS

JI Trials

PD JAN 14

PY 2021

VL 22

IS 1

AR 58

DI 10.1186/s13063-020-05000-y

PG 12

WC Medicine, Research & Experimental

WE Science Citation Index Expanded (SCI-EXPANDED)

SC Research & Experimental Medicine

GA PW2NV

UT WOS:000610512200003

PM 33446270

OA gold, Green Published

DA 2023-08-10

ER

PT J

AU Lazic, I

Markovic, IP

Antunovic, SS

Nikolic, D

Aleksic, T

Bukumiric, D

AF Lazic, Irena

Markovic, Ivana Petronic

Antunovic, Sanja Sindjic

Nikolic, Dejan

Aleksic, Tanja

Bukumiric, Dragica

TI Influence of physical activity on prevention and occurrence of spinal

deformities in children during development

SO VOJNOSANITETSKI PREGLED

LA English

DT Article

DE adolescent; child; exercise; kyphosis; scoliosis; spine; spinal

curvatures; surveys and questionnaires

ID IDIOPATHIC SCOLIOSIS

AB Background/Aim. The published data indicate that the appearance of spinal deformities in children is significantly influenced by physical activity. The aim of our study was to examine the influence of physical activity on prevention and occurrence of spinal deformities in children. Methods. The study was conducted as observational, clinical study in the period from 2016 to 2018. Participants were children with spinal deformities, which were examined, for the first time, by physiatrists and pediatric surgeons. The sample included 100 children with spinal deformities, aged 7-17 years. The control group consisted of 100 children without spinal deformity, of similar age. The study instrument was a questionnaire based on a survey filled by children or parents/legal guardians. The questions were related to different parameters of the possible significance for the existence of spinal deformity and especially to the influence of physical activity. The collected data were processed using methods of descriptive and analytical statistics. Results. Scoliosis the most common deformity of the spinal column, represented in about 67% of children (p = 0.0006). Respondents from both groups did not differ significantly in terms of gender. Children in the group with spinal deformities were older (11.5 +/- 3.1 years vs. 10.4 +/- 3.1 years, p = 0.016), with increased body weight (43.9 +/- 16.0 kg vs. 39.3 +/- 16.6 kg, p = 0.046) and height (151.7 +/- 17.2 cm vs. 145.8 +/- 18.2 cm, p = 0.019), as well as with less physical activity (81.0% vs. 92.02%, p = 0.001). Over 80% of children were regularly engaged in physical activity, more often recreationally and on average 2.5-3 hours per week. Conclusion. Children in the spinal deformity group were significantly less involved in physical activity than the control group, but there was no significant difference in the frequency and duration of time spent in physical activities during the week. It is important for children to be involved in physical activities of a recreational nature, and according to our research, 3 hours during the week.

C1 [Lazic, Irena; Markovic, Ivana Petronic; Antunovic, Sanja Sindjic; Nikolic, Dejan] Univ Childrens Hosp, Tirsova 10, Belgrade 11000, Serbia.

[Markovic, Ivana Petronic; Antunovic, Sanja Sindjic; Nikolic, Dejan] Univ Belgrade, Fac Med, Belgrade, Serbia.

[Aleksic, Tanja; Bukumiric, Dragica] Healthcare Facil Pancevo, Pancevo, Serbia.

C3 University of Belgrade; University of Belgrade

RP Lazic, I (通讯作者)，Univ Childrens Hosp, Tirsova 10, Belgrade 11000, Serbia.

EM socijalnamedicina74@gmail.com

OI Nikolic, Dejan/0000-0002-0609-9916

CR Abbott A, 2013, BMC MUSCULOSKEL DIS, V14, DOI 10.1186/1471-2474-14-261

*AM COLL SPORTS ME, 1988, MED SCI SPORTS EXERC, V20, P422, DOI DOI 10.1249/00005768-198808000-00022

Anwer S, 2015, BIOMED RES INT-UK, V2015, DOI 10.1155/2015/123848

Baxter-Jones ADG, 2003, BRIT J SPORT MED, V37, P96, DOI 10.1136/bjsm.37.2.96

Bettany-Saltikov J, 2016, ASIAN SPINE J, V10, P1170, DOI 10.4184/asj.2016.10.6.1170

Determinants of Health, HLTH PEOPL 2020

Fearnbach SN, 2016, NUTR J, V15, DOI 10.1186/s12937-016-0206-5

Fernandez I, 2017, EUR J PEDIATR, V176, P57, DOI 10.1007/s00431-016-2809-4

Gunawardena N, 2016, INT J BEHAV NUTR PHY, V13, DOI 10.1186/s12966-016-0369-7

Hawes MC, 2006, SCOLIOSIS SPINAL DIS, V1, DOI 10.1186/1748-7161-1-3

Kakar Rumit Singh, 2017, Int J Exerc Sci, V10, P166

Lizak-Popiolek D, 2014, SCI REV PHYS CULTURE, V4

McMaster MJ, 2006, SPINE, V31, P2284, DOI 10.1097/01.brs.0000238975.90422.c4

Plaszewski M, 2015, J BACK MUSCULOSKELET, V28, P453, DOI 10.3233/BMR-140540

Schulze A, 2015, ORTHOPADE, V44, P836, DOI 10.1007/s00132-015-3165-1

Sedrez Juliana Adami, 2015, Rev. paul. pediatr., V33, P72, DOI 10.1016/j.rpped.2014.11.012

Tsirikos AI, 2011, J BONE JOINT SURG BR, V93B, P857, DOI 10.1302/0301-620X.93B7.26129

NR 17

TC 1

Z9 1

U1 2

U2 4

PU MILITARY MEDICAL ACAD-INI

PI BELGRADE

PA CRNOTRAVSKA 17, PO BOX 33-35, BELGRADE, 11040, SERBIA

SN 0042-8450

EI 2406-0720

J9 VOJNOSANIT PREGL

JI Vojnosanit. Pregl.

PY 2021

VL 78

IS 7

BP 730

EP 735

DI 10.2298/VSP190702127L

PG 6

WC Medicine, General & Internal

WE Science Citation Index Expanded (SCI-EXPANDED)

SC General & Internal Medicine

GA UE7OV

UT WOS:000688074000005

OA gold

DA 2023-08-10

ER

PT J

AU Won, SH

Oh, DW

Shen, M

AF Won, Sang-Hee

Oh, Duck-Won

Shen, Min

TI An 18-month follow-up study on the effect of a neuromuscular

stabilization technique on Cobb's angle in adolescent idiopathic

scoliosis: A single-blind, age-matched controlled trial

SO JOURNAL OF BACK AND MUSCULOSKELETAL REHABILITATION

LA English

DT Article

DE Neuromuscular stabilization technique; Cobb's angle; Scoliosis; Spinal

alignment

ID EXERCISE

AB BACKGROUND: The benefits of spinal realignment and stabilization in scoliosis need to be examined.

OBJECTIVE: We aimed to investigate the long-term effect of a neuromuscular stabilization technique (NST) on Cobb's angle in patients with adolescent idiopathic scoliosis.

METHODS: Twenty females recruited from two hospitals participated in this study. On the basis of convenience of location, participants were allocated to either the experimental group (EG) that underwent the NST, or the control group (CG) that received education for a home exercise program. The NST for the EG was performed for an average of 30 min per session, three times a week for six months, and consisted of spinal realignment and stabilization. Then, 12- and 18-month measurements for long-term follow-ups were conducted for the EG. The outcome measure was Cobb's angle.

RESULTS: Between-group comparison revealed a statistically significant difference at post-test (t = -3.26, p < 0.01) but not pre-test (t = -1.36, p = 0.19). Participants of the EG (-6.20 +/- 2.49 degrees) showed greater differences between pre- and post-test scores compared to participants of the CG (-1.40 +/- 0.52 degrees) (p < 0.05). Within-group comparisons showed a significant difference in both groups (p < 0.05). In the EG, Cobb's angle significantly changed across the follow-up sessions (p <

0.05), indicating more improvements by the 12-month (8.50 +/- 4.03 degrees) and 18-month (6.60 +/- 3.89 degrees) follow-ups.

CONCLUSION: This study shows that the NST may be a beneficial option to correct spinal alignments in patients with adolescent idiopathic scoliosis.

C1 [Won, Sang-Hee] NCR Sling Exercise Ctr, Dongtan, South Korea.

[Oh, Duck-Won] Cheongju Univ, Coll Hlth & Med Sci, Dept Phys Therapy, 298 Daeseong Ro, Cheongju 28503, Chungcheongbuk, South Korea.

[Shen, Min] Shanghai Rehabil & Vocat Training Ctr Disabled, Shanghai, china.

C3 Cheongju University

RP Oh, DW (通讯作者)， Cheongju Univ, Coll Hlth & Med Sci, Dept Phys Therapy, 298 Daeseong Ro, Cheongju 28503, Chungcheongbuk, South Korea.

EM odduck@cju.ac.kr

CR Bruyneel AV, 2011, EUR SPINE J, V20, P247, DOI 10.1007/s00586-010-1574-8

Cheon M, 2013, EURASIP J WIREL COMM, DOI 10.1186/1687-1499-2013-132

Coley BD, 2013, CAFFEYS PEDIAT DIAGN, P1429

Durmala J, 2015, INT J ENV RES PUB HE, V12, P9444, DOI 10.3390/ijerph120809444

Fusco C., 2011, Physiotherapy Theory and Practice, V27, P80, DOI 10.3109/09593985.2010.533342

Gstoettner M, 2007, EUR SPINE J, V16, P1587, DOI 10.1007/s00586-007-0401-3

Gur G, 2017, PROSTHET ORTHOT INT, V41, P303, DOI 10.1177/0309364616664151

Horne JP, 2014, AM FAM PHYSICIAN, V89, P193

Huh Seokwon, 2015, Korean J Pediatr, V58, P218, DOI 10.3345/kjp.2015.58.6.218

Julie AH, 2001, SPINE, V26, P243

Kim EY, 2012, CLIN REHABIL, V26, P132, DOI 10.1177/0269215511411498

Kisner C, 2012, THERAPEUTIC EXERCISE, P498

Koumbourlis AC, 2006, PAEDIATR RESPIR REV, V7, P152, DOI 10.1016/j.prrv.2006.04.009

Malfair D, 2010, AM J ROENTGENOL, V194, pS8, DOI 10.2214/AJR.07.7145

Maruyama T, 2009, STUD HEALTH TECHNOL, V135, P246

Negrini S, 2006, SCOLIOSIS SPINAL DIS, V1, DOI 10.1186/1748-7161-1-14

Negrini S, 2012, SCOLIOSIS SPINAL DIS, V7, DOI 10.1186/1748-7161-7-3

Oh DW, 2017, KOR J NEUROMUSCUL RE, V7, P26

Otman S, 2005, SAUDI MED J, V26, P1429

Park JH, 2018, EUR J PHYS REHAB MED, V54, P440, DOI 10.23736/S1973-9087.17.04461-6

Romano M, 2012, COCHRANE DB SYST REV, DOI 10.1002/14651858.CD007837.pub2

Saraiva BMA, 2018, PEDIATR EXERC SCI, V30, P243, DOI 10.1123/pes.2017-0080

Schreiber S, 2016, PLOS ONE, V11, DOI 10.1371/journal.pone.0168746

Silva FE., 2009, SURG MANAGEMENT SPIN, P97, DOI 10.1016/B978-141603372-1.50010-X

Smith Jason R, 2008, JAAPA, V21, P40

Sperandio EF, 2014, SPINE J, V14, P2366, DOI 10.1016/j.spinee.2014.01.041

Stokes IAF, 2006, SCOLIOSIS SPINAL DIS, V1, DOI 10.1186/1748-7161-1-16

Wang Qiang, 2018, CHIN J PHYS MED REHA, V40, P644

Weiss H R., 2013, OA MUSCULOSKELETAL M, V3, P1

Westrick ER, 2011, J PEDIATR ORTHOPED, V31, pS61, DOI 10.1097/BPO.0b013e3181fd87d5

Won SH, 2016, EFFECTS SEGMENTAL TR

NR 31

TC 2

Z9 2

U1 1

U2 6

PU IOS PRESS

PI AMSTERDAM

PA NIEUWE HEMWEG 6B, 1013 BG AMSTERDAM, NETHERLANDS

SN 1053-8127

EI 1878-6324

J9 J BACK MUSCULOSKELET

JI J. Back Musculoskelet. Rehabil.

PY 2021

VL 34

IS 1

BP 87

EP 93

DI 10.3233/BMR-191559

PG 7

WC Orthopedics; Rehabilitation

WE Science Citation Index Expanded (SCI-EXPANDED)

SC Orthopedics; Rehabilitation

GA PX7SK

UT WOS:000611554000011

PM 32986652

OA Bronze

DA 2023-08-10

ER

PT J

AU Li, X

Shen, J

Liang, JP

Zhou, X

Yang, YQ

Wang, DX

Wang, SS

Wang, LX

Wang, H

Du, Q

AF Li, Xin

Shen, Jie

Liang, Juping

Zhou, Xuan

Yang, Yuqi

Wang, Dexuan

Wang, Shanshan

Wang, Lixia

Wang, Hong

Du, Qing

TI Effect of core-based exercise in people with scoliosis: A systematic review and meta-analysis

SO CLINICAL REHABILITATION

LA English

DT Review

DE Scoliosis; core-based exercise; systematic review; meta-analysis

ID ADOLESCENT IDIOPATHIC SCOLIOSIS; QUALITY-OF-LIFE; STABILIZATION EXERCISE; SPINE DEFORMATION; SCHROTH EXERCISES; RELIABILITY; VALIDITY; PROGRESSION; CURVATURE; STRENGTH

AB Objective: To systematically assess the effectiveness of core-based exercise for correcting a spinal deformity and improving quality of life in people with scoliosis.

Data sources: The PubMed, Embase, Cochrane Library, Cumulative Index of Nursing and Allied Health Literature (CINAHL), and Web of Science databases were searched from inception up to September 30, 2020.

Methods: Clinical controlled trials were eligible if they compared the effectiveness of core-based exercise to other nonsurgical interventions in people with scoliosis. The revised Cochrane risk of bias assessment tool for randomized trials and the methodological index for non-randomized studies scale were used to assess the risk of bias. The outcomes included the Cobb angle, the angle of trunk rotation and quality of life. RevMan 5.3 was used, and intergroup differences were determined by calculating mean differences (MD) and 95% confidence intervals (CIs).

Results: After screening 1348 studies, nine studies with 325 participants met the inclusion criteria. The exercise group had significantly lower Cobb angles (MD = -2.08, 95% CI: -3.89 to -0.28, P = 0.02) and significantly better quality of life as measured by the Scoliosis Research Society-22 questionnaire (MD = 0.25, 95% CI: 0.02 to 0.49, P = 0.03) than the control groups. However, no significant difference was observed regarding the angle of trunk rotation between groups (MD = -0.69, 95% CI: -2.61 to 1.22, P = 0.48). Furthermore, no serious adverse events were reported. The overall quality of evidence ranged from low to very low.

Conclusion: Core-based exercise may have a beneficial role in reducing the Cobb angle and improving quality of life in people with scoliosis in the short term.

C1 [Li, Xin; Liang, Juping; Zhou, Xuan; Du, Qing] Shanghai Jiao Tong Univ, Xinhua Hosp, Sch Med, Dept Rehabil, 1665 Kongjiang Rd, Shanghai 200092, china.

[Li, Xin; Shen, Jie; Wang, Lixia] Shanghai Univ Sport, Sch Kinesiol, Shanghai, china.

[Yang, Yuqi; Wang, Hong] Shanghai Univ Med & Hlth Sci, 279 Zhouzhu Rd, Shanghai 201318, china.

[Wang, Dexuan; Wang, Shanshan] Shanghai Jiao Tong Univ, Xinhua Hosp, Sch Med, Shanghai, china.

[Du, Qing] Shanghai Jiao Tong Univ, Sch Med, Xinhua Hosp, Dept Rehabil,Chongming Branch, Shanghai, china.

C3 Shanghai Jiao Tong University; Shanghai University of Sport; Shanghai

University of Medicine & Health Sciences; Shanghai Jiao Tong University;

Shanghai Jiao Tong University

RP Du, Q (通讯作者)， Shanghai Jiao Tong Univ, Xinhua Hosp, Sch Med, Dept Rehabil, 1665 Kongjiang Rd, Shanghai 200092, china.; Wang, H (通讯作者)， Shanghai Univ Med & Hlth Sci, 279 Zhouzhu Rd, Shanghai 201318, china.

EM wanghongplus@163.com; duqing@xinhuamed.com.cn

RI Du, Qing/HKN-6976-2023

OI Shen, Jie/0000-0001-5426-1036

FU General Program of National Natural Science Foundation of China [81972030]; Advanced and Appropriate Technology Promotion Projects of Shanghai Municipal Health Commission [2019SY021]; Chongming District Medical Key Specialty Project

FX The author(s) disclosed receipt of the following financial support for the research, authorship, and/or publication of this article: This work was supported by the General Program of National Natural Science Foundation of China (81972030), Advanced and Appropriate Technology Promotion Projects of Shanghai Municipal Health Commission (2019SY021), and Chongming District Medical Key Specialty Project.

CR Alanazi MH, 2018, EUR J PHYS REHAB MED, V54, P647, DOI 10.23736/S1973-9087.17.05062-6

de Araujo MEA, 2012, J BODYW MOV THER, V16, P191, DOI 10.1016/j.jbmt.2011.04.002

AMENDT LE, 1990, PHYS THER, V70, P108, DOI 10.1093/ptj/70.2.108

Asher M, 2003, SPINE, V28, P63, DOI 10.1097/00007632-200301010-00015

Ayhan C, 2014, CLIN REHABIL, V28, P36, DOI 10.1177/0269215513492443

Bachmann KR, 2019, CHILD NERV SYST, V35, P1585, DOI 10.1007/s00381-019-04239-4

Balague F, 2016, SCOLIOSIS SPINAL DIS, V11, DOI 10.1186/s13013-016-0086-7

Berdishevsky H, 2016, SCOLIOSIS SPINAL DIS, V11, DOI 10.1186/s13013-016-0076-9

Carreon LY, 2010, SPINE, V35, P2079, DOI 10.1097/BRS.0b013e3181c61fd7

Chan JSY, 2019, J PSYCHOL, V153, P102, DOI 10.1080/00223980.2018.1470487

Cheon M, 2013, EURASIP J WIREL COMM, DOI 10.1186/1687-1499-2013-132

Coulombe BJ, 2017, J ATHL TRAINING, V52, P71, DOI 10.4085/1062-6050-51.11.16

Delamothe T, 2004, BMJ-BRIT MED J, V328, P1, DOI 10.1136/bmj.328.7430.1

Gao CF, 2019, AM J PHYS MED REHAB, V98, P642, DOI 10.1097/PHM.0000000000001160

Gur G, 2017, PROSTHET ORTHOT INT, V41, P303, DOI 10.1177/0309364616664151

Hawes MC, 2006, SCOLIOSIS SPINAL DIS, V1, DOI 10.1186/1748-7161-1-3

Higgins JPT, 2003, BMJ-BRIT MED J, V327, P557, DOI 10.1136/bmj.327.7414.557

Hozo S.P., 2005, BMC MED RES METHODOL, V5, DOI DOI 10.1186/1471-2288-5-13

Hresko MT, 2016, J BONE JOINT SURG AM, V98, DOI 10.2106/JBJS.16.00224

Hutton B, 2015, ANN INTERN MED, V162, P777, DOI 10.7326/M14-2385

JAMES JIP, 1954, J BONE JOINT SURG BR, V36, P36, DOI 10.1302/0301-620X.36B1.36

Kim SY, 2013, J CLIN EPIDEMIOL, V66, P408, DOI 10.1016/j.jclinepi.2012.09.016

Ko KJ, 2017, J EXERC REHABIL, V13, P244, DOI 10.12965/jer.1734952.476

Kuru T, 2016, CLIN REHABIL, V30, P181, DOI 10.1177/0269215515575745

Leal-Hernandez M, 2018, Semergen, V44, P227, DOI 10.1016/j.semerg.2016.11.011

Lee M, 2015, J PHYS THER SCI, V27, P1519, DOI 10.1589/jpts.27.1519

Marshall PWM, 2011, J STRENGTH COND RES, V25, P3404, DOI 10.1519/JSC.0b013e318215fc49

Negrini A, 2016, SCOLIOSIS SPINAL DIS, V11, DOI 10.1186/s13013-016-0100-0

Negrini S, 2014, EUR J PHYS REHAB MED, V50, P87

Negrini S, 2008, J REHABIL MED, V40, P451, DOI 10.2340/16501977-0195

Noshchenko A, 2015, WORLD J ORTHOP, V6, P537, DOI 10.5312/wjo.v6.i7.537

Park JH, 2018, EUR J PHYS REHAB MED, V54, P440, DOI 10.23736/S1973-9087.17.04461-6

Park Y, 2014, J PHYS THER SCI, V26, P1157, DOI 10.1589/jpts.26.1157

Romano M., 2007, SCOLIOSIS, V2, pS8

Romano M, 2013, SPINE, V38, pE883, DOI 10.1097/BRS.0b013e31829459f8

Schreiber S, 2016, PLOS ONE, V11, DOI 10.1371/journal.pone.0168746

Schreiber S, 2015, SCOLIOSIS SPINAL DIS, V10, DOI 10.1186/s13013-015-0048-5

Smith JS, 2008, J NEUROSURG-SPINE, V9, P326, DOI 10.3171/SPI.2008.9.10.326

Standaert CJ, 2008, SPINE J, V8, P114, DOI 10.1016/j.spinee.2007.10.015

Stang A, 2010, EUR J EPIDEMIOL, V25, P603, DOI 10.1007/s10654-010-9491-z

Sterne JAC, 2019, BMJ-BRIT MED J, V366, DOI 10.1136/bmj.l4898

Vasiliadis E, 2009, STUD HEALTH TECHNOL, V135, P409

Villemure I, 2004, EUR SPINE J, V13, P83, DOI 10.1007/s00586-003-0565-4

Wong E, 2017, ORTHOPEDICS, V40, pE930, DOI 10.3928/01477447-20170606-02

Wong HK, 2005, SPINE, V30, P1188, DOI 10.1097/01.brs.0000162280.95076.bb

Wu HD, 2020, EUR SPINE J, V29, P717, DOI 10.1007/s00586-019-06280-y

Yagci G, 2019, PROSTHET ORTHOT INT, V43, P301, DOI 10.1177/0309364618820144

Yang JM, 2015, J PHYS THER SCI, V27, P2667, DOI 10.1589/jpts.27.2667

Yesil H, 2018, SPINE, V43, pE1174, DOI 10.1097/BRS.0000000000002663

Zapata KA, 2019, PEDIATR PHYS THER, V31, P280, DOI 10.1097/PEP.0000000000000621

Zheng Y, 2018, SPINE, V43, pE494, DOI 10.1097/BRS.0000000000002412

NR 51

TC 8

Z9 10

U1 3

U2 18

PU SAGE PUBLICATIONS LTD

PI LONDON

PA 1 OLIVERS YARD, 55 CITY ROAD, LONDON EC1Y 1SP, united kingdom

SN 0269-2155

EI 1477-0873

J9 CLIN REHABIL

JI Clin. Rehabil.

PD MAY

PY 2021

VL 35

IS 5

BP 669

EP 680

DI 10.1177/0269215520975105

EA DEC 2020

PG 12

WC Rehabilitation

WE Science Citation Index Expanded (SCI-EXPANDED)

SC Rehabilitation

GA RR7QT

UT WOS:000630801700001

PM 33356498

OA hybrid, Green Published

DA 2023-08-10

ER

PT J

AU Ogonowska-Slodownik, A

Kaczmarczyk, K

Kokowicz, G

Morgulec-Adamowicz, N

AF Ogonowska-Slodownik, Anna

Kaczmarczyk, Katarzyna

Kokowicz, Gabriela

Morgulec-Adamowicz, Natalia

TI Does the Aquatic Breathing Program Improve Lung Function in Adolescents

with Scoliosis?

SO PHYSICAL & OCCUPATIONAL THERAPY IN PEDIATRICS

LA English

DT Article

DE Aquatic breathing program; aquatic therapy; respiratory muscles;

scoliosis; spirometry

AB Aim: To determine pulmonary parameters in adolescents with scoliosis after three weeks of intensive physiotherapeutic scoliosis-specific exercises based on the Aquatic Breathing Program (ABP) compared to corrective swimming (CS).

Methods: A pretest-posttest control group design was used. The ABP group comprised 13 adolescents (age 14.2 +/- 1.4) and the CS group 10 adolescents (age 14.1 +/- 1.5) with mild to moderate scoliosis. Both groups participated in a threeweek intervention consisted of three types of exercises: corrective, general and aquatic. ABP group participated in the Aquatic Breathing Program and CS group took part in corrective swimming. Spirometric measurements were taken before and after the intervention.

Results: ABP group demonstrated a greater increase (p = .05) in the predicted percentage values of forced expiratory volume in 1 s (FEV1) and maximal expiratory flow at 25% of forced vital capacity (MEF25), both in absolute terms and in percentage values (MEF25%pred) compared to the CS group. CS group exhibited lower values of all measured parameters in the second examination, and some of those differences were found to be statistically significant.

Conclusions: The Aquatic Breathing Program can be used in adolescents with mild to moderate scoliosis to improve lung function.

C1 [Ogonowska-Slodownik, Anna; Kaczmarczyk, Katarzyna; Morgulec-Adamowicz, Natalia] Jozef Pilsudski Univ Phys Educ Warsaw, Fac Rehabil, Marymoncka 34, PL-00968 Warsaw, Poland.

[Kokowicz, Gabriela] Childrens Mem Hlth Inst, Dept Rehabil, Miedzylesie, Poland.

C3 Jozef Pilsudski University Physical Education in Warsaw; Children's

Memorial Health Institute

RP Ogonowska-Slodownik, A (通讯作者)， Jozef Pilsudski Univ Phys Educ Warsaw, Fac Rehabil, Marymoncka 34, PL-00968 Warsaw, Poland.

EM anna.ogonowskaslodownik@gmail.com

RI Kaczmarczyk, Katarzyna/A-9073-2018; Morgulec-Adamowicz,

Natalia/G-3404-2017; Ogonowska-Slodownik, Anna/A-9755-2018

OI Kaczmarczyk, Katarzyna/0000-0003-4110-8571; Morgulec-Adamowicz,

Natalia/0000-0003-3888-633X; Ogonowska-Slodownik,

Anna/0000-0002-3318-6513; Kokowicz, Gabriela/0000-0001-8224-1454

CR Armijo-Olivo S, 2020, AM J PHYS MED REHAB, V99, P198, DOI 10.1097/PHM.0000000000001377

Barczyk Katarzyna, 2009, Ortop Traumatol Rehabil, V11, P209

Barczyk-Pawelec K., 2012, ACTA BIOOPT INFORM M, V18, P9

Bush, 2006, KENDIGS DISORDERS RE, P733

CAMPBELL RM, 2004, J BONE JOINT SURG, V86

Dziubek, 2005, PHYSIOTHERAPY, V13, P50

Fusco C., 2011, Physiotherapy Theory and Practice, V27, P80, DOI 10.3109/09593985.2010.533342

Ghanem I, 2019, CURR OPIN PEDIATR, V31, P48, DOI 10.1097/MOP.0000000000000705

Hawes Martha C, 2003, Pediatr Rehabil, V6, P171

Koumbourlis AC, 2006, PAEDIATR RESPIR REV, V7, P152, DOI 10.1016/j.prrv.2006.04.009

Kumar A, 2017, J CLIN DIAGN RES, V11, pYC1, DOI 10.7860/JCDR/2017/27497.10335

Lin MC, 2001, ARCH PHYS MED REHAB, V82, P335, DOI 10.1053/apmr.2001.21528

Lin YX, 2019, J BONE JOINT SURG AM, V101, P1109, DOI 10.2106/JBJS.18.00935

Lubkowska W, 2014, CENTR EUR J SPORT SC, V6, P93

Malmivaara A, 2020, AM J PHYS MED REHAB, V99, P183, DOI 10.1097/PHM.0000000000001369

McPhail GL, 2015, J PEDIATR-US, V166, P1018, DOI 10.1016/j.jpeds.2014.12.070

Moramarco M, 2016, CURR PEDIATR REV, V12, P17, DOI 10.2174/1573396312666151117120514

Negrini S, 2018, SCOLIOSIS SPINAL DIS, V13, DOI 10.1186/s13013-017-0145-8

Neve V, 2002, EUR RESPIR J, V20, P1292, DOI 10.1183/09031936.02.00208102

Pendergast DR, 2015, COMPR PHYSIOL, V5, P1705, DOI 10.1002/cphy.c140018

Redding G, 2017, SPINE, V42, P1799, DOI 10.1097/BRS.0000000000002351

Szopa A, 2017, MEDICINE, V96, DOI 10.1097/MD.0000000000007032

Tsiligiannis T, 2012, SCOLIOSIS SPINAL DIS, V7, DOI 10.1186/1748-7161-7-7

WANG X, 1993, PEDIATR PULM, V15

Watanabe K, 2017, J BONE JOINT SURG AM, V99, P284, DOI 10.2106/JBJS.16.00459

Watkins M.P., 2009, FDN CLIN RES APPL PR, P733

WEINSTEIN SL, 2008, LANCET LONDON ENGLAN, V371

Zaina F, 2015, J PEDIATR-US, V166, P163, DOI 10.1016/j.jpeds.2014.09.024

NR 28

TC 1

Z9 1

U1 0

U2 11

PU TAYLOR & FRANCIS INC

PI PHILADELPHIA

PA 530 WALNUT STREET, STE 850, PHILADELPHIA, PA 19106 USA

SN 0194-2638

EI 1541-3144

J9 PHYS OCCUP THER PEDI

JI Phys. Occup. Ther. Pediatr.

PD DEC 2

PY 2020

VL 41

IS 3

BP 259

EP 270

DI 10.1080/01942638.2020.1856285

EA DEC 2020

PG 12

WC Pediatrics; Rehabilitation

WE Science Citation Index Expanded (SCI-EXPANDED); Social Science Citation Index (SSCI)

SC Pediatrics; Rehabilitation

GA QU2TB

UT WOS:000601010600001

PM 33350882

DA 2023-08-10

ER

PT J

AU Caviedes, JE

Li, BX

Jammula, VC

AF Caviedes, Jorge E.

Li, Baoxin

Jammula, Varun C.

TI Wearable Sensor Array Design for Spine Posture Monitoring During

Exercise Incorporating Biofeedback

SO IEEE TRANSACTIONS ON BIOMEDICAL ENGINEERING

LA English

DT Article

DE Biofeedback; soft sensors; spine posture monitoring; stretch sensors;

therapeutic exercise; wearable sensors

ID LOW-BACK-PAIN; ADOLESCENT IDIOPATHIC SCOLIOSIS

AB Physical therapy (PT) exercise is an evidence-based intervention for non-specific chronic low back pain, spinal deformities and poor posture. Home based PT programs are aimed at strengthening core muscle groups, improving mobility and flexibility, and promoting proper posture. However, assessing unsupervised home-based PT outcomes is a generally difficult problem due to lack of reliable methods to monitor execution correctness and compliance. We propose a monitoring method consisting of a wearable sensor array to monitor three geodesic distances between two points on the surface of the shoulders and one point on the lower back. The sensor array may be built into a custom garment or a light weight harness wirelessly linked to a pattern recognition algorithm implemented in a mobile app. We use a new type of triangular stretch sensor array design which can generate a unique signature for a correct spine therapy exercise when performed by a specific subject. We conducted a pilot test consisting of three experiments: (i) two exercise patterns simulated by a mechanical device, (ii) one PT case of a scoliosis therapy exercise including spinal flexion, extension, and rotation performed by one volunteer patient, and (iii) a set of three lower back flexibility exercises performed by six subjects. Overall, the results of correctness recognition show 70-100% sensitivity and 100% specificity. The pilot test provides key data for further development including clinical trials. The significance of the method includes simplicity of design and training method, ability to test with simulated signals, and potential to provide real time biofeedback.

C1 [Caviedes, Jorge E.; Li, Baoxin; Jammula, Varun C.] Arizona State Univ, Tempe, AZ 85281 USA.

C3 Arizona State University; Arizona State University-Tempe

RP Caviedes, JE (通讯作者)， Arizona State Univ, Tempe, AZ 85281 USA.

EM jorge.caviedes@asu.edu

RI Lu, Xiaomei/IUQ-2139-2023; Caviedes, Jorge/ABI-2562-2020; Liu,

Jian/IQS-8197-2023; Xu, Chen/AAQ-5514-2020; YU, Liang/IYT-4334-2023; li,

xiao/HKV-8405-2023

OI Xu, Chen/0000-0001-8068-2109; YU, Liang/0009-0007-3922-3454; Jammula,

Varun Chandra/0000-0002-1249-8534; Caviedes, Jorge/0000-0003-0102-6782

FU Arizona State University Global Sport Institute; Fulton School of

Engineering Research faculty Support Seed Program

FX This work was supported by seed grants from Arizona State University

Global Sport Institute and the Fulton School of Engineering Research

faculty Support Seed Program.

CR Anar S.O., 2016, J PHYS THER SCI, V28, DOI [10.1186/s13013-016-0076-9, DOI 10.1186/S13013-016-0076-9]

Antal A., 2015, OPTICS, V4, P24

Asamoah V, 2000, ORTHOPADE, V29, P480, DOI 10.1007/s001320050486

Bagraith KS, 2017, DISABIL HEALTH J, V10, P621, DOI 10.1016/j.dhjo.2017.01.001

Bentsen H, 1997, SPINE, V22, P1494, DOI 10.1097/00007632-199707010-00014

Berdishevsky H, 2016, SCOLIOSIS SPINAL DIS, V11, DOI 10.1186/s13013-016-0076-9

Betsch M, 2013, PLOS ONE, V8, DOI 10.1371/journal.pone.0070581

Betsch M, 2011, COMPUT BIOL MED, V41, P308, DOI 10.1016/j.compbiomed.2011.03.008

Bulling A, 2014, ACM COMPUT SURV, V46, DOI 10.1145/2499621

Cao H, 2012, UBICOMP'12: PROCEEDINGS OF THE 2012 ACM INTERNATIONAL CONFERENCE ON UBIQUITOUS COMPUTING, P331

Cohen L, 2017, SCOLIOSIS SPINAL DIS, V12, DOI 10.1186/s13013-017-0135-x

Coillard C, 2008, DISABIL REHABIL-ASSI, V3, P112, DOI 10.1080/17483100801903913

Dunne LE, 2008, IEEE T BIOMED CIRC S, V2, P97, DOI 10.1109/TBCAS.2008.927246

DWORKIN B, 1985, P NATL ACAD SCI USA, V82, P2493, DOI 10.1073/pnas.82.8.2493

Fathi A, 2017, J KING SAUD UNIV SCI, V29, P553, DOI 10.1016/j.jksus.2017.09.014

Fritz JM, 2008, SPINE, V33, P1800, DOI 10.1097/BRS.0b013e31817bd853

Giggins OM, 2013, J NEUROENG REHABIL, V10, DOI 10.1186/1743-0003-10-60

Goodvin C, 2006, MED BIOL ENG COMPUT, V44, P1061, DOI 10.1007/s11517-006-0132-3

Goras L., 2010, PHYS CONTROL EMERGEN, V15, P20

Humbert L, 2009, MED ENG PHYS, V31, P681, DOI 10.1016/j.medengphy.2009.01.003

Jack K, 2010, MANUAL THER, V15, P220, DOI 10.1016/j.math.2009.12.004

Kavcic N, 2004, SPINE, V29, P1254, DOI 10.1097/00007632-200406010-00016

Kisner C., 1990, THERAPEUTIC EXERCISE, V7th, P429

Kos A, 2018, HUM-COMPUT INT-SPRIN, P1, DOI 10.1007/978-3-319-91349-0

Kuru T, 2016, CLIN REHABIL, V30, P181, DOI 10.1177/0269215515575745

Kwok G, 2015, BIOMED RES INT, V2015, DOI 10.1155/2015/958450

Lockhart Thurmon E, 2013, Biomed Sci Instrum, V49, P224

Lynn SK, 2018, J SPORT SCI MED, V17, P205

Nag A, 2017, IEEE SENS J, V17, P3949, DOI 10.1109/JSEN.2017.2705700

Papi E, 2017, J BIOMECH, V64, P186, DOI 10.1016/j.jbiomech.2017.09.037

Pop-Jordanova N., 2010, MACED J MED SCI, V3, P113, DOI [DOI 10.3889/MJMS.1957-5773.2010.0098, DOI 10.3889/MJMS.1857-5773.2010.0098]

Santiesteban Y., 2006, LECT NOTES COMPUTER, V4225

Sardini E, 2015, IEEE T INSTRUM MEAS, V64, P439, DOI 10.1109/TIM.2014.2343411

Tu ZG, 2016, INT C PATT RECOG, P3524, DOI 10.1109/ICPR.2016.7900180

Wibmer C, 2016, SCOLIOSIS SPINAL DIS, V11, DOI 10.1186/s13013-016-0104-9

NR 35

TC 13

Z9 13

U1 2

U2 25

PU IEEE-INST ELECTRICAL ELECTRONICS ENGINEERS INC

PI PISCATAWAY

PA 445 HOES LANE, PISCATAWAY, NJ 08855-4141 USA

SN 0018-9294

EI 1558-2531

J9 IEEE T BIO-MED ENG

JI IEEE Trans. Biomed. Eng.

PD OCT

PY 2020

VL 67

IS 10

BP 2828

EP 2838

DI 10.1109/TBME.2020.2971907

PG 11

WC Engineering, Biomedical

WE Science Citation Index Expanded (SCI-EXPANDED)

SC Engineering

GA NR7LG

UT WOS:000571741600013

PM 32031929

DA 2023-08-10

ER

PT J

AU Celebioglu, E

Yataganbaba, A

Bekmez, S

Oncel, A

Degirmenci, C

Tekin, F

Demirkiran, G

Yalcin, E

Demir, AU

Yazici, M

AF Celebioglu, Ebru

Yataganbaba, Alper

Bekmez, Senol

Oncel, Asli

Degirmenci, Ceren

Tekin, Fatih

Demirkiran, Gokhan

Yalcin, Ebru

Demir, Ahmet U.

Yazici, Muharrem

TI Growing-rod Graduates With Idiopathic Early-onset Scoliosis Have Comparable Exercise Tolerance to Patients With Surgically Treated Adolescent Idiopathic Scoliosis

SO JOURNAL OF PEDIATRIC ORTHOPAEDICS

LA English

DT Article

DE adolescent idiopathic scoliosis; cardiopulmonary exercise test; early-onset scoliosis; exercise capacity; growing rod; growing rod graduate; pulmonary function test; pulmonary reserve; scoliosis

ID PROSTHETIC TITANIUM RIB; BREATHING RESERVE INDEX; CHEST-WALL DEFORMITIES; PULMONARY-FUNCTION; LACTATE THRESHOLD; FUSED RIBS; CHILDREN; FUSION; SURGERIES; INSERTION

AB Background: Growing-rod (GR) treatment is the current standard for progressive idiopathic early-onset scoliosis (I-EOS) in young children. Despite good radiographic outcomes, the impact of scoliosis treatment on pulmonary functions is not well-defined in this patient population. The aim of this study was to evaluate pulmonary functions and exercise tolerance in I-EOS patients graduated

from GR treatment and to compare them with age-matched, surgically treated adolescent idiopathic scoliosis (AIS) patients and healthy controls. Methods: Eight GR graduates with I-EOS with pulmonary function tests and complete radiographic results were compared with a group of 9 thoracic AIS patients at least 2 years out from posterior fusion. Both groups were also compared with a set of 10 healthy individuals. All subjects underwent cardiopulmonary exercise testing and spirometry to evaluate pulmonary function. Results: Age, sex, height, arm span, weight, residual deformity, and level of instrumentation in GR and AIS patients were similar. In the GR group, forced vital capacity % and forced expiratory volume in 1 second % values were reduced compared with the healthy controls and AIS group (P<0.001, <0.001 and 0.036, 0.046, respectively). Breathing reserve index at lactate threshold (BRILT) was higher in GR and AIS patients (P=0.001 and 0.002, respectively), and was similar between GR and AIS patients (P=0.916). Heart rate at lactate threshold was higher in GR and AIS groups compared with controls (P<0.001 and 0.001, respectively). Conclusions: AIS and GR patients demonstrated reduced pulmonary reserve and exercise tolerance compared with their peers with no spinal deformity. However, exercise tolerance of I-EOS patients treated with the GR method was similar to that of operated AIS patients. These results suggest a positive impact of GR treatment in children with I-EOS.

C1 [Celebioglu, Ebru; Oncel, Asli; Degirmenci, Ceren; Tekin, Fatih; Demir, Ahmet U.] Hacettepe Univ, Sch Med, Dept Chest Dis, Ankara, Turkey.

[Yataganbaba, Alper; Demirkiran, Gokhan; Yazici, Muharrem] Hacettepe Univ, Sch Med, Dept Orthopaed & Traumatol, TR-06100 Ankara, Turkey.

[Yalcin, Ebru] Hacettepe Univ, Sch Med, Dept Pediat Chest Dis, Ankara, Turkey.

[Bekmez, Senol] Cankaya Hosp, Dept Orthopaed & Traumatol, Ankara, Turkey.

C3 Hacettepe University; Hacettepe University; Hacettepe University;

Private Cankaya Hospital

RP Yazici, M (通讯作者)， Hacettepe Univ, Sch Med, Dept Orthopaed & Traumatol, TR-06100 Ankara, Turkey.

EM mimyazici@gmail.com

RI Bekmez, Senol/M-4212-2014; Alkan, Aslı/HHC-7785-2022

OI Alkan, Aslı/0000-0001-7708-1732; Tekin, Fatih/0000-0001-6688-1427

CR Aslan C., 2017, SPINE DEFORM, V5, P334, DOI [DOI 10.1016/j.jspd.2017.03.007, 10.1016/j.jspd.2017.03.007, DOI 10.1016/J.JSPD.2017.03.007]

Campbell RM, 2003, J BONE JOINT SURG AM, V85A, P399, DOI 10.2106/00004623-200303000-00001

DIMEGLIO A, 1992, J PEDIATR ORTHOP B, V1, P102, DOI 10.1097/01202412-199201020-00003

Doany ME, 2018, SPINE, V43, P148, DOI 10.1097/BRS.0000000000002274

Emans JB, 2005, SPINE, V30, pS58, DOI 10.1097/01.brs.0000175194.31986.2f

JACKSON RP, 1989, SPINE, V14, P1391, DOI 10.1097/00007632-198912000-00018

Jeans KA, 2017, SPINE DEFORM, V5, P416, DOI [DOI 10.1016/J.JSPD.2017.04.001, 10.1016/j.jspd.2017.04.001]

Jeans Kelly A, 2016, Spine Deform, V4, P413, DOI 10.1016/j.jspd.2016.06.002

Jiang Y, 2011, CHINESE MED J-PEKING, V124, P3858, DOI 10.3760/cma.j.issn.0366-6999.2011.23.005

Karol LA, 2008, J BONE JOINT SURG AM, V90A, P1272, DOI 10.2106/JBJS.G.00184

Karol LA, 2011, CLIN ORTHOP RELAT R, V469, P1323, DOI 10.1007/s11999-010-1622-z

LISBOA C, 1985, AM REV RESPIR DIS, V132, P48

Matsumoto H, 2014, J PEDIATR ORTHOPED, V34, P172, DOI 10.1097/BPO.0b013e3182a11d73

Mayer O, 2016, CURR PROB PEDIATR AD, V46, P72, DOI 10.1016/j.cppeds.2015.11.001

Mayer OH, 2009, J PEDIATR ORTHOPED, V29, P35, DOI 10.1097/BPO.0b013e3181929c8b

Medoff BD, 1998, CHEST, V113, P913, DOI 10.1378/chest.113.4.913

Motoyama EK, 2009, PAEDIATR RESPIR REV, V10, P12, DOI 10.1016/j.prrv.2008.10.004

Odent T, 2015, ORTHOP TRAUMATOL-SUR, V101, pS281, DOI 10.1016/j.otsr.2015.07.004

Plowman SA., 2017, EXERCISE PHYSL HLTH

Redding GJ, 2011, CLIN ORTHOP RELAT R, V469, P1330, DOI 10.1007/s11999-010-1621-0

Sexauer WP, 2003, CHEST, V124, P1469, DOI 10.1378/chest.124.4.1469

Sponseller PD, 2007, SPINE, V32, pS81, DOI 10.1097/BRS.0b013e3181453073

Sun ZJ, 2015, EUR SPINE J, V24, P1434, DOI 10.1007/s00586-014-3668-1

Tantisira KG, 2002, AM J RESP CRIT CARE, V165, P1629, DOI 10.1164/rccm.2105090

UPADHYAY SS, 1995, SPINE, V20, P2415, DOI 10.1097/00007632-199511001-00008

WEINSTEIN SL, 1981, J BONE JOINT SURG AM, V63, P702, DOI 10.2106/00004623-198163050-00003

Yaszay B, 2017, EUR SPINE J, V26, P1658, DOI 10.1007/s00586-016-4694-y

Yaszay B, 2009, J SPINAL DISORD TECH, V22, P278, DOI 10.1097/BSD.0b013e31816d2530

NR 28

TC 3

Z9 3

U1 0

U2 4

PU LIPPINCOTT WILLIAMS & WILKINS

PI PHILADELPHIA

PA TWO COMMERCE SQ, 2001 MARKET ST, PHILADELPHIA, PA 19103 USA

SN 0271-6798

EI 1539-2570

J9 J PEDIATR ORTHOPED

JI J. Pediatr. Orthop.

PD SEP

PY 2020

VL 40

IS 8

BP E734

EP E739

DI 10.1097/BPO.0000000000001567

PG 6

WC Orthopedics; Pediatrics

WE Science Citation Index Expanded (SCI-EXPANDED)

SC Orthopedics; Pediatrics

GA NP2QP

UT WOS:000570026100024

PM 32282619

DA 2023-08-10

ER

PT J

AU Dolan, LA

Donzelli, S

Zaina, F

Weinstein, SL

Negrini, S

AF Dolan, Lori A.

Donzelli, Sabrina

Zaina, Fabio

Weinstein, Stuart L.

Negrini, Stefano

TI Adolescent Idiopathic Scoliosis Bracing Success Is Influenced by Time in

Brace Effectiveness Analysis of BrAIST and ISICO Cohorts

SO SPINE

LA English

DT Article

DE adolescent idiopathic scoliosis; bracing; comparative effectiveness

study; non-operative treatment; orthotics

AB Study Design. Comparative effectiveness study

Objective. To evaluate factors leading to higher percentage of brace failures in a cohort of North American patients with adolescent idiopathic scoliosis relative to their peers in Italy.

Summary of Background Data. Studies of bracing in United States have shown worse outcomes than studies from European centers, possibly due to sample characteristics or treatment approaches.

Methods: Sample: Braced patients, aged 10 to 15, Risser <3, Cobb 20 degrees- to 40 degrees, observed to Cobb >= 40 degrees and/or >= Risser 4 selected from prospective databases. Comparators: Bracing per Bracing in Adolescent Idiopathic Scoliosis Trial (BrAIST) (TLSO) and Italian Scientific Spine Institute (ISICO) protocol (SPoRT braces with or without SEAS exercises). Baseline characteristics (sex, age, BMI, Risser, Cobb, curve type) and average hours of brace wear/day. Differences in programs (e.g., SEAS, type of brace, weaning protocol) were captured by a variable named "SITE." Outcome: Treatment failure (Cobb >= 40 before Risser 4). Statistics: Comparison of baseline characteristics, analyses of risk factors, treatment components, and outcomes within and between cohorts using logistic regression.

Results: A total of 157 BrAIST and 81 ISICO subjects were included. Cohorts were similar at baseline but differed significantly in terms of average hours of brace wear: 18.31 in the ISICO versus 11.76 in the BrAIST cohort. Twelve percent of the ISICO and 39% of the BrAIST cohort had failed treatment. Age, Risser, Cobb, and a thoracic apex predicted failure in both groups. SITE was related to failure (odds ratio [OR] = 0.19), indicating lower odds of failure with ISICO versus BrAIST approach. With both SITE and wear time in the model, SITE loose significance. In the final model, the adjusted odds of failure were higher in boys (OR = 3.34), and those with lowest BMI (OR = 9.83); the odds increased with the Cobb angle (OR = 1.23), and decreased with age (OR = 0.41) and hours of wear (OR = 0.86).

Conclusion: Treatment at the ISICO resulted in a lower failure rate, primarily explained by longer average hours of brace wear.

C1 [Dolan, Lori A.; Weinstein, Stuart L.] Univ Iowa, Dept Orthopaed & Rehabil, Iowa City, IA USA.

[Donzelli, Sabrina; Zaina, Fabio; Negrini, Stefano] Italian Sci Spine Inst ISICO, Via Bellarmino 13, Milan, Italy.

[Negrini, Stefano] Univ Brescia, Brescia, Italy.

[Negrini, Stefano] Fdn Don C Gnocchi, Milan, Italy.

C3 University of Iowa; University of Brescia; IRCCS Fondazione Don Carlo

Gnocchi Onlus

RP Donzelli, S (通讯作者)， Italian Sci Spine Inst ISICO, Via Bellarmino 13, Milan, Italy.

EM sabrina.donzelli@isico.it

RI Donzelli, Sabrina/IQU-0779-2023; Negrini, Stefano/B-6667-2013; Zaina,

Fabio/H-3261-2013

OI Negrini, Stefano/0000-0002-1878-2747; Zaina, Fabio/0000-0002-1256-5362;

Weinstein, Stuart/0000-0002-3676-6687

FU National Institute of Arthritis and Musculoskeletal and Skin Diseases [R21AR049587, R01AR052113]; Children's Miracle Network; Canadian Institutes of Health Research [FRN81050]; Shriners Hospitals for Children; Children's Mercy Kansas City; University of Rochester

FX BrAIST was funded by the National Institute of Arthritis and Musculoskeletal and Skin Diseases (R21AR049587 and R01AR052113), the Children's Miracle Network, the Canadian Institutes of Health Research (FRN81050), the Shriners Hospitals for Children, the University of Rochester, and the Children's Mercy Kansas City.

CR AKAIKE H, 1974, IEEE T AUTOMAT CONTR, VAC19, P716, DOI 10.1109/TAC.1974.1100705

[Anonymous], 2019, **NON-TRADITIONAL**

Babatunde F, 2017, BMC HEALTH SERV RES, V17, DOI 10.1186/s12913-017-2311-3

Danielsson AJ, 2007, SPINE, V32, P2198, DOI 10.1097/BRS.0b013e31814b851f

De Giorgi S, 2013, EUR SPINE J, V22, pS815, DOI 10.1007/s00586-013-3020-1

Donzelli S, 2012, SCOLIOSIS SPINAL DIS, V7, DOI 10.1186/1748-7161-7-12

Gammon SR, 2010, J PEDIATR ORTHOPED, V30, P531, DOI 10.1097/BPO.0b013e3181e4f761

Goodbody CM, 2016, J CHILD ORTHOP, V10, P395, DOI 10.1007/s11832-016-0763-3

Gutman G, 2016, SPINE J, V16, P626, DOI 10.1016/j.spinee.2016.01.020

Hosmer DW, 2000, TEAM LOGISTIC REGRES

Karol LA, 2016, J BONE JOINT SURG AM, V98, P1253, DOI 10.2106/JBJS.15.01313

Karol LA, 2016, J BONE JOINT SURG AM, V98, P9, DOI 10.2106/JBJS.O.00359

Korovessis P., 2018, SPINE DEFORM, V6, P514, DOI [10.1016/j.jspd.2018.01.006, DOI 10.1016/J.JSPD.2018.01.006]

Miller DJ, 2012, SPINE, V37, P717, DOI 10.1097/BRS.0b013e31822f4306

Minsk MK, 2017, SCOLIOSIS SPINAL DIS, V12, DOI 10.1186/s13013-017-0117-z

Morton A, 2008, J PEDIATR ORTHOPED, V28, P336, DOI 10.1097/BPO.0b013e318168d154

Negrini S, 2014, BMC MUSCULOSKEL DIS, V15, DOI 10.1186/1471-2474-15-263

Negrini S, 2011, SCOLIOSIS SPINAL DIS, V6, DOI 10.1186/1748-7161-6-8

Pasquini G, 2016, EUR J PHYS REHAB MED, V52, P618

Richards BS, 2005, SPINE, V30, P2068, DOI 10.1097/01.brs.0000178819.90239.d0

Rivett L, 2009, BMC MUSCULOSKEL DIS, V10, DOI 10.1186/1471-2474-10-5

Romano M, 2015, SCOLIOSIS SPINAL DIS, V10, DOI 10.1186/s13013-014-0027-2

Sanders JO, 2014, J BONE JOINT SURG AM, V96A, P649, DOI 10.2106/JBJS.M.00290

Tavernaro M, 2012, SCOLIOSIS SPINAL DIS, V7, DOI 10.1186/1748-7161-7-17

Uno H, 2014, NEW ENGL J MED, V370, P680, DOI 10.1056/NEJMc1314229

WEINSTEIN SL, 1983, J BONE JOINT SURG AM, V65, P447, DOI 10.2106/00004623-198365040-00004

Weinstein SL, 2013, SPINE, V38, P1832, DOI 10.1097/01.brs.0000435048.23726.3e

Ylikoski M, 2005, J PEDIATR ORTHOP B, V14, P320, DOI 10.1097/01202412-200509000-00002

Zaborowska-Sapeta K, 2011, SCOLIOSIS SPINAL DIS, V6, DOI 10.1186/1748-7161-6-2

Zolnierek KBH, 2009, MED CARE, V47, P826, DOI 10.1097/MLR.0b013e31819a5acc

NR 30

TC 17

Z9 18

U1 0

U2 6

PU LIPPINCOTT WILLIAMS & WILKINS

PI PHILADELPHIA

PA TWO COMMERCE SQ, 2001 MARKET ST, PHILADELPHIA, PA 19103 USA

SN 0362-2436

EI 1528-1159

J9 SPINE

JI SPINE

PD SEP 1

PY 2020

VL 45

IS 17

BP 1193

EP 1199

DI 10.1097/BRS.0000000000003506

PG 7

WC Clinical Neurology; Orthopedics

WE Science Citation Index Expanded (SCI-EXPANDED)

SC Neurosciences & Neurology; Orthopedics

GA QJ2HJ

UT WOS:000619511000016

PM 32205704

DA 2023-08-10

ER

PT J

AU Liu, DL

Yang, YL

Yu, XX

Yang, JF

Xuan, XL

Yang, JL

Huang, ZF

AF Liu, Delong

Yang, Yunlin

Yu, Xuexiang

Yang, Jingfan

Xuan, Xiaoling

Yang, Junlin

Huang, Zifang

TI Effects of Specific Exercise Therapy on Adolescent Patients With

Idiopathic Scoliosis A Prospective Controlled Cohort Study

SO SPINE

LA English

DT Article

DE age; curve progression; efficacy; idiopathic scoliosis; intervention

window; prospective controlled cohort study; Risser sign; specific

exercise

AB Study Design. A prospective controlled cohort study.

Objective. The aim of this study was to explore the interventional effect of exercise therapy on idiopathic scoliosis (IS) and identify an optimal intervention window.

Summary of Background Data. Early conservative treatment is helpful for IS. In addition to bracing, current evidence suggests that exercise can play an important role.

Methods. We included 99 patients with IS who were treated at the Guangdong Xinmiao Scoliosis Center from August 2013 to September 2017. The inclusion criteria were: new IS diagnosis, Cobb angle 10 degrees to 25 degrees, Risser 0 to 3 grade, only treated with the Xinmiao treatment system (XTS; > 3 days/week, >1 h/day), and follow-up >1 year. Patients were divided into three age groups: A, <10 years (n = 29); B, 10 to 12 years (n = 24); and C, 13 to 15 years (n = 46). The percentages of curve improvement (Cobb angle decrease >= 5 degrees), stability (Cobb angle change +/- 5 degrees), and progression (Cobb angle increase >= 5 degrees) were compared.

Results. The groups showed significant differences for major curve correction, Risser sign, first referral, and final follow-up of the main curve (all P< 0.05). The major curve in group A decreased significantly by 6.8 degrees (44% correction), compared to 3.1 degrees (18% correction) and 1.5 degrees (9% correction) in groups B and C, respectively. In group A, 69.0% (20/29) had curve improvement, 27.6% (8/29) stabilized and 3.4% (1/29) progressed. In group B, 45.8% (11/24) improved, 50% (12/24) stabilized, and 4.2% (1/24) progressed. In group C, 26.1% (12/46) improved, 63.0% (29/46) stabilized, and 10.9% (5/46) progressed. There was also a significant difference in final Risser grade among the groups (P<0.05).

Conclusion. For IS patients with Cobb angles between 10 degrees and 25 degrees, our exercise protocol can effectively control or improve curve progression. Younger patients with a lower Risser grade are most likely to respond.

C1 [Liu, Delong; Huang, Zifang] Sun Yat Sen Univ, Dept Spine Surg, Affiliated Hosp 1, Guangzhou 510120, Guangdong, china.

[Yang, Yunlin] Chengdu Univ Technol, Phys Educ Inst, Chengdu, china.

[Yu, Xuexiang] Guangzhou Sport Univ, Dept Sports & Arts, Guangzhou, Guangdong, china.

[Yang, Jingfan; Yang, Junlin] Shanghai Jiao Tong Univ, Spine Ctr, Xinhua Hosp Affiliated, Sch Med, Shanghai 200092, china.

[Xuan, Xiaoling] Xinmiao Scoliosis Prevent Ctr Guangdong Prov, Guangzhou, Guangdong, china.

C3 Sun Yat Sen University; Chengdu University of Technology; Guangzhou

Sport University; Shanghai Jiao Tong University

RP Huang, ZF (通讯作者)， Sun Yat Sen Univ, Dept Spine Surg, Affiliated Hosp 1, Guangzhou 510120, Guangdong, china.; Yang, JL (通讯作者)， Shanghai Jiao Tong Univ, Spine Ctr, Xinhua Hosp Affiliated, Sch Med, Shanghai 200092, china.

EM yjunlin@126.com; laughten@126.com

OI Junlin, Yang/0000-0001-9809-1219

FU National Key Research and Development Program [2018YFC0116500]; National

Natural Science Foundation Fund for Overseas and HongKong and Macao

Scholars Joint Research Project [81828007]; Key Project of

Transformational Medicine Cross-Research Fund of Shanghai Jiaotong

University [ZH2018ZDB04]; Guangdong Science and Technology Planning

Project [2014B02021202]; National Natural Science Foundation of China

[31771330]; Sun Yat-Sen University Clinical Research 5010 Program
[2012003]
FX National Key Research and Development Program(2018YFC0116500), the
National Natural Science Foundation Fund for Overseas and HongKong and
Macao Scholars Joint Research Project (81828007), the Key Project of
Transformational Medicine Cross-Research Fund of Shanghai Jiaotong
University (ZH2018ZDB04), Guangdong Science and Technology Planning
Project (2014B02021202), National Natural Science Foundation of China
(31771330), Sun Yat-Sen University Clinical Research 5010 Program
(NO:2012003) funds were received in support of this work.
CR Berdishevsky H, 2016, SCOLIOSIS SPINAL DIS, V11, DOI 10.1186/s13013-016-0076-9
Bettany-Saltikov J, 2014, EUR J PHYS REHAB MED, V50, P111
Cheung JPY, 2018, CLIN ORTHOP RELAT R, V476, P429, DOI 10.1007/s11999.0000000000000027
Dunn J, 2018, JAMA-J AM MED ASSOC, V319, P173, DOI 10.1001/jama.2017.11669
GOLDBERG MS, 1994, SPINE, V19, P1551, DOI 10.1097/00007632-199407001-00003
Hawes Martha C, 2003, Pediatr Rehabil, V6, P171
Koumbourlis AC, 2006, PAEDIATR RESPIR REV, V7, P152, DOI 10.1016/j.prrv.2006.04.009
Kwan KYH, 2017, SCOLIOSIS SPINAL DIS, V12, DOI 10.1186/s13013-017-0139-6
LONSTEIN JE, 1984, J BONE JOINT SURG AM, V66A, P1061, DOI 10.2106/00004623-198466070-00013
Lonstein JE, 2006, CLIN ORTHOP RELAT R, P248, DOI 10.1097/01.blo.0000198725.54891.73
Monticone M, 2014, EUR SPINE J, V23, P1204, DOI 10.1007/s00586-014-3241-y
Moramarco Marc, 2017, Open Orthop J, V11, P1490, DOI 10.2174/1874325001711011490
Mordecai SC, 2012, EUR SPINE J, V21, P382, DOI 10.1007/s00586-011-2063-4
Negrini A, 2008, SCOLIOSIS SPINAL DIS, V3, DOI 10.1186/1748-7161-3-20
Negrini S, 2008, DISABIL REHABIL, V30, P772, DOI 10.1080/09638280801889568

Negrini S, 2008, J REHABIL MED, V40, P451, DOI 10.2340/16501977-0195

Negrini S, 2018, SCOLIOSIS SPINAL DIS, V13, DOI 10.1186/s13013-017-0145-8

Otman Saadet, 2005, Neurosciences (Riyadh), V10, P277

Park JH, 2018, EUR J PHYS REHAB MED, V54, P440, DOI 10.23736/S1973-9087.17.04461-6

Romano M, 2013, SPINE, V38, pE883, DOI 10.1097/BRS.0b013e31829459f8

Sarwark JF, 2018, JAMA-J AM MED ASSOC, V319, P127, DOI 10.1001/jama.2017.20190

Schreiber S, 2016, PLOS ONE, V11, DOI 10.1371/journal.pone.0168746

Stokes IAF, 2006, SCOLIOSIS SPINAL DIS, V1, DOI 10.1186/1748-7161-1-16

Tones M, 2006, SPINE, V31, P3027, DOI 10.1097/01.brs.0000249555.87601.fc

Weinstein SL, 2008, LANCET, V371, P1527, DOI 10.1016/S0140-6736(08)60658-3

Weiss HR, 2008, EUR J PHYS REHAB MED, V44, P177

Weiss Hans-Rudolf, 2003, Pediatr Rehabil, V6, P23, DOI 10.1080/1363849031000095288

Weiss HR, 2009, STUD HEALTH TECHNOL, V135, P164

Zapata KA, 2019, PEDIATR PHYS THER, V31, P280, DOI 10.1097/PEP.0000000000000621

Zheng YP, 2016, SCOLIOSIS SPINAL DIS, V11, DOI 10.1186/s13013-016-0074-y

NR 30

TC 18

Z9 20

U1 1

U2 11

PU LIPPINCOTT WILLIAMS & WILKINS

PI PHILADELPHIA

PA TWO COMMERCE SQ, 2001 MARKET ST, PHILADELPHIA, PA 19103 USA

SN 0362-2436

EI 1528-1159

J9 SPINE

JI SPINE

PD AUG 1

PY 2020

VL 45

IS 15

BP 1039

EP 1046

DI 10.1097/BRS.0000000000003451

PG 8

WC Clinical Neurology; Orthopedics

WE Science Citation Index Expanded (SCI-EXPANDED)

SC Neurosciences & Neurology; Orthopedics

GA QJ2FR

UT WOS:000619506100022

PM 32675606

OA Green Published

DA 2023-08-10

ER

PT J

AU Vollner, F

Dingeldey, E

Schmitz, S

Grifka, J

Matussek, J

AF Voellner, Florian

Dingeldey, Esther

Schmitz, Stephanie

Grifka, Joachim

Matussek, Jan

TI Conservative and surgical treatment of idiopathic scoliosis

SO ORTHOPADE

LA German

DT Article

DE Deformity; Braces; Infantile scoliosis; Juvenile scoliosis; Adolescent

scoliosis

ID CURVE PROGRESSION

AB Idiopathic scoliosis is the largest group of all forms of scoliosis in the growth phase accounting for 80-90%. A distinction is made between idiopathic infantile (0-3 years), juvenile (4-10 years) and adolescent scoliosis (>10 years), depending on the age when scoliosis appears. The treatment depends on the skeletal age, the Cobb angle and the progression behavior of scoliosis. Mild cases are treated conservatively using physiotherapy, exercises and bracing but in advanced stages scoliosis should be surgically treated. With existing growth potential various techniques, such as traditional growing rods, magnetically controlled growth rods and vertebral body tethering are available. After the end of the growth phase a fusion should be recommended for scoliosis >50 degrees.

C1 [Voellner, Florian; Dingeldey, Esther; Schmitz, Stephanie; Grifka, Joachim] Univ Regensburg, Asklepios Klinikum Bad Abbach, Orthopad Klin, Kaiser Karl V Allee 3, D-93077 Bad Abbach, Germany.

[Matussek, Jan] Helios Klinikum Emil von Behring, Klin Kinderorthopad & Kindertraumatol, Berlin, Germany.

C3 University of Regensburg; Helios Kliniken

RP Vollner, F (通讯作者)， Univ Regensburg, Asklepios Klinikum Bad Abbach, Orthopad Klin, Kaiser Karl V Allee 3, D-93077 Bad Abbach, Germany.

EM Florian.voellner@klinik.uni-regensburg.de

CR [Anonymous], 1950, RADIOGRAPHIC ATLAS S

Aulisa AG, 2017, SCOLIOSIS SPINAL DIS, V12, DOI 10.1186/s13013-017-0142-y

BUNNELL WP, 1988, CLIN ORTHOP RELAT R, P20

Charles YP, 2007, J BONE JOINT SURG AM, V89A, P2737, DOI 10.2106/JBJS.G.00124

Danielsson AJ, 2001, SPINE, V26, P516

Drerup B, 2014, SCOLIOSIS SPINAL DIS, V9, DOI 10.1186/s13013-014-0022-7

KING HA, 1983, J BONE JOINT SURG AM, V65, P1302, DOI 10.2106/00004623-198365090-00012

Marion TE, 2018, 50 LANDMARK PAPERS, P173, DOI DOI 10.1201/9781315154053-33

Mehta M H, 1973, J Bone Joint Surg Br, V55, P513

Minkara A, 2020, J PEDIATR ORTHOPED, V40, P60, DOI 10.1097/BPO.0000000000001135

NASH CL, 1969, J BONE JOINT SURG AM, VA 51, P223, DOI 10.2106/00004623-196951020-00002

Negrini S, 2018, SCOLIOSIS SPINAL DIS, V13, DOI 10.1186/s13013-017-0145-8

Pellios S, 2016, SCOLIOSIS SPINAL DIS, V11, DOI 10.1186/s13013-016-0065-z

Redding Gregory J, 2014, Spine Deform, V2, P425, DOI 10.1016/j.jspd.2014.04.010

Robinson CM, 1996, J BONE JOINT SURG AM, V78A, P1140, DOI 10.2106/00004623-199608000-00003

Sanders JO, 2008, J BONE JOINT SURG AM, V90A, P540, DOI 10.2106/JBJS.G.00004

SAUVEGRAIN J, 1962, Ann Radiol (Paris), V5, P542

WEINSTEIN SL, 1983, J BONE JOINT SURG AM, V65, P447, DOI 10.2106/00004623-198365040-00004

Weinstein SL, 2008, LANCET, V371, P1527, DOI 10.1016/S0140-6736(08)60658-3

Weinstein SL, 2013, NEW ENGL J MED, V369, P1512, DOI 10.1056/NEJMoa1307337

NR 20

TC 2

Z9 3

U1 4

U2 20

PU SPRINGER

PI NEW YORK

PA ONE NEW YORK PLAZA, SUITE 4600, NEW YORK, NY, UNITED STATES

SN 0085-4530

EI 1433-0431

J9 ORTHOPADE

JI Orthopade

PD JUL

PY 2020

VL 49

IS 7

SI SI

BP 635

EP 646

DI 10.1007/s00132-020-03928-2

EA JUN 2020

PG 12

WC Orthopedics

WE Science Citation Index Expanded (SCI-EXPANDED)

SC Orthopedics

GA MF5HI

UT WOS:000540398200001

PM 32542427

DA 2023-08-10

ER

PT J

AU Nisser, J

Smolenski, U

Sliwinski, GE

Schumann, P

Heinke, A

Malberg, H

Werner, M

Elsner, S

Drossel, WG

Sliwinski, Z

Derlien, S

AF Nisser, Jenny

Smolenski, Ulrich

Sliwinski, Grzegorz Eugeniusz

Schumann, Paula

Heinke, Andreas

Malberg, Hagen

Werner, Michael

Elsner, Sophia

Drossel, Welf-Guntram

Sliwinski, Zbigniew

Derlien, Steffen

TI The FED-Method (Fixation, Elongation, Derotation) - Machine-supported

Treatment Approach to Patients with Idiopathic Scoliosis - Systematic

Review

SO ZEITSCHRIFT FUR ORTHOPADIE UND UNFALLCHIRURGIE

LA German

DT Review

DE adolescent idiopathic scoliosis (AIS); scoliosis specific physiotherapy;

conservative treatment; FED method

AB Background The FED method (Fixation, Elongation, Derotation) is a treatment method approach to Patients with scoliosis. The FED method is especially established in Spain and Poland, whereby in Germany it is less well- known. Nevertheless the FED method is within the scope of a research project (Project Number: 19200 BR/3). The purpose of the paper is to characterize the FED method and to highlight the specificities in contrast to the Schroth method, which is international established and especially in Germany.

Methods This systematic literature research was conducted in Nov 2017-Jan 2018. Therefore common medical and physiotherapeutic databases were used. Furthermore there was a hand search in selected scientific journals. Only a small number of relevant references were identified. That is why the respective authors were asked to provide the full-texts of their papers and to recommend further references.

Results A total of 378 references were identified. After removing duplicates and the content-related selection, 19 references were deemed to be relevant. Based on the analysis of this relevant literature, the FED method was comprehensively characterized. First of all the general structure of the FED method and the scientific evidence for its effectiveness was described. And as a result of the literature research, the operating principles of the FED method were pointed out. Then these operating principles were discussed in comparison with the Schroth method. The Schroth method based on sensomotoric and kinesthetic principles and the correction of the pathologic posture was performed by selective muscle activation and breathing-pattern. Thus, the posture correction will be performed by the patients (auto correction). Compared to the Schroth method, the FED method implements the posture correction by the FED-device. This correction is influenced by mechanical forces with a comparatively high strength and intensity. The repetitive mechanical correction stimulates the sensomotoric system. And due to trophic/biochemical adaptations, the physiological bone growth will be stimulated.

Conclusion In total the authors want to clarify, that both treatment methods (Schroth method, FED method) supposed to be applied in consideration of the preconditions of the patients and the pursue of the different treatment goals. Thus, the implementation of treatment methods should be used according to the individual treatment demand and on different stages in the treatment process.

C1 [Nisser, Jenny; Smolenski, Ulrich; Derlien, Steffen] Univ Klinikum Jena, Inst Physiotherapie, Klinikum 1, D-07747 Jena, Germany.

[Sliwinski, Grzegorz Eugeniusz; Schumann, Paula; Heinke, Andreas; Malberg, Hagen] Tech Univ Dresden, Inst Biomed Tech, Dresden, Germany.

[Werner, Michael; Elsner, Sophia; Drossel, Welf-Guntram] Fraunhofer Inst Werkzeugmaschinen & Umformtech, Abt Med Tech, Dresden, Germany.

[Sliwinski, Zbigniew] Uniwersytet Jana Kochanowskiego Kielcach, Fac Med & Hlth Sci, Kielce, Poland.

C3 Friedrich Schiller University of Jena; Technische Universitat Dresden;

Fraunhofer Gesellschaft; Jan Kochanowski University

RP Nisser, J (通讯作者)， Univ Klinikum Jena, Inst Physiotherapie, Klinikum 1, D-07747 Jena, Germany.

EM jenny.nisser@med.uni-jena.de

RI Sliwiński, Zbigniew/O-2678-2014

OI Werner, Michael/0000-0002-7864-9446

CR [Anonymous], 2007, BEWEGUNGSLEHRE SPORT

Barrios C, 2002, ST HEAL T, V88, P290

Berdishevsky H, 2016, SCOLIOSIS SPINAL DIS, V11, DOI 10.1186/s13013-016-0076-9

Biedermann H, 2008, MAN MED, V46, P17, DOI 10.1007/s00337-008-0572-y

Caciulan E., 2011, REV ROMANA KINETOTER, V17, P10

Fusco C., 2011, Physiotherapy Theory and Practice, V27, P80, DOI 10.3109/09593985.2010.533342

Grymek S, 2012, KEY ENG MATER, V490, P8, DOI 10.4028/www.scientific.net/KEM.490.8

Karwat W.., 2015, THESIS U MEDYCZNY WR, P52

Kuru T, 2016, CLIN REHABIL, V30, P181, DOI 10.1177/0269215515575745

Lapuente JP, 2002, ST HEAL T, V88, P258

Montes Manas A.., 2014, EFECTIVIDAD METODO F, P47

MotowCzyz M., 2012, SPOLECZENSTWO EDUKAC, V1, P375

Negrini S, 2012, SCOLIOSIS SPINAL DIS, V7, DOI 10.1186/1748-7161-7-3

Nisser J, 2018, PHYS MED REHAB KUROR, V28, P88, DOI 10.1055/s-0043-124472

Otman S, 2005, SAUDI MED J, V26, P1429

PERDRIOLLE R, 1993, SPINE, V18, P343, DOI 10.1097/00007632-199303000-00007

Romano M, 2013, SPINE, V38, pE883, DOI 10.1097/BRS.0b013e31829459f8

Ruchholtz S, 2010, ORTHOPADIE UNFALLCHI

Sastre Fernandez S, 2006, METODO TRATAMIENTO E

Sastre Fernandez S., 1994, EUROPEAN SPINAL RESO, V3, P7

Sastre Fernandez S, 2008, METODA LECZENIA SKOL

Sastre Fernandez S, 2009, TREATMENT SCOLIOSIS, P1

Sastre Fernandez S., 1989, FISIOTERAPIA, V39, P7

Sastre S, 2002, ST HEAL T, V88, P270

Sastre S, 1999, ST HEAL T, V59, P171

Sastre S, 1997, ST HEAL T, V37, P393

Sliwinski G, 2014, SCOLIOSIS SPINAL DIS, V9, pO55, DOI [10.1186/1748-7161-9-S1-O55, DOI 10.1186/1748-7161-9-S1-O55]

Sliwinski Z, 2014, SCOLIOSIS SPINAL DIS, V9, pP13, DOI [10.1186/1748-7161-9-S1-P13, DOI 10.1186/1748-7161-9-S1-P13]

Sliwinski Z., 2014, SCOLIOSIS SPINAL DIS, V9, pP15, DOI [10.1186/1748-7161-9-S1-P15, DOI 10.1186/1748-7161-9-S1-P15]

Sliwinski Z, 2014, SCOLIOSIS SPINAL DIS, V9, P14

Stokes IAF, 1996, SPINE, V21, P1162, DOI 10.1097/00007632-199605150-00007

Suchanowski A, 2013, BALT J HEALTH PHYS A, V5, P132, DOI 10.2478/bjha-2013-0013

Tresserra J, 1989, REV ORTOP TRAUM, V33, P117

Trzcinska S., 2017, FIZJOTER POL, V17, P6

Voelcker-Rehage C., 2005, DEUT Z SPORTMED, V56, P358

Wei H-R., 2003, Orthopade, V32, P146, DOI 10.1007/s00132-002-0430-x

Weiss Hans-Rudolf, 2003, Pediatr Rehabil, V6, P183

Weiss HR, 2011, SCOLIOSIS SPINAL DIS, V6, DOI 10.1186/1748-7161-6-17

Weiss HR, 2002, ST HEAL T, V88, P304

WEISS HR, 1995, Z ORTHOP GRENZGEB, V133, P114, DOI 10.1055/s-2008-1039421

Wulker N., 2015, TASCHENLEHRBUCH ORTH

NR 41

TC 4

Z9 4

U1 2

U2 6

PU THIEME MEDICAL PUBL INC

PI NEW YORK

PA 333 SEVENTH AVE, NEW YORK, NY 10001 USA

SN 1864-6697

EI 1864-6743

J9 Z ORTHOP UNFALLCHIR

JI Z. Orthop. Unfallchir.

PD JUN

PY 2020

VL 158

IS 3

BP 318

EP 332

DI 10.1055/a-0881-3430

PG 15

WC Orthopedics

WE Science Citation Index Expanded (SCI-EXPANDED)

SC Orthopedics

GA QC3SU

UT WOS:000614754800014

PM 31404938

DA 2023-08-10

ER

PT J

AU Cheung, JPY

Cheung, PWH

Shigematsu, H

Takahashi, S

Kwan, MK

Chan, CYW

Chiu, CK

Sakai, D

AF Cheung, Jason Pui Yin

Cheung, Prudence Wing Hang

Shigematsu, Hideki

Takahashi, Shinji

Kwan, Mun Keong

Chan, Chris Yin Wei

Chiu, Chee Kidd

Sakai, Daisuke

CA APSS Scoliosis Focus Grp

TI Controversies with nonoperative management for adolescent idiopathic scoliosis: Study from the APSS Scoliosis Focus Group

SO JOURNAL OF ORTHOPAEDIC SURGERY

LA English

DT Article

DE adolescent idiopathic scoliosis; AIS; bracing; nonoperative

ID ASSESSING SKELETAL MATURITY; PREDICTS CURVE PROGRESSION; LARGE-SCALE

MULTICENTER; ULNA CLASSIFICATION; DISTAL RADIUS; ASIA-PACIFIC;

DEGENERATIVE SPONDYLOLISTHESIS; TROPISM; SYSTEM; BRACE

AB Purpose: To determine consensus among Asia-Pacific surgeons regarding nonoperative management for adolescent idiopathic scoliosis (AIS). Methods: An online REDCap questionnaire was circulated to surgeons in the Asia-Pacific region during the period of July 2019 to September 2019 to inquire about various components of nonoperative treatment for AIS. Aspects under study included access to screening, when MRIs were obtained, quality-of-life assessments used, role of scoliosis-specific exercises, bracing criteria, type of brace used, maturity parameters used, brace wear regimen, follow-up criteria, and how braces were weaned. Comparisons were made between middle-high income and low-income countries, and experience with nonoperative treatment. Results: A total of 103 responses were collected. About half (52.4%) of the responders had scoliosis screening programs and were particularly situated in middle-high income countries. Up to 34% obtained MRIs for all cases, while most would obtain MRIs for neurological problems. The brace criteria were highly variable and was usually based on menarche status (74.7%), age (59%), and Risser staging (92.8%). Up to 52.4% of surgeons elected to brace patients with large curves before offering surgery. Only 28% of responders utilized CAD-CAM techniques for brace fabrication and most (76.8%) still utilized negative molds. There were no standardized criteria for brace weaning. Conclusion: There are highly variable practices related to nonoperative treatment for AIS and may be related to availability of resources in certain countries. Relative consensus was achieved for when MRI should be obtained and an acceptable brace compliance should be more than 16 hours a day.

C1 [Cheung, Jason Pui Yin; Cheung, Prudence Wing Hang] Univ Hong Kong, Dept Orthopaed & Traumatol, Pokfulam, 5th Floor,Professorial Block,102 Pokfulam Rd, Hong Kong, china.

[Shigematsu, Hideki] Nara Med Univ, Dept Orthopaed Surg, Nara, Japan.

[Takahashi, Shinji] Osaka City Univ, Dept Orthopaed Surg, Osaka, Japan.

[Kwan, Mun Keong; Chan, Chris Yin Wei; Chiu, Chee Kidd] Univ Malaya, Dept Orthopaed Surg, Kuala Lumpur, Malaysia.

[Sakai, Daisuke] Tokai Univ, Sch Med, Dept Orthoped Surg, Hiratsuka, Kanagawa, Japan.

C3 University of Hong Kong; Nara Medical University; Osaka Metropolitan

University; Universiti Malaya; Tokai University

RP Cheung, JPY (通讯作者), Univ Hong Kong, Dept Orthopaed & Traumatol, Pokfulam, 5th Floor,Professorial Block,102 Pokfulam Rd, Hong Kong, china.

EM cheungjp@hku.hk

RI Chan, Chris Yin Wei/H-1377-2014; Takahashi, Shinji/ABC-5330-2020;

Cheung, Jason Pui Yin/J-2214-2012; Chiu, Chee Kidd/D-2760-2014; Cheung,

Jason Pui Yin/AAJ-2016-2020; Kwan, Mun Keong/C-6815-2011; Sakai,

Daisuke/X-9164-2019

OI Chan, Chris Yin Wei/0000-0001-7245-0295; Takahashi,

Shinji/0000-0002-6906-5406; Chiu, Chee Kidd/0000-0002-4198-1541; Cheung,

Jason Pui Yin/0000-0002-7052-0875; Kwan, Mun Keong/0000-0002-9512-3155;

Sakai, Daisuke/0000-0003-4189-9270; Cheung, Prudence/0000-0002-3213-7373

CR Aulisa AG, 2017, SCOLIOSIS SPINAL DIS, V12, DOI 10.1186/s13013-017-0142-y

Canavese F, 2011, INDIAN J ORTHOP, V45, P7, DOI 10.4103/0019-5413.73655

CHENG JC, 2015, NAT REV DIS PRIMERS, V1, DOI DOI 10.1038/NRDP.2015.30

Cheung JPY, 2020, BONE JOINT J, V102B, P254, DOI 10.1302/0301-620X.102B2.BJJ-2019-0916.R1

Cheung JPY, 2019, BONE JOINT J, V101B, P1370, DOI 10.1302/0301-620X.101B11.BJJ-2019-0515.R1

Cheung JPY, 2016, BONE JOINT J, V98B, P1689, DOI 10.1302/0301-620X.98B12.BJJ-2016-0158.R1

Cheung JPY, 2020, CLIN ORTHOP RELAT R, V478, P334, DOI 10.1097/CORR.0000000000000989

Cheung JPY, 2019, CLIN ORTHOP RELAT R, V477, P2145, DOI 10.1097/CORR.0000000000000781

Cheung JPY, 2018, SPINE, V43, P971, DOI 10.1097/BRS.0000000000002503

Cheung JPY, 2018, ASIAN SPINE J, V12, P202, DOI 10.4184/asj.2018.12.2.202

Cheung JPY, 2018, CLIN ORTHOP RELAT R, V476, P429, DOI 10.1007/s11999.0000000000000027

Cheung JPY, 2016, GLOB SPINE J, V6, P164, DOI 10.1055/s-0035-1557142

Cheung JPY, 2015, J PEDIATR ORTHOP B, V24, P546, DOI 10.1097/BPB.0000000000000214

Cheung PWH, 2019, SPINE, V44, pE596, DOI 10.1097/BRS.0000000000002918

Cheung PWH, 2018, EUR SPINE J, V27, P278, DOI 10.1007/s00586-017-5330-1

Cheung PWH, 2016, SCOLIOSIS SPINAL DIS, V11, DOI 10.1186/s13013-016-0083-x

Cobetto Nikita, 2014, Spine Deform, V2, P276, DOI 10.1016/j.jspd.2014.03.005

Do T, 2001, J BONE JOINT SURG AM, V83A, P577, DOI 10.2106/00004623-200104000-00014

Faloon M, 2018, CLIN ORTHOP RELAT R, V476, P1506, DOI 10.1007/s11999.0000000000000196

Lusini M, 2014, SPINE J, V14, P1951, DOI 10.1016/j.spinee.2013.11.040

Nault ML, 2010, J BONE JOINT SURG AM, V92A, P1073, DOI 10.2106/JBJS.H.01759

Negrini A, 2016, SCOLIOSIS SPINAL DIS, V11, DOI 10.1186/s13013-016-0100-0

Ohrt-Nissen S, 2019, J ORTHOP SURG-HONG K, V27, DOI 10.1177/2309499019860017

Ohrt-Nissen S, 2016, SPINE, V41, P1724, DOI 10.1097/BRS.0000000000001634

Ohrt-Nissen S, 2016, SPINE, V41, P798, DOI 10.1097/BRS.0000000000001519

Roye BD, 2020, SPINE DEFORM, V8, P597, DOI 10.1007/s43390-020-00060-1

Samartzis D, 2016, GLOB SPINE J, V6, P414, DOI 10.1055/s-0035-1564417

Samartzis D, 2016, SCOLIOSIS SPINAL DIS, V11, DOI 10.1186/s13013-016-0062-2

Sanders JO, 2007, J BONE JOINT SURG AM, V89A, P64, DOI 10.2106/JBJS.F.00067

Shi Benlong, 2016, Spine Deform, V4, P200, DOI 10.1016/j.jspd.2015.12.002

Weinstein SL, 2013, NEW ENGL J MED, V369, P1512, DOI 10.1056/NEJMoa1307337

Williams R, 2016, GLOB SPINE J, V6, P35, DOI 10.1055/s-0035-1555655

Wong AYL, 2019, CLIN ORTHOP RELAT R, V477, P676, DOI 10.1097/CORR.0000000000000569

Wong CKH, 2019, SPINE, V44, P1507, DOI 10.1097/BRS.0000000000003116

Wong CKH, 2019, EUR J HEALTH ECON, V20, P647, DOI 10.1007/s10198-018-1026-7

Wong CKM, 2017, PLOS ONE, V12, DOI 10.1371/journal.pone.0177266

Wong M. S., 2011, Physiotherapy Theory and Practice, V27, P74, DOI 10.3109/09593980903269741

NR 37

TC 2

Z9 2

U1 0

U2 5

PU SAGE PUBLICATIONS LTD

PI LONDON

PA 1 OLIVERS YARD, 55 CITY ROAD, LONDON EC1Y 1SP, united kingdom

SN 1022-5536

EI 2309-4990

J9 J ORTHOP SURG-HONG K

JI J. Orthop. Surg.

PD MAY

PY 2020

VL 28

IS 2

AR 2309499020930291

DI 10.1177/2309499020930291

PG 7

WC Orthopedics; Surgery

WE Science Citation Index Expanded (SCI-EXPANDED)

SC Orthopedics; Surgery

GA MB0ZY

UT WOS:000542338300001

PM 32529908

OA gold

DA 2023-08-10

ER

PT J

AU Normand, E

Franco, A

Marcil, V

AF Normand, Emilie

Franco, Anita

Marcil, Valerie

TI Nutrition and physical activity level of adolescents with idiopathic

scoliosis: a narrative review

SO SPINE JOURNAL

LA English

DT Review

DE Adolescent idiopathic scoliosis; Energy intake; Nutrient intake;

Nutrition; Nutritional status; Physical activity

ID BONE-MINERAL DENSITY; BODY-MASS INDEX; VITAMIN-D; CALCIUM INTAKE;

PREVALENCE; SERUM; ASSOCIATION; GHRELIN; WEIGHT; HEIGHT

AB Some studies have shown that patients with adolescent idiopathic scoliosis (AIS) have different anthropometric features compared with their peers such as taller stature, lower body mass index, and bone mineral density. Yet the causes explaining these differences remain uncertain. Nutritional intake and status, combined with physical activity, could explain these discrepancies. We aimed to review the current literature on energy and nutrient intake, on nutritional status and physical activity in relation to AIS and to discuss study methodologies and propose avenues for future studies.

Studies describing energy or nutrient intake in AIS mostly focused on total energy and calcium and found no difference between AIS and control cohorts. Regarding nutritional status, it was found that

AIS patients have lower vitamin D levels than controls and that most patients have insufficient or deficient vitamin D serum levels. Lower concentration of parathyroid hormones and calcitonin were also found in AIS compared to controls as well as anomalies in trace elements. In the studies that have assessed physical activity, three found that AIS girls were less active than controls, but four did not observe differences between groups. In this review, we highlight that nutrition and physical activity are important topics in AIS that require further research as they could help understand anthropometric discrepancies and disease etiology. (C) 2019 Elsevier Inc. All rights reserved.

C1 [Normand, Emilie; Franco, Anita; Marcil, Valerie] St Justine Univ Hosp, Res Ctr, 3175 Cote Ste Catherine Room 4-17-006, Montreal, PQ H3T 1C5, Canada.

[Normand, Emilie; Marcil, Valerie] Univ Montreal, Fac Med, Dept Nutr, Montreal, PQ H3T 1J4, Canada.

[Franco, Anita] St Justine Univ Hosp, Viscogliosi Lab Mol Genet Musculoskeletal Dis, Res Ctr, 3175 Cote Ste Catherine Room 4-17-006, Montreal, PQ H3T 1C5, Canada.

C3 Universite de Montreal; Universite de Montreal; Universite de Montreal

RP Marcil, V (通讯作者)，St Justine Univ Hosp, Res Ctr, 3175 Cote Ste Catherine Room 4-17-006, Montreal, PQ H3T 1C5, Canada.

EM valerie.marcil@umontreal.ca

FU Fondation Yves Cotrel -Institut de France

FX The authors thank Dr. Florina Moldovan for reviewing the manuscript.

This work was supported by a research grant from the Fondation Yves

Cotrel -Institut de France.

CR [Anonymous], 2018 PHYS ACT GUID A

Asher MA, 2006, SCOLIOSIS SPINAL DIS, V1, DOI 10.1186/1748-7161-1-2

BAECKE JAH, 1982, AM J CLIN NUTR, V36, P936

Balioglu MB, 2017, J PEDIATR ORTHOP B, V26, P48, DOI 10.1097/BPB.0000000000000320

Barr SI, 2001, J BONE MINER RES, V16, P940, DOI 10.1359/jbmr.2001.16.5.940

Barrios C, 2011, SPINE, V36, P1470, DOI 10.1097/BRS.0b013e3181f55083

Beck B, 2002, BIOCHEM BIOPH RES CO, V292, P1031, DOI 10.1006/bbrc.2002.6737

Bedford JL, 2011, NUTRIENTS, V3, P951, DOI 10.3390/nu3110951

BJURE J, 1968, SCAND J CLIN LAB INV, V21, P190, DOI 10.3109/00365516809084283

Booth SL, 2003, AM J CLIN NUTR, V77, P512, DOI 10.1093/ajcn/77.2.512

Cheng JCY, 1999, SPINE, V24, P1218, DOI 10.1097/00007632-199906150-00008

Cheng JCY, 2000, J BONE MINER RES, V15, P1587, DOI 10.1359/jbmr.2000.15.8.1587

Cheng JCY., 2007, HONG KONG MED J, V13, pS33

Cheuk KY, 2015, CALCIFIED TISSUE INT, V97, P343, DOI 10.1007/s00223-015-0025-2

Cheung CSK, 2006, SPINE, V31, P330, DOI 10.1097/01.brs.0000197410.92525.10

Considine RV, 1996, NEW ENGL J MED, V334, P292, DOI 10.1056/NEJM199602013340503

Dastych M, 2008, J ORTHOP RES, V26, P1279, DOI 10.1002/jor.20629

Dastych M, 2002, BIOL TRACE ELEM RES, V89, P105, DOI 10.1385/BTER:89:2:105

Date Y, 2000, ENDOCRINOLOGY, V141, P4255, DOI 10.1210/en.141.11.4255

Dawson-Hughes B, 2005, OSTEOPOROSIS INT, V16, P713, DOI 10.1007/s00198-005-1867-7

Diarbakerli E, 2016, SCOLIOSIS SPINAL DIS, V11, DOI 10.1186/s13013-016-0082-y

DICKSON RA, 1980, BRIT MED J, V281, P265, DOI 10.1136/bmj.281.6235.265

Du Q, 2016, BMC MUSCULOSKEL DIS, V17, DOI 10.1186/s12891-016-1140-6

FAVUS MJ, 1985, AM J PHYSIOL, V248, pG147, DOI 10.1152/ajpgi.1985.248.2.G147

Gozdzialska A, 2016, SPINE, V41, P693, DOI 10.1097/BRS.0000000000001286

Gracia-Marco L, 2011, EUR J APPL PHYSIOL, V111, P2671, DOI 10.1007/s00421-011-1897-0

Hershkovich O, 2014, SPINE J, V14, P1581, DOI 10.1016/j.spinee.2013.09.034

Hollis BW, 1996, CALCIFIED TISSUE INT, V58, P4, DOI 10.1007/s002239900002

Ilich JZ, 1998, J AM COLL NUTR, V17, P136, DOI 10.1080/07315724.1998.10718739

Julian-Almarcegui C, 2015, NUTR REV, V73, P127, DOI 10.1093/nutrit/nuu065

Khundmiri SJ, 2016, COMPR PHYSIOL, V6, P561, DOI 10.1002/cphy.c140071

Kim J, 2008, ASIA PAC J CLIN NUTR, V17, P270

Lee WTK, 2005, OSTEOPOROSIS INT, V16, P1024, DOI 10.1007/s00198-004-1792-1

Li XF, 2008, EUR SPINE J, V17, P1431, DOI 10.1007/s00586-008-0757-z

Matusik E, 2016, NUTRIENTS, V8, DOI 10.3390/nu8020071

Medicine Io, 1997, DIET REF INT CALC PH

Michaelsson K, 2003, NEW ENGL J MED, V348, P287, DOI 10.1056/NEJMoa021171

Mindell JS, 2014, P NUTR SOC, V73, P218, DOI 10.1017/S0029665113003820

MORAIS T, 1985, AM J PUBLIC HEALTH, V75, P1377, DOI 10.2105/AJPH.75.12.1377

Negrini S, 2018, SCOLIOSIS SPINAL DIS, V13, DOI 10.1186/s13013-017-0145-8

Ng SY, 2018, ASIAN SPINE J, V12, P1127, DOI 10.31616/asj.2018.12.6.1127

NISSINEN M, 1993, ACTA PAEDIATR, V82, P77, DOI 10.1111/j.1651-2227.1993.tb12521.x

Ocke MC, 2013, P NUTR SOC, V72, P191, DOI 10.1017/S0029665113000013

Opoku H., 2018, SPINE DEFORM, V6, P220, DOI [10.1016/j.jspd.2017.09.052, DOI 10.1016/J.JSPD.2017.09.052]

Otten J. J., 2006, DIETARY REFERENCE IN

POLS MA, 1995, INT J EPIDEMIOL, V24, P381, DOI 10.1093/ije/24.2.381

Rockett HRH, 1997, PREV MED, V26, P808, DOI 10.1006/pmed.1997.0200

Ross AC, 2011, Dietary Reference Intakes for Calcium and Vitamin D, P1

Sadat-Ali M, 2008, EUR SPINE J, V17, P944, DOI 10.1007/s00586-008-0671-4

Sanders A.E., 2018, SPINE DEFORM, V6, P435, DOI [10.1016/j.jspd.2017.12.014, DOI 10.1016/J.JSPD.2017.12.014]

SCRAGG R, 1995, AUST NZ J MED, V25, P218, DOI 10.1111/j.1445-5994.1995.tb01526.x

Shen CL, 2018, OSTEOPOROSIS INT, V29, P881, DOI 10.1007/s00198-017-4356-x

Shi BL, 2016, EUR SPINE J, V25, P3341, DOI 10.1007/s00586-016-4530-4

Shim JS, 2014, EPIDEMIOL HEALTH, V36, DOI 10.4178/epih/e2014009

SLEMENDA CW, 1991, J BONE MINER RES, V6, P1227

SNYDER BD, 1995, SPINE, V20, P1554, DOI 10.1097/00007632-199507150-00002

Suh KT, 2010, EUR SPINE J, V19, P1545, DOI 10.1007/s00586-010-1385-y

Tam EMS, 2016, SPINE, V41, P940, DOI 10.1097/BRS.0000000000001376

Tarrant RC, 2018, EUR J CLIN NUTR, V72, P1463, DOI 10.1038/s41430-018-0095-0

Tarrant RC, 2015, EUR SPINE J, V24, P281, DOI 10.1007/s00586-014-3622-2

Teegarden D, 1998, AM J CLIN NUTR, V68, P749, DOI 10.1093/ajcn/68.3.749

Silva RTE, 2017, ACTA ORTOP BRAS, V25, P15, DOI 10.1590/1413-785220172501168600

Thompson FE, 2017, NUTRITION IN THE PREVENTION AND TREATMENT OF DISEASE, 4TH EDITION, P5, DOI 10.1016/B978-0-12-802928-2.00001-1

Tucker KL, 2005, J BONE MINER RES, V20, P152, DOI [10.1359/JBMR.041018, 10.1359/jbmr.2005.20.1.152]

Tyrakowski M, 2014, EUR SPINE J, V23, P1244, DOI 10.1007/s00586-014-3275-1

van den Hooven EH, 2015, AM J CLIN NUTR, V102, P1035, DOI 10.3945/ajcn.115.110502

Wang WJ, 2010, EUR SPINE J, V19, P726, DOI 10.1007/s00586-009-1247-7

Wren AM, 2001, J CLIN ENDOCR METAB, V86, P5992, DOI 10.1210/jc.86.12.5992

Yawn BP, 1999, JAMA-J AM MED ASSOC, V282, P1427, DOI 10.1001/jama.282.15.1427

Yin XH, 2018, MEDICINE, V97, DOI 10.1097/MD.0000000000013822

Yu HJ, 2018, MATH PROBL ENG, V2018, DOI 10.1155/2018/5147565

Yu WS, 2014, BONE, V61, P109, DOI 10.1016/j.bone.2013.12.033

Zheng Y, 2017, J REHABIL MED, V49, P512, DOI 10.2340/16501977-2240

Zhong Y, 2009, J BIOL CHEM, V284, P11059, DOI 10.1074/jbc.M806561200

NR 74

TC 4

Z9 6

U1 2

U2 11

PU ELSEVIER SCIENCE INC

PI NEW YORK

PA STE 800, 230 PARK AVE, NEW YORK, NY 10169 USA

SN 1529-9430

EI 1878-1632

J9 SPINE J

JI Spine Journal

PD MAY

PY 2020

VL 20

IS 5

BP 785

EP 799

DI 10.1016/j.spinee.2019.11.012

PG 15

WC Clinical Neurology; Orthopedics

WE Science Citation Index Expanded (SCI-EXPANDED)

SC Neurosciences & Neurology; Orthopedics

GA LP7TT

UT WOS:000534521900014

PM 31783126

DA 2023-08-10

ER

PT J

AU Zhang, YB

Zhang, JG

AF Zhang, Yan-Bin

Zhang, Jian-Guo

TI Treatment of early-onset scoliosis: techniques, indications, and

complications

SO CHINESE MEDICAL JOURNAL

LA English

DT Review

DE Early-onset scoliosis; Techniques; Conservative treament; Hemivertebra

resection; Fusionless; Growth-friendly

ID SHILLA GROWTH GUIDANCE; GROWING-ROD SURGERY; PROSTHETIC TITANIUM RIB;

SHORT SEGMENTAL FUSION; CONGENITAL SCOLIOSIS; IDIOPATHIC SCOLIOSIS;

RISK-FACTORS; SPINAL DEFORMITY; MANAGEMENT; CHILDREN

AB The treatments for early-onset scoliosis (EOS) remain great challenges for spine surgeons. This study aimed to comprehensively review the treatments for EOS, especially the advancements made in the last decade. Current studies on EOS were retrieved through a search on PubMed, UpToDate, the Web of Science Core Collection and Scopus were reviewed. The most pertinent information related to the current treatments for EOS was collected. The foci of treatments for EOS have included creating a well-developed thoracic cavity, improving lung volume, and improving pulmonary function. Conservative treatments include bracing, casting, halo-gravity traction, and physiotherapy. Serial casting is the most effective conservative treatment for EOS. Surgical treatments mainly include growth-friendly techniques, which are generally classified into three types according to the amount of correction force applied: distraction-based, compression-based, and growth-guided. The distraction-based systems include traditional or conventional growing rods, magnetically controlled growing rods, and vertical expandable prosthesis titanium ribs. The compression-based systems include vertebral body stapling and tethering. The growth-guided systems include the Shilla system and modern Luque trolley. In addition, some newer techniques have emerged in recent years, such as posterior dynamic deformity correction (ApiFix). For EOS patients presenting with sharp deformities in a long, congenital spinal deformity, a hybrid technique, one-stage posterior osteotomy with short segmental fusion and dual growing rods, may be a good choice. Hemivertebra resection is the gold standard for congenital scoliosis caused by single hemivertebra. Although the patient's growth potential is preserved in growth-friendly surgeries, a high complication rate should be expected, as well as a prolonged treatment duration and additional costs. Knowledge about EOS and its treatment options is rapidly expanding. Conservative treatments have specific limitations. For curves requiring a surgical intervention, surgical techniques may vary depending on the patients' characteristics, the surgeon's experience, and the actual state of the country.

C1 [Zhang, Yan-Bin] Peking Univ, Beijing Jishuitan Hosp, Dept Educ, Clin Med Coll 4, Beijing 100035, china.

[Zhang, Jian-Guo] Chinese Acad Med Sci & Peking Union Med Coll, Peking Union Med Coll Hosp, Dept Orthoped, Beijing 100730, china.

C3 Peking University; Chinese Academy of Medical Sciences - Peking Union

Medical College; Peking Union Medical College; Peking Union Medical

College Hospital

RP Zhang, JG (通讯作者)， Peking Union Med Coll Hosp, Dept Orthoped, 1 Shuaifuyuan Rd, Beijing 100730, china.

EM jgzhang_pumch@yahoo.com

RI zhang, jian/HPD-1712-2023; jin, li/IWU-4648-2023; zhan, y/ISA-2807-2023

FU National Natural Science Foundation of China [81972037]

FX The study was granted by a grant from the National Natural Science

Foundation of China (No. 81972037).

CR Akbarnia BA, 2005, SPINE, V30, pS46, DOI 10.1097/01.brs.0000175190.08134.73

Akbarnia BA, 2013, SPINE, V38, P665, DOI 10.1097/BRS.0b013e3182773560

Alkhalife YI, 2019, ORTHOP CLIN N AM, V50, P57, DOI 10.1016/j.ocl.2018.08.014

Aslan C, 2019, SPINE, V44, pE656, DOI 10.1097/BRS.0000000000002938

Baulesh DM, 2012, J PEDIATR ORTHOPED, V32, P658, DOI 10.1097/BPO.0b013e318269c438

Bess S, 2010, J BONE JOINT SURG AM, V92A, P2533, DOI 10.2106/JBJS.I.01471

Betz RR, 2010, SPINE, V35, P169, DOI 10.1097/BRS.0b013e3181c6dff5

Bumpass DB, 2015, SPINE, V40, pE1305, DOI 10.1097/BRS.0000000000001135

Cahill PJ, 2018, SPINE DEFORMITY, V6, P28, DOI [10.1016/j.jspd.2017.03.004, DOI 10.1016/J.JSPD.2017.03.004]

Campbell RM, 2004, J BONE JOINT SURG AM, V86A, P1659, DOI 10.2106/00004623-200408000-00009

Canavese F, 2015, WORLD J ORTHOP, V6, P935, DOI 10.5312/wjo.v6.i11.935

Canavese F, 2013, WORLD J ORTHOP, V4, P167, DOI 10.5312/wjo.v4.i4.167

Cao J, 2017, J ORTHOP SURG RES, V12, DOI 10.1186/s13018-017-0554-7

Cheung JPY, 2016, J ORTHOP SURG-HONG K, V24, P332, DOI 10.1177/1602400312

Crawford CH, 2010, J BONE JOINT SURG AM, V92A, P202, DOI 10.2106/JBJS.H.01728

Cyr M, 2017, J PEDIATR ORTHOPED, V37, pE1, DOI 10.1097/BPO.0000000000000688

Dede O, 2016, J PEDIATR ORTHOPED, V36, P336, DOI 10.1097/BPO.0000000000000467

El-Hawary Ron, 2015, Spine Deform, V3, P105, DOI 10.1016/j.jspd.2015.01.003

Fedorak GT, 2019, J PEDIATR ORTHOPED, V39, pE303, DOI 10.1097/BPO.0000000000001288

Fletcher ND, 2012, J PEDIATR ORTHOPED, V32, P664, DOI 10.1097/BPO.0b013e31824bdb55

Floman Y, 2015, SCOLIOSIS SPINAL DIS, V10, DOI 10.1186/s13013-015-0028-9

Guille JT, 2007, ORTHOP CLIN N AM, V38, P541, DOI 10.1016/j.ocl.2007.07.003

Gussous Yazeed M, 2015, Spine Deform, V3, P233, DOI 10.1016/j.jspd.2014.10.001

Hardesty C.K., 2018, SPINE DEFORM, V6, P467, DOI [DOI 10.1016/J.JSPD.2017.12.012, 10.1016/j.jspd.2017.12.012]

HARRINGTON PR, 1962, J BONE JOINT SURG AM, V44, P591, DOI 10.2106/00004623-196244040-00001

Iorio J, 2017, J PEDIATR ORTHOPED, V37, P311, DOI 10.1097/BPO.0000000000000654

Iyer S, 2019, SPINE, V44, pE841, DOI 10.1097/BRS.0000000000002997

Karol LA, 2008, J BONE JOINT SURG AM, V90A, P1272, DOI 10.2106/JBJS.G.00184

La Rosa G, 2017, J PEDIATR ORTHOPED, V37, P79, DOI 10.1097/BPO.0000000000000597

Liang JQ, 2015, CLIN NEUROL NEUROSUR, V136, P15, DOI 10.1016/j.clineuro.2015.05.026

Luhmann SJ, 2017, J PEDIATR ORTHOPED, V37, pE567, DOI 10.1097/BPO.0000000000000751

McCarthy RE, 2015, J BONE JOINT SURG AM, V97A, P1578, DOI 10.2106/JBJS.N.01083

McCarthy RE, 2014, J PEDIATR ORTHOPED, V34, P1, DOI 10.1097/BPO.0b013e31829f92dc

MCMASTER MJ, 1982, J BONE JOINT SURG AM, V64, P1128, DOI 10.2106/00004623-198264080-00003

Mehta MH, 2005, J BONE JOINT SURG BR, V87B, P1237, DOI 10.1302/0301-620X.87B9.16124

Moreau S, 2014, ORTHOP TRAUMATOL-SUR, V100, P935, DOI 10.1016/j.otsr.2014.05.024

NACHLAS IW, 1951, J BONE JOINT SURG AM, V33-A, P24, DOI 10.2106/00004623-195133010-00002

Ouellet J, 2011, CLIN ORTHOP RELAT R, V469, P1356, DOI 10.1007/s11999-011-1783-4

Piantoni Lucas, 2015, Spine Deform, V3, P541, DOI 10.1016/j.jspd.2015.04.008

Samdani AF, 2015, EUR SPINE J, V24, P1533, DOI 10.1007/s00586-014-3706-z

Sanders JO, 2009, J PEDIATR ORTHOPED, V29, P581, DOI 10.1097/BPO.0b013e3181b2f8df

Shah SA, 2014, SPINE, V39, pE1311, DOI 10.1097/BRS.0000000000000565

SMITH AD, 1954, J BONE JOINT SURG AM, V36-A, P342, DOI 10.2106/00004623-195436020-00011

Sun X, 2019, SPINE, V44, P707, DOI 10.1097/BRS.0000000000002933

Sun ZJ, 2015, EUR SPINE J, V24, P1434, DOI 10.1007/s00586-014-3668-1

Takaso M, 1998, J Orthop Sci, V3, P336, DOI 10.1007/s007760050062

Teoh KH, 2016, SPINE J, V16, pS40, DOI 10.1016/j.spinee.2015.12.099

Thometz J, 2019, J PEDIATR ORTHOPED, V39, pE185, DOI 10.1097/BPO.0000000000001287

Thometz J, 2018, SCOLIOSIS SPINAL DIS, V13, DOI 10.1186/s13013-018-0160-4

Trobisch PD, 2011, OPER ORTHOP TRAUMATO, V23, P227, DOI 10.1007/s00064-011-0032-z

Waldhausen JHT, 2016, J PEDIATR SURG, V51, P1747, DOI 10.1016/j.jpedsurg.2016.06.014

Wang SR, 2017, SPINE, V42, P1687, DOI 10.1097/BRS.0000000000002197

Wang SR, 2014, SPINE, V39, pE294, DOI 10.1097/BRS.0000000000000119

Watanabe K, 2016, CLIN SPINE SURG, V29, pE428, DOI 10.1097/BSD.0000000000000127

Watanabe K, 2013, SPINE, V38, pE464, DOI 10.1097/BRS.0b013e318288671a

Weinstein SL, 2013, NEW ENGL J MED, V369, P1512, DOI 10.1056/NEJMoa1307337

Welborn MC, 2019, J PEDIATR ORTHOPED, V39, pE293, DOI 10.1097/BPO.0000000000001282

Wilkinson JT, 2019, J PEDIATR ORTHOPED, V39, P400, DOI 10.1097/BPO.0000000000000983

Williams BA, 2014, J BONE JOINT SURG AM, V96A, P1359, DOI 10.2106/JBJS.M.00253

Xu EJ, 2019, WORLD NEUROSURG, V126, pE173, DOI 10.1016/j.wneu.2019.01.290

Yang JS, 2010, J PEDIATR ORTHOPED, V30, P264, DOI 10.1097/BPO.0b013e3181d40f94

Yang S, 2016, PEDIATRICS, V137, DOI 10.1542/peds.2015-0709

Yazici M, 2009, SPINE, V34, P1800, DOI 10.1097/BRS.0b013e3181978ec9

NR 63

TC 27

Z9 32

U1 5

U2 16

PU LIPPINCOTT WILLIAMS & WILKINS

PI PHILADELPHIA

PA TWO COMMERCE SQ, 2001 MARKET ST, PHILADELPHIA, PA 19103 USA

SN 0366-6999

EI 2542-5641

J9 CHINESE MED J-PEKING

JI Chin. Med. J.

PD FEB 5

PY 2020

VL 133

IS 3

BP 351

EP 357

DI 10.1097/CM9.0000000000000614

PG 7

WC Medicine, General & Internal

WE Science Citation Index Expanded (SCI-EXPANDED)

SC General & Internal Medicine

GA KJ3TR

UT WOS:000511982300013

PM 31904727

OA gold, Green Published

DA 2023-08-10

ER

PT J

AU Gao, CF

Zheng, Y

Fan, CJ

Yang, Y

He, CQ

Wong, MS

AF Gao, Chengfei

Zheng, Yu

Fan, Chunjiang

Yang, Yan

He, Chengqi

Wong, Mansang

TI Could the Clinical Effectiveness Be Improved Under the Integration of

Orthotic Intervention and Scoliosis-Specific Exercise in Managing

Adolescent Idiopathic Scoliosis? A Randomized Controlled Trial Study

SO AMERICAN JOURNAL OF PHYSICAL MEDICINE & REHABILITATION

LA English

DT Article

DE Adolescent Idiopathic Scoliosis; Orthotic Intervention;

Scoliosis-Specific Exercise; Spinal Deformity; Back Muscle Function;

Pulmonary Function

ID BIERING-SORENSEN TEST; BRACE; BACK; EFFICACY; VALIDITY

AB Objective The aim of the study was to compare the effectiveness of the integration of orthotic intervention and scoliosis-specific exercise with orthotic intervention only via assessing the spinal deformity, back muscle endurance, and pulmonary function of the patients with adolescent idiopathic scoliosis. Design It is a prospective randomized controlled study. Patients who fulfilled the SRS criteria for orthotic intervention were randomly assigned to the orthosis combined with exercise group (combined orthotic and exercise intervention) or the orthotic intervention group (orthotic intervention only). All the subjects were prescribed with a rigid thoracolumbosacral orthosis and scoliosis-specific exercise program was provided to the subjects in the orthosis combined with exercise group. Cobb angle, back muscle endurance, and pulmonary function of subjects were measured at baseline, 1-mo, and 6-mo follow-up visits. Results After 6 mos of intervention, the subjects in the orthosis combined with exercise group showed better Cobb angle correction than those in the orthotic intervention group. The back muscle endurance and pulmonary function decreased in the subjects of the orthotic intervention group, whereas some improvement happened in the subjects of the orthosis combined with exercise group. Between-group statistical significance was detected at the 6-mo follow-up among back muscle endurance time and parameters of pulmonary function. Conclusions In this study, orthotic intervention combined with scoliosis-specific exercise offered better Cobb angle correction and improvement of the respiratory parameters and back muscle endurance of the patients with adolescent idiopathic scoliosis as compared with orthotic intervention only.

C1 [Gao, Chengfei; He, Chengqi] Sichuan Univ, West China Hosp, Ctr Rehabil Med, 37 Guo Xue Xiang, Chengdu 610041, Sichuan, china.

[Gao, Chengfei; Wong, Mansang] Hong Kong Polytech Univ, Dept Biomed Engn, Hong Kong 999077, china.

[He, Chengqi; Wong, Mansang] Hong Kong Polytech Univ, Sichuan Univ, Inst Disaster Management & Reconstruct, Chengdu, Sichuan, china.

[Zheng, Yu] Nanjing Med Univ, Affiliated Hosp 1, Dept Rehabil Med, Nanjing, Jiangsu, china.

[Gao, Chengfei; Zheng, Yu; Fan, Chunjiang; Yang, Yan; Wong, Mansang] Wuxi Rehabil Hosp, Dept Rehabil Med, Wuxi, Jiangsu, china.

C3 Sichuan University; Hong Kong Polytechnic University; Hong Kong

Polytechnic University; Sichuan University; Nanjing Medical University

RP He, CQ (通讯作者)， Sichuan Univ, West China Hosp, Ctr Rehabil Med, 37 Guo Xue Xiang, Chengdu 610041, Sichuan, china.; Wong, MS (通讯作者)， Hong Kong Polytech Univ, Dept Biomed Engn, Hong Kong 999077, china.

OI ZHENG, Yu/0000-0003-4891-5472; WONG, MS/0000-0002-4157-9528

CR Berdishevsky H, 2016, SCOLIOSIS SPINAL DIS, V11, DOI 10.1186/s13013-016-0076-9

Cobb JR., 1948, INSTR COURSE LECT, V5, P261

Coorevits P, 2008, J ELECTROMYOGR KINES, V18, P997, DOI 10.1016/j.jelekin.2007.10.012

Danielsson AJ, 2006, SPINE, V31, P275, DOI 10.1097/01.brs.0000197652.52890.71

Eisinger DB, 1996, AM J PHYS MED REHAB, V75, P194, DOI 10.1097/00002060-199605000-00008

Hedayati Z, 2018, AM J PHYS MED REHAB, V97, P104, DOI 10.1097/PHM.0000000000000812

Hresko MT, 2013, NEW ENGL J MED, V368, P834, DOI 10.1056/NEJMcp1209063

Jorgensen K, 1997, ACTA PHYSIOL SCAND, V160, P5

Kalichman L, 2016, J BODYW MOV THER, V20, P56, DOI 10.1016/j.jbmt.2015.04.007

Katz DE, 1997, SPINE, V22, P1302, DOI 10.1097/00007632-199706150-00005

KENNEDY JD, 1987, THORAX, V42, P959, DOI 10.1136/thx.42.12.959

Korovessis P, 1996, SPINE, V21, P1979, DOI 10.1097/00007632-199609010-00008

Kwan KYH, 2017, SCOLIOSIS SPINAL DIS, V12, DOI 10.1186/s13013-017-0139-6

Latimer J, 1999, SPINE, V24, P2085, DOI 10.1097/00007632-199910150-00004

Moramarco M, 2016, CURR PEDIATR REV, V12, P17, DOI 10.2174/1573396312666151117120514

Mordecai SC, 2012, EUR SPINE J, V21, P382, DOI 10.1007/s00586-011-2063-4

Muller C, 2011, EUR SPINE J, V20, P1127, DOI 10.1007/s00586-011-1791-9

Negrini S, 2012, SCOLIOSIS SPINAL DIS, V7, DOI 10.1186/1748-7161-7-3

Negrini S, 2009, SCOLIOSIS SPINAL DIS, V4, DOI 10.1186/1748-7161-4-2

Negrini S, 2009, SCOLIOSIS SPINAL DIS, V4, DOI 10.1186/1748-7161-4-19

PONSETI IV, 1950, J BONE JOINT SURG AM, V32-A, P381, DOI 10.2106/00004623-195032020-00017

Richards BS, 2005, SPINE, V30, P2068, DOI 10.1097/01.brs.0000178819.90239.d0

Romano M, 2006, SCOLIOSIS, V1, P1

Romano M, 2015, SCOLIOSIS SPINAL DIS, V10, DOI 10.1186/s13013-014-0027-2

Romano M, 2013, SPINE, V38, pE883, DOI 10.1097/BRS.0b013e31829459f8

Schreiber S, 2015, SCOLIOSIS SPINAL DIS, V10, DOI 10.1186/s13013-015-0048-5

Weinstein SL, 2013, NEW ENGL J MED, V369, P1512, DOI 10.1056/NEJMoa1307337

Weiss HR, 2009, STUD HEALTH TECHNOL, V135, P164

Wong MS, 2008, SPINE, V33, P1360, DOI 10.1097/BRS.0b013e31817329d9

Zamecnik J, 2016, MOL MED REP, V14, P5719, DOI 10.3892/mmr.2016.5927

Zheng Y, 2018, SPINE, V43, pE494, DOI 10.1097/BRS.0000000000002412

NR 31

TC 16

Z9 18

U1 3

U2 28

PU LIPPINCOTT WILLIAMS & WILKINS

PI PHILADELPHIA

PA TWO COMMERCE SQ, 2001 MARKET ST, PHILADELPHIA, PA 19103 USA

SN 0894-9115

EI 1537-7385

J9 AM J PHYS MED REHAB

JI Am. J. Phys. Med. Rehabil.

PD AUG

PY 2019

VL 98

IS 8

BP 642

EP 648

DI 10.1097/PHM.0000000000001160

PG 7

WC Rehabilitation; Sport Sciences

WE Science Citation Index Expanded (SCI-EXPANDED)

SC Rehabilitation; Sport Sciences

GA IJ5NB

UT WOS:000475948700008

PM 31318743

OA Green Accepted

DA 2023-08-10

ER

PT J

AU Tobias, JH

Fairbank, J

Harding, I

Taylor, HJ

Clark, EM

AF Tobias, Jon H.

Fairbank, Jeremy

Harding, Ian

Taylor, Hilary J.

Clark, Emma M.

TI Association between physical activity and scoliosis: a prospective cohort study

SO INTERNATIONAL JOURNAL OF EPIDEMIOLOGY

LA English

DT Article

DE Scoliosis; ALSPAC; cohort study; physical activity

ID ADOLESCENT IDIOPATHIC SCOLIOSIS; BONE MASS; EXERCISE CAPACITY;

FOLLOW-UP; LIMITATION; CHILDREN

AB Background Little is understood about the causes of adolescent onset idiopathic scoliosis (AIS). No prospective studies assessing the association between physical activity and idiopathic adolescent scoliosis have been carried out. We aimed to carry out the first prospective population-based study of this association.

Methods The Avon Longitudinal Study of Parents and Children (ALSPAC) collected self-reported measures of physical ability/activity at ages 18months and 10years. Objective measures of physical activity were collected by accelerometry at age 11years. scoliosis was identified using the dxa scoliosis Method at age 15years. Participants with scoliosis at age 10years were excluded.

Results Of 4640 participants at age 15years who had DXA scans, 267 (5.8%) had scoliosis. At age 18months, those infants who were able to stand up without being supported were 66% less likely to have developed scoliosis by age 15 (P=0.030) compared with infants who could not. Those children whose mothers reported they did most vigorous physical activity at age 10years were 53% less likely to develop scoliosis (P=0.027). Those children who did more objectively measured moderate/vigorous physical activity at age 11 were 30% less likely to have developed scoliosis (P<0.001). Results were not affected by adjustment for age, gender, lean mass, fat mass or back pain.

Conclusions We report reduced physical ability and activity as early as age 18months in those who go on to develop scoliosis by age 15years. Further research is justified to examine the mechanisms underlying this association.

C1 [Tobias, Jon H.; Taylor, Hilary J.; Clark, Emma M.] Univ Bristol, Southmead Hosp, Musculoskeletal Res Unit, Bristol, Avon, united kingdom.

[Fairbank, Jeremy] Univ Oxford, Nuffield Orthopaed Ctr, Nuffield Dept Orthopaed, Oxford, united kingdom.

[Harding, Ian] North Bristol NHS Trust, Southmead Hosp, Musculoskeletal Directorate, Bristol, Avon, united kingdom.

C3 Southmead Hospital; University of Bristol; Nuffield Orthopaedic Centre;

University of Oxford; North Bristol NHS Trust; Southmead Hospital

RP Clark, EM (通讯作者)，Univ Bristol, Southmead Hosp, Avon Orthopaed Ctr, Musculoskeletal Res Unit, Bristol BS10 5NB, Avon, united kingdom.

EM emma.clark@bristol.ac.uk

RI Fairbank, Jeremy/AAA-3411-2019; Tobias, Jon/E-2832-2014

OI Tobias, Jon/0000-0002-7475-3932; Taylor, Hilary/0000-0002-7948-5535;
Clark, Emma/0000-0001-8332-9052

FU British Scoliosis Research Foundation; Wellcome Trust [102215/2/13/2];
MRC [MC_PC_19009] Funding Source: UKRI

FX This research was supported by the British Scoliosis Research
Foundation. The UK Medical Research Council, the Wellcome Trust (grant
102215/2/13/2) and the University of Bristol provide core support for
ALSPAC.

CR AARO S, 1984, SPINE, V9, P220, DOI 10.1097/00007632-198403000-00015
Barrios C, 2011, SPINE, V36, P1470, DOI 10.1097/BRS.0b013e3181f55083
Boyd A, 2013, INT J EPIDEMIOL, V42, P111, DOI 10.1093/ije/dys064
Burwell RG, 2016, SCOLIOSIS SPINAL DIS, V11, DOI 10.1186/s13013-016-0063-1
Clark EM, 2005, J BONE MINER RES, V20, P2082, DOI 10.1359/JBMR.050808
Clark EM, 2008, J BONE MINER RES, V23, P1012, DOI 10.1359/JBMR.080303
Clark EM, 2016, SPINE, V41, pE611, DOI 10.1097/BRS.0000000000001330
Clark EM, 2014, J BONE MINER RES, V29, P1729, DOI 10.1002/jbmr.2207
Cobb JR., 1948, INSTR COURSE LECT, V5, P261
Czaprowski D, 2012, EUR SPINE J, V21, P1099, DOI 10.1007/s00586-011-2068-z
Dagli A. I., 1998, GENEREVIEWS
Diarbakerli E, 2016, SCOLIOSIS SPINAL DIS, V11, DOI 10.1186/s13013-016-0082-y
FOWLES JV, 1978, CLIN ORTHOP RELAT R, P212
FRANKENBURG WK, 1967, J PEDIATR-US, V71, P181, DOI 10.1016/S0022-3476(67)80070-2
KANE WJ, 1977, CLIN ORTHOP RELAT R, P43
Karachalios T, 1999, SPINE, V24, P2318, DOI 10.1097/00007632-199911150-00006
Kenanidis E, 2008, SPINE, V33, P2160, DOI 10.1097/BRS.0b013e31817d6db3

KESTEN S, 1991, CHEST, V99, P663, DOI 10.1378/chest.99.3.663

Lee WTK, 2005, OSTEOPOROSIS INT, V16, P1024, DOI 10.1007/s00198-004-1792-1

Mahaudens P, 2009, EUR SPINE J, V18, P1160, DOI 10.1007/s00586-009-1002-0

Martinez-Llorens J, 2010, EUR RESPIR J, V36, P393, DOI 10.1183/09031936.00025509

Meyer C, 2006, SCAND J MED SCI SPOR, V16, P231, DOI 10.1111/j.1600-0838.2005.00482.x

Meyer C, 2008, SCAND J MED SCI SPOR, V18, P751, DOI 10.1111/j.1600-0838.2007.00750.x

Miller NH, 1999, ORTHOP CLIN N AM, V30, P343, DOI 10.1016/S0030-5898(05)70091-2

Mohammadi P, 2014, PHYSIOTHER THEOR PR, V30, P552, DOI 10.3109/09593985.2014.938382

Parsch D, 2002, CLIN J SPORT MED, V12, P95, DOI 10.1097/00042752-200203000-00005

Sperandio EF, 2014, SPINE J, V14, P2366, DOI 10.1016/j.spinee.2014.01.041

Steppan CM, 1999, BIOCHEM BIOPH RES CO, V256, P600, DOI 10.1006/bbrc.1999.0382

Tanner JM, 1978, TXB PEDIAT

Taylor HJ, 2013, CALCIFIED TISSUE INT, V92, P539, DOI 10.1007/s00223-013-9713-y

Tobias JH, 2007, J BONE MINER RES, V22, P101, DOI 10.1359/JBMR.060913

WEINSTEIN SL, 1983, J BONE JOINT SURG AM, V65, P447, DOI 10.2106/00004623-198365040-00004

WEINSTEIN SL, 1981, J BONE JOINT SURG AM, V63, P702, DOI 10.2106/00004623-198163050-00003

Weinstein SL, 2008, LANCET, V371, P1527, DOI 10.1016/S0140-6736(08)60658-3

Zapata KA, 2015, PEDIATR PHYS THER, V27, P396, DOI 10.1097/PEP.0000000000000174

NR 35

TC 14

Z9 15

U1 14

U2 39

PU OXFORD UNIV PRESS

PI OXFORD

PA GREAT CLARENDON ST, OXFORD OX2 6DP, united kingdom

SN 0300-5771

EI 1464-3685

J9 INT J EPIDEMIOL

JI Int. J. Epidemiol.

PD AUG

PY 2019

VL 48

IS 4

BP 1152

EP 1160

DI 10.1093/ije/dyy268

PG 9

WC Public, Environmental & Occupational Health

WE Science Citation Index Expanded (SCI-EXPANDED)

SC Public, Environmental & Occupational Health

GA IV6MU

UT WOS:000484383300021

PM 30535285

OA Green Submitted, Green Published, Bronze

DA 2023-08-10

ER

PT J

AU Segreto, FA

Messina, JC

Doran, JP

Walker, SE

Aylyarov, A

Shah, NV

Mixa, PJ

Ahmed, N

Paltoo, K

Opare-Sem, K

Kaur, H

Day, LM

Naziri, Q

Paulino, CB

Scott, CB

Hesham, K

Urban, WP

Diebo, BG

AF Segreto, Frank A.

Messina, James C.

Doran, James P.

Walker, Sarah E.

Aylyarov, Alexandr

Shah, Neil, V

Mixa, Patrick J.

Ahmed, Natasha

Paltoo, Karen

Opare-Sem, Kwaku

Kaur, Harleen

Day, Louis M.

Naziri, Qais

Paulino, Carl B.

Scott, Claude B.

Hesham, Khalid

Urban, William P.

Diebo, Bassel G.

TI Noncontact sports participation in adolescent idiopathic scoliosis:

effects on parent-reported and patient-reported outcomes

SO JOURNAL OF PEDIATRIC ORTHOPAEDICS-PART B

LA English

DT Article

DE adolescent idiopathic scoliosis; parent-reported outcome;

patient-reported outcome; pediatric spinal deformity; perception; sports

participation

ID QUALITY-OF-LIFE

AB Comparing risks against benefits of adolescent idiopathic scoliosis (AIS) patients participating in sports represents a controversial topic in the literature. Previous studies have reported sports participation as a possible risk factor for AIS development, while others describe its functional benefits for AIS athletes. The objective of this study was to determine if sports participation had an impact on pain, function, mental status, and self-perception of deformity in patients and their parents. Patients had full spine radiographs and completed baseline surveys of demographics, socioeconomics, and patient-reported outcomes (PRO): Scoliosis Research Society (SRS)-30, Body Image Disturbance Questionnaire, and Spinal Appearance Questionnaire (SAQ: Children and Parent). Patients were grouped by their participation (sports) or nonparticipation (no-sports) in noncontact sports. Demographics, radiographic parameters, and PRO were compared using parametric/nonparametric tests with means/medians reported. Linear regression models identified significant predictors of PRO. Forty-nine patients were included (sports: n=29, no-sports: n=20).

Both groups had comparable age, sex, BMI, bracing status, and history of physical therapy (all P>0.05). Sports and no-sports also had similar coronal deformity (major Cobb: 31.1 degrees vs. 31.5 degrees). Sagittal alignment profiles (pelvic incidence, pelvic incidence minus lumbar lordosis, thoracic kyphosis, and sagittal vertical axis) were similar between groups (all P>0.05). Sports had better SRS-30 (Function, Self-image, and Total) scores, SAQ-Child Expectations, and SAQ-Parent Total Scores (P<0.05). Regression models revealed major Cobb angle (beta coefficient: -0.312) and sports participation (beta coefficient: 0.422) as significant predictors of SRS-30 Function score (R=0.434, P<0.05). Our data show that for AIS patients with statistically similar bracing status and coronal and sagittal deformities, patients who participated in sports were more likely to have improved functionality, self-image, expectations, and parental perception of deformity. Further investigation is warranted to acquire a comprehensive understanding of the relationship between AIS and patient participation in sports. Maintaining moderate levels of physical activity and participating in safe sports may benefit treatment outcomes. Level of Evidence III - Retrospective Comparative Study.

C1 [Segreto, Frank A.; Doran, James P.; Walker, Sarah E.; Aylyarov, Alexandr; Shah, Neil, V; Mixa, Patrick J.; Kaur, Harleen; Day, Louis M.; Naziri, Qais; Paulino, Carl B.; Hesham, Khalid; Urban, William P.; Diebo, Bassel G.] SUNY Downstate Med Ctr, Dept Orthopaed Surg, Brooklyn, NY 11203 USA.

[Messina, James C.; Paltoo, Karen; Opare-Sem, Kwaku] SUNY, Downstate Coll Med, Brooklyn, NY USA.

[Scott, Claude B.] Kings Cty Hosp Ctr, Dept Orthopaed Surg, Brooklyn, NY USA.

[Ahmed, Natasha] Saba Univ, Sch Med, Saba, Netherlands.

C3 State University of New York (SUNY) System; State University of New York

(SUNY) Downstate Medical Center; State University of New York (SUNY)

System; State University of New York (SUNY) Downstate Medical Center;

SUNY Maritime College

RP Diebo, BG (通讯作者)， SUNY Downstate Med Ctr, 450 Clarkson Ave,MSC 30, Brooklyn, NY 11203 USA.

EM dr.basseldiebo@gmail.com

RI Shah, Neil V./K-1839-2019; Naziri, Qais/AAE-1620-2022; Diebo, Bassel

G./G-8835-2015

OI Shah, Neil V./0000-0002-3439-3071; Naziri, Qais/0000-0001-9076-305X;

Diebo, Bassel G./0000-0002-7835-2263

CR Athanasopoulos S, 1999, SCAND J MED SCI SPOR, V9, P36

Auerbach Joshua D, 2014, J Bone Joint Surg Am, V96, pe61, DOI 10.2106/JBJS.L.00867

Glowacki M, 2013, J DEV PHYS DISABIL, V25, P203, DOI 10.1007/s10882-012-9296-y

Goodbody CM, 2017, J PEDIATR ORTHOPED, V37, P41, DOI 10.1097/BPO.0000000000000580

Kenanidis E, 2008, SPINE, V33, P2160, DOI 10.1097/BRS.0b013e31817d6db3

Koh JO, 2003, BRAIN INJURY, V17, P901, DOI 10.1080/0269905031000088869

Konieczny MR, 2013, J CHILD ORTHOP, V7, P3, DOI 10.1007/s11832-012-0457-4

Longworth B, 2014, ARCH PHYS MED REHAB, V95, P1725, DOI 10.1016/j.apmr.2014.02.027

MACLEAN WE, 1989, J PEDIATR ORTHOPED, V9, P257

Matamalas A, 2014, HEALTH QUAL LIFE OUT, V12, DOI 10.1186/1477-7525-12-81

Meyer C, 2006, SCAND J MED SCI SPOR, V16, P231, DOI 10.1111/j.1600-0838.2005.00482.x

Ng BKW, 2015, SCOLIOSIS SPINAL DIS, V10, DOI 10.1186/1748-7161-10-S2-S19

Paajanen H, 2011, ARCH ORTHOP TRAUM SU, V131, P261, DOI 10.1007/s00402-010-1169-1

Pellegrino LN, 2014, J SPINAL DISORD TECH, V27, P409, DOI 10.1097/BSD.0b013e3182797a5e

Raggio CL, 2006, ORTHOP CLIN N AM, V37, P555, DOI 10.1016/j.ocl.2006.09.010

Roberts DW, 2011, SPINE, V36, P53

Sanders JO, 2007, SPINE, V32, P2719, DOI 10.1097/BRS.0b013e31815a5959

Schwieger T, 2016, SPINE, V41, P311, DOI 10.1097/BRS.0000000000001210

Sperandio EF, 2014, SPINE J, V14, P2366, DOI 10.1016/j.spinee.2014.01.041

Watanabe K, 2017, J BONE JOINT SURG AM, V99, P284, DOI 10.2106/JBJS.16.00459

NR 20

TC 6

Z9 8

U1 1

U2 13

PU LIPPINCOTT WILLIAMS & WILKINS

PI PHILADELPHIA

PA TWO COMMERCE SQ, 2001 MARKET ST, PHILADELPHIA, PA 19103 USA

SN 1060-152X

EI 1473-5865

J9 J PEDIATR ORTHOP B

JI J. Pediatr. Orthop.-Part B

PD JUL

PY 2019

VL 28

IS 4

BP 356

EP 361

DI 10.1097/BPB.0000000000000574

PG 6

WC Orthopedics; Pediatrics

WE Science Citation Index Expanded (SCI-EXPANDED)

SC Orthopedics; Pediatrics

GA IG1EZ

UT WOS:000473534500010

PM 30489444

DA 2023-08-10

ER

PT J

AU Zapata, KA

Sucato, DJ

Jo, CH

AF Zapata, Karina A.

Sucato, Daniel J.

Jo, Chan-Hee

TI Physical Therapy Scoliosis-Specific Exercises May Reduce Curve

Progression in Mild Adolescent Idiopathic Scoliosis Curves

SO PEDIATRIC PHYSICAL THERAPY

LA English

DT Article

DE adolescent idiopathic scoliosis; spine deformity

ID QUESTIONNAIRE; RELIABILITY; VALIDITY; BRACE

AB Purpose: To evaluate the curve magnitude in participants with mild adolescent idiopathic scoliosis (AIS) at high risk of progression who received outpatient physical therapy scoliosis-specific exercises (PSSEs). Methods: Participants with AIS curves 12 degrees to 20 degrees and Risser grade 0 chose either the PSSE or the control group. The PSSE group was instructed in the Barcelona Scoliosis Physical Therapy School. The control group was observed. Cobb angles were measured by one observer masked to group type at baseline, 6-month follow-up, and 1-year follow-up. Results: Forty-nine participants were enrolled (26 exercise vs 23 controls). Thirty-three participants (19 exercise vs 14 controls) were seen at 1-year follow-up. At 1-year follow-up, the exercise group had smaller curves than controls (16.3 degrees vs 21.6 degrees, P = .04) and less curve progression (0 degrees vs 5.6 degrees, P = .02). Bracing was performed similarly between groups at 1-year follow-up (37% vs 43%). Conclusions: In this small prospective series, PSSE resulted in significantly less curve progression compared with controls.

C1 [Zapata, Karina A.; Sucato, Daniel J.; Jo, Chan-Hee] Texas Scottish Rite Hosp Children, 2222 Welborn St, Dallas, TX 75219 USA.

C3 Texas Scottish Rite Hospital for Children

RP Zapata, KA (通讯作者)，Texas Scottish Rite Hosp Children, 2222 Welborn St, Dallas, TX 75219 USA.

EM Karina.zapata@tsrh.org

CR Asher M, 2003, SPINE, V28, P63, DOI 10.1097/00007632-200301010-00015

Berdishevsky H, 2016, SCOLIOSIS SPINAL DIS, V11, DOI 10.1186/s13013-016-0076-9

BUNNELL WP, 1986, SPINE, V11, P773, DOI 10.1097/00007632-198610000-00003

Deviren V, 2002, SPINE, V27, P2346, DOI 10.1097/00007632-200211010-00007

Dimeglio A, 2011, J PEDIATR ORTHOPED, V31, pS28, DOI 10.1097/BPO.0b013e318202c25d

Fusco C., 2011, Physiotherapy Theory and Practice, V27, P80, DOI 10.3109/09593985.2010.533342

Grivas TB, 2008, STUD HEALTH TECHNOL, V140, P33, DOI 10.3233/978-1-58603-888-5-33

Herring JA, 2008, TACHDJIANS PEDIAT OR, V4th

Kuru T, 2016, CLIN REHABIL, V30, P181, DOI 10.1177/0269215515575745

Langensiepen S, 2013, EUR SPINE J, V22, P2360, DOI 10.1007/s00586-013-2693-9

Lehnert-Schroth C., 2007, 3 DIMENSIONAL TREATM, V7th ed.

Lenssinck MLB, 2005, PHYS THER, V85, P1329, DOI 10.1093/ptj/85.12.1329

Manring MM, 2009, CLIN ORTHOP RELAT R, V468, P643

Monticone M, 2014, EUR SPINE J, V23, P1204, DOI 10.1007/s00586-014-3241-y

Negrini S, 2008, J REHABIL MED, V40, P451, DOI 10.2340/16501977-0195

Negrini S, 2006, SCOLIOSIS SPINAL DIS, V1, DOI 10.1186/1748-7161-1-4

Negrini S, 2012, SCOLIOSIS SPINAL DIS, V7, DOI 10.1186/1748-7161-7-3

Richards BS, 2005, SPINE, V30, P2068, DOI 10.1097/01.brs.0000178819.90239.d0

Rigo M, 2009, STUD HEALTH TECHNOL, V135, P208

Romano M, 2012, COCHRANE DB SYST REV, DOI 10.1002/14651858.CD007837.pub2

Sanders JO, 2007, SPINE, V32, P2719, DOI 10.1097/BRS.0b013e31815a5959

Sanders JO, 1997, SPINE, V22, P1352, DOI 10.1097/00007632-199706150-00013

Schreiber S, 2016, PLOS ONE, V11, DOI 10.1371/journal.pone.0168746

Weinstein SL, 2008, LANCET, V371, P1527, DOI 10.1016/S0140-6736(08)60658-3

Weiss HR, 2011, SCOLIOSIS SPINAL DIS, V6, DOI 10.1186/1748-7161-6-17

Weiss HR, 2009, STUD HEALTH TECHNOL, V135, P173

Zapata KA, 2015, PEDIATR PHYS THER, V27, P119, DOI 10.1097/PEP.0000000000000131

NR 27

TC 16

Z9 17

U1 4

U2 25

PU LIPPINCOTT WILLIAMS & WILKINS

PI PHILADELPHIA

PA TWO COMMERCE SQ, 2001 MARKET ST, PHILADELPHIA, PA 19103 USA

SN 0898-5669

EI 1538-005X

J9 PEDIATR PHYS THER

JI Pediatr. Phys. Ther.

PD JUL

PY 2019

VL 31

IS 3

BP 280

EP 285

DI 10.1097/PEP.0000000000000621

PG 6

WC Pediatrics; Rehabilitation

WE Science Citation Index Expanded (SCI-EXPANDED)

SC Pediatrics; Rehabilitation

GA IH1KN

UT WOS:000474249800016

PM 31220013

OA Bronze

DA 2023-08-10

ER

PT J

AU Thompson, JY

Williamson, EM

Williams, MA

Heine, PJ

Lamb, SE

Crossman, RJ

Toye, F

Dritsaki, M

Petrou, S

Lall, R

Barker, K

Fairbank, J

Harding, I

Gardner, A

Kalyan, R

Slowther, A

Coulson, N

Bunce, S

Richards, A

Billing, L

Cockbain, E

Walker, H

Trees, A

AF Thompson, J. Y.

Williamson, E. M.

Williams, M. A.

Heine, P. J.

Lamb, S. E.

Crossman, R. J.

Toye, F.

Dritsaki, M.

Petrou, S.

Lall, R.

Barker, K.

Fairbank, J.

Harding, I

Gardner, A.

Kalyan, R.

Slowther, A.

Coulson, N.

Bunce, S.

Richards, A.

Billing, L.

Cockbain, E.

Walker, H.

Trees, A.

CA ACTIvATeS Study Grp

TI Effectiveness of scoliosis-specific exercises for adolescent idiopathic

scoliosis compared with other non-surgical interventions: a systematic

review and meta-analysis

SO PHYSIOTHERAPY

LA English

DT Review

DE Scoliosis; Adolescents; Exercise therapy; Review; Meta-analysis

ID THERAPY

AB Study design Systematic review and meta-analysis.

Objective To assess the effectiveness of scoliosis-specific exercises (SSE) on adolescent idiopathic scoliosis (AIS) compared with other non-surgical interventions.

Background AIS is a complex deformity of the spine that develops between the age of 10 years and skeletal maturity. SSE are prescribed to patients to reduce or slow curve progression, although their effectiveness is unknown.

Methods Electronic databases were searched for relevant studies. Randomised controlled trials were eligible if they compared SSE with nonsurgical interventions for individuals with AIS. Three authors independently extracted data, evaluated methodological quality and assessed the quality of evidence. Meta-analysis was performed where possible; otherwise, descriptive syntheses are reported.

Results Nine randomised controlled trials were included. Four had a high risk of bias, three had an unclear risk and two had a low risk. Very-low-quality evidence indicated that SSE improved some measures of spinal deformity, function, pain and overall health-related quality of life (HRQoL). Very-low-quality evidence suggested that SSE had no effect on self-image and mental health. Very-low-quality evidence showed that bracing was more effective than SSE on measures of spinal deformity. However, SSE showed greater improvements in function, HRQoL, self-image, mental health and patient satisfaction with treatment. No differences were found for pain or trunk rotation.

Conclusions SSE may be effective for improving measures of spinal deformity for people with AIS, but the evidence is of very low quality. Future studies should evaluate relevant clinical measures and cost-effectiveness using rigorous methods and reporting standards. (C) 2018 Chartered Society of Physiotherapy. Published by Elsevier Ltd. All rights reserved.

C1 [Thompson, J. Y.; Williamson, E. M.; Heine, P. J.; Lamb, S. E.] Univ Oxford, Nuffield Dept Orthopaed Rheumatol & Musculoskelet, Oxford, united kingdom.

[Williams, M. A.] Oxford Brookes Univ, Dept Sport & Hlth Sci, Oxford, united kingdom.

[Heine, P. J.; Lamb, S. E.] Univ Warwick, Warwick Clin Trials Unit, Coventry, W Midlands, united kingdom.

C3 University of Oxford; Oxford Brookes University; University of Warwick

RP Thompson, JY (通讯作者)， Univ Oxford, Nuffield Dept Orthopaed Rheumatol & Musculoskelet, Oxford, united kingdom.

EM jacqueline.thompson@ndorms.ox.ac.uk

RI Thompson, Jacqueline/I-5118-2019; Thompson, Jacqueline/AAO-4065-2021;

Fairbank, Jeremy/AAA-3411-2019; Jacqueline, Thompson/GPT-2977-2022;

Petrou, Stavros/ABD-8323-2021

OI Thompson, Jacqueline/0000-0002-9775-361X; Petrou,

Stavros/0000-0003-3121-6050; Williamson, Esther/0000-0003-0638-0406;

Lamb, Sarah/0000-0003-4349-7195; Gardner, Anna/0000-0002-4625-9769

FU National Institute for Health Research Health Technology Assessment

(NIHR HTA) -Active Treatment for Idiopathic Adolescent Scoliosis

(ACTIvATeS): a feasibility study [10/38/03]; NIHR Collaboration for

Leadership in Applied Health Research and Care Oxford at Oxford Health

NHS Foundation Trust; NIHR Oxford Musculoskeletal Biomedical Research

Unit

FX This project was funded by the National Institute for Health Research

Health Technology Assessment (NIHR HTA) -Active Treatment for Idiopathic

Adolescent Scoliosis (ACTIvATeS): a feasibility study (Project Number

10/38/03). The research was supported by the NIHR Collaboration for

Leadership in Applied Health Research and Care Oxford at Oxford Health

NHS Foundation Trust, and the NIHR Oxford Musculoskeletal Biomedical

Research Unit. The views expressed are those of the author(s) and not necessarily those of the HTA, NHS, NIHR or the Department of Health and Social Care.

CR Abbott A, 2013, BMC MUSCULOSKEL DIS, V14, DOI 10.1186/1471-2474-14-261

Alayat Mohamed Salaheldien Mohamed, 2017, J Phys Ther Sci, V29, P1658, DOI 10.1589/jpts.29.1658

[Anonymous], 2010, BMJ BRIT MED J

[Anonymous], CHIN J CLIN REHABIL

Bettany-Saltikov J, 2014, EUR J PHYS REHAB MED, V50, P111

Bettany-Saltikov J, 2015, COCHRANE DB SYST REV, V4

Dantas Diego De Sousa, 2017, J Phys Ther Sci, V29, P1, DOI 10.1589/jpts.29.1

Davies Evan, 2011, Evid Based Spine Care J, V2, P25, DOI 10.1055/s-0030-1267102

Diab AA, 2012, CLIN REHABIL, V26, P1123, DOI 10.1177/0269215512447085

Dolan LA, 2007, SPINE, V32, pS91, DOI 10.1097/BRS.0b013e318134ead9

Fusco C., 2011, Physiotherapy Theory and Practice, V27, P80, DOI 10.3109/09593985.2010.533342

Gur G, 2017, PROSTHET ORTHOT INT, V41, P303, DOI 10.1177/0309364616664151

Guyatt GH, 2011, J CLIN EPIDEMIOL, V64, P1294, DOI 10.1016/j.jclinepi.2011.03.017

Higgins J, 2012, COCHRANE DB SYST REV, DOI 10.1002/14651858.ED000049

Kim G, 2016, J PHYS THER SCI, V28, P1012, DOI 10.1589/jpts.28.1012

Lenssinck MLB, 2005, PHYS THER, V85, P1329, DOI 10.1093/ptj/85.12.1329

Maruyama Toru, 2011, Physiotherapy Theory and Practice, V27, P26, DOI 10.3109/09593985.2010.503989

Misterska E, 2018, PLOS ONE, V13, DOI 10.1371/journal.pone.0193447

Mordecai SC, 2012, EUR SPINE J, V21, P382, DOI 10.1007/s00586-011-2063-4

Negrini S, 2008, DISABIL REHABIL, V30, P772, DOI 10.1080/09638280801889568

Negrini S, 2014, SCOLIOSIS, V9, pO71

Negrini S, 2010, EVID BASED CHILD HLT, V5, P1681

Negrini S, 2012, Scoliosis, V7, P1

Negrini S, 2018, SCOLIOSIS SPINAL DIS, V13, DOI 10.1186/s13013-017-0145-8

Negrini S, 2016, SPINE, V41, P1813, DOI 10.1097/BRS.0000000000001887

Negrini S, 2015, SCOLIOSIS SPINAL DIS, V10, DOI 10.1186/s13013-014-0025-4

Rigo M, 2003, Pediatr Rehabil, V6, P209

Romano M, 2013, SPINE, V38, pE883, DOI 10.1097/BRS.0b013e31829459f8

Rubinstein SM, 2013, SPINE, V38, pE158, DOI 10.1097/BRS.0b013e31827dd89d

Sanders James O, 2012, J Pediatr Orthop, V32 Suppl 2, pS153, DOI 10.1097/BPO.0b013e31825199e5

Schreiber S, 2016, PLOS ONE, V11, DOI 10.1371/journal.pone.0168746

Schreiber S, 2015, SCOLIOSIS SPINAL DIS, V10, DOI 10.1186/s13013-015-0048-5

Schreiber Sanja, 2014, J Physiother, V60, P234, DOI 10.1016/j.jphys.2014.08.005

Toledo Pollyana Coelho Vieira, 2011, Fisioter. Pesqui., V18, P329

WEISS HR, 2008, SCOLIOSIS SPINAL DIS, V3

Zheng Y, 2018, SPINE, V43, pE494, DOI 10.1097/BRS.0000000000002412

[No title captured]

2017, EUR J PHYS REHABIL M, V53, P351

2003, SPINE, V28, P1290

2016, CLIN REHABIL, V30, P181

2014, EUR SPINE J, V23, P1204

2009, EUR SPINE J, V18, P1898

2010, SPINE, V35, P2079

NR 43

TC 21

Z9 25

U1 3

U2 48

PU ELSEVIER SCI LTD

PI OXFORD

PA THE BOULEVARD, LANGFORD LANE, KIDLINGTON, OXFORD OX5 1GB, OXON, united kingdom

SN 0031-9406

EI 1873-1465

J9 PHYSIOTHERAPY

JI Physiotherapy

PD JUN

PY 2019

VL 105

IS 2

BP 214

EP 234

DI 10.1016/j.physio.2018.10.004

PG 21

WC Rehabilitation

WE Science Citation Index Expanded (SCI-EXPANDED)

SC Rehabilitation

GA HX6VH

UT WOS:000467541000007

PM 30824243

OA Green Accepted

DA 2023-08-10

ER

PT J

AU Yagci, G

Yakut, Y

AF Yagci, Gozde

Yakut, Yavuz

TI Core stabilization exercises versus scoliosis-specific exercises in

moderate idiopathic scoliosis treatment

SO PROSTHETICS AND ORTHOTICS INTERNATIONAL

LA English

DT Article

DE Scoliosis; exercise; bracing

ID QUALITY-OF-LIFE; SPINAL DEFORMITY; RELIABILITY; VALIDITY; PATIENT; BRACE

AB Background: There are several kinds of scoliosis-specific and general physiotherapeutic exercise methods used in scoliosis rehabilitation. But there is need for comparable studies on the effectiveness of different exercise approaches for the treatment of adolescent idiopathic scoliosis. Objectives: Comparison of the effects of combined core stabilization exercise and bracing treatment with Scientific Exercises Approach to Scoliosis and bracing treatment in patients with moderate adolescent idiopathic scoliosis. Methods: Thirty females with adolescent idiopathic scoliosis, who have moderate curves (20 degrees-45 degrees), were randomly divided into two groups. In addition to brace wearing for 4 months, one group received core stabilization exercise therapy, while the other received scientific exercises approach to scoliosis exercise therapy. The outcome measures were based on Cobb angle, angle of trunk rotation, body symmetry, cosmetic trunk deformity, and quality of life. Results: Thoracic and lumbar Cobb angles and trunk rotation angles, body symmetry, and cosmetic trunk deformity improved for both groups. Quality of life did not change in either group. The pain domain of the Scoliosis Research Society-22 questionnaire improved in the core stabilization group only. Conclusion: Both treatment conditions including core stabilization with bracing and scientific exercises approach to scoliosis with bracing had similar effects in the short-term treatment of moderate adolescent idiopathic scoliosis.

C1 [Yagci, Gozde] Hacettepe Univ, Fac Hlth Sci, Sch Phys Therapy & Rehabil Sci, Orthot & Biomech Dept, Ankara, Turkey.

[Yakut, Yavuz] Hasan Kalyoncu Univ, Physiotherapy & Rehabil Dept, Gaziantep, Turkey.

C3 Hacettepe University; Hasan Kalyoncu University

RP Yagci, G (通讯作者)， Hacettepe Univ, Fac Hlth Sci, Sch Phys Therapy & Rehabil Sci, TR-06100 Ankara, Turkey.

EM gozdeygc8@gmail.com

RI YAGCI, GOZDE/J-1185-2013; YAKUT, YAVUZ/J-1174-2013

OI YAGCI, GOZDE/0000-0002-4603-7162;

CR Akuthota V, 2008, CURR SPORT MED REP, V7, P39, DOI 10.1097/01.CSMR.0000308663.13278.69

Alanay A, 2005, SPINE, V30, P2464, DOI 10.1097/01.brs.0000184366.71761.84

Altaf F, 2013, BMJ-BRIT MED J, V346, DOI 10.1136/bmj.f2508

de Araujo MEA, 2012, J BODYW MOV THER, V16, P191, DOI 10.1016/j.jbmt.2011.04.002

Asher M, 2003, SPINE, V28, P63, DOI 10.1097/00007632-200301010-00015

BUNNELL WP, 1984, J BONE JOINT SURG AM, V66A, P1381, DOI 10.2106/00004623-198466090-00010

Carreon LY, 2010, SPINE, V35, P2079, DOI 10.1097/BRS.0b013e3181c61fd7

Cobb JR., 1948, INSTR COURSE LECT, V5, P261

Coelho DM, 2013, BRAZ J PHYS THER, V17, P179, DOI 10.1590/S1413-35552012005000081

Fusco C., 2011, Physiotherapy Theory and Practice, V27, P80, DOI 10.3109/09593985.2010.533342

Gur G, 2017, PROSTHET ORTHOT INT, V41, P303, DOI 10.1177/0309364616664151

Gur G, 2015, GAIT POSTURE, V41, P93, DOI 10.1016/j.gaitpost.2014.09.001

Horne JP, 2014, AM FAM PHYSICIAN, V89, P193

Inami K., 1999, RES SPINAL DEFORMITI, V2, P85

Katz DE, 1997, SPINE, V22, P1302, DOI 10.1097/00007632-199706150-00005

Lonner B, 2013, SPINE, V38, P1049, DOI 10.1097/BRS.0b013e3182893c01

Negrini S, 2006, ST HEAL T, V123, P519

Negrini S, 2006, SCOLIOSIS SPINAL DIS, V1, DOI 10.1186/1748-7161-1-4

Negrini S, 2018, SCOLIOSIS SPINAL DIS, V13, DOI 10.1186/s13013-017-0145-8

Negrini S, 2012, SCOLIOSIS SPINAL DIS, V7, DOI 10.1186/1748-7161-7-3

Negrini S, 2014, EUR SPINE J, V23, P2218, DOI 10.1007/s00586-014-3464-y

Paszewski M, 2014, WORLD J, V2014

Pineda S, 2006, SCOLIOSIS SPINAL DIS, V1, DOI 10.1186/1748-7161-1-18

Risser J., 1957, CLIN ORTHOP RELAT R, V11, P111

Romano M., 2007, SCOLIOSIS, V2, pS8

Romano M, 2015, SCOLIOSIS SPINAL DIS, V10, DOI 10.1186/s13013-014-0027-2

Romano M, 2013, SPINE, V38, pE883, DOI 10.1097/BRS.0b013e31829459f8

Sanders JO, 2003, SPINE, V28, P2158, DOI 10.1097/01.BRS.0000084629.97042.0B

Smania N, 2008, DISABIL REHABIL, V30, P763, DOI 10.1080/17483100801921311

Tavernaro M, 2012, SCOLIOSIS SPINAL DIS, V7, DOI 10.1186/1748-7161-7-17

Vasseljen O, 2012, SPINE, V37, P1101, DOI 10.1097/BRS.0b013e318241377c

Weinstein SL, 2013, NEW ENGL J MED, V369, P1512, DOI 10.1056/NEJMoa1307337

Zaina F, 2009, SCOLIOSIS SPINAL DIS, V4, DOI 10.1186/1748-7161-4-8

[No title captured]

NR 34

TC 23

Z9 24

U1 3

U2 48

PU SAGE PUBLICATIONS LTD

PI LONDON

PA 1 OLIVERS YARD, 55 CITY ROAD, LONDON EC1Y 1SP, united kingdom

SN 0309-3646

EI 1746-1553

J9 PROSTHET ORTHOT INT

JI Prosthet. Orthot. Int.

PD JUN

PY 2019

VL 43

IS 3

BP 301

EP 308

DI 10.1177/0309364618820144

PG 8

WC Orthopedics; Rehabilitation

WE Science Citation Index Expanded (SCI-EXPANDED)

SC Orthopedics; Rehabilitation

GA IG0OH

UT WOS:000473489500008

PM 30628526

DA 2023-08-10

ER

PT J

AU Wong, AYL

Samartzis, D

Cheung, PWH

Cheung, JPY

AF Wong, Arnold Y. L.

Samartzis, Dino

Cheung, Prudence W. H.

Cheung, Jason Pui Yin

TI How Common Is Back Pain and What Biopsychosocial Factors Are Associated

With Back Pain in Patients With Adolescent Idiopathic Scoliosis?

SO CLINICAL ORTHOPAEDICS AND RELATED RESEARCH

LA English

DT Article

ID INSOMNIA SEVERITY INDEX; CONCURRENT VALIDITY; PROSPECTIVE COHORT;

WIDESPREAD PAIN; RISK-FACTORS; FOLLOW-UP; CHILDREN; RELIABILITY;

PREVALENCE; SLEEP

AB Background Adolescent idiopathic scoliosis (AIS) is the most common spine deformity in adolescent patients. Although structural deformity may affect spinal biomechanics of patients with AIS, little is known regarding various period prevalence proportions of back pain and chronic back pain and factors associated with back pain in such patients.

Questions/purposes (1) What are the period prevalence rates of back pain among teenagers with AIS? (2) Is back pain in patients with AIS associated with curve severity? Methods A total of 987 patients with AIS who were treated without surgery were recruited from a single center's scoliosis clinic. Between December 2016 and July 2017, this center treated 1116 patients with suspected AIS. During that time, patients were offered surgery when their Cobb angle was at least 50 degrees and had evidence of curve progression between two visits, and most of the patients who were offered surgery underwent it; other patients with AIS were managed nonsurgically with regular observation, brace prescription, posture training, and reassurance. To be included in this prospective, cross-sectional study, a patient needed to be aged between 10 and 18 years with a Cobb angle > 10 degrees. No followup data were required. A total of 1097 patients with AIS were managed nonsurgically (98.3% of the group seen during the period in question). After obtaining parental consent, patients provided data related to their demographics; physical activity levels; lifetime, 12-month, 30-day, 7-day, and current thoracic pain and low back pain (LBP); chronic back pain (thoracic pain/LBP); brace use; and treatments for scoliosis/back pain. Pain was rated on a 10-point numeric rating scale for pain. The Insomnia Severity Index, Epworth Sleepiness Scale, and Depression Anxiety Stress Scales were also assessed. These features and radiologic study parameters between patients with and without back pain were also compared. Factors associated with current and 12-month back pain as well as chronic back pain were analyzed by multivariate analyses.

Results Depending on the types of period prevalence, the prevalence of thoracic pain ranged from 6% (55 of 987) within 12 months to 14% (139 of 987) within 7 days, whereas that of LBP ranged from 6% (54 of 987) to 29% (289 of 987). Specifically, chronic thoracic pain or LBP had the lowest prevalence. Compared with the no pain group, patients with current back pain had more severe insomnia (odds ratio [OR], 1.80; p = 0.02; 95% confidence interval [CI], 1.10-2.93) and daytime

sleepiness (OR, 2.41; p < 0.001, 95% CI, 1.43-4.07). Those with chronic back pain had the same problems along with moderate depression (OR, 2.49; p = 0.03; 95% CI, 1.08-5.71). Older age (OR range, 1.17-1.42; all p values <= 0.030) and Cobb angle > 40 degrees (OR range, 2.38-3.74; all p values <= 0.015), daytime sleepiness (OR range, 2.39-2.41; all p values <= 0.011), and insomnia (OR range, 1.76-2.31; all p values <= 0.001) were associated with episodic and/or chronic back pain. Females were more likely to experience back pain in the last 12 months than males. Moderate depression (OR, 3.29; 1.45-7.47; p = 0.004) and wearing a brace (OR, 3.00; 1.47-6.15; p = 0.003) were independently associated with chronic back pain.

Conclusions Biopsychosocial factors are associated with the presence and severity of back pain in the AIS population. Our results highlight the importance of considering back pain screening/management for patients with AIS with their psychosocial profile in addition to curve magnitude monitoring. In particular, sleep quality should be routinely assessed. Longitudinal changes and effects of psychotherapy should be determined in future studies. Level of Evidence Level II, prognostic study.

C1 [Wong, Arnold Y. L.] Hong Kong Polytech Univ, Dept Rehabil Sci, Hong Kong, china.

[Samartzis, Dino] Rush Univ, Med Ctr, Dept Orthopaed Surg, Chicago, IL 60612 USA.

[Cheung, Prudence W. H.; Cheung, Jason Pui Yin] Univ Hong Kong, Dept Orthopaed & Traumatol, Hong Kong, china.

[Wong, Arnold Y. L.] Hong Kong Polytech Univ, Dept Rehabil Sci, Hung Hom, ST512,5-F Ng Wing Hong Bldg, Hong Kong, china.

C3 Hong Kong Polytechnic University; Rush University; University of Hong

Kong; Hong Kong Polytechnic University

RP Cheung, JPY (通讯作者)， Univ Hong Kong, Dept Orthopaed & Traumatol, Hong Kong, china.;

Wong, AYL (通讯作者)， Hong Kong Polytech Univ, Dept Rehabil Sci, Hung Hom, ST512,5-F Ng Wing Hong Bldg, Hong Kong, china.

EM arnold.wong@polyu.edu.hk; cheungjp@hku.hk

RI Wong, Arnold YL/AAG-6682-2021; Cheung, Jason Pui Yin/AAJ-2016-2020;

Samartzis, Dino/F-3359-2010; Cheung, Jason Pui Yin/J-2214-2012

OI Wong, Arnold YL/0000-0002-5911-5756; Cheung, Jason Pui

Yin/0000-0002-7052-0875; Cheung, Prudence/0000-0002-3213-7373;

Samartzis, Dino/0000-0002-7473-1311

FU General Research Fund of the Research Grants Council [17156416]

FX The authors received a grant from the General Research Fund of the

Research Grants Council reference #17156416 (JPYC).

CR [Anonymous], SPINE PHILA PA 1976

[Anonymous], NEUROSURGERY

[Anonymous], BMC PEDIAT

Asher M, 2003, SPINE, V28, P74, DOI 10.1097/00007632-200301010-00017

Asher M, 2003, SPINE, V28, P63, DOI 10.1097/00007632-200301010-00015

Auvinen JP, 2010, EUR SPINE J, V19, P641, DOI 10.1007/s00586-009-1215-2

BALAGUE F, 1995, SPINE, V20, P1265, DOI 10.1097/00007632-199506000-00012

Brattberg G, 2004, EUR J PAIN, V8, P187, DOI 10.1016/j.ejpain.2003.08.001

Burton AK, 2005, BEST PRACT RES CL RH, V19, P541, DOI 10.1016/j.berh.2005.03.001

Cheung KMC, 2007, SPINE, V32, P1141, DOI 10.1097/01.brs.0000261562.48888.e3

Childs JD, 2005, SPINE, V30, P1331, DOI 10.1097/01.brs.0000164099.92112.29

Chung KF, 2011, SLEEP MED, V12, P463, DOI 10.1016/j.sleep.2010.09.019

Clark EM, 2016, SPINE, V41, pE611, DOI 10.1097/BRS.0000000000001330

DICKSON JH, 1990, J BONE JOINT SURG AM, V72A, P678, DOI 10.2106/00004623-199072050-00006

EICH E, 1985, PAIN, V23, P375, DOI 10.1016/0304-3959(85)90007-7

Eyvazov K, 2017, BMC MUSCULOSKEL DIS, V18, DOI 10.1186/s12891-017-1423-6

Fong DYT, 2015, SPINE J, V15, P825, DOI 10.1016/j.spinee.2015.01.019

Gendreau M, 2003, BEST PRACT RES CL RH, V17, P575, DOI 10.1016/S1521-6942(03)00031-7

Girardo M, 2011, EUR SPINE J, V20, P68, DOI 10.1007/s00586-011-1750-5

GOODMAN JE, 1991, PAIN, V46, P247, DOI 10.1016/0304-3959(91)90108-A

HARREBY M, 1995, SPINE, V20, P2298, DOI 10.1097/00007632-199511000-00007

JAMISON RN, 1989, PAIN, V37, P289, DOI 10.1016/0304-3959(89)90193-0

Jeffries LJ, 2007, SPINE, V32, P2630, DOI 10.1097/BRS.0b013e318158d70b

Joncas J, 1996, ANN CHIR, V50, P637

Kassebaum NJ, 2016, LANCET, V388, P1603, DOI 10.1016/S0140-6736(16)31460-X

Lenke LG, 2001, J BONE JOINT SURG AM, V83A, P1169, DOI 10.2106/00004623-200108000-00006

Makino T, 2015, SPRINGERPLUS, V4, DOI 10.1186/s40064-015-1189-y

Mikkelsson M, 2008, PAIN, V138, P681, DOI 10.1016/j.pain.2008.06.005

Milanese Steven, 2010, J Pain Res, V3, P57

Le MTH, 2017, PLOS ONE, V12, DOI 10.1371/journal.pone.0180557

NASH CL, 1969, J BONE JOINT SURG AM, VA 51, P223, DOI 10.2106/00004623-196951020-00002

Negrini S, 2015, SCOLIOSIS SPINAL DIS, V10, DOI 10.1186/s13013-014-0025-4

O'Sullivan P, 2011, PEDIATR RHEUMATOL, V9, DOI 10.1186/1546-0096-9-3

O'Sullivan PB, 2012, BMC PUBLIC HEALTH, V12, DOI 10.1186/1471-2458-12-100

Osman A, 2012, J CLIN PSYCHOL, V68, P1322, DOI 10.1002/jclp.21908

Pakpour AH, 2018, PAIN PRACT, V18, P79, DOI 10.1111/papr.12584

Pratt RK, 2002, SPINE, V27, P1543, DOI 10.1097/00007632-200207150-00012

Ramirez N, 1997, J BONE JOINT SURG AM, V79A, P364, DOI 10.2106/00004623-199703000-00007

Sato T, 2011, EUR SPINE J, V20, P274, DOI 10.1007/s00586-010-1657-6

Smorgick Y, 2013, J PEDIATR ORTHOPED, V33, P289, DOI 10.1097/BPO.0b013e31827d0b43

Theroux J, 2017, SPINE, V42, pE914, DOI 10.1097/BRS.0000000000001986

Theroux J, 2017, CHIROPR MAN THER, V25, DOI 10.1186/s12998-017-0143-1

Theroux J, 2015, PAIN RES MANAG, V20, P153, DOI 10.1155/2015/674354

Tully PJ, 2009, J ABNORM CHILD PSYCH, V37, P717, DOI 10.1007/s10802-009-9306-4

WEINSTEIN SL, 1981, J BONE JOINT SURG AM, V63, P702, DOI 10.2106/00004623-198163050-00003

Weinstein SL, 2008, LANCET, V371, P1527, DOI 10.1016/S0140-6736(08)60658-3

Wong AYL, 2016, CLIN BIOMECH, V34, P45, DOI 10.1016/j.clinbiomech.2016.03.006

Yu DSF, 2010, J ADV NURS, V66, P2350, DOI 10.1111/j.1365-2648.2010.05394.x

NR 48

TC 25

Z9 25

U1 3

U2 15

PU LIPPINCOTT WILLIAMS & WILKINS

PI PHILADELPHIA

PA TWO COMMERCE SQ, 2001 MARKET ST, PHILADELPHIA, PA 19103 USA

SN 0009-921X

EI 1528-1132

J9 CLIN ORTHOP RELAT R

JI Clin. Orthop. Rel. Res.

PD APR

PY 2019

VL 477

IS 4

BP 676

EP 686

DI 10.1097/CORR.0000000000000569

PG 11

WC Orthopedics; Surgery

WE Science Citation Index Expanded (SCI-EXPANDED); Social Science Citation Index (SSCI)

SC Orthopedics; Surgery

GA IE8FI

UT WOS:000472608900003

PM 30516661

OA Green Published, Bronze

DA 2023-08-10

ER

PT J

AU Negrini, S

Donzelli, S

Negrini, A

Parzini, S

Romano, M

Zaina, F

AF Negrini, Stefano

Donzelli, Sabrina

Negrini, Alessandra

Parzini, Silvana

Romano, Michele

Zaina, Fabio

TI Specific exercises reduce the need for bracing in adolescents with

idiopathic scoliosis: A practical clinical trial

SO ANNALS OF PHYSICAL AND REHABILITATION MEDICINE

LA English

DT Article

DE Scoliosis; Adolescents; Exercise

ID QUALITY-OF-LIFE; RISSER SIGN; PROGRESSION; CURVE; GIRLS; EFFICACY

AB Background: In an ideal experimental setting, 2 randomized controlled trials recently showed the efficacy of physiotherapeutic scoliosis-specific exercises (PSSEs) for adolescents with idiopathic scoliosis (AIS). Now large observational studies are needed to check the generalizability of these results to everyday clinical life.

Objective: To explore the effectiveness of PSSEs for avoiding bracing or progression of AIS in everyday clinics.

Methods: This was a longitudinal comparative observational multicenter study, nested in a prospective database of outpatient tertiary referral clinics, including 327 consecutive patients. Inclusion criteria were AIS, age >= 10 years old at first evaluation, Risser sign 0-2, and 11-20 degrees Cobb angle. Exclusion criteria were consultations only and brace prescription at baseline. Groups performed PSSE according to the SEAS (Scientific Exercise Approach to Scoliosis) School, usual physiotherapy (UP) and no therapy (controls [CON]). End of treatment was medical discharge, Risser sign 3, or failure (defined by the need for bracing before the end of growth or Cobb angle > 29 degrees). The probability of failure was estimated by the risk ratio (RR) and 95% confidence interval (CI). The number needed to treat was estimated. Statistical analysis included intent-to-treat analysis, considering all participants (dropouts as failures), and efficacy analysis, considering only end-of-treatment participants. Propensity scores were used to reduce the potential effects of confounders related to the observational design.

Results: We included 293 eligible subjects after propensity score matching (SEAS, n = 145; UP, n = 95; controls, n = 53). The risk of success was increased 1.7-fold (P = 0.007) and 1.5-fold (P = 0.006) with SEAS versus controls in the efficacy and intent-to-treat analyses, respectively, and the number needed to treat for testing SEAS versus controls was 3.5 (95% CI 3.2-3.7) and 1.8 (95% CI 1.5-2.0), respectively. The success rate was higher with SEAS than UP in the efficacy analysis.

Conclusions: SEAS reduced the bracing rate in AIS and was more effective than UP. PSSEs are additional tools that can be included in the therapeutic toolbox for AIS treatment. (C) 2018 Elsevier Masson SAS. All rights reserved.

C1 [Negrini, Stefano] Univ Brescia, Clin & Expt Sci Dept, I-25121 Brescia, Italy.

[Negrini, Stefano] IRCCS Fdn Don Carlo Gnocchi, Milan, Italy.

[Donzelli, Sabrina; Negrini, Alessandra; Parzini, Silvana; Romano, Michele; Zaina, Fabio] ISICO Italian Sci Spine Inst, Via Roberto Bellarmino 13-1, I-20141 Milan, Italy.

C3 University of Brescia; IRCCS Fondazione Don Carlo Gnocchi Onlus

RP Negrini, S (通讯作者)， Univ Brescia, Clin & Expt Sci Dept, I-25121 Brescia, Italy.

EM stefano.negrini@unibs.it

RI Negrini, Stefano/B-6667-2013; Donzelli, Sabrina/IQU-0779-2023; Zaina,

Fabio/H-3261-2013

OI Negrini, Stefano/0000-0002-1878-2747; Zaina, Fabio/0000-0002-1256-5362

CR Aulisa AG, 2010, SCOLIOSIS SPINAL DIS, V5, DOI 10.1186/1748-7161-5-21

Aulisa AG, 2009, SCOLIOSIS SPINAL DIS, V4, DOI 10.1186/1748-7161-4-21

Aulisa AG, 2018, SPINE, V43, P114, DOI 10.1097/BRS.0b013e3181ee77f9

Black N, 1996, BRIT MED J, V312, P1215

BUNNELL WP, 1984, J BONE JOINT SURG AM, V66A, P1381, DOI 10.2106/00004623-198466090-00010

Climent JM, 1999, SPINE, V24, P1903, DOI 10.1097/00007632-199909150-00007

Di Felice F, 2018, AM J PHYS MED REHAB, V97, P346, DOI 10.1097/PHM.0000000000000861

Dolan LA, 2007, SPINE, V32, pS91, DOI 10.1097/BRS.0b013e318134ead9

Guo J, 2014, EUR SPINE J, V23, P2650, DOI 10.1007/s00586-013-3146-1

Hresko MT, 2013, NEW ENGL J MED, V368, P834, DOI 10.1056/NEJMcp1209063

Knott P, 2014, SCOLIOSIS SPINAL DIS, V9, DOI 10.1186/1748-7161-9-4

Kotwicki T, 2009, SCOLIOSIS SPINAL DIS, V4, DOI 10.1186/1748-7161-4-26

Kotwicki T, 2008, EUR SPINE J, V17, P1676, DOI 10.1007/s00586-008-0794-7

Kuru T, 2016, CLIN REHABIL, V30, P181, DOI 10.1177/0269215515575745

Lee CF, 2012, SPINE J, V12, P989, DOI 10.1016/j.spinee.2012.05.009

LONSTEIN JE, 1994, LANCET, V344, P1407

Malmivaara A, 2015, ANN MED, V47, P332, DOI 10.3109/07853890.2015.1027255

Meyer C, 2008, SCAND J MED SCI SPOR, V18, P751, DOI 10.1111/j.1600-0838.2007.00750.x

Monticone M, 2008, EUR J PHYS REHAB MED, V44, P467

Monticone M, 2014, EUR SPINE J, V23, P1204, DOI 10.1007/s00586-014-3241-y

NACHEMSON AL, 1995, J BONE JOINT SURG AM, V77A, P815, DOI 10.2106/00004623-199506000-00001

Negrini S, 2014, EUR J PHYS REHAB MED, V50, P83

Negrini S, 2014, EUR SPINE J, DOI [10.1007/s00586-014-3241-y, DOI 10.1007/S00586-014-3241-Y]

Negrini S, 2012, SCOLIOSIS S, V7, pO16, DOI DOI 10.1186/1748-7161-7-S1-016

Negrini S, 2008, J REHABIL MED, V40, P451, DOI 10.2340/16501977-0195

Negrini S, 2006, ST HEAL T, V123, P523

Negrini S, 2018, SCOLIOSIS SPINAL DIS, V13, DOI 10.1186/s13013-017-0145-8

Negrini S, 2015, COCHRANE DB SYST REV, DOI 10.1002/14651858.CD006850.pub3

Negrini S, 2015, SCOLIOSIS SPINAL DIS, V10, DOI 10.1186/s13013-014-0025-4

PETERSON LE, 1995, J BONE JOINT SURG AM, V77A, P823, DOI 10.2106/00004623-199506000-00002

Richards BS, 2005, SPINE, V30, P2068, DOI 10.1097/01.brs.0000178819.90239.d0

Romano M, 2015, SCOLIOSIS SPINAL DIS, V10, DOI 10.1186/s13013-014-0027-2

Romano M, 2012, COCHRANE DB SYST REV, DOI 10.1002/14651858.CD007837.pub2

Schreiber S, 2016, PLOS ONE, V11, DOI 10.1371/journal.pone.0168746

Schreiber S, 2015, SCOLIOSIS SPINAL DIS, V10, DOI 10.1186/s13013-015-0048-5

Wang WWJ, 2009, SPINE, V34, P1849, DOI 10.1097/BRS.0b013e3181ab358c

Weinstein SL, 2008, LANCET, V371, P1527, DOI 10.1016/S0140-6736(08)60658-3

Weinstein SL, 2013, SPINE, V38, P1832, DOI 10.1097/01.brs.0000435048.23726.3e

Weinstein SL, 2013, NEW ENGL J MED, V369, P1512, DOI 10.1056/NEJMoa1307337

Zaina F, 2009, SCOLIOSIS SPINAL DIS, V4, DOI 10.1186/1748-7161-4-3

NR 40

TC 21

Z9 23

U1 3

U2 26

PU ELSEVIER FRANCE-EDITIONS SCIENTIFIQUES MEDICALES ELSEVIER

PI ISSY-LES-MOULINEAUX

PA 65 RUE CAMILLE DESMOULINS, CS50083, 92442 ISSY-LES-MOULINEAUX, FRANCE

SN 1877-0657

EI 1877-0665

J9 ANN PHYS REHABIL MED

JI Ann. Phys. Rehabil. Med.

PD MAR

PY 2019

VL 62

IS 2

BP 69

EP 76

DI 10.1016/j.rehab.2018.07.010

PG 8

WC Rehabilitation

WE Science Citation Index Expanded (SCI-EXPANDED)

SC Rehabilitation

GA HN1KF

UT WOS:000459945200001

PM 30145241

OA Green Published

DA 2023-08-10

ER

PT J

AU Wibmer, C

Trotsenko, P

Gilg, MM

Leithner, A

Sperl, M

Saraph, V

AF Wibmer, Christine

Trotsenko, Pawel

Gilg, Magdalena M.

Leithner, Andreas

Sperl, Matthias

Saraph, Vinay

TI Observational retrospective study on socio-economic and quality of life outcomes in 41 patients with adolescent idiopathic scoliosis 5years after bracing combined with physiotherapeutic scoliosis-specific exercises (PSSE)

SO EUROPEAN SPINE JOURNAL

LA English

DT Article

DE Adolescent idiopathic scoliosis; Conservative treatment; Full time rigid bracing; Physiotherapeutic scoliosis-specific exercises; Quality of life questionnaire

ID FOLLOW-UP; INTRAOBSERVER; INTEROBSERVER

AB PurposeWe analysed socio-economic- and health-related quality of life (primary outcome) and radiological outcome (secondary outcome) 5years after full time rigid bracing (FTRB) plus physiotherapeutic scoliosis-specific exercises (PSSE) in adolescent idiopathic scoliosis. MethodsWe included 41 patients (38 female and 3 male) treated with FTRB (Chenau brace) and PSSE (Schroth) between 2001 and 2010. The study protocol included a clinical and radiological examination, the completion of the SRS-24 questionnaire and acquisition of data on socio-economic factors (educational and employment status) and physical activity. Descriptive statistics were used to analyse the data; differences between patients with mild (<30 degrees) and severe curves (30 degrees-50 degrees) regarding hrQoL outcomes were analysed using the Mann-Whitney U Test.ResultsPrimary outcomes showed full employment (higher education, 39%, and full-time work, 61%) and the

majority of the patients (79.9%) performing moderate sports two to four times per week. The SRS-24 questionnaire produced a total mean score (MS) of 4.0 (79.9%). Patients with mild curves had a significantly better total score (MS 4.1 vs. MS 3.8, p=0.020) and were more satisfied with the treatment (MS 4.3 vs MS 3.9, p=0.020). As to the secondary outcomes, from start of bracing to follow-up (a mean of 6.7years after brace weaning), the average Cobb angle changed from 28.6 degrees to 25.6 degrees (range 4 degrees-48 degrees); in 51.2% the curve angles could be maintained after weaning.ConclusionsCompared with the literature, our results showed similar curve development and hrQoL (pain, self-image and function) and better results regarding satisfaction with treatment. In comparison with the average population, there were no disadvantages as to occupation, education and sports.

[GRAPHICS]

.

C1 [Wibmer, Christine; Gilg, Magdalena M.; Leithner, Andreas] Med Univ Graz, Dept Orthoped Surg & Trauma, Auenbruggerpl 5, A-8036 Graz, Austria.

[Trotsenko, Pawel; Sperl, Matthias; Saraph, Vinay] Med Univ Graz, Dept Pediat Surg, Pediat Orthoped Unit, Auenbruggerpl 34, A-8036 Graz, Austria.

C3 Medical University of Graz; Medical University of Graz

RP Wibmer, C (通讯作者)， Med Univ Graz, Dept Orthoped Surg & Trauma, Auenbruggerpl 5, A-8036 Graz, Austria.

EM Christine.wibmer@medunigraz.at

RI Saraph, Vinay/GOE-5365-2022; Gilg, Magdalena/AAW-1931-2021

OI Gilg, Magdalena/0000-0002-2084-250X; Trotsenko,

Pawel/0000-0003-2851-4292

FU Medical University of Graz

FX Open access funding provided by Medical University of Graz. Many thanks

to Eugenia Lamont for language editing and to Milan Wibmer for his

patient and continuous support in data analysis and preparation of the

manuscript.

CR Anwer S, 2015, BIOMED RES INT-UK, V2015, DOI 10.1155/2015/123848

CARMAN DL, 1990, J BONE JOINT SURG AM, V72A, P328, DOI 10.2106/00004623-199072030-00003

Cobb J., 1948, INSTRUCTIONAL COURSE, V5, P261

Colaka TK, 2017, J BACK MUSCULOSKELET, V30, P597, DOI 10.3233/BMR-160564

Danielsson AJ, 2007, SPINE, V32, P2198, DOI 10.1097/BRS.0b013e31814b851f

Danielsson AJ, 2010, SPINE, V35, P199, DOI 10.1097/BRS.0b013e3181c89f4a

Danielsson AJ, 2001, EUR SPINE J, V10, P278, DOI 10.1007/s005860100309

Danielsson AJ, 2003, SPINE, V28, P2078

Gstoettner M, 2007, EUR SPINE J, V16, P1587, DOI 10.1007/s00586-007-0401-3

Haher TR, 1999, SPINE, V24, P1435, DOI 10.1097/00007632-199907150-00008

Klimont J, AUSTRIAN HLTH INTERV

Kuru T, 2016, CLIN REHABIL, V30, P181, DOI 10.1177/0269215515575745

Lange JE, 2011, SCOLIOSIS SPINAL DIS, V6, DOI 10.1186/1748-7161-6-18

Lange JE, 2009, SCOLIOSIS SPINAL DIS, V4, DOI 10.1186/1748-7161-4-17

Lehnert-Schroth C, 2014, DREIDIMENSIONALE SKO

Lou EHM, 2016, EUR SPINE J, V25, P495, DOI 10.1007/s00586-015-4233-2

Meng ZD, 2017, MEDICINE, V96, DOI 10.1097/MD.0000000000006828

Negrini S, 2014, EUR J PHYS REHAB MED, V50, P87

Negrini S, 2012, SCOLIOSIS SPINAL DIS, V7, DOI 10.1186/1748-7161-7-3

Negrini S, 2015, COCHRANE DB SYST REV, DOI 10.1002/14651858.CD006850.pub3

Negrini S, 2014, BMC MUSCULOSKEL DIS, V15, DOI 10.1186/1471-2474-15-263

Negrini S, 2010, SPINE, V35, P1285, DOI 10.1097/BRS.0b013e3181dc48f4

Parsch D, 2002, CLIN J SPORT MED, V12, P95, DOI 10.1097/00042752-200203000-00005

Richards BS, 2005, SPINE, V30, P2068, DOI 10.1097/01.brs.0000178819.90239.d0

Schreiber S, 2015, SCOLIOSIS SPINAL DIS, V10, DOI 10.1186/s13013-015-0048-5

Schwieger T, 2017, J PEDIATR ORTHOPED, V37, pE519, DOI 10.1097/BPO.0000000000000734

Scoliosis Research Society, 2015, SCOL RES SOC SCOL PA

Simony A, 2015, SCOLIOSIS SPINAL DIS, V10, DOI 10.1186/s13013-015-0045-8

Weinstein SL, 2013, SPINE, V38, P1832, DOI 10.1097/01.brs.0000435048.23726.3e

Weinstein SL, 2013, NEW ENGL J MED, V369, P1512, DOI 10.1056/NEJMoa1307337

Zaborowska-Sapeta K, 2011, SCOLIOSIS SPINAL DIS, V6, DOI 10.1186/1748-7161-6-2

NR 31

TC 3

Z9 3

U1 0

U2 16

PU SPRINGER

PI NEW YORK

PA 233 SPRING ST, NEW YORK, NY 10013 USA

SN 0940-6719

EI 1432-0932

J9 EUR SPINE J

JI Eur. Spine J.

PD MAR

PY 2019

VL 28

IS 3

BP 611

EP 618

DI 10.1007/s00586-018-5746-2

PG 8

WC Clinical Neurology; Orthopedics

WE Science Citation Index Expanded (SCI-EXPANDED)

SC Neurosciences & Neurology; Orthopedics

GA HO9MY

UT WOS:000461290800017

PM 30145658

OA hybrid

DA 2023-08-10

ER

PT J

AU Ghanem, I

Rizkallah, M

AF Ghanem, Ismat

Rizkallah, Maroun

TI Adolescent idiopathic scoliosis for the primary care physician:

frequently asked questions

SO CURRENT OPINION IN PEDIATRICS

LA English

DT Review

DE adolescent idiopathic scoliosis; exercising; physical activity;

schoolbag; women heath

ID QUALITY-OF-LIFE; BACK-PAIN; FOLLOW-UP; BACKPACKS; WEIGHT; WOMEN; GAIT;

PREGNANCY; BRACE; PREVALENCE

AB Purpose of review

Provide primary care physicians with the best available evidence to support answers to frequently asked questions by caregivers of patients with adolescent idiopathic scoliosis (AIS).

Recent findings

A review of best available evidence shows that schoolbag design and weight are not associated with higher odds of developing AIS. However, patients with AIS are more prone to balance problems with asymmetric backpack carrying and with rising weights. In patients with AIS, the backpack should be worn in a symmetric way and should never exceed 10% of the child's body weight. Although no relationship was found between systematic exercising in general and development of AIS, classical ballet and professional swimming in skeletally immature adolescents were associated to higher odds of having AIS. Since AIS affects adolescent girls mainly, women health issues are of paramount importance in this disease. Except for limited sexual activity and higher need for infertility treatment, patients with AIS perform similar to controls in marriage rates, age at first pregnancy, gestational age, offspring, labor and delivery. Best available evidence shows that adults with AIS diagnosed during adolescence have similar physical activity level compared with controls. In patients with AIS, adults with surgically treated idiopathic scoliosis have slightly lower physical activity level than previously braced and untreated patients.

Summary

Although schoolbag weight isn't associated with higher odds of developing AIS, classical ballet and professional swimming in adolescents are. Except for limited sexual activity, patients with AIS perform similar to controls in remaining women health issues. Adults with AIS diagnosed during adolescence have similar physical activity level compared with controls.

C1 [Ghanem, Ismat; Rizkallah, Maroun] St Joseph Univ, Hotel Dieu France Hosp, Orthoped Surg Dept, Beirut, Lebanon.

RP Ghanem, I (通讯作者)， St Joseph Univ, Hotel Dieu France Hosp, Orthoped Surg Dept, Beirut, Lebanon.

EM ismat.ghanem@gmail.com

CR Akazawa T, 2012, SPINE, V37, P1899, DOI 10.1097/BRS.0b013e31825a22c2

Al-Othman AA, 2017, ASIAN SPINE J, V11, P167, DOI 10.4184/asj.2017.11.2.167

Bauchat JR, 2015, ANESTH ANALG, V121, P981, DOI 10.1213/ANE.0000000000000690

BECKER TJ, 1986, CLIN SPORT MED, V5, P149

BETZ RR, 1987, J BONE JOINT SURG AM, V69A, P90, DOI 10.2106/00004623-198769010-00015

Castelein RM, 2005, MED HYPOTHESES, V65, P501, DOI 10.1016/j.mehy.2005.03.025

Chan P, 2017, SPINE, V42, pE1245, DOI 10.1097/BRS.0000000000002147

CHENG JC, 2015, NAT REV DIS PRIMERS, V1, DOI DOI 10.1038/NRDP.2015.30

Cottalorda J, 2004, REV CHIR ORTHOP, V90, P207

Cottalorda J, 2003, J PEDIATR ORTHOP B, V12, P357, DOI 10.1097/00009957-200311000-00001

Danielsson AJ, 2010, SPINE, V35, P199, DOI 10.1097/BRS.0b013e3181c89f4a

Danielsson AJ, 2001, EUR SPINE J, V10, P278, DOI 10.1007/s005860100309

Danielsson AJ, 2001, SPINE, V26, P1449, DOI 10.1097/00007632-200107010-00015

Dewan MC, 2018, EUR SPINE J, V27, P253, DOI 10.1007/s00586-017-5203-7

Diarbakerli E, 2018, J BONE JOINT SURG AM, V100, P811, DOI 10.2106/JBJS.17.00822

Diarbakerli E, 2017, SPINE, V42, pE404, DOI 10.1097/BRS.0000000000001841

Dockrell S, 2013, J SCHOOL HEALTH, V83, P368, DOI 10.1111/josh.12040

DRISCOLL DM, 1984, J PEDIATR ORTHOPED, V4, P677, DOI 10.1097/01241398-198411000-00004

Falick-Michaeli T, 2015, GLOB SPINE J, V5, P179, DOI 10.1055/s-0035-1552987

Gelalis ID, 2012, EUR SPINE J, V21, P1936, DOI 10.1007/s00586-012-2328-6

Grauers A, 2016, SCOLIOSIS SPINAL DIS, V11, DOI 10.1186/s13013-016-0105-8

Grauers A, 2014, SPINE, V39, P886, DOI 10.1097/BRS.0000000000000312

GREGORIC M, 1981, ACTA ORTHOP SCAND, V52, P59, DOI 10.3109/17453678108991759

Horne JP, 2014, AM FAM PHYSICIAN, V89, P193

Hosalkar Harish S, 2003, Pediatr Case Rev, V3, P189, DOI 10.1097/01.PCA.0000084260.07223.DC

Inoue M, 1998, J BONE JOINT SURG BR, V80B, P212, DOI 10.1302/0301-620X.80B2.7544

Kellis E, 2009, J PEDIATR ORTHOP B, V18, P275, DOI 10.1097/BPB.0b013e32832d5d3b

Kenanidis E, 2008, SPINE, V33, P2160, DOI 10.1097/BRS.0b013e31817d6db3

Kersten I, 2014, BMC PREGNANCY CHILDB, V14, DOI 10.1186/1471-2393-14-75

Kesling KL, 1997, SPINE, V22, P2009, DOI 10.1097/00007632-199709010-00014

Konieczny MR, 2013, J CHILD ORTHOP, V7, P3, DOI 10.1007/s11832-012-0457-4

Lebel DE, 2012, J MATERN-FETAL NEO M, V25, P639, DOI 10.3109/14767058.2011.598587

Longworth B, 2014, ARCH PHYS MED REHAB, V95, P1725, DOI 10.1016/j.apmr.2014.02.027

Mahaudens P, 2013, EUR SPINE J, V22, P2399, DOI 10.1007/s00586-013-2837-y

McMaster ME, 2015, SCOLIOSIS SPINAL DIS, V10, DOI 10.1186/s13013-015-0029-8

Miller NH, 2007, CLIN ORTHOP RELAT R, P6, DOI 10.1097/BLO.0b013e318126c062

Minghelli B, 2016, WORK, V54, P197, DOI 10.3233/WOR-162284

Nault ML, 2002, SPINE, V27, P1911, DOI 10.1097/00007632-200209010-00018

Ng Shu-Yan, 2017, Open Orthop J, V11, P1548, DOI 10.2174/1874325001711011548

Ogura Y, 2015, AM J HUM GENET, V97, P337, DOI 10.1016/j.ajhg.2015.06.012

Owen KB, 2014, PREV MED, V67, P270, DOI 10.1016/j.ypmed.2014.07.033

Parsch D, 2002, CLIN J SPORT MED, V12, P95, DOI 10.1097/00042752-200203000-00005

Pascoe DD, 1997, ERGONOMICS, V40, P631, DOI 10.1080/001401397187928

Ridley K, 2018, J SCI MED SPORT, V21, P930, DOI 10.1016/j.jsams.2018.01.019

Rizkallah M, 2018, ORTHOP TRAUMATOL-SUR, V104, P631, DOI 10.1016/j.otsr.2017.12.007

Rodriguez-Oviedo P, 2012, ARCH DIS CHILD, V97, P730, DOI 10.1136/archdischild-2011-301253

Rose PS, 2007, ORTHOP CLIN N AM, V38, P521, DOI 10.1016/j.ocl.2007.06.001

Sahli S, 2013, SPINE J, V13, P1835, DOI 10.1016/j.spinee.2013.06.023

Schwieger T, 2016, SCOLIOSIS SPINAL DIS, V11, DOI 10.1186/s13013-016-0084-9

Shamsoddini AR, 2010, IRAN J PUBLIC HEALTH, V39, P120

Skaggs DL, 2006, J PEDIATR ORTHOPED, V26, P358, DOI 10.1097/01.bpo.0000217723.14631.6e

Smith PS, 2003, INT J OBSTET ANESTH, V12, P17, DOI 10.1016/S0959-289X(02)00136-x

Tambe AD, 2018, BONE JOINT J, V100B, P415, DOI 10.1302/0301-620X.100B4.BJJ-2017-0846.R2

Tanchev PI, 2000, SPINE, V25, P1367, DOI 10.1097/00007632-200006010-00008

van Gent C, 2003, SPINE, V28, P916, DOI 10.1097/00007632-200305010-00014

Wall EJ, 2003, J PEDIATR ORTHOPED, V23, P437

Ward WT, 2017, SPINE, V42, P1233, DOI 10.1097/BRS.0000000000002004

WARREN MP, 1986, NEW ENGL J MED, V314, P1348, DOI 10.1056/NEJM198605223142104

Watanabe K, 2017, J BONE JOINT SURG AM, V99, P284, DOI 10.2106/JBJS.16.00459

Yaman O, 2014, TURK NEUROSURG, V24, P646, DOI 10.5137/1019-5149.JTN.8838-13.0

Zaina F, 2016, EUR SPINE J, V25, P2938, DOI 10.1007/s00586-016-4452-1

Zaina F, 2015, J PEDIATR-US, V166, P163, DOI 10.1016/j.jpeds.2014.09.024

1990, ACTA RADIOL, V31, P127

2018, J ORTHOP, V15, P319, DOI DOI 10.1016/J.JOR.2018.02.003

NR 64

TC 2

Z9 3

U1 2

U2 8

PU LIPPINCOTT WILLIAMS & WILKINS

PI PHILADELPHIA

PA TWO COMMERCE SQ, 2001 MARKET ST, PHILADELPHIA, PA 19103 USA

SN 1040-8703

EI 1531-698X

J9 CURR OPIN PEDIATR

JI CURR. OPIN. PEDIATR.

PD FEB

PY 2019

VL 31

IS 1

BP 48

EP 53

DI 10.1097/MOP.0000000000000705

PG 6

WC Pediatrics

WE Science Citation Index Expanded (SCI-EXPANDED)

SC Pediatrics

GA HX5LI

UT WOS:000467442300008

PM 30461512

DA 2023-08-10

ER

PT J

AU Bidari, S

Kamyab, M

Ahmadi, A

Ganjavian, MS

AF Bidari, Shahrbanoo

Kamyab, Mojtaba

Ahmadi, Amir

Ganjavian, Mohammad Saleh

TI Effect of exercise on static balance and Cobb angle during the weaning phase of brace management in idiopathic scoliosis and hyperkyphosis: A preliminary study

SO JOURNAL OF BACK AND MUSCULOSKELETAL REHABILITATION

LA English

DT Article

DE Spinal deformity; Blount and Moe exercise; static balance; brace

ID MILWAUKEE-BRACE; SCHEUERMANN KYPHOSIS

AB BACKGROUND: Exercises are usually prescribed in association with orthotic intervention for management of idiopathic scoliosis, however the role of these exercises on the efficacy of brace and/or balance is not clear yet.

OBJECTIVES: To investigate the role of exercise (the Blount and Moe protocol) on static balance and Cobb angle changes in adolescents with spinal deformities during weaning from brace.

METHODS: Seventeen brace users were allocated into 3 groups (good, moderate, and weak), according to their exercise quality and quantity static balance was evaluated on 4 conditions (standing on a platform/foam; with/without brace) using a force platform. Center of pressure displacement parameters were compared among the 3 groups. The mean Cobb angles of scoliosis and kyphosis at the beginning of brace use and at the start of the weaning phase were compared in general and among the 3 analogous groups.

RESULTS: No significant difference was found in the static balance parameters and also in Cobb angles among the 3 groups. However, scoliosis and kyphosis Cobb angles were improved significantly as a result of using the brace ($p < 0.01$).

CONCLUSIONS: The exercise quantity and quality in association with bracing, up to the weaning phase, has no effect on static balance and changes in scoliosis and kyphosis, but the curvature of scoliosis and kyphosis is reduced after wearing a brace.

C1 [Bidari, Shahrbanoo; Kamyab, Mojtaba] Iran Univ Med Sci, Sch Rehabil Sci, Dept Orthot & Prosthet, Sh Shahnazari St,Madar Sq,Mirdamad Blvd, Tehran 158754391, Iran.

[Ahmadi, Amir] Iran Univ Med Sci, Sch Rehabil Sci, Dept Physiotherapy, Tehran, Iran.

[Ganjavian, Mohammad Saleh] Iran Univ Med Sci, Shafa Yahyaiian Hosp, Dept Orthopaed Surg, Tehran, Iran.

C3 Iran University of Medical Sciences; Iran University of Medical

Sciences; Iran University of Medical Sciences

RP Kamyab, M (通讯作者)，Iran Univ Med Sci, Sch Rehabil Sci, Dept Orthot & Prosthet, Sh Shahnazari St,Madar Sq,Mirdamad Blvd, Tehran 158754391, Iran.

EM kamyab.m@iums.ac.ir

RI Kamyab, Mojtaba/AAA-5537-2019; bidari, shahrbanoo/ABG-9174-2020

OI Kamyab, Mojtaba/0000-0003-1081-3083;

CR Aubin CE, 1997, SPINE, V22, P629, DOI 10.1097/00007632-199703150-00010

Berdishevsky H, 2016, SCOLIOSIS SPINAL DIS, V11, DOI 10.1186/s13013-016-0076-9

BLOUNT W, 1973, MILWAUKEE BRACE

BoachieAdjei O, 1996, PEDIATR CLIN N AM, V43, P883, DOI 10.1016/S0031-3955(05)70440-5

CARMAN D, 1985, J PEDIATR ORTHOPED, V5, P65, DOI 10.1097/01241398-198501000-00011

Chow DHK, 2007, EUR SPINE J, V16, P1351, DOI 10.1007/s00586-007-0333-y

Coillard C, 1996, Eur Spine J, V5, P91, DOI 10.1007/BF00298387

Dalleau G, 2011, EUR SPINE J, V20, P123, DOI 10.1007/s00586-010-1554-z

DAY BL, 1993, J PHYSIOL-LONDON, V469, P479, DOI 10.1113/jphysiol.1993.sp019824

De Gauzy JS, 2002, ST HEAL T, V88, P239

De Mauroy JC, 2008, DISABIL REHABIL-ASSI, V3, P139, DOI 10.1080/17483100801904069

Dubousset J, 2001, SPINE, V26, P1001, DOI 10.1097/00007632-200105010-00002

DUVALBEAUPERE G, 1985, SPINE, V10, P428, DOI 10.1097/00007632-198506000-00005

Eisinger DB, 1996, AM J PHYS MED REHAB, V75, P194, DOI 10.1097/00002060-199605000-00008

Faul F, 2007, BEHAV RES METHODS, V39, P175, DOI 10.3758/BF03193146

FORD DM, 1984, SPINE, V9, P373, DOI 10.1097/00007632-198405000-00008

Friedrich M, 1996, PHYS THER, V76, P1082, DOI 10.1093/ptj/76.10.1082

Fusco C., 2011, Physiotherapy Theory and Practice, V27, P80, DOI 10.3109/09593985.2010.533342

Haumont T, 2011, SPINE, V36, pE847, DOI 10.1097/BRS.0b013e3181ff5837

HERMAN R, 1985, SPINE, V10, P1, DOI 10.1097/00007632-198501000-00001

HORAK FB, 1987, PHYS THER, V67, P1881, DOI 10.1093/ptj/67.12.1881

Konz R, 2006, J REHABILITATION RES, V43

LONSTEIN JE, 1994, J BONE JOINT SURG AM, V76A, P1207, DOI 10.2106/00004623-199408000-00011

Machida M, 1999, SPINE, V24, P2576, DOI 10.1097/00007632-199912150-00004

Mehdikhani M, 2013, PROSTHET ORTHOT INT

MONTGOMERY SP, 1981, SPINE, V6, P5, DOI 10.1097/00007632-198101000-00002

Monticone M, 2014, EUR SPINE J, V23, P1204, DOI 10.1007/s00586-014-3241-y

NACHEMSON A L, 1977, Spine, V2, P176, DOI 10.1097/00007632-197709000-00003

Nault ML, 2002, SPINE, V27, P1911, DOI 10.1097/00007632-200209010-00018

Negrini A, 2001, EUR MED PHYS, V37, P181

Negrini S., 2007, EVIDENCE BASED ISICO

Negrini S, 2008, J REHABIL MED, V40, P451, DOI 10.2340/16501977-0195

Negrini S, 2011, SCOLIOSIS SPINAL DIS, V6, DOI 10.1186/1748-7161-6-8

Nitzschke E, 1989, Z ORTHOPADIE IHRE GR, V128, P477

Nowakowski A, 1997, CHIRURG NARZADOW RUC, V63, P317

Richards BS, 2005, SPINE, V30, P2068, DOI 10.1097/01.brs.0000178819.90239.d0

Rigo M, 2017, SCOLIOSIS SPINAL DIS, V12, P1, DOI 10.1186/s13013-017-0114-2

Romano M, 2006, SCOLIOSIS, V1, P1

Romano M, 2013, SPINE, V38, pE883, DOI 10.1097/BRS.0b013e31829459f8

Rowe DE, 1997, J BONE JOINT SURG AM, V79A, P664, DOI 10.2106/00004623-199705000-00005

SACHS B, 1987, J BONE JOINT SURG AM, V69A, P50, DOI 10.2106/00004623-198769010-00009

SHUMWAYCOOK A, 1986, PHYS THER, V66, P1548, DOI 10.1093/ptj/66.10.1548

STOKES IAF, 1987, J ORTHOP RES, V5, P102, DOI 10.1002/jor.1100050113

Stokes OM, 2013, BONE JOINT J, V95B, P1308, DOI 10.1302/0301-620X.95B10.31474

Weinstein SL, 2013, NEW ENGL J MED, V369, P1512, DOI 10.1056/NEJMoa1307337

Wenger DR, 1999, SPINE, V24, P2630, DOI 10.1097/00007632-199912150-00010

WOOLF SH, 1993, JAMA-J AM MED ASSOC, V269, P2667

WYNARSKY GT, 1989, SPINE, V14, P1283, DOI 10.1097/00007632-198912000-00002

Zaina F, 2009, SCOLIOSIS SPINAL DIS, V4, DOI 10.1186/1748-7161-4-8

NR 49

TC 6

Z9 5

U1 1

U2 9

PU IOS PRESS

PI AMSTERDAM

PA NIEUWE HEMWEG 6B, 1013 BG AMSTERDAM, NETHERLANDS

SN 1053-8127

EI 1878-6324

J9 J BACK MUSCULOSKELET

JI J. Back Musculoskelet. Rehabil.

PY 2019

VL 32

IS 4

BP 639

EP 646

DI 10.3233/BMR-181128

PG 8

WC Orthopedics; Rehabilitation

WE Science Citation Index Expanded (SCI-EXPANDED)

SC Orthopedics; Rehabilitation

GA IM5CU

UT WOS:000478012600014

PM 30614790

DA 2023-08-10

ER

PT J

AU Levi, D

Springer, S

Parmet, Y

Ovadia, D

Ben-Sira, D

AF Levi, Dror

Springer, Shmuel

Parmet, Yisrael

Ovadia, Dror

Ben-Sira, David

TI Acute muscle stretching and the ability to maintain posture in females

with adolescent idiopathic scoliosis

SO JOURNAL OF BACK AND MUSCULOSKELETAL REHABILITATION

LA English

DT Article

DE Scoliosis; posture; exercise; stretching

ID REACTION-TIME; PEAK TORQUE; STRENGTH; EXERCISES; ENDURANCE; BALANCE;

SPINE; POWER; BACK

AB BACKGROUND: Physiotherapy scoliosis specific exercises include exercises to attain and maintain proper posture, as well as flexibility movements such as stretching.

OBJECTIVE: To examine the effect of prior muscle stretching on the performance of posture exercise in females with adolescent idiopathic scoliosis (AIS).

METHODS: Eighteen females with AIS were randomly assigned to perform a posture maintenance task for three minutes preceded by either stretching protocol (group A) or no-stretching (group B). A

second session was carried out after three days, where the same procedure was repeated in a reverse order between groups. During each session, three outcomes were tested: the ability to complete the task, the ability to maintain postural body alignment, and the perceived effort.

RESULTS: All participants completed the task in both sessions. Subjects' ability to preserve the required lower trunk alignment decreased following stretching compared to no-stretching. Lower-trunk angle changed toward flexion by 10 degrees and 4.3 degrees respectively, p = 0.032. There was no difference in perceived effort.

CONCLUSIONS: Prior muscle stretching has a negative effect on the ability to preserve body positional alignment during posture exercise. The present findings should be considered by practitioners when designing protocols for scoliosis-specific exercises. Stretching immediately prior to posture maintenance exercises should be avoided.

C1 [Levi, Dror] Macabbi Hlth Care Serv, Bat Yam, Israel.

[Springer, Shmuel] Ariel Univ, Fac Hlth Sci, Dept Phys Therapy, Ariel, Israel.

[Parmet, Yisrael] Ben Gurion Univ Negev, Beer Sheva, Israel.

[Ovadia, Dror] Dana Childrens Hosp, Tel Aviv Med Ctr, Dept Pediat Orthoped, Tel Aviv, Israel.

[Levi, Dror; Ben-Sira, David] Zinman Coll Phys Educ & Sport Sci, Wingate, Israel.

C3 Ariel University; Ben Gurion University; Tel Aviv University; Sackler

Faculty of Medicine

RP Springer, S (通讯作者)， Ariel Univ, Fac Hlth Sci, Dept Phys Therapy, Ariel, Israel.

EM shmuels@ariel.ac.il

RI PARMET, YISRAEL/F-1982-2012

OI PARMET, YISRAEL/0000-0002-2071-7338

CR Avela J, 1999, J APPL PHYSIOL, V86, P1292, DOI 10.1152/jappl.1999.86.4.1292

Behm DG, 2011, EUR J APPL PHYSIOL, V111, P2633, DOI 10.1007/s00421-011-1879-2

Behm DG, 2004, MED SCI SPORT EXER, V36, P1397, DOI 10.1249/01.MSS.0000135788.23012.5F

Bengtsson V, 2017, J SPORTS MED PHYS FI

Bettany-Saltikov J, 2014, EUR J PHYS REHAB MED, V50, P111

BORG GAV, 1982, MED SCI SPORT EXER, V14, P377, DOI 10.1249/00005768-198205000-00012

Borghuis J, 2008, SPORTS MED, V38, P893, DOI 10.2165/00007256-200838110-00002

Chatzopoulos D, 2014, J SPORT SCI MED, V13, P403

Czaprowski D, 2011, SCOLIOSIS SPINAL DIS, V6, DOI 10.1186/1748-7161-6-22

Danielsson AJ, 2006, SPINE, V31, P275, DOI 10.1097/01.brs.0000197652.52890.71

Fowles JR, 2000, J APPL PHYSIOL, V89, P1179, DOI 10.1152/jappl.2000.89.3.1179

Guadagnoli MA, 2004, J MOTOR BEHAV, V36, P212, DOI 10.3200/JMBR.36.2.212-224

Hawes Martha C, 2003, Pediatr Rehabil, V6, P171

Herda TJ, 2008, J STRENGTH COND RES, V22, P809, DOI 10.1519/JSC.0b013e31816a82ec

Kee D, 2012, APPL ERGON, V43, P277, DOI 10.1016/j.apergo.2011.06.002

Kubo K, 2002, ACTA PHYSIOL SCAND, V175, P157, DOI 10.1046/j.1365-201X.2002.00976.x

Kubo K, 2001, J APPL PHYSIOL, V90, P520, DOI 10.1152/jappl.2001.90.2.520

Le Berre M, 2016, EUR SPINE J, P1

Lehnert-Schroth C., 2007, 3 DIMENSIONAL TREATM, V7th ed.

Magee DJ, 2020, ORTHOPEDIC PHYS ASSE

Manoel ME, 2008, J STRENGTH COND RES, V22, P1528, DOI 10.1519/JSC.0b013e31817b0433

Marek SM, 2005, J ATHL TRAINING, V40, P94

Minehisa K, 2003, J PHYSIC THERAP SCI, V15, P105, DOI [10.1589/jpts.15.105, DOI 10.1589/JPTS.15.105]

Moon C., 2005, J KOREAN I IND ENG, V31, P289

Myers T. W., 2013, ANATOMY TRAINS E BOO

Negrini S, 2017, EUR J PHYS REHAB MED, V53, P125, DOI 10.23736/S1973-9087.16.04406-3

Negrini S, 2012, SCOLIOSIS SPINAL DIS, V7, DOI 10.1186/1748-7161-7-3

Nelson AG, 2005, J STRENGTH COND RES, V19, P338

Page P, 2012, INT J SPORTS PHYS TH, V7, P109

Park J, 2017, EUR J PHYS REHAB MED

Park W, 2009, ERGONOMICS, V52, P1169, DOI 10.1080/00140130902971908

Rigo M, 2003, Pediatr Rehabil, V6, P209

Rubini EC, 2007, SPORTS MED, V37, P213, DOI 10.2165/00007256-200737030-00003

Sherman KJ, 2013, EVIDENCE BASED COMPL, V2013

Shin G, 2009, SPINE, V34, P1873, DOI 10.1097/BRS.0b013e3181aa6a55

SHRIER I, 2002, EVIDENCE BASED SPORT, V9, P43

Siatras TA, 2008, J STRENGTH COND RES, V22, P40, DOI 10.1519/JSC.0b013e31815f970c

Watson AWS, 2000, J SPORT MED PHYS FIT, V40, P260

Weerapong P., 2004, PHYS THERAPY REV, V9, P189, DOI [DOI 10.1179/108331904225007078, 10.1179/108331904225007078]

Weinstein SL, 2008, LANCET, V371, P1527, DOI 10.1016/S0140-6736(08)60658-3

Weiss HR, 2008, EUR J PHYS REHAB MED, V44, P177

WILSON GJ, 1994, J APPL PHYSIOL, V76, P2714, DOI 10.1152/jappl.1994.76.6.2714

Yang JM, 2015, J PHYS THER SCI, V27, P2667, DOI 10.1589/jpts.27.2667

Zakaria A, 2012, J PHYS THER SCI, V24, P1127, DOI 10.1589/jpts.24.1127

Zhu XH, 2013, J ELECTROMYOGR KINES, V23, P801, DOI 10.1016/j.jelekin.2013.04.009

NR 45

TC 3

Z9 3

U1 4

U2 10

PU IOS PRESS

PI AMSTERDAM

PA NIEUWE HEMWEG 6B, 1013 BG AMSTERDAM, NETHERLANDS

SN 1053-8127

EI 1878-6324

J9 J BACK MUSCULOSKELET

JI J. Back Musculoskelet. Rehabil.

PY 2019

VL 32

IS 4

BP 655

EP 662

DI 10.3233/BMR-181175

PG 8

WC Orthopedics; Rehabilitation

WE Science Citation Index Expanded (SCI-EXPANDED); Social Science Citation Index (SSCI)

SC Orthopedics; Rehabilitation

GA IM5CU

UT WOS:000478012600016

PM 30636726

DA 2023-08-10

ER

PT J

AU Ko, JY

Suh, JH

Kim, H

Ryu, JS

AF Ko, Jin Young

Suh, Jee Hyun

Kim, Hayoung

Ryu, Ju Seok

TI Proposal of a new exercise protocol for idiopathic scoliosis A

preliminary study

SO MEDICINE

LA English

DT Article

DE exercise therapy; idiopathic scoliosis; rehabilitation; spinal

curvatures; spinal disease

ID MUSCLES; EFFICACY

AB In clinical practice, we found a unilateral instability in patients with right thoracic scoliosis during asymmetric spinal stabilization exercise (ASSE), which can be an important clue to identify the pathophysiology of idiopathic scoliosis (IS).

We investigated the relationship between unilateral postural instability and weakness of paraspinal muscles according to curve pattern. And finally, we propose the new exercise method based on the curve pattern.

Combined use of prospective and retrospective clinical trials.

Fifteen participants without IS and 10 patients with IS in 1 tertiary referral hospital.

In 15 participants without IS, surface electromyography (sEMG) was used to evaluate the muscular activation patterns in the bilateral erector spinae (ES), rectus abdominis (RA), and external oblique (EO) muscles during ASSE. In addition, to assess the clinical effect of ASSE, Cobb angle and rotation grade were measured from 10 patients with IS.

The most significant findings from the sEMG data were the increased activities of ipsilateral 7th thoracic ES during hand-up motion, ipsilateral 3rd lumbar ES during leg-up motion, and 12th thoracic and 3rd lumbar ES during side-bridging. In a radiographic analysis, specific components of ASSE that activates the concave side muscles were found to be effective for IS.

The paraspinal muscle strengthening of the concave side using ASSE can improve the severity of scoliosis. Based on this research, we could propose a new exercise protocol that can be personalized according to the curve pattern.

C1 [Ko, Jin Young; Kim, Hayoung; Ryu, Ju Seok] Seoul Natl Univ, Coll Med, Bundang Hosp, Dept Rehabil Med, 82 Gumi Ro 173 Beon Gil, Seongnam Si 463707, Gyeonggi Do, South Korea.

[Suh, Jee Hyun] Bobath Childrens Clin, Dept Rehabil Med, Yongin, Gyeonggi Do, South Korea.

C3 Seoul National University (SNU)

RP Ryu, JS (通讯作者)， Seoul Natl Univ, Coll Med, Bundang Hosp, Dept Rehabil Med, 82 Gumi Ro 173 Beon Gil, Seongnam Si 463707, Gyeonggi Do, South Korea.

EM jseok337@snu.ac.kr

RI Kim, Hayoung/GQP-0924-2022; Ryu, Ju Seok/W-4027-2019

OI Ryu, Ju Seok/0000-0003-3299-3038; Ko, Jin Young/0000-0002-6263-8355

FU SNUBH Research Fund [09-2017-001]

FX This work was Supported by grant no 09-2017-001 from the SNUBH Research

Fund.

CR Avikainen VJ, 1999, J SPINAL DISORD, V12, P61

Berdishevsky H, 2016, SCOLIOSIS SPINAL DIS, V11, DOI 10.1186/s13013-016-0076-9

Cheung J, 2005, EUR SPINE J, V14, P130, DOI 10.1007/s00586-004-0780-7

Cheung J, 2004, SPINE, V29, P1011, DOI 10.1097/00007632-200405010-00012

Chwala W, 2012, STUD HEALTH TECHNOL, V176, P129, DOI 10.3233/978-1-61499-067-3-129

DICKSON RA, 1984, J BONE JOINT SURG BR, V66, P8, DOI 10.1302/0301-620X.66B1.6693483

Gaudreault N, 2005, BMC MUSCULOSKEL DIS, V6, DOI 10.1186/1471-2474-6-14

Hresko MT, 2013, NEW ENGL J MED, V368, P834, DOI 10.1056/NEJMcp1209063

Kim CR, 2016, PM&R, V8, P979, DOI 10.1016/j.pmrj.2016.05.017

Kuru T, 2016, CLIN REHABIL, V30, P181, DOI 10.1177/0269215515575745

Lee HS, 2016, J BACK MUSCULOSKELET, V29, P603, DOI 10.3233/BMR-160724

Mamyama Toni, 2002, Stud Health Technol Inform, V91, P361

Mannion A F, 1998, Eur Spine J, V7, P289, DOI 10.1007/s005860050077

Maruyama Toru, 2003, Pediatr Rehabil, V6, P215

Mordecai SC, 2012, EUR SPINE J, V21, P382, DOI 10.1007/s00586-011-2063-4

NAEIJE M, 1989, J ORAL REHABIL, V16, P63, DOI 10.1111/j.1365-2842.1989.tb01318.x

Negrini S, 2014, BMC MUSCULOSKEL DIS, V15, DOI 10.1186/1471-2474-15-263

Plaszewski M, 2014, PLOS ONE, V9, DOI 10.1371/journal.pone.0110254

Rigo M, 2009, STUD HEALTH TECHNOL, V135, P208

Romano M, 2015, SCOLIOSIS SPINAL DIS, V10, DOI 10.1186/s13013-014-0027-2

Romano M, 2013, SPINE, V38, pE883, DOI 10.1097/BRS.0b013e31829459f8

Schmid AB, 2010, J SPORT REHABIL, V19, P315, DOI 10.1123/jsr.19.3.315

Schreiber S, 2016, PLOS ONE, V11, DOI 10.1371/journal.pone.0168746

Suh SW, 2011, EUR SPINE J, V20, P1087, DOI 10.1007/s00586-011-1695-8

Weiss HR, 2006, SCOLIOSIS SPINAL DIS, V1, DOI 10.1186/1748-7161-1-6

NR 25

TC 9

Z9 9

U1 0

U2 10

PU LIPPINCOTT WILLIAMS & WILKINS

PI PHILADELPHIA

PA TWO COMMERCE SQ, 2001 MARKET ST, PHILADELPHIA, PA 19103 USA

SN 0025-7974

EI 1536-5964

J9 MEDICINE

JI Medicine (Baltimore)

PD DEC

PY 2018

VL 97

IS 49

AR e13336

DI 10.1097/MD.0000000000013336

PG 9

WC Medicine, General & Internal

WE Science Citation Index Expanded (SCI-EXPANDED)

SC General & Internal Medicine

GA HI3AM

UT WOS:000456318600030

PM 30544395

OA Green Published, gold

DA 2023-08-10

ER

PT J

AU Liang, JP

Zhou, X

Chen, N

Li, X

Yu, H

Yang, YQ

Song, YY

Du, Q

AF Liang, Juping

Zhou, Xuan

Chen, Nan

Li, Xin

Yu, Hong

Yang, Yuqi

Song, Yuanyuan

Du, Qing

TI Efficacy of three-dimensionally integrated exercise for scoliosis in

patients with adolescent idiopathic scoliosis: study protocol for a

randomized controlled trial

SO TRIALS

LA English

DT Article

DE Adolescent idiopathic scoliosis; Three-dimensionally integrated

exercise; Cobb angle; ATR; Sagittal profile; Quality of life

ID QUALITY-OF-LIFE; SCHROTH EXERCISES; QUESTIONNAIRE; RELIABILITY;

VALIDITY; KYPHOSIS; ANGLE

AB Background: Adolescent idiopathic scoliosis (AIS) is one of the most prevalent spinal deformities that may progress sharply during growth. The aim of this study will be to evaluate the efficacy of three-dimensionally integrated exercise on the Cobb angle, angle of trunk rotation, sagittal profile, and quality of life in patients with AIS.

Methods/design: The study is designed as a randomized controlled trial. Participants include 42 patients with AIS aged 10-16 years. Randomly assigned patients will follow a 6-month treatment, either in a control group with standard care of observation following the Scoliosis Research Society criteria or in an experimental group with three-dimensionally integrated exercise for scoliosis. Blinded assessments at baseline and immediately after intervention will include the change of Cobb angle, angle of trunk rotation, sagittal index, and quality of life.

Discussion: If we find that the intervention is effective in improving Cobb angle, angle of trunk rotation, sagittal profile, and quality of life in patients with AIS, this trial will have a positive impact and warrant a change in clinical practice.

C1 [Liang, Juping; Zhou, Xuan; Chen, Nan; Li, Xin; Yu, Hong; Du, Qing] Shanghai Jiao Tong Univ, Sch Med, Xinhua Hosp, Dept Rehabil Med, Shanghai 200092, china.

[Yang, Yuqi] Shanghai Univ Med & Hlth Sci, Sch Nursing & Hlth Management, Shanghai 201318, china.

[Song, Yuanyuan] Shanghai Jiao Tong Univ, Xinhua Hosp, Sch Med, Dept Rehabil Med,Chongming Branch, Shanghai 202150, china.

C3 Shanghai Jiao Tong University; Shanghai University of Medicine & Health Sciences; Shanghai Jiao Tong University

RP Du, Q (通讯作者)， Shanghai Jiao Tong Univ, Sch Med, Xinhua Hosp, Dept Rehabil Med, Shanghai 200092, china.

EM duqing@xinhuamed.com.cn

RI Du, Qing/HKN-6976-2023

FU Xinhua Hospital affiliated to Shanghai Jiao Tong University School of Medicine [17CSK02]; Clinical Research Unit of Xinhua Hospital; Key Developing Disciplines Construction Program (Rehabilitation Medicine) of Shanghai Municipal Commission of Health and Family Planning [2015ZB0406]; Xinhua Hospital affiliated to Shanghai Jiao Tong University School of Medicine Chongming Branch [YL201701]

FX This work is supported by the Xinhua Hospital affiliated to Shanghai Jiao Tong University School of Medicine and the Clinical Research Unit of Xinhua Hospital. It is funded by the Xinhua Hospital affiliated to Shanghai Jiao Tong University School of Medicine (17CSK02), the Key Developing Disciplines Construction Program (Rehabilitation Medicine) of Shanghai Municipal Commission of Health and Family Planning (2015ZB0406), and Xinhua Hospital affiliated to Shanghai Jiao Tong University School of Medicine Chongming Branch (YL201701).

CR [Anonymous], SCOLIOSIS S2

Asher M, 2003, SPINE, V28, P74, DOI 10.1097/00007632-200301010-00017

Asher M, 2003, SPINE, V28, P63, DOI 10.1097/00007632-200301010-00015

Asher M, 2003, SPINE, V28, P70, DOI 10.1097/00007632-200301010-00016

Berdishevsky H, 2016, SCOLIOSIS SPINAL DIS, V11, DOI 10.1186/s13013-016-0076-9

Bialek M, 2015, MEDICINE, V94, DOI 10.1097/MD.0000000000000863

BUNNELL WP, 1984, J BONE JOINT SURG AM, V66A, P1381, DOI 10.2106/00004623-198466090-00010

BUNNELL WP, 1993, SPINE, V18, P1572, DOI 10.1097/00007632-199309000-00001

Chen N, 2015, ZHONGGUO KANG FU, V30, P247

Cobb JR, 1948, PHYS THER, V59, P764

Coelho DM, 2013, BRAZ J PHYS THER, V17, P179, DOI 10.1590/S1413-35552012005000081

Cohen J., 2013, STAT POWER ANAL BEHA, V2nd

Du Q, 2013, ZHONGGUO KANG FU YI, V28, P507

Du Q., 2014, STUDY REHABILITATION

Du Q, 2016, BMC MUSCULOSKEL DIS, V17, DOI 10.1186/s12891-016-1140-6

Du Q, 2015, J MANIP PHYSIOL THER, V38, P434, DOI 10.1016/j.jmpt.2015.06.009

Emans JB, 2003, REFERENCE MANUAL BOS

Fan HW, 2016, SPINE, V41, P259, DOI 10.1097/BRS.0000000000001197

Friedrich M, 1998, ARCH PHYS MED REHAB, V79, P475, DOI 10.1016/S0003-9993(98)90059-4

Fusco C, 2014, SCOLIOSIS SPINAL DIS, V9, DOI 10.1186/1748-7161-9-12

Jelacic M, 2012, SCOLIOSIS, V7, pO57

Kuru T, 2016, CLIN REHABIL, V30, P181, DOI 10.1177/0269215515575745

Little RJ, 2012, NEW ENGL J MED, V367, P1355, DOI 10.1056/NEJMsr1203730

Liu W, 2011, ORTHOP J CHIN, V19, P1244

Monticone M, 2014, EUR SPINE J, V23, P1204, DOI 10.1007/s00586-014-3241-y

MORRISSY RT, 1990, J BONE JOINT SURG AM, V72A, P320, DOI 10.2106/00004623-199072030-00002

Negrini A, 2016, SCOLIOSIS SPINAL DIS, V11, DOI 10.1186/s13013-016-0100-0

Negrini S., 2007, EVIDENCE BASED ISICO

Negrini S, 2012, SCOLIOSIS SPINAL DIS, V7, DOI 10.1186/1748-7161-7-3

Parent EC, 2013, SCOLIOSIS SUPPL 2, V8, pO45

PONSETI IV, 1950, J BONE JOINT SURG AM, V32-A, P381, DOI 10.2106/00004623-195032020-00017

Risser J., 1957, CLIN ORTHOP RELAT R, V11, P111

Romano M, 2015, SCOLIOSIS SPINAL DIS, V10, DOI 10.1186/s13013-014-0027-2

Schreiber S, 2017, SCOLIOSIS SPINAL DIS, V12, DOI 10.1186/s13013-017-0137-8

Schreiber S, 2015, SCOLIOSIS SPINAL DIS, V10, DOI 10.1186/s13013-015-0048-5

Schulz KF, 2010, J PHARMACOL PHARMACO, V1, P100, DOI [10.1016/j.jclinepi.2010.02.005, 10.1016/j.ijsu.2011.09.004, 10.1136/bmj.c869, 10.4103/0976-500X.72352, 10.1016/j.jclinepi.2010.03.004, 10.1186/1741-7015-8-18]

Scoliosis Research Society, AF ID SCOL TREATM

Senn S., 2007, STAT ISSUES DRUG DEV, Vsecond ed.

STRECHER VJ, 1983, PATIENT COUNS HEALTH, V4, P129, DOI 10.1016/S0190-2040(83)80002-0

Weinstein SL, 2008, LANCET, V371, P1527, DOI 10.1016/S0140-6736(08)60658-3

Williams MA, 2015, HEALTH TECHNOL ASSES, V19, DOI 10.3310/hta19550

World Health Organization (WHO), WHO TRIAL REG DAT SE

Zaina F, 2009, EUR J PHYS REHAB MED, V45, P595

Zaina F, 2012, STUD HEALTH TECHNOL, V176, P264, DOI 10.3233/978-1-61499-067-3-264

Zaina F, 2009, STUD HEALTH TECHNOL, V135, P125

Zhao L, 2007, J CHILD ORTHOP, V1, P351, DOI 10.1007/s11832-007-0061-1

Zhou X, 2011, ZHONGHUA WU LI YI XU, V33, P668

NR 47

TC 5

Z9 6

U1 3

U2 26

PU BMC

PI LONDON

PA CAMPUS, 4 CRINAN ST, LONDON N1 9XW, united kingdom

EI 1745-6215

J9 TRIALS

JI Trials

PD SEP 10

PY 2018

VL 19

AR 485

DI 10.1186/s13063-018-2834-x

PG 11

WC Medicine, Research & Experimental

WE Science Citation Index Expanded (SCI-EXPANDED)

SC Research & Experimental Medicine

GA GT0PI

UT WOS:000444146900007

PM 30201050

OA gold, Green Published

DA 2023-08-10

ER

PT J

AU Laita, LC

Cubillo, CT

Gomez, TM

del Barrio, SJ

AF Ceballos Laita, Luis

Tejedor Cubillo, Cristina

Mingo Gomez, Teresa

Jimenez del Barrio, Sandra

TI Effects of corrective, therapeutic exercise techniques on adolescent

idiopathic scoliosis. A systematic review

SO ARCHIVOS ARGENTINOS DE PEDIATRIA

LA English

DT Review

DE scoliosis; adolescent; applied kinesiology

ID RANDOMIZED CONTROLLED-TRIAL; LOW-BACK-PAIN; STABILIZATION EXERCISE;

GENERAL EXERCISE; REHABILITATION; SCHROTH; REDUCE; MANAGEMENT;

ENDURANCE; ANGLE

AB The objective of this study was to determine the effects of corrective, therapeutic exercise techniques on subjects with adolescent idiopathic scoliosis. A systematic review was conducted by searching the Cochrane Library Plus, Pubmed, PEDro, and SCOPUS databases. Studies in patients diagnosed with adolescent idiopathic scoliosis that considered corrective, therapeutic exercise as an independent outcome measure and symptoms, functional capacity, Cobb's angle and/or other angles or body asymmetries as dependent outcome measures were included. A total of 9 controlled clinical trials that carried out corrective, therapeutic exercise were included. Corrective, therapeutic exercise appears to have positive effects by reducing symptoms and improving function, as well as various angles and body asymmetries. However, further studies with better methodological quality are required to confirm these outcomes and determine the best therapeutic exercise intervention.

C1 [Ceballos Laita, Luis; Mingo Gomez, Teresa; Jimenez del Barrio, Sandra] Univ Valladolid, Sch Fac Phys Therapy, Duques de Soria Univ Campus, Soria, Spain.

C3 Universidad de Valladolid

RP Laita, LC (通讯作者)，Univ Valladolid, Sch Fac Phys Therapy, Duques de Soria Univ Campus, Soria, Spain.

EM Luis.ceballos@uva.es

RI Ceballos-Laita, Luis/AAY-2167-2021

OI Ceballos, Luis/0000-0002-2170-8783

CR Ajimsha MS, 2015, J BODYW MOV THER, V19, P102, DOI 10.1016/j.jbmt.2014.06.001

Asociacion Espanola de Pediatria de Atencion Primaria, PED EUR ORG FORM

Berdishevsky H, 2016, SCOLIOSIS SPINAL DIS, V11, DOI 10.1186/s13013-016-0076-9

BUNNELL WP, 1986, SPINE, V11, P773, DOI 10.1097/00007632-198610000-00003

Chin K., 2001, CONT PEDIAT, V18, P77

Danielsson AJ, 2006, SPINE, V31, P275, DOI 10.1097/01.brs.0000197652.52890.71

Dantas Diego De Sousa, 2017, J Phys Ther Sci, V29, P1, DOI 10.1589/jpts.29.1

Diab AA, 2012, CLIN REHABIL, V26, P1123, DOI 10.1177/0269215512447085

Alves VLD, 2006, CHEST, V130, P500, DOI 10.1378/chest.130.2.500

Fusco C., 2011, Physiotherapy Theory and Practice, V27, P80, DOI 10.3109/09593985.2010.533342

Gacitua MV, 2016, ARCH ARGENT PEDIATR, V114, P585, DOI 10.5546/aap.2016.585

Gotfryd Alberto Ofenhejm, 2014, MedicalExpress (São Paulo, online), V1, P170, DOI 10.5935/MedicalExpress.2014.04.02

Gur G, 2017, PROSTHET ORTHOT INT, V41, P303, DOI 10.1177/0309364616664151

Hawes Martha C, 2003, Pediatr Rehabil, V6, P171

Kim G, 2016, J PHYS THER SCI, V28, P1012, DOI 10.1589/jpts.28.1012

Koumantakis GA, 2005, CLIN BIOMECH, V20, P474, DOI 10.1016/j.clinbiomech.2004.12.006

Koumantakis GA, 2005, PHYS THER, V85, P209, DOI 10.1093/ptj/85.3.209

Kumar A, 2017, J CLIN DIAGN RES, V11, pYC1, DOI 10.7860/JCDR/2017/27497.10335

Kuru T, 2016, CLIN REHABIL, V30, P181, DOI 10.1177/0269215515575745

Lapuente JP, 2002, ST HEAL T, V88, P258

Louw A, 2011, ARCH PHYS MED REHAB, V92, P2041, DOI 10.1016/j.apmr.2011.07.198

Martinez-Llorens J, 2010, EUR RESPIR J, V36, P393, DOI 10.1183/09031936.00025509

Marzo-Castillejo M, 2007, GUIAS CLIN S1, V7, P1

Monticone M, 2014, EUR SPINE J, V23, P1204, DOI 10.1007/s00586-014-3241-y

Negrini A, 2001, EUR MED PHYS, V37, P181

Negrini S, 2008, DISABIL REHABIL, V30, P772, DOI 10.1080/09638280801889568

Negrini S, 2008, J REHABIL MED, V40, P451, DOI 10.2340/16501977-0195

Negrini S, 2012, SCOLIOSIS SPINAL DIS, V7, DOI 10.1186/1748-7161-7-3

Negrini S, 2015, SCOLIOSIS SPINAL DIS, V10, DOI 10.1186/s13013-014-0025-4

Plewka Barbara, 2013, Pol Orthop Traumatol, V78, P85

Reamy BV, 2001, AM FAM PHYSICIAN, V64, P111

Rigo M, 2009, STUD HEALTH TECHNOL, V135, P208

Sato T, 2011, EUR SPINE J, V20, P274, DOI 10.1007/s00586-010-1657-6

Schlosser TPC, 2014, PLOS ONE, V9, DOI 10.1371/journal.pone.0097461

Schreiber S, 2016, PLOS ONE, V11, DOI 10.1371/journal.pone.0168746

Schreiber S, 2015, SCOLIOSIS SPINAL DIS, V10, DOI 10.1186/s13013-015-0048-5

Shamseer L, 2015, BMJ-BRIT MED J, V349, DOI [10.1136/bmj.i4086, 10.1186/2046-4053-4-1, 10.1136/bmj.g7647, 10.1136/bmj.b2535]

Smorgick Y, 2013, J PEDIATR ORTHOPED, V33, P289, DOI 10.1097/BPO.0b013e31827d0b43

Theroux J, 2015, PAIN RES MANAG, V20, P153, DOI 10.1155/2015/674354

Trobisch P, 2010, DTSCH ARZTEBL INT, V107, P875, DOI 10.3238/arztebl.2010.0875

Weiss HR, 2006, ST HEAL T, V123, P594

Weiss HR, 2016, ASIAN SPINE J, V10, P570, DOI 10.4184/asj.2016.10.3.570

Yang JM, 2015, J PHYS THER SCI, V27, P2667, DOI 10.1589/jpts.27.2667

Zapata KA, 2017, PEDIATR PHYS THER, V29, P62, DOI 10.1097/PEP.0000000000000325

Zapata KA, 2015, PEDIATR PHYS THER, V27, P396, DOI 10.1097/PEP.0000000000000174

NR 45

TC 17

Z9 18

U1 3

U2 45

PU SOC ARGENTINA PEDIATRIA

PI CAP FED BUENO AIRES

PA AV COLONEL DIAZ 1971-75-C1425DQF, CAP FED BUENO AIRES, 00000, ARGENTINA

SN 0325-0075

EI 1668-3501

J9 ARCH ARGENT PEDIATR

JI Arch. Argent. Pediatr.

PD AUG

PY 2018

VL 116

IS 4

BP E582

EP E589

DI 10.5546/aap.2018.eng.e582

PG 8

WC Pediatrics

WE Science Citation Index Expanded (SCI-EXPANDED)

SC Pediatrics

GA GO2OL

UT WOS:000439814600022

PM 30016036

OA gold

DA 2023-08-10

ER

PT J

AU Park, JH

Jeon, HS

Park, HW

AF Park, Joo-Hee

Jeon, Hye-Seon

Park, Ha-Won

TI Effects of the Schroth exercise on idiopathic scoliosis: a meta-analysis

SO EUROPEAN JOURNAL OF PHYSICAL AND REHABILITATION MEDICINE

LA English

DT Review

DE Meta-analysis; Exercise; Scoliosis

ID REHABILITATION; ADOLESCENTS; QUALITY; THERAPY; REDUCE; AIS

AB INTRODUCTION: The purpose of this study was to examine the effects of the Schroth exercise on idiopathic scoliosis. The overall effect size was analyzed in 15 primary studies and a subgroup analysis of the standardized mean differences of effect sizes from 15 primary studies was also conducted.

EVIDENCE ACQUISITION: We used PUBMED, MEDLINE, NDSL, EMBASE, and Web of Science. The key terms used in these searches were "Schroth," "scoliosis-specific exercise," "scoliosis," and "idiopathic scoliosis."

EVIDENCE SYNTHESIS: Cobb's angle, asymmetry, angle of trunk rotation (ATR), strength of back extensor, strength of trunk flexor, quality of life (QOL), balance, chest expansion, and pulmonary function were coded as outcome measures for computing effect sizes. Potential moderating variables of the Schroth exercise included: 1) pre-intervention severity of the scoliosis; 2) duration; and 3) specific types of Schroth exercise.

CONCLUSIONS: The overall effect size of the Schroth exercise is high (g=0.724). In addition, Schroth exercise may be more beneficial for scoliosis patients who have a 10 to 30 degrees Cobb's angle than for those with a greater than 30 degrees Cobb's angle. Patients should practice the exercise for at least one month to have a better effect. Thus, therapists should consider patients' initial curve status and exercise duration before prescribing the Schroth exercise program. Core muscle strength was most influenced, and structural deformity also changed after the Schroth exercise. In sum, the Schroth exercise is a recommended treatment method for scoliosis patients.

C1 [Park, Joo-Hee] Yonsei Univ, Grad Sch, Dept Phys Therapy, 1 Yonseidae Gil, Wonju, South Korea.

[Jeon, Hye-Seon] Yonsei Univ, Coll Hlth Sci, Dept Phys Therapy, Wonju, South Korea.

[Park, Ha-Won] Seoul Natl Univ, Bundang Hosp, Dept Rehabil Med, Seongnam, South Korea.

C3 Yonsei University; Yonsei University; Seoul National University (SNU)

RP Jeon, HS (通讯作者)， Yonsei Univ, Grad Sch, Dept Phys Therapy, 1 Yonseidae Gil, Wonju, South Korea.

EM hyeseonj@yonsei.ac.kr

RI Clark, Richard C/C-6190-2011

FU National Research Foundation of Korea; Korean Government

[NRF-2013S1A-5B8A01055336]

FX This work was supported by a National Research Foundation of Korea

Grant, with funding by the Korean Government (NRF-2013S1A-5B8A01055336).

CR [Anonymous], 2004, SPINAL DEFORMITY STU

[Anonymous], 2014, STAT METHODS METAANA

BECKER BJ, 1988, BRIT J MATH STAT PSY, V41, P257, DOI 10.1111/j.2044-8317.1988.tb00901.x

Bettany-Saltikov J, 2014, EUR J PHYS REHAB MED, V50, P111

Borysov M, 2016, CURR PEDIATR REV, V12, P31, DOI 10.2174/1573396312666151117120746

Borysov M, 2016, CURR PEDIATR REV, V12, P12, DOI 10.2174/1573396312666151117120313

Borysov M, 2012, SCOLIOSIS SPINAL DIS, V7, DOI 10.1186/1748-7161-7-1

Cobb JR., 1948, INSTR COURSE LECT, V5, P261

Cooper H.M., 1998, SYNTHESIZING RES GUI, V2

DEVRIES HA, 1970, J GERONTOL, V25, P325, DOI 10.1093/geronj/25.4.325

Downs SH, 1998, J EPIDEMIOL COMMUN H, V52, P377, DOI 10.1136/jech.52.6.377

Fusco C., 2011, Physiotherapy Theory and Practice, V27, P80, DOI 10.3109/09593985.2010.533342

HwangBo P., 2015, THESIS

Jin Youngwan, 2014, [Korean society for Wellness, 한국웰니스학회], V9, P185

Kong B., 2014, THESIS

Kuru T, 2015, CLIN REHABIL, V2015

Lee SJ, 2015, J STROKE, V17, P67, DOI 10.5853/jos.2015.17.1.67

Lehnert-Schroth C., 2007, 3 DIMENSIONAL TREATM, V7th ed.

Monticone M, 2014, EUR SPINE J, V23, P1204, DOI 10.1007/s00586-014-3241-y

Moramarco M, 2016, CURR PEDIATR REV, V12, P17, DOI 10.2174/1573396312666151117120514

Negrini S, 2008, DISABIL REHABIL, V30, P772, DOI 10.1080/09638280801889568

Negrini S, 2008, DISABIL REHABIL, V30, P731, DOI 10.1080/09638280801889485

정두영, 2014, [The Asian Journal of Kinesiology, 아시아 운동학 학술지], V16, P63

Otman S, 2005, SAUDI MED J, V26, P1429

Park Sang-yong, 2014, [Journal of the Korea Convergence Society, 한국융합학회논문지], V5, P61

Park Sunghee, 2012, THESIS

Pugacheva N, 2012, STUD HEALTH TECHNOL, V176, P365, DOI 10.3233/978-1-61499-067-3-365

Reamy BV, 2001, AM FAM PHYSICIAN, V64, P111

Schreiber S, 2015, SOSORT 2015 AWARD WI, V10, P1

Schroth K, 1931, NATURARZT, P11

Shindle M, 2005, J SURG ORTHOP ADV, V15, P43

Society SR, 2011, ST29112011 SOC MOT P, P1

Weiss H, 2010, INTERNET J REHABIL, V1, P11

Weiss H., 1991, ITAL J ORTHOP TRAUMA, V18, P395

Weiss HR, 2008, EUR J PHYS REHAB MED, V44, P177

Weiss H R, 1997, Pediatr Rehabil, V1, P35

Weiss Hans-Rudolf, 2003, Pediatr Rehabil, V6, P23, DOI 10.1080/1363849031000095288

Weiss HR, 2008, PATIENT SAF SURG, V2, DOI 10.1186/1754-9493-2-25

김정현, 2015, [Journal of Korea Academia-Industrial cooperation Society, 한국산학기술학회논문지], V16, P4098, DOI 10.5762/KAIS.2015.16.6.4098

이준호, 2014, [Journal of the Korean Society of Physical Medicine, 대한물리의학회지], V9, P11

이충일, 2013, [Journal of Coaching Development, 코칭능력개발지], V15, P165

NR 41

TC 38

Z9 45

U1 19

U2 79

PU EDIZIONI MINERVA MEDICA

PI TURIN

PA CORSO BRAMANTE 83-85 INT JOURNALS DEPT., 10126 TURIN, ITALY

SN 1973-9087

EI 1973-9095

J9 EUR J PHYS REHAB MED

JI Eur. J. Phys. Rehabil. Med.

PD JUN

PY 2018

VL 54

IS 3

BP 440

EP +

DI 10.23736/S1973-9087.17.04461-6

PG 13

WC Rehabilitation

WE Science Citation Index Expanded (SCI-EXPANDED)

SC Rehabilitation

GA GU5AO

UT WOS:000445297000013

PM 28976171

DA 2023-08-10

ER

PT J

AU Ridderbusch, K

Spiro, AS

Kunkel, P

Grolle, B

Stucker, R

Rupprecht, M

AF Ridderbusch, Karsten

Spiro, Alexander S.

Kunkel, Philip

Grolle, Benjamin

Stuecker, Ralf

Rupprecht, Martin

TI Strategies for Treating Scoliosis in Early Childhood

SO DEUTSCHES ARZTEBLATT INTERNATIONAL

LA English

DT Review

ID ADOLESCENT IDIOPATHIC SCOLIOSIS; EARLY-ONSET SCOLIOSIS; THORACIC

INSUFFICIENCY SYNDROME; TERM-FOLLOW-UP; NATURAL-HISTORY; CURVE

PROGRESSION; GROWTH; CHILDREN; INFANTILE; MANAGEMENT

AB Background: Scoliosis in early childhood is defined as abnormal curvature of the spine of any etiology that arises before age 10. The affected children are at high risk of developing restrictive pulmonary dysfunction. The treatment presents major challenges because of the complexity and high morbidity of the disease.

Methods: This article is based on pertinent articles retrieved by a selective literature search, and on the results of a retrospective study by the authors.

Results: In addition to conservative treatment methods including physiotherapy, casts, and corsets, progressive scoliosis usually requires early surgical intervention. In recent years, many different so-called non-fusion techniques have been developed for the surgical treatment of early childhood scoliosis. The goal of this new strategy is to avoid early fusion procedures and to enable further growth of the rib cage, lungs, and spine in addition to correcting the scoliosis. The authors also present their own intermediate-term results with a novel growth-preserving spinal operation that exploits magnet technology.

Conclusion: Because of the low prevalence and heterogeneous etiology of early childhood scoliosis, the literature to date contains no randomized controlled therapeutic trials concerning this small group of high-risk patients. For the treatment to succeed, it is essential for specialists from all of the involved medical disciplines to work closely together. Conservative measures such as physiotherapy, casts, and corsets can delay the (frequent) need for surgery or even make surgery unnecessary, particularly in the idiopathic types of early childhood scoliosis. The new non-fusion techniques enable continued growth of the spine, rib cage, and lung in addition to correcting the scoliosis.

C1 [Ridderbusch, Karsten; Spiro, Alexander S.; Stuecker, Ralf; Rupprecht, Martin] Altona Children s Hosp, Dept Pediat Orthoped, Hamburg, Germany.

[Ridderbusch, Karsten; Spiro, Alexander S.; Stuecker, Ralf; Rupprecht, Martin] Univ Med Ctr Hamburg Eppendorf UKE, Dept Orthoped, Hamburg, Germany.

[Kunkel, Philip] Altona Childrens Hosp, Dept Pediat Neurosurg, Hamburg, Germany.

[Grolle, Benjamin] Altona Childrens Hosp, Dept Pediat, Hamburg, Germany.

C3 University of Hamburg; University Medical Center Hamburg-Eppendorf;

University of Hamburg; University Medical Center Hamburg-Eppendorf;

University of Hamburg; University Medical Center Hamburg-Eppendorf

RP Ridderbusch, K (通讯作者)， Altonaer Kinderkrankenhaus, Abt Kinderorthopadie, Bleickenallee 38, D-22763 Hamburg, Germany.

EM karsten.ridderbusch@kinderkrankenhaus.net

RI Kunkel, Philip/HPE-6635-2023

FU Nuvasive

FX Dr. Ridderbusch received reimbursement of travel and accommodation expenses from Orthovative. He received fees for preparing continuing medical education events from Nuvasive.; Prof. Stucker received fees for conference participation and reimbursement of travel and accommodation expenses from Nuvasive. He also received fees from Nuvasive for preparing continuing medical education events.; Dr. Kunkel received fees for preparing continuing medical education events from Nuvasive.

CR Akbarnia BA, 2015, SPINE DEFORM, V3, P105

Akbarnia BA, 2013, SPINE, V38, P665, DOI 10.1097/BRS.0b013e3182773560

Bess S, 2010, J BONE JOINT SURG AM, V92A, P2533, DOI 10.2106/JBJS.I.01471

Boudissa M, 2017, CHILD NERV SYST, V33, P813, DOI 10.1007/s00381-017-3367-4

Burri PH, 1997, LUNG GROWTH DEV, P1

Campbell RM, 2003, J BONE JOINT SURG AM, V85A, P399, DOI 10.2106/00004623-200303000-00001

Campbell RM, 2007, J BONE JOINT SURG AM, V89A, P108, DOI 10.2106/JBJS.F.00270

Cheung KMC, 2012, LANCET, V379, P1967, DOI 10.1016/S0140-6736(12)60112-3

Dannawi Z, 2013, BONE JOINT J, V95B, P75, DOI 10.1302/0301-620X.95B1.29565

Dimeglio A, 2011, J PEDIATR ORTHOPED, V31, pS28, DOI 10.1097/BPO.0b013e318202c25d

EDGAR MA, 1988, J BONE JOINT SURG BR, V70, P712, DOI 10.1302/0301-620X.70B5.3192566

Fernandes P, 2007, J BONE JOINT SURG AM, V89A, P21, DOI 10.2106/JBJS.F.00754

Flynn JM, 2012, J PEDIATR ORTHOPED, V32, P594, DOI 10.1097/BPO.0b013e31826028ea

Helfenstein A, 2006, SPINE, V31, P339, DOI 10.1097/01.brs.0000197412.70050.0d

Karol LA, 2008, J BONE JOINT SURG AM, V90A, P1272, DOI 10.2106/JBJS.G.00184

Katz DE, 2010, J BONE JOINT SURG AM, V92A, P1343, DOI 10.2106/JBJS.I.01142

Koumbourlis AC, 2014, PAEDIATR RESPIR REV, V15, P246, DOI 10.1016/j.prrv.2013.12.003

LLOYD-ROBERTS G C, 1965, J Bone Joint Surg Br, V47, P520

MCMASTER MJ, 1979, J BONE JOINT SURG BR, V61, P36, DOI 10.1302/0301-620X.61B1.422633

Mehta HP, 2010, SPINE, V35, P153, DOI 10.1097/BRS.0b013e3181c4b8c7

Mehta MH, 2005, J BONE JOINT SURG BR, V87B, P1237, DOI 10.1302/0301-620X.87B9.16124

MUIRHEAD A, 1985, J BONE JOINT SURG BR, V67, P699, DOI 10.1302/0301-620X.67B5.4055863

PEHRSSON K, 1992, SPINE, V17, P1091, DOI 10.1097/00007632-199209000-00014

Ridderbusch K, 2013, ORTHOPADE, V42, P1030, DOI 10.1007/s00132-012-2049-x

Ridderbusch K, 2017, J PEDIATR ORTHOPED, V37, pE575, DOI 10.1097/BPO.0000000000000752

Rigo M, 2009, STUD HEALTH TECHNOL, V135, P208

Sankar WN, 2010, SPINE, V35, P2091, DOI 10.1097/BRS.0b013e3181c6edd7

Schreiber S, 2015, SCOLIOSIS SPINAL DIS, V10, DOI 10.1186/s13013-015-0048-5

Skaggs D., 2015, SPINE DEFORM, V3, P107, DOI [10.1016/j.jspd.2015.01.002, DOI 10.1016/J.JSPD.2015.01.002]

Sponseller PD, 2007, SPINE, V32, pS81, DOI 10.1097/BRS.0b013e3181453073

Stucker R, 2016, ORTHOPADE, V45, P534, DOI 10.1007/s00132-016-3277-2

Trobisch P, 2010, DTSCH ARZTEBL INT, V107, P875, DOI 10.3238/arztebl.2010.0875

WEINSTEIN SL, 1983, J BONE JOINT SURG AM, V65, P447, DOI 10.2106/00004623-198365040-00004

WEINSTEIN SL, 1986, SPINE, V11, P780, DOI 10.1097/00007632-198610000-00006

Weinstein SL, 1999, SPINE, V24, P2592, DOI 10.1097/00007632-199912150-00006

Weinstein SL, 2013, SPINE, V38, P1832, DOI 10.1097/01.brs.0000435048.23726.3e

Weinstein SL, 2013, NEW ENGL J MED, V369, P1512, DOI 10.1056/NEJMoa1307337

Weiss H-R, 2006, Pediatr Rehabil, V9, P190, DOI 10.1080/13638490500079583

Wilkinson JT, 2017, J PEDIAT ORTHOP, DOI 10.1097/BPO.0000000000000983

Zhang W, 2016, BMC MUSCULOSKEL DIS, V17, DOI 10.1186/s12891-016-1026-7

NR 40

TC 8

Z9 10

U1 2

U2 18

PU DEUTSCHER AERZTE-VERLAG GMBH

PI COLOGNE

PA DIESELSTRABE 2, POSTFACH 400265, D-50859 COLOGNE, GERMANY

SN 1866-0452

J9 DTSCH ARZTEBL INT

JI Dtsch. Arztebl. Int.

PD JUN 1

PY 2018

VL 115

IS 22

BP 371

EP +

DI 10.3238/arztebl.2018.0371

PG 7

WC Medicine, General & Internal

WE Science Citation Index Expanded (SCI-EXPANDED)

SC General & Internal Medicine

GA GK2JW

UT WOS:000435956700003

PM 29932047

OA Green Published

DA 2023-08-10

ER

PT J

AU Zheng, Y

Dang, YN

Yang, Y

Li, HB

Zhang, LJ

Lou, EHM

He, CQ

Wong, MS

AF Zheng, Yu

Dang, Yini

Yang, Yan

Li, Huabo

Zhang, Lijie

Lou, Edmond H. M.

He, Chengqi

Wong, Mansang

TI Whether Orthotic Management and Exercise are Equally Effective to the

Patients With Adolescent Idiopathic Scoliosis in Mainland China? A

Randomized Controlled Trial Study

SO SPINE

LA English

DT Article

DE adolescent idiopathic scoliosis; body symmetry; cobb angle; esthetics;

mental health; quality of life; scoliosis-specific exercise; shoulder

balance; spinal curvature; spinal orthoses

ID QUALITY-OF-LIFE; TRUNK APPEARANCE; SHOULDER BALANCE; SPINAL ORTHOSIS;

BRACE; STRESS; PERCEPTION; EFFICACY; HEALTH

AB Study Design. A prospective randomized controlled trial.

Objective. The aim of this study was to investigate the effectiveness of orthotic management versus exercise on spinal curvature, body symmetry, and quality of life.

Summary of Background Data. A number of well-designed studies comparing conservative treatment of adolescent idiopathic scoliosis (AIS) have been conducted and the evidence becomes stronger. However, there is a lack of the information on the effectiveness of orthotic management versus exercise.

Methods. The inclusion criteria recommended by the Scoliosis Research Society (SRS) and the international Society on Scoliosis Orthopedic and Rehabilitation Treatment (SOSORT) were used during enrollment. Eligible patients were randomly assigned to either bracing group or exercise group. Patients in the bracing group were prescribed with a rigid thoracolumbosacral orthosis and requested to wear 23 h/day, while patients in the exercise group were treated with the protocol of the Scientific Exercise Approach to Scoliosis. Data regarding angle of trunk inclination, Cobb angle, shoulder balance, body image, and quality of life (QoL) were collected every 6 months.

Results. Twenty-four patients in the bracing group and 29 patients in the exercise group participated in this study. For the intergroup comparison, the bracing group showed better results about the correction of spinal curvature (Cobb angle at the first 12 months of intervention, $P = 0.039$), scores concerning QoL, especially function ($P < 0.001$), mental health ($P < 0.001$), and total score ($P < 0.001$), were higher than that of the exercise group. The results of body symmetry evaluation did not differ significantly between the two groups. For the intragroup comparison, parameters of spinal curvature (baseline vs. 12-month, $P < 0.03$ in the exercise group and $P < 0.001$ in the bracing group), QoL (baseline vs. 12-month, $P < 0.001$), and TAPS (baseline vs. 12-month, $P < 0.033$) significantly improved over the studied period. Shoulder balance (baseline vs. 12-month, $P < 0.005$) showed significant improvement only in the bracing group.

Conclusion. Both interventions of bracing and exercise showed significant treatment effectiveness on the patients with AIS. Bracing was superior to capture corrections in parameters of spinal curvature and body symmetry, while the QoL, especially in aspect of the functional and psychological status, was significantly better in the exercise group.

C1 [Zheng, Yu; Wong, Mansang] Hong Kong Polytech Univ, Interdisciplinary Div Biomed Engn, Hong Kong 999077, Hong Kong, china.

[Zheng, Yu; He, Chengqi] Sichuan Univ, West China Hosp, Ctr Rehabil Med, 37 Guo Xue Xiang, Chengdu 610041, Sichuan, china.

[Zheng, Yu; He, Chengqi] Sichuan Univ Hong Kong Polytech Univ, Inst Disaster Management & Reconstruct, Chengdu, Sichuan, china.

[Dang, Yini] Nanjing Med Univ, Dept Gastroenterol, Affiliated Hosp 1, Nanjing, Jiangsu, china.

[Zheng, Yu; Yang, Yan; Li, Huabo; Zhang, Lijie] Wuxi Rehabil Hosp, Dept Rehabil Med, Wuxi, Jiangsu, china.

[Lou, Edmond H. M.] Univ Alberta, Dept Surg, Glenrose Rehabil Res Ctr, Edmonton, AB, Canada.

C3 Hong Kong Polytechnic University; Sichuan University; Hong Kong

Polytechnic University; Nanjing Medical University; University of

Alberta

RP Wong, MS (通讯作者)， Hong Kong Polytech Univ, Interdisciplinary Div Biomed Engn, Hong Kong 999077, Hong Kong, china.; He, CQ (通讯作者)， Sichuan Univ, West China Hosp, Ctr Rehabil Med, 37 Guo Xue Xiang, Chengdu 610041, Sichuan, china.

EM hxkfhcq2015@126.com; m.s.wong@polyu.edu.hk

OI ZHENG, Yu/0000-0003-4891-5472; WONG, MS/0000-0002-4157-9528

FU Wuxi Science and Technology Program (WSTP), China [ZD201408]

FX Wuxi Science and Technology Program (WSTP), China (Grant number:

ZD201408) funds were received in support of this work.

CR Akel I, 2008, EUR SPINE J, V17, P348, DOI 10.1007/s00586-007-0546-0

[Anonymous], CHIN J CLIN REHABIL

Bago J, 2010, SCOLIOSIS SPINAL DIS, V5, DOI 10.1186/1748-7161-5-6

Bago J, 2009, EUR SPINE J, V18, P1898, DOI 10.1007/s00586-009-1066-x

Bettany-Saltikov J, 2014, EUR J PHYS REHAB MED, V50, P111

Carrasco MIB, 2014, REV ESC ENFERM USP, V48, P748, DOI 10.1590/S0080-623420140000400024

Bunge EM, 2010, EUR SPINE J, V19, P747, DOI 10.1007/s00586-010-1337-6

Chan SL, 2014, SCOLIOSIS SPINAL DIS, V9, DOI 10.1186/1748-7161-9-1

CLAYSON D, 1987, SPINE, V12, P983, DOI 10.1097/00007632-198712000-00007

Cobb JR., 1948, INSTR COURSE LECT, V5, P261

Coelho DM, 2013, BRAZ J PHYS THER, V17, P179, DOI 10.1590/S1413-35552012005000081

Coillard C, 2014, EUR J PHYS REHAB MED, V50, P479

Danielsson AJ, 2001, SPINE, V26, P516

FALLSTROM K, 1986, SPINE, V11, P756

Freidel K, 2002, SPINE, V27, pE87, DOI 10.1097/00007632-200202150-00013

Hong JY, 2013, SPINE, V38, pE1684, DOI 10.1097/BRS.0b013e3182a18486

Hresko MT, 2013, NEW ENGL J MED, V368, P834, DOI 10.1056/NEJMcp1209063

Korbel K, 2013, POLISH ORTHOP TRAUMA, V79, P118

Kotwicki T, 2007, SCOLIOSIS SPINAL DIS, V2, DOI 10.1186/1748-7161-2-1

Kuru T, 2016, CLIN REHABIL, V30, P181, DOI 10.1177/0269215515575745

Li M, 2009, SPINE, V34, P1321, DOI 10.1097/BRS.0b013e31819812b7

Lonstein JE, 2006, CLIN ORTHOP RELAT R, P248, DOI 10.1097/01.blo.0000198725.54891.73

Lou E, 2012, STUD HEALTH TECHNOL, V176, P338, DOI 10.3233/978-1-61499-067-3-338

MACLEAN WE, 1989, J PEDIATR ORTHOPED, V9, P257

Misterska E, 2013, QUAL LIFE RES, V22, P1633, DOI 10.1007/s11136-012-0316-2

Negrini S, 2016, SPINE, V41, P1813, DOI 10.1097/BRS.0000000000001887

Negrini S, 2012, SCOLIOSIS SPINAL DIS, V7, DOI 10.1186/1748-7161-7-3

Negrini S, 2015, SCOLIOSIS SPINAL DIS, V10, DOI 10.1186/s13013-014-0025-4

Parent Stefan, 2005, Instr Course Lect, V54, P529

Qiu Yong, 2008, Chinese Journal of Orthopaedics, V28, P459

Richards BS, 2005, SPINE, V30, P2068, DOI 10.1097/01.brs.0000178819.90239.d0

Romano M, 2015, SCOLIOSIS SPINAL DIS, V10, DOI 10.1186/s13013-014-0027-2

Romano M, 2012, COCHRANE DB SYST REV, DOI 10.1002/14651858.CD007837.pub2

Smania N, 2008, DISABIL REHABIL, V30, P763, DOI 10.1080/17483100801921311

Vasiliadis E, 2009, STUD HEALTH TECHNOL, V135, P409

Weinstein SL, 2013, SPINE, V38, P1832, DOI 10.1097/01.brs.0000435048.23726.3e

Weinstein SL, 2013, NEW ENGL J MED, V369, P1512, DOI 10.1056/NEJMoa1307337

Weiss HR, 2007, SCOLIOSIS SPINAL DIS, V2, DOI 10.1186/1748-7161-2-10

Wong MS, 2008, SPINE, V33, P1360, DOI 10.1097/BRS.0b013e31817329d9

Zhang HQ, 2015, SPINE, V40, P41, DOI 10.1097/BRS.0000000000000664

NR 40

TC 25

Z9 26

U1 2

U2 34

PU LIPPINCOTT WILLIAMS & WILKINS

PI PHILADELPHIA

PA TWO COMMERCE SQ, 2001 MARKET ST, PHILADELPHIA, PA 19103 USA

SN 0362-2436

EI 1528-1159

J9 SPINE

JI SPINE

PD MAY 1

PY 2018

VL 43

IS 9

BP E494

EP E503

DI 10.1097/BRS.0000000000002412

PG 10

WC Clinical Neurology; Orthopedics

WE Science Citation Index Expanded (SCI-EXPANDED)

SC Neurosciences & Neurology; Orthopedics

GA GP0TM

UT WOS:000440523400002

PM 28885287

DA 2023-08-10

ER

PT J

AU Nisser, J

Smolenski, UC

Sliwinski, GE

Kruger, P

Heinke, A

Malberg, H

Werner, M

Drossel, WG

Sliwinski, Z

Derlien, S

AF Nisser, Jenny

Smolenski, Ulrich Christian

Sliwinski, Grzegorz Eugeniusz

Krueger, Paula

Heinke, Andreas

Malberg, Hagen

Werner, Michael

Drossel, Welf-Guntram

Sliwinski, Zbigniew

Derlien, Steffen

TI Scoliosis Specific Physiotherapy Approach to Adolescent Idiopathic

Scoliosis (AIS) - A Narrative Review

SO PHYSIKALISCHE MEDIZIN REHABILITATIONSMEDIZIN KURORTMEDIZIN

LA German

DT Review

DE adolescent idiopathic scoliosis (AIS); scoliosis specific physiotherapy;

conservative treatment

ID OF-THE-LITERATURE; CONSERVATIVE TREATMENT; PHYSICAL-THERAPY; EXERCISE

THERAPY; EFFICACY

AB Zusammenfassung

Fragestellung Die Arbeit soll einen aktuellen uberblick uber die konservativen Therapieverfahren (skoliosespezifischen Physiotherapie) bei Patienten mit idiopathischer Adoleszentenskoliose (AIS) im Allgemeinen liefern. Im Speziellen ist die FED-Methode (Fixation, Elongation, Derotation), welche Inhalt eines Forschungsprojektes (Forderkennzeichen: 19200 BR/3) ist, Gegenstand der Recherche.

Material und Methoden Diese narrative Literaturrecherche wurde in den Zeitraum von Nov. 2016 bis Jul. 2017 durchgefuhrt. Die erste Recherche erzielt die Auflistung der Therapieverfahren fur Skoliosepatienten, wahrend der zweite Teil der Charakterisierung dieser Verfahren dient. Ergebnisse Insgesamt wurden 34 relevante Literaturstellen identifiziert und der Volltextanalyse unterzogen. Es existieren Behandlungsverfahren mit und ohne Autokorrektur. Zu den Behandlungsverfahren mit Autokorrektur zahlen die folgenden Konzepte: Schroth-Methode, Side Shift Program, SEAS-

Program, DoboMed, Lyon School, BSPTS, FITS und teilweise Klapp'sches Kriechen. Die Verfahren Vojta-Therapie, PEP-Programm, Hanke-Konzept E-Technik (R), Bobath-Konzept, PNF und Riemenubungen gehoren zu den Behandlungsverfahren ohne Autokorrektur.

Diskussion Die Schroth-Methode ist ein grundlegendes Therapieverfahren, woraus sich weitere Verfahren ableiteten. Zur Charakterisierung der FED-Methode (Fixation, Elongation, Derotation) wurde keine Referenz gefunden. Dieses Behandlungsverfahren wurde lediglich kurz in einer Arbeit erwahnt.

Schlussfolgerung Verschiedene Verfahren werden bei AIS angewendet. Diese Arbeit bietet einen uberblick uber diese Therapieverfahren. Weitere Untersuchungen sollten sich mit den Wirkmechanismen der die FED-Methode befassen.

Abstract

Purpose In general, the purpose is to give a currently overview about conservative treatment methods (scoliosis specific physiotherapy) applied to treat adolescent idiopathic scoliosis (AIS). In particular, the FED-Method (Fixation, Elongation, Derotation), which is within the scope of a research project (Project Number: 19200 BR/3), is an object of the present paper.

Materials and Methods This narrative literature research was conducted in Nov 2016-Jul 2017. The first part of the literature research aims for a listing of the applied treatment procedures for patients with AIS. Whereas, the second part aims for the characterization of these procedures. Results In total, 34 relevant references were identified and analyzed. There are procedures with and without autocorrection. The procedures with autocorrection are: Schroth-Methode, Side Shift Program, SEAS-Program, DoboMed, Lyon School, BSPTS, FITS and partly Klapp'sches Kriechen. The procedurce without autocorrection are: Vojta-Therapie, PEP-Programm, Hanke-Konzept E-Technik (R), Bobath-Konzept, PNF and Riemenubungen.

Discussion The Schorth-Method is a fundamental treatment procedure, from which other procedures were deduced. No reference was found, which characterized the FED-Method. There was only a mention.

Conclusions Various treatment procedures are applied for patients with AIS. This article gives an overview of these procedures. Further investigations should address the mode of action of the FED-Methode.

C1 [Nisser, Jenny; Smolenski, Ulrich Christian; Derlien, Steffen] Univ Klinikum Jena, Inst Physiotherapie, Klinikum 1, D-07743 Jena, Germany.

[Sliwinski, Grzegorz Eugeniusz; Krueger, Paula; Heinke, Andreas; Malberg, Hagen] Tech Univ Dresden, Inst Biomed Tech, Dresden, Germany.

[Werner, Michael; Drossel, Welf-Guntram] Fraunhofer Inst Werkzeugmaschinen & Umformtech, Abt Med Tech, Dresden, Germany.

[Sliwinski, Zbigniew] Uniwersytet Jana Kochanowskiego Kielcach, Fac Med & Hlth Sci, Kielce, Poland.

C3 Friedrich Schiller University of Jena; Technische Universitat Dresden;

Fraunhofer Gesellschaft; Jan Kochanowski University

RP Nisser, J (通讯作者)， Univ Klinikum Jena, Inst Physiotherapie, Klinikum 1, D-07743 Jena, Germany.

EM jenny.nisser@med.uni-jena.de

RI Sliwiński, Zbigniew/O-2678-2014

OI Sliwinski, Zbigniew/0000-0001-7402-1793

CR Berdishevsky H, 2016, SCOLIOSIS SPINAL DIS, V11, DOI 10.1186/s13013-016-0076-9

Bettany-Saltikov J, 2014, EUR J PHYS REHAB MED, V50, P111

Bettany-Saltikov J, 2015, COCHRANE LIB

Bialek M, 2011, SCOLIOSIS SPINAL DIS, V6, DOI 10.1186/1748-7161-6-25

Boni T, 2002, ORTHOPADE, V31, P11, DOI 10.1007/s132-002-8270-4

Dantas Diego De Sousa, 2017, J Phys Ther Sci, V29, P1, DOI 10.1589/jpts.29.1

DGOOC (Deutsche Gesellschaft fur Orthopadie und Orthopadische Chirurgie BdFO BOV (Berufsverband der Arzte fur Orthopadie)., 2009, DTSCH GES ORTH ORTH

Dobosiewicz K, 2007, STUDIES HLTH TECHNOL, V135, P228

Durmala J, 2009, SCOLIOSIS S2, V4, pO29

Fusco C., 2011, Physiotherapy Theory and Practice, V27, P80, DOI 10.3109/09593985.2010.533342

Iunes DH, 2010, BRAZ J PHYS THER, V14, P133, DOI 10.1590/S1413-35552010005000009

Kolster BC, 2017, HDB PHYSIOTHERAPIE U

Kuru T, 2015, CLIN REHABIL

Lee BK, 2016, J EXERC REHABIL, V12, P567, DOI 10.12965/jer.1632796.398

Mamyama T, 2001, STUDIES HLTH TECHNOL, V91, P361

Matussek J, 2014, ORTHOPADE, V43, P689, DOI 10.1007/s00132-014-2307-1

Matussek J, 2013, ERKRANKUNGEN KINDLIC, P937

Mordecai SC, 2012, EUR SPINE J, V21, P382, DOI 10.1007/s00586-011-2063-4

Negrini S, 2008, DISABIL REHABIL, V30, P772, DOI 10.1080/09638280801889568

Negrini S, 2012, SCOLIOSIS SPINAL DIS, V7, DOI 10.1186/1748-7161-7-3

Oldevig J M, 1913, A T EIN NEUES GERAT, P9

Otman S, 2005, SAUDI MED J, V26, P1429

Romano M, 2012, EXERCISES ADOLESCENT

Romano M, 2015, SCOLIOSIS SPINAL DIS, V10, DOI 10.1186/s13013-014-0027-2

Rother Edna Terezinha, 2007, Acta paul. enferm., V20, pv, DOI 10.1590/S0103-21002007000200001

Sandel ME, 2013, PM&R, V5, P453, DOI 10.1016/j.pmrj.2013.04.020

Sliwinski Z, 2014, SCOLIOSIS SPINAL DIS, V9, pP13, DOI [10.1186/1748-7161-9-S1-P13, DOI 10.1186/1748-7161-9-S1-P13]

Sliwinski Z., 2014, SCOLIOSIS SPINAL DIS, V9, pP15, DOI [10.1186/1748-7161-9-S1-P15, DOI 10.1186/1748-7161-9-S1-P15]

Sliwinski Z, 2014, SCOLIOSIS SPINAL DIS, V9, P14

Steffan K, 2015, ORTHOPADE, V44, P852, DOI 10.1007/s00132-015-3174-0

Stucker R., 2010, ORTHOP DIE UNF UP2DA, V5, P39, DOI [10.1055/s-0029-1243953, DOI 10.1055/S-0029-1243953]

Weiβ H-R., 1994, PRINZIPIEN ERGEBNISS

Weiss Hans-Rudolf, 2003, Pediatr Rehabil, V6, P183

Weiss HR, 2011, SCOLIOSIS SPINAL DIS, V6, DOI 10.1186/1748-7161-6-17

Weiss HR, 2003, ORTHOPADE, V32, P146, DOI 10.1007/s00132-002-0430-x

WEISS HR, 1995, Z ORTHOP GRENZGEB, V133, P114, DOI 10.1055/s-2008-1039421

WEISS HR, 2000, SKOLIOSEREHABILITATI

Wnuk B, 2012, SCOLIOSIS, V7, pO58

Zapata K, 2016, SCOLIOSIS SPINAL DIS, V11, DOI 10.1186/s13013-016-0101-z

NR 39

TC 2

Z9 2

U1 4

U2 47

PU GEORG THIEME VERLAG KG

PI STUTTGART

PA RUDIGERSTR 14, D-70469 STUTTGART, GERMANY

SN 0940-6689

EI 1439-085X

J9 PHYS MED REHAB KUROR

JI Physik. Med. Rehabilitationsmed. Kurort.

PD APR

PY 2018

VL 28

IS 2

BP 88

EP 102

DI 10.1055/s-0043-124472

PG 15

WC Rehabilitation; Sport Sciences

WE Science Citation Index Expanded (SCI-EXPANDED)

SC Rehabilitation; Sport Sciences

GA GD5PD

UT WOS:000430558000002

DA 2023-08-10

ER

PT J

AU Hedayati, Z

Ahmadi, A

Kamyab, M

Babaee, T

Ganjavian, MS

AF Hedayati, Zahra

Ahmadi, Amir

Kamyab, Mojtaba

Babaee, Taher

Ganjavian, Mohammad Saleh

TI Effect of Group Exercising and Adjusting the Brace at Shorter Intervals

on Cobb Angle and Quality of Life of Patients With Idiopathic Scoliosis

SO AMERICAN JOURNAL OF PHYSICAL MEDICINE & REHABILITATION

LA English

DT Article

DE Idiopathic Scoliosis; Quality of Life; Scoliosis Brace; Group Exercises

ID MILWAUKEE BRACE; SRS-22 QUESTIONNAIRE; CHENEAU BRACE; ADOLESCENT

AB Objective The aim of the study was to evaluate the effect of group exercise with brace adjustment at shorter intervals than used in routine practice in late-onset idiopathic scoliosis patients.

Design This was a quasi-experimental study. Thirty patients with progressive scoliosis curves of 15-50 degrees and a prescription for a brace were divided into experimental and control groups, both of which participated in an 11-wk treatment program. Those in the experimental group underwent brace adjustment twice per week and performed group exercise, whereas those in the control group received a routine protocol. The quality of life and Cobb angle of patients in both groups were evaluated based on baseline and final results of the 22-item Scoliosis Research Society questionnaire and primary and secondary radiographs.

Results In the experimental group, the improvement in Cobb angle and patient satisfaction was greater than that in the control group ($P < 0.05$). Moreover, in patients with Cobb angles of less than 30 degrees, the self-image and satisfaction domains and the total scores of patients in the

experimental group were significantly different from those in the control group at the final assessment ($P < 0.05$).

Conclusions Brace adjustment at shorter intervals combined with group exercise increases patient satisfaction and reduces scoliosis Cobb angles.

C1 [Hedayati, Zahra; Kamyab, Mojtaba; Babaee, Taher] Iran Univ Med Sci, Sch Rehabil Sci, Dept Orthot & Prosthet, Tehran, Iran.

[Ahmadi, Amir] Iran Univ Med Sci, Sch Rehabil Sci, Dept Physiotherapy, Madadkaran Ave,Shahnazari St,Madar Sq,Mirdamad, Tehran, Iran.

[Ganjavian, Mohammad Saleh] Iran Univ Med Sci, Shafa Yahyaiian Hosp, Dept Orthopaed Surg, Tehran, Iran.

C3 Iran University of Medical Sciences; Iran University of Medical

Sciences; Iran University of Medical Sciences

RP Ahmadi, A (通讯作者)， Iran Univ Med Sci, Sch Rehabil Sci, Dept Physiotherapy, Madadkaran Ave,Shahnazari St,Madar Sq,Mirdamad, Tehran, Iran.

RI Kehoe, Clare/ABC-7984-2020; babaee, taher/I-2025-2018; hedayati,

zahra/AAG-1613-2019; Kamyab, Mojtaba/AAA-5537-2019

OI babaee, taher/0000-0002-1487-2374; Kamyab, Mojtaba/0000-0003-1081-3083

FU Iran University of Medical Sciences

FX This research did not receive any specific grant from funding agencies

in the public, commercial, or not-for-profit sectors. This study was

supported by the research committee of Iran University of Medical

Sciences.

CR [Anonymous], THESIS

Babaee T, 2014, CURR ORTHOP PRACT, V25, P478, DOI 10.1097/BCO.0000000000000138

Biddle SJ, 2007, PSYCHOL PHYS ACTIVIT, P137

CASSELLA MC, 1991, PHYS THER, V71, P897, DOI 10.1093/ptj/71.12.897

Chan A, 2013, J CHILD ORTHOP, V7, P309, DOI 10.1007/s11832-013-0500-0

Cheung KMC, 2007, INT ORTHOP, V31, P507, DOI 10.1007/s00264-006-0209-5

Du Q, 2013, CHIN J REHABIL MED, V28, P10

Friedrich M, 1996, PHYS THER, V76, P1082, DOI 10.1093/ptj/76.10.1082

Ganjavian Mohammad Saleh, 2011, Acta Med Iran, V49, P598

GRATZ RR, 1984, J ADOLESCENT HEALTH, V5, P237, DOI 10.1016/S0197-0070(84)80124-2

Haher TR, 1999, SPINE, V24, P1435, DOI 10.1097/00007632-199907150-00008

Katz D. E, 2008, AAOS ATLAS ORTHOSES, P125

LONSTEIN JE, 1994, J BONE JOINT SURG AM, V76A, P1207, DOI 10.2106/00004623-199408000-00011

Lusini M, 2014, SPINE J, V14, P1951, DOI 10.1016/j.spinee.2013.11.040

Moe JH, 1973, MILWAUKEE BRACE

Mousavi SJ, 2010, SPINE, V35, P784, DOI 10.1097/BRS.0b013e3181bad0e8

Negrini S, 2006, SCOLIOSIS SPINAL DIS, V1, DOI 10.1186/1748-7161-1-4

Negrini S, 2014, BMC MUSCULOSKEL DIS, V15, DOI 10.1186/1471-2474-15-263

Pham V. M., 2008, Annales de Readaptation et de Medecine Physique, V51, P9, DOI 10.1016/j.annrmp.2007.08.007

Pham V. M., 2008, Annales de Readaptation et de Medecine Physique, V51, P3, DOI 10.1016/j.annrmp.2007.08.008

Wiley JW, 2000, SPINE, V25, P2326, DOI 10.1097/00007632-200009150-00010

Zheng X, 2012, EUR SPINE J, V21, P1157, DOI 10.1007/s00586-012-2258-3

NR 22

TC 8

Z9 8

U1 2

U2 25

PU LIPPINCOTT WILLIAMS & WILKINS

PI PHILADELPHIA

PA TWO COMMERCE SQ, 2001 MARKET ST, PHILADELPHIA, PA 19103 USA

SN 0894-9115

EI 1537-7385

J9 AM J PHYS MED REHAB

JI Am. J. Phys. Med. Rehabil.

PD FEB

PY 2018

VL 97

IS 2

BP 104

EP 109

DI 10.1097/PHM.0000000000000812

PG 6

WC Rehabilitation; Sport Sciences

WE Science Citation Index Expanded (SCI-EXPANDED)

SC Rehabilitation; Sport Sciences

GA FU3DE

UT WOS:000423729500008

PM 28816707

DA 2023-08-10

ER

PT J

AU Dunn, J

Henrikson, NB

Morrison, CC

Blasi, PR

Nguyen, M

Lin, JS

AF Dunn, John

Henrikson, Nora B.

Morrison, Caitlin C.

Blasi, Paula R.

Nguyen, Matt

Lin, Jennifer S.

TI Screening for Adolescent Idiopathic Scoliosis Evidence Report and

Systematic Review for the US Preventive Services Task Force

SO JAMA-JOURNAL OF THE AMERICAN MEDICAL ASSOCIATION

LA English

DT Review

ID QUALITY-OF-LIFE; RANDOMIZED CONTROLLED-TRIAL; LONG-TERM TREATMENT;

NATURAL-HISTORY; FOLLOW-UP; CURVE PROGRESSION; BRACING PATIENTS;

CHILDREN; RECOMMENDATIONS; PREVALENCE

AB IMPORTANCE Adolescent idiopathic scoliosis (AIS), a spinal curvature of 10 degrees or more, is the most common form of scoliosis, with a prevalence of 1% to 3%. Curves progress in approximately two-thirds of patients with AIS before skeletal maturity, and large curves (>50 degrees) may be associated with adverse health outcomes.

OBJECTIVE To systematically review evidence on benefits and harms of AIS screening for the US Preventive Services Task Force (USPSTF).

DATA SOURCES Cochrane Central Register of Controlled Trials, MEDLINE, ERIC, PubMed, CINAHL, and relevant systematic reviews were searched for studies published from January 1966 to October 20, 2016; studies included in a previous USPSTF report were also reviewed. Surveillance was conducted through July 24, 2017.

STUDY SELECTION Fair-and good-quality studies that evaluated the accuracy of screening children and adolescents aged 10 to 18 years for AIS, the benefits of AIS treatment, the harms of AIS screening or treatment, or long-term health outcomes.

DATA EXTRACTION AND SYNTHESIS Two investigators independently reviewed abstracts and full-text articles and extracted data into evidence tables. Results were qualitatively summarized.

MAIN OUTCOMES AND MEASURES Health outcomes and spinal curvature in adolescence and adulthood, accuracy of screening for AIS, any harm of AIS screening or treatment.

RESULTS Fourteen studies (N = 448 276) in 26 articles were included. Accuracy of AIS screening was highest (93.8% sensitivity; 99.2% specificity) in a cohort study of a clinic-based program using forward bend test, scoliometer, and Moire topography screening (n = 306 082); accuracy was lower in cohort studies of 6 programs using fewer modalities (n = 141 161). Four controlled studies (n = 587) found evidence for benefit of bracing on curve progression compared with controls. A randomized clinical trial and a nonrandomized trial of exercise treatment (N = 184) found favorable reductions in Cobb angle of 0.67 degrees to 4.9 degrees in the intervention group compared with increases of 1.38 degrees to 2.8 degrees in the control group. Two cohort studies (n = 339) on long-term outcomes found that braced participants reported more negative treatment experience and body appearance compared with surgically treated or untreated participants. A study that combined a randomized clinical trial and cohort design (n = 242) reported harms of bracing, which included skin problems on the trunk and nonback body pains. There was no evidence on the effect of AIS screening on adult health outcomes.

CONCLUSIONS AND RELEVANCE Screening can detect AIS. Bracing and possibly exercise treatment can interrupt or slow progression of curvature in adolescence. However, there is little or no evidence on long-term outcomes for AIS treated in adolescence, the association between curvature at skeletal maturity and adult health outcomes, the harms of AIS screening or treatment, or the effect of AIS screening on adult health outcomes.

C1 [Dunn, John; Henrikson, Nora B.; Morrison, Caitlin C.; Blasi, Paula R.; Nguyen, Matt] Kaiser Permanente Washington Hlth Res Inst, Kaiser Permanente Res Affiliates Evidence Based P, Seattle, WA 98101 USA.

[Lin, Jennifer S.] Kaiser Permanente Ctr Hlth Res, Kaiser Permanente Res Affiliates Evidence Based P, Portland, OR USA.

C3 Kaiser Permanente; Kaiser Permanente

RP Dunn, J (通讯作者)，Kaiser Permanente Washington Hlth Res Inst, 1730 Minor Ave, Seattle, WA 98101 USA.

EM dunn.jb@ghc.org

RI Kehoe, Clare/ABC-7984-2020

OI Blasi, Paula/0000-0002-7094-706X

FU Agency for Healthcare Research and Quality (AHRQ), US Department of

Health and Human Services [HHSA-290-2012-00015-I-357 EPC4]

FX This research was funded under contract HHSA-290-2012-00015-I-357 EPC4,

Task Order 6, from the Agency for Healthcare Research and Quality
(AHRQ), US Department of Health and Human Services, under a contract to
support the USPSTF.

CR Adobor RD, 2011, SCOLIOSIS SPINAL DIS, V6, DOI 10.1186/1748-7161-6-23
[Anonymous], AHRQ PUBLICATION
ASCANI E, 1986, SPINE, V11, P784, DOI 10.1097/00007632-198610000-00007
Asher MA, 2006, SCOLIOSIS SPINAL DIS, V1, DOI 10.1186/1748-7161-1-2
Atkins D, 2004, BMC HEALTH SERV RES, V4, DOI 10.1186/1472-6963-4-38
Bunge EM, 2010, EUR SPINE J, V19, P747, DOI 10.1007/s00586-010-1337-6
Bunge EM, 2008, BMC MUSCULOSKEL DIS, V9, DOI 10.1186/1471-2474-9-57
BUNNELL WP, 1986, SPINE, V11, P773, DOI 10.1097/00007632-198610000-00003
Canavese F, 2011, INDIAN J ORTHOP, V45, P7, DOI 10.4103/0019-5413.73655
Cobb JR, 1948, OUTLINE STUDY SCOLIO
Coillard C, 2014, EUR J PHYS REHAB MED, V50, P479
Danielsson AJ, 2012, SPINE, V37, P755, DOI 10.1097/BRS.0b013e318231493c
Danielsson AJ, 2010, SPINE, V35, P199, DOI 10.1097/BRS.0b013e3181c89f4a
Danielsson AJ, 2001, SPINE, V26, P516
Danielsson AJ, 2001, EUR SPINE J, V10, P278, DOI 10.1007/s005860100309
Danielsson AJ, 2001, SPINE, V26, P1449, DOI 10.1097/00007632-200107010-00015
Doody MM, 2000, SPINE, V25, P2052
DUNN J, 2017, SCREENING ADOLESCENT
Fong DYT, 2015, SPINE J, V15, P825, DOI 10.1016/j.spinee.2015.01.019
Fong DYT, 2010, SPINE, V35, P1061, DOI 10.1097/BRS.0b013e3181bcc835
Goldberg C J, 1993, Eur Spine J, V2, P29, DOI 10.1007/BF00301052
GOLDBERG CJ, 1995, SPINE, V20, P1368, DOI 10.1097/00007632-199506000-00007

GOLDBERG CJ, 1993, SPINE, V18, P902, DOI 10.1097/00007632-199306000-00015

GOLDSTEIN LA, 1973, CLIN ORTHOP RELAT R, P10

Himmetoglu S, 2015, MINERVA PEDIATR, V67, P245

Hresko MT, 2015, POSITION STATEMENT S

Karachalios T, 1999, SPINE, V24, P2318, DOI 10.1097/00007632-199911150-00006

Konieczny MR, 2013, J CHILD ORTHOP, V7, P3, DOI 10.1007/s11832-012-0457-4

Lee CF, 2010, SPINE, V35, pE1492, DOI 10.1097/BRS.0b013e3181ecf3fe

Levy AR, 1996, SPINE, V21, P1540, DOI 10.1097/00007632-199607010-00011

Luk KDK, 2010, SPINE, V35, P1607, DOI 10.1097/BRS.0b013e3181c7cb8c

MCALISTER WH, 1975, RADIOL CLIN N AM, V13, P113

Monticone M, 2014, EUR SPINE J, V23, P1204, DOI 10.1007/s00586-014-3241-y

NACHEMSON AL, 1995, J BONE JOINT SURG AM, V77A, P815, DOI 10.2106/00004623-199506000-00001

NASH CL, 1979, J BONE JOINT SURG AM, V61, P371, DOI 10.2106/00004623-197961030-00009

NEGRINI S, 2015, COCHRANE DB SYST REV, V6

Negrini S, 2008, J REHABIL MED, V40, P451, DOI 10.2340/16501977-0195

Negrini S, 2012, SCOLIOSIS SPINAL DIS, V7, DOI 10.1186/1748-7161-7-3

Pehrsson K, 2001, THORAX, V56, P388, DOI 10.1136/thorax.56.5.388

PETERSON LE, 1995, J BONE JOINT SURG AM, V77A, P823, DOI 10.2106/00004623-199506000-00002

Richards BS, 2005, SPINE, V30, P2068, DOI 10.1097/01.brs.0000178819.90239.d0

RISEBOROUGH EJ, 1973, J BONE JOINT SURG AM, VA 55, P974, DOI 10.2106/00004623-197355050-00006

Soucacos P N, 1998, Eur Spine J, V7, P270, DOI 10.1007/s005860050074

Soucacos PN, 2000, ORTHOPEDICS, V23, P833

Soucacos PN, 1997, J BONE JOINT SURG AM, V79A, P1498, DOI 10.2106/00004623-199710000-00006

THULBOURNE T, 1976, J BONE JOINT SURG BR, V58, P64, DOI 10.1302/0301-620X.58B1.1270497

U. S. Preventive Services Task Force (USPSTF), 2015, US PREV SERV TASK FO

*US PREV SERV TASK, 2004, SCREEN ID SCOL AD RE

WEINSTEIN SL, 1983, J BONE JOINT SURG AM, V65, P447, DOI 10.2106/00004623-198365040-00004

WEINSTEIN SL, 1981, J BONE JOINT SURG AM, V63, P702, DOI 10.2106/00004623-198163050-00003

Weinstein SL, 1999, SPINE, V24, P2592, DOI 10.1097/00007632-199912150-00006

Weinstein SL, 2013, SPINE, V38, P1832, DOI 10.1097/01.brs.0000435048.23726.3e

Weinstein SL, 2013, NEW ENGL J MED, V369, P1512, DOI 10.1056/NEJMoa1307337

Wiemann JM, 2014, J PEDIATR ORTHOPED, V34, P603, DOI 10.1097/BPO.0000000000000221

Wong HK, 2005, SPINE, V30, P1188, DOI 10.1097/01.brs.0000162280.95076.bb

Yawn BP, 1999, JAMA-J AM MED ASSOC, V282, P1427, DOI 10.1001/jama.282.15.1427

NR 56

TC 79

Z9 88

U1 1

U2 28

PU AMER MEDICAL ASSOC

PI CHICAGO

PA 330 N WABASH AVE, STE 39300, CHICAGO, IL 60611-5885 USA

SN 0098-7484

EI 1538-3598

J9 JAMA-J AM MED ASSOC

JI JAMA-J. Am. Med. Assoc.

PD JAN 9

PY 2018

VL 319

IS 2

BP 173

EP 187

DI 10.1001/jama.2017.11669

PG 15

WC Medicine, General & Internal

WE Science Citation Index Expanded (SCI-EXPANDED)

SC General & Internal Medicine

GA FS4PR

UT WOS:000419775500017

PM 29318283

OA Bronze

DA 2023-08-10

ER

PT J

AU Yagci, G

Ayhan, C

Yakut, Y

AF Yagci, Gozde

Ayhan, Cigdem

Yakut, Yavuz

TI Effectiveness of basic body awareness therapy in adolescents with

idiopathic scoliosis: A randomized controlled study

SO JOURNAL OF BACK AND MUSCULOSKELETAL REHABILITATION

LA English

DT Article

DE Idiopathic scoliosis; basic body awareness therapy; curve progression;

body symmetry

ID QUALITY-OF-LIFE; EXERCISES REDUCE; CONTROLLED-TRIAL; BOSTON BRACE;

RELIABILITY; DEFORMITIES; MANAGEMENT; PATIENT; PAIN

AB BACKGROUND: In scoliosis, curve progresses due to muscle imbalance and poor posture. Basic body awareness therapy (BBAT) aims to improve posture, coordination, and balance by increasing body awareness, which may help decrease deformities.

OBJECTIVE: This study aimed to investigate effects of Basic body awareness therapy (BBAT) on curve magnitude, trunk asymmetry, cosmetic deformity, and quality of life in adolescent idiopathic scoliosis (AIS) patients.

METHODS: Twenty female AIS patients were randomly assigned to BBAT and traditional exercises (TEs) groups. The BBAT group received BBAT and traditional exercises (TEs), while the TEs group received only TEs. The following assessments were included: Cobb angles using X-ray, angle of trunk rotation (ATR) using scoliometer, trunk asymmetry using the Posterior Trunk Symmetry Index (POTSI), cosmetic deformity using the Walter Reed Visual Assessment Scale (WRVAS), and quality of life using the SRS-22 test. Measurements were conducted at baseline examination and ten weeks later. Patients were instructed to wear their brace 23 h daily. Results were analyzed using the Wilcoxon rank-sum test to compare repeated measurements and Mann-Whitney U test to compare the groups.

RESULTS: The BBAT group had greater improvement in the thoracic Cobb angle than the TEs group. Cosmetic deformity improved in both groups, whereas body asymmetry improved in only the BBAT group. SRS-22 scores were unchanged in both groups.

CONCLUSIONS: BBAT as an additive to bracing and TEs improve curve magnitude, body symmetry and trunk deformity.

C1 [Yagci, Gozde; Ayhan, Cigdem] Hacettepe Univ, Fac Hlth Sci, Sch Phys Therapy & Rehabil Sci, TR-06100 Ankara, Turkey.

[Yakut, Yavuz] Hasan Kalyoncu Univ, Physiotherapy & Rehabil Dept, Gaziantep, Turkey.

C3 Hacettepe University; Hasan Kalyoncu University

RP Yagci, G (通讯作者)， Hacettepe Univ, Fac Hlth Sci, Sch Phys Therapy & Rehabil Sci, TR-06100 Ankara, Turkey.

EM gosdegr@hotmail.com

RI YAGCI, GOZDE/J-1185-2013; Kehoe, Clare/ABC-7984-2020; KURU, CIGDEM
AYHAN/J-1183-2013; YAKUT, YAVUZ/J-1174-2013

OI YAGCI, GOZDE/0000-0002-4603-7162; KURU, CIGDEM
AYHAN/0000-0002-4555-4563;

FU Scientific Research Coordination Unit of Hacettepe University [014 T11
102 002]

FX This work was supported by the Scientific Research Coordination Unit of
Hacettepe University (project no. 014 T11 102 002).

CR Alanay A, 2005, SPINE, V30, P2464, DOI 10.1097/01.brs.0000184366.71761.84

Bisdorff AR, 1996, BRAIN, V119, P1523, DOI 10.1093/brain/119.5.1523

Cakrt O, 2011, J VESTIBUL RES-EQUIL, V21, P161, DOI 10.3233/VES-2011-0414

Chan SL, 2014, SCOLIOSIS SPINAL DIS, V9, DOI 10.1186/1748-7161-9-1

Climent JM, 1999, SPINE, V24, P1903, DOI 10.1097/00007632-199909150-00007

Cobb JR., 1948, INSTR COURSE LECT, V5, P261

Coelho DM, 2013, BRAZ J PHYS THER, V17, P179, DOI 10.1590/S1413-35552012005000081

Cote P, 1998, SPINE, V23, P796, DOI 10.1097/00007632-199804010-00011

Deviren V, 2002, SPINE, V27, P2346, DOI 10.1097/00007632-200211010-00007

Diab AA, 2012, CLIN REHABIL, V26, P1123, DOI 10.1177/0269215512447085

Dixit Swati, 2011, Indian J Community Med, V36, P197, DOI 10.4103/0970-0218.86520

Dropsy J., 1998, QUALITY MOVEMENT ART, P21

Fjellman-Wiklund A, 2004, INT J IND ERGONOM, V33, P357, DOI 10.1016/j.ergon.2003.10.008

Gard G, 2005, DISABIL REHABIL, V27, P725, DOI 10.1080/09638280400009071

최지혜, 2011, Child Health Nursing Research, V17, P167

Gur G, 2015, GAIT POSTURE, V41, P93, DOI 10.1016/j.gaitpost.2014.09.001

Gyllensten A. L., 2001, THESIS

Inami K., 1999, RES SPINAL DEFORMITI, V2, P85

Katz DE, 1997, SPINE, V22, P1302, DOI 10.1097/00007632-199706150-00005

KING HA, 1983, J BONE JOINT SURG AM, V65, P1302, DOI 10.2106/00004623-198365090-00012

Korovessis P, 2000, SPINE, V25, P2064, DOI 10.1097/00007632-200008150-00010

LAURNEN EL, 1983, SPINE, V8, P388, DOI 10.1097/00007632-198305000-00009

Mehling Wolf E, 2011, Philos Ethics Humanit Med, V6, P6, DOI 10.1186/1747-5341-6-6

Monticone M, 2014, EUR SPINE J, V23, P1204, DOI 10.1007/s00586-014-3241-y

Mooney V, 2003, ORTHOPEDICS, V26, P167

Nault ML, 2002, SPINE, V27, P1911, DOI 10.1097/00007632-200209010-00018

Negrini S, 2008, DISABIL REHABIL, V30, P772, DOI 10.1080/09638280801889568

Negrini S, 2006, SCOLIOSIS SPINAL DIS, V1, DOI 10.1186/1748-7161-1-14

Negrini S, 2010, SPINE, V35, P1285, DOI 10.1097/BRS.0b013e3181dc48f4

Olsen AL, 2017, DISABIL REHABIL, V39, P1631, DOI 10.1080/09638288.2016.1209578

Rahman T, 2005, J PEDIATR ORTHOPED, V25, P420, DOI 10.1097/01.bpo.0000161097.61586.bb

Richards BS, 2005, SPINE, V30, P2068, DOI 10.1097/01.brs.0000178819.90239.d0

Romano M, 2013, SPINE, V38, pE883, DOI 10.1097/BRS.0b013e31829459f8

Roxendal G., 1985, DOCTORAL THESIS

Sanders JO, 2003, SPINE, V28, P2158, DOI 10.1097/01.BRS.0000084629.97042.0B

Seifert J, 2009, ORTHOPADE, V38, P151, DOI 10.1007/s00132-008-1367-5

SHNEERSON JM, 1979, ACTA ORTHOP SCAND, V50, P303, DOI 10.3109/17453677908989771

Stokes I, 2001, STUD HLTH TECHNOL IN, V91, P314

STONE B, 1979, PHYS THER, V59, P759, DOI 10.1093/ptj/59.6.759

Takemitsu M, 2004, SPINE, V29, P2070, DOI 10.1097/01.brs.0000138280.43663.7b

Ugwonali Obinwanne F, 2004, Spine J, V4, P254

Weinstein SL, 2003, JAMA-J AM MED ASSOC, V289, P559, DOI 10.1001/jama.289.5.559

Weinstein SL, 2013, NEW ENGL J MED, V369, P1512, DOI 10.1056/NEJMoa1307337

Yang JH, 2013, EUR SPINE J, V22, P2407, DOI 10.1007/s00586-013-2845-y

NR 44

TC 19

Z9 19

U1 3

U2 20

PU IOS PRESS

PI AMSTERDAM

PA NIEUWE HEMWEG 6B, 1013 BG AMSTERDAM, NETHERLANDS

SN 1053-8127

EI 1878-6324

J9 J BACK MUSCULOSKELET

JI J. Back Musculoskelet. Rehabil.

PY 2018

VL 31

IS 4

BP 693

EP 701

DI 10.3233/BMR-170868

PG 9

WC Orthopedics; Rehabilitation

WE Science Citation Index Expanded (SCI-EXPANDED); Social Science Citation Index (SSCI)

SC Orthopedics; Rehabilitation

GA GU1FH

UT WOS:000445002100013

PM 29630516

DA 2023-08-10

ER

PT J

AU Yagci, G

Yakut, Y

Simsek, E

AF Yagci, Gozde

Yakut, Yavuz

Simsek, Engin

TI The effects of exercise on perception of verticality in adolescent

idiopathic scoliosis

SO PHYSIOTHERAPY THEORY AND PRACTICE

LA English

DT Article

DE Subjective visual verticality; postural perception; scoliosis

ID SWISS BALL EXERCISE; RESISTANCE EXERCISE; MOVEMENT QUALITY; POSTURAL

CONTROL; BALANCE CONTROL; ORIENTATION; STABILITY; HUMANS; MECHANISMS;

DISORDERS

AB Purpose: Visual and proprioceptive cues are important for body orientation to maintain correct posture. This study investigated the effects of exercise training on subjective visual, postural, and haptic perception of verticality in patients with scoliosis. Subjects and Methods: Thirty-two female adolescents with moderate idiopathic scoliosis were randomly allocated to Core Stabilization Exercise (CSE), Body Awareness, or Traditional Exercise (TE) groups. Each group completed a 1-hour supervised program, two days per week for 10weeks while continuing to wear spinal braces. Perceptual visual, postural, and haptic estimates were assessed before and after treatment. Results:

Subjective visual vertical perception only improved in the awareness group. Subjective visual horizontal perception, postural vertical perception, total postural perception scores, total haptic perception scores, and haptic perception 45 degrees to the right were significantly improved in the stabilization and awareness groups. For the 60 degrees right and 60 degrees left postural perception parameters, as well as the 45 degrees left haptic perception parameters, perception improved only in the stabilization group. No improvement was observed in the traditional group. Conclusion: Improvements in visual, postural, and haptic verticality perception within the stabilization exercise training and Basic Body Awareness group treatment suggest the addition of these exercise methods for the treatment of idiopathic scoliosis to improve internal body orientation.

C1 [Yagci, Gozde] Hacettepe Univ, Sch Phys Therapy & Rehabil Sci, Fac Hlth Sci, Ankara, Turkey.

[Yakut, Yavuz] Hasan Kalyoncu Univ, Physiotherapy & Rehabil Dept, Fac Hlth Sci, Altindag Turkey, Turkey.

[Simsek, Engin] Dokuz Eylul Univ, Sch Phys Therapy & Rehabil Sci, Izmir, Turkey.

C3 Hacettepe University; Dokuz Eylul University

RP Yagci, G (通讯作者)， Hacettepe Univ, Fac Hlth Sci, Physiotherapy & Rehabil Dept, TR-06100 Ankara, Turkey.

EM gosdegr@hotmail.com

RI YAGCI, GOZDE/J-1185-2013; YAKUT, YAVUZ/J-1174-2013

OI YAGCI, GOZDE/0000-0002-4603-7162;

CR Akuthota V, 2004, ARCH PHYS MED REHAB, V85, pS86, DOI 10.1053/j.apmr.2003.12.005

Akuthota V, 2008, CURR SPORT MED REP, V7, P39, DOI 10.1097/01.CSMR.0000308663.13278.69

de Araujo MEA, 2012, J BODYW MOV THER, V16, P191, DOI 10.1016/j.jbmt.2011.04.002

Barra J, 2010, BRAIN, V133, P3552, DOI 10.1093/brain/awq311

Bergmann J, 2015, ATTEN PERCEPT PSYCHO, V77, P953, DOI 10.3758/s13414-014-0815-z

Bisdorff AR, 1996, BRAIN, V119, P1523, DOI 10.1093/brain/119.5.1523

Bronstein AM, 1999, CURR OPIN NEUROL, V12, P5, DOI 10.1097/00019052-199902000-00002

Cakrt O, 2011, J VESTIBUL RES-EQUIL, V21, P161, DOI 10.3233/VES-2011-0414

Cheung J, 2002, J ORTHOPAED RES, V20, P416, DOI 10.1016/S0736-0266(01)00129-2

Choi J, 2013, ASIAN NURS RES, V7, P120, DOI 10.1016/j.anr.2013.07.001

Cobb J, 1948, INSTRUCTIONAL COURSE

COHEN LA, 1961, J NEUROPHYSIOL, V24, P1

Danielsson L, 2015, INT J QUAL STUD HEAL, V10, DOI 10.3402/qhw.v10.27069

DIETZ V, 1992, EXP BRAIN RES, V89, P229

Fusco C., 2011, Physiotherapy Theory and Practice, V27, P80, DOI 10.3109/09593985.2010.533342

Gandor F, 2016, PARKINSONS DIS-US, V2016, DOI 10.1155/2016/7489105

Gard G, 2005, DISABIL REHABIL, V27, P725, DOI 10.1080/09638280400009071

Guo X, 2006, SPINE, V31, pE437, DOI 10.1097/01.brs.0000222048.47010.bf

Gur G, 2015, GAIT POSTURE, V41, P93, DOI 10.1016/j.gaitpost.2014.09.001

Gyllensten A. L., 2003, ADV PHYSIOTHER, V5, P179, DOI DOI 10.1080/14038109310012061

HERMAN R, 1985, SPINE, V10, P1, DOI 10.1097/00007632-198501000-00001

Imai A, 2010, J ORTHOP SPORT PHYS, V40, P369, DOI 10.2519/jospt.2010.3211

Jeon J, 2012, ANGLE METER VERSION

Kerkhoff G, 1999, NEUROPSYCHOLOGIA, V37, P1387, DOI 10.1016/S0028-3932(99)00031-7

Kim JJ, 2015, J PHYS THER SCI, V27, P1775, DOI 10.1589/jpts.27.1775

LEBLANC R, 1995, ANN CHIR, V49, P762

Luyat M, 1997, ACTA PSYCHOL, V95, P181, DOI 10.1016/S0001-6918(96)00015-7

Malmgren-Olsson Eva-Britt, 2001, Physiotherapy Theory and Practice, V17, P77, DOI 10.1080/095939801750334167

Mast F, 1996, BRAIN RES BULL, V40, P393, DOI 10.1016/0361-9230(96)00132-3

Michaelson P, 2003, J REHABIL MED, V35, P229, DOI 10.1080/16501970306093

Monticone M, 2014, EUR SPINE J, V23, P1204, DOI 10.1007/s00586-014-3241-y

Nault ML, 2002, SPINE, V27, P1911, DOI 10.1097/00007632-200209010-00018

Negrini S, 2012, SCOLIOSIS SPINAL DIS, V7, DOI 10.1186/1748-7161-7-3

Oppenlander K, 2015, NEUROPSYCHOLOGIA, V74, P178, DOI 10.1016/j.neuropsychologia.2015.03.004

Park JY, 2013, J PHYS THER SCI, V25, P1629, DOI 10.1589/jpts.25.1629

Pereira CB, 2014, J NEUROL SCI, V346, P60, DOI 10.1016/j.jns.2014.07.057

Perennou DA, 1998, NEUROSCI LETT, V252, P75, DOI 10.1016/S0304-3940(98)00501-1

Risser J., 1957, CLIN ORTHOP RELAT R, V11, P111

SAHLSTRAND T, 1978, ACTA ORTHOP SCAND, V49, P354, DOI 10.3109/17453677809050088

Saj A, 2005, STROKE, V36, P588, DOI 10.1161/01.STR.0000155740.44599.48

Shin SS, 2012, J PHYS THER SCI, V24, P211

Simoneau M, 2006, EXP BRAIN RES, V170, P576, DOI 10.1007/s00221-005-0246-0

Skjaerven Liv H., 2008, Physiotherapy Theory and Practice, V24, P13, DOI 10.1080/01460860701378042

Skjaerven LH, 2010, PHYS THER, V90, P1479, DOI 10.2522/ptj.20090059

Song GB, 2015, J PHYS THER SCI, V27, P3879, DOI 10.1589/jpts.27.3879

Vaugoyeau M, 2008, GAIT POSTURE, V27, P294, DOI 10.1016/j.gaitpost.2007.04.003

Vibert D, 1999, J VESTIBUL RES-EQUIL, V9, P145

Webber SC, 2004, CLIN BIOMECH, V19, P777, DOI 10.1016/j.clinbiomech.2004.05.014

NR 48

TC 8

Z9 9

U1 0

U2 20

PU TAYLOR & FRANCIS INC

PI PHILADELPHIA

PA 530 WALNUT STREET, STE 850, PHILADELPHIA, PA 19106 USA

SN 0959-3985

EI 1532-5040

J9 PHYSIOTHER THEOR PR

JI Physiother. Theory Pract.

PY 2018

VL 34

IS 8

BP 579

EP 588

DI 10.1080/09593985.2017.1423429

PG 10

WC Rehabilitation

WE Science Citation Index Expanded (SCI-EXPANDED); Social Science Citation Index (SSCI)

SC Rehabilitation

GA GC9EB

UT WOS:000430099200001

PM 29308950

DA 2023-08-10

ER

PT J

AU Langensiepen, S

Stark, C

Sobottke, R

Semler, O

Franklin, J

Schraeder, M

Siewe, J

Eysel, P

Schoenau, E

AF Langensiepen, S.

Stark, C.

Sobottke, R.

Semler, O.

Franklin, J.

Schraeder, M.

Siewe, J.

Eysel, P.

Schoenau, E.

TI Home-based vibration assisted exercise as a new treatment option for

scoliosis - A randomised controlled trial

SO JOURNAL OF MUSCULOSKELETAL & NEURONAL INTERACTIONS

LA English

DT Article

DE Adolescent Idiopathic Scoliosis; Cobb Angle; Whole Body Vibration;

Physical Exercise; Home-training Program

ID WHOLE-BODY VIBRATION; ADOLESCENT IDIOPATHIC SCOLIOSIS; CHILDREN;

REHABILITATION

AB Objectives: The aim of this study was to evaluate the effect of scoliosis specific exercises (SSE) on a side-alternating whole body vibration platform (sWBV) as a home-training program in girls with adolescent idiopathic scoliosis (AIS). Methods: 40 female AIS patients (10-17 years) wearing a brace were randomly assigned to two groups. The intervention was a six months, home-based, SSE program on a sWBV platform five times per week. Exercises included standing, sitting and kneeling. The control group received regular SSE (treatment as usual). The Cobb angle was measured at start and after six months. Onset of menarche was documented for sub-group analysis. Results: The major curve in the sWBV group decreased significantly by -2.3 degrees (SD +/- 3.8) (95% CI -4.1 to -0.5; P=0.014) compared to the difference in the control group of 0.3 degrees (SD +/- 3.7) (95% CI -1.5 to 2.2; P=0.682) (P=0.035). In the sWBV group 20% (n=4) improved, 75% (n=15) stabilized and 5% (n=1) deteriorated by >= 5 degrees. In the control group 0% (n=0) improved, 89% (n=16) stabilized and 11% (n=2) deteriorated. The clinically largest change was observed in the 'before-menarche' sub-group. Conclusions: Home-based SSE combined with sWBV for six months counteracts the

progression of scoliosis in girls with AIS; the results were more obvious before the onset of the menarche.

C1 [Langensiepen, S.] Univ Cologne, Ctr Prevent & Rehabil, Cologne, Germany.

[Stark, C.] Univ Cologne, Childrens & Adolescents Hosp, Kerpener Str 62, D-50939 Cologne, Germany.

[Schoenau, E.] Univ Cologne, CCMB, Cologne, Germany.

[Sobottke, R.] Rhein Maas Klinikum, Staedte Region Aachen, Wuerselen, Germany.

[Franklin, J.] Univ Cologne, Inst Med Stat & Computat Biol, Cologne, Germany.

[Schraeder, M.] Upright MRI Ctr Cologne, Cologne, Germany.

[Siewe, J.; Eysel, P.] Univ Cologne, Dept Orthopaed & Trauma Surg, Cologne, Germany.

[Semler, O.] Univ Cologne, Ctr Rare Skeletal Dis Childhood, Cologne, Germany.

C3 University of Cologne; University of Cologne; University of Cologne;

University of Cologne; University of Cologne; University of Cologne

RP Stark, C (通讯作者)，Univ Cologne, Childrens & Adolescents Hosp, Kerpener Str 62, D-50939 Cologne, Germany.

EM christina.stark@uk-koeln.de

RI Stark, Christina/AAF-1707-2020; Langensiepen, Sina/AAU-5082-2021

OI Stark, Christina/0000-0003-4247-0679; Franklin,

Jeremy/0000-0003-1536-0925; Langensiepen, Sina/0000-0001-6404-764X

CR Abercromby AFJ, 2007, MED SCI SPORT EXER, V39, P1794, DOI 10.1249/mss.0b013e3181238a0f

Anwer S, 2015, BIOMED RES INT-UK, V2015, DOI 10.1155/2015/123848

Cochrane DJ, 2011, J SPORT SCI MED, V10, P19

Fusco C., 2011, Physiotherapy Theory and Practice, V27, P80, DOI 10.3109/09593985.2010.533342

Hoyer-Kuhn H, 2014, J MUSCULOSKEL NEURON, V14, P445

Konieczny MR, 2013, J CHILD ORTHOP, V7, P3, DOI 10.1007/s11832-012-0457-4

Kuru T, 2016, CLIN REHABIL, V30, P181, DOI 10.1177/0269215515575745

Lehnert-Schroth C., 1999, DREIDIMENSIONALE SKO

Lenssinck MLB, 2005, PHYS THER, V85, P1329, DOI 10.1093/ptj/85.12.1329

Matute-Llorente A, 2014, J ADOLESCENT HEALTH, V54, P385, DOI 10.1016/j.jadohealth.2013.11.001

Negrini S, 2008, J REHABIL MED, V40, P451, DOI 10.2340/16501977-0195

Negrini S, 2012, SCOLIOSIS SPINAL DIS, V7, DOI 10.1186/1748-7161-7-3

Negrini S, 2015, COCHRANE DB SYST REV, DOI 10.1002/14651858.CD006850.pub3

Rauch F, 2009, DEV MED CHILD NEUROL, V51, P166, DOI 10.1111/j.1469-8749.2009.03418.x

Rittweger J, 2003, CLIN PHYSIOL FUNCT I, V23, P81, DOI 10.1046/j.1475-097X.2003.00473.x

Rittweger J, 2010, EUR J APPL PHYSIOL, V108, P877, DOI 10.1007/s00421-009-1303-3

Ritzmann R, 2013, SCAND J MED SCI SPOR, V23, P331, DOI 10.1111/j.1600-0838.2011.01388.x

Romano M, 2013, SPINE, V38, pE883, DOI 10.1097/BRS.0b013e31829459f8

Schreiber S, 2016, PLOS ONE, V11, DOI 10.1371/journal.pone.0168746

Stark C, 2015, CHILD NERV SYST, V31, P301, DOI 10.1007/s00381-014-2577-2

Stark C, 2013, MONATSSCHR KINDERH, V161, P625, DOI 10.1007/s00112-013-2910-y

Tanure MC, 2010, SPINE J, V10, P769, DOI 10.1016/j.spinee.2010.02.020

WEINSTEIN SL, 1983, J BONE JOINT SURG AM, V65, P447, DOI 10.2106/00004623-198365040-00004

Weinstein SL, 2013, NEW ENGL J MED, V369, P1512, DOI 10.1056/NEJMoa1307337

NR 24

TC 5

Z9 6

U1 2

U2 15

PU JMNI

PI NAFPLION

PA 7 SPILIADOU SQ, NAFPLION, 21 100, GREECE

SN 1108-7161

J9 J MUSCULOSKEL NEURON

JI J. Musculoskelet. Neuronal Interact.

PD DEC

PY 2017

VL 17

IS 4

BP 259

EP 267

PG 9

WC Neurosciences; Physiology

WE Science Citation Index Expanded (SCI-EXPANDED)

SC Neurosciences & Neurology; Physiology

GA FR2MC

UT WOS:000418900100002

PM 29199184

DA 2023-08-10

ER

PT J

AU Luo, HJ

Lin, SX

Wu, SK

Tsai, MW

Lee, SJ

AF Luo, Hong-Ji

Lin, Shi-Xiang

Wu, Shyi-Kuen

Tsai, Mei-Wun

Lee, Shwn-Jen

TI Comparison of segmental spinal movement control in adolescents with and

without idiopathic scoliosis using modified pressure biofeedback unit

SO PLOS ONE

LA English

DT Article

ID STABILIZING SYSTEM; ABDOMINAL-MUSCLES; CORE STABILITY; PROPRIOCEPTION;

PATHOGENESIS

AB Background

Postural rehabilitation emphasizing on motor control training of segmental spinal movements has been proposed to effectively reduce the scoliotic spinal deformities in adolescent idiopathic scoliosis (AIS). However, information regarding the impairments of segmental spinal movement control involving segmental spinal stabilizers in adolescent idiopathic scoliosis remains limited. Examination of segmental spinal movement control may provide a window for investigating the features of impaired movement control specific to spinal segments that may assist in the development of physiotherapeutic management of AIS.

Objectives

To compare segmental spinal movement control in adolescents with and without idiopathic scoliosis using modified pressure biofeedback unit.

Methods

Segmental spinal movement control was assessed in twenty adolescents with idiopathic scoliosis (AISG) and twenty healthy adolescents (CG) using a modified pressure biofeedback unit. Participants performed segmental spinal movements that primarily involved segmental spinal stabilizing muscles with graded and sustained muscle contraction against/off a pressure cuff from baseline to target pressures and then maintained for 1 min. Pressure data during the 1-minute

maintenance phase were collected for further analysis. Pressure deviation were calculated and compared between groups.

Results

The AISG had significantly greater pressure deviations for all segmental spinal movements of cervical, thoracic, and lumbar spine than the CG.

Conclusion

Pressure biofeedback unit was feasible for assessing segmental spinal movement control in AIS. AISG exhibited poorer ability to grade and sustain muscle activities for local movements of cervical, thoracic, and lumbar spine, suggesting motor control training of segmental spinal movements involving segmental spinal stabilizing muscles on frontal, sagittal, and transverse planes were required.

C1 [Luo, Hong-Ji; Lin, Shi-Xiang; Tsai, Mei-Wun; Lee, Shwn-Jen] Natl Yang Ming Univ, Dept Phys Therapy & Assist Technol, Taipei, Taiwan.

[Luo, Hong-Ji; Lee, Shwn-Jen] Natl Yang Ming Univ, Res Ctr Int Classificat Functioning Disabil & Hlt, Taipei, Taiwan.

[Wu, Shyi-Kuen] Hungkuang Univ, Dept Phys Therapy, Taichung, Taiwan.

C3 National Yang Ming Chiao Tung University; National Yang Ming Chiao Tung

University; Hungkuang University

RP Luo, HJ (通讯作者)， Natl Yang Ming Univ, Dept Phys Therapy & Assist Technol, Taipei, Taiwan.; Luo, HJ (通讯作者)， Natl Yang Ming Univ, Res Ctr Int Classificat Functioning Disabil & Hlt, Taipei, Taiwan.

EM hjluo@ym.edu.tw

CR Akuthota V, 2008, CURR SPORT MED REP, V7, P39, DOI 10.1097/01.CSMR.0000308663.13278.69

[Anonymous], 2005, INT J OSTEOPATH MED, DOI DOI 10.1016/J.IJ0SM.2005.07.003

[Anonymous], 2009, FYSIOTER

Asher MA, 2006, SCOLIOSIS SPINAL DIS, V1, DOI 10.1186/1748-7161-1-2

Burwell RG, 2016, SCOLIOSIS SPINAL DIS, V11, DOI 10.1186/s13013-016-0063-1

Carlsson H, 2013, MANUAL THER, V18, P103, DOI 10.1016/j.math.2012.08.004

Chattanooga Group Inc, 2005, STAB PRESS BIOF OP I

Cheung J, 2005, EUR SPINE J, V14, P130, DOI 10.1007/s00586-004-0780-7

Chwala W, 2014, BIOMED RES INT, V2014, DOI 10.1155/2014/573276

Cobb JR., 1948, INSTR COURSE LECT, V5, P261

COOK SD, 1986, CLIN ORTHOP RELAT R, P118

Domenech J, 2011, EUR SPINE J, V20, P1069, DOI 10.1007/s00586-011-1776-8

GOLDBERG MS, 1994, SPINE, V19, P1562, DOI 10.1097/00007632-199407001-00004

Gur G, 2017, PROSTHET ORTHOT INT, V41, P303, DOI 10.1177/0309364616664151

Guyot MA, 2016, EUR SPINE J, V25, P3130, DOI 10.1007/s00586-016-4551-z

Hagins M, 1999, J ORTHOP SPORT PHYS, V29, P546, DOI 10.2519/jospt.1999.29.9.546

Hefti F, 2013, J CHILD ORTHOP, V7, P17, DOI 10.1007/s11832-012-0460-9

HERMAN R, 1985, SPINE, V10, P1, DOI 10.1097/00007632-198501000-00001

Iqbal ZA, 2013, J PHYS THER SCI, V25, P657, DOI 10.1589/jpts.25.657

KENNELLY KP, 1993, SPINE, V18, P913, DOI 10.1097/00007632-199306000-00017

Konieczny MR, 2013, J CHILD ORTHOP, V7, P3, DOI 10.1007/s11832-012-0457-4

Linek P, 2015, J PHYS THER SCI, V27, P465, DOI 10.1589/jpts.27.465

Lombardi G, 2011, ADV CLIN CHEM, V54, P165, DOI 10.1016/B978-0-12-387025-4.00007-8

PANJABI MM, 1992, J SPINAL DISORD, V5, P383, DOI 10.1097/00002517-199212000-00001

Pialasse JP, 2016, J MANIP PHYSIOL THER, V39, P473, DOI 10.1016/j.jmpt.2016.06.001

Pialasse JP, 2015, GAIT POSTURE, V42, P558, DOI 10.1016/j.gaitpost.2015.08.013

Romano M, 2015, SCOLIOSIS SPINAL DIS, V10, DOI 10.1186/s13013-014-0027-2

SHIRADO O, 1995, ARCH PHYS MED REHAB, V76, P621, DOI 10.1016/S0003-9993(95)80630-X

Simoneau M, 2006, BMC NEUROSCI, V7, DOI 10.1186/1471-2202-7-68

SLAGER UT, 1986, DEV MED CHILD NEUROL, V28, P749

Wang WJ, 2011, J PEDIATR ORTHOPED, V31, pS14, DOI 10.1097/BPO.0b013e3181f73c12

WEINSTEIN SL, 1983, J BONE JOINT SURG AM, V65, P447, DOI 10.2106/00004623-198365040-00004

Weinstein SL, 2008, LANCET, V371, P1527, DOI 10.1016/S0140-6736(08)60658-3

Weiss HR, 2016, ASIAN SPINE J, V10, P570, DOI 10.4184/asj.2016.10.3.570

Willson JD, 2005, J AM ACAD ORTHOP SUR, V13, P316, DOI 10.5435/00124635-200509000-00005

Yang HS, 2014, BIO-MED MATER ENG, V24, P453, DOI 10.3233/BME-130830

Zapata KA, 2015, PEDIATR PHYS THER, V27, P119, DOI 10.1097/PEP.0000000000000131

NR 37

TC 7

Z9 7

U1 1

U2 11

PU PUBLIC LIBRARY SCIENCE

PI SAN FRANCISCO

PA 1160 BATTERY STREET, STE 100, SAN FRANCISCO, CA 94111 USA

SN 1932-6203

J9 PLOS ONE

JI PLoS One

PD JUL 28

PY 2017

VL 12

IS 7

AR e0181915

DI 10.1371/journal.pone.0181915

PG 12

WC Multidisciplinary Sciences

WE Science Citation Index Expanded (SCI-EXPANDED)

SC Science & Technology - Other Topics

GA FC1ED

UT WOS:000406579300033

PM 28753636

OA Green Submitted, gold, Green Published

DA 2023-08-10

ER

PT J

AU Johnston, CE

Tran, DP

McClung, A

AF Johnston, Charles E.

Tran, Dong-Phuong

McClung, Anna

TI Functional and Radiographic Outcomes Following Growth-Sparing Management of Early-Onset Scoliosis

SO JOURNAL OF BONE AND JOINT SURGERY-AMERICAN VOLUME

LA English

DT Article

ID ADOLESCENT IDIOPATHIC SCOLIOSIS; PROSTHETIC TITANIUM RIB; CONGENITAL SCOLIOSIS; SURGICAL-CORRECTION; FUSED RIBS; SPINE; THORACOSTOMY; PULMONARY; CHILDREN

AB Background: In this study, we sought to evaluate radiographic, functional, and quality-of-life outcomes of patients who have completed growth-sparing management of early-onset scoliosis.

Methods: This prospective study involved patients with early-onset scoliosis who underwent growth-sparing treatment and either "final" fusion or observation for >= 2 years since the last

lengthening procedure. Demographics, radiographic parameters, pulmonary function test (PFT) values, and scores of patient-reported assessments (Early-Onset Scoliosis Questionnaire [EOSQ] and Scoliosis Research Society [SRS]-30) were obtained. At the most recent follow-up, patients performed 2 additional functional outcome tests: step-activity monitoring and a treadmill exercise-tolerance test.

Results: Twelve patients were evaluated as "graduates" of growth-sparing management of early-onset scoliosis (mean of 37 months since the most recent surgery). The major scoliosis curve measurement averaged 88(circle) before treatment and 47(circle) at the most recent follow-up. T1-S1 height increased from a mean of 22.3 cm to 34.7 cm and T1-T12 height, from 13.3 to 22.3 cm. At the most recent follow-up, the mean forced expiratory volume in 1 second (FEV1) and forced vital capacity (FVC) as a percentage of the predicted volume were 52.1% and 55.3%, respectively, and were essentially unchanged from the earliest PFT that patients could perform (FEV1 = 53.8% of predicted and FVC = 53.5% of predicted). There was no difference between graduates and controls with respect to activity time or total steps in step-activity monitoring, and in the exercise-tolerance test, graduates walked at the same speed but at a higher heart rate and at a significantly higher ($p < 0.001$) VO2 cost (rate of oxygen consumed per distance traveled). The EOSQ mean score was 102.2 of a possible 120 points, and the SRS mean score was 4.1 of a possible 5 points.

Conclusions: A realistic long-term goal for the management of early-onset scoliosis appears to be spine elongation and maintenance of pulmonary function at a level that is no less than the percentage of normal at initial presentation. Functional testing and patient-reported outcomes at a mean of 3 years from the last surgery suggest that activity levels were generally equal to those of controls but required greater physiologic demand. General health and physical function outcomes revealed continued impairment in these domains.

C1 [Johnston, Charles E.; Tran, Dong-Phuong; McClung, Anna] Texas Scottish Rite Hosp Crippled Children, Dallas, TX 75219 USA.

C3 Texas Scottish Rite Hospital for Children

RP Johnston, CE (通讯作者)，Texas Scottish Rite Hosp Crippled Children, Dallas, TX 75219 USA.

EM charles.johnston@tsrh.org

CR Akbarnia BA, 2005, SPINE, V30, pS46, DOI 10.1097/01.brs.0000175190.08134.73

Cahill PJ, 2010, SPINE, V35, pE1199, DOI 10.1097/BRS.0b013e3181e21b50

Campbell RM, 2004, J BONE JOINT SURG AM, V86A, P1659, DOI 10.2106/00004623-200408000-00009

Campbell RM, 2003, J BONE JOINT SURG AM, V85A, P399, DOI 10.2106/00004623-200303000-00001

Carreon LY, 2011, SPINE, V36, P965, DOI 10.1097/BRS.0b013e3181e92b1d

Dede O, 2014, J BONE JOINT SURG AM, V96A, P1295, DOI 10.2106/JBJS.M.01218

Emans JB, 2005, SPINE, V30, P2824, DOI 10.1097/01.brs.0000190865.47673.6a

Emans JB, 2005, SPINE, V30, pS58, DOI 10.1097/01.brs.0000175194.31986.2f

Flynn JM, 2013, J BONE JOINT SURG AM, V95A, P1745, DOI 10.2106/JBJS.L.01386

Jeans Kelly A, 2016, Spine Deform, V4, P413, DOI 10.1016/j.jspd.2016.06.002

Johnston Charles E, 2014, Spine Deform, V2, P460, DOI 10.1016/j.jspd.2014.04.005

Kamaci S, 2014, J PEDIATR ORTHOPED, V34, P607, DOI 10.1097/BPO.0000000000000169

Karol LA, 2008, J BONE JOINT SURG AM, V90A, P1272, DOI 10.2106/JBJS.G.00184

Long FR, 1999, RADIOLOGY, V212, P588, DOI 10.1148/radiology.212.2.r99au06588

Mariconda M, 2016, EUR SPINE J, V25, P3331, DOI 10.1007/s00586-016-4510-8

Matsumoto H, 2016, J PEDIAT ORTHOP

Stevens WR, 2015, 9 ANN INT C EARL ONS

Thompson GH, 2005, SPINE, V30, P2039, DOI 10.1097/01.brs.0000179082.92712.89

NR 18

TC 23

Z9 25

U1 0

U2 5

PU JOURNAL BONE JOINT SURGERY

PI NEEDHAM

PA 20 PICKERING ST, NEEDHAM, MA 02492 USA

SN 0021-9355

EI 1535-1386

J9 J BONE JOINT SURG AM

JI J. Bone Joint Surg.-Am. Vol.

PD JUN 21

PY 2017

VL 99

IS 12

BP 1036

EP 1042

DI 10.2106/JBJS.16.00796

PG 7

WC Orthopedics; Surgery

WE Science Citation Index Expanded (SCI-EXPANDED)

SC Orthopedics; Surgery

GA EY5OL

UT WOS:000404026500016

PM 28632593

DA 2023-08-10

ER

PT J

AU Gur, G

Ayhan, C

Yakut, Y

AF Gur, Gozde

Ayhan, Cigdem

Yakut, Yavuz

TI The effectiveness of core stabilization exercise in adolescent idiopathic scoliosis: A randomized controlled trial

SO PROSTHETICS AND ORTHOTICS INTERNATIONAL

LA English

DT Article

DE Adolescent idiopathic scoliosis; core stabilization; curve magnitude;

posture; rehabilitation; scoliosis

ID QUALITY-OF-LIFE; RELIABILITY; DEFORMITY; VALIDITY; MUSCLES; BRACE

AB Background: Core stabilization training is used to improve postural balance in musculoskeletal problems.

Objectives: The purpose of this study was to investigate the effectiveness of stabilization training in adolescent idiopathic scoliosis.

Study design: A randomized controlled trial, pretest-posttest design.

Methods: In total, 25 subjects with adolescent idiopathic scoliosis were randomly divided into two groups: stabilization group (n=12) and control group (n=13). The stabilization group received core stabilization in addition to traditional rehabilitation, and the control group received traditional rehabilitation for 10weeks. Assessment included Cobb's angle on radiograph, apical vertebral rotation in Adam's test, trunk asymmetry (Posterior Trunk Symmetry Index), cosmetic trunk deformity (Trunk Appearance Perception Scale), and quality of life (Scoliosis Research Society-22 questionnaire).

Results: Inter-group comparisons showed significantly greater improvements in the mean change in lumbar apical vertebral rotation degree and the pain domain of Scoliosis Research Society-22 in the stabilization group than those in the control group ($p<0.05$). No significant differences were observed for other measurements between the groups; however, trends toward greater improvement were observed in the stabilization group.

Conclusion: Core stabilization training in addition to traditional exercises was more effective than traditional exercises alone in the correction of vertebral rotation and reduction of pain in adolescent idiopathic scoliosis.

Clinical relevance Stabilization exercises are more effective in reducing rotation deformity and pain than traditional exercises in the conservative rehabilitation of adolescent idiopathic scoliosis. These improvements suggest that stabilization training should be added to rehabilitation programs in adolescent idiopathic scoliosis.

C1 [Gur, Gozde; Ayhan, Cigdem; Yakut, Yavuz] Hacettepe Univ, Fac Hlth Sci, Ankara, Turkey.

C3 Hacettepe University

RP Gur, G (通讯作者)， Hacettepe Univ, Physiotherapy & Rehabil Dept, Fac Hlth Sci, TR-06100 Ankara, Turkey.

EM gosdegr@hotmail.com

RI YAGCI, GOZDE/J-1185-2013; YAKUT, YAVUZ/J-1174-2013; KURU, CIGDEM AYHAN/J-1183-2013

OI YAGCI, GOZDE/0000-0002-4603-7162; KURU, CIGDEM AYHAN/0000-0002-4555-4563

FU Scientific Research Coordination Unit of Hacettepe University [014 T11 102 002]

FX The author(s) disclosed receipt of the following financial support for the research, authorship, and/or publication of this article: This work was supported by the Scientific Research Coordination Unit of Hacettepe University (project no. 014 T11 102 002).

CR Akuthota V, 2004, ARCH PHYS MED REHAB, V85, pS86, DOI 10.1053/j.apmr.2003.12.005

de Araujo MEA, 2012, J BODYW MOV THER, V16, P191, DOI 10.1016/j.jbmt.2011.04.002

AMENDT LE, 1990, PHYS THER, V70, P108, DOI 10.1093/ptj/70.2.108

Asher M, 2003, SPINE, V28, P63, DOI 10.1097/00007632-200301010-00015

Aulisa AG, 2015, BMC MUSCULOSKEL DIS, V16, DOI 10.1186/s12891-015-0782-0

Ayhan C, 2014, CLIN REHABIL, V28, P36, DOI 10.1177/0269215513492443

Bago J, 2010, SCOLIOSIS SPINAL DIS, V5, DOI 10.1186/1748-7161-5-6

BUNNELL WP, 1993, SPINE, V18, P1572, DOI 10.1097/00007632-199309000-00001

Chan YL, 1999, PEDIATR RADIOL, V29, P360, DOI 10.1007/s002470050607

de Mauroy JC, 2009, STUD HEALTH TECHNOL, V135, P53

Emery K, 2010, CLIN BIOMECH, V25, P124, DOI 10.1016/j.clinbiomech.2009.10.003

Gotfryd Alberto Ofenhejm, 2014, MedicalExpress (São Paulo, online), V1, P170, DOI 10.5935/MedicalExpress.2014.04.02

Gur G, 2015, GAIT POSTURE, V41, P93, DOI 10.1016/j.gaitpost.2014.09.001

HERMAN R, 1985, SPINE, V10, P1, DOI 10.1097/00007632-198501000-00001

Hides J, 2006, SPINE, V31, pE175, DOI 10.1097/01.brs.0000202740.86338.df

Rojas JI, 2009, COCHRANE DB SYST REV, DOI 10.1002/14651858.CD006643.pub2

Inami K., 1999, RES SPINAL DEFORMITI, V2, P85

Kim H, 2013, EUR SPINE J, V22, P1332, DOI 10.1007/s00586-013-2740-6

KING HA, 1983, J BONE JOINT SURG AM, V65, P1302, DOI 10.2106/00004623-198365090-00012

Koumantakis GA, 2005, CLIN BIOMECH, V20, P474, DOI 10.1016/j.clinbiomech.2004.12.006

Lee M, 2013, J PHYS THER SCI, V25, P445, DOI 10.1589/jpts.25.445

Mannion A F, 1998, Eur Spine J, V7, P289, DOI 10.1007/s005860050077

Monticone M, 2014, EUR SPINE J, V23, P1204, DOI 10.1007/s00586-014-3241-y

Muthukrishnan R, 2010, BMC SPORTS SCI MED R, V2, DOI 10.1186/1758-2555-2-13

NACHEMSON AL, 1995, J BONE JOINT SURG AM, V77A, P815, DOI 10.2106/00004623-199506000-00001

Negrini S, 2006, ST HEAL T, V123, P519

Negrini S, 2006, SCOLIOSIS SPINAL DIS, V1, DOI 10.1186/1748-7161-1-4

Risser J., 1957, CLIN ORTHOP RELAT R, V11, P111

Seifert J, 2009, ORTHOPADE, V38, P151, DOI 10.1007/s00132-008-1367-5

Shaughnessy M, 2004, INT J REHABIL RES, V27, P297, DOI 10.1097/00004356-200412000-00007

Shin SS, 2012, J PHYS THER SCI, V24, P211

Soucacos P N, 1998, Eur Spine J, V7, P270, DOI 10.1007/s005860050074

Standaert CJ, 2008, SPINE J, V8, P114, DOI 10.1016/j.spinee.2007.10.015

Vasiliadis E, 2009, STUD HEALTH TECHNOL, V135, P409

Weiss H R, 1993, Eur Spine J, V1, P240, DOI 10.1007/BF00298367

Weiss Hans-Rudolf, 2003, Pediatr Rehabil, V6, P23, DOI 10.1080/1363849031000095288

Wells C, 2012, COMPLEMENT THER MED, V20, P253, DOI 10.1016/j.ctim.2012.02.005

Wever DJ, 1999, EUR SPINE J, V8, P252, DOI 10.1007/s005860050169

Zaina Fabio, 2011, Physiotherapy Theory and Practice, V27, P54, DOI 10.3109/09593985.2010.503988

Zoabli G, 2007, SPINE J, V7, P338, DOI 10.1016/j.spinee.2006.04.001

NR 40

TC 26

Z9 27

U1 4

U2 42

PU SAGE PUBLICATIONS LTD

PI LONDON

PA 1 OLIVERS YARD, 55 CITY ROAD, LONDON EC1Y 1SP, united kingdom

SN 0309-3646

EI 1746-1553

J9 PROSTHET ORTHOT INT

JI Prosthet. Orthot. Int.

PD JUN

PY 2017

VL 41

IS 3

BP 303

EP 310

DI 10.1177/0309364616664151

PG 8

WC Orthopedics; Rehabilitation

WE Science Citation Index Expanded (SCI-EXPANDED)

SC Orthopedics; Rehabilitation

GA EU1BE

UT WOS:000400746500011

PM 27625122

DA 2023-08-10

ER

PT J

AU Atici, Y

Aydin, CG

Atici, A

Buyukkuscu, MO

Arikan, Y

Balioglu, MB

AF Atici, Yunus

Aydin, Canan Gonen

Atici, Aysegul

Buyukkuscu, Mehmet Ozbey

Arikan, Yavuz

Balioglu, Mehmet Bulent

TI The effect of Kinesio taping on back pain in patients with Lenke Type 1

adolescent idiopathic scoliosis: A randomized controlled trial

SO ACTA ORTHOPAEDICA ET TRAUMATOLOGICA TURCICA

LA English

DT Article

DE Adolescent idiopathic scoliosis; Back pain; Exercise; Kinesio taping

ID CHILDREN; PREVALENCE; DISABILITY; MANAGEMENT; EXERCISE; ADULT

AB Purpose: This study investigated the short-term effects of KT on back pain (BP) in patients with Lenke Type 1 adolescent idiopathic scoliosis (AIS).

Methods: We chosen Lenke Type 1 scoliosis who have had only back pain (the localization of the pain: the only in the apical convex edge). Forty patients suffering from BP with Lenke Type 1 AIS were randomly separated into two groups, Group 1 (20 patients) and Group 2 (20 patients). Group 1 was given KT with tension and home exercises and Group 2 was given KT without tension and home exercises. KT and home exercises was applied to the thoracic area of the patients in both groups for four weeks. Pain intensity was measured using a visual analog scale (VAS) and SRS-22 (subtotal SRS-20) before and after treatment.

Results: Mean age of both groups was 16.1 years. Mean Cobb angle of the thoracic scoliosis was 31.8 degrees (range: 17 degrees-44 degrees) in Group 1 and 32.8 degrees (range: 19 degrees-43 degrees) in Group 2 before the treatment. The decrease in VAS score of Group 1 after taping was higher than that of Group 2. The difference between the pre- and post-treatment VAS scores of both groups was statistically significant ($p < 0.05$). The increase in mean SRS-20 score of Group 1 following taping application was significantly higher than the increase in the control group ($p < 0.05$).

Conclusion: Results demonstrated that KT application with tension effectively leads to back pain relief shortly after application. In addition, KT has a positive impact on quality of life. Thus, KT may be a suitable intervention in treating back pain of patients with AIS. Level of Evidence: Level 1, Therapeutic study (C) 2017 Turkish Association of Orthopaedics and Traumatology. Publishing services by Elsevier B.V.

C1 [Atici, Yunus] Okan Univ, Med Fac, Dept Orthopaed & Traumatol, Istanbul, Turkey.

[Aydin, Canan Gonen; Buyukkuscu, Mehmet Ozbey; Arikan, Yavuz; Balioglu, Mehmet Bulent] Metin Sabanci Baltalimani Bone Dis Training & Res, Istanbul, Turkey.

[Atici, Aysegul] Kartal Training & Res Hosp, Istanbul, Turkey.

[Atici, Aysegul] Carsamba State Hosp, Samsun, Turkey.

C3 Okan University; Baltalimani Bone Diseases Training & Research Hospital;

Istanbul Kartal Dr Lutfi Kirdar Training & Research Hospital; Carsamba

State Hospital

RP Atici, Y (通讯作者)， Okan Univ Hosp, Aydinli Yolu Cad,Aydemir Sok 2, TR-34947 Istanbul, Turkey.

EM yunatici@hotmail.com

RI Balioglu, Mehmet Bulent/D-3422-2015; Balioglu, Mehmet/HZJ-7874-2023

OI Balioglu, Mehmet Bulent/0000-0001-5127-2004;

CR Ahlqwist A, 2008, SPINE, V33, pE721, DOI 10.1097/BRS.0b013e318182c347

Ahlqwist A, 2012, INT J QUAL STUD HEAL, V7, DOI 10.3402/qhw.v7i0.15471

Alanay A, 2005, SPINE, V30, P2464, DOI 10.1097/01.brs.0000184366.71761.84

Bae SH, 2013, J PHYS THER SCI, V25, P1367, DOI 10.1589/jpts.25.1367

Balague F, 2012, LANCET, V379, P482, DOI 10.1016/S0140-6736(11)60610-7

Boonstra AM, 2014, PAIN, V155, P2545, DOI 10.1016/j.pain.2014.09.014

Calvo-Munoz I, 2013, BMC MUSCULOSKEL DIS, V14, DOI 10.1186/1471-2474-14-55

Lins CAD, 2013, MANUAL THER, V18, P41, DOI 10.1016/j.math.2012.06.009

DOWNIE WW, 1978, ANN RHEUM DIS, V37, P378, DOI 10.1136/ard.37.4.378

Fanucchi GL, 2009, AUST J PHYSIOTHER, V55, P97, DOI 10.1016/S0004-9514(09)70039-X

Fratocchi G, 2013, J SCI MED SPORT, V16, P245, DOI 10.1016/j.jsams.2012.06.003

Freidel K, 1981, SPINE, V15, P87

Glassman SD, 2005, SPINE, V30, P2024, DOI 10.1097/01.brs.0000179086.30449.96

Hestbaek Lise, 2010, Chiropr Osteopat, V18, P15, DOI 10.1186/1746-1340-18-15

Joncas J, 1996, ANN CHIR, V50, P637

Jones M, 2007, PEDIATR EXERC SCI, V19, P349, DOI 10.1123/pes.19.3.349

Kachanathu SJ, 2014, J PHYS THER SCI, V26, P1185, DOI 10.1589/jpts.26.1185

Kase K, 2003, ILLUSTRATED KINESIO, P6

Kase K., 2003, CLIN THERAPEUTIC APP, V3rd ed

Kase K, 2013, CLIN THERAPEUTIC APP, P12

Kim HS, 2014, ASIAN SPINE J, V8, P695, DOI 10.4184/asj.2014.8.5.695

Langendoen J, 2011, WHAT YOU NEED KNOW T, P9

Lenke LG, 2001, J BONE JOINT SURG AM, V83A, P1169, DOI 10.2106/00004623-200108000-00006

Luz MA, 2015, BRAZ J PHYS THER, V19, P482, DOI 10.1590/bjpt-rbf.2014.0128

Castro-Sanchez AM, 2012, J PHYSIOTHER, V58, P89, DOI 10.1016/S1836-9553(12)70088-7

MAYO NE, 1994, SPINE, V19, P1573, DOI 10.1097/00007632-199407001-00005

Negrini S, 2008, DISABIL REHABIL, V30, P731, DOI 10.1080/09638280801889485

Paoloni M, 2011, EUR J PHYS REHAB MED, V47, P237

Pillastrini P, 2012, JOINT BONE SPINE, V79, P176, DOI 10.1016/j.jbspin.2011.03.019

Pratt RK, 2002, SPINE, V27, P1543, DOI 10.1097/00007632-200207150-00012

Ramirez N, 1997, J BONE JOINT SURG AM, V79A, P364, DOI 10.2106/00004623-199703000-00007

Sato T, 2011, EUR SPINE J, V20, P274, DOI 10.1007/s00586-010-1657-6

Parreira PDS, 2014, J PHYSIOTHER, V60, P90, DOI 10.1016/j.jphys.2014.05.003

Theroux J, 2015, PAIN RES MANAG, V20, P153, DOI 10.1155/2015/674354

Van Middelkoop M, 2014, BEST PRACT RES CLIN, V24, P193

Vanti C, 2015, PHYS THER, V95, P493, DOI 10.2522/ptj.20130619

WEINSTEIN SL, 1981, J BONE JOINT SURG AM, V63, P702, DOI 10.2106/00004623-198163050-00003

Yang JM, 2015, J PHYS THER SCI, V27, P2667, DOI 10.1589/jpts.27.2667

Zakaria A, 2012, J PHYS THER SCI, V24, P1127, DOI 10.1589/jpts.24.1127

NR 39

TC 5

Z9 6

U1 1

U2 18

PU TURKISH ASSOC ORTHOPAEDICS TRAUMATOLOGY

PI ISTANBUL

PA SEHREMINI MAH KOYUNCU SK CIGDEM APT NO 4 D 5 FATIH, ISTANBUL, 00000,

TURKEY

SN 1017-995X

J9 ACTA ORTHOP TRAUMATO

JI Acta Orthop. Traumatol. Turc.

PD MAY

PY 2017

VL 51

IS 3

BP 191

EP 196

DI 10.1016/j.aott.2017.01.002

PG 6

WC Orthopedics

WE Science Citation Index Expanded (SCI-EXPANDED)

SC Orthopedics

GA FC4CX

UT WOS:000406787300002

PM 28330700

OA Green Published, gold

DA 2023-08-10

ER

PT J

AU Diarbakerli, E

Grauers, A

Danielsson, A

Gerdhem, P

AF Diarbakerli, Elias

Grauers, Anna

Danielsson, Aina

Gerdhem, Paul

TI Adults With Idiopathic Scoliosis Diagnosed at Youth Experience Similar

Physical Activity and Fracture Rate as Controls

SO SPINE

LA English

DT Article

DE Brace; fractures; idiopathic; juvenile; osteopenia; osteoporosis;

physical activity; scoliosis; surgery

ID LEAST 20 YEARS; ACTIVITY QUESTIONNAIRE; FOLLOW-UP; OSTEOPOROSIS

PREVENTION; YOUNG-ADULTS; BONE MASS; EXERCISE; VALIDITY; SURGERY; BRACE

AB Study Design. Cross-sectional.

Objective. To describe physical activity level and fracture rates in adults with idiopathic scoliosis, diagnosed before maturity, and to compare with a control group.

Summary of Background Data. A previous study found a lower level of sporting activities in adults treated for idiopathic scoliosis compared with controls. Other studies have shown a lower bone mass in adults with idiopathic scoliosis compared with controls.

Methods. One thousand two hundred seventy-eight adults (aged 18-71 yr) with idiopathic scoliosis and 214 controls (aged 18-70 yr) were included and answered the International Physical Activity Questionnaire - Short Form (IPAQ-SF) and questions about previous fractures. The three scoliosis treatment groups (untreated n=360, brace n=460, and surgically treated n=458) were compared. Furthermore, a comparison based on onset (juvenile n=169 or adolescent n=976) was performed. Achieved weekly moderate activity level and metabolic equivalent task (MET) minutes/week were assessed for patients and controls. Statistical comparisons were made with analysis of covariance with adjustments for age, body mass index, and sex.

Results. The proportion achieving weekly moderate activity level was 962 out of 1278 for individuals with idiopathic scoliosis (75%) and 157 out of 214 (73%) for controls (P=0.40). The scoliosis patients reported 2016MET-minutes/week (median value) and the controls 2456 (P=0.06). Fracture rates did not differ (P=0.72). Fewer surgically treated individuals achieved moderate activity level (P=0.046) compared with the untreated and the previously braced individuals. No difference was seen regarding MET-minutes/week (P=0.86). No differences were seen between individuals with a juvenile onset compared with individuals with an adolescent onset (all P >= 0.05).

Conclusion. Adults with idiopathic scoliosis have similar physical activity level and do not sustain more fractures compared with controls. Adults with surgically treated idiopathic scoliosis have

slightly lower physical activity level than previously braced and untreated patients. Onset of idiopathic scoliosis does not affect physical activity level.

C1 [Diarbakerli, Elias; Grauers, Anna; Gerdhem, Paul] Karolinska Inst, Dept Clin Sci Intervent & Technol CLINTEC, SE-14186 Stockholm, Sweden.

[Diarbakerli, Elias; Gerdhem, Paul] Karolinska Univ Hosp, Dept Orthopaed, K54, SE-14186 Stockholm, Sweden.

[Grauers, Anna] Sundsvall & Harnosand Cty Hosp, Dept Orthopaed, Sundsvall, Sweden.

[Danielsson, Aina] Sahlgrens Univ Hosp, Dept Orthopaed, Gothenburg, Sweden.

[Danielsson, Aina] Univ Gothenburg, Sahlgrenska Acad, Inst Clin Sci, Dept Orthopaed, Gothenburg, Sweden.

C3 Karolinska Institutet; Karolinska Institutet; Karolinska University

Hospital; Sahlgrenska University Hospital; University of Gothenburg

RP Diarbakerli, E (通讯作者)， Karolinska Inst, Dept Clin Sci Intervent & Technol CLINTEC, SE-14186 Stockholm, Sweden.; Diarbakerli, E (通讯作者)， Karolinska Univ Hosp, Dept Orthopaed, K54, SE-14186 Stockholm, Sweden.

EM elias.diarbakerli@karolinska.se

RI Diarbakerli, Elias/O-1074-2019

OI Gerdhem, Paul/0000-0001-8061-7163

CR Ainsworth BE, 2000, MED SCI SPORT EXER, V32, pS498, DOI 10.1097/00005768-200009001-00009

Akseer N, 2015, SCOLIOSIS SPINAL DIS, V10, DOI 10.1186/s13013-015-0031-1

Alghadir AH, 2015, J PHYS THER SCI, V27, P2261, DOI 10.1589/jpts.27.2261

Behensky H, 1998, J SPINAL DISORD, V11, P155

Bielemann RM, 2013, BMC MUSCULOSKEL DIS, V14, DOI 10.1186/1471-2474-14-77

Bradney M, 1998, J BONE MINER RES, V13, P1814, DOI 10.1359/jbmr.1998.13.12.1814

Brown W, 2004, AUST NZ J PUBL HEAL, V28, P128, DOI 10.1111/j.1467-842X.2004.tb00925.x

Cheng JCY, 1999, SPINE, V24, P1218, DOI 10.1097/00007632-199906150-00008

Cobb JR., 1948, INSTR COURSE LECT, V5, P261

Craig CL, 2003, MED SCI SPORT EXER, V35, P1381, DOI 10.1249/01.MSS.0000078924.61453.FB

Danielsson AJ, 2001, EUR SPINE J, V10, P278, DOI 10.1007/s005860100309

Danielsson AJ, 2006, SPINE, V31, P275, DOI 10.1097/01.brs.0000197652.52890.71

Engsberg JR, 2003, SPINE, V28, P1836, DOI 10.1097/00007632-200308150-00012

Fogelholm M, 2006, MED SCI SPORT EXER, V38, P753, DOI 10.1249/01.mss.0000194075.16960.20

Gerdhem P, 2007, J BONE JOINT SURG BR, V89B, P1627, DOI 10.1302/0301-620X.89B12.18946

Grauers A, 2014, SPINE, V39, P886, DOI 10.1097/BRS.0000000000000312

Grauers A, 2013, EUR SPINE J, V22, P2421, DOI 10.1007/s00586-013-2860-z

Grauers A, 2012, EUR SPINE J, V21, P1069, DOI 10.1007/s00586-011-2074-1

IPAQ Research Committee, GUID DAT PROC AN INT

Kemmler W, 2015, OSTEOPOROSIS INT, V26, P2491, DOI 10.1007/s00198-015-3165-3

Landry BW, 2012, PM&R, V4, P826, DOI 10.1016/j.pmrj.2012.09.585

Lee MC, 2013, SPINE, V38, pE1405, DOI 10.1097/BRS.0b013e3182a4038b

Lee PH, 2011, INT J BEHAV NUTR PHY, V8, DOI 10.1186/1479-5868-8-115

Li XF, 2008, EUR SPINE J, V17, P1431, DOI 10.1007/s00586-008-0757-z

Linden C, 2006, J BONE MINER RES, V21, P829, DOI 10.1359/JBMR.060304

Mahaudens P, 2013, EUR SPINE J, V22, P2399, DOI 10.1007/s00586-013-2837-y

Mahaudens P, 2010, EUR SPINE J, V19, P1179, DOI 10.1007/s00586-010-1292-2

Parsch D, 2002, CLIN J SPORT MED, V12, P95, DOI 10.1097/00042752-200203000-00005

WILLNER S, 1982, ACTA ORTHOP SCAND, V53, P233, DOI 10.3109/17453678208992208

Zernicke R, 2006, APPL PHYSIOL NUTR ME, V31, P655, DOI 10.1139/h06-051

NR 30

TC 10

Z9 11

U1 1

U2 9

PU LIPPINCOTT WILLIAMS & WILKINS

PI PHILADELPHIA

PA TWO COMMERCE SQ, 2001 MARKET ST, PHILADELPHIA, PA 19103 USA

SN 0362-2436

EI 1528-1159

J9 SPINE

JI SPINE

PD APR 1

PY 2017

VL 42

IS 7

BP E404

EP E410

DI 10.1097/BRS.0000000000001841

PG 7

WC Clinical Neurology; Orthopedics

WE Science Citation Index Expanded (SCI-EXPANDED)

SC Neurosciences & Neurology; Orthopedics

GA EQ6ZF

UT WOS:000398231600018

PM 27496666

DA 2023-08-10

ER

PT J

AU Chan, CYW

Aziz, I

Chai, FW

Kwan, MK

AF Chan, Chris Yin Wei

Aziz, Izzuddin

Chai, Fong Wei

Kwan, Mun Keong

TI A Silver Medal Winner at the 13th World Wu Shu Championship 2015 17

Months After Selective Thoracic Fusion for Adolescent Idiopathic

Scoliosis A Case Report

SO SPINE

LA English

DT Article

DE Adolescent Idiopathic Scoliosis; instrumentation; martial art; selective

thoracic fusion; silver medal; spinal flexibility; sports; Taolu

exponent; World Wu Shu Championship 2015; Wu Shu

ID POSTERIOR SPINAL-FUSION; ATHLETIC ACTIVITY; TRUNK RANGE; SURGERY;

RETURN; MOTION

AB Study Design. Case report.

Objective. To report the successful rehabilitation and the training progress of an elite high performance martial art exponent after selective thoracic fusion for Adolescent Idiopathic Scoliosis (AIS).

Summary of Background Data. Posterior spinal fusion for AIS will result in loss of spinal flexibility. The process of rehabilitation after posterior spinal fusion for AIS remains controversial and there are few reports of return to elite sports performance after posterior spinal fusion for AIS.

Methods. We report a case of a 25-year-old lady who was a national Wu Shu exponent. She was a Taolu (Exhibition) exponent. She underwent Selective Thoracic Fusion (T4 to T12) using alternate level pedicle screw placement augmented with autogenous local bone graft in June 2014. She commenced her training at 3-month postsurgery and the intensity of her training was increased after 6 months postsurgery. We followed her up to 2 years postsurgery and showed no instrumentation failure or lost of correction.

Results. After selective thoracic fusion, her training process consisted of mainly speed training, core strengthening, limb strengthening, and flexibility exercises. At 17 months of postoperation, she participated in 13th World Wu Shu Championship 2015 and won the silver medal.

Conclusion. Return to elite high-performance martial arts sports was possible after selective thoracic fusion for AIS. The accelerated and intensive training regime did not lead to any instrumentation failure and complications.

C1 [Chan, Chris Yin Wei; Aziz, Izzuddin; Chai, Fong Wei; Kwan, Mun Keong] Univ Malaya, Fac Med, Dept Orthopaed Surg NOCERAL, Kuala Lumpur 50603, Malaysia.

C3 Universiti Malaya

RP Kwan, MK (通讯作者)， Univ Malaya, Fac Med, Dept Orthopaed Surg NOCERAL, Kuala Lumpur 50603, Malaysia.

EM munkeong42@hotmail.com

RI Chan, Chris Yin Wei/H-1377-2014; Kwan, Mun Keong/C-6815-2011

OI Chan, Chris Yin Wei/0000-0001-7245-0295; Kwan, Mun

Keong/0000-0002-9512-3155

CR [Anonymous], 2009, STACYS STORY

[Anonymous], 2015, TABLE RESULTS 13 WOR

Artioli GG, 2009, J STRENGTH COND RES, V23, P20, DOI 10.1519/JSC.0b013e318187687a

Engsberg JB, 2002, SPINE, V27, P1346, DOI 10.1097/00007632-200206150-00018

Engsberg JR, 2003, SPINE, V28, P1993, DOI 10.1097/01.BRS.0000087209.34602.42

Fabricant PD, 2012, J PEDIATR ORTHOPED, V32, P259, DOI 10.1097/BPO.0b013e31824b285f

Lehman RA, 2015, SPINE J, V15, P951, DOI 10.1016/j.spinee.2013.06.035

Rubery PT, 2002, SPINE, V27, P423, DOI 10.1097/00007632-200202150-00019

NR 8

TC 5

Z9 6

U1 1

U2 14

PU LIPPINCOTT WILLIAMS & WILKINS

PI PHILADELPHIA

PA TWO COMMERCE SQ, 2001 MARKET ST, PHILADELPHIA, PA 19103 USA

SN 0362-2436

EI 1528-1159

J9 SPINE

JI SPINE

PD FEB 15

PY 2017

VL 42

IS 4

BP E248

EP E252

DI 10.1097/BRS.0000000000001748

PG 5

WC Clinical Neurology; Orthopedics

WE Science Citation Index Expanded (SCI-EXPANDED)

SC Neurosciences & Neurology; Orthopedics

GA EM9OS

UT WOS:000395641600008

PM 28207671

DA 2023-08-10

ER

PT J

AU Chongov, B

Alexiev, V

Georgiev, H

Kalinov, K

Dimitrova, E

AF Chongov, Borislav

Alexiev, Venelin

Georgiev, Hristo

Kalinov, Krasimir

Dimitrova, Evgenia

TI CORRELATION BETWEEN SCOLIOSIS DEFORMITY TYPE AND TRUNK SYMMETRY BEFORE

AND AFTER SCHROTH PHYSIOTHERAPEUTIC EXERCISES

SO COMPTES RENDUS DE L ACADEMIE BULGARE DES SCIENCES

LA English

DT Article

DE scoliosis; POTSI; ATSI; Rigo classification; BSPTS; Schroth method

AB The variety of the scoliosis deformities is large. They can differ in type and severity. Our interest is the effectiveness of teaching in corrective exercises. Our aim is to follow the dynamics of the posterior (POTSI) and anterior trunk symmetry index (ATSI) in relation to the type of scoliosis deformity according to Rigo classification.

For a period of 2 years and 8 months (April 2014 - November 2016) we accessed 128 children with adolescent idiopathic scoliosis with different severity - low 7.8%, low to moderate 20.3%, moderate 39.1%, moderate to severe 17.2%, severe 11.7% and very severe 3.9%. They were distributed in 4 groups according to Manuel Rigo: 3C (single thoracic or thoracolumbar) 29.6%, 4C (double major) 25%, N3N4 (scoliosis with good balance) 31.3% and G 1-2 (single lumbar) 14.1%. We made photos from anterior and posterior for calculating POTSI and ATSI in the beginning of therapy and on the 5th day after exercises. All patients we taught to make exercises for correction according to Schroth

method after the initial evaluation. Teaching the patients included 5 consecutive days of 120 min in small groups of 2 children. The highest initial POTSI asymmetry was found in group 3C 42.14 +/- 20, with lowest POTSI asymmetry in group N3N4 with 23.22 +/- 11.38. With ATSI the highest initial asymmetry was in 3C 29.09 +/- 12.73 and 4C 30.11 +/- 13.49, with lowest asymmetry in G 1-2 20.41 +/- 12.86. We have statistically significant improvement in all patients as for POTSI from initial 31.27 +/- 17.1 to the end value of 23.08 +/- 14.38 (0.634, $p < 0.001$), as also for ATSI from initial 27.09 +/- 12.37 to end value of 22.9 +/- 10.69 (0.501, $p < 0.001$). Statistically remarkable is the improvement of POTSI in group 3C from initial 42.14 +/- 19.98 to end value of 27.36 +/- 17.63 (0.607, $p < 0.001$), which could be explained with the higher initial asymmetry. In the other groups improvement of POTSI is with similar values: 4C from initial 29.72 +/- 14.17 to end 26.23 +/- 15.04 (0.704, $p < 0.001$); N3N4 from initial 23.22 +/- 11.38 to end 17.09 +/- 8.85 (0.480,$p < 0.01$); G 1-2 from initial 28.53 +/- 14.49 to end 21.54 +/- 10.71 (0.590, $p < 0.1$).

Improvement in the trunk symmetry does not depend on the severity and type of scoliosis, but only on the initial values. With PSSE we have statistically significant improvement in the symmetry indices (POTSI and ATSI) only for 5 days intensive training. Single thoracic and single thoracolumbar scoliosis leads to the most pronounced asymmetry in comparison to the scoliosis with good trunk balance.

C1 [Chongov, Borislav; Dimitrova, Evgenia] Natl Sports Acad V Levski, Dept Physiotherapy, Sofia, Bulgaria.

[Chongov, Borislav; Alexiev, Venelin; Georgiev, Hristo] Sofia Med Univ, Orthopaed Hosp Prof B Boichev & MC Orthomed, 56,N Petkov Blvd, Sofia 1614, Bulgaria.

[Kalinov, Krasimir] New Bulgarian Univ, Dept Informat, 21,Montevideo St, Sofia 1618, Bulgaria.

C3 National Sports Academy - Bulgaria; Medical University Sofia; New

Bulgarian University

RP Chongov, B (通讯作者)， Natl Sports Acad V Levski, Dept Physiotherapy, Sofia, Bulgaria.;

Chongov, B (通讯作者)， Sofia Med Univ, Orthopaed Hosp Prof B Boichev & MC Orthomed, 56,N Petkov Blvd, Sofia 1614, Bulgaria.

EM bobychongov@gmail.com

CR [Anonymous], SCOLIOSIS S2

Bettany-Saltikov J, 2012, PHYSICAL THERAPY PERSPECTIVES IN THE 21ST CENTURY - CHALLENGES AND POSSIBILITIES, P3

Lehnert-Schroth C., 2007, 3 DIMENSIONAL TREATM, V7th ed.

LEHNERTSCHROTH CH, 2000, DREIDIMENSIONALE SKO

Lenke LG, 2001, J BONE JOINT SURG AM, V83A, P1169, DOI 10.2106/00004623-200108000-00006

Negrini S., 2011, SCOLIOSIS, V7.1

Patias P, 2010, SCOLIOSIS SPINAL DIS, V5, DOI 10.1186/1748-7161-5-12

RIGO M, 2007, SCOLIOSIS 1, V2, P7

Rigo M, 2009, STUD HEALTH TECHNOL, V135, P303

Rigo M, 2009, STUD HEALTH TECHNOL, V135, P208

Schreiber S, 2016, PLOS ONE, V11, DOI 10.1371/journal.pone.0168746

Schreiber S, 2015, SCOLIOSIS SPINAL DIS, V10, DOI 10.1186/s13013-015-0048-5

STOLINSKI L, 2012, SCOLIOSIS S1, V7, pO65

Stolinski L, 2012, STUD HEALTH TECHNOL, V176, P242, DOI 10.3233/978-1-61499-067-3-242

Suzuki N, 1999, ST HEAL T, V59, P81

WEISS HR, 1995, Z ORTHOP GRENZGEB, V133, P114, DOI 10.1055/s-2008-1039421

WEISS HR, 2011, SCOLIOSIS SPINAL DIS, V6

Zapata K, 2016, SCOLIOSIS SPINAL DIS, V11, DOI 10.1186/s13013-016-0101-z

NR 18

TC 3

Z9 3

U1 1

U2 13

PU PUBL HOUSE BULGARIAN ACAD SCI

PI SOFIA

PA ACADEMICIAN G BONCEV ST, 1113 SOFIA, BULGARIA

SN 1310-1331

J9 CR ACAD BULG SCI

JI C. R. Acad. Bulg. Sci.

PY 2017

VL 70

IS 10

BP 1467

EP 1474

PG 8

WC Multidisciplinary Sciences

WE Science Citation Index Expanded (SCI-EXPANDED)

SC Science & Technology - Other Topics

GA FN8SV

UT WOS:000416296700016

DA 2023-08-10

ER

PT J

AU Zapata, KA

Wang-Price, SS

Sucato, DJ

AF Zapata, Karina A.

Wang-Price, Sharon S.

Sucato, Daniel J.

TI Six-Month Follow-up of Supervised Spinal Stabilization Exercises for Low Back Pain in Adolescent Idiopathic Scoliosis

SO PEDIATRIC PHYSICAL THERAPY

LA English

DT Article

DE core exercises; physical therapy; spine deformity

ID DISABILITY QUESTIONNAIRE; FUNCTIONAL SCALE; CHILDREN; VALIDATION

AB Purpose: To evaluate the effectiveness of 8 weeks of weekly spinal stabilization exercises compared with 1-time treatment in participants with adolescent idiopathic scoliosis and low back pain at a 6-month follow-up. Methods: Thirty-two participants were evaluated at a 6-month follow-up. The supervised group received weekly spinal stabilization exercises. The unsupervised group received a 1-time treatment and home exercise program. Results: Both groups improved in all outcome measures. The supervised group had significantly reduced Numeric Pain Rating Scale and Improved Global Rating of Change scores, but had no differences in the revised Oswestry Back Pain Disability Questionnaire, or Patient-Specific Functional Scale scores in comparison with the unsupervised group.

C1 [Zapata, Karina A.; Sucato, Daniel J.] Texas Scottish Rite Hosp Children, 2222 Welborn St, Dallas, TX 75219 USA.

[Wang-Price, Sharon S.] Texas Womans Univ, Sch Phys Therapy, Dallas, TX USA.

C3 Texas Scottish Rite Hospital for Children; Texas Womans University

RP Zapata, KA (通讯作者)， Texas Scottish Rite Hosp Children, 2222 Welborn St, Dallas, TX 75219 USA.

EM Karina.zapata@tsrh.org

FU APTA Section on Pediatrics

FX Grant funding was received from the APTA Section on Pediatrics for this

work.

CR Ahlqwist A, 2008, SPINE, V33, pE721, DOI 10.1097/BRS.0b013e318182c347

[Anonymous], 1989, BACK PAIN NEW APPROA

Bailey B, 2010, PAIN, V149, P216, DOI 10.1016/j.pain.2009.12.008

Cairns MC, 2006, SPINE, V31, pE670, DOI 10.1097/01.brs.0000232787.71938.5d

Calvo-Munoz I, 2013, BMC MUSCULOSKEL DIS, V14, DOI 10.1186/1471-2474-14-55

Fanucchi GL, 2009, AUST J PHYSIOTHER, V55, P97, DOI 10.1016/S0004-9514(09)70039-X

Faul F, 2007, BEHAV RES METHODS, V39, P175, DOI 10.3758/BF03193146

Fritz JM, 2001, PHYS THER, V81, P776, DOI 10.1093/ptj/81.2.776

Hall AM, 2011, EUR SPINE J, V20, P79, DOI 10.1007/s00586-010-1521-8

Hicks GE, 2005, ARCH PHYS MED REHAB, V86, P1753, DOI 10.1016/j.apmr.2005.03.033

Hides J A, 2001, Spine (Phila Pa 1976), V26, pE243, DOI 10.1097/00007632-200106010-00004

Jolles BM, 2005, J CLIN EPIDEMIOL, V58, P791, DOI 10.1016/j.jclinepi.2005.01.012

Jones M, 2007, PEDIATR EXERC SCI, V19, P349, DOI 10.1123/pes.19.3.349

Kamper SJ, 2009, J MAN MANIP THER, V17, P163, DOI 10.1179/jmt.2009.17.3.163

Maughan EF, 2010, EUR SPINE J, V19, P1484, DOI 10.1007/s00586-010-1353-6

Schmitt JS, 2014, PHYS THER, V94, P534, DOI 10.2522/ptj.20130162

Stratford P., 1995, PHYSIOTHER CAN, V47, P258, DOI DOI 10.3138/PTC.47.4.258

Westaway MD, 1998, J ORTHOP SPORT PHYS, V27, P331, DOI 10.2519/jospt.1998.27.5.331

Williamson A, 2005, J CLIN NURS, V14, P798, DOI 10.1111/j.1365-2702.2005.01121.x

Zapata KA, 2015, PEDIATR PHYS THER, V27, P119, DOI 10.1097/PEP.0000000000000131

NR 20

TC 7

Z9 7

U1 0

U2 25

PU LIPPINCOTT WILLIAMS & WILKINS

PI PHILADELPHIA

PA TWO COMMERCE SQ, 2001 MARKET ST, PHILADELPHIA, PA 19103 USA

SN 0898-5669

EI 1538-005X

J9 PEDIATR PHYS THER

JI Pediatr. Phys. Ther.

PD JAN

PY 2017

VL 29

IS 1

BP 62

EP 66

DI 10.1097/PEP.0000000000000325

PG 5

WC Pediatrics; Rehabilitation

WE Science Citation Index Expanded (SCI-EXPANDED)

SC Pediatrics; Rehabilitation

GA EH6DI

UT WOS:000391862500019

PM 27984472

DA 2023-08-10

ER

PT J

AU Schreiber, S

Parent, EC

Moez, EK

Hedden, DM

Hill, DL

Moreau, M

Lou, E

Watkins, EM

Southon, SC

AF Schreiber, Sanja

Parent, Eric C.

Moez, Elham Khodayari

Hedden, Douglas M.

Hill, Douglas L.

Moreau, Marc

Lou, Edmond

Watkins, Elise M.

Southon, Sarah C.

TI Schroth Physiotherapeutic Scoliosis-Specific Exercises Added to the

Standard of Care Lead to Better Cobb Angle Outcomes in Adolescents with

Idiopathic Scoliosis - an Assessor and Statistician Blinded Randomized

Controlled Trial

SO PLOS ONE

LA English

DT Article

ID REDUCE SPINAL DEFORMITY; ACTIVE SELF-CORRECTION; QUALITY-OF-LIFE; CURVE

PROGRESSION; BRACE PRESCRIPTION; MONTICONE M; CAZZANIGA D; AMBROSINI E;

BACK-PAIN; ROCCA B

AB Background

The North American non-surgical standard of care for adolescent idiopathic scoliosis (AIS) includes observation and bracing, but not exercises. Schroth physiotherapeutic scoliosis-specific exercises (PSSE) showed promise in several studies of suboptimal methodology. The Scoliosis Research Society calls for rigorous studies supporting the role of exercises before including it as a treatment recommendation for scoliosis.

Objectives

To determine the effect of a six-month Schroth PSSE intervention added to standard of care (Experimental group) on the Cobb angle compared to standard of care alone (Control group) in patients with AIS.

Methods

Fifty patients with AIS aged 10-18 years, with curves of 10 degrees-45 degrees and Risser grade 0-5 were recruited from a single pediatric scoliosis clinic and randomized to the Experimental or Control group. Outcomes included the change in the Cobb angles of the Largest Curve and Sum of Curves from baseline to six months. The intervention consisted of a 30-45 minute daily home program and weekly supervised sessions. Intention-to-treat and per protocol linear mixed effects model analyses are reported.

Results

In the intention-to-treat analysis, after six months, the Schroth group had significantly smaller Largest Curve than controls (-3.5 degrees, 95% CI-1.1 degrees to-5.9 degrees, p = 0.006). Likewise, the between-group difference in the square root of the Sum of Curves was -0.40 degrees, (95% CI -0.03 degrees to -0.8 degrees, p= 0.046), suggesting that an average patient with 51.2 degrees at baseline, will have a 49.3 degrees Sum of Curves at six months in the Schroth group, and 55.1 degrees in the control group with the difference between groups increasing with severity. Per protocol analyses produced similar, but larger differences: Largest Curve = -4.1 degrees (95% CI-1.7 degrees to -6.5 degrees, p= 0.002) and root Sum of Curves = degrees 0.5 (95% CI-0.8 to 0.2, p = 0.006).

Conclusion

Schroth PSSE added to the standard of care were superior compared to standard of care alone for reducing the curve severity in patients with AIS.

C1 [Schreiber, Sanja; Watkins, Elise M.] Univ Alberta, Fac Rehabil Med, Edmonton, AB, Canada.

[Parent, Eric C.] Univ Alberta, Dept Phys Therapy, Edmonton, AB, Canada.

[Moez, Elham Khodayari] Univ Alberta, Sch Publ Hlth, Edmonton, AB, Canada.

[Hedden, Douglas M.; Hill, Douglas L.; Moreau, Marc; Lou, Edmond; Southon, Sarah C.] Univ Alberta, Alberta Hlth Serv, Dept Surg, Edmonton, AB, Canada.

[Hedden, Douglas M.; Hill, Douglas L.; Moreau, Marc; Southon, Sarah C.] Alberta Hlth Serv, Edmonton, AB, Canada.

[Lou, Edmond] Alberta Hlth Serv, Glenrose Rehabil Res Ctr, Edmonton, AB, Canada.

C3 University of Alberta; University of Alberta; University of Alberta;

Alberta Health Services (AHS); University of Alberta; Alberta Health

Services (AHS); Alberta Health Services (AHS)

RP Schreiber, S (通讯作者)， Univ Alberta, Fac Rehabil Med, Edmonton, AB, Canada.; Parent, EC (通讯作者)， Univ Alberta, Dept Phys Therapy, Edmonton, AB, Canada.

EM sanja.schreiber@ualberta.ca; eparent@ualberta.ca

RI Schreiber, Sanja/AAQ-2974-2021

OI Schreiber, Sanja/0000-0002-8231-5131; Khodayari Moez, Elham/0000-0002-9639-4205; Parent, Eric/0000-0003-3835-0607

FU Scoliosis Research Society Small Exploratory Grant; Glenrose Rehabilitation Hospital Foundation; Glenrose Clinical Research Fund; Faculty of Medicine and Dentistry and Faculty of Rehabilitation Medicine

FX This study was funded by: Scoliosis Research Society 2010 Small Exploratory Grant (US$ 10,000), ECP, EMW, SS, DLH, DMH, MJM, SCS, http://www.srs.org/professionals/research-and-journal/research-grants/grants-awarded; Glenrose Rehabilitation Hospital Foundation, Glenrose Clinical Research Fund (CAD$ 10,000), ECP, EMW, DLH, MJM, http://www.albertahealthservices.ca/Facilities/GRH/page58.asp; Interdepartmental Graduate Studentship jointly awarded by the Faculty of Medicine and Dentistry and Faculty of Rehabilitation Medicine supported PhD work of Sanja Schreiber (CAD$ 23,000/annum for 4 years). The funders had no role in study design, data collection and analysis, decision to publish, or preparation of the manuscript.

CR [Anonymous], 2005, ZHONGGUO LINCHUANG K

Bettany-Saltikov J, 2014, EUR J PHYS REHAB MED, V50, P111

Danielsson AJ, 2003, SPINE, V28, pE373, DOI 10.1097/01.BRS.0000084267.41183.75

Danielsson AJ, 2003, SPINE, V28, P2078

Dishman RK, 2002, PREV MED, V34, P100, DOI 10.1006/pmed.2001.0959

Fitzmaurice GM., 2011, APPL LONGITUDINAL AN

Fusco C., 2011, Physiotherapy Theory and Practice, V27, P80, DOI 10.3109/09593985.2010.533342

Hennes A, 2011, SCHROTH METHOD

Konieczny MR, 2013, J CHILD ORTHOP, V7, P3, DOI 10.1007/s11832-012-0457-4

Kotwicki Tomasz, 2009, Ortop Traumatol Rehabil, V11, P379

Kuru T, 2016, CLIN REHABIL, V30, P181, DOI 10.1177/0269215515575745

Lenssinck MLB, 2005, PHYS THER, V85, P1329, DOI 10.1093/ptj/85.12.1329

LONSTEIN JE, 1984, J BONE JOINT SURG AM, V66A, P1061, DOI 10.2106/00004623-198466070-00013

Lonstein JE, 1984, J BONE JOINT SURG AM

MACLEAN WE, 1989, J PEDIATR ORTHOPED, V9, P257

Martinez-Llorens J, 2010, EUR RESPIR J, V36, P393, DOI 10.1183/09031936.00025509

Monticone M, 2014, EUR SPINE J, V23, P2221, DOI 10.1007/s00586-014-3465-x

Monticone M, 2014, EUR SPINE J, V23, P1204, DOI 10.1007/s00586-014-3241-y

Mordecai SC, 2012, EUR SPINE J, V21, P382, DOI 10.1007/s00586-011-2063-4

Negrini S, 2005, Eura Medicophys, V41, P183

Negrini S, 2008, J REHABIL MED, V40, P451, DOI 10.2340/16501977-0195

Negrini S, 2008, DISABIL REHABIL, V30, P731, DOI 10.1080/09638280801889485

Negrini S, 2006, SCOLIOSIS SPINAL DIS, V1, DOI 10.1186/1748-7161-1-14

Negrini Stefano, 2003, Pediatr Rehabil, V6, P227, DOI: 10.1080/13638490310001636781

Negrini S, 2012, SCOLIOSIS SPINAL DIS, V7, DOI 10.1186/1748-7161-7-3

Negrini S, 2015, SCOLIOSIS SPINAL DIS, V10, DOI 10.1186/s13013-014-0025-4

Negrini S, 2014, EUR SPINE J, V23, P2218, DOI 10.1007/s00586-014-3464-y

Noh DK, 2014, J BACK MUSCULOSKELET, V27, P331, DOI 10.3233/BMR-130452

Otman S, 2005, SAUDI MED J, V26, P1429

Payne WK, 1997, SPINE, V22, P1380, DOI 10.1097/00007632-199706150-00017

Plaszewski M, 2014, PLOS ONE, V9, DOI 10.1371/journal.pone.0110254

R Core Team, 2020, R LANG ENV STAT COMP

REFSUM HE, 1990, SPINE, V15, P420, DOI 10.1097/00007632-199005000-00014

Richards BS, 2005, SPINE, V30, P2068, DOI 10.1097/01.brs.0000178819.90239.d0

Rigo M, 2003, Pediatr Rehabil, V6, P209

Romano M, 2012, COCHRANE DB SYST REV, DOI 10.1002/14651858.CD007837.pub2

Roy-Beaudry M, 2010, STUD HEALTH TECHNOL, V158, P152, DOI 10.3233/978-1-60750-573-0-152

Salkind N, 2010, ENCY RES DESIGN, DOI 1041.35/9781412961288.n147

Sanders JO, 2007, J BONE JOINT SURG AM, V89A, P64, DOI 10.2106/JBJS.F.00067

Schlosser TPC, 2014, PLOS ONE, V9, DOI 10.1371/journal.pone.0097461

Schreiber S, 2015, EFFECT SCHROTH EXERC, V10, P1

Schreiber S, 2012, BIOMED CENTRAL LT S1, V7, pO53

Schreiber Sanja, 2014, J Physiother, V60, P234, DOI 10.1016/j.jphys.2014.08.005

Scoliosis Research Society, AD ID SCOL TREATM

Scoliosis Research Society, 2016, AD ID SCOL, P1

Stokes I. A. F., 2002, Journal of Musculoskeletal & Neuronal Interactions, V2, P277

Stokes IA, 2006, SCOLIOSIS, V18, P1

Stokes IAF, 2004, SPINE, V29, P2103, DOI 10.1097/01.brs.0000141182.42544.1f

Stokes IAF, 2007, EUR SPINE J, V16, P1621, DOI 10.1007/s00586-007-0442-7

Tan KJ, 2009, SPINE, V34, P697, DOI 10.1097/BRS.0b013e31819c9431

Venables W.N., 2002, MODERN APPL STAT S

Watkins EM, 2012, ALGORITHMS PRESCR S1, V7, pP22

Weinstein SL, 2008, LANCET, V371, P1527, DOI 10.1016/S0140-6736(08)60658-3

Weinstein SL, 2013, NEW ENGL J MED, V369, P1512, DOI 10.1056/NEJMoa1307337

Weiss Hans-Rudolf, 2003, Pediatr Rehabil, V6, P23, DOI 10.1080/1363849031000095288

Westrick ER, 2011, J PEDIATR ORTHOPED, V31, pS61, DOI 10.1097/BPO.0b013e3181fd87d5

Zhang JH, 2010, J SPINAL DISORD TECH, V23, P383, DOI 10.1097/BSD.0b013e3181bb9a3c

NR 57

TC 56

Z9 60

U1 3

U2 55

PU PUBLIC LIBRARY SCIENCE

PI SAN FRANCISCO

PA 1160 BATTERY STREET, STE 100, SAN FRANCISCO, CA 94111 USA

SN 1932-6203

J9 PLOS ONE

JI PLoS One

PD DEC 29

PY 2016

VL 11

IS 12

AR e0168746

DI 10.1371/journal.pone.0168746

PG 17

WC Multidisciplinary Sciences

WE Science Citation Index Expanded (SCI-EXPANDED)

SC Science & Technology - Other Topics

GA EG7KQ

UT WOS:000391226900042

PM 28033399

OA gold, Green Published, Green Submitted

DA 2023-08-10

ER

PT J

AU Alves, VLD

Avanzi, O

AF Dos Santos Alves, Vera Lucia

Avanzi, Osmar

TI RESPIRATORY MUSCLE STRENGTH IN IDIOPATHIC SCOLIOSIS AFTER TRAINING PROGRAM

SO ACTA ORTOPEDICA BRASILEIRA

LA English

DT Article

DE Scoliosis; Muscle strength; Exercise therapy

ID PULMONARY-FUNCTION; EXERCISE; ADOLESCENTS; MILD; REHABILITATION

AB Objective: To analyze the impact of a physiotherapy protocol in maximum inspiratory and expiratory pressure in patients with adolescent idiopathic scoliosis (AIS) by manovacuometry. AIS may change the respiratory dynamics and the performance of inspiratory and expiratory muscles, affecting ventilatory capacity. Methods: Patients with AIS aged 10 to 20 years old were randomly assigned to receive an aerobic exercise-training program or no treatment. They were evaluated for respiratory muscle strength before and after the treatment period by means of manovacuometry, thorax and spine radiographs. Physical therapy exercising protocol comprised three weekly sessions including stretching and aerobic exercises during four months. Results: Forty five patients received physical therapy and 45 patients received no treatment (control group). The mean maximum inspiratory pressure (Pi(max)) was -52.13 cm H2O and the maximum expiratory pressure (Pe(max)) was 62.38 cm H2O. There was a significant increase of Pi(max) and Pe(max) (p=0,000) in the group receiving physical therapy. There were no drop-outs and no adverse events in this study. Respiratory muscle strength, scoliosis and kyphosis degrees were not statistically correlated. Conclusion: Exercising is beneficial to patients with AIS, who have shown significant increases in respiratory muscle strength after physical therapy. There was no correlation between respiratory pressure and spine deformity.

C1 [Dos Santos Alves, Vera Lucia; Avanzi, Osmar] Santa Casa de Misericordia Sao Paulo, Sao Paulo, SP, Brazil.

RP Alves, VLD (通讯作者)，Av Dr Arnaldo 2088, BR-01255000 Sao Paulo, SP, Brazil.

EM fisioterapiasc@uol.com.br

RI avanzi, osmar/D-1964-2012; Alves, Vera Lúcia dos Santos/D-2385-2012;

alves, vera/IAO-0451-2023

OI Alves, Vera Lúcia dos Santos/0000-0002-9623-8704;

CR Aldrich TK, 2002, AM J RESP CRIT CARE, V166, P548, DOI 10.1164/rccm.166.4.518

[Anonymous], 1994, PHYS ACTIVITY HLTH I

Barrios C, 2005, SPINE, V30, P1610, DOI 10.1097/01.brs.0000169447.55556.01

BLACK LF, 1969, AM REV RESPIR DIS, V99, P696

Cobb JR., 1948, INSTR COURSE LECT, V5, P261

Covey MK, 1999, MED SCI SPORT EXER, V31, P1257, DOI 10.1097/00005768-199909000-00005

DIROCCO PJ, 1988, ARCH PHYS MED REHAB, V69, P198

Alves VLD, 2006, CHEST, V130, P500, DOI 10.1378/chest.130.2.500

Dourado VZ, 2006, CHEST, V129, P551, DOI 10.1378/chest.129.3.551

Helbling D, 1997, RESP PHYSIOL, V109, P219, DOI 10.1016/S0034-5687(97)00054-6

KEARON C, 1993, AM REV RESPIR DIS, V148, P295, DOI 10.1164/ajrccm/148.2.295

Kotani T, 2004, SPINE, V29, P298, DOI 10.1097/01.BRS.0000106490.82936.89

Koumbourlis AC, 2006, PAEDIATR RESPIR REV, V7, P152, DOI 10.1016/j.prrv.2006.04.009

Lacasse Y, 2004, SWISS MED WKLY, V134, P601

Lenke LG, 2002, SPINE, V27, P2041, DOI 10.1097/00007632-200209150-00014

SHNEERSON JM, 1980, THORAX, V35, P347, DOI 10.1136/thx.35.5.347

SMYTH RJ, 1984, THORAX, V39, P901, DOI 10.1136/thx.39.12.901

Takahashi S, 2007, SPINE, V32, P106, DOI 10.1097/01.brs.0000251005.31255.25

Weinstein SL, 2008, LANCET, V371, P1527, DOI 10.1016/S0140-6736(08)60658-3

Windisch W, 2004, EUR RESPIR J, V23, P708, DOI 10.1183/09031936.04.00136104

Zaba Ryszard, 2003, Przegl Lek, V60 Suppl 6, P73

NR 21

TC 5

Z9 5

U1 7

U2 15

PU ATHA COMUNICACAO & EDITORA

PI SAO PAULO SP

PA RUA MACHADO BITTENCOURT, 190-40 ANDAR, CONJ 410, SAO PAULO SP, 00000, BRAZIL

SN 1413-7852

EI 1809-4406

J9 ACTA ORTOP BRAS

JI Acta Ortop. Bras.

PD NOV-DEC

PY 2016

VL 24

IS 6

BP 296

EP 299

DI 10.1590/1413-785220162406120752

PG 4

WC Orthopedics

WE Science Citation Index Expanded (SCI-EXPANDED)

SC Orthopedics

GA EJ0RZ

UT WOS:000392919400002

PM 28924353

OA Green Submitted, Green Published, gold

DA 2023-08-10

ER

PT J

AU Shen, JX

Lin, YX

Luo, JM

Xiao, Y

AF Shen, Jianxiong

Lin, Youxi

Luo, Jinmei

Xiao, Yi

TI Cardiopulmonary Exercise Testing in Patients with Idiopathic Scoliosis

SO JOURNAL OF BONE AND JOINT SURGERY-AMERICAN VOLUME

LA English

DT Article

ID PULMONARY-FUNCTION TESTS; SURGICAL-TREATMENT; CHEST CAGE; CAPACITY;

CHILDREN; ADOLESCENTS; LIMITATION; DEFORMITY; MODERATE; DISEASE

AB Background: Scoliosis causes impairment of the respiratory and cardiovascular systems. Traditional pulmonary function tests only examine patients under static conditions. The aim of our study was to investigate the correlation between radiographic parameters and dynamic cardiopulmonary capacity in patients with idiopathic scoliosis.

Methods: Forty patients with idiopathic scoliosis were included in this prospective study from January 2014 to February 2016. The patients underwent full radiographic assessment of deformity, pulmonary function testing, and cardiopulmonary bicycle ergometer testing. The impact of the severity of thoracic curvature and kyphosis on pulmonary function and physical capacity was investigated.

Results: Thirty-three female patients with a mean age of 15.5 years (range, 11 to 35 years) and coronal thoracic curvature of 49.4 degrees (range, 24 degrees to 76 degrees) and 7 male subjects with a mean age of 15.9 years (range, 13 to 18 years) and coronal thoracic curvature of 47.1 degrees (range, 22 degrees to 80 degrees) were included. No correlation was found between coronal thoracic curvature and pulmonary function test results in the female patients. Female patients with a thoracic curve of >= 60 degrees had lower blood oxygen saturation at maximal exercise in the cardiopulmonary exercise test ($p = 0.032$). Female patients with a thoracic curve of >= 50 degrees had a higher respiratory rate ($p = 0.041$) and ventilation volume per minute ($p = 0.046$) and lower breathing reserve at maximal exercise ($p = 0.038$). Thoracic kyphosis in female patients was positively correlated with pulmonary function, as shown by the forced expiratory volume in 1 second ($r = 0.456$, $p = 0.01$), forced vital capacity ($r = 0.366$, $p = 0.043$), vital capacity ($r = 0.525$, $p = 0.006$), and total lung capacity ($r = 0.388$, $p = 0.031$), as well as with tidal volume ($r = 0.401$, $p = 0.025$) in cardiopulmonary exercise testing. Female patients who engaged in regular exercise had better peak oxygen intake normalized by body weight ($p < 0.001$), peak oxygen intake normalized by the predicted value ($p = 0.003$), maximumheart rate ($p = 0.020$), and heart rate reserve ($p = 0.014$).

Conclusions: Overall exercise tolerance was not correlated with the magnitude of the thoracic curve and kyphosis. Some parameters of ventilatory function and pulmonary gas exchange worsened as thoracic curvature increased or kyphosis decreased. Exercise capacity was better in patients who engaged in regular aerobic exercise, and physical activity is recommended for patients with idiopathic scoliosis.

C1 [Shen, Jianxiong; Lin, Youxi] Chinese Acad Med Sci, Peking Union Med Coll Hosp, Peking Union Med Coll, Dept Orthoped, Beijing, china.

[Luo, Jinmei; Xiao, Yi] Chinese Acad Med Sci, Peking Union Med Coll Hosp, Peking Union Med Coll, Dept Resp Med, Beijing, china.

C3 Chinese Academy of Medical Sciences - Peking Union Medical College;

Peking Union Medical College; Peking Union Medical College Hospital;

Chinese Academy of Medical Sciences - Peking Union Medical College;

Peking Union Medical College; Peking Union Medical College Hospital

RP Shen, JX (通讯作者)， Chinese Acad Med Sci, Peking Union Med Coll Hosp, Peking Union Med Coll, Dept Orthoped, Beijing, china.

EM shenjianxiong@medmail.com.cn

RI Lin, Youxi/AEY-8541-2022; Shen, Jianxiong/AAH-6200-2019

CR Asher MA, 2006, SCOLIOSIS SPINAL DIS, V1, DOI 10.1186/1748-7161-1-2

ATS, 2003, AM J RESP CRIT CARE, V167, P211, DOI 10.1164/rccm.167.2.211

Balady GJ, 2010, CIRCULATION, V122, P191, DOI 10.1161/CIR.0b013e3181e52e69

Barois A, 1999, B ACAD NAT MED PARIS, V183, P721

Barrios C, 2005, SPINE, V30, P1610, DOI 10.1097/01.brs.0000169447.55556.01

Bas P, 2011, EUR SPINE J, V20, P415, DOI 10.1007/s00586-011-1902-7

BJURE J, 1969, ACTA ORTHOP SCAND, V40, P325, DOI 10.3109/17453676908989511

Borel JC, 2009, RESP PHYSIOL NEUROBI, V167, P168, DOI 10.1016/j.resp.2009.03.014

Boyer J, 1996, CHEST, V109, P1532, DOI 10.1378/chest.109.6.1532

Buckley JP, 2011, APPL PHYSIOL NUTR ME, V36, P682, DOI [10.1139/h11-078, 10.1139/H11-078]

Budweiser S, 2006, ARCH PHYS MED REHAB, V87, P1559, DOI 10.1016/j.apmr.2006.08.340

Czaprowski D, 2012, EUR SPINE J, V21, P1099, DOI 10.1007/s00586-011-2068-z

Dreimann M, 2014, SPINE, V39, P2024, DOI 10.1097/BRS.0000000000000601

Giles LV, 2014, MED SCI SPORT EXER, V46, P1999, DOI 10.1249/MSS.0000000000000309

Greiner KA, 2002, AM FAM PHYSICIAN, V65, P1817

Guenette JA, 2013, PULM MED, V2013, DOI 10.1155/2013/956081

HEY EN, 1966, RESP PHYSIOL, V1, P193, DOI 10.1016/0034-5687(66)90016-8

Huh Seokwon, 2015, Korean J Pediatr, V58, P218, DOI 10.3345/kjp.2015.58.6.218

Johnston CE, 2011, SPINE, V36, P1096, DOI 10.1097/BRS.0b013e3181f8c931

KAFER ER, 1976, J CLIN INVEST, V58, P825, DOI 10.1172/JCI108535

KEARON C, 1993, AM REV RESPIR DIS, V148, P295, DOI 10.1164/ajrccm/148.2.295

Lao LF, 2013, J ORTHOP SURG RES, V8, DOI 10.1186/1749-799X-8-32

Leong JCY, 1999, SPINE, V24, P1310, DOI 10.1097/00007632-199907010-00007

Liu JM, 2012, CHINESE MED J-PEKING, V125, P249, DOI 10.3760/cma.j.issn.0366-6999.2012.02.016

Martinez-Llorens J, 2010, EUR RESPIR J, V36, P393, DOI 10.1183/09031936.00025509

McPhail GL, 2015, J PEDIATR-US, V166, P1018, DOI 10.1016/j.jpeds.2014.12.070

Menon B, 2007, NEUROSCIENCES, V12, P293

MITCHELL JH, 1958, J CLIN INVEST, V37, P538, DOI 10.1172/JCI103636

Mohammadi P, 2014, PHYSIOTHER THEOR PR, V30, P552, DOI 10.3109/09593985.2014.938382

Newton PO, 2005, J BONE JOINT SURG AM, V87A, P1937, DOI 10.2106/JBJS.D.02209

Parent S, 2002, ST HEAL T, V88, P387

Sperandio EF, 2014, SPINE J, V14, P2366, DOI 10.1016/j.spinee.2014.01.041

Stickland MK, 2012, PULM MED, V2012, DOI 10.1155/2012/824091

Tsiligiannis T, 2012, SCOLIOSIS SPINAL DIS, V7, DOI 10.1186/1748-7161-7-7

Verges S, 2009, RESP PHYSIOL NEUROBI, V169, P282, DOI 10.1016/j.resp.2009.09.005

Wagner PD, 1996, ANNU REV PHYSIOL, V58, P21, DOI 10.1146/annurev.physiol.58.1.21

Xue XH, 2015, EUR SPINE J, V24, P1415, DOI 10.1007/s00586-014-3327-6

NR 37

TC 14

Z9 15

U1 1

U2 9

PU LIPPINCOTT WILLIAMS & WILKINS

PI PHILADELPHIA

PA TWO COMMERCE SQ, 2001 MARKET ST, PHILADELPHIA, PA 19103 USA

SN 0021-9355

EI 1535-1386

J9 J BONE JOINT SURG AM

JI J. Bone Joint Surg.-Am. Vol.

PD OCT 5

PY 2016

VL 98

IS 19

BP 1614

EP 1622

DI 10.2106/JBJS.15.01403

PG 9

WC Orthopedics; Surgery

WE Science Citation Index Expanded (SCI-EXPANDED)

SC Orthopedics; Surgery

GA EN4AL

UT WOS:000395949500011

PM 27707847

DA 2023-08-10

ER

PT J

AU Monticone, M

Ambrosini, E

Cazzaniga, D

Rocca, B

Motta, L

Cerri, C

Brayda-Bruno, M

Lovi, A

AF Monticone, Marco

Ambrosini, Emilia

Cazzaniga, Daniele

Rocca, Barbara

Motta, Lorenzo

Cerri, Cesare

Brayda-Bruno, Marco

Lovi, Alessio

TI Adults with idiopathic scoliosis improve disability after motor and

cognitive rehabilitation: results of a randomised controlled trial

SO EUROPEAN SPINE JOURNAL

LA English

DT Article

DE Adult scoliosis; Self-correction; Task-oriented exercises;

Cognitive-behavioural therapy; Randomised controlled trial

ID CROSS-CULTURAL ADAPTATION; QUALITY-OF-LIFE; SPINAL DEFORMITY;

NONOPERATIVE TREATMENT; NONSURGICAL TREATMENT; CURVE PROGRESSION; LUMBAR

SCOLIOSIS; ITALIAN VERSION; BACK-PAIN; FOLLOW-UP

AB To evaluate the effects of motor and cognitive rehabilitation on disability in adults with idiopathic scoliosis at lower risk of progression.

130 adults with idiopathic scoliosis (main curve < 35A degrees) were randomly assigned to a 20-week rehabilitation programme consisting of active self-correction, task-oriented exercises and cognitive-behavioural therapy (experimental group, 65 subjects, mean age of 51.6, females 48) or general physiotherapy consisting of active and passive mobilizations, stretching, and strengthening exercises of the spinal muscles (control group, 65 subjects, mean age of 51.7, females 46). Before, at the end, and 12 months after treatment, each participant completed the Oswestry disability index (ODI) (primary outcome), the Tampa scale for kinesiophobia, the pain catastrophizing scale, a pain numerical rating scale, and the Scoliosis Research Society-22 Patient Questionnaire. Radiological (Cobb angle) and clinical deformity (angle of trunk rotation) changes were also investigated. A linear mixed model for repeated measures was used for each outcome.

Significant effects of time, group, and time by group interaction were found for all outcome measures (P < 0.001). After training, the primary outcome showed a clinically significant between-group change (12 % points), which was preserved at follow-up. At follow-up, the radiological deformities showed a significant, although not clinically meaningful, between-group difference of 4A degrees in favour of the experimental group.

The experimental programme was superior to general physiotherapy in reducing disability of adults with idiopathic scoliosis. Motor and cognitive rehabilitation also led to improvements in dysfunctional thoughts, pain, and quality of life. Changes were maintained for at least 1 year.

C1 [Monticone, Marco; Ambrosini, Emilia; Cazzaniga, Daniele; Rocca, Barbara] Salvatore Maugeri Fdn, Inst Care & Res IRCCS, Sci Inst Lissone, Phys Med & Rehabil Unit, Via Monsignor Bernasconi 16, I-20851 Lissone, Monza Brianza, Italy.

[Ambrosini, Emilia] Politecn Milan, Neuroengn & Med Robot Lab, Dept Elect Informat & Bioengn, Milan, Italy.

[Motta, Lorenzo; Cerri, Cesare] Bicocca Univ Milan, Sch Phys & Rehabil Med, Milan, Italy.

[Brayda-Bruno, Marco; Lovi, Alessio] Inst Care & Res IRCCS, Galeazzi Orthoped Inst, Dept Spinal Surg 3, Milan, Italy.

C3 Istituti Clinici Scientifici Maugeri IRCCS; Polytechnic University of

Milan; University of Milano-Bicocca; IRCCS Istituto Ortopedico Galeazzi

RP Monticone, M (通讯作者)，Salvatore Maugeri Fdn, Inst Care & Res IRCCS, Sci Inst Lissone, Phys Med & Rehabil Unit, Via Monsignor Bernasconi 16, I-20851 Lissone, Monza Brianza, Italy.

EM marco.monticone@fsm.it

RI Ambrosini, Emilia/R-1371-2016; Monticone, Marco/AAC-4494-2022;

Cazzaniga, Daniele/F-8914-2019; Lovi, Alessio/AHE-1127-2022;

Brayda-Bruno, Marco/B-8639-2017

OI Ambrosini, Emilia/0000-0002-6527-0779; Cazzaniga,

Daniele/0000-0001-8248-5790; Lovi, Alessio/0000-0001-7564-8897;

Brayda-Bruno, Marco/0000-0002-8608-0114

CR Aebi M, 2005, EUR SPINE J, V14, P925, DOI 10.1007/s00586-005-1053-9

Barrios C, 2005, SPINE, V30, P1610, DOI 10.1097/01.brs.0000169447.55556.01

Bess S, 2009, SPINE, V34, P2186, DOI 10.1097/BRS.0b013e3181b05146

Blum Charles L, 2002, J Manipulative Physiol Ther, V25, pE3, DOI 10.1067/mmt.2002.123336

Bridwell KH, 2009, SPINE, V34, P2171, DOI 10.1097/BRS.0b013e3181a8fdc8

Brooks WJ, 2009, SCOLIOSIS SPINAL DIS, V4, DOI 10.1186/1748-7161-4-27

BUNNELL WP, 1984, J BONE JOINT SURG AM, V66A, P1381, DOI 10.2106/00004623-198466090-00010

BUNNELL WP, 1993, SPINE, V18, P1572, DOI 10.1097/00007632-199309000-00001

Cooper Grant, 2004, Pain Physician, V7, P311

Crombez G, 1999, PAIN, V80, P329, DOI 10.1016/S0304-3959(98)00229-2

DAVIS CM, 2002, J GERIATR PHYS THER, V25, P33

Everett CR, 2007, SPINE, V32, pS130, DOI 10.1097/BRS.0b013e318134ea88

Fielding S, 2012, CONTEMP CLIN TRIALS, V33, P461, DOI 10.1016/j.cct.2011.12.002

Haefeli M, 2006, SPINE, V31, P355, DOI 10.1097/01.brs.0000197664.02098.09

Hales Jeremiah, 2002, Spine (Phila Pa 1976), V27, pE71, DOI 10.1097/00007632-200202010-00012

Hawes MC, 2001, CHEST, V120, P672, DOI 10.1378/chest.120.2.672

HUSKISSON EC, 1974, LANCET, V2, P1127, DOI 10.1016/S0140-6736(74)90884-8

Kluba T, 2009, ARCH ORTHOP TRAUM SU, V129, P1, DOI 10.1007/s00402-008-0673-z

Li G, 2009, SPINE, V34, P2165, DOI 10.1097/BRS.0b013e3181b3ff0c

Lonstein JE, 2006, CLIN ORTHOP RELAT R, P248, DOI 10.1097/01.blo.0000198725.54891.73

Lowe T, 2006, SPINE, V31, pS119, DOI 10.1097/01.brs.0000232709.48446.be

Mamyama Toni, 2002, Stud Health Technol Inform, V91, P361

Marty-Poumarat C, 2007, SPINE, V32, P1227, DOI 10.1097/01.brs.0000263328.89135.a6

Monticone M, 2014, EUR SPINE J, V23, P1204, DOI 10.1007/s00586-014-3241-y

Monticone M, 2012, QUAL LIFE RES, V21, P1045, DOI 10.1007/s11136-011-0007-4

Monticone M, 2012, EUR SPINE J, V21, P122, DOI 10.1007/s00586-011-1959-3

Monticone M, 2010, SPINE, V35, pE1412, DOI 10.1097/BRS.0b013e3181e88981

Monticone M, 2010, SPINE, V35, P1241, DOI 10.1097/BRS.0b013e3181bfcbf6

Monticone M, 2009, SPINE, V34, P2090, DOI 10.1097/BRS.0b013e3181aa1e6b

Morningstar MW, 2004, BMC MUSCULOSKEL DIS, V5, DOI 10.1186/1471-2474-5-32

MORRISSY RT, 1990, J BONE JOINT SURG AM, V72A, P320, DOI 10.2106/00004623-199072030-00002

Negrini A, 2008, SCOLIOSIS SPINAL DIS, V3, DOI 10.1186/1748-7161-3-20

Paulus MC, 2014, SPINE, V39, P388, DOI 10.1097/BRS.0000000000000150

Schwab F, 2005, SPINE, V30, P1082, DOI 10.1097/01.brs.0000160842.43482.cd

Schwab F, 2003, SPINE, V28, P602, DOI 10.1097/00007632-200303150-00016

Schwab FJ, 2008, SPINE, V33, P2243, DOI 10.1097/BRS.0b013e31817d1d4e

Shea KG, 1998, SPINE, V23, P551, DOI 10.1097/00007632-199803010-00007

Siddiqui O, 2009, J BIOPHARM STAT, V19, P227, DOI 10.1080/10543400802609797

Tanure MC, 2010, SPINE J, V10, P769, DOI 10.1016/j.spinee.2010.02.020

TAROLA GA, 1994, J MANIP PHYSIOL THER, V17, P253

TORELL G, 1985, SPINE, V10, P425, DOI 10.1097/00007632-198506000-00004

van Middelkoop M, 2011, EUR SPINE J, V20, P19, DOI 10.1007/s00586-010-1518-3

WEINSTEIN SL, 1983, J BONE JOINT SURG AM, V65, P447, DOI 10.2106/00004623-198365040-00004

WEINSTEIN SL, 1981, J BONE JOINT SURG AM, V63, P702, DOI 10.2106/00004623-198163050-00003

Weinstein SL, 2003, JAMA-J AM MED ASSOC, V289, P559, DOI 10.1001/jama.289.5.559

Weiss HR, 2006, ST HEAL T, V123, P582

Weiss HR, 2006, STUD HEALTH TECHNOL, V123, P586

NR 47

TC 24

Z9 25

U1 0

U2 18

PU SPRINGER

PI NEW YORK

PA ONE NEW YORK PLAZA, SUITE 4600, NEW YORK, NY, UNITED STATES

SN 0940-6719

EI 1432-0932

J9 EUR SPINE J

JI Eur. Spine J.

PD OCT

PY 2016

VL 25

IS 10

BP 3120

EP 3129

DI 10.1007/s00586-016-4528-y

PG 10

WC Clinical Neurology; Orthopedics

WE Science Citation Index Expanded (SCI-EXPANDED)

SC Neurosciences & Neurology; Orthopedics

GA DY5ZY

UT WOS:000385186100015

PM 27015689

DA 2023-08-10

ER

PT J

AU Porte, M

Patte, K

Dupeyron, A

Cottalorda, J

AF Porte, M.

Patte, K.

Dupeyron, A.

Cottalorda, J.

TI Exercise therapy in the treatment of idiopathic adolescent scoliosis: Is

it useful?

SO ARCHIVES DE PEDIATRIE

LA French

DT Article

ID OF-THE-LITERATURE; EFFICACY; PROGRAM; GIRLS; BRACE

AB Many practitioners, pediatricians, and general practitioners prescribe physical therapy when tracking scoliosis. However, has physical therapy alone proved its efficacy in the care of the scoliosis to slow down progression? Our purpose is to report the results of a literature review on the effectiveness of rehabilitation in idiopathic scoliosis. No current study presents sufficient scientific proof to validate the efficacy of isolated exercise therapy in scoliosis. Learned societies recognize, however, the efficacy of combining conservative therapy (brace + physiotherapy) in idiopathic scoliosis. Should we then still prescribe rehabilitation without brace treatment? Although physical therapy alone does not seem effective in treating scoliosis, it can limit potential painful phenomena and be beneficial for respiratory function. The physical therapist can also teach the teenager the classic principles of hygiene of the back. It may therefore be appropriate to prescribe physical therapy, but the principles and objectives must be explained to the patient and family in light of current evidence-based medicine. (C) 2016 Elsevier Masson SAS. All rights reserved.

C1 [Porte, M.; Dupeyron, A.] CHU Nimes, Serv Med Phys & Readaptat, Pl Pr Robert Debre, F-30029 Nimes 9, France.

[Porte, M.; Patte, K.] Inst St Pierre, Unite Reeduc, 371 Ave Eveche de Maguelone, F-34250 Palavas Les Flots, France.

[Cottalorda, J.] CHU Montpellier, Serv Orthopedie Infantile, 371 Ave Doyen Gaston Giraud, F-34295 Montpellier 5, France.

C3 Universite de Montpellier; CHU de Nimes; Universite de Montpellier; CHU

de Montpellier

RP Porte, M (通讯作者)， CHU Nimes, Serv Med Phys & Readaptat, Pl Pr Robert Debre, F-30029 Nimes 9, France.; Porte, M (通讯作者)， Inst St Pierre, Unite Reeduc, 371 Ave Eveche de Maguelone, F-34250 Palavas Les Flots, France.

EM melanie.porte@chu-nimes.fr

CR Athanasopoulos S, 1999, SCAND J MED SCI SPOR, V9, P36

Callens C., 2008, KINESIR REV, V80, P14

Coelho L, 2010, ACTA REUMATOL PORT, V35, P406

den Boer WA, 1999, EUR SPINE J, V8, P406, DOI 10.1007/s005860050195

Iunes DH, 2010, BRAZ J PHYS THER, V14, P133, DOI 10.1590/S1413-35552010005000009

Mallet JF, 2012, MONOGRAPHIE SOFOP, P209

Maruyama Toru, 2003, Pediatr Rehabil, V6, P215

Mordecai SC, 2012, EUR SPINE J, V21, P382, DOI 10.1007/s00586-011-2063-4

NACHEMSON AL, 1995, J BONE JOINT SURG AM, V77A, P815, DOI 10.2106/00004623-199506000-00001

Negrini S, 2008, EUR J PHYS REHAB MED, V44, P169

Negrini S, 2008, DISABIL REHABIL, V30, P772, DOI 10.1080/09638280801889568

Negrini S, 2008, J REHABIL MED, V40, P451, DOI 10.2340/16501977-0195

Negrini Stefano, 2003, Pediatr Rehabil, V6, P227, DOI: 10.1080/13638490310001636781

Negrini S, 2009, SCOLIOSIS SPINAL DIS, V4, DOI 10.1186/1748-7161-4-2

Negrini S, 2009, SCOLIOSIS SPINAL DIS, V4, DOI 10.1186/1748-7161-4-19

Otman S, 2005, SAUDI MED J, V26, P1429

Rigo M, 2003, Pediatr Rehabil, V6, P209

Stagnara P, 1978, REEDUCATION SCOLIOSE

STONE B, 1979, PHYS THER, V59, P759, DOI 10.1093/ptj/59.6.759

Weiss H R, 1992, Ital J Orthop Traumatol, V18, P395

Weiss HR, 2006, SCOLIOSIS SPINAL DIS, V1, DOI [10.1186/1748-7161-1-5, 10.1186/1748-7161-1-1]

Weiss Hans-Rudolf, 2003, Pediatr Rehabil, V6, P183

Weiss Hans-Rudolf, 2003, Pediatr Rehabil, V6, P23, DOI 10.1080/1363849031000095288

Weiss HR, 2011, SCOLIOSIS SPINAL DIS, V6, DOI 10.1186/1748-7161-6-17

Weiss HR, 2009, STUD HEALTH TECHNOL, V135, P173

WEISS HR, 1991, SPINE, V16, P88, DOI 10.1097/00007632-199101000-00016

NR 26

TC 3

Z9 3

U1 0

U2 43

PU ELSEVIER FRANCE-EDITIONS SCIENTIFIQUES MEDICALES ELSEVIER

PI PARIS

PA 23 RUE LINOIS, 75724 PARIS, FRANCE

SN 0929-693X

EI 1769-664X

J9 ARCH PEDIATRIE

JI Arch. Pediatr.

PD JUN

PY 2016

VL 23

IS 6

BP 624

EP 628

DI 10.1016/j.arcped.2016.03.004

PG 5

WC Pediatrics

WE Science Citation Index Expanded (SCI-EXPANDED)

SC Pediatrics

GA DO4ER

UT WOS:000377735100014

PM 27117993

DA 2023-08-10

ER

PT J

AU Putzier, M

Gro, C

Zahn, RK

Pumberger, M

Strube, P

AF Putzier, M.

Gro, C.

Zahn, R. K.

Pumberger, M.

Strube, P.

TI Characteristics of neuromuscular scoliosis

SO ORTHOPADE

LA German

DT Article

DE Braces; Orthosis; Scoliosis; Spondylodesis; Treatment

ID DUCHENNE MUSCULAR-DYSTROPHY; ADOLESCENT IDIOPATHIC SCOLIOSIS; SPINAL-FUSION; PEDICLE SCREWS; HOOK INSTRUMENTATION; SURGICAL-MANAGEMENT; PULMONARY-FUNCTION; NATURAL-HISTORY; CEREBRAL-PALSY; RISK-FACTORS

AB Usually, neuromuscular scolioses become clinically symptomatic relatively early and are rapidly progressive even after the end of growth. Without sufficient treatment they lead to a severe reduction of quality of life, to a loss of the ability of walking, standing or sitting as well as to an impairment of the cardiopulmonary system resulting in an increased mortality. Therefore, an intensive interdisciplinary treatment by physio- and ergotherapists, internists, pediatricians, orthotists, and orthopedists is indispensable. In contrast to idiopathic scoliosis the treatment of patients with neuromuscular scoliosis with orthosis is controversially discussed, whereas physiotherapy is established and essential to prevent contractures and to maintain the residual sensorimotor function.

Frequently, the surgical treatment of the scoliosis is indicated. It should be noted that only long-segment posterior correction and fusion of the whole deformity leads to a significant improvement of the quality of life as well as to a prevention of a progression of the scoliosis and the development of junctional problems. The surgical intervention is usually performed before the end of growth. A prolonged delay of surgical intervention does not result in an increased height but only in a deformity progression and is therefore not justifiable. In early onset neuromuscular scolioses guided-growth implants are used to guarantee the adequat development. Because of the high complication rates, further optimization of these implant systems with regard to efficiency and safety have to be addressed in future research.

C1 [Putzier, M.; Zahn, R. K.; Pumberger, M.] Charite, Orthopad Klin, Ctr Muskuloskeletale Chirurg, Charite Pl 1, D-10117 Berlin, Germany.

[Gro, C.] Helios Klinikum Emil von Behring, Klinr Orthopadie & Unfallchirurg, Berlin, Germany.

[Strube, P.] Univ Jena, Klin Orthopadie, Campus Waldkrankenhaus Rudolf Elle GmbH, Eisenberg, Germany.

C3 Free University of Berlin; Humboldt University of Berlin; Charite Universitatsmedizin Berlin; Helios Kliniken; Friedrich Schiller University of Jena

RP Putzier, M (通讯作者)， Charite, Orthopad Klin, Ctr Muskuloskeletale Chirurg, Charite Pl 1, D-10117 Berlin, Germany.

EM michael.putzier@charite.de

RI Strube, Patrick/AAH-5408-2019; Pumberger, Matthias/AAH-9086-2019

OI Strube, Patrick/0000-0003-3210-5301; Zahn, Robert
Karl/0000-0001-6362-1507; Pumberger, Matthias/0000-0002-0885-7370

CR Abul-Kasim Kasim, 2010, J Orthop Surg (Hong Kong), V18, P1
Akbarnia BA, 2011, J CHILD ORTHOP, V5, P159, DOI 10.1007/s11832-011-0342-6
Akbarnia BA, 2010, SPINE, V35, P2193, DOI 10.1097/BRS.0b013e3181f070b5
Arun R, 2010, EUR SPINE J, V19, P376, DOI 10.1007/s00586-009-1163-x
Barr SJ, 1997, SPINE, V22, P1369, DOI 10.1097/00007632-199706150-00016
Benson ER, 1998, SPINE, V23, P2308, DOI 10.1097/00007632-199811010-00012
Berven S, 2002, SEMIN NEUROL, V22, P167, DOI 10.1055/s-2002-36540
Bridwell KH, 1999, SPINE, V24, P1300, DOI 10.1097/00007632-199907010-00006
Brunner R, 2002, ORTHOPADE, V31, P51, DOI 10.1007/s132-002-8274-5
Canavese F, 2014, WORLD J ORTHOP, V5, P124, DOI 10.5312/wjo.v5.i2.124
Carstens C, 1999, ORTHOPADE, V28, P622
CARSTENS C, 1990, Z ORTHOP GRENZGEB, V128, P174, DOI 10.1055/s-2008-1039496
Comstock CP, 1998, SPINE, V23, P1412, DOI 10.1097/00007632-199806150-00022
Di Silvestre M, 2007, SPINE, V32, P1655, DOI 10.1097/BRS.0b013e318074d604
DUPORT G, 1995, SEMIN NEUROL, V15, P29, DOI 10.1055/s-2008-1041004
Eagle M, 2002, NEUROMUSCULAR DISORD, V12, P926, DOI 10.1016/S0960-8966(02)00140-2
Flynn JM, 2011, CLIN ORTHOP RELAT R, V469, P1291, DOI 10.1007/s11999-010-1620-1
Forst R, 1997, Z ORTHOP GRENZGEB, V135, P95, DOI 10.1055/s-2008-1039563
Fujak A, 2010, ORTHOPADE, V39, P38, DOI 10.1007/s00132-009-1536-1
Furderer S, 1999, EUR SPINE J, V8, P451, DOI 10.1007/s005860050204
Gaine WJ, 2004, J BONE JOINT SURG BR, V86B, P550, DOI 10.1302/0301-620X.86B4.14481

GALASKO CSB, 1992, J BONE JOINT SURG BR, V74, P210, DOI 10.1302/0301-620X.74B2.1544954

Gotze C, 2001, Z ORTHOP GRENZGEB, V139, P31, DOI 10.1055/s-2001-11868

Hahn F, 2008, EUR SPINE J, V17, P255, DOI 10.1007/s00586-007-0558-9

Hicks JM, 2010, SPINE, V35, pE465, DOI 10.1097/BRS.0b013e3181d1021a

HSU JD, 1983, SPINE, V8, P771, DOI 10.1097/00007632-198310000-00014

Karol LA, 2008, J BONE JOINT SURG AM, V90A, P1272, DOI 10.2106/JBJS.G.00184

Kim YJ, 2004, SPINE, V29, P2040, DOI 10.1097/01.brs.0000138268.12324.1a

Kotwicki T, 2008, DISABIL REHABIL, V30, P792, DOI 10.1080/09638280801889584

Kotwicki T, 2008, DISABIL REHABIL-ASSI, V3, P161, DOI 10.1080/17483100801905900

Larsson ELC, 2005, SPINE, V30, P2145, DOI 10.1097/01.brs.0000180403.11757.6a

Liljenqvist U, 2002, EUR SPINE J, V11, P336, DOI 10.1007/s00586-002-0415-9

Majd ME, 1997, SPINE, V22, P1461, DOI 10.1097/00007632-199707010-00007

Master DL, 2011, SPINE, V36, P564, DOI 10.1097/BRS.0b013e3181e193e9

Master DL, 2011, SPINE, V36, pE179, DOI 10.1097/BRS.0b013e3181db7afe

McCall RE, 2005, SPINE, V30, P2056, DOI 10.1097/01.brs.0000178817.34368.16

Modi HN, 2010, SCOLIOSIS SPINAL DIS, V5, DOI 10.1186/1748-7161-5-16

Modi Hitesh N, 2008, J Orthop Surg Res, V3, P23, DOI 10.1186/1749-799X-3-23

Mullender MG, 2008, SCOLIOSIS SPINAL DIS, V3, DOI 10.1186/1748-7161-3-14

Olafsson Y, 1999, J PEDIATR ORTHOPED, V19, P376, DOI 10.1097/00004694-199905000-00017

Sarwark J, 2007, ORTHOP CLIN N AM, V38, P485, DOI 10.1016/j.ocl.2007.07.001

Sengupta DK, 2002, SPINE, V27, P2072, DOI 10.1097/00007632-200209150-00020

Sponseller PD, 2009, SPINE, V34, P1706, DOI 10.1097/BRS.0b013e3181ab240e

Tangsrud SE, 2001, ARCH DIS CHILD, V84, P521, DOI 10.1136/adc.84.6.521

Tokala DP, 2007, EUR SPINE J, V16, P91, DOI 10.1007/s00586-006-0105-0

Vitale MG, 2008, SPINE, V33, P1242, DOI 10.1097/BRS.0b013e3181714536

NR 46

TC 8

Z9 10

U1 2

U2 11

PU SPRINGER

PI NEW YORK

PA 233 SPRING ST, NEW YORK, NY 10013 USA

SN 0085-4530

EI 1433-0431

J9 ORTHOPADE

JI Orthopade

PD JUN

PY 2016

VL 45

IS 6

BP 500

EP 508

DI 10.1007/s00132-016-3272-7

PG 9

WC Orthopedics

WE Science Citation Index Expanded (SCI-EXPANDED)

SC Orthopedics

GA DN9PU

UT WOS:000377412700006

PM 27197823

DA 2023-08-10

ER

PT J

AU Kim, G

HwangBo, PN

AF Kim, Gichul

HwangBo, Pil-neo

TI Effects of Schroth and Pilates exercises on the Cobb angle and weight

distribution of patients with scoliosis

SO JOURNAL OF PHYSICAL THERAPY SCIENCE

LA English

DT Article

DE Schroth exercise; Pilates exercise; Cobb angle

ID IDIOPATHIC SCOLIOSIS

AB [Purpose] The purpose of this study was to compare the effect of Schroth and Pilates exercises on the Cobb angle and body weight distribution of patients with idiopathic scoliosis. [Subjects] Twenty-four scoliosis patients with a Cobb angle of >= 20 degrees were divided into the Schroth exercise group (SEG, n = 12) and the Pilates exercise group (PEG, n = 12). [Methods] The SEG and PEG performed Schroth and Pilates exercises, respectively, three times a week for 12 weeks. The Cobb angle was measured in the standing position with a radiography apparatus, and weight load was measured with Gait View Pro 1.0. [Results] In the intragroup comparison, both groups showed significant changes in the Cobb angle. For weight distribution, the SEG showed significant differences in the total weight between the concave and convex sides, but the PEG did not show significant differences. Furthermore, in the intragroup comparison, the SEG showed significant differences in the changes in the Cobb angle and weight distribution compared with the PEG. [Conclusion] Both Schroth and Pilates exercises were effective in changing the Cobb angle and weight distribution of scoliosis patients; however, the intergroup comparison showed that the Schroth exercise was more effective than the Pilates exercise.

C1 [Kim, Gichul] Daegu Hlth Coll, Dept Phys Therapy, Daegu, South Korea.

[HwangBo, Pil-neo] Daegu Univ, Grad Sch Phys Therapy, Dept Phys Therapy, Gyongsan, Gyeongsangbuk D, South Korea.

C3 Daegu University

RP HwangBo, PN (通讯作者)， Daegu Univ, Grad Sch Phys Therapy, Dept Phys Therapy, Gyongsan, Gyeongsangbuk D, South Korea.

EM pt486pn@naver.com

CR de Araujo MEA, 2012, J BODYW MOV THER, V16, P191, DOI 10.1016/j.jbmt.2011.04.002

[Anonymous], 2015, THESIS

Dasuji C, 2010, THESIS

Emery K, 2010, CLIN BIOMECH, V25, P124, DOI 10.1016/j.clinbiomech.2009.10.003

Fusco C., 2011, Physiotherapy Theory and Practice, V27, P80, DOI 10.3109/09593985.2010.533342

Gauchard GC, 2001, SPINE, V26, P1052, DOI 10.1097/00007632-200105010-00014

Junghee K, 2004, KOREAN J DANCE RES, V4, P53

Kim B. Y., 2014, THESIS

Kramers-de Quervain IA, 2004, EUR SPINE J, V13, P449, DOI 10.1007/s00586-003-0588-x

Landauer Franz, 2003, Pediatr Rehabil, V6, P201

Lenssinck MLB, 2005, PHYS THER, V85, P1329, DOI 10.1093/ptj/85.12.1329

Mooney V, 2000, J SPINAL DISORD, V13, P102, DOI 10.1097/00002517-200004000-00002

Park JY, 2013, J PHYS THER SCI, V25, P1629, DOI 10.1589/jpts.25.1629

Trobisch P, 2010, DTSCH ARZTEBL INT, V107, P875, DOI 10.3238/arztebl.2010.0875

Weiss HR, 2010, SCOLIOSIS SPINAL DIS, V5, DOI 10.1186/1748-7161-5-22

Yang JM, 2015, J PHYS THER SCI, V27, P2667, DOI 10.1589/jpts.27.2667

Zabjek KF, 2001, EUR SPINE J, V10, P107, DOI 10.1007/s005860000244

이준호, 2014, [Journal of the Korean Society of Physical Medicine, 대한물리의학회지], V9, P11

NR 18

TC 24

Z9 26

U1 9

U2 59

PU SOC PHYSICAL THERAPY SCIENCE

PI TOKYO

PA C/O PUBLICATION CENTER, 1-24-12 SUGAMO, TOSHIMA-KU, TOKYO, 170-0002, JAPAN

SN 0915-5287

EI 2187-5626

J9 J PHYS THER SCI

JI J. Phys. Ther. Sci.

PD MAR

PY 2016

VL 28

IS 3

BP 1012

EP 1015

DI 10.1589/jpts.28.1012

PG 4

WC Rehabilitation

WE Science Citation Index Expanded (SCI-EXPANDED)

SC Rehabilitation

GA DP9ZB

UT WOS:000378855600058

PM 27134403

OA Green Published, gold, Green Submitted

DA 2023-08-10

ER

PT J

AU Kim, KD

Hwangbo, PN

AF Kim, Kyoung-Don

Hwangbo, Pil-Neo

TI Effects of the Schroth exercise on the Cobb's angle and vital capacity

of patients with idiopathic scoliosis that is an operative indication

SO JOURNAL OF PHYSICAL THERAPY SCIENCE

LA English

DT Article

DE Schroth exercise; Cobb's angle; Scoliosis

ID ADULT

AB [Purpose] The purpose of this study was to investigate the effects of the Schroth exercise on the Cobb's angle and vital capacity of patients with growing idiopathic scoliosis, an operative indication. [Subjects] Five idiopathic scoliosis patients with a Cobb's angle of the thoracic vertebra of 40 degrees or higher and Risser sign stage 3 or higher. [Methods] The Schroth exercise was applied 3 times a week for 12 weeks. We measured the thoracic trunk inclination, Cobb's angle, and vital capacity before and after the exercise program. [Results] The thoracic trunk rotation angle decreased from 11.86 +/- 3.32 degrees to 4.90 +/- 1.91 degrees on average, the thoracic Cobb's angle decreased from 42.40 +/- 7.86 degrees to 26.0 +/- 3.65 degrees on average, and the vital capacity also increased from 2.83 +/- 1.23 degrees to 4.04 degrees +/- 1.67 degrees on average. All these effects were significant. [Conclusion] The 12-week Schroth exercise caused significant effects in the thoracic trunk inclination, Cobb's angle, and vital capacity. The conservative treatment method was found to be effective even at a 40 degree or higher Cobb's angle. In the future, universal exercise approach methods and preventive training for the treatment of scoliosis should be developed further.

C1 [Kim, Kyoung-Don] Daegu Fatima Hosp, Daegu, South Korea.

[Hwangbo, Pil-Neo] Daegu Univ, Grad Sch Phys Therapy, Dept Phys Therapy, Gyongsan, Gyeongsangbuk D, South Korea.

C3 Daegu Fatima Hospital; Daegu University

RP Hwangbo, PN (通讯作者)， Daegu Univ, Grad Sch Phys Therapy, Dept Phys Therapy, Gyongsan, Gyeongsangbuk D, South Korea.

EM pt486pn@naver.com

CR Benameur S, 2003, COMPUT MED IMAG GRAP, V27, P321, DOI 10.1016/S0895-6111(03)00019-3

Borysov M, 2012, SCOLIOSIS SPINAL DIS, V7, DOI 10.1186/1748-7161-7-1

Brooks WJ, 2009, SCOLIOSIS SPINAL DIS, V4, DOI 10.1186/1748-7161-4-27

Cassar-Pullicino VN, 2002, CLIN RADIOL, V57, P543, DOI 10.1053/crad.2001.0909

Fusco C., 2011, Physiotherapy Theory and Practice, V27, P80, DOI 10.3109/09593985.2010.533342

Kim B. Y., 2014, THESIS

Kim JJ, 2015, J PHYS THER SCI, V27, P1775, DOI 10.1589/jpts.27.1775

Kotwicki T, 2008, J PEDIATR ORTHOPED, V28, P225, DOI 10.1097/BPO.0b013e3181647c4a

Landauer Franz, 2003, Pediatr Rehabil, V6, P201

Lee M, 2013, J PHYS THER SCI, V25, P445, DOI 10.1589/jpts.25.445

Lehnert-Schroth C, 2007, PHYSIOTHERAPEUTIC ME

Lehnert-Schroth C, 2010, 3 DIMENSIONAL TREATM

LONSTEIN JE, 1994, LANCET, V344, P1407

Mahaudens P, 2013, EUR SPINE J, V22, P2399, DOI 10.1007/s00586-013-2837-y

Negrini S, 2008, DISABIL REHABIL, V30, P772, DOI 10.1080/09638280801889568

Negrini Stefano, 2003, Pediatr Rehabil, V6, P227, DOI: 10.1080/13638490310001636781

Otman Saadet, 2005, Neurosciences (Riyadh), V10, P277

Park JY, 2013, J PHYS THER SCI, V25, P1629, DOI 10.1589/jpts.25.1629

Rigo M, 2009, STUD HEALTH TECHNOL, V135, P208

Roach JW, 1999, ORTHOP CLIN N AM, V30, P353, DOI 10.1016/S0030-5898(05)70092-4

Trobisch P, 2010, DTSCH ARZTEBL INT, V107, P875, DOI 10.3238/arztebl.2010.0875

Weiss HR, 2010, SCOLIOSIS SPINAL DIS, V5, DOI 10.1186/1748-7161-5-22

Weiss HR, 2007, PATIENT SAF SURG, V1, DOI 10.1186/1754-9493-1-7

WEISS HR, 1991, SPINE, V16, P88, DOI 10.1097/00007632-199101000-00016

Yang JM, 2015, J PHYS THER SCI, V27, P2667, DOI 10.1589/jpts.27.2667

Zabjek KF, 2001, EUR SPINE J, V10, P107, DOI 10.1007/s005860000244

NR 26

TC 12

Z9 13

U1 2

U2 22

PU SOC PHYSICAL THERAPY SCIENCE

PI TOKYO

PA C/O PUBLICATION CENTER, 1-24-12 SUGAMO, TOSHIMA-KU, TOKYO, 170-0002, JAPAN

SN 0915-5287

EI 2187-5626

J9 J PHYS THER SCI

JI J. Phys. Ther. Sci.

PD MAR

PY 2016

VL 28

IS 3

BP 923

EP 926

DI 10.1589/jpts.28.923

PG 4

WC Rehabilitation

WE Science Citation Index Expanded (SCI-EXPANDED)

SC Rehabilitation

GA DP9ZB

UT WOS:000378855600040

PM 27134385

OA Green Published, gold

DA 2023-08-10

ER

PT J

AU Noh, DK

Koh, JH

You, JH

AF Noh, Dong Koog

Koh, Jae-Hyun

You, Joshua (Sung)-H.

TI Inter- and intratester reliability values of ultrasound imaging

measurements of diaphragm movement in the thoracic and thoracolumbar

curves in adolescent idiopathic scoliosis

SO PHYSIOTHERAPY THEORY AND PRACTICE

LA English

DT Article

DE Adolescent idiopathic scoliosis; reliability; diaphragm movement;

ultrasound imaging

ID CHILDREN; DYSFUNCTION

AB Purpose: The purpose of this study was to determine intertester and intratester reliability of ultrasound measurements of bilateral diaphragm excursions in the thoracic and thoracolumbar spinal curves of 31 females with adolescent idiopathic scoliosis (AIS) (mean age = 14.1 +/- 1.8 years). Method: Subjects were tested during tidal breathing using real-time ultrasound imaging with a 3.5 MHz curvilinear transducer. Results: There were no significant differences in intratester and intertester reliability values in bilateral diaphragmatic excursions measured at the thoracolumbar spinal curve, whereas significant differences were observed in measurements taken at the thoracic spinal curve ($p < 0.05$). Overall, the intertester and intratester reliabilities of the thoracic and thoracolumbar curves in AIS ranged from 0.764 to 0.998. Conclusions: These findings suggest that ultrasound imaging is highly reliable between and within testers and is useful to precisely discriminate pathological diaphragm movement in idiopathic thoracic scoliosis and idiopathic thoracolumbar scoliosis.

C1 [Noh, Dong Koog; Koh, Jae-Hyun] Seoul Hyu Rehabil Hosp, Dept Phys Med & Rehabil, Gyeonggi Do, South Korea.

[You, Joshua (Sung)-H.] Yonsei Univ, Dept Phys Therapy, Coll Hlth Sci, Kangwon Do, South Korea.

C3 Yonsei University

RP Noh, DK (通讯作者)， 1 Yonseidae Kil, Wonju 220710, Kangwon Do, South Korea.

EM neurorehab@yonsei.ac.kr

CR [Anonymous], 2016, SPSS WINDOWS STEP ST

[Anonymous], 2007, FDN CLIN RES APPL PR

Ayoub J, 2001, ANESTH ANALG, V92, P755

Bagnall KM, 2009, SCOLIOSIS SPINAL DIS, V4, DOI 10.1186/1748-7161-4-28

BAKE B, 1972, THORAX, V27, P703, DOI 10.1136/thx.27.6.703

Blaney F, 1999, AUST J PHYSIOTHER, V45, P41

Boussuges A, 2009, CHEST, V135, P391, DOI 10.1378/chest.08-1541

Campbell RM, 2004, J BONE JOINT SURG AM, V86A, P1659, DOI 10.2106/00004623-200408000-00009

COHEN E, 1994, THORAX, V49, P890, DOI 10.1136/thx.49.9.890

CRESSWELL AG, 1994, EXP BRAIN RES, V98, P336

Gerscovich EO, 2001, J ULTRAS MED, V20, P597

HIDES JA, 1994, SPINE, V19, P165, DOI 10.1097/00007632-199401001-00009

Hodges PW, 2000, J PHYSIOL-LONDON, V522, P165, DOI 10.1111/j.1469-7793.2000.t01-1-00165.xm

HOUSTON JG, 1995, J NEUROL NEUROSUR PS, V58, P738, DOI 10.1136/jnnp.58.6.738

HOUSTON JG, 1994, THORAX, V49, P500, DOI 10.1136/thx.49.5.500

HOUSTON JG, 1992, CLIN RADIOL, V46, P405, DOI 10.1016/S0009-9260(05)80688-9

Kalpakcioglu B, 2009, J BACK MUSCULOSKELET, V22, P27, DOI 10.3233/BMR-2009-0212

Kim WY, 2011, CRIT CARE MED, V39, P2627, DOI 10.1097/CCM.0b013e3182266408

Lenke LG, 2001, SPINE, V26, P2347, DOI 10.1097/00007632-200111010-00011

Lerolle N, 2009, CHEST, V135, P401, DOI 10.1378/chest.08-1531

Noh DK, 2014, BIO-MED MATER ENG, V24, P947, DOI 10.3233/BME-130889

Redding G, 2008, SPINE J, V8, P639, DOI 10.1016/j.spinee.2007.04.020

Redding GJ, 2011, PEDIAT ALLER IMM PUL, V24, P89, DOI 10.1089/ped.2011.0080

RIMMER KP, 1995, J APPL PHYSIOL, V79, P1556, DOI 10.1152/jappl.1995.79.5.1556

URVOAS E, 1994, PEDIATR RADIOL, V24, P564, DOI 10.1007/BF02012733

NR 25

TC 8

Z9 9

U1 0

U2 4

PU TAYLOR & FRANCIS INC

PI PHILADELPHIA

PA 530 WALNUT STREET, STE 850, PHILADELPHIA, PA 19106 USA

SN 0959-3985

EI 1532-5040

J9 PHYSIOTHER THEOR PR

JI Physiother. Theory Pract.

PD FEB 17

PY 2016

VL 32

IS 2

BP 139

EP 143

DI 10.3109/09593985.2015.1091871

PG 5

WC Rehabilitation

WE Science Citation Index Expanded (SCI-EXPANDED)

SC Rehabilitation

GA DG0GO

UT WOS:000371743500008

PM 26863479

DA 2023-08-10

ER

PT J

AU Kuru, T

Yeldan, I

Dereli, EE

Ozdincler, AR

Dikici, F

Colak, I

AF Kuru, Tugba

Yeldan, Ipek

Dereli, E. Elcin

Ozdincler, Arzu R.

Dikici, Fatih

Colak, Ilker

TI The efficacy of three-dimensional Schroth exercises in adolescent

idiopathic scoliosis: a randomised controlled clinical trial

SO CLINICAL REHABILITATION

LA English

DT Article

DE Adolescent idiopathic scoliosis; exercise; Schroth method

ID PUBERTAL CHANGES; RELIABILITY; VALIDITY; PATTERN

AB Objective: To compare the efficacy of three-dimensional (3D) Schroth exercises in patients with adolescent idiopathic scoliosis.

Design: A randomised-controlled study.

Setting: An outpatient exercise-unit and in a home setting.

Subjects: Fifty-one patients with adolescent idiopathic scoliosis.

Interventions: Forty-five patients with adolescent idiopathic scoliosis meeting the inclusion criteria were divided into three groups. Schroth's 3D exercises were applied to the first group in the clinic and were given as a home program for the second group; the third group was the control.

Main Measures: Scoliosis angle (Cobb method), angle of rotation (scoliometer), waist asymmetry (waist - elbow distance), maximum hump height of the patients and quality of life (QoL) (SRS-23) were assessed pre-treatment and, at the 6(th), 12(th) and 24(th) weeks.

Results: The Cobb (-2.53 degrees; P=0.003) and rotation angles (-4.23 degrees; P=0.000) significantly decreased, which indicated an improvement in the clinic exercise group compared to the other groups. The gibbosity (-68.66mm; P=0.000) and waist asymmetry improved only in the clinic exercise group, whereas the results of the other groups worsened. QoL did not change significantly in either group.

Conclusion: According to the results of this study the Schroth exercise program applied in the clinic under physiotherapist supervision was superior to the home exercise and control groups; additionally, we observed that scoliosis progressed in the control group, which received no treatment.

C1 [Kuru, Tugba] Marmara Univ, Dept Physiotherapy & Rehabil, Fac Hlth Sci, TR-34865 Istanbul, Turkey.

[Yeldan, Ipek; Ozdincler, Arzu R.] Istanbul Univ, Fac Hlth Sci, Dept Physiotherapy & Rehabil, Istanbul, Turkey.

[Dereli, E. Elcin] Istanbul Bilgi Univ, Sch Hlth Sci, Dept Physiotherapy & Rehabil, Istanbul, Turkey.

[Dikici, Fatih] Istanbul Univ, Fac Med, Dept Orthopaed & Traumatol, Istanbul, Turkey.

[Colak, Ilker] Kartal Educ & Res Hosp, Dept Orthopaed & Traumatol, Istanbul, Turkey.

C3 Marmara University; Istanbul University; Istanbul Bilgi University;

Istanbul University; Istanbul Kartal Dr Lutfi Kirdar Training & Research

Hospital

RP Kuru, T (通讯作者)， Marmara Univ, Dr Lutfi Kirdar Kartal Egitim Arastirma Hastanes, Suzan Yazici Acil Tip Merkezi Yani, Dept Physiotherapy & Rehabil,Fac Hlth Sci, E-5 Yanyol Uzeri, TR-34865 Istanbul, Turkey.

EM tugbakuru@gmail.com

RI Kuru, Tuğba/AAC-6019-2020; Dereli, Elif Elçin E/G-1829-2015; Çolak,

Tuğba Kuru/AAC-5785-2020; Çolak, İlker/AAZ-6799-2020; YELDAN,

IPEK/E-4649-2019

OI YELDAN, IPEK/0000-0002-6344-4157

CR Alanay A, 2005, SPINE, V30, P2464, DOI 10.1097/01.brs.0000184366.71761.84

AMENDT LE, 1990, PHYS THER, V70, P108, DOI 10.1093/ptj/70.2.108

Freidel K, 2002, SPINE, V27, pE87, DOI 10.1097/00007632-200202150-00013

Hawes MC, 2006, SCOLIOSIS SPINAL DIS, V1, DOI 10.1186/1748-7161-1-3

Hawes Martha C, 2003, Pediatr Rehabil, V6, P171

Herring J.A., 2002, TACHDJIANS PEDIAT OR, V3rd ed., P213

Kotwicki T, 2008, STUD HEALTH TECHNOL, V140, P44, DOI 10.3233/978-1-58603-888-5-44

Lenhert-Schroth C., 2007, SCHROTH SCOLIOSIS 3

Lenhert-Schroth C., 1992, PHYSIOTHERAPY, V78, P810, DOI [10.1016/S0031-9406(10)60451-8, DOI 10.1016/S0031-9406(10)60451-8]

MARSHALL WA, 1969, ARCH DIS CHILD, V44, P291, DOI 10.1136/adc.44.235.291

MARSHALL WA, 1970, ARCH DIS CHILD, V45, P13, DOI 10.1136/adc.45.239.13

Negrini A, 2001, EUR MED PHYS, V37, P181

Negrini S, 2008, J REHABIL MED, V40, P451, DOI 10.2340/16501977-0195

Negrini S, 2012, SCOLIOSIS SPINAL DIS, V7, DOI 10.1186/1748-7161-7-3

Otman S, 2005, SAUDI MED J, V26, P1429

Rigo M, 2003, Pediatr Rehabil, V6, P209

Romano M, 2013, SPINE, V38, pE883, DOI 10.1097/BRS.0b013e31829459f8

Vasiliadis E, 2009, STUD HEALTH TECHNOL, V135, P409

Weiss H, 2007, BEST PRACTISE CONSER

Weiss HR, 2006, SCOLIOSIS SPINAL DIS, V1, DOI [10.1186/1748-7161-1-5, 10.1186/1748-7161-1-1]

Weiss Hans-Rudolf, 2003, Pediatr Rehabil, V6, P183

[No title captured]

[No title captured]

[No title captured]

[No title captured]

[No title captured]

[No title captured]

[No title captured]

NR 28

TC 92

Z9 99

U1 8

U2 77

PU SAGE PUBLICATIONS LTD

PI LONDON

PA 1 OLIVERS YARD, 55 CITY ROAD, LONDON EC1Y 1SP, united kingdom

SN 0269-2155

EI 1477-0873

J9 CLIN REHABIL

JI Clin. Rehabil.

PD FEB

PY 2016

VL 30

IS 2

BP 181

EP 190

DI 10.1177/0269215515575745

PG 10

WC Rehabilitation

WE Science Citation Index Expanded (SCI-EXPANDED)

SC Rehabilitation

GA CZ9XB

UT WOS:000367449800009

PM 25780260

DA 2023-08-10

ER

PT J

AU Zapata, KA

Wang-Price, SS

Sucato, DJ

Thompson, M

Trudelle-Jackson, E

Lovelace-Chandler, V

AF Zapata, Karina Amani

Wang-Price, Sharon S.

Sucato, Daniel J.

Thompson, Mary

Trudelle-Jackson, Elaine

Lovelace-Chandler, Venita

TI Spinal Stabilization Exercise Effectiveness for Low Back Pain in

Adolescent Idiopathic Scoliosis: A Randomized Trial

SO PEDIATRIC PHYSICAL THERAPY

LA English

DT Article

DE adolescent; exercise therapy/methods; female; human; low back

pain/physiopathology; low back pain/therapy; male; muscle

strength/physiology; pain measurement; patient education as topic;

physical therapy/methods; questionnaires; scoliosis; self-care;

treatment outcomes

ID DISABILITY QUESTIONNAIRE; CHILDREN; PREVALENCE; PREDICTORS; SCALE

AB Purpose: To compare 8 weeks of weekly supervised spinal stabilization exercises with 1-time treatment in participants with low back pain and adolescent idiopathic scoliosis. Methods: Participants were randomly assigned to the supervised or unsupervised group. Seventeen participants in the supervised group received weekly physical therapy, and 17 participants in the unsupervised group received a 1-time treatment followed by home exercises. Results: Significant between-group differences were found in the Numeric Pain Rating Scale and the Patient-Specific Functional Scale scores after 8 weeks (P < .01), indicating the supervised group had significantly more pain reduction and functional improvements than the unsupervised group. However, no between-group differences were found in back muscle endurance, the revised Oswestry Back Pain Disability Questionnaire scores, or the Global Rating of Change scores. Conclusions: Supervised physical therapy may be more effective than 1-time treatment in reducing pain and improving function in patients with adolescent idiopathic scoliosis and low back pain. Video Abstract: For more insights from the authors, access Supplemental Digital Content 1, at http://links.lww.com/PPT/A85.

C1 [Zapata, Karina Amani] Texas Scottish Rite Hosp Children, Phys Therapy Dept, Dallas, TX 75219 USA.

[Sucato, Daniel J.] Texas Scottish Rite Hosp Children, Dept Orthopaed, Dallas, TX 75219 USA.

[Wang-Price, Sharon S.; Thompson, Mary; Trudelle-Jackson, Elaine] Texas Womans Univ, Sch Phys Therapy, Dallas, TX USA.

[Lovelace-Chandler, Venita] Univ N Texas, Hlth Sci Ctr, Dept Phys Therapy, Ft Worth, TX USA.

C3 Texas Scottish Rite Hospital for Children; Texas Scottish Rite Hospital

for Children; Texas Womans University; University of North Texas System;

University of North Texas Health Science Center

RP Zapata, KA (通讯作者)， Texas Scottish Rite Hosp Children, 2222 Welborn St, Dallas, TX 75219 USA.

EM Karina.zapata@tsrh.org

OI Thompson, Mary/0000-0002-4830-2222

FU American Physical Therapy Association Section on Pediatrics

FX Grant funding was received from the American Physical Therapy

Association Section on Pediatrics for this work.

CR Ahlqwist A, 2008, SPINE, V33, pE721, DOI 10.1097/BRS.0b013e318182c347

Andersen MO, 2006, SPINE, V31, P350, DOI 10.1097/01.brs.0000197649.29712.de

[Anonymous], 1989, BACK PAIN NEW APPROA

Arab AM, 2007, CLIN REHABIL, V21, P640, DOI 10.1177/0269215507076353

Bailey B, 2010, PAIN, V149, P216, DOI 10.1016/j.pain.2009.12.008

Bystrom MG, 2013, SPINE, V38, pE350, DOI 10.1097/BRS.0b013e31828435fb

Delitto A, 2012, J ORTHOP SPORT PHYS, V42, pA1, DOI 10.2519/jospt.2012.42.4.A1

Fanucchi GL, 2009, AUST J PHYSIOTHER, V55, P97, DOI 10.1016/S0004-9514(09)70039-X

Fritz JM, 2001, PHYS THER, V81, P776, DOI 10.1093/ptj/81.2.776

Haladay DE, 2013, J ORTHOP SPORT PHYS, V43, P242, DOI 10.2519/jospt.2013.4346

Hall AM, 2011, EUR SPINE J, V20, P79, DOI 10.1007/s00586-010-1521-8

Hestbaek L, 2006, SPINE, V31, P468, DOI 10.1097/01.brs.0000199958.04073.d9

Hicks GE, 2005, ARCH PHYS MED REHAB, V86, P1753, DOI 10.1016/j.apmr.2005.03.033

Hides J A, 2001, Spine (Phila Pa 1976), V26, pE243, DOI 10.1097/00007632-200106010-00004

Jeffries LJ, 2007, SPINE, V32, P2630, DOI 10.1097/BRS.0b013e318158d70b

Jones M, 2007, PEDIATR EXERC SCI, V19, P349, DOI 10.1123/pes.19.3.349

Kamper SJ, 2009, J MAN MANIP THER, V17, P163, DOI 10.1179/jmt.2009.17.3.163

Landman Z, 2011, SPINE, V36, P825, DOI 10.1097/BRS.0b013e3181de8c2b

Maughan EF, 2010, EUR SPINE J, V19, P1484, DOI 10.1007/s00586-010-1353-6

McIntosh G, 1998, J REHABIL OUTCOMES M, V2, P20

Ramirez N, 1997, J BONE JOINT SURG AM, V79A, P364, DOI 10.2106/00004623-199703000-00007

Sato T, 2011, EUR SPINE J, V20, P274, DOI 10.1007/s00586-010-1657-6

Smorgick Y, 2013, J PEDIATR ORTHOPED, V33, P289, DOI 10.1097/BPO.0b013e31827d0b43

Stratford P., 1995, PHYSIOTHER CAN, V47, P258, DOI DOI 10.3138/PTC.47.4.258

Williamson A, 2005, J CLIN NURS, V14, P798, DOI 10.1111/j.1365-2702.2005.01121.x

NR 25

TC 15

Z9 20

U1 8

U2 42

PU LIPPINCOTT WILLIAMS & WILKINS

PI PHILADELPHIA

PA TWO COMMERCE SQ, 2001 MARKET ST, PHILADELPHIA, PA 19103 USA

SN 0898-5669

EI 1538-005X

J9 PEDIATR PHYS THER

JI Pediatr. Phys. Ther.

PD WIN

PY 2015

VL 27

IS 4

BP 396

EP 402

DI 10.1097/PEP.0000000000000174

PG 7

WC Pediatrics; Rehabilitation

WE Science Citation Index Expanded (SCI-EXPANDED)

SC Pediatrics; Rehabilitation

GA DD1YP

UT WOS:000369719600017

PM 26397085

DA 2023-08-10

ER

PT J

AU Steffan, K

AF Steffan, K.

TI Physical therapy for idiopathic scoliosis

SO ORTHOPADE

LA German

DT Article

DE Physical therapy modalities; Scoliosis; Ambulatory care; Vojta; Schroth

ID FOLLOW-UP

AB Objective. The objective is the description and summary of the current state of idiopathic scoliosis treatment with physical therapy based on new scientific knowledge and concluded from more than 15 years of experience as a leading physician in two well-known clinics specializing in the conservative treatment of scoliosis.

Materials and methods. Based on current scientific publications on physical therapy in scoliosis treatment and resulting from the considerable personal experience gained working with conservative treatment and consulting scoliosis patients (as inpatients and outpatients), the current methods of physical therapy have been compared and evaluated.

Results. Physical therapy according to Schroth and Vojta therapy are at present the most common and effective methods in the physical treatment of idiopathic scoliosis. These methods can be applied during inpatient or outpatient treatment or intensified in the practice of specialized therapists.

Discussion. As there are only a few scientific studies on this subject, the author's findings are based mainly on his own experiences of the conservative treatment of idiopathic scoliosis. Athough these experiences are the results of over 15 years of working in the field of therapy, and the Schroth method in combination with corrective bracing presents highly promising results, it would nevertheless be desirable to conduct detailed scientific studies to verify the effectiveness of conservative treatment.

C1 [Steffan, K.] Asklepios Katharina Schroth Klin Bad Sobernheim, D-55566 Bad Sobernheim, Germany.

RP Steffan, K (通讯作者)， Asklepios Katharina Schroth Klin Bad Sobernheim, Korczakstr 2, D-55566 Bad Sobernheim, Germany.

EM kay-steffan@t-online.de

CR DEACON P, 1984, J BONE JOINT SURG BR, V66, P509, DOI 10.1302/0301-620X.66B4.6746683

HEINE J, 1972, Z ORTHOP GRENZGEB, V110, P56

Helmond van AF, 1992, VERENDING SCOLIOSEPA

Huber L, 1987, PSYCHOSOZIALE PROBLE

Karch J, 1992, WIRBELSAULENDERFORMI, V2

Karch J, 1989, Z KRANKENGYMNASTIK, V5, P467

Lehnert Schroth C, 2000, DREIDIMENSIONALE SKO

Lohnstein JE, 1984, J BONE JOINT SURG AM, V66-A, P1061

Mariconda M, 2005, EUR SPINE J, V14, P854, DOI 10.1007/s00586-004-0853-7

Mau H, 1982, ATHIOPATHOGENESE SKO

PONSETI IV, 1976, CLIN ORTHOP RELAT R, P268

Pugacheva N, 2012, CORRECTIVE EXERCISES

Reichel Dagmar, 2003, Pediatr Rehabil, V6, P221

Rigo M, 1991, WIRBELSAULENDEFORMIT, V1

Schonberger F, 1987, Z KRANKENGYMNASTIK, V12, P865

Schoning N, 1989, Z KRANKENGYMNASTIK, V5, P461

Singer RN, 1985, MOTORISCHES LERNEN M

Tomaschewski R, 1992, WIRBELSAULENDEFORMIT, V2

Tomaschewski R, 1987, THESIS FAKULTAT WISS

WEINSTEIN SL, 1981, J BONE JOINT SURG AM, V63, P702, DOI 10.2106/00004623-198163050-00003

WEINSTEIN SL, 1986, SPINE, V11, P780, DOI 10.1097/00007632-198610000-00006

Weiss HR, VORTR SEM IND ZIEL K

Weiss HR, 1991, Z KRANKENGYMNASTIK, V4, P361

Weiss HR, 1991, SPINE, V16, P89

Weiss HR, 1998, ORTHOP PRAX, V34, P770

NR 25

TC 6

Z9 6

U1 1

U2 22

PU SPRINGER

PI NEW YORK

PA 233 SPRING ST, NEW YORK, NY 10013 USA

SN 0085-4530

EI 1433-0431

J9 ORTHOPADE

JI Orthopade

PD NOV

PY 2015

VL 44

IS 11

BP 852

EP 858

DI 10.1007/s00132-015-3174-0

PG 7

WC Orthopedics

WE Science Citation Index Expanded (SCI-EXPANDED)

SC Orthopedics

GA CV4FB

UT WOS:000364221000004

PM 26415607

DA 2023-08-10

ER

PT J

AU Yang, JM

Lee, JH

Lee, DH

AF Yang, Jae-Man

Lee, Jung-Hoon

Lee, Dae-Hee

TI Effects of consecutive application of stretching, Schroth, and

strengthening exercises on Cobb's angle and the rib hump in an adult

with idiopathic scoliosis

SO JOURNAL OF PHYSICAL THERAPY SCIENCE

LA English

DT Article

DE Adam's forward bend test; Back pain; Rotational breathing

ID SURGICAL-TREATMENT

AB [Purpose] To report the effects of consecutive application of stretching, Schroth, and strengthening exercises in an adult with idiopathic scoliosis. [Subject] A 26-year-old woman with idiopathic scoliosis, Cobb's angle of 20.51 degrees, and back pain. [Methods] The exercise program consisted of 3 sessions: 10 minutes of stretching exercises, 20 minutes of Schroth exercises, and 10 minutes of strengthening exercises. This program was implemented 3 times a week, for 8 weeks. [Results] The thoracic Cobb's angle decreased from 20.51 degrees to 16.35 degrees, and the rib hump decreased from 15 degrees to 9 degrees. [Conclusion] Consecutive application of stretching, Schroth, and strengthening exercises may help reduce Cobb's angle and the rib hump in adults with idiopathic scoliosis.

C1 [Yang, Jae-Man] Dong Eui Univ, Grad Sch, Dept Biomed Hlth Sci, Busan, South Korea.

[Lee, Jung-Hoon] Dong Eui Univ, Coll Nursing & Healthcare Sci, Dept Phys Therapy, Busan 614714, South Korea.

C3 Dong-Eui University; Dong-Eui University

RP Lee, JH (通讯作者)， Dong Eui Univ, Coll Nursing & Healthcare Sci, Dept Phys Therapy, 176 Eomgwangno, Busan 614714, South Korea.

EM dreampt@hanmail.net

CR Campbell RM, 2004, J BONE JOINT SURG AM, V86A, P1659, DOI 10.2106/00004623-200408000-00009

Cote P, 1998, SPINE, V23, P796, DOI 10.1097/00007632-199804010-00011

Glassman SD, 2005, SPINE, V30, P2024, DOI 10.1097/01.brs.0000179086.30449.96

Green BN, 2009, J CHIROPR MED, V8, P25, DOI 10.1016/j.jcm.2008.11.001

GRUBB SA, 1994, SPINE, V19, P1619, DOI 10.1097/00007632-199407001-00011

Koumbourlis AC, 2006, PAEDIATR RESPIR REV, V7, P152, DOI 10.1016/j.prrv.2006.04.009

Lee M, 2013, J PHYS THER SCI, V25, P445, DOI 10.1589/jpts.25.445

Lehnert-Schroth C., 2007, 3 DIMENSIONAL TREATM, V7th ed.

LEHNERTSCHROTH C, 1992, PHYSIOTHERAPY, V78, P810

Lenssinck MLB, 2005, PHYS THER, V85, P1329, DOI 10.1093/ptj/85.12.1329

Linek P, 2015, J PHYS THER SCI, V27, P465, DOI 10.1589/jpts.27.465

Mooney V, 2000, J SPINAL DISORD, V13, P102, DOI 10.1097/00002517-200004000-00002

MORRISSY RT, 1990, J BONE JOINT SURG AM, V72A, P320, DOI 10.2106/00004623-199072030-00002

Otman S, 2005, SAUDI MED J, V26, P1429

Park JY, 2013, J PHYS THER SCI, V25, P1629, DOI 10.1589/jpts.25.1629

Redding GJ, 2011, CLIN ORTHOP RELAT R, V469, P1330, DOI 10.1007/s11999-010-1621-0

Weinstein SL, 2008, LANCET, V371, P1527, DOI 10.1016/S0140-6736(08)60658-3

Zakaria A, 2012, J PHYS THER SCI, V24, P1127, DOI 10.1589/jpts.24.1127

NR 18

TC 13

Z9 15

U1 0

U2 31

PU SOC PHYSICAL THERAPY SCIENCE

PI TOKYO

PA C/O PUBLICATION CENTER, 1-24-12 SUGAMO, TOSHIMA-KU, TOKYO, 170-0002, JAPAN

SN 0915-5287

EI 2187-5626

J9 J PHYS THER SCI

JI J. Phys. Ther. Sci.

PD AUG

PY 2015

VL 27

IS 8

BP 2667

EP 2669

DI 10.1589/jpts.27.2667

PG 3

WC Rehabilitation

WE Science Citation Index Expanded (SCI-EXPANDED)

SC Rehabilitation

GA CP6SX

UT WOS:000360019600063

PM 26355577

OA Green Published, gold, Green Submitted

DA 2023-08-10

ER

PT J

AU Lee, S

Shim, J

AF Lee, Suemin

Shim, Jemyung

TI The effects of backpack loads and spinal stabilization exercises on the

dynamic foot pressure of elementary school children with idiopathic

scoliosis

SO JOURNAL OF PHYSICAL THERAPY SCIENCE

LA English

DT Article

DE Idiopathic scoliosis; Dynamic foot pressure; Backpack load

ID PLANTAR PRESSURE; GAIT; BALANCE; WALKING; POSTURE; WEIGHT

AB [Purpose] The purpose of this study was to measure and observe the changes in dynamic plantar pressures when school children carried specific bag loads, and to determine whether improved physical balance after an eight-week spinal stabilization exercise program can influences plantar pressures. [Subjects] The subjects were 10 school students with Cobb angles of 10 degrees or greater. [Methods] Gait View Pro 1.0 (Alfoots, Korea) was were based on to measure the pressure of the participants' feet. Spinal stabilization exercises used TOGU Multi-roll Functional (TOGU, Germany) training. Dynamic plantar pressures were measured with bag loads of 0% no bag and 15% of subjects' body weight. The independent t test was performed to analyze changes in plantar pressures. [Results] The plantar pressure measurements of bag load of 0% of subjects' body weight before and after the spinal stabilization exercise program were not significantly different, but those of two foot areas with a 15% load were statistically significant (mt5, 67.32 +/- 24.25 and 51.77 +/- 25.52 kPa; lat heel, 126.00 +/- 20.46 and 102.08 +/- 23.87 kPa). [Conclusion] After performance of the spinal stabilization exercises subjects' overall plantar pressures were reduced, which may suggest that physical balance improved.

C1 [Lee, Suemin] Kangwon Natl Univ, Dept Phys Therapy, Emergency Med Rehabil, Samcheok Si 245905, Gangwon Do, South Korea.

[Shim, Jemyung] Kangwon Natl Univ, Coll Hlth & Sci, Dept Phys Therapy, Samcheok Si 245905, Gangwon Do, South Korea.

C3 Kangwon National University; Kangwon National University

RP Shim, J (通讯作者)，Kangwon Natl Univ, Coll Hlth & Sci, Dept Phys Therapy, 346 Hwangjo Ri, Samcheok Si 245905, Gangwon Do, South Korea.

EM sjm7897@hanmail.net

CR Asher MA, 2006, SCOLIOSIS SPINAL DIS, V1, DOI 10.1186/1748-7161-1-2

Brackley HM, 2004, SPINE, V29, P2184, DOI 10.1097/01.brs.0000141183.20124.a9

Chow DHK, 2006, GAIT POSTURE, V24, P173, DOI 10.1016/j.gaitpost.2005.08.007

Cottalorda J, 2004, ORTHOPEDICS, V27, P1172, DOI 10.3928/0147-7447-20041101-14

Drerup B, 2008, CLIN BIOMECH, V23, P1073, DOI 10.1016/j.clinbiomech.2008.04.014

Giacomozzi C, 2010, GAIT POSTURE, V32, P141, DOI 10.1016/j.gaitpost.2010.03.014

Gong W, 2010, J PHYS THER SCI, V22, P255, DOI 10.1589/jpts.22.255

Hessert Mary Josephine, 2005, BMC Geriatr, V5, P8

Hong YL, 2003, GAIT POSTURE, V17, P28, DOI 10.1016/S0966-6362(02)00050-4

Horimoto Y, 2012, J PHYS THER SCI, V24, P763, DOI 10.1589/jpts.24.763

Kellis E, 2001, GAIT POSTURE, V14, P92, DOI 10.1016/S0966-6362(01)00129-1

Kim YJ, 2007, SPINE, V32, P2685, DOI 10.1097/BRS.0b013e31815a7b17

Laghi F, 2003, AM J RESP CRIT CARE, V168, P10, DOI 10.1164/rccm.2206020

Lee WJ, 2012, J KOREAN PHYS MED, V7, P59, DOI DOI 10.13066/KSPM.2012.7.1.059

Mahaudens P, 2009, EUR SPINE J, V18, P512, DOI 10.1007/s00586-009-0899-7

Monteiro M, 2010, CLIN BIOMECH, V25, P461, DOI 10.1016/j.clinbiomech.2010.01.017

Negrini S, 2006, SCOLIOSIS SPINAL DIS, V1, DOI 10.1186/1748-7161-1-4

Nurse MA, 2001, CLIN BIOMECH, V16, P719, DOI 10.1016/S0268-0033(01)00090-0

Park， Jae-Hyeon, 2008, [Journal of Sensor Science and Technology, 센서학회지], V17, P210

Park JY, 2013, J PHYS THER SCI, V25, P1629, DOI 10.1589/jpts.25.1629

Periyasamy R, 2011, Foot (Edinb), V21, P142, DOI 10.1016/j.foot.2011.03.001

Peterka RJ, 2004, J NEUROPHYSIOL, V91, P410, DOI 10.1152/jn.00516.2003

Rai D., 2006, POLISH J MED PHYS EN, V12, P25

Rateau Margaret R, 2004, Orthop Nurs, V23, P101, DOI 10.1097/00006416-200403000-00004

Reamy BV, 2001, AM FAM PHYSICIAN, V64, P111

Richardson CA, 1999, THERAPEUTIC EXERCISE, P145

Sahli S, 2013, SPINE J, V13, P1835, DOI 10.1016/j.spinee.2013.06.023

Shelton Yvonne A, 2007, Adolesc Med State Art Rev, V18, P121

Simoneau M, 2006, EXP BRAIN RES, V170, P576, DOI 10.1007/s00221-005-0246-0

Son H, 2013, J PHYS THER SCI, V25, P1383, DOI 10.1589/jpts.25.1383

Weinstein SL, 2008, LANCET, V371, P1527, DOI 10.1016/S0140-6736(08)60658-3

Weiss HR, 2006, SCOLIOSIS SPINAL DIS, V1, DOI [10.1186/1748-7161-1-5, 10.1186/1748-7161-1-1]

유재호, 2010, [The Journal of Korean Society of Physical Therapy, 대한물리치료학회지], V22, P65

NR 33

TC 4

Z9 6

U1 0

U2 20

PU SOC PHYSICAL THERAPY SCIENCE

PI TOKYO

PA C/O PUBLICATION CENTER, 1-24-12 SUGAMO, TOSHIMA-KU, TOKYO, 170-0002, JAPAN

SN 0915-5287

EI 2187-5626

J9 J PHYS THER SCI

JI J. Phys. Ther. Sci.

PD JUL

PY 2015

VL 27

IS 7

BP 2257

EP 2260

DI 10.1589/jpts.27.2257

PG 4

WC Rehabilitation

WE Science Citation Index Expanded (SCI-EXPANDED)

SC Rehabilitation

GA CN5QW

UT WOS:000358485400050

PM 26311964

OA Green Submitted, gold, Green Published

DA 2023-08-10

ER

PT J

AU Falk, B

Rigby, WA

Akseer, N

AF Falk, Bareket

Rigby, W. Alan

Akseer, Nasreen

TI Adolescent idiopathic scoliosis: the possible harm of bracing and the

likely benefit of exercise

SO SPINE JOURNAL

LA English

DT Article

ID BONE-MINERAL DENSITY; PHYSICAL-ACTIVITY; CHILDREN; IMPACT; ASSOCIATION;

OSTEOPENIA; GIRLS; MASS

C1 [Falk, Bareket; Rigby, W. Alan; Akseer, Nasreen] Brock Univ, Fac Appl Hlth Sci, Dept Kinesiol, St Catharines, ON L2S 3A1, Canada.

[Falk, Bareket] Brock Univ, Fac Appl Hlth Sci, Ctr Bone & Muscle Hlth, St Catharines, ON L2S 3A1, Canada.

C3 Brock University; Brock University

RP Falk, B (通讯作者)，Brock Univ, Fac Appl Hlth Sci, Dept Kinesiol, 500 Glenridge Ave, St Catharines, ON L2S 3A1, Canada.

EM bfalk@brocku.ca

RI Falk, Bareket/AAS-6817-2021

OI Falk, Bareket/0000-0001-7105-0185

FU Canadian Institutes of Health Research [134765]

FX BF: Grants: Canadian Institutes of Health Research (134765): Pressure

Sore Risk Management in Children with Cerebral Palsy: Local and Whole

Body Thermoregulation Strategies, Material Interface and Care Protocols

(G over 3 years, Paid directly to institution). WAR: Nothing to

disclose. NA: Nothing to disclose.

CR Behringer M, 2014, J BONE MINER RES, V29, P467, DOI 10.1002/jbmr.2036

Cheng J C, 2001, Spine (Phila Pa 1976), V26, pE19

Cheng JCY, 1999, SPINE, V24, P1218, DOI 10.1097/00007632-199906150-00008

Cheng JCY, 2000, J BONE MINER RES, V15, P1587, DOI 10.1359/jbmr.2000.15.8.1587

Climent JM, 1999, SPINE, V24, P1903, DOI 10.1097/00007632-199909150-00007

Danielsson AJ, 2006, SPINE, V31, P275, DOI 10.1097/01.brs.0000197652.52890.71

Fuchs RK, 2002, J PEDIATR-US, V141, P357, DOI 10.1067/mpd.2002.127275

Green BN, 2009, J CHIROPR MED, V8, P25, DOI 10.1016/j.jcm.2008.11.001

Gunter K, 2008, J BONE MINER RES, V23, P986, DOI 10.1359/JBMR.071201

HOUDE JP, 1995, CLIN ORTHOP RELAT R, P199

JAMES JIP, 1954, J BONE JOINT SURG BR, V36, P36, DOI 10.1302/0301-620X.36B1.36

Lam TP, 2011, SPINE, V36, P1211, DOI 10.1097/BRS.0b013e3181ebab39

Lee WTK, 2005, OSTEOPOROSIS INT, V16, P1924, DOI 10.1007/s00198-005-1964-7

Li XF, 2008, EUR SPINE J, V17, P1431, DOI 10.1007/s00586-008-0757-z

Lusini M, 2014, SPINE J, V14, P1951, DOI 10.1016/j.spinee.2013.11.040

Maruyama Toru, 2011, Physiotherapy Theory and Practice, V27, P26, DOI 10.3109/09593985.2010.503989

Mountjoy M, 2011, BRIT J SPORT MED, V45, P839, DOI 10.1136/bjsports-2011-090228

Muller C, 2011, EUR SPINE J, V20, P1127, DOI 10.1007/s00586-011-1791-9

Rahman T, 2005, J PEDIATR ORTHOPED, V25, P420, DOI 10.1097/01.bpo.0000161097.61586.bb

Roach JW, 1999, ORTHOP CLIN N AM, V30, P353, DOI 10.1016/S0030-5898(05)70092-4

ROGALA EJ, 1978, J BONE JOINT SURG AM, V60, P173, DOI 10.2106/00004623-197860020-00005

Romano M, 2013, SPINE, V38, pE883, DOI 10.1097/BRS.0b013e31829459f8

Sadat-Ali M, 2008, EUR SPINE J, V17, P944, DOI 10.1007/s00586-008-0671-4

Seifert J, 2009, ORTHOPADE, V38, P151, DOI 10.1007/s00132-008-1367-5

Snyder BD, 2005, J PEDIATR ORTHOPED, V25, P423, DOI 10.1097/01.bpo.0000158001.23177.8d

Tan VPS, 2014, J BONE MINER RES, V29, P2161, DOI 10.1002/jbmr.2254

Weinstein SL, 2013, NEW ENGL J MED, V369, P1512, DOI 10.1056/NEJMoa1307337

Weiss HR, 2013, PATIENT SAF SURG, V7, DOI 10.1186/1754-9493-7-17

NR 28

TC 3

Z9 3

U1 1

U2 17

PU ELSEVIER SCIENCE INC

PI NEW YORK

PA 360 PARK AVE SOUTH, NEW YORK, NY 10010-1710 USA

SN 1529-9430

EI 1878-1632

J9 SPINE J

JI Spine Journal

PD JUN 1

PY 2015

VL 15

IS 6

BP 1169

EP 1171

DI 10.1016/j.spinee.2014.05.006

PG 3

WC Clinical Neurology; Orthopedics

WE Science Citation Index Expanded (SCI-EXPANDED)

SC Neurosciences & Neurology; Orthopedics

GA CI6NF

UT WOS:000354875700011

PM 24846846

DA 2023-08-10

ER

PT J

AU Kim, JJ

Bin Song, G

Park, EC

AF Kim, Jwa Jun

Bin Song, Gui

Park, Eun Cho

TI Effects of Swiss ball exercise and resistance exercise on respiratory

function and trunk control ability in patients with scoliosis

SO JOURNAL OF PHYSICAL THERAPY SCIENCE

LA English

DT Article

DE Scoliosis; Respiratory function; Trunk control ability

ID ADOLESCENT IDIOPATHIC SCOLIOSIS; NONSURGICAL TREATMENT; MILWAUKEE BRACE;

PROGRAM; TRACTION

AB [Purpose] This study compared the effects of Swiss ball exercise and resistance exercise on the respiratory function and trunk control ability of patients with scoliosis. [Subjects] Forty scoliosis patients were randomly divided into the Swiss ball exercise group (n= 20) and resistance exercise

group (n = 20). [Methods] The Swiss ball and resistance exercise groups performed chest expansion and breathing exercises with a Swiss ball and a therapist's resistance, respectively. Both groups received training 30 min per day, 5 times per week for 8 weeks. [Results] Both groups exhibited significant changes in forced vital capacity, forced expiratory volume in one second, and trunk impairment scale after the intervention. However, there was no significant change in the forced expiratory volume in one second/forced vital capacity ratio after the intervention in either group. Meanwhile, forced expiratory volume in one second and trunk impairment scale were significantly greater in the resistance exercise group after the intervention. [Conclusion] Both Swiss ball exercise and resistance exercise are effective for improving the respiratory function and trunk control ability of patients with scoliosis. However, resistance exercise is more effective for increasing the forced expiratory volume in one second and trunk control ability.

C1 [Kim, Jwa Jun] Choonhae Coll Hlth Sci, Dept Phys Therapy, Ulsan, South Korea.

[Bin Song, Gui] Yeungnam Univ Coll, Dept Phys Therapy, Gyongsan, Gyeongsangbuk D, South Korea.

[Park, Eun Cho] Daegu Univ, Coll Rehabil Sci, Dept Phys Therapy, Gyongsan 712714, Kyeongbuk, South Korea.

C3 Yeungnam University; Daegu University

RP Park, EC (通讯作者)， Daegu Univ, Coll Rehabil Sci, Dept Phys Therapy, 15 Jillyang, Gyongsan 712714, Kyeongbuk, South Korea.

EM euncho76@naver.com

CR AXELGAARD J, 1983, SPINE, V8, P463, DOI 10.1097/00007632-198307000-00004

BLOUNT WP, 1958, J BONE JOINT SURG AM, V40, P511, DOI 10.2106/00004623-195840030-00003

Cailliet R, 1983, EXERCISE SCOLIOSIS

CARMAN D, 1985, J PEDIATR ORTHOPED, V5, P65, DOI 10.1097/01241398-198501000-00011

Cocker D, 1976, RESP THERAPY

Colby LA, 1990, THERAPEUTIC EXERCISE, P519

COTREL Y, 1975, J BONE JOINT SURG BR, V57, P260

Creager CC, 1994, THERAPEUTIC EXERCISE

Alves VLD, 2006, CHEST, V130, P500, DOI 10.1378/chest.130.2.500

Dyner-Jama I, 2000, Wiad Lek, V53, P603

Fabian Krzysztof Marek, 2010, Ortop Traumatol Rehabil, V12, P301

FOCARILE FA, 1991, SPINE, V16, P395, DOI 10.1097/00007632-199104000-00001

Hawes MC, 2001, CHEST, V120, P672, DOI 10.1378/chest.120.2.672

Janda V, 1998, SWISS BALL THEORY BA

KEHL DK, 1988, CLIN ORTHOP RELAT R, P34

Kim Misook, 2003, [Korean Journal of Sport Studies, 한국체육학회지], V42, P579

Lee M, 2013, J PHYS THER SCI, V25, P445, DOI 10.1589/jpts.25.445

Minehisa K, 2003, J PHYSIC THERAP SCI, V15, P105, DOI [10.1589/jpts.15.105, DOI 10.1589/JPTS.15.105]

MITCHELL RI, 1990, SPINE, V15, P514, DOI 10.1097/00007632-199006000-00016

Moon JH, 1996, ANN REHAB MED, V20, P424

NACHEMSON AL, 1995, J BONE JOINT SURG AM, V77A, P815, DOI 10.2106/00004623-199506000-00001

OLAFSSON Y, 1995, J PEDIATR ORTHOPED, V15, P524, DOI 10.1097/01241398-199507000-00023

RHIM YONG-TAEK, 2011, [Korean Society For The Study Of Physical Education, 한국체육교육학회지], V16, P159

WEISS HR, 1991, SPINE, V16, P88, DOI 10.1097/00007632-199101000-00016

Zakaria A, 2012, J PHYS THER SCI, V24, P1127, DOI 10.1589/jpts.24.1127

NR 25

TC 6

Z9 6

U1 1

U2 13

PU SOC PHYSICAL THERAPY SCIENCE

PI TOKYO

PA C/O PUBLICATION CENTER, 1-24-12 SUGAMO, TOSHIMA-KU, TOKYO, 170-0002,

JAPAN

SN 0915-5287

EI 2187-5626

J9 J PHYS THER SCI

JI J. Phys. Ther. Sci.

PD JUN

PY 2015

VL 27

IS 6

BP 1775

EP 1778

DI 10.1589/jpts.27.1775

PG 4

WC Rehabilitation

WE Science Citation Index Expanded (SCI-EXPANDED)

SC Rehabilitation

GA CM8FN

UT WOS:000357933800032

PM 26180318

OA gold, Green Published, Green Submitted

DA 2023-08-10

ER

PT J

AU Bialek, M

AF Bialek, Marianna

TI Mild Angle Early Onset Idiopathic Scoliosis Childrenavoid Progression

Under FITS Method (Functional Individual Therapy of Scoliosis)

SO MEDICINE

LA English

DT Article

ID SOSORT

C1 FITS Ctr, Jawor, Poland.

RP Bialek, M (通讯作者)，FITS Ctr, Jawor, Poland.

EM mbfits@o2.pl

CR [Anonymous], POINT MYOFASCIAL PAI

[Anonymous], U MED SCI

[Anonymous], MED MANUALNA

Aviva P, 2006, MANUAL STAT OUTLINE, V1

Bialek M., 2012, FITS METHOD PHYS THE, P26

Bialek M, 2011, SCOLIOSIS SPINAL DIS, V6, DOI 10.1186/1748-7161-6-25

Bialek M, 2009, STUD HEALTH TECHNOL, V135, P250

Chaitow L, 2010, MUSCLE ENERGYTECHNIQ, P143

Chaitow L, 2007, POSITIONAL RELEASE T, P197

Czaprowski D, 2011, SCOLIOSIS SPINAL DIS, V6, DOI 10.1186/1748-7161-6-22

DeStefano L, 2010, GREENMANS PRINCIPLES, Vfourth

Goldberg CJ, 2002, ST HEAL T, V88, P67

Lewit K., 2001, ZL NATURA, V3, P195

MANNHERZ RE, 1988, SPINE, V13, P1087, DOI 10.1097/00007632-198810000-00003

Stokes IA, 2006, SCOLIOSIS, V18, P1

NR 15

TC 6

Z9 10

U1 0

U2 4

PU LIPPINCOTT WILLIAMS & WILKINS

PI PHILADELPHIA

PA TWO COMMERCE SQ, 2001 MARKET ST, PHILADELPHIA, PA 19103 USA

SN 0025-7974

EI 1536-5964

J9 MEDICINE

JI Medicine (Baltimore)

PD MAY

PY 2015

VL 94

IS 20

AR e863

DI 10.1097/MD.0000000000000863

PG 14

WC Medicine, General & Internal

WE Science Citation Index Expanded (SCI-EXPANDED)

SC General & Internal Medicine

GA CI7ZA

UT WOS:000354984600031

PM 25997065

OA gold, Green Published

DA 2023-08-10

ER

PT J

AU Bettany-Saltikov, J

Weiss, HR

Chockalingam, N

Taranu, R

Srinivas, S

Hogg, J

Whittaker, V

Kalyan, RV

Arnell, T

AF Bettany-Saltikov, Josette

Weiss, Hans-Rudolf

Chockalingam, Nachiappan

Taranu, Razvan

Srinivas, Shreya

Hogg, Julie

Whittaker, Victoria

Kalyan, Raman V.

Arnell, Tracey

TI Surgical versus non-surgical interventions in people with adolescent idiopathic scoliosis

SO COCHRANE DATABASE OF SYSTEMATIC REVIEWS

LA English

DT Review

ID QUALITY-OF-LIFE; UPDATED METHOD GUIDELINES; LEAST 20 YEARS; SYSTEMATIC

REVIEWS; CURVE PROGRESSION; BRACE TREATMENT; BACK-PAIN; FOLLOW-UP;

DEFORMITY; VALIDITY

AB Background

Adolescent idiopathic scoliosis (AIS) is a three-dimensional deformity of the spine. While AIS can progress during growth and cause a surface deformity, it is usually not symptomatic. However, if the final spinal curvature surpasses a certain critical threshold, the risk of health problems and curve progression is increased. Interventions for the prevention of AIS progression include scoliosis-specific exercises, bracing, and surgery. The main aims of all types of interventions are to correct the deformity and prevent further deterioration of the curve and to restore trunk asymmetry and balance, while minimising morbidity and pain, allowing return to full function. Surgery is normally recommended for curvatures exceeding 40 to 50 degrees to stop curvature progression with a view to achieving better truncal balance and cosmesis. Short-term results of the surgical treatment of people with AIS demonstrate the ability of surgery to improve various outcome measures. However there is a clear paucity of information on long-term follow-up of surgical treatment of people with AIS.

Objectives

To examine the impact of surgical versus non-surgical interventions in people with AIS who have severe curves of over 45 degrees, with a focus on trunk balance, progression of scoliosis, cosmetic issues, quality of life, disability, psychological issues, back pain, and adverse effects, at both the short term (a few months) and the long term (over 20 years).

Search methods

We searched the Cochrane Back Review Group Trials Register, the Cochrane Central Register of Controlled Trials (CENTRAL), MEDLINE, EMBASE, four other databases, and three trials registers up to August 2014 with no language limitations. We also checked the reference lists of relevant articles and conducted an extensive handsearch of the grey literature.

Selection criteria

We searched for randomised controlled trials (RCTs) and prospective controlled trials comparing spinal fusion surgery with non-surgical interventions in people with AIS with a Cobb angle greater than 45 degrees. We were interested in all types of instrumented surgical interventions with fusion that aimed to provide curve correction and spine stabilisation.

Data collection and analysis

We found no RCTs or prospective controlled trials that met our inclusion criteria.

Main results

We did not identify any evidence comparing surgical to non-surgical interventions for AIS with severe curves of over 45 degrees.

Authors' conclusions

We cannot draw any conclusions.

C1 [Bettany-Saltikov, Josette; Hogg, Julie; Whittaker, Victoria] Univ Teesside, Sch Hlth & Social Care, Middlesbrough TS1 3BA, Cleveland, united kingdom.

[Weiss, Hans-Rudolf] Spinal Deform Rehabil Serv, Orthoped Practice, Gensingen, Germany.

[Chockalingam, Nachiappan] Staffordshire Univ, Fac Hlth, Stoke On Trent ST4 2DE, Staffs, united kingdom.

[Taranu, Razvan] Northumbria Healthcare NHS Fdn Trust, Dept Trauma & Orthopaed, Ashington, Tyne & Wear, united kingdom.

[Srinivas, Shreya] James Cook Univ Hosp, Northern Deanery, Middlesbrough, Cleveland, united kingdom.

[Kalyan, Raman V.] James Cook Univ Hosp, Middlesbrough, Cleveland, united kingdom.

[Arnell, Tracey] Newcastle Coll, Newcastle, Tyne & Wear, united kingdom.

C3 University of Teesside; Staffordshire University; James Cook University

Hospital; James Cook University Hospital

RP Bettany-Saltikov, J (通讯作者)， Univ Teesside, Sch Hlth & Social Care, Victoria Rd, Middlesbrough TS1 3BA, Cleveland, united kingdom.

EM j.b.saltikov@tees.ac.uk

RI Bettany-Saltikov, Josette/C-5388-2011; Chockalingam,

Nachiappan/C-4423-2014

OI Bettany-Saltikov, Josette/0000-0001-7784-500X; Chockalingam,

Nachiappan/0000-0002-7072-1271

FU University of Teesside, UK

FX Internal sources; The University of Teesside, UK.; SAR Grant

CR Andersen MO, 2006, SPINE, V31, P350, DOI 10.1097/01.brs.0000197649.29712.de

[Anonymous], 2014, COCHR COLL REV MAN R

Asher M, 2003, SPINE, V28, P63, DOI 10.1097/00007632-200301010-00015

Asher MA, 2006, SCOLIOSIS SPINAL DIS, V1, DOI 10.1186/1748-7161-1-2

Bago J, 2010, SCOLIOSIS SPINAL DIS, V5, DOI 10.1186/1748-7161-5-6

Berryman F, 2008, EUR SPINE J, V17, P663, DOI 10.1007/s00586-007-0581-x

Bettany-Saltikov, 2012, DO SYSTEMATIC REV ST

Bradford D, 1987, MOES TXB SCOLIOSIS O, V2

Bridwell KH, 1999, SPINE, V24, P2607, DOI 10.1097/00007632-199912150-00008

Bunge EM, 2007, EUR SPINE J, V16, P83, DOI 10.1007/s00586-006-0097-9

Castro Frank P Jr, 2003, Spine J, V3, P180, DOI 10.1016/S1529-9430(02)00557-0

Climent JM, 1999, SPINE, V24, P1903, DOI 10.1097/00007632-199909150-00007

Coillard C, 2003, EUR SPINE J, V12, P141, DOI 10.1007/s00586-002-0467-x

Coillard C, 2002, ST HEAL T, V88, P215

Danielsson AJ, 2001, SPINE, V26, P516

Danielsson AJ, 2001, EUR SPINE J, V10, P278, DOI 10.1007/s005860100309

Danielsson AJ, 2001, SPINE, V26, P1449, DOI 10.1097/00007632-200107010-00015

Danielsson AJ, 2003, SPINE, V28, P2078

Danielsson AJ, 2006, SPINE, V31, P275, DOI 10.1097/01.brs.0000197652.52890.71

Dolan LA, 2007, SPINE, V32, pS91, DOI 10.1097/BRS.0b013e318134ead9

Downs SH, 1998, J EPIDEMIOL COMMUN H, V52, P377, DOI 10.1136/jech.52.6.377

FALLSTROM K, 1986, SPINE, V11, P756

Freidel K, 2002, SPINE, V27, pE87, DOI 10.1097/00007632-200202150-00013

Furlan AD, 2009, SPINE, V34, P1929, DOI 10.1097/BRS.0b013e3181b1c99f

Goldberg CJ, 2002, ST HEAL T, V88, P67

Grivas T Vasiliadis, 2006, SCOLIOSIS, V1, P1

Grivas TB, 2008, DISABIL REHABIL, V30, P752, DOI 10.1080/09638280802041086

Haher T, 2003, ATLAS SPINE SURG TEC, V1

Hawes Martha, 2006, Pediatr Rehabil, V9, P318, DOI 10.1080/13638490500402264

Kadoury S, 2009, EUR SPINE J, V18, P23, DOI 10.1007/s00586-008-0817-4

Kanayama M, 1996, J BIOMECH ENG-T ASME, V118, P247, DOI 10.1115/1.2795967

Katz DE, 2001, SPINE, V26, P2354, DOI 10.1097/00007632-200111010-00012

Knott P, 2010, SCOLIOSIS S1, V5, P10, DOI [10.1186/1748-7161-5-S1-O10, DOI 10.1186/1748-7161-5-S1-O10, 10.1186/1748-7161-5-s1-o10]

Lenssinck MLB, 2005, PHYS THER, V85, P1329, DOI 10.1093/ptj/85.12.1329

Lonner Baron S, 2007, J Bone Joint Surg Am, V89 Suppl 2 Pt.1, P142, DOI 10.2106/JBJS.F.01389

Lonstein JE, 2006, CLIN ORTHOP RELAT R, P248, DOI 10.1097/01.blo.0000198725.54891.73

Lupparelli S, 2002, Stud Health Technol Inform, V91, P81

Lykissas MG, 2013, SPINE, V38, pE113, DOI 10.1097/BRS.0b013e31827ae3d0

Macdonald G, 2012, COCHRANE DB SYST REV, DOI 10.1002/14651858.CD001930.pub3

Maruyama T, 2008, SCOLIOSIS, V3, P1

Merola AA, 2002, SPINE, V27, P2046, DOI 10.1097/00007632-200209150-00015

Moramarco K, 2013, HARD TISSUE, V2, P27, DOI DOI 10.13172/2050-2303-2-3-498

Negrini S, 2007, Eura Medicophys, V43, P171

Negrini S, 2005, Eura Medicophys, V41, P183

Negrini S, 2010, COCHRANE DB SYST REV, DOI 10.1002/14651858.CD006850.pub2

Negrini S, 2012, SCOLIOSIS SPINAL DIS, V7, DOI 10.1186/1748-7161-7-3

Noonan KJ, 1997, J PEDIATR ORTHOPED, V17, P712, DOI 10.1097/00004694-199711000-00004

Odermatt D, 2003, J ORTHOP RES, V21, P931, DOI 10.1016/S0736-0266(03)00038-X

Olgun ZD, 2013, J CHILD ORTHOP, V7, P69, DOI 10.1007/s11832-012-0456-5

Oxborrow NJ, 2000, ARCH DIS CHILD, V83, P453, DOI 10.1136/adc.83.5.453

Pehrsson K, 2001, THORAX, V56, P388, DOI 10.1136/thorax.56.5.388

Pineda S, 2006, SCOLIOSIS SPINAL DIS, V1, DOI 10.1186/1748-7161-1-18

Reichel Dagmar, 2003, Pediatr Rehabil, V6, P221

Rigo M, 2006, SCOLIOSIS SPINAL DIS, V1, DOI 10.1186/1748-7161-1-11

Roaf R., 1980, SPINAL DEFORMITIES

Romano M, 2012, COCHRANE DB SYST REV, DOI 10.1002/14651858.CD007837.pub2

Rowe DE, 1997, J BONE JOINT SURG AM, V79A, P664, DOI 10.2106/00004623-199705000-00005

Sanders JO, 2007, SPINE, V32, P2719, DOI 10.1097/BRS.0b013e31815a5959

SHEKELLE PG, 1994, SPINE, V19, pS2028

Smania N, 2008, DISABIL REHABIL, V30, P763, DOI 10.1080/17483100801921311

Stokes IAF, 1996, SPINE, V21, P1162, DOI 10.1097/00007632-199605150-00007

Stokes IAF, 2006, SCOLIOSIS SPINAL DIS, V1, DOI 10.1186/1748-7161-1-16

Sutton AJ, 2000, BMJ-BRIT MED J, V320, P1574, DOI 10.1136/bmj.320.7249.1574

van Tulder M, 2003, SPINE, V28, P1290, DOI 10.1097/00007632-200306150-00014

Weiss H, 2004, SPINE J, V4, P485

Weiss HR, 2008, EUR J PHYS REHAB MED, V44, P177

Weiss HR, 2008, DISABIL REHABIL, V30, P799, DOI 10.1080/09638280801889717

Weiss HR, 2006, SCOLIOSIS SPINAL DIS, V1, DOI [10.1186/1748-7161-1-5, 10.1186/1748-7161-1-1]

Weiss HR, 2006, ST HEAL T, V123, P347

Weiss HR, 2008, SCOLIOSIS SPINAL DIS, V3, DOI 10.1186/1748-7161-3-9

Westrick ER, 2011, J PEDIATR ORTHOPED, V31, pS61, DOI 10.1097/BPO.0b013e3181fd87d5

White A, 1990, CLIN BIOMECHANICS SP

NR 72

TC 37

Z9 40

U1 2

U2 29

PU WILEY

PI HOBOKEN

PA 111 RIVER ST, HOBOKEN 07030-5774, NJ USA

SN 1469-493X

EI 1361-6137

J9 COCHRANE DB SYST REV

JI Cochrane Database Syst Rev.

PY 2015

IS 4

AR CD010663

DI 10.1002/14651858.CD010663.pub2

PG 32

WC Medicine, General & Internal

WE Science Citation Index Expanded (SCI-EXPANDED); Social Science Citation Index (SSCI)

SC General & Internal Medicine

GA CK1UQ

UT WOS:000355993500048

PM 25908428

OA Bronze, Green Accepted, Green Published

DA 2023-08-10

ER

PT J

AU Plaszewski, M

Kotwicki, T

Chwala, W

Terech, J

Cieslinski, I

AF Plaszewski, Maciej

Kotwicki, Tomasz

Chwala, Wieslaw

Terech, Jacek

Cieslinski, Igor

TI Study protocol and overview of the literature on long-term health and

quality of life outcomes in patients treated in adolescence for

scoliosis with therapeutic exercises

SO JOURNAL OF BACK AND MUSCULOSKELETAL REHABILITATION

LA English

DT Article

DE Scoliosis; exercises; long-term outcomes; quality of life; functioning;

mental health

ID LEAST 20 YEARS; IDIOPATHIC SCOLIOSIS; FOLLOW-UP; BACK-PAIN;

SURGICAL-TREATMENT; CURVE PROGRESSION; BRACE TREATMENT; COHORT; FUSION;

QUESTIONNAIRE

AB BACKGROUND: Scoliosis, the most prevalent orthopaedic condition affecting children and adolescents, may have lasting physical, psychological and social consequences. With limited evidence-base, scoliosis-specific exercise therapies are an option.

OBJECTIVE: An overview of the subject and description of a long-term follow-up study including adults who in adolescence were treated with a scoliosis-specific exercise programme investigating the association of the exercise regime with present physical activity, physical functioning and subjective wellbeing. To the authors' best knowledge, this is the first long-term outcome study on scoliosis-specific exercises, in opposition to a number of studies in adults who were braced or treated surgically in adolescence.

METHODS: Observational, registry-based case-control study. Adult subjects who in adolescence were treated with an exercise programme or were under observation are invited. Spine and trunk deformity, respiratory function, physical capacity and trunk muscles' function are measured. Health-

related quality of life with generic and condition-specific instruments, general mental health, depression and anxiety symptoms, disability due to low back problems and physical activity are assessed.

CONCLUSIONS: The report is believed to provide the readers with an overview of this controversial aspect of rehabilitation, and that the proposed protocol will assist researchers designing their studies.

C1 [Plaszewski, Maciej; Cieslinski, Igor] Warsaw Univ, Sch Phys Educ, Inst Physiotherapy, Fac Phys Educ, PL-21500 Biala Podlaska, Poland.

[Kotwicki, Tomasz] Univ Med Sci, Poznan, Poland.

[Chwala, Wieslaw] Cracow Univ, Sch Phys Educ, Sect Biomech, Krakow, Poland.

[Terech, Jacek] Ctr Pulmonol & Thorac Surg, Bystra Slaska, Poland.

C3 4EU+; University of Warsaw; Jozef Pilsudski University Physical

Education in Warsaw; 1EUROPE; Jagiellonian University

RP Plaszewski, M (通讯作者)， Warsaw Univ, Fac Phys Educ & Sport, Inst Physiotherapy, Sch Phys Educ,Akad 2, PL-21500 Biala Podlaska, Poland.

EM maciej.plaszewski@awf-bp.edu.pl

OI Chwala, Wieslaw/0000-0002-2906-6455; Cieslinski,

Igor/0000-0001-8672-9334

FU University School of Physical Education, Warsaw, Poland [DS.136];

Ministry of Science and Higher Education, Poland

FX The authors gratefully acknowledge the generous support from Doctor

Bartosz Molik, and they wish to thank the participants, who offer their

time and energy to contribute for this project. Funding for this study

was provided by the University School of Physical Education, Warsaw,

Poland (research project DS.136), and sponsored by the Ministry of

Science and Higher Education, Poland.

CR [Anonymous], AD ID SCOL

Aprile I, 2007, EUR SPINE J, V16, P1962, DOI 10.1007/s00586-007-0461-4

Asher M, 2003, SPINE, V28, P63, DOI 10.1097/00007632-200301010-00015

Asher MA, 2006, SCOLIOSIS SPINAL DIS, V1, DOI 10.1186/1748-7161-1-2

Carter RE, 2011, REHABILITATION RES, p[94, 402]

Chwala W, 2012, STUD HEALTH TECHNOL, V176, P129, DOI 10.3233/978-1-61499-067-3-129

Czaprowski D, 2012, EUR SPINE J, V21, P1099, DOI 10.1007/s00586-011-2068-z

Danielsson AJ, 2007, SPINE, V32, P2198, DOI 10.1097/BRS.0b013e31814b851f

Danielsson AJ, 2012, SPINE, V37, P755, DOI 10.1097/BRS.0b013e318231493c

Danielsson AJ, 2010, SPINE, V35, P199, DOI 10.1097/BRS.0b013e3181c89f4a

Danielsson AJ, 2001, SPINE, V26, P516

Danielsson AJ, 2003, SPINE, V28, pE373, DOI 10.1097/01.BRS.0000084267.41183.75

Danielsson AJ, 2001, EUR SPINE J, V10, P278, DOI 10.1007/s005860100309

Danielsson AJ, 2001, SPINE, V26, P1449, DOI 10.1097/00007632-200107010-00015

Danielsson AJ, 2003, SPINE, V28, P2078

Danielsson AJ, 2006, SPINE, V31, P275, DOI 10.1097/01.brs.0000197652.52890.71

Danielsson AJ, 2001, ACTA RADIOL, V42, P187, DOI 10.1034/j.1600-0455.2001.042002187.x

Davidson M, 2005, AUST J PHYSIOTHER, V51, P270, DOI 10.1016/S0004-9514(05)70016-7

Edwards P, 2002, BRIT MED J, V324, P1183, DOI 10.1136/bmj.324.7347.1183

Fairbank JCT, 2000, SPINE, V25, P2940, DOI 10.1097/00007632-200011150-00017

Glowacki M, 2009, SPINE, V34, P1060, DOI 10.1097/BRS.0b013e31819c1ec3

Goldberg D, 2001, ASSESSMENT MENTAL HL

GOLDBERG MS, 1994, SPINE, V19, P1562, DOI 10.1097/00007632-199407001-00004

GOLDBERG MS, 1994, SPINE, V19, P1551, DOI 10.1097/00007632-199407001-00003

Groth-Marnat G, 2009, HDB PSYCHOL ASSESSME, P526

Hawes Martha C, 2003, Pediatr Rehabil, V6, P171

Koumbourlis AC, 2006, PAEDIATR RESPIR REV, V7, P152, DOI 10.1016/j.prrv.2006.04.009

Landgraf JM., 2007, ASSESSING QUALITY LI, P346

LONSTEIN JE, 1994, LANCET, V344, P1407

MAYO NE, 1994, SPINE, V19, P1573, DOI 10.1097/00007632-199407001-00005

Misterska E, 2011, SPINE, V36, pE1722, DOI 10.1097/BRS.0b013e318216ad48

Muldoon MF, 1998, BMJ-BRIT MED J, V316, P542, DOI 10.1136/bmj.316.7130.542

NACHEMSON AL, 1995, J BONE JOINT SURG AM, V77A, P815, DOI 10.2106/00004623-199506000-00001

Negrini S, 2010, COCHRANE DB SYST REV, V1

Negrini S, 2012, SCOLIOSIS SPINAL DIS, V7, DOI 10.1186/1748-7161-7-3

PATRICK DL, 1993, HLTH STATUS HLTH POL, P22

Pehrsson K, 2001, THORAX, V56, P388, DOI 10.1136/thorax.56.5.388

Plaszewski M, 2012, SCOLIOSIS S1, V7

Plaszewski M, 2012, SCOLIOSIS S1, V7, pO9

POITRAS B, 1994, SPINE, V19, P1582, DOI 10.1097/00007632-199407001-00006

Puzynski S, 2002, PSYCHIATRIA, P453

Reamy BV, 2001, AM FAM PHYSICIAN, V64, P111

Rigo MD, 2010, SCOLIOSIS SPINAL DIS, V5, DOI 10.1186/1748-7161-5-27

Robinson JP, 1991, MEASURES PERSONALITY, P201

Romano M, 2012, COCHRANE DB SYST REV, DOI 10.1002/14651858.CD007837.pub2

Rowe DE, 1997, J BONE JOINT SURG AM, V79A, P664, DOI 10.2106/00004623-199705000-00005

Skaggs DL, 1996, AM FAM PHYSICIAN, V53, P2327

Spielberger C. D., 1983, MANUAL STATE TRAIT A

Tones M, 2006, SPINE, V31, P3027, DOI 10.1097/01.brs.0000249555.87601.fc

WEINSTEIN SL, 1981, J BONE JOINT SURG AM, V63, P702, DOI 10.2106/00004623-198163050-00003

Weinstein SL, 2003, JAMA-J AM MED ASSOC, V289, P559, DOI 10.1001/jama.289.5.559

Weinstein SL, 2008, LANCET, V371, P1527, DOI 10.1016/S0140-6736(08)60658-3

Weiss HR, 2008, EUR J PHYS REHAB MED, V44, P177

WHO, 1993, MNH7PSF939 WHO

Wong MS, 2003, PROSTHET ORTHOT INT, V27, P242, DOI 10.1080/03093640308726688

Wrzesniewski K, 2006, POLISH ADAPTATION ST

NR 56

TC 3

Z9 7

U1 2

U2 26

PU IOS PRESS

PI AMSTERDAM

PA NIEUWE HEMWEG 6B, 1013 BG AMSTERDAM, NETHERLANDS

SN 1053-8127

EI 1878-6324

J9 J BACK MUSCULOSKELET

JI J. Back Musculoskelet. Rehabil.

PY 2015

VL 28

IS 3

BP 453

EP 462

DI 10.3233/BMR-140540

PG 10

WC Orthopedics; Rehabilitation

WE Science Citation Index Expanded (SCI-EXPANDED)

SC Orthopedics; Rehabilitation

GA CL2XI

UT WOS:000356810600005

PM 25322738

DA 2023-08-10

ER

PT J

AU Jones, ML

Evans, N

Tefertiller, C

Backus, D

Sweatman, M

Tansey, K

Morrison, S

AF Jones, Michael. L.

Evans, Nicholas

Tefertiller, Candace

Backus, Deborah

Sweatman, Mark

Tansey, Keith

Morrison, Sarah

TI Activity-Based Therapy for Recovery of Walking in Chronic Spinal Cord Injury: Results From a Secondary Analysis to Determine Responsiveness to Therapy

SO ARCHIVES OF PHYSICAL MEDICINE AND REHABILITATION

LA English

DT Article

DE Exercise therapy; Gait disorders; neurologic; Motor activity;

Rehabilitation; Spinal cord injuries

ID REHABILITATION; SPEED; MOTOR

AB Objective: To gain insight into who is likely to benefit from activity-based therapy (ABT), as assessed by secondary analysis of data obtained from a clinical trial.

Design: Secondary analysis of results from a randomized controlled trial with delayed treatment design.

Setting: Outpatient program in a private, nonprofit rehabilitation hospital.

Participants: Volunteer sample of adults (N=38; 27 men; 11 women; age, 22-63y) with chronic (>12mo postinjury), motor-incomplete (American Spinal Injury Association [ASIA] Impairment Scale [AIS] grade C or D) spinal cord injury (SCI).

Interventions: A total of 9h/wk of ABT for 24 weeks including developmental sequencing; resistance training; repetitive, patterned motor activity; and task-specific locomotor training. Algorithms were used to guide group allocation, functional electrical stimulation utilization, and locomotor training progression.

Main Outcome Measures: Walking speed and endurance (10-meter walk test and 6-minute walk test) and functional ambulation (timed Up and Go test).

Results: This secondary analysis identified likely responders to ABT on the basis of injury characteristics: AIS classification, time since injury, and initial walking ability. Training effects were the most clinically significant in AIS grade D participants with injuries <3 years in duration. This information, along with information about preliminary responsiveness to therapy (gains after 12wk), can help predict the degree of recovery likely from participation in an ABT program.

Conclusions: ABT has the potential to promote neurologic recovery and enhance walking ability in individuals with chronic, motor-incomplete SCI. However, not everyone with goals of walking recovery will benefit. Individuals with SCI should be advised of the time, effort, and resources required to undertake ABT. Practitioners are encouraged to use the findings from this trial to assist prospective participants in establishing realistic expectations for recovery. (C) 2014 by the American Congress of Rehabilitation Medicine

C1 [Jones, Michael. L.; Evans, Nicholas; Tefertiller, Candace; Backus, Deborah; Sweatman, Mark; Tansey, Keith; Morrison, Sarah] Shepherd Ctr, Virginia C Crawford Res Inst, Atlanta, GA 30309 USA.

RP Jones, ML (通讯作者)，Shepherd Ctr, 2020 Peachtree Rd NW, Atlanta, GA 30309 USA.

EM mike_jones@shepherd.org

RI Evans, Nicholas/AAT-1330-2020

FU National Institute on Disability and Rehabilitation Research (NIDRR), U.S. Department of Education [H1330080031-10]

FX Supported in part by the National Institute on Disability and Rehabilitation Research (NIDRR), U.S. Department of Education (grant no. H1330080031-10). The opinions contained in this article are those of the authors and do not necessarily reflect those of the US. Department of Education or the NIDRR

CR American Spinal Injury Association, 2011, INT STAND NEUR CLASS

Behrman AL, 2006, PHYS THER, V86, P1406, DOI 10.2522/ptj.20050212

Benz EN, 2005, ARCH PHYS MED REHAB, V86, P52, DOI 10.1016/j.apmr.2004.01.033

Buehner JJ, 2012, ARCH PHYS MED REHAB, V93, P1530, DOI 10.1016/j.apmr.2012.02.035

Field-Fote Edelle C, 2005, J Neurol Phys Ther, V29, P127

Fritz S, 2009, J GERIATR PHYS THER, V32, P2, DOI 10.1519/00139143-200932020-00002

Harkema SJ, 2012, ARCH PHYS MED REHAB, V93, P1508, DOI 10.1016/j.apmr.2011.01.024

Itzkovich M, 2007, DISABIL REHABIL, V29, P1926, DOI 10.1080/09638280601046302

Jones ML, 2014, ARCH PHYS MED REHABI, DOI 10.1016/j.apmr.2014.07.401

Kleim JA, 2008, J SPEECH LANG HEAR R, V51, pS225, DOI 10.1044/1092-4388(2008/018)

Lam T, 2008, SPINAL CORD, V46, P246, DOI 10.1038/sj.sc.3102134

Lorenz DJ, 2012, ARCH PHYS MED REHAB, V93, P1541, DOI 10.1016/j.apmr.2012.01.027

Musselman KE, 2007, PHYS THER REV, V12, P287, DOI 10.1179/108331907X223128

WATERS RL, 1994, ARCH PHYS MED REHAB, V75, P306, DOI 10.1016/0003-9993(94)90034-5

Winchester P, 2009, J SPINAL CORD MED, V32, P63, DOI 10.1080/10790268.2009.11760754

Wirz M, 2001, J NEUROL NEUROSUR PS, V71, P93, DOI 10.1136/jnnp.71.1.93

NR 16

TC 23

Z9 24

U1 1

U2 20

PU W B SAUNDERS CO-ELSEVIER INC

PI PHILADELPHIA

PA 1600 JOHN F KENNEDY BOULEVARD, STE 1800, PHILADELPHIA, PA 19103-2899 USA

SN 0003-9993

EI 1532-821X

J9 ARCH PHYS MED REHAB

JI Arch. Phys. Med. Rehabil.

PD DEC

PY 2014

VL 95

IS 12

BP 2247

EP 2252

DI 10.1016/j.apmr.2014.07.401

PG 6

WC Rehabilitation; Sport Sciences

WE Science Citation Index Expanded (SCI-EXPANDED)

SC Rehabilitation; Sport Sciences

GA AW0AY

UT WOS:000345954000003

PM 25102385

DA 2023-08-10

ER

PT J

AU Mohammadi, P

Akbari, M

Sarrafzadeh, J

Moradi, Z

AF Mohammadi, Pirayeh

Akbari, Mohammad

Sarrafzadeh, Javad

Moradi, Zahra

TI Comparison of respiratory muscles activity and exercise capacity in

patients with idiopathic scoliosis and healthy individuals

SO PHYSIOTHERAPY THEORY AND PRACTICE

LA English

DT Article

DE Electromyography; idiopathic scoliosis; respiratory muscles

ID PULMONARY-FUNCTION; ADOLESCENTS; MILD; VENTILATION; TOLERANCE; DISEASE

AB Purpose: Idiopathic scoliosis causes respiratory muscles weakness and reduced exercise capacity. However, the mechanism of these symptoms is still unknown. The main objective of this study was to determine the intensity of respiratory muscle activity and exercise capacity in patients with idiopathic scoliosis in comparison with healthy people. Subjects: In this study, 20 female patients with adult idiopathic scoliosis (10 mild and 10 moderate) as well as 10 healthy matched individuals with characteristics of the patients were selected. Methods: The subjects were fatigued through a maximal incremental cycle ergometry protocol. Meanwhile, the electromyography values of the external intercostal muscles and diaphragm were recorded bilaterally, and fatigue duration was determined. Results: The root mean square of concave external intercostal muscles and concave

diaphragm in patients with idiopathic scoliosis was significantly reduced during the fatiguing exercise protocol compared with healthy individuals. The median frequencies of the two sides differed significantly and were lower in patients with moderate scoliosis than healthy subjects. Fatigue duration (minutes) also was lower in patients with moderate scoliosis than healthy subjects. Conclusions: Scoliosis causes respiratory muscle weakness and reduced fatigue duration in response to mild physical activity compared with healthy subjects and these dysfunctions appear to be related to the severity of scoliosis curvature (moderate > mild).

C1 [Mohammadi, Pirayeh; Akbari, Mohammad; Sarrafzadeh, Javad; Moradi, Zahra] Iran Univ Med Sci, Dept Phys Therapy, Tehran 1545913187, Iran.

C3 Iran University of Medical Sciences

RP Akbari, M (通讯作者), Iran Univ Med Sci, Dept Phys Therapy, Shahnazari St,Madar Sq,Mirdamad Blv, Tehran 1545913187, Iran.

EM akbari.mo@iums.ac.ir

RI sarrafzadeh, javad/M-8489-2018

OI sarrafzadeh, javad/0000-0002-0686-2854

FU Iran University of Medical Sciences [92.d.130.769]

FX The study was funded and supported by Iran University of Medical Sciences (Grant No: 92.d.130.769). The authors report no conflict of interest.

CR Barrios C, 2005, SPINE, V30, P1610, DOI 10.1097/01.brs.0000169447.55556.01

Bianchi L, 1998, EUR RESPIR J, V11, P422, DOI 10.1183/09031936.98.11020422

Borel JC, 2009, RESP PHYSIOL NEUROBI, V167, P168, DOI 10.1016/j.resp.2009.03.014

Budweiser S, 2006, ARCH PHYS MED REHAB, V87, P1559, DOI 10.1016/j.apmr.2006.08.340

Chien MY, 2008, SENSORS-BASEL, V8, P2174, DOI 10.3390/s8042174

Cobb JR., 1948, INSTR COURSE LECT, V5, P261

Alves VLD, 2006, CHEST, V130, P500, DOI 10.1378/chest.130.2.500

Alves VLD, 2009, SPINE, V34, pE926, DOI 10.1097/BRS.0b013e3181afd1b2

Duiverman ML, 2009, RESP PHYSIOL NEUROBI, V167, P195, DOI 10.1016/j.resp.2009.04.018

Durmala J, 2009, STUD HEALTH TECHNOL, V135, P237

Harrison RA, 2007, J BONE MINER RES, V22, P447, DOI 10.1359/jbmr.061202

Hebela NM, 2009, SEMINARS SPINE SURG, V21, P16, DOI DOI 10.1053/J.SEMSS.2008.11.003

Koumbourlis AC, 2006, PAEDIATR RESPIR REV, V7, P152, DOI 10.1016/j.prrv.2006.04.009

LEECH JA, 1985, J PEDIATR-US, V106, P143, DOI 10.1016/S0022-3476(85)80487-X

Martinez-Llorens J, 2010, EUR RESPIR J, V36, P393, DOI 10.1183/09031936.00025509

Nnadi C., 2010, PAED CHILD HEALT-CAN, V20, P215, DOI [DOI 10.1016/J.PAED.2009.11.009, 10.1016/j.paed.2009.11.009]

SMYTH RJ, 1984, THORAX, V39, P901, DOI 10.1136/thx.39.12.901

Verges S, 2009, RESP PHYSIOL NEUROBI, V169, P282, DOI 10.1016/j.resp.2009.09.005

NR 18

TC 7

Z9 8

U1 1

U2 22

PU TAYLOR & FRANCIS INC

PI PHILADELPHIA

PA 530 WALNUT STREET, STE 850, PHILADELPHIA, PA 19106 USA

SN 0959-3985

EI 1532-5040

J9 PHYSIOTHER THEOR PR

JI Physiother. Theory Pract.

PD NOV

PY 2014

VL 30

IS 8

BP 552

EP 556

DI 10.3109/09593985.2014.938382

PG 5

WC Rehabilitation

WE Science Citation Index Expanded (SCI-EXPANDED)

SC Rehabilitation

GA AR6WS

UT WOS:000343722200005

PM 25051355

DA 2023-08-10

ER

PT J

AU Plaszewski, M

Bettany-Saltikov, J

AF Plaszewski, Maciej

Bettany-Saltikov, Josette

TI Non-Surgical Interventions for Adolescents with Idiopathic Scoliosis: An

Overview of Systematic Reviews

SO PLOS ONE

LA English

DT Article

ID EXERCISES REDUCE; SPINAL DEFORMITY; MEASUREMENT TOOL; EFFICACY; QUALITY

AB Background: Non-surgical interventions for adolescents with idiopathic scoliosis remain highly controversial. Despite the publication of numerous reviews no explicit methodological evaluation of

papers labeled as, or having a layout of, a systematic review, addressing this subject matter, is available.

Objectives: Analysis and comparison of the content, methodology, and evidence-base from systematic reviews regarding non-surgical interventions for adolescents with idiopathic scoliosis.

Design: Systematic overview of systematic reviews. Methods: Articles meeting the minimal criteria for a systematic review, regarding any non-surgical intervention for adolescent idiopathic scoliosis, with any outcomes measured, were included. Multiple general and systematic review specific databases, guideline registries, reference lists and websites of institutions were searched. The AMSTAR tool was used to critically appraise the methodology, and the Oxford Centre for Evidence Based Medicine and the Joanna Briggs Institute's hierarchies were applied to analyze the levels of evidence from included reviews.

Results: From 469 citations, twenty one papers were included for analysis. Five reviews assessed the effectiveness of scoliosis-specific exercise treatments, four assessed manual therapies, five evaluated bracing, four assessed different combinations of interventions, and one evaluated usual physical activity. Two reviews addressed the adverse effects of bracing. Two papers were high quality Cochrane reviews, Three were of moderate, and the remaining sixteen were of low or very low methodological quality. The level of evidence of these reviews ranged from 1 or 1+ to 4, and in some reviews, due to their low methodological quality and/or poor reporting, this could not be established.

Conclusions: Higher quality reviews indicate that generally there is insufficient evidence to make a judgment on whether non-surgical interventions in adolescent idiopathic scoliosis are effective. Papers labeled as systematic reviews need to be considered in terms of their methodological rigor; otherwise they may be mistakenly regarded as high quality sources of evidence.

C1 [Plaszewski, Maciej] Univ Sch Phys Educ, Fac Phys Educ & Sport Biala Podlaska, Warsaw, Biala Podlaska, Poland.

[Bettany-Saltikov, Josette] Univ Teesside, Sch Hlth & Social Care, Middlesbrough, Cleveland, united kingdom.

C3 Jozef Pilsudski University Physical Education in Warsaw; University of

Teesside

RP Plaszewski, M (通讯作者)， Univ Sch Phys Educ, Fac Phys Educ & Sport Biala Podlaska, Ul Akad 2, Warsaw, Biala Podlaska, Poland.

EM plaszewski@wp.pl

RI Bettany-Saltikov, Josette/C-5388-2011

OI Bettany-Saltikov, Josette/0000-0001-7784-500X

FU Faculty of Physical Education and Sport in Biala Podlaska, Warsaw

University School of Physical Education, Poland

FX Publication of this manuscript was supported by the Faculty of Physical Education and Sport in Biala Podlaska, Warsaw University School of Physical Education, Poland. The funders had no role in study design, data collection and analysis, decision to publish, or preparation of the manuscript. The authors received no other financial support for conducting this study.

CR Altaf F, 2013, BMJ-BRIT MED J, V346, DOI 10.1136/bmj.f2508

[Anonymous], 2010, HS, V122, p[1, 4]

[Anonymous], 2008, DISABIL REHABIL, V30, P731

Bettany-Saltikov J, 2014, EUR J PHYS REHAB MED, V50, P111

CASSELLA MC, 1991, PHYS THER, V71, P897, DOI 10.1093/ptj/71.12.897

Centre for Reviews and Dissemination, 2008, SYST REV CRDS GUID U

Davies Evan, 2011, Evid Based Spine Care J, V2, P25, DOI 10.1055/s-0030-1267102

Dickson RA, 1999, SPINE, V24, P2601, DOI 10.1097/00007632-199912150-00007

Dolan LA, 2007, SPINE, V32, pS91, DOI 10.1097/BRS.0b013e318134ead9

Falk B, 2014, SPINE J EPUB AHEAD P, DOI [10.1016/j.spinee.2014.05.00, DOI 10.1016/J.SPINEE.2014.05.00]

FOCARILE FA, 1991, SPINE, V16, P395, DOI 10.1097/00007632-199104000-00001

Fred M, 2011, CURR REV MUSCULOSKE, V4, P175, DOI 10.1007/s12178-011-9100-0

Fusco C., 2011, Physiotherapy Theory and Practice, V27, P80, DOI 10.3109/09593985.2010.533342

Gagnier Joel J, 2013, J Bone Joint Surg Am, V95, pe771, DOI 10.2106/JBJS.L.00597

Gianola S, 2013, PHYS THER, V93, P1456, DOI 10.2522/ptj.20120382

Gleberzon Brian J, 2012, J Can Chiropr Assoc, V56, P128

Green BN, 2009, J CHIROPR MED, V8, P25, DOI 10.1016/j.jcm.2008.11.001

Hartling L, 2012, PLOS ONE, V7, DOI 10.1371/journal.pone.0049667

Hasson S, 2011, PHYSIOTHER THEOR PRA, V271

Hawes Martha C, 2003, Pediatr Rehabil, V6, P171

Horne JP, 2014, AM FAM PHYSICIAN, V89, P193

Howick J, 2011, EXPLANATION 20111 OX

Hresko MT, 2013, NEW ENGL J MED, V368, P834, DOI 10.1056/NEJMcp1209063

Karolinska Institutet, 2012, TRIAL 3 TREAT SCOL C

Kepler CK, 2012, SPINE J, V12, P676, DOI 10.1016/j.spinee.2012.05.011

Lenssinck MLB, 2005, PHYS THER, V85, P1329, DOI 10.1093/ptj/85.12.1329

Li XF, 2008, EUR SPINE J, V17, P1431, DOI 10.1007/s00586-008-0757-z

Liberati A, 2009, BMJ-BRIT MED J, V339, DOI [10.1136/bmj.b2700, 10.1136/bmj.b4037, 10.1371/journal.pmed.1000097, 10.7326/0003-4819-151-4-200908180-00136]

LONSTEIN JE, 1994, LANCET, V344, P1407

Maruyama Toru, 2011, Physiotherapy Theory and Practice, V27, P26, DOI 10.3109/09593985.2010.503989

McKenney K, 2013, J ATHL TRAINING, V48, P522, DOI 10.4085/1062-6050-48.3.17

Moher D, 2015, SYST REV-LONDON, V4, DOI [10.1136/bmj.b2535, 10.1186/s13643-015-0087-2, 10.1016/j.ijsu.2010.02.007, 10.1016/j.ijsu.2010.07.299]

Monticone M, 2014, EUR SPINE J, V23, P1204, DOI 10.1007/s00586-014-3241-y

Mordecai SC, 2012, EUR SPINE J, V21, P382, DOI 10.1007/s00586-011-2063-4

National Institute for Health Research Health Technology Assessment Programme, 2012, ACT TREAT ID AD SCO

Negrini S, 2008, DISABIL REHABIL, V30, P772, DOI 10.1080/09638280801889568

Negrini S, 2010, COCHRANE DB SYST REV, DOI 10.1002/14651858.CD006850.pub2

Negrini Stefano, 2003, Pediatr Rehabil, V6, P227, DOI: 10.1080/13638490310001636781

Negrini S, 2012, SCOLIOSIS SPINAL DIS, V7, DOI 10.1186/1748-7161-7-3

OCEBM Levels of Evidence Working Group, 2011, OXFORD 2011 LEVELS E

Plaszewski M, 2014, EUR SPINE J, V23, P2572, DOI 10.1007/s00586-014-3307-x

Popovich I, 2012, PLOS ONE, V7, DOI 10.1371/journal.pone.0050403

Posadzki P, 2013, PEDIATRICS, V132, P140, DOI 10.1542/peds.2012-3959

Posadzki P, 2011, NEW ZEAL MED J, V124, P55

Reamy BV, 2001, AM FAM PHYSICIAN, V64, P111

Rihn JA, 2009, AM J MED QUAL, V24, p4S, DOI 10.1177/1062860609349214

Romano M, 2008, SCOLIOSIS, V3, P2

Romano M, 2012, COCHRANE DB SYST REV, DOI 10.1002/14651858.CD007837.pub2

Rowe DE, 1997, J BONE JOINT SURG AM, V79A, P664, DOI 10.2106/00004623-199705000-00005

Saccucci M, 2011, SCOLIOSIS SPINAL DIS, V6, DOI 10.1186/1748-7161-6-15

Sanders JO, 2014, J BONE JOINT SURG AM, V96A, P649, DOI 10.2106/JBJS.M.00290

Sanders James O, 2012, J Pediatr Orthop, V32 Suppl 2, pS153, DOI 10.1097/BPO.0b013e31825199e5

Schiller JR, 2010, CLIN ORTHOP RELAT R, V468, P670, DOI 10.1007/s11999-009-0884-9

Scoliosis Research Society, 2014, AD ID SCO TREAT

Shea BJ, 2007, BMC MED RES METHODOL, V7, DOI 10.1186/1471-2288-7-10

Shea BJ, 2007, PLOS ONE, V2, DOI 10.1371/journal.pone.0001350

Skaggs DL, 1996, AM FAM PHYSICIAN, V53, P2327

Sponseller PD, 2011, J PEDIATR ORTHOPED, V31, pS53, DOI 10.1097/BPO.0b013e3181f73e87

Stokes OM, 2013, BONE JOINT J, V95B, P1308, DOI 10.1302/0301-620X.95B10.31474

Stroup DF, 2000, JAMA-J AM MED ASSOC, V283, P2008, DOI 10.1001/jama.283.15.2008

The Joanna Briggs Institute Levels of Evidence and Grades of Recommendation Working Party, 2014, SUPP DOC JOANN BRIGG

The Joanna Briggs Institute Levels of Evidence and Grades of Recommendation Working Party, 2014, NEW JBI LEV EV

University of Alberta, 2012, SCHROTH EX TRIAL SCO

US Preventive Services Task Force, 2004, AHRQ PUB, V05-0568-B

Weinstein SL, 2008, LANCET, V371, P1527, DOI 10.1016/S0140-6736(08)60658-3

Weinstein SL, 2013, NEW ENGL J MED, V369, P1512, DOI 10.1056/NEJMoa1307337

Weiss HR, 2008, EUR J PHYS REHAB MED, V44, P177

Weiss Hans-Rudolf, 2003, Pediatr Rehabil, V6, P183

Weiss HR, 2012, POL ANN MED, V19, P72

Weiss HR, 2006, SCOLIOSIS, V11, P1

Zaina F, 2014, EUR J PHYS REHAB MED, V50, P93

Zarzycka Maja, 2009, Ortop Traumatol Rehabil, V11, P396

NR 72

TC 10

Z9 11

U1 2

U2 37

PU PUBLIC LIBRARY SCIENCE

PI SAN FRANCISCO

PA 1160 BATTERY STREET, STE 100, SAN FRANCISCO, CA 94111 USA

SN 1932-6203

J9 PLOS ONE

JI PLoS One

PD OCT 29

PY 2014

VL 9

IS 10

AR e110254

DI 10.1371/journal.pone.0110254

PG 19

WC Multidisciplinary Sciences

WE Science Citation Index Expanded (SCI-EXPANDED)

SC Science & Technology - Other Topics

GA AT8TG

UT WOS:000345204200019

PM 25353954

OA Green Published, gold, Green Submitted

DA 2023-08-10

ER

PT J

AU Sperandio, EF

Alexandre, AS

Yi, LC

Poletto, PR

Gotfryd, AO

Vidotto, MC

Dourado, VZ

AF Sperandio, Evandro F.

Alexandre, Anderson S.

Yi, Liu C.

Poletto, Patricia R.

Gotfryd, Alberto O.

Vidotto, Milena C.

Dourado, Victor Z.

TI Functional aerobic exercise capacity limitation in adolescent idiopathic

scoliosis

SO SPINE JOURNAL

LA English

DT Article

DE Exercise limitation; Lung function; Adolescent idiopathic scoliosis;

Spine; Respiratory muscles; Cardiovascular deconditioning

ID SHUTTLE WALKING TEST; PULMONARY-FUNCTION; REFERENCE VALUES; ADULTS

AB BACKGROUND CONTEXT: Exercise limitation has been described in patients with adolescent idiopathic scoliosis (AIS); however, whether the walking performance is impaired in these patients should be elucidated.

PURPOSE: Thus, we aimed to evaluate the physiologic responses to the incremental shuttle walk test (ISWT) in patients with AIS.

STUDY DESIGN/SETTING: Cross-sectional study.

PATIENT SAMPLE: Twenty-nine patients with AIS and 20 healthy adolescents aged between 11 and 18 years old.

OUTCOME MEASURES: Oxygen uptake (VO2), incremental shuttle walk distance (ISWD), Delta VO2/Delta walking velocity, Delta HR/Delta VO2, Delta VE/Delta VCO2, and linearized Delta tidal volume (VT)/Delta lnVE, forced expiratory volume in the first second of expiration (FEV1), and forced vital capacity (FVC).

METHODS: We performed two ISWTs, and the data used were acquired in the second test. We also evaluated the lung function and respiratory muscle strength through spirometry test and manovacuometry, respectively. All authors confirm that there are no conflicts of interest. To compare the means or medians of variables between patients and healthy subjects, we used the unpaired t test or Mann-Whitney U test, respectively. The correlations were assessed by Pearson or Spearman coefficients according to the distribution of the studied variables. The probability of alpha error was set at 5% for all analyses.

RESULTS: Adolescent idiopathic scoliosis patients showed significant lower values of ISWD, VO2, and ventilation at the end of the ISWT, as well as lower FEV1 and FVC; they also presented significantly shallower slope of Delta VT/Delta lnVE, whereas VO2 related significantly with ISWD ($r = 0.80$), FVC ($r = 0.78$), FEV1 ($r = 0.73$), and Delta VT/Delta lnVE ($r = 0.58$).

CONCLUSIONS: Adolescent idiopathic scoliosis correlated to walking limitation and was associated to reduced pulmonary function and worse breathing pattern during exercise. Our results suggest that walking-based aerobic exercises should be encouraged in these patients. (C) 2014 Elsevier Inc. All rights reserved.

C1 [Sperandio, Evandro F.; Alexandre, Anderson S.; Yi, Liu C.; Poletto, Patricia R.; Vidotto, Milena C.; Dourado, Victor Z.] Univ Fed Sao Paulo, UNIFESP, Dept Human Movement Sci, Lab Human Motr, BR-11060001 Santos, SP, Brazil.

[Gotfryd, Alberto O.] Santa Casa da Misericordia Santos Hosp, Dept Orthoped, BR-11075900 Santos, SP, Brazil.

C3 Universidade Federal de Sao Paulo (UNIFESP)

RP Sperandio, EF (通讯作者)， Univ Fed Sao Paulo, UNIFESP, Dept Human Movement Sci, Lab Human Motr, Ave D Ana Costa 95-6, BR-11060001 Santos, SP, Brazil.

EM evandrosperandio@yahoo.com

RI Dourado, Victor/E-6784-2012; Poletto, Patrícia/Q-7994-2019; Sperandio,

E.F./K-5155-2015; Gotfryd, Alberto O/I-1868-2014; Vidotto, Milena

C/B-5930-2014; Yi, Liu C/K-8705-2013

OI Dourado, Victor/0000-0002-6222-3555; Poletto,

Patrícia/0000-0001-6709-8143; Sperandio, E.F./0000-0002-8580-458X;

Gotfryd, Alberto O/0000-0003-3143-2845; Yi, Liu C/0000-0003-0202-2337;

VIDOTTO, MILENA/0000-0003-2879-6541

CR Arena R, 2011, CIRCULATION, V123, P668, DOI 10.1161/CIRCULATIONAHA.109.914788

Athanasopoulos S, 1999, SCAND J MED SCI SPOR, V9, P36

Barrios C, 2005, SPINE, V30, P1610, DOI 10.1097/01.brs.0000169447.55556.01

Borowitz D, 2001, PEDIATR PULM, V31, P86, DOI 10.1002/1099-0496(200101)31:1<86::AID-PPUL1012>3.0.CO;2-3

Coelho Cristiane Cenachi, 2007, J. bras. pneumol., V33, P168, DOI 10.1590/S1806-37132007000200011

Czaprowski D, 2012, EUR SPINE J, V21, P1099, DOI 10.1007/s00586-011-2068-z

Alves VLD, 2006, CHEST, V130, P500, DOI 10.1378/chest.130.2.500

Alves VLD, 2009, SPINE, V34, pE926, DOI 10.1097/BRS.0b013e3181afd1b2

Dourado Victor Zuniga, 2013, J. bras. pneumol., V39, P190, DOI 10.1590/S1806-37132013000200010

Elias Hernandez M T, 1997, Arch Bronconeumol, V33, P498

Garber CE, 2011, MED SCI SPORT EXER, V43, P1334, DOI 10.1249/MSS.0b013e318213fefb

Highcock MP, 2002, CHEST, V121, P1555, DOI 10.1378/chest.121.5.1555

Johnston CE, 2011, SPINE, V36, P1096, DOI 10.1097/BRS.0b013e3181f8c931

Jurgensen SP, 2011, RESPIRATION, V81, P223, DOI 10.1159/000319037

KEARON C, 1993, AM REV RESPIR DIS, V148, P288, DOI 10.1164/ajrccm/148.2.288

KESTEN S, 1991, CHEST, V99, P663, DOI 10.1378/chest.99.3.663

Lenke LG, 2002, SPINE, V27, P2041, DOI 10.1097/00007632-200209150-00014

LISBOA C, 1985, AM REV RESPIR DIS, V132, P48

Martinez-Llorens J, 2010, EUR RESPIR J, V36, P393, DOI 10.1183/09031936.00025509

Neder JA, 2001, AM J RESP CRIT CARE, V164, P1481, DOI 10.1164/ajrccm.164.8.2103007

Newton PO, 2005, J BONE JOINT SURG AM, V87A, P1937, DOI 10.2106/JBJS.D.02209

Parent S, 2002, SPINE, V27, P2305, DOI 10.1097/00007632-200211010-00002

PEREIRA CAC, 2002, J PNEUMOL S3, V28, pS1

Pereira Carlos Alberto de Castro, 2007, J. bras. pneumol., V33, P397, DOI 10.1590/S1806-37132007000400008

Roca J, 1997, EUR RESPIR J, V10, P2662

Singh S, 2007, EUR RESPIR MONOGR, P148, DOI 10.1183/1025448x.00040007

SINGH SJ, 1994, EUR RESPIR J, V7, P2016

SINGH SJ, 1992, THORAX, V47, P1019, DOI 10.1136/thx.47.12.1019

Takahashi S, 2007, SPINE, V32, P106, DOI 10.1097/01.brs.0000251005.31255.25

Wasserman K., 2005, PRINCIPLES EXERCISE, V4th

Zwerink M, 2013, RESP MED, V107, P242, DOI 10.1016/j.rmed.2012.09.018

NR 31

TC 30

Z9 37

U1 5

U2 19

PU ELSEVIER SCIENCE INC

PI NEW YORK

PA STE 800, 230 PARK AVE, NEW YORK, NY 10169 USA

SN 1529-9430

EI 1878-1632

J9 SPINE J

JI Spine Journal

PD OCT

PY 2014

VL 14

IS 10

BP 2366

EP 2372

DI 10.1016/j.spinee.2014.01.041

PG 7

WC Clinical Neurology; Orthopedics

WE Science Citation Index Expanded (SCI-EXPANDED)

SC Neurosciences & Neurology; Orthopedics

GA AQ8RZ

UT WOS:000343100600011

PM 24486477

DA 2023-08-10

ER

PT J

AU Yilmaz, HG

AF Yilmaz, Hurriyet Gursel

TI Exercise Prescription in Idiopathic Scoliosis

SO TURKIYE FIZIKSEL TIP VE REHABILITASYON DERGISI-TURKISH JOURNAL OF
PHYSICAL MEDICINE AND REHABILITATION

LA Turkish

DT Article

DE Idiopathic scoliosis; Schroth exercise; scoliosis exercises

AB Causing rotation and bending of the spine, scoliosis leads to postural changes. Predominantly, the abdominal muscles, thorax, back, and low back extensors are affected. However, it also causes biomechanical changes of the pelvis, shoulder girdle, and even lower extremities and feet. For this reason, a thorough examination of the musculoskleletal system has to be performed for every patient. After determination of the problems, in addition to convenient therapeutic alternatives, exercise therapy and physiotherapy have to be planned individually. In the literature, there are various exercise methodologies, which are named Schroth, Dobomed, Method Lyonnaise, myofasial relase, and side shift. All of these exercise therapies share the same basic principles. They all are based on elongating the short muscles, relaxing the stretched muscles, and using diaphragmatic and deep breath techniques during the practice. Many of these applications place emphasis on specific training and certification in this field. In fact, every physiatrist has the knowledge of composing an exercise program for scoliosis. It is very important to make a good evaluation of the patient and to employ a good therapeutic approach for his problematic segments, muscles, and postural disturbances. It is essential to have experience and sufficient practice in this area, to spare the maximum time needed for the patients, and to follow them up by consistent controls.

C1 Halic Univ, Sagl Bilmleri Yuksek Okulu Fizyoterapi & Rehabi, Istanbul, Turkey.
[Yilmaz, Hurriyet Gursel] Halic Univ, Rehabil Bolumu, Istanbul, Turkey.

C3 Halic University; Halic University

RP Yilmaz, HG (通讯作者)， Halic Univ, Sagl Bilmleri Yuksek Okulu Fizyoterapi & Rehabi, Istanbul, Turkey.

EM hurriyet@formed.com.tr

RI Yılmaz, Hurriyet/ABH-1734-2021; Yilmaz, Hurriyet/B-6137-2014; Yılmaz,
Hurriyet Gürsel/ABE-9550-2021

OI Yilmaz, Hurriyet/0000-0002-6763-7706; Yılmaz, Hurriyet

Gürsel/0000-0002-7734-806X

CR [Anonymous], 2004, UL SKOL KONS TED K B

Asher MA, 2006, SCOLIOSIS SPINAL DIS, V1, DOI 10.1186/1748-7161-1-2

Schroth C., 2007, 3 DIMENSIONAL TREATM

Sosort, 2014, SOSORT 2014 SCOLIOSI

Weinstein SL, 2008, LANCET, V371, P1527, DOI 10.1016/S0140-6736(08)60658-3

Yilmaz H, 2012, SCOLIOSIS S1, V7, P37

NR 6

TC 4

Z9 4

U1 4

U2 32

PU AVES

PI SISLI

PA BUYUKDERE CAD 105-9, MECIDIYEKOY, SISLI, ISTANBUL 34096, TURKEY

SN 1302-0234

EI 1308-6316

J9 TURK FIZ TIP REHAB D

JI Turk. Fiz. Tip Rehabil. Derg.

PD OCT

PY 2014

VL 60

SU 2

BP S31

EP S35

DI 10.5152/tftrd.2014.84669

PG 5

WC Rehabilitation

WE Science Citation Index Expanded (SCI-EXPANDED)

SC Rehabilitation

GA CM0AQ

UT WOS:000357341000006

OA Bronze

DA 2023-08-10

ER

PT J

AU Yaman, O

Dalbayrak, S

AF Yaman, Onur

Dalbayrak, Sedat

TI Idiopathic Scoliosis

SO TURKISH NEUROSURGERY

LA English

DT Review

DE Idiopathic; Scoliosis; Review

ID CHARLESTON BENDING BRACE; BONE-MINERAL DENSITY; ESTROGEN-RECEPTOR;

NATURAL-HISTORY; INTEROBSERVER RELIABILITY; ANTERIOR INSTRUMENTATION;

PEDICLE SCREWS; GENE; CLASSIFICATION; POLYMORPHISMS

AB Scoliosis refers to curves exceeding 10 degrees observed through posterioanterior direct radiography. In fact, the diagnosis for idiopathic scoliosis is accepted to exclude already available causes. The aim of this paper was to review the etiopathogenesis, classification systems and the

treatment management of idiopathic scoliosis. A search in the National Library of Medicine (Pubmed) database using the key words 'idiopathic' and 'scoliosis' was performed. For the literature review, papers concerning the etiopathogenesis, classification and treatment were selected among these articles. A search in the National Library of Medicine (Pubmed) database using the key words 'idiopathic' and 'scoliosis' yielded 4518 articles published between 1947 and 2013. The main hypothesis put forward included genetic factors, hormonal factors, bone and connective tissue anomalies. King, Lenke, Coonrad and Peking Union Medical College (PUMC) classifications were the main classification systems for idiopathic scoliosis. Exercise, bracing and anterior, posterior or combined surgery when indicated are the choices for the treatment. Every idiopathic scoliosis case has to be managed to its own characteristics. It is the post-operative appearance that the surgeons are perhaps the least interested but the adolescent patients the most interested in.The aim of scoliosis surgery is to restore the spine without neurological deficit.

C1 [Yaman, Onur] Tepecik Educ & Training Hosp, Clin Neurosurg, Izmir, Turkey.

[Dalbayrak, Sedat] Neurospinal Acad, Istanbul, Turkey.

C3 Izmir Tepecik Training & Research Hospital

RP Yaman, O (通讯作者)， Tepecik Educ & Training Hosp, Clin Neurosurg, Izmir, Turkey.

EM dronuryaman@yahoo.com

CR Acaroglu E, 2009, SPINE, V34, pE659, DOI 10.1097/BRS.0b013e3181a3c7a2

Adams W., 1865, LECT PATHOLOGY TREAT

Akbarnia BA, 2005, SPINE, V30, pS46, DOI 10.1097/01.brs.0000175190.08134.73

Alden KJ, 2006, SPINE, V31, P1815, DOI 10.1097/01.brs.0000227264.23603.dc

Asher M, 2004, SPINE, V29, P2013, DOI 10.1097/01.brs.0000138275.49220.81

Bagnall K, 1999, J BONE JOINT SURG AM, V81A, P191, DOI 10.2106/00004623-199902000-00006

Bashiardes S, 2004, HUM GENET, V115, P81, DOI 10.1007/s00439-004-1121-y

BASSETT GS, 1986, J BONE JOINT SURG AM, V68A, P602, DOI 10.2106/00004623-198668040-00019

BELL M, 1995, AM J MED GENET, V55, P112, DOI 10.1002/ajmg.1320550126

Betz RR, 2003, SPINE, V28, pS255, DOI 10.1097/01.BRS.0000092484.31316.32

BUNNELL WP, 1986, SPINE, V11, P773, DOI 10.1097/00007632-198610000-00003

Campbell RM, 2004, J BONE JOINT SURG AM, V86A, P1659, DOI 10.2106/00004623-200408000-00009

Chan V, 2002, AM J HUM GENET, V71, P401, DOI 10.1086/341607

Cheng I, 2005, SPINE, V30, P2104, DOI 10.1097/01.brs.0000179261.70845.b7

Cobb JR., 1948, INSTR COURSE LECT, V5, P261

Coillard C, 2007, J PEDIATR ORTHOPED, V27, P375, DOI 10.1097/01.bpb.0000271330.64234.db

Coonrad RW, 1998, SPINE, V23, P1380, DOI 10.1097/00007632-199806150-00016

COTREL Y, 1988, CLIN ORTHOP RELAT R, P10

COWELL HR, 1972, CLIN ORTHOP RELAT R, P121, DOI 10.1097/00003086-197207000-00018

Cummings RJ, 1998, J BONE JOINT SURG AM, V80A, P1107

Dobosiewicz Krystyna, 2002, Stud Health Technol Inform, V91, P336

Dubousset J., 1983, ORTHOP T, V7, P7

Edery P, 2011, EUR J HUM GENET, V19, P865, DOI 10.1038/ejhg.2011.31

EDGAR MA, 1987, ORTHOPEDICS, V10, P931

Esposito T, 2009, J STEROID BIOCHEM, V116, P56, DOI 10.1016/j.jsbmb.2009.04.010

Farkas A, 1941, J BONE JOINT SURG, V23, P607

Filardo EJ, 2005, TRENDS ENDOCRIN MET, V16, P362, DOI 10.1016/j.tem.2005.08.005

Filho NA, 1971, J BONE JOINT SURG AM, V53, P199

Garland HG, 1934, BRIT MED J, V1934, P328, DOI 10.1136/bmj.1.3816.328

Grivas TB, 1991, J BONE JOINT SURG BR, V32, P32

Hannes B, 2002, SPINE, V27, P762, DOI 10.1097/00007632-200204010-00015

HARRINGTON PR, 1962, J BONE JOINT SURG AM, V44, P591, DOI 10.2106/00004623-196244040-00001

Hee HT, 2007, SPINE, V32, P1533, DOI 10.1097/BRS.0b013e318067dc3d

Herring J.A., 2002, TACHDJIANS PEDIAT OR, V3rd ed., P213

Horton D, 2002, EMERY RIMOINS PRINCI, P4236

JAMES JIP, 1954, J BONE JOINT SURG BR, V36, P36, DOI 10.1302/0301-620X.36B1.36

Jiang J, 2012, SPINE, V37, P41, DOI 10.1097/BRS.0b013e31820e71e3

Justice CM, 2003, SPINE, V28, P589, DOI 10.1097/00007632-200303150-00014

KAROL LA, 1993, J BONE JOINT SURG AM, V75A, P1804, DOI 10.2106/00004623-199312000-00010

Katz DE, 1997, SPINE, V22, P1302, DOI 10.1097/00007632-199706150-00005

KING HA, 1983, J BONE JOINT SURG AM, V65, P1302, DOI 10.2106/00004623-198365090-00012

Kou I, 2013, NAT GENET, V45, P676, DOI 10.1038/ng.2639

Leboeuf D, 2009, TRENDS ENDOCRIN MET, V20, P147, DOI 10.1016/j.tem.2008.12.004

Lee JS, 2010, J BONE JOINT SURG BR, V92B, P1118, DOI 10.1302/0301-620X.92B8.23676

Lenke LG, 1998, J BONE JOINT SURG AM, V80A, P1097, DOI 10.2106/00004623-199808000-00002

Lenke LG, 2001, J BONE JOINT SURG AM, V83A, P1169, DOI 10.2106/00004623-200108000-00006

Letellier K, 2008, J PINEAL RES, V45, P383, DOI 10.1111/j.1600-079X.2008.00603.x

LITTLE DG, 1994, J PEDIATR ORTHOPED, V14, P569, DOI 10.1097/01241398-199409000-00003

LONSTEIN JE, 1994, J BONE JOINT SURG AM, V76A, P1207, DOI 10.2106/00004623-199408000-00011

Lonstein JE, 2006, CLIN ORTHOP RELAT R, P248, DOI 10.1097/01.blo.0000198725.54891.73

MACHIDA M, 1995, J BONE JOINT SURG BR, V77B, P134, DOI 10.1302/0301-620X.77B1.7822371

MACHIDA M, 1994, J PEDIATR ORTHOPED, V14, P329, DOI 10.1097/01241398-199405000-00010

Maggiolini M, 2010, J ENDOCRINOL, V204, P105, DOI 10.1677/JOE-09-0242

Mamyama Toni, 2002, Stud Health Technol Inform, V91, P361

Man GCW, 2011, J PINEAL RES, V50, P395, DOI 10.1111/j.1600-079X.2011.00857.x

Marks DS, 1996, SPINE, V21, P1884, DOI 10.1097/00007632-199608150-00010

McIntire KL, 2008, J SPINAL DISORD TECH, V21, P349, DOI 10.1097/BSD.0b013e318145b7e9

Miller NH, 2012, HUM HERED, V74, P36, DOI 10.1159/000343751

Millner P A, 1996, Eur Spine J, V5, P362, DOI 10.1007/BF00301963

MOE JH, 1984, CLIN ORTHOP RELAT R, P35

Montanaro L, 2006, SCOLIOSIS SPINAL DIS, V1, DOI 10.1186/1748-7161-1-21

Mooney V, 2003, ORTHOPEDICS, V26, P167

Morocz M, 2011, SPINE, V36, pE123, DOI 10.1097/BRS.0b013e318a511b0e

NACHEMSON AL, 1995, J BONE JOINT SURG AM, V77A, P815, DOI 10.2106/00004623-199506000-00001

Negrini Stefano, 2003, Pediatr Rehabil, V6, P227, DOI: 10.1080/13638490310001636781

Newton PO, 2003, SPINE, V28, pS249, DOI 10.1097/01.BRS.0000092475.04293.F5

Niemeyer T, 2006, SPINE, V311, P2103

Niemeyer T, 2006, SPINE, V311, P2108

Ocaka L, 2008, J MED GENET, V45, P87, DOI 10.1136/jmg.2007.051896

Ogon M, 2002, SPINE, V27, P858, DOI 10.1097/00007632-200204150-00014

Otman S, 2005, SAUDI MED J, V26, P1429

Park WW, 2009, EUR SPINE J, V18, P1920, DOI 10.1007/s00586-009-1129-z

Peng Y, 2012, INT ORTHOP, V36, P671, DOI 10.1007/s00264-011-1374-8

PICAULT C, 1986, SPINE, V11, P777, DOI 10.1097/00007632-198610000-00004

Picetti GD, 2002, NEUROSURGERY, V51, P978, DOI 10.1097/00006123-200210000-00023

Potter BK, 2005, SPINE, V30, P1859, DOI 10.1097/01.brs.0000174118.72916.96

Price CT, 1997, J PEDIATR ORTHOPED, V17, P703, DOI 10.1097/00004694-199711000-00002

Prossnitz ER, 2008, J STEROID BIOCHEM, V109, P350, DOI 10.1016/j.jsbmb.2008.03.006

Qiu GX, 2008, SPINE, V33, pE836, DOI 10.1097/BRS.0b013e318187bb10

Qiu GX, 2005, SPINE, V30, P1419, DOI 10.1097/01.brs.0000166531.52232.0c

Qiu Y, 2012, SPINE, V37, P127, DOI 10.1097/BRS.0b013e31823e5890

RICHARDS BS, 1992, SPINE, V17, pS282

Richards BS, 2005, SPINE, V30, P2068, DOI 10.1097/01.brs.0000178819.90239.d0

RISEBOROUGH EJ, 1973, J BONE JOINT SURG AM, VA 55, P974, DOI 10.2106/00004623-197355050-00006

RISSER J C, 1958, Clin Orthop, V11, P111

ROBIN GC, 1975, J BONE JOINT SURG BR, V57, P146, DOI 10.1302/0301-620X.57B2.146

ROYE DP, 1992, SPINE, V17, pS270

Salehi LB, 2002, HUM GENET, V111, P401, DOI 10.1007/s00439-002-0785-4

Sanders JO, 2006, SPINE, V31, P2289, DOI 10.1097/01.brs.0000236844.41595.26

Shaughnessy WJ, 2007, ORTHOP CLIN N AM, V38, P469, DOI 10.1016/j.ocl.2007.07.002

Shaw GTW, 2011, PLOS ONE, V6, DOI 10.1371/journal.pone.0029314

STAGNARA P, 1982, SPINE, V7, P335, DOI 10.1097/00007632-198207000-00003

Sucato DJ, 2004, SPINE, V29, P554, DOI 10.1097/01.BRS.0000106495.91477.92

Suh KT, 2010, EUR SPINE J, V19, P1545, DOI 10.1007/s00586-010-1385-y

Suk SI, 2005, SPINE, V30, P1602, DOI 10.1097/01.brs.0000169452.50705.61

THILLARD MJ, 1959, CR HEBD ACAD SCI, V248, P1238

Trivedi JM, 2001, J PEDIATR ORTHOPED, V21, P277, DOI 10.1097/00004694-200105000-00002

Waller T, 2013, BIOMED ENG ONLINE, V12, DOI 10.1186/1475-925X-12-1

Wang H, 2008, SPINE, V33, P2199, DOI 10.1097/BRS.0b013e31817c03f9

WEINSTEIN SL, 1983, J BONE JOINT SURG AM, V65, P447, DOI 10.2106/00004623-198365040-00004

Weiss H R, 1992, Ital J Orthop Traumatol, V18, P395

Weiss HR, 2006, SCOLIOSIS SPINAL DIS, V1, DOI [10.1186/1748-7161-1-5, 10.1186/1748-7161-1-1]

Weiss Hans-Rudolf, 2003, Pediatr Rehabil, V6, P23, DOI 10.1080/1363849031000095288

Wise CA, 2000, SPINE, V25, P2372, DOI 10.1097/00007632-200009150-00017

Wu J, 2006, SPINE, V31, P1131, DOI 10.1097/01.brs.0000216603.91330.6f

WYNNEDAVIES R, 1975, J BONE JOINT SURG BR, V57, P138, DOI 10.1302/0301-620X.57B2.138

Zhao Dong, 2009, Orthop Surg, V1, P222, DOI 10.1111/j.1757-7861.2009.00038.x

Zhou S, 2012, STUD HEALTH TECHNOL, V176, P47, DOI 10.3233/978-1-61499-067-3-47

NR 108

TC 39

Z9 43

U1 4

U2 30

PU TURKISH NEUROSURGICAL SOC

PI BAHCELIEVLER

PA TASKENT CADDESI 13-4, BAHCELIEVLER, ANKARA 06500, TURKEY

SN 1019-5149

J9 TURK NEUROSURG

JI Turk. Neurosurg.

PD SEP

PY 2014

VL 24

IS 5

BP 646

EP 657

DI 10.5137/1019-5149.JTN.8838-13.0

PG 12

WC Clinical Neurology; Surgery

WE Science Citation Index Expanded (SCI-EXPANDED)

SC Neurosciences & Neurology; Surgery

GA AR4AZ

UT WOS:000343531700007

PM 25269032

OA Bronze

DA 2023-08-10

ER

PT J

AU Monticone, M

Ambrosini, E

Cazzaniga, D

Rocca, B

Ferrante, S

AF Monticone, Marco

Ambrosini, Emilia

Cazzaniga, Daniele

Rocca, Barbara

Ferrante, Simona

TI Active self-correction and task-oriented exercises reduce spinal deformity and improve quality of life in subjects with mild adolescent idiopathic scoliosis. Results of a randomised controlled trial

SO EUROPEAN SPINE JOURNAL

LA English

DT Article

DE Adolescent idiopathic scoliosis; Rehabilitation; Self-correction; Task-oriented exercises; Education

ID LOW-BACK-PAIN; CURVE PROGRESSION; PATIENT QUESTIONNAIRE; SKELETAL

MATURITY; EFFICACY; RELIABILITY; VALIDITY; THERAPY; COHORT; BRACE

AB To evaluate the effect of a programme of active self-correction and task-oriented exercises on spinal deformities and health-related quality of life (HRQL) in patients with mild adolescent idiopathic scoliosis (AIS) (Cobb angle < 25A degrees).

This was a parallel-group, randomised, superiority-controlled study in which 110 patients were randomly assigned to a rehabilitation programme consisting of active self-correction, task-oriented spinal exercises and education (experimental group, 55 subjects) or traditional spinal exercises (control group, 55 subjects). Before treatment, at the end of treatment (analysis at skeletal maturity), and 12 months later (follow-up), all of the patients underwent radiological deformity (Cobb angle), surface deformity (angle of trunk rotation) and HRQL evaluations (SRS-22 questionnaire). A linear mixed model for repeated measures was used for each outcome measure.

There were main effects of time ($p < 0.001$), group ($p < 0.001$) and time by group interaction ($p < 0.001$) on radiological deformity: training in the experimental group led to a significant improvement (decrease in Cobb angle of > 5A degrees), whereas the control group remained stable. Analysis of all of the secondary outcome measures revealed significant effects of time, group and time by group interaction in favour of the experimental group.

The programme of active self-correction and task-oriented exercises was superior to traditional exercises in reducing spinal deformities and enhancing the HRQL in patients with mild AIS. The effects lasted for at least 1 year after the intervention ended.

C1 [Monticone, Marco; Ambrosini, Emilia; Cazzaniga, Daniele; Rocca, Barbara] Salvatore Maugeri Fdn, Inst Care & Res, IRCCS, Phys Med & Rehabil Unit,Sci Inst Lissone, I-20035 Milan, Italy.

[Ambrosini, Emilia; Ferrante, Simona] Politecn Milan, Dept Elect Informat & Bioengn, Neuroengn & Med Robot Lab, I-20133 Milan, MI, Italy.

C3 Istituti Clinici Scientifici Maugeri IRCCS; Polytechnic University of

Milan

RP Monticone, M (通讯作者)， Salvatore Maugeri Fdn, Inst Care & Res, IRCCS, Phys Med & Rehabil Unit,Sci Inst Lissone, Via Monsignor Bernasconi 16, I-20035 Milan, Italy.

EM marco.monticone@fsm.it

RI Monticone, Marco/AAC-4494-2022; Ferrante, Simona/K-4122-2016; Cazzaniga,

Daniele/F-8914-2019; Ambrosini, Emilia/R-1371-2016

OI Ferrante, Simona/0000-0002-7835-1965; Cazzaniga,

Daniele/0000-0001-8248-5790; Ambrosini, Emilia/0000-0002-6527-0779

CR [Anonymous], 2005, ZHONGGUO LINCHUANG K

ASCANI E, 1986, SPINE, V11, P784, DOI 10.1097/00007632-198610000-00007

Asher M, 2003, SPINE, V28, P63, DOI 10.1097/00007632-200301010-00015

Asher MA, 2006, SCOLIOSIS SPINAL DIS, V1, DOI 10.1186/1748-7161-1-2

BUNNELL WP, 1984, J BONE JOINT SURG AM, V66A, P1381, DOI 10.2106/00004623-198466090-00010

BUNNELL WP, 1993, SPINE, V18, P1572, DOI 10.1097/00007632-199309000-00001

Burton AK, 2006, EUR SPINE J, V15, pS136, DOI 10.1007/s00586-006-1070-3

de Jong JR, 2005, CLIN J PAIN, V21, P9, DOI 10.1097/00002508-200501000-00002

Diab AA, 2012, CLIN REHABIL, V26, P1123, DOI 10.1177/0269215512447085

Durmala Jacek, 2003, Ortop Traumatol Rehabil, V5, P80

el-Sayyad M, 1994, Int J Rehabil Res, V17, P70, DOI 10.1097/00004356-199403000-00008

Fielding S, 2012, CONTEMP CLIN TRIALS, V33, P461, DOI 10.1016/j.cct.2011.12.002

GOLDBERG MS, 1994, SPINE, V19, P1562, DOI 10.1097/00007632-199407001-00004

LONSTEIN JE, 1984, J BONE JOINT SURG AM, V66A, P1061, DOI 10.2106/00004623-198466070-00013

Mamyama Toni, 2002, Stud Health Technol Inform, V91, P361

Matthews JNS, 2006, INTRO RANDOMIZED CON, P43

McIntire KL, 2008, J SPINAL DISORD TECH, V21, P349, DOI 10.1097/BSD.0b013e318145b7e9

Monticone M, 2010, SPINE, V35, pE1412, DOI 10.1097/BRS.0b013e3181e88981

Mooney V, 2003, ORTHOPEDICS, V26, P167

Mordecai SC, 2012, EUR SPINE J, V21, P382, DOI 10.1007/s00586-011-2063-4

Morley S, 2011, PAIN, V152, pS99, DOI 10.1016/j.pain.2010.10.042

MORRISSY RT, 1990, J BONE JOINT SURG AM, V72A, P320, DOI 10.2106/00004623-199072030-00002

NACHEMSON AL, 1995, J BONE JOINT SURG AM, V77A, P815, DOI 10.2106/00004623-199506000-00001

Negrini S, 2008, J REHABIL MED, V40, P451, DOI 10.2340/16501977-0195

Otman S, 2005, SAUDI MED J, V26, P1429

PONSETI IV, 1950, J BONE JOINT SURG AM, V32-A, P381, DOI 10.2106/00004623-195032020-00017

Shea KG, 1998, SPINE, V23, P551, DOI 10.1097/00007632-199803010-00007

Siddiqui O, 2009, J BIOPHARM STAT, V19, P227, DOI 10.1080/10543400802609797

Tan KJ, 2009, SPINE, V34, P697, DOI 10.1097/BRS.0b013e31819c9431

Tanure MC, 2010, SPINE J, V10, P769, DOI 10.1016/j.spinee.2010.02.020

WEINSTEIN SL, 1983, J BONE JOINT SURG AM, V65, P447, DOI 10.2106/00004623-198365040-00004

Weinstein SL, 2008, LANCET, V371, P1527, DOI 10.1016/S0140-6736(08)60658-3

Weinstein SL, 2013, NEW ENGL J MED, V369, P1512, DOI 10.1056/NEJMoa1307337

Weiss H R, 1997, Pediatr Rehabil, V1, P35

Weiss H-R, 2006, Pediatr Rehabil, V9, P190, DOI 10.1080/13638490500079583

Weiss Hans-Rudolf, 2003, Pediatr Rehabil, V6, P23, DOI 10.1080/1363849031000095288

Wong MS, 2008, SPINE, V33, P1360, DOI 10.1097/BRS.0b013e31817329d9

NR 37

TC 121

Z9 128

U1 0

U2 59

PU SPRINGER

PI NEW YORK

PA 233 SPRING ST, NEW YORK, NY 10013 USA

SN 0940-6719

EI 1432-0932

J9 EUR SPINE J

JI Eur. Spine J.

PD JUN

PY 2014

VL 23

IS 6

BP 1204

EP 1214

DI 10.1007/s00586-014-3241-y

PG 11

WC Clinical Neurology; Orthopedics

WE Science Citation Index Expanded (SCI-EXPANDED)

SC Neurosciences & Neurology; Orthopedics

GA AK0TR

UT WOS:000338128900008

PM 24682356

OA Green Submitted

DA 2023-08-10

ER

PT J

AU Bettany-Saltikov, J

Parent, E

Romano, M

Villagrasa, M

Negrini, S

AF Bettany-Saltikov, J.

Parent, E.

Romano, M.

Villagrasa, M.

Negrini, S.

TI Physiotherapeutic scoliosis-specific exercises for adolescents with

idiopathic scoliosis

SO EUROPEAN JOURNAL OF PHYSICAL AND REHABILITATION MEDICINE

LA English

DT Review

DE Scoliosis; Exercise; Therapeutics

ID CONSERVATIVE TREATMENT; PHYSICAL EXERCISES; SEAS.02 EXERCISES; BRACE

TREATMENT; BACK-PAIN; FOLLOW-UP; EFFICACY; SURGERY; REHABILITATION;

PROGRESSION

AB The use of exercises for the treatment of Adolescents with Idiopathic Scoliosis is controversial. Whilst exercises are routinely used in a number of central and southern European countries, most centres in the rest of the world (mainly in Anglo-Saxon countries), do not advocate its use. One of the reasons for this is that. many health care professionals are usually not conversant with the differences between generalised physiotherapy exercises and physiotherapeutic scoliosis-specific exercises (PSSE): while the former are generic exercises usually consisting of low-impact stretching and strengthening activities like yoga, Pilates and the Alexander technique, PSSE consist of a program of curve-specific exercise protocols which are individually adapted to a patients' curve site, magnitude and clinical characteristics. PSSEs are performed with the therapeutic aim of reducing the deformity and preventing its progression. It also aims to stabilise the improvements achieved with the ultimate goal of limiting the need for corrective braces or the necessity of surgery This paper introduces the different 'Schools' and approaches of PSSE currently practiced (Scientific Exercise Approach to Scoliosis - SEAS, Schroth, Barcelona Scoliosis Physical Therapy School - BSPTS, Dobomed, Side Shift, Functional Individual Therapy of Scoliosis - FITS and Lyon) and discusses their commonalities and differences.

C1 [Bettany-Saltikov, J.] Univ Teesside, Inst Hlth & Social Care, Middlesbrough, Cleveland, united kingdom.

[Parent, E.] Univ Alberta, Dept Phys Therapy, Edmonton, AB T6G 2G4, Canada.

[Romano, M.; Negrini, S.] ISICO Italian Sci Spine Inst, Milan, Italy.

[Villagrasa, M.] Elena Salva Inst, Barcelona, Spain.

C3 University of Teesside; University of Alberta

RP Parent, E (通讯作者)， Univ Alberta, Dept Phys Therapy, 2-50 Corbett Hall, Edmonton, AB T6G 2G4, Canada.

EM eparent@ualberta.ca

RI Bettany-Saltikov (nee Damato), Josette Arielle/C-5388-2011; Negrini,

Stefano/B-6667-2013

OI Bettany-Saltikov (nee Damato), Josette Arielle/0000-0001-7784-500X;

Parent, Eric/0000-0003-3835-0607; Negrini, Stefano/0000-0002-1878-2747

CR Abbott A, 2013, BMC MUSCULOSKEL DIS, V14, DOI 10.1186/1471-2474-14-261

American Academy of Orthopaedic Surgeons, 2008, BURD MUSC DIS US

[Anonymous], 1992, PHYSIOTHERAPY

[Anonymous], KINESITHERAPIE SCI

[Anonymous], CHIN J CLIN REHABIL

Asher MA, 2006, SCOLIOSIS SPINAL DIS, V1, DOI 10.1186/1748-7161-1-2

Beausejour M, 2007, SPINE, V32, P1349, DOI 10.1097/BRS.0b013e318059b5f7

Berg DC, 2002, MED BIOL ENG COMPUT, V40, P290, DOI 10.1007/BF02344210

BETTANY J, 1995, ST HEAL T, V15, P321

Bettany JA, 1993, INVESTIGATION TOPOGR

Bettany-Saltikov J, 2012, PHYSICAL THERAPY PERSPECTIVES IN THE 21ST CENTURY - CHALLENGES AND POSSIBILITIES, P3

Bialek M, 2011, SCOLIOSIS SPINAL DIS, V6, DOI 10.1186/1748-7161-6-25

Bialek M, 2009, STUD HEALTH TECHNOL, V135, P250

Blicharska I, 2012, STUD HEALTH TECHNOL, V176, P387, DOI 10.3233/978-1-61499-067-3-387

Climent JM, 1999, SPINE, V24, P1903, DOI 10.1097/00007632-199909150-00007

Cobb JR., 1948, INSTR COURSE LECT, V5, P261

Danielsson AJ, 2001, SPINE, V26, P516

Danielsson AJ, 2003, SPINE, V28, pE373, DOI 10.1097/01.BRS.0000084267.41183.75

Danielsson AJ, 2003, SPINE, V28, P2078

de Mauroy JC, 2014, KINESITHERAPIE

Dobosiewicz K, 2009, STUD HEALTH TECHNOL, V135, P228

FALLSTROM K, 1986, SPINE, V11, P756

Freidel K, 2002, ST HEAL T, V88, P24

Freidel K, 2002, J ORTHOP SPORT PHYS, V32, P536

Fusco C., 2011, Physiotherapy Theory and Practice, V27, P80, DOI 10.3109/09593985.2010.533342

Hawes Martha, 2006, Pediatr Rehabil, V9, P318, DOI 10.1080/13638490500402264

Hawes MC, 2008, DISABIL REHABIL, V30, P808, DOI 10.1080/09638280801889972

Lenssinck MLB, 2005, PHYS THER, V85, P1329, DOI 10.1093/ptj/85.12.1329

Lonstein JE, 2006, CLIN ORTHOP RELAT R, P248, DOI 10.1097/01.blo.0000198725.54891.73

MACLEAN WE, 1989, J PEDIATR ORTHOPED, V9, P257

Maruyama Toru, 2003, Pediatr Rehabil, V6, P215

Maruyama T, 2009, STUD HEALTH TECHNOL, V135, P246

MAYO NE, 1994, SPINE, V19, P1573, DOI 10.1097/00007632-199407001-00005

McIntire KL, 2008, J SPINAL DISORD TECH, V21, P349, DOI 10.1097/BSD.0b013e318145b7e9

Mooney V, 2003, ORTHOPEDICS, V26, P167

Negrini A, 2008, SCOLIOSIS SPINAL DIS, V3, DOI 10.1186/1748-7161-3-20

Negrini S, 2008, DISABIL REHABIL, V30, P772, DOI 10.1080/09638280801889568

Negrini S, 2005, Eura Medicophys, V41, P183

Negrini S., 2010, BRACES IDIOPATHIC SC

Negrini S, 2007, COCHRANE DATABASE SY

Negrini S, 2008, J REHABIL MED, V40, P451, DOI 10.2340/16501977-0195

Negrini Stefano, 2007, J Surg Orthop Adv, V16, P98

Negrini S, 2008, DISABIL REHABIL, V30, P731, DOI 10.1080/09638280801889485

Negrini S, 2006, ST HEAL T, V123, P523

Negrini S, 2006, ST HEAL T, V123, P519

Negrini S, 2006, SCOLIOSIS SPINAL DIS, V1, DOI 10.1186/1748-7161-1-14

Negrini S, 2006, SCOLIOSIS SPINAL DIS, V1, DOI 10.1186/1748-7161-1-4

Negrini Stefano, 2003, Pediatr Rehabil, V6, P227, DOI: 10.1080/13638490310001636781

Negrini S, 2012, SCOLIOSIS SPINAL DIS, V7, DOI 10.1186/1748-7161-7-3

Negrini S, 2009, STUD HEALTH TECHNOL, V135, P395

Otman S, 2005, SAUDI MED J, V26, P1429

Parent EC, 2013, SCOLIOSIS S1, V8

Parent Stefan, 2005, Instr Course Lect, V54, P529

PEHRSSON K, 1992, SPINE, V17, P1091, DOI 10.1097/00007632-199209000-00014

Pehrsson K, 2001, THORAX, V56, P388, DOI 10.1136/thorax.56.5.388

Raso VJ, 1987, SURFACE TOPOGRAPHY S, P13

Rigo M, 2003, Pediatr Rehabil, V6, P209

Rigo M, 1991, P BOOK 3 11 INT C WO, P1319

Rigo M, 2009, STUD HEALTH TECHNOL, V135, P208

Rigo MD, 2010, SCOLIOSIS SPINAL DIS, V5, DOI 10.1186/1748-7161-5-27

Romano M, 2008, STUD HLTH TECHNOL IN, V140, P331

Romano M, 2009, COCHRANE DB SYST REV, V4

Romano M, 2012, COCHRANE DB SYST REV, DOI 10.1002/14651858.CD007837.pub2

Romano M, 2009, STUD HEALTH TECHNOL, V135, P191

Romano M, 2008, SCOLIOSIS SPINAL DIS, V3, DOI 10.1186/1748-7161-3-2

Rowe DE, 1997, J BONE JOINT SURG AM, V79A, P664, DOI 10.2106/00004623-199705000-00005

Schreiber S, 2012, 9 INT C CONS MAN SPI, P44

Schreiber S, 2013, SCOLIOSIS S2, V8

Scoliosis Research Society, 2014, SCOL RES SOC AD ID S

Shindle Michael K, 2006, J Surg Orthop Adv, V15, P43

Spinal Deformity Study Group, 2004, SPIN DEF STUD GROUP, P1

STONE B, 1979, PHYS THER, V59, P759, DOI 10.1093/ptj/59.6.759

Ugwonali Obinwanne F, 2004, Spine J, V4, P254

Weinstein SL, 2003, JAMA-J AM MED ASSOC, V289, P559, DOI 10.1001/jama.289.5.559

Weinstein SL, 2008, LANCET, V371, P1527, DOI 10.1016/S0140-6736(08)60658-3

Weinstein SL, 2013, NEW ENGL J MED, V369, P1512, DOI 10.1056/NEJMoa1307337

Weiss H R, 1993, Eur Spine J, V1, P240, DOI 10.1007/BF00298367

Weiss H R, 1992, Ital J Orthop Traumatol, V18, P395

Weiss H R, 1997, Pediatr Rehabil, V1, P35

Weiss H.-R., 1993, EUR J PHYS MED REHAB, V3, P91

Weiss H-R, 2006, Pediatr Rehabil, V9, P190, DOI 10.1080/13638490500079583

Weiss HR, 2008, DISABIL REHABIL, V30, P799, DOI 10.1080/09638280801889717

Weiss HR, 2006, SCOLIOSIS SPINAL DIS, V1, DOI [10.1186/1748-7161-1-5, 10.1186/1748-7161-1-1]

Weiss HR, 2006, SCOLIOSIS SPINAL DIS, V1, DOI 10.1186/1748-7161-1-6

Weiss Hans-Rudolf, 2003, Pediatr Rehabil, V6, P183

Weiss Hans-Rudolf, 2002, Stud Health Technol Inform, V91, P352

Weiss Hans-Rudolf, 2003, Pediatr Rehabil, V6, P23, DOI 10.1080/1363849031000095288

Weiss HR, 2011, SCOLIOSIS SPINAL DIS, V6, DOI 10.1186/1748-7161-6-17

Weiss HR, 2008, SCOLIOSIS SPINAL DIS, V3, DOI 10.1186/1748-7161-3-9

Weiss HR, 2002, ST HEAL T, V88, P304

WEISS HR, 1995, Z ORTHOP GRENZGEB, V133, P114, DOI 10.1055/s-2008-1039421

WEISS HR, 1991, SPINE, V16, P88, DOI 10.1097/00007632-199101000-00016

World Health Organization, 2001, INT CLASS FUNCT DIS, P1

Zaina F, 2009, SCOLIOSIS SPINAL DIS, V4, DOI 10.1186/1748-7161-4-8

NR 94

TC 51

Z9 58

U1 2

U2 114

PU EDIZIONI MINERVA MEDICA

PI TURIN

PA CORSO BRAMANTE 83-85 INT JOURNALS DEPT., 10126 TURIN, ITALY

SN 1973-9087

EI 1973-9095

J9 EUR J PHYS REHAB MED

JI Eur. J. Phys. Rehabil. Med.

PD FEB

PY 2014

VL 50

IS 1

BP 111

EP 121

PG 11

WC Rehabilitation

WE Science Citation Index Expanded (SCI-EXPANDED)

SC Rehabilitation

GA AI4GP

UT WOS:000336824000014

PM 24525556

DA 2023-08-10

ER

PT J

AU Noh, DK

You, JH

Koh, JH

Kim, H

Kim, D

Ko, SM

Shin, JY

AF Noh, Dong Koog

You, Joshua (Sung)-H

Koh, Jae-Hyun

Kim, Hoseong

Kim, Donghyun

Ko, Sung-Mok

Shin, Ji-Youn

TI Effects of novel corrective spinal technique on adolescent idiopathic scoliosis as assessed by radiographic imaging

SO JOURNAL OF BACK AND MUSCULOSKELETAL REHABILITATION

LA English

DT Article

DE Adolescent idiopathic scoliosis; X-ray imaging; corrective spinal

exercise

ID TERM-FOLLOW-UP; RELIABILITY; PELVIS; HEALTH; BRACE

AB OBJECTIVE: To compare the therapeutic effects of a 3-dimensional corrective spinal technique (CST) and a conventional exercise program (CE) on altered spinal curvature and health related quality-of-life in patients with adolescent idiopathic scoliosis (AIS).

METHODS: Adolescents with idiopathic scoliosis (N = 32, 6 males and 26 females) between 10 and 19 years of age (14.34 +/- 2.60 years) were recruited and underwent the CST or CE for 60 minutes/day, 2-3 times a week, and an average of total 30 sessions. Diagnostic X-ray imaging technique was used to determine intervention-related changes in the Cobb angle, thoracic kyphosis angle, lumbar lordosis angle, sacral slope, pelvic tilt, pelvic incidence, and vertebral rotation (Nash-Moe method). The Scoliosis Research Society-22 (SRS-22) health related quality-of-life questionnaire was used. Data were analysed using independent t-test, paired t-test, and non-parametric Mann-Whitney U-test at $p < 0.05$.

RESULTS: CST showed greater improvements in Cobb angle ($p = 0.003$), vertebral rotation ($p = 0.000$), and SRS-22 scores (self-image and treatment satisfaction subscale scores and total score, $p = 0.026$, $p = 0.039$, and $p = 0.041$, respectively) as compared to the controls. There were no significant changes in the other measures between the two groups.

CONCLUSIONS: This is the first clinical trial to investigate the effects of the 3-dimensional CST on spinal curvatures and health related quality-of-life in AIS, providing the important clinical rationale and compelling evidence for the effective management of AIS.

C1 [Noh, Dong Koog; Koh, Jae-Hyun; Kim, Hoseong; Kim, Donghyun; Ko, Sung-Mok; Shin, Ji-Youn] Seoul Hyu Clin, Dept Phys Med & Rehabil, Gyeonggi Do, South Korea.

[Noh, Dong Koog; You, Joshua (Sung)-H] Yonsei Univ, Coll Hlth Sci, Dept Phys Therapy, Kangwon Do, South Korea.

C3 Yonsei University

RP You, JH (通讯作者)， Yonsei Univ, Dept Phys Therapy, 1 Yonsei Dae Kil, Wonju 220710, Kangwon Do, South Korea.

EM neurorehab@yonsei.ac.kr

CR [Anonymous], 2007, REHABILITATION SPINE

Asher M, 2003, SPINE, V28, P63, DOI 10.1097/00007632-200301010-00015

Bagnall KM, 2009, SCOLIOSIS SPINAL DIS, V4, DOI 10.1186/1748-7161-4-28

Danielsson AJ, 2001, EUR SPINE J, V10, P278, DOI 10.1007/s005860100309

Dobosiewicz Krystyna, 2002, Stud Health Technol Inform, V91, P348

DUVALBEAUPERE G, 1992, ANN BIOMED ENG, V20, P451, DOI 10.1007/BF02368136

Greiner KA, 2002, AM FAM PHYSICIAN, V65, P1817

Guo X, 2003, J BONE JOINT SURG BR, V85B, P1026, DOI 10.1302/0301-620X.85B7.14046

Hopkins WG, 2000, SPORTS MED, V30, P1, DOI 10.2165/00007256-200030010-00001

Mac-Thiong JM, 2003, SPINE, V28, P1404, DOI 10.1097/01.BRS.0000067118.60199.D1

Mamyama T, 2002, STUD HLTH TECHNOL IN, V91, P631

McIntire KL, 2008, J SPINAL DISORD TECH, V21, P349, DOI 10.1097/BSD.0b013e318145b7e9

Mooney V, 2000, J SPINAL DISORD, V13, P102, DOI 10.1097/00002517-200004000-00002

MORRISSY RT, 1990, J BONE JOINT SURG AM, V72A, P320, DOI 10.2106/00004623-199072030-00002

NASH CL, 1969, J BONE JOINT SURG AM, VA 51, P223, DOI 10.2106/00004623-196951020-00002

Negrini S, 2008, J REHABIL MED, V40, P451, DOI 10.2340/16501977-0195

Negrini S, 2011, SPINE J, V11, P369, DOI 10.1016/j.spinee.2010.12.001

Otman S, 2005, SAUDI MED J, V26, P1429

PEHRSSON K, 1992, SPINE, V17, P1091, DOI 10.1097/00007632-199209000-00014

Reem J, 2009, SKELETAL RADIOL, V38, P371, DOI 10.1007/s00256-008-0603-8

Roubal PJ, 1999, PHYSIOTHERAPY, V85, P259

SHROUT PE, 1979, PSYCHOL BULL, V86, P420, DOI 10.1037/0033-2909.86.2.420

Upasani VV, 2007, SPINE, V32, P1355, DOI 10.1097/BRS.0b013e318059321d

Voda Sandra C, 2009, Nursing, V39, P42, DOI 10.1097/01.NURSE.0000365025.40773.4c

WEINSTEIN SL, 1981, J BONE JOINT SURG AM, V63, P702, DOI 10.2106/00004623-198163050-00003

Weinstein SL, 2003, JAMA-J AM MED ASSOC, V289, P559, DOI 10.1001/jama.289.5.559

Weiss H-R, 2006, Pediatr Rehabil, V9, P190, DOI 10.1080/13638490500079583

Weiss Hans-Rudolf, 2003, Pediatr Rehabil, V6, P23, DOI 10.1080/1363849031000095288

NR 28

TC 25

Z9 27

U1 0

U2 9

PU IOS PRESS

PI AMSTERDAM

PA NIEUWE HEMWEG 6B, 1013 BG AMSTERDAM, NETHERLANDS

SN 1053-8127

EI 1878-6324

J9 J BACK MUSCULOSKELET

JI J. Back Musculoskelet. Rehabil.

PY 2014

VL 27

IS 3

BP 331

EP 338

DI 10.3233/BMR-130452

PG 8

WC Orthopedics; Rehabilitation

WE Science Citation Index Expanded (SCI-EXPANDED)

SC Orthopedics; Rehabilitation

GA AN6NG

UT WOS:000340710800009

PM 24361823

DA 2023-08-10

ER

PT J

AU Plaszewski, M

Cieslinski, I

Nowobilski, R

Kotwicki, T

Terech, J

Furgal, M

AF Plaszewski, Maciej

Cieslinski, Igor

Nowobilski, Roman

Kotwicki, Tomasz

Terech, Jacek

Furgal, Mariusz

TI Mental Health of Adults Treated in Adolescence with Scoliosis-Specific

Exercise Program or Observed for Idiopathic Scoliosis

SO SCIENTIFIC WORLD JOURNAL

LA English

DT Article

ID QUALITY-OF-LIFE; BECK DEPRESSION INVENTORY; PHYSICAL-ACTIVITY;

BODY-IMAGE; BRACE; THERAPY

AB Objective. To examine general mental health in adult males and females, who in adolescence participated in a scoliosis-specific therapeutic exercise program or were under observation due to diagnosis of scoliosis. Design. Registry-based, cross-sectional study with retrospective data collection. Methods. Sixty-eight subjects (43 women) aged 30.10 (25-39) years, with mild or moderate scoliosis (11-36 degrees Cobb angle), and 76 (38 women) nonscoliotic subjects, aged 30.11 (24-38) years, participated. The time period since the end of the exercise or observation regimes was 16.5 (12-26) years. Beck Depression Inventory (BDI) and General Health Questionnaire (GHQ-28) scores were analyzed with the chi(2) and U tests. Multiple regression analyses for confounders were also performed. Results. Intergroup differences of demographic characteristics were nonsignificant. Scoliosis, gender, participation in the exercise program, employment, and marital status were

associated with BDI scores. The presence of scoliosis and participation in the exercise program manifested association with the symptoms. Higher GHQ-28 "somatic symptoms" subscale scores interacted with the education level. Conclusions. Our findings correspond to the reports of a negative impact of the diagnosis of scoliosis and treatment on mental health. The decision to introduce a therapeutic program in children with mild deformities should be made with judgment of potential benefits, risks, and harm.

C1 [Plaszewski, Maciej; Cieslinski, Igor] Warsaw Univ, Sch Phys Educ, Inst Physiotherapy, Fac Phys Educ Biala Podlaska, PL-21500 Biala Podlaska, Poland.

[Nowobilski, Roman] Jagiellonian Univ, Inst Physiotherapy, Fac Hlth Sci, PL-31126 Krakow, Poland.

[Kotwicki, Tomasz] Poznan Univ Med Sci, Dept Pediat Orthoped & Traumatol, PL-61545 Poznan, Poland.

[Terech, Jacek] Ctr Pulmonol & Thorac Surg, PL-43360 Bystra, Poland.

[Furgal, Mariusz] Jagiellonian Univ, Coll Med, Dept Psychiat, PL-31501 Krakow, Poland.

C3 4EU+; University of Warsaw; Jozef Pilsudski University Physical

Education in Warsaw; 1EUROPE; Jagiellonian University; Poznan University

of Medical Sciences; 1EUROPE; Jagiellonian University; Collegium Medicum

Jagiellonian University

RP Plaszewski, M (通讯作者)， Warsaw Univ, Sch Phys Educ, Inst Physiotherapy, Fac Phys Educ Biala Podlaska, Akad 2, PL-21500 Biala Podlaska, Poland.

EM plaszewski@wp.pl

OI Cieslinski, Igor/0000-0001-8672-9334

FU Ministry of Science and Higher Education, Poland [DS.136]

FX This paper is a part of the Research Project DS.136, University School

of Physical Education, Warsaw, sponsored by the Ministry of Science and

Higher Education, Poland. The authors thank Doctor Ryszard Batycki for

his involvement in interrater orthopedic examinations.

CR Aebi M, 2005, EUR SPINE J, V14, P925, DOI 10.1007/s00586-005-1053-9

[Anonymous], 2009, HDB PSYCHOL ASSESSME

Asher MA, 2006, SCOLIOSIS SPINAL DIS, V1, DOI 10.1186/1748-7161-1-2

Beck AT, 1996, J PERS ASSESS, V67, P588, DOI 10.1207/s15327752jpa6703_13

Bridwell KH, 2000, SPINE, V25, P2392, DOI 10.1097/00007632-200009150-00020

Carek PJ, 2011, INT J PSYCHIAT MED, V41, P15, DOI 10.2190/PM.41.1.c

Danielsson AJ, 2012, SPINE, V37, P755, DOI 10.1097/BRS.0b013e318231493c

Danielsson AJ, 2001, EUR SPINE J, V10, P278, DOI 10.1007/s005860100309

de Riddler D., 2004, HLTH PSYCHOL

Edwards P, 2002, BRIT MED J, V324, P1183, DOI 10.1136/bmj.324.7347.1183

Goldberg D, 2001, ASSESSMENT MENTAL HL

GOLDBERG MS, 1994, SPINE, V19, P1562, DOI 10.1097/00007632-199407001-00004

Gotze C, 2002, SPINE, V27, P1456, DOI 10.1097/00007632-200207010-00016

Green BN, 2009, J CHIROPR MED, V8, P25, DOI 10.1016/j.jcm.2008.11.001

Greiner KA, 2002, AM FAM PHYSICIAN, V65, P1817

Helenius I, 2005, SPINE, V30, P462, DOI 10.1097/01.brs.0000153347.11559.de

Helman C, 2007, CULTURE HLTH ILLNESS

Jackman S., 2001, PSCL CLASSES METHODS

Jackson C, 2007, OCCUP MED-OXFORD, V57, P79, DOI 10.1093/occmed/kql169

Kebaish KM, 2011, SPINE, V36, P731, DOI 10.1097/BRS.0b013e3181e9f120

Kessler RC, 2003, JAMA-J AM MED ASSOC, V289, P3095, DOI 10.1001/jama.289.23.3095

Leplege A, 1997, JAMA-J AM MED ASSOC, V278, P47, DOI 10.1001/jama.278.1.47

Matsunaga S, 2005, SPINE, V30, P547, DOI 10.1097/01.brs.0000154648.53535.52

Mordecai SC, 2012, EUR SPINE J, V21, P382, DOI 10.1007/s00586-011-2063-4

Noonan KJ, 1997, J PEDIATR ORTHOPED, V17, P712, DOI 10.1097/00004694-199711000-00004

Puzynski S, 2002, PSYCHIATRIA, P453

Reichel Dagmar, 2003, Pediatr Rehabil, V6, P221

Richter P, 1998, PSYCHOPATHOLOGY, V31, P160, DOI 10.1159/000066239

Robinson JP, 1991, MEASURES PERSONALITY, P201

Romano M, 2012, COCHRANE DB SYST REV, DOI 10.1002/14651858.CD007837.pub2

Sapountzi-Krepia DS, 2001, J ADV NURS, V35, P683, DOI 10.1046/j.1365-2648.2001.01900.x

Schwab F, 2005, SPINE, V30, P1082, DOI 10.1097/01.brs.0000160842.43482.cd

Scoliosis and Physical Activity Guideline Committee, 2009, APPR PHYS SPORT ACT

Strohle A, 2009, J NEURAL TRANSM, V116, P777, DOI 10.1007/s00702-008-0092-x

Tones M, 2006, SPINE, V31, P3027, DOI 10.1097/01.brs.0000249555.87601.fc

Vandenbroucke JP, 2007, PLOS MED, V4, P1628, DOI 10.1371/journal.pmed.0040297

Venables W. N., 2008, MODERN APPL STAT S

Weinstein SL, 2003, JAMA-J AM MED ASSOC, V289, P559, DOI 10.1001/jama.289.5.559

Weinstein SL, 2008, LANCET, V371, P1527, DOI 10.1016/S0140-6736(08)60658-3

Zeileis A, 2008, J STAT SOFTW, V27, P1, DOI 10.18637/jss.v027.i08

NR 40

TC 3

Z9 4

U1 0

U2 18

PU HINDAWI LTD

PI LONDON

PA ADAM HOUSE, 3RD FLR, 1 FITZROY SQ, LONDON, W1T 5HF, united kingdom

SN 1537-744X

J9 SCI WORLD J

JI Sci. World J.

PY 2014

AR 932827

DI 10.1155/2014/932827

PG 10

WC Multidisciplinary Sciences

WE Science Citation Index Expanded (SCI-EXPANDED); Social Science Citation Index (SSCI)

SC Science & Technology - Other Topics

GA AA0CZ

UT WOS:000330764000001

PM 24574935

OA Green Published, Green Submitted, gold

DA 2023-08-10

ER

PT J

AU Shakil, H

Iqbal, ZA

Al-Ghadir, AH

AF Shakil, Halima

Iqbal, Zaheen A.

Al-Ghadir, Ahmad H.

TI Scoliosis: Review of types of curves, etiological theories and

conservative treatment

SO JOURNAL OF BACK AND MUSCULOSKELETAL REHABILITATION

LA English

DT Review

DE Scoliosis; etiology; pathology; conservative treatment

ID ADOLESCENT IDIOPATHIC SCOLIOSIS; PHYSICAL EXERCISES; RADIOGRAPHS;

MANAGEMENT; THERAPY; STRESS; SPINE

AB BACKGROUND: Scoliosis is the deviation in the normal vertical spine. Although there are numerous studies available about treatment approaches for scoliosis, the numbers of studies that talk about its etiology and pathology are limited.

OBJECTIVE: Aim of this study was to discuss the different types of scoliosis; its curves and etiological theories; and to note their implication on its treatment.

METHODS: We examined various electronic databases including Pub MED, Medline, Cinhal, Cochrane library and Google scholar using key words "scoliosis", "etiology", "pathology" and "conservative treatment". References of obtained articles were also examined for cross references. The search was limited to articles in English language.

RESULTS: A total of 145 papers, about Prevalence, History, Symptoms, classification, Biomechanics, Pathogenesis, Kinematics and Treatment of scoliosis were identified to be relevant.

CONCLUSION: To choose the appropriate treatment approach for scoliosis we need to understand its etiology and pathogenesis first. Early intervention with conservative treatment like physiotherapy and bracing can prevent surgery.

C1 [Shakil, Halima] Jamia Millia Islamia, Ctr Physiotherapy & Rehabil Sci, New Delhi 110025, India.

[Iqbal, Zaheen A.; Al-Ghadir, Ahmad H.] King Saud Univ, Coll Appl Med Sci, Dept Rehabil Sci, Riyadh 11433, Saudi Arabia.

C3 Jamia Millia Islamia; King Saud University

RP Iqbal, ZA (通讯作者)， King Saud Univ, RRC, POB 10219, Riyadh 11433, Saudi Arabia.

EM z_iqbal001@yahoo.com

RI Alghadir, Ahmad H/D-4422-2015; Alghadir, Ahmad/HKO-9206-2023; Iqbal,

Zaheen/AAN-5738-2020

OI Alghadir, Ahmad/0000-0002-1204-476X; Iqbal, Zaheen/0000-0002-0504-6863

FU Deanship of Scientific Research at King Saud University [RGP-VPP-209]

FX The authors extend their appreciation to the Deanship of Scientific

Research at King Saud University for funding the work through the

research project no. RGP-VPP-209.

CR Anderson SM, 2007, RADIOLOGIC TECHNOLOG, V79, P6

Anderson Susan M, 2007, Radiol Technol, V79, P44

Asher MA, 2006, SCOLIOSIS SPINAL DIS, V1, DOI 10.1186/1748-7161-1-2

BRIDWELL KH, 1994, SPINE, V19, P1095, DOI 10.1097/00007632-199405000-00020

CARMAN DL, 1990, J BONE JOINT SURG AM, V72A, P328, DOI 10.2106/00004623-199072030-00003

CASSELLA MC, 1991, PHYS THER, V71, P897, DOI 10.1093/ptj/71.12.897

Chen ZQ, 2012, CHINESE MED J-PEKING, V125, P1439, DOI 10.3760/cma.j.issn.0366-6999.2012.08.015

Czupryna Krzysztof, 2012, Ortop Traumatol Rehabil, V14, P103, DOI 10.5604/15093492.992293

de Seze M., 2012, Annals of Physical and Rehabilitation Medicine, V55, P128, DOI 10.1016/j.rehab.2012.01.003

DICKSON RA, 1985, J BONE JOINT SURG BR, V67, P176, DOI 10.1302/0301-620X.67B2.3872301

FARADY JA, 1983, PHYS THER, V63, P512, DOI 10.1093/ptj/63.4.512

Fletcher ND, 2012, CURR REV MUSCULOSKE, V5, P102, DOI 10.1007/s12178-012-9116-0

Fusco C., 2011, Physiotherapy Theory and Practice, V27, P80, DOI 10.3109/09593985.2010.533342

Glassman SD, 2005, SPINE, V30, P682, DOI 10.1097/01.brs.0000155425.04536.f7

Goldberg C, 2006, ST HEAL T, V123, P442

Harrison DE, 2002, J MANIP PHYSIOL THER, V25, P93, DOI 10.1067/mmt.2002.121411

JEFFERSON RJ, 1988, J BONE JOINT SURG BR, V70, P261, DOI 10.1302/0301-620X.70B2.3346300

Kinel E, 2012, STUD HEALTH TECHNOL, V176, P419, DOI 10.3233/978-1-61499-067-3-419

Knoeller SM, 2002, SAUDI MED J, V23, P1181

LOYNES R D, 1972, Journal of Bone and Joint Surgery British Volume, V54B, P484

Misterska E, 2012, SPINE, V37, P1218, DOI 10.1097/BRS.0b013e31824b66d4

MORRISSY RT, 1990, J BONE JOINT SURG AM, V72A, P320, DOI 10.2106/00004623-199072030-00002

Negrini S, 2008, DISABIL REHABIL, V30, P772, DOI 10.1080/09638280801889568

Negrini Stefano, 2003, Pediatr Rehabil, V6, P227, DOI: 10.1080/13638490310001636781

Negrini S, 2012, STUD HEALTH TECHNOL, V176, P354, DOI 10.3233/978-1-61499-067-3-354

ODA M, 1982, J PEDIATR ORTHOPED, V2, P378, DOI 10.1097/01241398-198210000-00005

PEDRINI VA, 1973, J LAB CLIN MED, V82, P938

ROAF ROBERT, 1966, J BONE JOINT SURG B BRIT, V48, P786

Sarnadskiy VN, 2012, STUD HEALTH TECHNOL, V176, P159, DOI 10.3233/978-1-61499-067-3-159

Sevastik B, 1997, Eur Spine J, V6, P84, DOI 10.1007/BF01358737

Sharma S, 2011, HUM MOL GENET, V20, P1456, DOI 10.1093/hmg/ddq571

Stokes IA, 2006, SCOLIOSIS, V18, P1

Weinstein S L, 1989, Instr Course Lect, V38, P115

Weiss HR, 2008, EUR J PHYS REHAB MED, V44, P177

Weiss HR, 2006, SCOLIOSIS SPINAL DIS, V1, DOI 10.1186/1748-7161-1-6

Weiss HR, 2012, SCOLIOSIS SPINAL DIS, V7, DOI 10.1186/1748-7161-7-4

Weiss HR, 2003, ORTHOPADE, V32, P146, DOI 10.1007/s00132-002-0430-x

WHITE AA, 1971, J BIOMECH, V4, P405, DOI 10.1016/0021-9290(71)90060-1

WHITE AA, 1976, CLIN ORTHOP RELAT R, P100

Zhou Chunguang, 2010, Zhongguo Xiu Fu Chong Jian Wai Ke Za Zhi, V24, P23

NR 40

TC 26

Z9 32

U1 0

U2 43

PU IOS PRESS

PI AMSTERDAM

PA NIEUWE HEMWEG 6B, 1013 BG AMSTERDAM, NETHERLANDS

SN 1053-8127

EI 1878-6324

J9 J BACK MUSCULOSKELET

JI J. Back Musculoskelet. Rehabil.

PY 2014

VL 27

IS 2

BP 111

EP 115

DI 10.3233/BMR-130438

PG 5

WC Orthopedics; Rehabilitation

WE Science Citation Index Expanded (SCI-EXPANDED)

SC Orthopedics; Rehabilitation

GA AH9JN

UT WOS:000336458300001

PM 24284269

DA 2023-08-10

ER

PT J

AU Stokes, OM

Luk, KDK

AF Stokes, O. M.

Luk, K. D. K.

TI The current status of bracing for patients with adolescent idiopathic

scoliosis

SO BONE & JOINT JOURNAL

LA English

DT Review

ID TERM-FOLLOW-UP; TECHNOLOGY THEMATIC SERIES; CHARLESTON BENDING BRACE;

CURVE PROGRESSION; NATURAL-HISTORY; MILWAUKEE BRACE; CONSERVATIVE

TREATMENT; OPERATIVE TREATMENT; SFORZESCO BRACE; CAD/CAM METHOD

AB Adolescent idiopathic scoliosis affects about 3% of children. Non-operative measures are aimed at altering the natural history to maintain the size of the curve below 40 degrees at skeletal maturity. The application of braces to treat spinal deformity pre-dates the era of evidence-based medicine, and there is a paucity of irrefutable prospective evidence in the literature to support their use and their effectiveness has been questioned.

This review considers this evidence. The weight of the evidence is in favour of bracing over observation. The most recent literature has moved away from addressing this question, and instead focuses on developments in the design of braces and ways to improve compliance.

C1 [Stokes, O. M.; Luk, K. D. K.] Univ Hong Kong, Queen Mary Hosp, Div Spine Surg, Dept Orthopaed & Traumatol, Pokfulam, Hong Kong, china.

C3 University of Hong Kong

RP Luk, KDK (通讯作者)， Univ Hong Kong, Queen Mary Hosp, Div Spine Surg, Dept Orthopaed & Traumatol, Pokfulam, Hong Kong, china.

EM hrmoldk@hku.hk

CR ADAIR IV, 1977, CLIN ORTHOP RELAT R, P165

Angevine PD, 2008, NEUROSURGERY, V63, pA86, DOI 10.1227/01.NEU.0000320427.28377.F7

[Anonymous], SRS 30 PAT QUEST

ASCANI E, 1986, SPINE, V11, P784, DOI 10.1097/00007632-198610000-00007

Athanasopoulos S, 1999, SCAND J MED SCI SPOR, V9, P36

Benish BM, 2012, SPINE, V37, P309, DOI 10.1097/BRS.0b013e31821e1488

BLOUNT W, 1973, MILWAUKEE BRACE

BLOUNT WP, 1958, J BONE JOINT SURG AM, V40, P511, DOI 10.2106/00004623-195840030-00003

BROOKS HL, 1975, J BONE JOINT SURG AM, V57, P968, DOI 10.2106/00004623-197557070-00015

Bunge EM, 2010, EUR SPINE J, V19, P747, DOI 10.1007/s00586-010-1337-6

Bunge EM, 2008, BMC MUSCULOSKEL DIS, V9, DOI 10.1186/1471-2474-9-57

BUNNELL WP, 1980, J BONE JOINT SURG AM, V62, P31, DOI 10.2106/00004623-198062010-00005

CARMAN DL, 1990, J BONE JOINT SURG AM, V72A, P328, DOI 10.2106/00004623-199072030-00003

Castro Frank P Jr, 2003, Spine J, V3, P180, DOI 10.1016/S1529-9430(02)00557-0

Chalmers E, 2012, IEEE T NEUR SYS REH, V20, P557, DOI 10.1109/TNSRE.2012.2192483

Chan A, 2012, MED ENG PHYS, V34, P1310, DOI 10.1016/j.medengphy.2011.12.022

Cheneau J., 1994, CORSET CHENEAU MANUE

Clin J, 2010, EUR SPINE J, V19, P1169, DOI 10.1007/s00586-009-1268-2

Cobb JR., 1948, INSTR COURSE LECT, V5, P261

Cottalorda J, 2005, SPINE, V30, P399, DOI 10.1097/01.brs.0000153346.40391.3b

DAMATOC, 2001, PROVIDENCE SCOLIOSIS

Danielsson AJ, 2007, SPINE, V32, P2198, DOI 10.1097/BRS.0b013e31814b851f

Danielsson AJ, 2001, SPINE, V26, P516

Danielsson AJ, 2006, SPINE, V31, P275, DOI 10.1097/01.brs.0000197652.52890.71

de Mauroy JC, 2011, SCOLIOSIS SPINAL DIS, V6, DOI 10.1186/1748-7161-6-4

den Boer WA, 1999, EUR SPINE J, V8, P406, DOI 10.1007/s005860050195

DESMET AA, 1984, SPINE, V9, P377, DOI 10.1097/00007632-198405000-00009

Dickson RA, 1999, J BONE JOINT SURG BR, V81B, P193, DOI 10.1302/0301-620X.81B2.9630

Dolan LA, 2007, SPINE, V32, pS91, DOI 10.1097/BRS.0b013e318134ead9

Dolan LA, 2008, J BONE JOINT SURG AM, V90A, P2594, DOI 10.2106/JBJS.G.01460

DURHAM JW, 1990, SPINE, V15, P888, DOI 10.1097/00007632-199009000-00010

EDGAR MA, 1988, J BONE JOINT SURG BR, V70, P712, DOI 10.1302/0301-620X.70B5.3192566

el-Sayyad M, 1994, Int J Rehabil Res, V17, P70, DOI 10.1097/00004356-199403000-00008

FERNANDEZFELIBERTI R, 1995, J PEDIATR ORTHOPED, V15, P176

Goldberg CJ, 2001, SPINE, V26, P42, DOI 10.1097/00007632-200101010-00009

GOLDBERG CJ, 1993, SPINE, V18, P902, DOI 10.1097/00007632-199306000-00015

GOLDBERG MS, 1994, SPINE, V19, P1562, DOI 10.1097/00007632-199407001-00004

Grivas TB, 2010, STUD HEALTH TECHNOL, V158, P157, DOI 10.3233/978-1-60750-573-0-157

GROSS C, 1983, B HOSP JOINT DIS ORT, V43, P171

HOOPER RC, 2013, CHARLESTON BENDING B

JAMES JIP, 1954, J BONE JOINT SURG BR, V36, P36, DOI 10.1302/0301-620X.36B1.36

Janicki JA, 2007, J PEDIATR ORTHOPED, V27, P369, DOI 10.1097/01.bpb.0000271331.71857.9a

KAHANOVITZ N, 1986, SPINE, V11, P753, DOI 10.1097/00007632-198609000-00017

KAHANOVITZ N, 1989, SPINE, V14, P483, DOI 10.1097/00007632-198905000-00001

Karol LA, 2001, SPINE, V26, P2001, DOI 10.1097/00007632-200109150-00013

Katz DE, 2001, SPINE, V26, P2354, DOI 10.1097/00007632-200111010-00012

Katz DE, 1997, SPINE, V22, P1302, DOI 10.1097/00007632-199706150-00005

Katz DE, 2010, J BONE JOINT SURG AM, V92A, P1343, DOI 10.2106/JBJS.I.01142

Kunz R, 1998, BRIT MED J, V317, P1185, DOI 10.1136/bmj.317.7167.1185

Labelle H, 2007, SPINE, V32, P835, DOI 10.1097/01.brs.0000259811.58372.87

Lenssinck MLB, 2005, PHYS THER, V85, P1329, DOI 10.1093/ptj/85.12.1329

LONSTEIN JE, 1984, J BONE JOINT SURG AM, V66A, P1061, DOI 10.2106/00004623-198466070-00013

LONSTEIN JE, 1994, J BONE JOINT SURG AM, V76A, P1207, DOI 10.2106/00004623-199408000-00011

Lou E, 2011, MED ENG PHYS, V33, P290, DOI 10.1016/j.medengphy.2010.10.016

Lovett RW, 1924, J BONE JOINT SURG, V6, P847

Luk KDK, 2010, SPINE, V35, P1607, DOI 10.1097/BRS.0b013e3181c7cb8c

Maruyama Toru, 2011, Physiotherapy Theory and Practice, V27, P26, DOI 10.3109/09593985.2010.503989

MAYO NE, 1994, SPINE, V19, P1573, DOI 10.1097/00007632-199407001-00005

Mehta MH., 1985, SCOLIOSIS PREVENTION, P126

MILLER JAA, 1984, SPINE, V9, P632, DOI 10.1097/00007632-198409000-00015

Miller NH, 2007, CLIN ORTHOP RELAT R, P6, DOI 10.1097/BLO.0b013e318126c062

MOE JH, 1971, CLIN ORTHOP RELAT R, P18

MORRISSY RT, 1990, J BONE JOINT SURG AM, V72A, P320, DOI 10.2106/00004623-199072030-00002

Muller C, 2011, EUR SPINE J, V20, P1127, DOI 10.1007/s00586-011-1791-9

NACHEMSON A, 1968, ACTA ORTHOP SCAND, V39, P466, DOI 10.3109/17453676808989664

NACHEMSON A, 1982, ANN M SCOL RES SOC C

NACHEMSON AL, 1995, J BONE JOINT SURG AM, V77A, P815, DOI 10.2106/00004623-199506000-00001

Negrini S, 2007, Eura Medicophys, V43, P171

Negrini S, 2006, STUD HEALTH TECHNOL, V123, P245

Negrini S, 2012, SCOLIOSIS SPINAL DIS, V7, DOI 10.1186/1748-7161-7-3

Negrini S, 2012, STUD HEALTH TECHNOL, V176, P437, DOI 10.3233/978-1-61499-067-3-437

Negrini S, 2011, SCOLIOSIS SPINAL DIS, V6, DOI 10.1186/1748-7161-6-8

Negrini S, 2010, SPINE, V35, P1285, DOI 10.1097/BRS.0b013e3181dc48f4

Negrini S, 2008, SCOLIOSIS SPINAL DIS, V3, DOI 10.1186/1748-7161-3-15

Negrini S, 2009, SCOLIOSIS SPINAL DIS, V4, DOI 10.1186/1748-7161-4-2

NEWTON PO, 2006, LOVELL WINTERS PEDIA, P693

Noonan KJ, 1997, J PEDIATR ORTHOPED, V17, P712, DOI 10.1097/00004694-199711000-00004

Noonan KJ, 1996, J BONE JOINT SURG AM, V78A, P557, DOI 10.2106/00004623-199604000-00009

O'Neill PJ, 2005, J BONE JOINT SURG AM, V87A, P1069, DOI 10.2106/JBJS.C.01707

Odgaard-Jensen J, 2011, COCHRANE DB SYST REV, DOI 10.1002/14651858.MR000012.pub3

PEHRSSON K, 1991, THORAX, V46, P474, DOI 10.1136/thx.46.7.474

PEHRSSON K, 1992, SPINE, V17, P1091, DOI 10.1097/00007632-199209000-00014

Pehrsson K, 2001, THORAX, V56, P388, DOI 10.1136/thorax.56.5.388

Pham V. M., 2008, Annales de Readaptation et de Medecine Physique, V51, P3, DOI 10.1016/j.annrmp.2007.08.008

Phan P, 2011, EUR SPINE J, V20, P1058, DOI 10.1007/s00586-011-1699-4

Price CT, 1997, J PEDIATR ORTHOPED, V17, P703, DOI 10.1097/00004694-199711000-00002

PRUIJS JEH, 1994, SKELETAL RADIOL, V23, P517, DOI 10.1007/BF00223081

Richards BS, 2005, SPINE, V30, P2068, DOI 10.1097/01.brs.0000178819.90239.d0

RISSER J C, 1955, Instr Course Lect, V12, P255

RISSER J C, 1958, Clin Orthop, V11, P111

RIVARD CH, 2002, EUR SPINE J S, V11, P14

RIVARD CH, SPINECOR SYSTEM

Roberts DW, 2011, SPINE, V36, pE53, DOI 10.1097/BRS.0b013e3181ef9efc

ROGALA EJ, 1978, J BONE JOINT SURG AM, V60, P173, DOI 10.2106/00004623-197860020-00005

Romano M, 2012, COCHRANE DB SYST REV, DOI 10.1002/14651858.CD007837.pub2

Rowe DE, 1997, J BONE JOINT SURG AM, V79A, P664, DOI 10.2106/00004623-199705000-00005

Sanders James O, 2012, J Pediatr Orthop, V32 Suppl 2, pS153, DOI 10.1097/BPO.0b013e31825199e5

Schiller JR, 2010, CLIN ORTHOP RELAT R, V468, P670, DOI 10.1007/s11999-009-0884-9

*SCOL RES SOC, SRS 30 PAT QUEST SCO

STOKES IAF, 1987, J ORTHOP RES, V5, P102, DOI 10.1002/jor.1100050113

Tavernaro M, 2012, SCOLIOSIS SPINAL DIS, V7, DOI 10.1186/1748-7161-7-17

Ugwonali Obinwanne F, 2004, Spine J, V4, P254

van Tulder M, 2003, SPINE, V28, P1290, DOI 10.1097/00007632-200306150-00014

Veldhuizen AG, 2002, MED ENG PHYS, V24, P209, DOI 10.1016/S1350-4533(02)00008-5

VELDHUIZEN AG, 1985, THESIS U GRONINGEN G

Wang WJ, 2012, SPINE, V37, P1586, DOI 10.1097/BRS.0b013e3182511d0c

WATTS HG, 1977, CLIN ORTHOP RELAT R, P87

WEINSTEIN SL, 1983, J BONE JOINT SURG AM, V65, P447, DOI 10.2106/00004623-198365040-00004

WEINSTEIN SL, 1981, J BONE JOINT SURG AM, V63, P702, DOI 10.2106/00004623-198163050-00003

Weinstein SL, 2003, JAMA-J AM MED ASSOC, V289, P559, DOI 10.1001/jama.289.5.559

Weiss HR, 2008, EUR J PHYS REHAB MED, V44, P177

Weiss Hans-Rudolf, 2005, Pediatr Rehabil, V8, P199

Weiss Hans-Rudolf, 2003, Pediatr Rehabil, V6, P23, DOI 10.1080/1363849031000095288

Weiss HR, 2012, STUD HEALTH TECHNOL, V176, P407, DOI 10.3233/978-1-61499-067-3-407

Weiss HR, 2010, SCOLIOSIS SPINAL DIS, V5, DOI 10.1186/1748-7161-5-19

WILLNER S, 1982, ACTA ORTHOP SCAND, V53, P233, DOI 10.3109/17453678208992208

WINTER RB, 1994, ORTHOP CLIN N AM, V25, P195

Wong MS, 2008, SPINE, V33, P1360, DOI 10.1097/BRS.0b013e31817329d9

Wong MS, 2005, PROSTHET ORTHOT INT, V29, P93, DOI 10.1080/17461550500066782

Wong MS, 2005, PROSTHET ORTHOT INT, V29, P105, DOI 10.1080/17461550500069547

Xu LL, 2011, EUR SPINE J, V20, P1757, DOI 10.1007/s00586-011-1874-7

Yrjonen T, 2006, EUR SPINE J, V15, P1139, DOI 10.1007/s00586-005-0049-9

SCOLIOSIS RES SOC BR

SRS TERMINOLOGY COMM

NR 124

TC 15

Z9 16

U1 0

U2 23

PU BRITISH EDITORIAL SOC BONE JOINT SURGERY

PI LONDON

PA 22 BUCKINGHAM STREET, LONDON WC2N 6ET, united kingdom

SN 2049-4394

J9 BONE JOINT J

JI Bone Joint J.

PD OCT

PY 2013

VL 95B

IS 10

BP 1308

EP 1316

DI 10.1302/0301-620X.95B10.31474

PG 9

WC Orthopedics; Surgery

WE Science Citation Index Expanded (SCI-EXPANDED)

SC Orthopedics; Surgery

GA 225RK

UT WOS:000324979800003

PM 24078524

DA 2023-08-10

ER

PT J

AU Lee, M

Hwang, J

Seo, B

Im, G

Jeon, N

Yang, H

Kim, T

Kim, H

Han, D

AF Lee, Myounggi

Hwang, Jinseok

Seo, Byungdo

Im, Gyudon

Jeon, Nakyu

Yang, Hyunho

Kim, Taeho

Kim, Hyungguen

Han, Dongwook

TI The Effects of the Core Muscle Release Technique on Scoliosis

SO JOURNAL OF PHYSICAL THERAPY SCIENCE

LA English

DT Article

DE Core muscle release technique; Scoliosis; Cobb angle

ID ADOLESCENT IDIOPATHIC SCOLIOSIS; EXERCISES

AB [Purpose] The object of this study was to examine the effects of the core muscle release technique (CRT) on correcting scoliosis. [Subjects] Ninety patients diagnosed with scoliosis participated in this study. [Methods] First, participants were divided into three groups according to method of treatment. The first group was administered 50 minutes of CRT five times a week for two weeks. The second group performed general exercise to treat scoliosis for the same duration. The third group received electrotherapy in 50-minute sessions, five times a week for two weeks. The CRT began with release treatment of the diaphragm, and then focused on control of paraspinal muscle tone. Respiratory therapy was also conducted during these treatments. Directly after their two-week treatment, the patients were re-examined, and another follow-up test was performed two weeks after treatment had finished. [Results] The data suggest that CRT, exercise and electrotherapy all helped to decrease the Cobb angle. Of these treatment methods, however, CRT was the most effective for the treatment of scoliosis. [Conclusion] CRT was effective in the correction of scoliosis.

C1 [Kim, Hyungguen] Hallym Univ, Dept Phys Therapy, Fac Hlth Sci, Chunchon, South Korea.

[Han, Dongwook] Silla Univ, Dept Phys Therapy, Coll Med & Life Sci, Pusan 617736, South Korea.

C3 Hallym University; Silla University

RP Han, D (通讯作者)， Silla Univ, Dept Phys Therapy, Coll Med & Life Sci, 700 Beon Gi,140 Baegyang Daero, Pusan 617736, South Korea.

EM dwhan@silla.ac.kr

CR Akuthota V, 2004, ARCH PHYS MED REHAB, V85, pS86, DOI 10.1053/j.apmr.2003.12.005

Akuthota V, 2008, CURR SPORT MED REP, V7, P39, DOI 10.1097/01.CSMR.0000308663.13278.69

BUNNELL WP, 1986, SPINE, V11, P773, DOI 10.1097/00007632-198610000-00003

Canavese F, 2011, INDIAN J ORTHOP, V45, P7, DOI 10.4103/0019-5413.73655

Chang Myoung-Jei, 2010, [Journal of adapted physical activity and exercise, 한국특수체육학회지], V18, P117

Chuah S L, 2001, Med J Malaysia, V56 Suppl C, P37

Fredericson Michael, 2005, Phys Med Rehabil Clin N Am, V16, P669, DOI 10.1016/j.pmr.2005.03.001

Fusco C., 2011, Physiotherapy Theory and Practice, V27, P80, DOI 10.3109/09593985.2010.533342

Harrison DE, 1999, J MANIP PHYSIOL THER, V22, P399, DOI 10.1016/S0161-4754(99)70086-2

Hides J, 2006, SPINE, V31, pE175, DOI 10.1097/01.brs.0000202740.86338.df

Hodges P, 1996, Physiother Res Int, V1, P30, DOI 10.1002/pri.45

Koumantakis GA, 2005, CLIN BIOMECH, V20, P474, DOI 10.1016/j.clinbiomech.2004.12.006

Lantz CA, 2001, J MANIP PHYSIOL THER, V24, P385, DOI 10.1067/mmt.2001.116419

이상기, 2010, [The Korean Society of Sports Science, 한국체육과학회지], V19, P1263

Marshall PW, 2005, ARCH PHYS MED REHAB, V86, P242, DOI 10.1016/j.apmr.2004.05.004

Negrini Stefano, 2003, Pediatr Rehabil, V6, P227, DOI: 10.1080/13638490310001636781

Richardson C A, 1995, Man Ther, V1, P2, DOI 10.1054/math.1995.0243

Romano M, 2008, SCOLIOSIS SPINAL DIS, V3, DOI 10.1186/1748-7161-3-2

Sapsford R, 2000, NEUROUROL URODYNAM, V19, P633, DOI 10.1002/1520-6777(2000)19:5<633::AID-NAU9>3.0.CO;2-Q

Stokes IA, 2003, WIRBELSAULEN DEFORMI

Vasiliadis E, 2009, STUD HEALTH TECHNOL, V135, P409

Wimmer C., 2003, MED ORTHOP TECH, V123, P33

Winter RB, 1995, TXB SCOLIOSIS OTHER

Zaborowska-Sapeta K, 2011, SCOLIOSIS SPINAL DIS, V6, DOI 10.1186/1748-7161-6-2

NR 24

TC 11

Z9 11

U1 0

U2 25

PU SOC PHYSICAL THERAPY SCIENCE

PI TOKYO

PA C/O PUBLICATION CENTER, 1-24-12 SUGAMO, TOSHIMA-KU, TOKYO, 170-0002,

JAPAN

SN 0915-5287

EI 2187-5626

J9 J PHYS THER SCI

JI J. Phys. Ther. Sci.

PD APR

PY 2013

VL 25

IS 4

BP 445

EP 448

DI 10.1589/jpts.25.445

PG 4

WC Rehabilitation

WE Science Citation Index Expanded (SCI-EXPANDED)

SC Rehabilitation

GA 191LW

UT WOS:000322415500019

OA Bronze

DA 2023-08-10

ER

PT J

AU Zakaria, A

Hafez, AR

Buragadda, S

Melam, GR

AF Zakaria, Abdulrahim

Hafez, Ashraf Ramadan

Buragadda, Syamala

Melam, Ganeswara Rao

TI Stretching Versus Mechanical Traction of the Spine in Treatment of

Idiopathic Scoliosis

SO JOURNAL OF PHYSICAL THERAPY SCIENCE

LA English

DT Article

DE Stretching exercises; Idiopathic scoliosis; Spinal deformity

ID LOW-BACK-PAIN; LUMBAR TRACTION; BODY; MANAGEMENT; DEFORMITY; POSTURE

AB [Purpose] Traction-based therapies are non-invasive and probably cost-effective and have received interest recently. This study was conducted to compare two training programs, traction and stretching exercises, in rehabilitation of moderate scoliotic patients. [Subjects] Forty patients who were 15 to 25 years of age and had moderate scoliosis (Cobb's angle of 20 to 40 degrees) were randomized to either a stretching exercises group (n=20) or mechanical traction group (n=20). [Methods] All the patients were informed about the testing and training procedure and were allocated randomly into two groups. Both the groups received a common physical therapy program of 3 sessions a week for 3 months. In addition, the stretching exercises group received stretching of muscles on the concave side with postural instructions for activities of daily living. The mechanical traction group received mechanical traction of the lumbar spine with postural instructions for activities of daily living. The outcome measures used were anteroposterior view of loading X-ray to detect any change in the Cobb's angle of the lower spine, tape measurement to detect forward flexion of the trunk by using fingertip-to-floor test (FFT), and the visual analogue scale (VAS) for pain measurement. [Results] There was more significant improvement of the Cobb's angle in the stretching exercises group than in the mechanical traction group. There was no significant difference in VAS and FFT values between the groups. [Conclusion] Stretching exercises led to significant improvement in the Cobb's angle and resulted in improvement of scoliotic curves in moderate scoliotic patients.

C1 [Zakaria, Abdulrahim; Hafez, Ashraf Ramadan; Buragadda, Syamala; Melam, Ganeswara Rao] King Saud Univ, Coll Appl Med Sci, Dept Rehabil Sci, Riyadh 11433, Saudi Arabia.

C3 King Saud University

RP Zakaria, A (通讯作者)，King Saud Univ, Coll Appl Med Sci, Dept Rehabil Sci, POB 10219, Riyadh 11433, Saudi Arabia.

EM syamala3110@yahoo.co.in

RI MELAM, GANESWARA RAO/AAF-9409-2020; Buragadda, Syamala/AAC-2530-2019

OI MELAM, GANESWARA RAO/0000-0002-6997-0668; Buragadda,

Syamala/0000-0003-2481-1214; Hafez, Ashraf/0000-0001-7529-9236

CR Beattie PF, 2008, ARCH PHYS MED REHAB, V89, P269, DOI 10.1016/j.apmr.2007.06.778

Burwell R G, 2003, Pediatr Rehabil, V6, P137

Clarke J, 2006, SPINE, V31, P1591, DOI 10.1097/01.brs.0000222043.09835.72

COBB JR, 1960, J BONE JOINT SURG AM, V42, P1413, DOI 10.2106/00004623-196042080-00012

Freidel K, 2002, SPINE, V27, pE87, DOI 10.1097/00007632-200202150-00013

Fusco C., 2011, Physiotherapy Theory and Practice, V27, P80, DOI 10.3109/09593985.2010.533342

Gay RE, 2008, SPINE J, V8, P234, DOI 10.1016/j.spinee.2007.10.025

GOLDBERG MS, 1994, SPINE, V19, P1562, DOI 10.1097/00007632-199407001-00004

JAMES JIP, 1954, J BONE JOINT SURG BR, V36, P36, DOI 10.1302/0301-620X.36B1.36

Koutedakis Y, 1997, INT J SPORTS MED, V18, P290, DOI 10.1055/s-2007-972636

Li YC, 1996, PHYS THER, V76, P836, DOI 10.1093/ptj/76.8.836

Macario Alex, 2008, Pain Pract, V8, P11, DOI 10.1111/j.1533-2500.2007.00167.x

MCCLURE D, 2006, EUR MUSCULOSKELETAL, V1, P45

Nault ML, 2002, SPINE, V27, P1911, DOI 10.1097/00007632-200209010-00018

Negrini S, 2007, J SCOLIOSIS, P2

Negrini Stefano, 2003, Pediatr Rehabil, V6, P227, DOI: 10.1080/13638490310001636781

O'Sullivan PB, 1997, SPINE, V22, P2959, DOI 10.1097/00007632-199712150-00020

Rigo M, 2003, Pediatr Rehabil, V6, P209

Rigo Manuel, 2011, Physiotherapy Theory and Practice, V27, P7, DOI 10.3109/09593985.2010.503990

Sato T, 2011, EUR SPINE J, V20, P274, DOI 10.1007/s00586-010-1657-6

Schimmel JJP, 2009, EUR SPINE J, V18, P1843, DOI 10.1007/s00586-009-1044-3

Shealy C.N., 2005, AM J PAIN MANAG, V15, P93

SHEALY CN, 1997, AM J PAIN MANAGE, V7, P663

Soucacos PN, 2000, ORTHOPEDICS, V23, P833

Stirling AJ, 1996, J BONE JOINT SURG AM, V78A, P1330, DOI 10.2106/00004623-199609000-00006

STOKES IAF, 1994, SPINE, V19, P236, DOI 10.1097/00007632-199401001-00020

Stokes IAF, 2006, SCOLIOSIS SPINAL DIS, V1, DOI 10.1186/1748-7161-1-16

WEINSTEIN SL, 1981, J BONE JOINT SURG AM, V63, P702, DOI 10.2106/00004623-198163050-00003

Weiss HR, 2007, SCOLIOSIS SPINAL DIS, V2, DOI 10.1186/1748-7161-2-19

Werners R, 1999, SPINE, V24, P1579, DOI 10.1097/00007632-199908010-00012

Wong HK, 2010, INDIAN J ORTHOP, V44, P9, DOI 10.4103/0019-5413.58601

NR 31

TC 14

Z9 14

U1 0

U2 26

PU SOC PHYSICAL THERAPY SCIENCE

PI TOKYO

PA C/O PUBLICATION CENTER, 1-24-12 SUGAMO, TOSHIMA-KU, TOKYO, 170-0002,

JAPAN

SN 0915-5287

EI 2187-5626

J9 J PHYS THER SCI

JI J. Phys. Ther. Sci.

PD DEC

PY 2012

VL 24

IS 11

BP 1127

EP 1131

DI 10.1589/jpts.24.1127

PG 5

WC Rehabilitation

WE Science Citation Index Expanded (SCI-EXPANDED)

SC Rehabilitation

GA 168CQ

UT WOS:000320683700013

OA Bronze

DA 2023-08-10

ER

PT J

AU Misterska, E

Glowacki, M

Latuszewska, J

AF Misterska, Ewa

Glowacki, Maciej

Latuszewska, Joanna

TI Female Patients' and Parents' Assessment of Deformity- and Brace-Related

Stress in the Conservative Treatment of Adolescent Idiopathic Scoliosis

SO SPINE

LA English

DT Article

DE adolescent idiopathic scoliosis (AIS); spinal deformity; brace

treatment; stress

ID PERCEPTIONS; SURGERY

AB Study Design. A cross-sectional analysis of parents' and patients' perceptions of deformity-and brace-related stress regarding conservative treatment of adolescent idiopathic scoliosis.

Objective. The purpose of this study was to determine the agreement between patients' and parents' assessments of emotional stress and to compare these assessments with radiographical measurements of spinal deformity.

Summary of Background Data. Conservative treatment in patients with scoliosis may cause emotional stress. To our knowledge, no group has ever reported patient and parental estimation of stress related to wearing a brace and spinal deformity in girls with adolescent idiopathic scoliosis.

Methods. Sixty-three pairs of parents and girls with adolescent idiopathic scoliosis treated with a Cheneau brace were separately asked to complete the Bad Sobberheim Stress Questionnaire-Deformity and the Bad Sobberheim Stress Questionnaire-Brace. The age range of the patients was from 10 to 17 years. Patients were assessed at a mean of 14.12 (SD, 10.99) months after the start of the conservative treatment.

Results. Patients thought that a moderate level of stress was connected with conservative treatment; however, the stress level, related to perceived trunk deformation, was low. From the parents' perspective, patients experienced a moderate level of stress during conservative treatment and related to spinal deformity. The study groups differ in their perception of stress levels due to body disfigurement but not during the conservative treatment. Parent-patient stress-level disparities were not related to body mass index, age of the patient, brace application, and radiographical measurements of spinal deformity.

Conclusion. Patients and parents perceive the emotional stress related to brace treatment in the same way; however, parents overestimate the assessment of stress levels related to body deformity. From the perspective of patients and parents, brace wearing increased the level of stress induced by the deformity alone. Complete assessment of conservative treatment should include evaluation of emotional stress from the perspective of patients and parents.

C1 [Misterska, Ewa; Glowacki, Maciej] Poznan Univ Med Sci, Dept Pediat Orthopaed & Traumatol, PL-61545 Poznan, Poland.

[Latuszewska, Joanna] Poznan Univ Phys Educ, Dept Motor Syst Rehabil, Poznan, Poland.

C3 Poznan University of Medical Sciences; Poznan University of Physical

Education

RP Misterska, E (通讯作者)， Poznan Univ Med Sci, Dept Pediat Orthopaed & Traumatol, Ul 26 Czerwca 1956 135-147, PL-61545 Poznan, Poland.

EM emisterska1@wp.pl

RI Misterska, Ewa/N-3429-2013

OI Misterska, Ewa/0000-0001-9726-9214

CR Botens-Helmus C, 2006, SCOLIOSIS SPINAL DIS, V1, DOI 10.1186/1748-7161-1-22

Climent JM, 1999, SPINE, V24, P1903, DOI 10.1097/00007632-199909150-00007

MACLEAN WE, 1989, J PEDIATR ORTHOPED, V9, P257

Matsunaga S, 2005, SPINE, V30, P547, DOI 10.1097/01.brs.0000154648.53535.52

Misterska E, 2009, EUR SPINE J, V18, P1911, DOI 10.1007/s00586-009-1126-2

Pratt RK, 2002, SPINE, V27, P1543, DOI 10.1097/00007632-200207150-00012

Rinella A, 2004, SPINE, V29, P303, DOI 10.1097/01.BRS.0000106489.03355.C5

Roy-Beaudry M, 2011, SPINE, V36, P746, DOI 10.1097/BRS.0b013e3181e040e7

Sanders JO, 2003, SPINE, V28, P2158, DOI 10.1097/01.BRS.0000084629.97042.0B

Sapountzi-Krepia DS, 2001, J ADV NURS, V35, P683, DOI 10.1046/j.1365-2648.2001.01900.x

Smith PL, 2006, SPINE, V31, P2367, DOI 10.1097/01.brs.0000240204.98960.dd

Vandal S, 1999, Issues Compr Pediatr Nurs, V22, P59

Weigert KP, 2006, EUR SPINE J, V15, P1108, DOI 10.1007/s00586-005-0014-7

NR 13

TC 21

Z9 22

U1 0

U2 10

PU LIPPINCOTT WILLIAMS & WILKINS

PI PHILADELPHIA

PA 530 WALNUT ST, PHILADELPHIA, PA 19106-3621 USA

SN 0362-2436

J9 SPINE

JI SPINE

PD JUN 15

PY 2012

VL 37

IS 14

BP 1218

EP 1223

DI 10.1097/BRS.0b013e31824b66d4

PG 6

WC Clinical Neurology; Orthopedics

WE Science Citation Index Expanded (SCI-EXPANDED)

SC Neurosciences & Neurology; Orthopedics

GA 959VH

UT WOS:000305343600015

PM 22310093

DA 2023-08-10

ER

PT J

AU Marks, M

Newton, PO

Petcharaporn, M

Bastrom, TP

Shah, S

Betz, R

Lonner, B

Miyanji, F

AF Marks, Michelle

Newton, Peter O.

Petcharaporn, Maty

Bastrom, Tracey P.

Shah, Suken

Betz, Randal

Lonner, Baron

Miyanji, Firoz

TI Postoperative Segmental Motion of the Unfused Spine Distal to the Fusion

in 100 Patients With Adolescent Idiopathic Scoliosis

SO SPINE

LA English

DT Article

DE adolescent idiopathic scoliosis; postoperative; motion assessment;

fusion-level selection decision making

ID THORACOLUMBAR DEFORMITY ARTHRODESIS; FLEXION-EXTENSION; DISC;

INSTABILITY; FATE; L5

AB Study Design. A cross-sectional study.

Objective. The purpose of this study was to assess intervertebral segmental and cumulative motion in the distal unfused segments of the spine in patients with adolescent idiopathic scoliosis after instrumentation as a function of the lowest instrumented level.

Summary of Background Data. The implications of hyper- or hypomobility in the unfused segments of the spine after instrumentation are poorly understood. There is little research on changes in functional movement capabilities of the spine after thoracolumbar spinal fusion.

Methods. Patients were prospectively offered inclusion into this institutional review board-approved cross-sectional study at their routine 2-, 3-, 4-, or 5-year postoperative visits at 1 of the 5 participating centers. Motion was assessed by standardized radiographs acquired in maximum right, left and forwarding bending positions. The intervertebral angles were measured via digital radiographic measuring software at each level from T12 to S1. The relationship of the vertebral segmental motion for each interspace to the lowest instrumented vertebrae was evaluated with an analysis of variance. The relationship between the cumulative preserved motion and each domain of the Scoliosis Research Society questionnaire were evaluated using a Pearson correlation coefficient.

Results. The data for 100 patients are included. The lowest instrumented vertebrae ranged from T10 to L4. In lateral bending, an association was detected between the lowest fused vertebral level and the degree of motion at the distal unfused segments. With a more distal instrumented vertebrae, there was significantly greater L2-L3, L3-L4, and L4-L5 segment motion ($P = 0.002$, 0.009, and 0.001, respectively). A similar trend was noticed at L5-S1 level. In addition, the summed motion from L3 to S1 also increased with a more distal fusion ($P = 0.001$). Similar results were not found in forward bending. None of the domains of the Scoliosis Research Society questionnaire correlated with the preserved L3-S1 motion.

Conclusion. In a group of postoperative patients with adolescent idiopathic scoliosis, evaluation of the distal unfused intervertebral motion showed that preservation of vertebral motion segments allowed greater distribution of functional motion across more levels. With each distal fusion level, motion was significantly increased at the L2-L3, L3-L4, and L4-L5 segmental levels in lateral bending. The relationship between the increased motion and subsequent disc degeneration with a more distal fusion is unknown, but suspected.

C1 [Marks, Michelle; Newton, Peter O.; Petcharaporn, Maty; Bastrom, Tracey P.] Rady Childrens Hosp, Dept Orthoped, San Diego, CA USA.

[Newton, Peter O.] Univ Calif San Diego, Dept Orthoped Surg, San Diego, CA 92103 USA.

[Shah, Suken] Nemours Childrens Clin, Dept Orthoped, Wilmington, DE USA.

[Betz, Randal] Shriners Hosp Children, Dept Orthoped, Philadelphia, PA USA.

[Lonner, Baron] Scoliosis Associates, New York, NY USA.

[Miyanji, Firoz] BC Childrens Hosp, Dept Orthoped, Vancouver, BC, Canada.

C3 Rady Childrens Hospital San Diego; University of California System;

University of California San Diego; Shriners Hospitals Children

Philadelphia; BC Childrens Hospital; University of British Columbia

RP Marks, M (通讯作者)，3030 Childrens Way,Suite 410, San Diego, CA 92123 USA.

EM mmarks@comcast.net

RI Shah, Suken/AAC-9857-2022

FU DePuy Spine

FX DePuy Spine funds were received by the Harms Study Group Foundation, as a research grant to support this work. One or more of the author(s) has/have received or will receive benefits for personal or professional use from a commercial party related directly or indirectly to the subject of this manuscript: e. g., honoraria, gifts, consultancies, royalties, stocks, stock options, decision making position.

CR AARO S, 1983, SPINE, V8, P570, DOI 10.1097/00007632-198309000-00002

Balderston RA, 1998, SPINE, V23, P54, DOI 10.1097/00007632-199801010-00011

BODEN SD, 1990, SPINE, V15, P571, DOI 10.1097/00007632-199006000-00026

Bridwell KH, 1999, SPINE, V24, P2607, DOI 10.1097/00007632-199912150-00008

COCHRAN T, 1983, SPINE, V8, P576, DOI 10.1097/00007632-198309000-00003

DVORAK J, 1991, SPINE, V16, P562

Edwards CC, 2003, SPINE, V28, P2122, DOI 10.1097/01.BRS.0000084266.37210.85

Engsberg JB, 2002, SPINE, V27, P1346, DOI 10.1097/00007632-200206150-00018

Fujiwara A, 2000, J SPINAL DISORD, V13, P444, DOI 10.1097/00002517-200010000-00013

Ginsberg HH, 1995, SCOL RES SOC 30 ANN

Kuhns CA, 2007, SPINE, V32, P2771, DOI 10.1097/BRS.0b013e31815a7ece

Lawhorne TW, 2008, ACCELERATED DISC DEG

Majdouline Y, 2007, J PEDIATR ORTHOPED, V27, P775, DOI 10.1097/BPO.0b013e31815588d8

PENNING L, 1984, DIAGN IMAG CLIN MED, V53, P186

Pfirrmann CWA, 2001, SPINE, V26, P1873, DOI 10.1097/00007632-200109010-00011

Thalgott JS, 2004, SPINE J, p167S

Violas P, 2007, SPINE, V32, pE405, DOI 10.1097/BRS.0b013e318074d69f

NR 17

TC 47

Z9 48

U1 0

U2 6

PU LIPPINCOTT WILLIAMS & WILKINS

PI PHILADELPHIA

PA TWO COMMERCE SQ, 2001 MARKET ST, PHILADELPHIA, PA 19103 USA

SN 0362-2436

EI 1528-1159

J9 SPINE

JI SPINE

PD MAY 1

PY 2012

VL 37

IS 10

BP 826

EP 832

DI 10.1097/BRS.0b013e31823b4eab

PG 7

WC Clinical Neurology; Orthopedics

WE Science Citation Index Expanded (SCI-EXPANDED)

SC Neurosciences & Neurology; Orthopedics

GA 935DP

UT WOS:000303499400012

PM 22024909

OA Bronze

DA 2023-08-10

ER

PT J

AU Mordecai, SC

Dabke, HV

AF Mordecai, Simon C.

Dabke, Harshad V.

TI Efficacy of exercise therapy for the treatment of adolescent idiopathic

scoliosis: a review of the literature

SO EUROPEAN SPINE JOURNAL

LA English

DT Review

ID SEAS.02 EXERCISES; COBB ANGLE; VARIABILITY; PROGRESSION; REDUCE

AB Current evidence regarding the use of exercise therapy in the treatment of adolescent idiopathic scoliosis (AIS) was assessed with a review of published literature.

An extensive literature search was carried out with commonly used medical databases. A total of 155 papers were identified out of which only 12 papers were deemed to be relevant.

There were nine prospective cohort studies, two retrospective studies and one case series. All studies endorsed the role of exercise therapy in AIS but several shortcomings were identified-lack of clarity of patient recruitment and in the method of assessment of curve magnitude, poor record of compliance, and lack of outcome scores. Many studies reported "significant" changes in the Cobb angle after treatment, which were actually of small magnitude and did not take into account the reported inter or intra-observer error rate. All studies had poor statistical analysis and did not report whether the small improvements noted were maintained in the long term.

This unbiased literature review has revealed poor quality evidence supporting the use of exercise therapy in the treatment of AIS. Well-designed randomised controlled studies are required to assess the role of exercise therapy in AIS.

C1 [Mordecai, Simon C.; Dabke, Harshad V.] Salisbury NHS Fdn Trust, Dept Trauma & Orthopaed, Salisbury Dist Hosp, Salisbury SP2 8BJ, Wilts, united kingdom.

C3 Salisbury District Hospital

RP Mordecai, SC (通讯作者)， Salisbury NHS Fdn Trust, Dept Trauma & Orthopaed, Salisbury Dist Hosp, Salisbury SP2 8BJ, Wilts, united kingdom.

EM Simon_mordecai@yahoo.com

CR Adam CJ, 2005, SPINE, V30, P1664, DOI 10.1097/01.brs.0000169449.68870.f8

[Anonymous], 2005, ZHONGGUO LINCHUANG K

Chromy CA, 2006, ARCH PHYS MED REHAB, V87, P1447, DOI 10.1016/j.apmr.2006.08.325

DARUWALLA JS, 1985, J BONE JOINT SURG BR, V67, P211, DOI 10.1302/0301-620X.67B2.3980527

DICKSON RA, 1984, J BONE JOINT SURG BR, V66, P8, DOI 10.1302/0301-620X.66B1.6693483

DICKSON RA, 1985, J BONE JOINT SURG BR, V67, P176, DOI 10.1302/0301-620X.67B2.3872301

Dobosiewicz Krystyna, 2002, Stud Health Technol Inform, V91, P336

el-Sayyad M, 1994, Int J Rehabil Res, V17, P70, DOI 10.1097/00004356-199403000-00008

EMANS JB, 1986, SPINE, V11, P792, DOI 10.1097/00007632-198610000-00009

Guo X, 2003, J BONE JOINT SURG BR, V85B, P1026, DOI 10.1302/0301-620X.85B7.14046

Lenssinck MLB, 2005, PHYS THER, V85, P1329, DOI 10.1093/ptj/85.12.1329

Mamyama Toni, 2002, Stud Health Technol Inform, V91, P361

McIntire KL, 2008, J SPINAL DISORD TECH, V21, P349, DOI 10.1097/BSD.0b013e318145b7e9

Mooney V, 2003, ORTHOPEDICS, V26, P167

MORRISSY RT, 1990, J BONE JOINT SURG AM, V72A, P320, DOI 10.2106/00004623-199072030-00002

Murray D W, 1996, Eur Spine J, V5, P251, DOI 10.1007/BF00301328

Negrini S, 2008, DISABIL REHABIL, V30, P772, DOI 10.1080/09638280801889568

Negrini S, 2008, J REHABIL MED, V40, P451, DOI 10.2340/16501977-0195

Negrini S, 2006, ST HEAL T, V123, P523

Negrini S, 2006, ST HEAL T, V123, P519

Negrini Stefano, 2003, Pediatr Rehabil, V6, P227, DOI: 10.1080/13638490310001636781

Shea KG, 1998, SPINE, V23, P551, DOI 10.1097/00007632-199803010-00007

Tanure MC, 2010, SPINE J, V10, P769, DOI 10.1016/j.spinee.2010.02.020

Weiss HR, 2008, EUR J PHYS REHAB MED, V44, P177

Weiss H R, 1992, Ital J Orthop Traumatol, V18, P395

Weiss H R, 1997, Pediatr Rehabil, V1, P35

Weiss H-R, 2006, Pediatr Rehabil, V9, P190, DOI 10.1080/13638490500079583

Weiss HR, 2006, SCOLIOSIS SPINAL DIS, V1, DOI [10.1186/1748-7161-1-5, 10.1186/1748-7161-1-1]

Weiss HR, 2006, ST HEAL T, V123, P594

Weiss Hans-Rudolf, 2002, Stud Health Technol Inform, V91, P352

Weiss Hans-Rudolf, 2003, Pediatr Rehabil, V6, P23, DOI 10.1080/1363849031000095288

WEISS HR, 1995, Z ORTHOP GRENZGEB, V133, P114, DOI 10.1055/s-2008-1039421

Weiss HR, 1995, Z ORTHOP IHRE GRENZG, V133, P118

Wong HK, 2010, INDIAN J ORTHOP, V44, P9, DOI 10.4103/0019-5413.58601

NR 34

TC 44

Z9 49

U1 0

U2 53

PU SPRINGER

PI NEW YORK

PA ONE NEW YORK PLAZA, SUITE 4600, NEW YORK, NY, UNITED STATES

SN 0940-6719

EI 1432-0932

J9 EUR SPINE J

JI Eur. Spine J.

PD MAR

PY 2012

VL 21

IS 3

BP 382

EP 389

DI 10.1007/s00586-011-2063-4

PG 8

WC Clinical Neurology; Orthopedics

WE Science Citation Index Expanded (SCI-EXPANDED)

SC Neurosciences & Neurology; Orthopedics

GA 907TK

UT WOS:000301441100002

PM 22065168

OA Green Published

DA 2023-08-10

ER

PT J

AU Shin, SS

Lee, YW

Song, CH

AF Shin, Seung Sub

Lee, Yong Woo

Song, Chang Ho

TI Effects of Lumbar Stabilization Exercise on Postural Sway of Patients

with Adolescent Idiopathic Scoliosis during Quiet Sitting

SO JOURNAL OF PHYSICAL THERAPY SCIENCE

LA English

DT Article

DE Adolescent idiopathic scoliosis; Lumbar stabilization exercise; Sitting

balance

ID LOW-BACK-PAIN; BALANCE CONTROL; MICRODISCECTOMY; REHABILITATION;

PERFORMANCE; STABILITY; IMMEDIATE; STRATEGY; SYMMETRY; STROKE

AB [Purpose] The purpose of this study was to investigate the effects of lumbar stabilization exercise on sitting balance of adolescent idiopathic scoliosis (AIS) patients. [Subjects] Eighteen patients with AIS, mean Cobb angle of 31.4 degrees, participated in this study. [Methods] The Lumbar Trunk Muscle Endurance Test (LTMET) and the Balance Performance Monitor (BPM; SMS Healthcare, Harlow, UK.) were used to measure trunk endurance and postural sway before and after the lumbar stabilization exercise, which was performed for 40 minutes per day, 3 times per week for three weeks. [Results] Sitting balance was improved as determined by the anterior-posterior sway angle, right-left sway angle and sway area under the eyes opened and closed conditions. No correlation was found between sitting balance parameters and trunk flexor endurance after the lumbar stabilization exercise. [Conclusion] The results demonstrate that lumbar stabilization exercise effectively improves sitting balance, suggesting that lumbar stabilization exercise can be clinically used for patients with AIS to improve their postural control when seated.

C1 [Shin, Seung Sub; Lee, Yong Woo; Song, Chang Ho] Sahmyook Univ, Dept Phys Therapy, Seoul 139742, South Korea.

C3 Sahmyook University

RP Shin, SS (通讯作者)， Sahmyook Univ, Dept Phys Therapy, 26-21 Gongneung,2 Dong, Seoul 139742, South Korea.

EM chsong@syu.ac.kr

FU Sahmyook University

FX This study was supported by Sahmyook University.

CR Akuthota V, 2004, ARCH PHYS MED REHAB, V85, pS86, DOI 10.1053/j.apmr.2003.12.005

[Anonymous], 2004, THERAPEUTIC EXERCISE

Barr KP, 2007, AM J PHYS MED REHAB, V86, P72, DOI 10.1097/01.phm.0000250566.44629.a0

Ben Kibler W, 2006, SPORTS MED, V36, P189, DOI 10.2165/00007256-200636030-00001

Bennett BC, 2004, SPINE, V29, pE449, DOI 10.1097/01.brs.0000142005.21714.32

Bruyneel AV, 2010, EUR SPINE J, V19, P739, DOI 10.1007/s00586-010-1325-x

Dalleau G, 2007, EUR SPINE J, V16, P1593, DOI 10.1007/s00586-007-0404-0

Drysdale CL, 2004, J ATHL TRAINING, V39, P32

Ebenbichler GR, 2001, MED SCI SPORT EXER, V33, P1889, DOI 10.1097/00005768-200111000-00014

Flanagan SP, 2007, J ORTHOP SPORT PHYS, V37, P356, DOI 10.2519/jospt.2007.2366

FORD DM, 1988, SPINE, V13, P461, DOI 10.1097/00007632-198805000-00004

Gram MC, 1999, SPINE, V24, P169, DOI 10.1097/00007632-199901150-00019

Guo X, 2006, SPINE, V31, pE437, DOI 10.1097/01.brs.0000222048.47010.bf

Haas B M, 2000, Physiother Res Int, V5, P19, DOI 10.1002/pri.181

Haas B M, 1998, Physiother Res Int, V3, P135, DOI 10.1002/pri.132

Hemami H, 2006, IEEE T NEUR SYS REH, V14, P470, DOI 10.1109/TNSRE.2006.886718

Ito T, 1996, ARCH PHYS MED REHAB, V77, P75, DOI 10.1016/S0003-9993(96)90224-5

KANE WJ, 1977, CLIN ORTHOP RELAT R, P43

Mallau S, 2007, SPINE, V32, pE14, DOI 10.1097/01.brs.0000251069.58498.eb

Mok NW, 2004, SPINE, V29, pE107, DOI 10.1097/01.BRS.0000115134.97854.C9

Moreau CE, 2001, J MANIP PHYSIOL THER, V24, P110, DOI 10.1067/mmt.2001.112563

Mudie MH, 2002, CLIN REHABIL, V16, P582, DOI 10.1191/0269215502cr527oa

Muthukrishnan R, 2010, BMC SPORTS SCI MED R, V2, DOI 10.1186/1758-2555-2-13

Nault ML, 2002, SPINE, V27, P1911, DOI 10.1097/00007632-200209010-00018

Perret C, 2001, EUR SPINE J, V10, P363, DOI 10.1007/s005860100279

Radebold A, 2001, SPINE, V26, P724, DOI 10.1097/00007632-200104010-00004

Sackley CM, 2005, CLIN REHABIL, V19, P746, DOI 10.1191/0269215505cr863oa

Selkowitz DM, 2006, BMC MUSCULOSKEL DIS, V7, DOI 10.1186/1471-2474-7-70

SHIRADO O, 1995, ARCH PHYS MED REHAB, V76, P621, DOI 10.1016/S0003-9993(95)80630-X

Simoneau M, 2006, EXP BRAIN RES, V170, P576, DOI 10.1007/s00221-005-0246-0

Standaert CJ, 2008, SPINE J, V8, P114, DOI 10.1016/j.spinee.2007.10.015

Tsao H, 2007, EXP BRAIN RES, V181, P537, DOI 10.1007/s00221-007-0950-z

Webber SC, 2004, CLIN BIOMECH, V19, P777, DOI 10.1016/j.clinbiomech.2004.05.014

Weinstein SL, 1999, SPINE, V24, P2592, DOI 10.1097/00007632-199912150-00006

Wilder DG, 1996, SPINE, V21, P2628, DOI 10.1097/00007632-199611150-00013

Zazulak B, 2008, J AM ACAD ORTHOP SUR, V16, P497, DOI 10.5435/00124635-200809000-00002

NR 36

TC 7

Z9 7

U1 0

U2 15

PU SOC PHYSICAL THERAPY SCIENCE

PI TOKYO

PA C/O PUBLICATION CENTER, 1-24-12 SUGAMO, TOSHIMA-KU, TOKYO, 170-0002,

JAPAN

SN 0915-5287

EI 2187-5626

J9 J PHYS THER SCI

JI J. Phys. Ther. Sci.

PD MAR

PY 2012

VL 24

IS 2

BP 211

EP 215

PG 5

WC Rehabilitation

WE Science Citation Index Expanded (SCI-EXPANDED)

SC Rehabilitation

GA 946EU

UT WOS:000304335100014

DA 2023-08-10

ER

PT J

AU Romano, M

Minozzi, S

Bettany-Saltikov, J

Zaina, F

Chockalingam, N

Kotwicki, T

Maier-Hennes, A

Negrini, S

AF Romano, Michele

Minozzi, Silvia

Bettany-Saltikov, Josette

Zaina, Fabio

Chockalingam, Nachiappan

Kotwicki, Tomasz

Maier-Hennes, Axel

Negrini, Stefano

TI Exercises for adolescent idiopathic scoliosis

SO COCHRANE DATABASE OF SYSTEMATIC REVIEWS

LA English

DT Review

ID UPDATED METHOD GUIDELINES; SYSTEMATIC REVIEWS; SEAS.02 EXERCISES; BRACE

TREATMENT; PROGRESSION; REHABILITATION; QUESTIONNAIRE; EFFICACY;

PROGRAM; REDUCE

AB Background

Adolescent idiopathic scoliosis (AIS) is a three-dimensional deformity of the spine. While AIS can progress during growth and cause a surface deformity, it is usually not symptomatic. However, in adulthood, if the final spinal curvature surpasses a certain critical threshold, the risk of health problems and curve progression is increased. The use of scoliosis-specific exercises (SSE) to reduce progression of AIS and postpone or avoid other more invasive treatments is controversial.

Objectives

To evaluate the efficacy of SSE in adolescent patients with AIS.

Search methods

The following databases (up to 30 March 2011) were searched with no language limitations: CENTRAL (The Cochrane Library 2011, issue 2), MEDLINE (from January 1966), EMBASE (from January 1980), CINHAL (from January 1982), SportDiscus (from January 1975), PsycInfo (from

January 1887), PEDro (from January 1929). We screened reference lists of articles and also conducted an extensive handsearch of grey literature.

Selection criteria

Randomised controlled trials and prospective cohort studies with a control group comparing exercises with no treatment, other treatment, surgery, and different types of exercises.

Data collection and analysis

Two review authors independently selected studies, assessed risk of bias and extracted data.

Main results

Two studies (154 participants) were included. There is low quality evidence from one randomised controlled study that exercises as an adjunctive to other conservative treatments increase the efficacy of these treatments (thoracic curve reduced: mean difference (MD) 9.00, (95% confidence interval (CI) 5.47 to 12.53); lumbar curve reduced: MD 8.00, (95% CI 5.08 to 10.92)). There is very low quality evidence from a prospective controlled cohort study that scoliosis-specific exercises structured within an exercise programme can reduce brace prescription (risk ratio (RR) 0.24, (95% CI 0.06 to1.04) as compared to usual physiotherapy (many different kinds of general exercises according to the preferences of the single therapists within different facilities).

Authors' conclusions

There is a lack of high quality evidence to recommend the use of SSE for AIS. One very low quality study suggested that these exercises may be more effective than electrostimulation, traction and postural training to avoid scoliosis progression, but better quality research needs to be conducted before the use of SSE can be recommended in clinical practice.

C1 [Romano, Michele; Zaina, Fabio] ISICO Italian Sci Spine Inst, I-20141 Milan, Italy.

[Minozzi, Silvia] Lazio Reg Hlth Serv, Dept Epidemiol, Rome, Italy.

[Bettany-Saltikov, Josette] Univ Teeside, Sch Hlth & Social Care, Middlesbrough, Cleveland, united kingdom.

[Chockalingam, Nachiappan] Staffordshire Univ, Fac Hlth, Stoke On Trent ST4 2DE, Staffs, united kingdom.

[Kotwicki, Tomasz] Univ Med Sci, Dept Pediat Orthoped & Traumatol, Poznan, Poland.

[Maier-Hennes, Axel] Scoliosis Rehabil Ctr, Bad Sobernheim, Germany.

[Negrini, Stefano] Univ Brescia, Don Gnocchi Fdn Milan, Brescia, Italy.

C3 University of Teesside; Staffordshire University; University of Brescia

RP Romano, M (通讯作者)， ISICO Italian Sci Spine Inst, Via Roberto Bellarmino 13-1, I-20141 Milan, Italy.

EM michele.romano@isico.it

RI Negrini, Stefano/B-6667-2013; Zaina, Fabio/H-3261-2013; Chockalingam,

Nachiappan/C-4423-2014; Bettany-Saltikov, Josette/C-5388-2011; Minozzi,

Silvia/ABG-1115-2020

OI Negrini, Stefano/0000-0002-1878-2747; Zaina, Fabio/0000-0002-1256-5362;

Chockalingam, Nachiappan/0000-0002-7072-1271; Bettany-Saltikov,

Josette/0000-0001-7784-500X; Minozzi, Silvia/0000-0003-0471-8581

CR [Anonymous], 1992, PHYSIOTHERAPY

[Anonymous], CHIN J CLIN REHABIL

[Anonymous], 2015, NEWCASTLE OTTAWA SCA

Asher M, 2003, SPINE, V28, P63, DOI 10.1097/00007632-200301010-00015

Athanasopoulos S, 1999, SCAND J MED SCI SPOR, V9, P36

Atkins D, 2004, BMJ-BRIT MED J, V328, P1490

Boutron I, 2005, J CLIN EPIDEMIOL, V58, P1233, DOI 10.1016/j.jclinepi.2005.05.004

CARMAN D, 1985, J PEDIATR ORTHOPED, V5, P65, DOI 10.1097/01241398-198501000-00011

Alves VLD, 2006, CHEST, V130, P500, DOI 10.1378/chest.130.2.500

Ducong P, 2002, RESONANCE EUROPEENNE, V10, P1229

Durmala Jacek, 2003, Ortop Traumatol Rehabil, V5, P80

Durmala Jacek, 2002, Stud Health Technol Inform, V91, P357

Dyner-Jama I, 2000, Wiad Lek, V53, P603

Furlan AD, 2009, SPINE, V34, P1929, DOI 10.1097/BRS.0b013e3181b1c99f

Hawes Martha C, 2003, Pediatr Rehabil, V6, P171

Higgins J, 2009, COCHRANE HDB SYSTEMA

Kotwicki T, 2007, SCOLIOSIS SPINAL DIS, V2, DOI 10.1186/1748-7161-2-1

Kowalski I M, 2001, Ortop Traumatol Rehabil, V3, P276

Lenssinck MLB, 2005, PHYS THER, V85, P1329, DOI 10.1093/ptj/85.12.1329

Lonstein JE, 2006, CLIN ORTHOP RELAT R, V443, P284

Mamyama Toni, 2002, Stud Health Technol Inform, V91, P361

Maruyama Toru, 2003, Pediatr Rehabil, V6, P215

McIntire KL, 2008, J SPINAL DISORD TECH, V21, P349, DOI 10.1097/BSD.0b013e318145b7e9

Mooney V, 2003, ORTHOPEDICS, V26, P167

Negrini S, 2008, DISABIL REHABIL, V30, P772, DOI 10.1080/09638280801889568

Negrini S, 2010, COCHRANE DB SYST REV, DOI 10.1002/14651858.CD006850.pub2

Negrini S, 2008, J REHABIL MED, V40, P451, DOI 10.2340/16501977-0195

Negrini S, 2006, ST HEAL T, V123, P523

Negrini S, 2006, ST HEAL T, V123, P519

Negrini Stefano, 2003, Pediatr Rehabil, V6, P227, DOI: 10.1080/13638490310001636781

Negrini S, 2012, SCOLIOSIS SPINAL DIS, V7, DOI 10.1186/1748-7161-7-3

Omeroglu H, 1996, Eur Spine J, V5, P167, DOI 10.1007/BF00395508

Reichel Dagmar, 2003, Pediatr Rehabil, V6, P221

Rigo M, 2006, STUD HEALTH TECHNOL, V123, P90

Romano M, 2006, SCOLIOSIS, V1, P1

Scoliosis Resarch Society, 2006, BRAC WEAR COMPL

Scoliosis Research Society, 2007, SCOL RES SOC BRAC MA

SHEKELLE PG, 1994, SPINE, V19, pS2028

van Tulder M, 2003, SPINE, V28, P1290, DOI 10.1097/00007632-200306150-00014

Vasiliadis E, 2006, SCOLIOSIS SPINAL DIS, V1, DOI 10.1186/1748-7161-1-7

WEINSTEIN SL, 1983, J BONE JOINT SURG AM, V65, P447, DOI 10.2106/00004623-198365040-00004

Weinstein SL, 2003, JAMA-J AM MED ASSOC, V289, P559, DOI 10.1001/jama.289.5.559

Weiss H R, 1995, Eur Spine J, V4, P34, DOI 10.1007/BF00298416

Weiss H R, 1997, Pediatr Rehabil, V1, P35

Weiss H-R, 2006, Pediatr Rehabil, V9, P190, DOI 10.1080/13638490500079583

Weiss HR, 2006, SCOLIOSIS SPINAL DIS, V1, DOI 10.1186/1748-7161-1-6

Weiss Hans-Rudolf, 2003, Pediatr Rehabil, V6, P23, DOI 10.1080/1363849031000095288

Weiss HR, 2002, ST HEAL T, V88, P304

WEISS HR, 1995, Z ORTHOP GRENZGEB, V133, P114, DOI 10.1055/s-2008-1039421

WEISS HR, 1991, SPINE, V16, P88, DOI 10.1097/00007632-199101000-00016

Weiss HR, 2003, PEDIAT REHABILITATIO, V6, P209

Zaina F, 2009, SCOLIOSIS, V4

NR 52

TC 82

Z9 84

U1 3

U2 61

PU WILEY

PI HOBOKEN

PA 111 RIVER ST, HOBOKEN 07030-5774, NJ USA

SN 1469-493X

EI 1361-6137

J9 COCHRANE DB SYST REV

JI Cochrane Database Syst Rev.

PY 2012

IS 8

AR CD007837

DI 10.1002/14651858.CD007837.pub2

PG 33

WC Medicine, General & Internal

WE Science Citation Index Expanded (SCI-EXPANDED)

SC General & Internal Medicine

GA 993BK

UT WOS:000307828900018

PM 22895967

OA Green Published

DA 2023-08-10

ER

PT J

AU Solache-Carranco, A

Sanchez-Bringas, MG

AF Solache-Carranco, Angela

Guadalupe Sanchez-Bringas, Maria

TI Evaluation of a respiratory rehabilitation program in children with

scoliosis

SO CIRUGIA Y CIRUJANOS

LA English

DT Article

DE scoliosis; respiratory rehabilitation; lung function; spirometry;

dyspnea

ID ADOLESCENT IDIOPATHIC SCOLIOSIS; PULMONARY-FUNCTION; SURGICAL-TREATMENT;

LUNG-FUNCTION; CHEST-WALL; UPDATE

AB Background: Thoracic scoliosis is a lateral curvature of the spine associated with restrictive lung defects, manifested by a decrease in respiratory function tests. We undertook this study to evaluate the effect of a respiratory rehabilitation program over lung function in children with scoliosis.

Methods: We carried out a prospective and deliberate interventional study including 25 consecutive patients, aged 6 to 18 years, diagnosed with thoracic scoliosis. The respiratory rehabilitation program was structured into two phases: institutional and private residence. Statistical analysis was carried out using descriptive parameters, paired t-test and Wilcoxon signed-ranks test. Spearman correlation was used to measure intensity of association among variables. Statistical significance was considered when $p < 0.05$.

Results: Idiopathic scoliosis was present in 52% of patients, with right dorsal curvature in 72%. Cobb angle average was 50.6 degrees +/- 29.7 degrees. Most importantly, we found a negative correlation between this angle on left curvature and lung function. Initially, the main respiratory symptoms were dyspnea with poor exercise tolerance in 52%. After treatment, 88% of patients were asymptomatic and only 4% presented poor exercise tolerance. Oxygen saturation and forced vital capacity percentage showed a significant increment after the program.

Conclusions: Respiratory rehabilitation has a positive effect on increasing pulmonary function of children with scoliosis.

C1 [Solache-Carranco, Angela] Inst Nacl Rehabil, Serv Rehabil Pulm, Mexico City 14389, DF, Mexico.

[Guadalupe Sanchez-Bringas, Maria] Inst Nacl Rehabil, Serv Cirugia Columna Vertebral, Mexico City 14389, DF, Mexico.

RP Solache-Carranco, A (通讯作者), Inst Nacl Rehabil, Serv Rehabil Pulm, Calzada Mexico Xochimilco 289, Mexico City 14389, DF, Mexico.

EM asolache@inr.gob.mx

FU Medico de Rehabilitacion, INR; Division de Rehabilitacion Geriatrica y

Cardiorrespiratoria, INR

FX We thank the following persons for their support: Dr. Alvaro Lomeli

Rivas, Subdirector Medico de Rehabilitacion, INR; Dra. Juana Zavala

Ramirez, Chief, Division de Rehabilitacion Geriatrica y

Cardiorrespiratoria, INR. This study was presented as a poster at the

Congreso Internacional de Investigacion en Rehabilitacion, Mexico,

September 27- October 1, 2010.

CR American Association of Cardiovascular and Pulmonary Rehabilitation, 2004, GUID PULM REH PROGR, P80

Avendano M, 2003, ARCH BRONCONEUMOL, V39, P559, DOI 10.1157/13054362

Barrios C, 2005, SPINE, V30, P1610, DOI 10.1097/01.brs.0000169447.55556.01

Campos MA, 2007, NEUROSURG CLIN N AM, V18, P515, DOI 10.1016/j.nec.2007.04.007

Chan G, 2009, SPINE, V34, P1766, DOI 10.1097/BRS.0b013e3181ab62d8

Colomina M J, 2005, Rev Esp Anestesiol Reanim, V52, P24

Crawford AH, 2007, ORTHOP CLIN N AM, V38, P553, DOI 10.1016/j.ocl.2007.03.008

Dyner-Jama I, 2000, Wiad Lek, V53, P603

Guell R, 2005, TRATADO REHABILITACI, P255

Jimenez-Cosmes L, 2009, REHABILITACION, V43, P246

Kim HJ, 2009, CURR OPIN PEDIATR, V21, P55, DOI 10.1097/MOP.0b013e328320a929

Kotani T, 2004, SPINE, V29, P298, DOI 10.1097/01.BRS.0000106490.82936.89

Koumbourlis AC, 2006, PAEDIATR RESPIR REV, V7, P152, DOI 10.1016/j.prrv.2006.04.009

McMaster MJ, 2007, J SPINAL DISORD TECH, V20, P203, DOI 10.1097/01.bsd.0000211270.51368.43

Molina A, 2003, ARCH BRONCONEUMOL, V39, P507, DOI 10.1157/13053215

NEPPLE JJ, 2009, SPINE J, V9, P9

Oskouian RJ, 2007, NEUROSURG CLIN N AM, V18, P479, DOI 10.1016/j.nec.2007.04.004

Pehrsson K, 2001, THORAX, V56, P388, DOI 10.1136/thorax.56.5.388

Redding G, 2008, SPINE J, V8, P639, DOI 10.1016/j.spinee.2007.04.020

Salcedo-Posadas A, 2001, AN ESP PEDIAT S2, V54, P41

Segal SL, 2006, CURR OPIN ORTHOP, V17, P493

Smiljanic I, 2009, COLLEGIUM ANTROPOL, V33, P145

Takahashi S, 2007, SPINE, V32, P106, DOI 10.1097/01.brs.0000251005.31255.25

Van Goethem J, 2007, NEUROIMAG CLIN N AM, V17, P105, DOI 10.1016/j.nic.2006.12.001

Vedantam R, 2000, SPINE, V25, P82, DOI 10.1097/00007632-200001010-00015

Vitale MG, 2008, SPINE, V33, P1242, DOI 10.1097/BRS.0b013e3181714536

Weinstein SL, 2008, LANCET, V371, P1527, DOI 10.1016/S0140-6736(08)60658-3

NR 27

TC 2

Z9 2

U1 0

U2 11

PU MEXICAN ACAD SURGERY

PI MEXICO D G

PA CENTRO MED NAC SIGLO XXI, EDIFICIO BLOQUE B, SOTANO, AVE CUAUHTEMOC NO

330, MEXICO D G, 06700, MEXICO

SN 0009-7411

J9 CIR CIR

JI Cir. Cir.

PD JAN-FEB

PY 2012

VL 80

IS 1

BP 11

EP 17

PG 7

WC Surgery

WE Science Citation Index Expanded (SCI-EXPANDED)

SC Surgery

GA 902QG

UT WOS:000301052400003

PM 22472147

DA 2023-08-10

ER

PT J

AU De Mauroy, JC

Vallese, P

Lalain, JJ

AF De Mauroy, J. C.

Vallese, P.

Lalain, J. J.

TI Lyon conservative treatment of adult scoliosis

SO MINERVA ORTOPEDICA E TRAUMATOLOGICA

LA English

DT Article

DE Scoliosis; Braces; Casts, surgical; Physical therapy modalities

ID TERM FOLLOW-UP

AB The aim of this paper was to evaluate evidence for the efficacy and effectiveness of proposed conservative treatment options in adult deformity. Adult deformity is a major demographic health issue in the geriatric population. Surgeons are often very conservative in the treatment of adult scoliosis because of the complication rates associated with the surgeries and the marginal bone quality endemic to this population. There is currently a lack of consensus on the most efficacious conservative treatments for adult deformity. The Lyon Conservative treatment requires: 1) a plaster cast made in a specific standing frame for 3 weeks; 2) a rigid polyethylene bivalve overlapped brace worn for at least 4 hours per day; 3) a specific physiotherapy to prevent muscle atrophy. The plaster cast is an indispensable prerequisite for this treatment. Besides the therapeutic role of muscular-ligamentous adjustment of paravertebral tension, it can also be used as a test. The patient must be pain-free while pursuing normal activities. Medical complications are a major concern in adult spinal deformity surgery. The incidence ranges between 40% to 86%, but there is indeterminate level III/IV evidence on the effectiveness of any usual conservative care option. Thirty-three adult scoliosis patients (30 women and 3 men), were controlled for at least 5 years after the beginning of the Lyon Conservative treatment. The average age at the beginning of treatment is 60 years. In every case the

pain and the posture improved. In 31 cases out of 33, the Cobb's angle was stabilized. 5 years after initiating treatment, half of the patients are continuing to wear the brace for more than 4 hours a day. Conservative care in general may be a helpful option in the care of adult deformity, but evidence for this decision is lacking. Lyon Conservative orthopedic treatment is effective. The decision regarding the treatment is taken thanks to the first stage of the plaster cast. Basic clinical research at any level would be helpful to further clarify the options.

C1 [De Mauroy, J. C.; Vallese, P.; Lalain, J. J.] Spine Orthoped Dept, F-69006 Lyon, France.

RP De Mauroy, JC (通讯作者)， Spine Orthoped Dept, Clin Parc,155 Blvd Stalingrad, F-69006 Lyon, France.

EM demauroy@aol.com

RI de Mauroy, Jean Claude/AAJ-3087-2020

CR Akbarnia BA, 2006, SPINE, V31, pS195, DOI 10.1097/01.brs.0000234732.43489.0f

Baron EM, 2006, SPINE, V31, pS106, DOI 10.1097/01.brs.0000232713.69342.df

Buttermann GR, 2008, EUR SPINE J, V17, P240, DOI 10.1007/s00586-007-0530-8

Chin Kingsley R, 2009, Am J Orthop (Belle Mead NJ), V38, P404

Chuah S L, 2001, Med J Malaysia, V56 Suppl C, P37

DUVALBEAUPERE G, 1972, REV CHIR ORTHOP, V58, P323

EDGAR MA, 1988, J BONE JOINT SURG BR, V70, P712, DOI 10.1302/0301-620X.70B5.3192566

Everett CR, 2007, SPINE, V32, pS130, DOI 10.1097/BRS.0b013e318134ea88

Hong JY, 2010, J SPINAL DISORD TECH, V23, P461, DOI 10.1097/BSD.0b013e3181bf1a85

Kluba T, 2009, ARCH ORTHOP TRAUM SU, V129, P1, DOI 10.1007/s00402-008-0673-z

Kobayashi T, 2006, SPINE, V31, P178, DOI 10.1097/01.brs.0000194777.87055.1b

KOROVESSIS P, 1994, SPINE, V19, P1926, DOI 10.1097/00007632-199409000-00012

Murata Y, 2002, SPINE, V27, P2268, DOI 10.1097/00007632-200210150-00016

OBRIEN J, 2010, SCOLIOSIS, V5, pO43

Ogilvie J W, 1992, Instr Course Lect, V41, P251

Ploumis A, 2009, SPINE, V34, P1581, DOI 10.1097/BRS.0b013e31819c94cc

Schwab FJ, 2002, SPINE, V27, P387, DOI 10.1097/00007632-200202150-00012

Schwab F, 2006, SPINE, V31, pE959, DOI 10.1097/01.brs.0000248126.96737.0f

Smith JS, 2011, SPINE, V36, P817, DOI 10.1097/BRS.0b013e3181e21783

VANDERPOOL DW, 1969, J BONE JOINT SURG AM, VA 51, P446, DOI 10.2106/00004623-196951030-00002

WEINSTEIN SL, 1981, J BONE JOINT SURG AM, V63, P702, DOI 10.2106/00004623-198163050-00003

NR 21

TC 3

Z9 3

U1 0

U2 8

PU EDIZIONI MINERVA MEDICA

PI TURIN

PA CORSO BRAMANTE 83-85 INT JOURNALS DEPT., 10126 TURIN, ITALY

SN 0026-4911

EI 1827-1707

J9 MINERVA ORTOP TRAUMA

JI Minerva Ortop. Traumatol.

PD OCT

PY 2011

VL 62

IS 5

BP 385

EP 396

PG 12

WC Orthopedics

WE Science Citation Index Expanded (SCI-EXPANDED)

SC Orthopedics

GA 832PF

UT WOS:000295816600008

DA 2023-08-10

ER

PT J

AU Bas, P

Romagnoli, M

Gomez-Cabrera, MC

Bas, JL

Aura, JV

Franco, N

Bas, T

AF Bas, Paloma

Romagnoli, Marco

Gomez-Cabrera, Mari-Carmen

Luis Bas, Jose

Villar Aura, Javier

Franco, Nuria

Bas, Teresa

TI Beneficial effects of aerobic training in adolescent patients with moderate idiopathic scoliosis

SO EUROPEAN SPINE JOURNAL

LA English

DT Article

DE Maximal oxygen consumption; Anaerobic threshold; Body fat; Life

expectancy

ID EXERCISE CAPACITY; MILD; GIRLS

AB Aim and Methods The major aim of this study was to determine whether after 6 weeks of aerobic training adolescent idiopathic scoliosis (AIS) girls who suffer from mild scoliotic curvatures (n = 6) behaved in a similar way than healthy controls (n = 6) in different biochemical, anthropometric, and cardio respiratory parameters.

Results The maximal power output and the power output achieved at the anaerobic threshold (AT), during the maximal exercise test, were significantly increased in both experimental groups, when compared with resting conditions. The training program caused significant changes in body composition (i.e., a decrease in body fat %) only in the scoliotic group. Regarding the cardio respiratory measurements, VO(2max) was increased by 17% in AIS group and 10% in the healthy group.

Conclusions Our results suggest that physical activity should be encouraged in scoliotic girls with mild curvatures.

C1 [Bas, Paloma; Bas, Teresa] Hosp Univ La Fe, Dept Orthoped Surg & Traumatol, Valencia, Spain.

[Romagnoli, Marco] Catholic Univ Valencia, Dept Sports, Valencia, Spain.

[Romagnoli, Marco] Univ Valencia, Dept Phys Educ & Sports, Valencia, Spain.

[Gomez-Cabrera, Mari-Carmen] Univ Valencia, Dept Physiol, Fac Med, Valencia, Spain.

[Gomez-Cabrera, Mari-Carmen] Fdn Invest Hosp Clin Univ INCLIVA, Valencia, Spain.

[Luis Bas, Jose] Hosp Gen Castellon, Dept Orthoped Surg & Traumatol, Castellon de La Plana, Spain.

[Villar Aura, Javier] Catholic Univ Valencia, Dept Phys Educ & Sports, Valencia, Spain.

[Franco, Nuria] Hosp Denia Alicante, Dept Orthoped Surg & Traumatol, Denia, Spain.

C3 Hospital Universitari i Politecnic La Fe; CONEXUS; Universidad Catolica

de Valencia San Vicente Martir; University of Valencia; University of

Valencia; CONEXUS; Universidad Catolica de Valencia San Vicente Martir

RP Bas, P (通讯作者)，Hosp Univ La Fe, Dept Orthoped Surg & Traumatol, Valencia, Spain.

EM palobasher@gmail.com

RI Gomez-Cabrera, Maria Carmen/H-6911-2018; Gomez-Cabrera, Mari
Carmen/O-3109-2019
OI Gomez-Cabrera, Maria Carmen/0000-0003-4000-1684; Gomez-Cabrera, Mari
Carmen/0000-0003-4000-1684; bas, teresa/0000-0003-0405-7883
FU GEER (Spanish Group for Spinal Disease); University of Valencia (Spain)
(Medical College Organisation)
FX This study was supported by grant from GEER (Spanish Group for Spinal
Disease) and grant from University of Valencia (Spain) (Medical College
Organisation).
CR Athanasopoulos S, 1999, SCAND J MED SCI SPOR, V9, P36
Barrios C, 2005, SPINE, V30, P1610, DOI 10.1097/01.brs.0000169447.55556.01
BECKER TJ, 1986, CLIN SPORT MED, V5, P149
BJURE J, 1969, ACTA ORTHOP SCAND, V40, P325, DOI 10.3109/17453676908989511
CHONG KC, 1981, J PEDIATR ORTHOPED, V1, P251, DOI 10.1097/01241398-198111000-00002
DIROCCO PJ, 1988, ARCH PHYS MED REHAB, V69, P198
DURNIN JVG, 1967, BRIT J NUTR, V21, P681, DOI 10.1079/BJN19670070
Kenanidis E, 2008, SPINE, V33, P2160, DOI 10.1097/BRS.0b013e31817d6db3
KESTEN S, 1991, CHEST, V99, P663, DOI 10.1378/chest.99.3.663
LEECH JA, 1985, J PEDIATR-US, V106, P143, DOI 10.1016/S0022-3476(85)80487-X
Lenke LG, 2001, J BONE JOINT SURG AM, V83A, P1169, DOI 10.2106/00004623-200108000-00006
McArdle WD., 2010, EXERCISE PHYSL NUTR
Myers J, 2002, NEW ENGL J MED, V346, P793, DOI 10.1056/NEJMoa011858
PATE RR, 1995, JAMA-J AM MED ASSOC, V273, P402, DOI 10.1001/jama.273.5.402
SHNEERSON JM, 1979, ACTA ORTHOP SCAND, V50, P303, DOI 10.3109/17453677908989771

Wagner PD, 1996, ANNU REV PHYSIOL, V58, P21, DOI 10.1146/annurev.physiol.58.1.21

WARREN MP, 1986, NEW ENGL J MED, V314, P1348, DOI 10.1056/NEJM198605223142104

Yusuf S, 2004, LANCET, V364, P937, DOI 10.1016/S0140-6736(04)17018-9

NR 18

TC 8

Z9 11

U1 3

U2 12

PU SPRINGER

PI NEW YORK

PA 233 SPRING ST, NEW YORK, NY 10013 USA

SN 0940-6719

J9 EUR SPINE J

JI Eur. Spine J.

PD AUG

PY 2011

VL 20

SU 3

SI SI

BP 415

EP 419

DI 10.1007/s00586-011-1902-7

PG 5

WC Clinical Neurology; Orthopedics

WE Science Citation Index Expanded (SCI-EXPANDED)

SC Neurosciences & Neurology; Orthopedics

GA 811NZ

UT WOS:000294219500011

PM 21779857

OA Green Published

DA 2023-08-10

ER

PT J

AU Muller, C

Fuchs, K

Winter, C

Rosenbaum, D

Schmidt, C

Bullmann, V

Schulte, TL

AF Mueller, Carsten

Fuchs, Katharina

Winter, Corinna

Rosenbaum, Dieter

Schmidt, Carolin

Bullmann, Viola

Schulte, Tobias L.

TI Prospective evaluation of physical activity in patients with idiopathic scoliosis or kyphosis receiving brace treatment

SO EUROPEAN SPINE JOURNAL

LA English

DT Article

DE Step activity monitoring (SAM); Bracing; Compliance; Idiopathic

adolescent scoliosis; Idiopathic adolescent kyphosis

ID SCHEUERMANN-KYPHOSIS; AMBULATORY ACTIVITY; ACTIVITY MONITOR; SPINAL

ORTHOSIS; GAIT; QUESTIONNAIRE; RELIABILITY; MANAGEMENT; YOUTH; LIFE

AB Bracing is an established method of conservative treatment for adolescent idiopathic scoliosis and kyphosis. Compliance among adolescents is frequently inadequate due to the discomfort of wearing a brace, cosmetic issues, and fear on the part of patients and parents that bracing may reduce everyday physical activities. The aim of this prospective, controlled study was to objectify the impact of spinal bracing on daily step activity in patients receiving conservative treatment for adolescent idiopathic scoliosis (AIS) or adolescent kyphosis (AK). Forty-eight consecutive patients (mean age 13.4 +/- A 2.3 years), consisting of 38 AIS patients (33 girls, 5 boys) and 10 AK patients (6 girls, 4 boys) were included. Once the decision to carry out bracing had been taken and while the patients were waiting for the individual brace to be built, step activity was assessed without braces by means of step activity monitoring (SAM) for seven consecutive days. After 8 weeks of brace wearing, step activity was assessed during regular brace treatment, again for seven consecutive days. In addition, brace-wearing times were simultaneously recorded using temperature probes implanted in the braces to measure compliance. Before and during brace treatment, patients completed the Scoliosis Research Society (SRS-22) questionnaire. The SAM was worn for an average of 12.7 +/- A 1.5 h/day during the first measurement and 12.3 +/- A 1.9 h on average during the second measurement. The mean gait cycles (GCs) per day and per hour before treatment were 5,036 +/- A 1,465 and 395 +/- A 105, respectively. No significant reduction in step activity was found at the follow-up measurement during bracing, at 4,880 +/- A 1,529 GCs/day and 403 +/- A 144 GCs/h. Taking the 23-h recommended time for brace wearing as a basis (100%), patients wore the brace for 72.7 +/- A 27.6% of the prescribed time, indicating an acceptable level of compliance. Girls showed a higher compliance level (75.6 +/- A 25.6%) in comparison with boys (56.7 +/- A 31.9%), although the difference was not significant (P = 0.093). The SRS-22 total score showed no differences between the two measurements (2.57 +/- A 0.23 vs. 2.56 +/- A 0.28). Implementing a simultaneous and objective method of assessing step activity and brace-wearing times in everyday life proved to be feasible, and it expands the information available regarding the impact of bracing on patients' quality of life. The results clearly show that brace treatment does not negatively interfere with daily step activity in AIS and AK patients. This is an important finding that should help reduce patients' and parents' worries concerning bracing.

C1 [Mueller, Carsten] Univ Hosp, Inst Expt Musculoskeletal Med IEMM, Movement Anal Lab, Munster, Germany.

[Mueller, Carsten; Winter, Corinna; Rosenbaum, Dieter] Univ Hosp Munster, Movement Anal Lab, Munster, Germany.

[Fuchs, Katharina; Schmidt, Carolin; Bullmann, Viola; Schulte, Tobias L.] Univ Hosp Munster, Dept Orthoped & Tumor Orthoped, Munster, Germany.

C3 University of Munster; University of Munster; University of Munster

RP Muller, C (通讯作者)， Univ Hosp, Inst Expt Musculoskeletal Med IEMM, Movement Anal Lab, Munster, Germany.

EM c.mueller@uni-muenster.de

RI Rosenbaum, Dieter/U-6089-2019; Müller, Carsten/G-6683-2014; Schmoelz,

Camilie/D-1707-2012

OI Müller, Carsten/0000-0002-6364-9937; Schmoelz,

Camilie/0000-0003-2221-9954

CR Arlet V, 2005, EUR SPINE J, V14, P817, DOI 10.1007/s00586-004-0750-0

Bjornson KF, 2007, PHYS THER, V87, P259, DOI 10.2522/ptj.20060157.ar

BLAND JM, 1986, LANCET, V1, P307, DOI 10.1016/s0140-6736(86)90837-8

Bulthuis GJ, 2008, EUR SPINE J, V17, P231, DOI 10.1007/s00586-007-0513-9

Bunge EM, 2007, EUR SPINE J, V16, P83, DOI 10.1007/s00586-006-0097-9

CASPERSEN CJ, 1985, PUBLIC HEALTH REP, V100, P126

Cheung KMC, 2007, INT ORTHOP, V31, P507, DOI 10.1007/s00264-006-0209-5

COUGHLIN SS, 1990, J CLIN EPIDEMIOL, V43, P87, DOI 10.1016/0895-4356(90)90060-3

de Mauroy JC, 2010, SCOLIOSIS SPINAL DIS, V5, DOI 10.1186/1748-7161-5-9

Dolan LA, 2007, SPINE, V32, pS91, DOI 10.1097/BRS.0b013e318134ead9

Heary RF, 2008, NEUROSURGERY, V63, pA222, DOI 10.1227/01.NEU.0000320384.93384.28

Heary RF, 2008, NEUROSURGERY, V63, pA125, DOI 10.1227/01.NEU.0000320387.93907.97

Helfenstein A, 2006, SPINE, V31, P339, DOI 10.1097/01.brs.0000197412.70050.0d

Karol LA, 2001, SPINE, V26, P2001, DOI 10.1097/00007632-200109150-00013

Katz DE, 2001, SPINE, V26, P2354, DOI 10.1097/00007632-200111010-00012

Korovessis P, 2007, EUR SPINE J, V16, P537, DOI 10.1007/s00586-006-0214-9

Liljenqvist U, 2006, SPORTVERLETZ SPORTSC, V20, P36, DOI 10.1055/s-2005-859029

Lowe TG, 2007, SPINE, V32, pS115, DOI 10.1097/BRS.0b013e3181354501

Mahaudens P, 2009, EUR SPINE J, V18, P1160, DOI 10.1007/s00586-009-1002-0

Mahaudens P, 2009, EUR SPINE J, V18, P512, DOI 10.1007/s00586-009-0899-7

McDonald CM, 2005, ARCH PHYS MED REHAB, V86, P793, DOI 10.1016/j.apmr.2004.10.011

Morton A, 2008, J PEDIATR ORTHOPED, V28, P336, DOI 10.1097/BPO.0b013e318168d154

Mudge S, 2008, CLIN REHABIL, V22, P871, DOI 10.1177/0269215508092822

Muller C, 2010, DEUT Z SPORTMED, V61, P11

Muller C, 2010, BIOMED TECH, V55, P117, DOI 10.1515/BMT.2010.026

Negrini S, 2010, COCHRANE DB SYST REV, DOI 10.1002/14651858.CD006850.pub2

Nicholson G P, 2002, Stud Health Technol Inform, V91, P372

Nicholson GP, 2003, SPINE, V28, P2243, DOI 10.1097/01.BRS.0000085098.69522.52

Niemeyer T, 2009, SPINE, V34, P818, DOI 10.1097/BRS.0b013e31819b33be

Pham V-M, 2007, Annales de Readaptation et de Medecine Physique, V50, P125, DOI 10.1016/j.annrmp.2006.11.003

Poolman RW, 2002, EUR SPINE J, V11, P561, DOI 10.1007/s00586-002-0418-6

Rahman T, 2005, J PEDIATR ORTHOPED, V25, P420, DOI 10.1097/01.bpo.0000161097.61586.bb

Richards BS, 2005, SPINE, V30, P2068, DOI 10.1097/01.brs.0000178819.90239.d0

Rowe DE, 1997, J BONE JOINT SURG AM, V79A, P664, DOI 10.2106/00004623-199705000-00005

Sawamura C, 2008, CLIN ORTHOP RELAT R, V466, P1302, DOI 10.1007/s11999-008-0231-6

Schulte TL, 2010, EUR SPINE J, V19, P1855, DOI 10.1007/s00586-010-1324-y

Takemitsu M, 2004, SPINE, V29, P2070, DOI 10.1097/01.brs.0000138280.43663.7b

Trost S G, 2001, Exerc Sport Sci Rev, V29, P32, DOI 10.1097/00003677-200101000-00007

Tudor-Locke C, 2009, RES Q EXERCISE SPORT, V80, P164, DOI 10.1080/02701367.2009.10599550

Tudor-Locke CE, 2001, RES Q EXERCISE SPORT, V72, P1

Uhlenbrock K, 2008, DEUT Z SPORTMED, V59, P228

Vandal S, 1999, Issues Compr Pediatr Nurs, V22, P59

Weiss HR, 2006, SCOLIOSIS SPINAL DIS, V1, DOI [10.1186/1748-7161-1-5, 10.1186/1748-7161-1-1]

Weiss HR, 2003, ORTHOPADE, V32, P146, DOI 10.1007/s00132-002-0430-x

WEISS HR, 1995, Z ORTHOP GRENZGEB, V133, P114, DOI 10.1055/s-2008-1039421

Wong MS, 2008, GAIT POSTURE, V27, P189, DOI 10.1016/j.gaitpost.2007.03.007

NR 46

TC 21

Z9 23

U1 0

U2 17

PU SPRINGER

PI NEW YORK

PA 233 SPRING ST, NEW YORK, NY 10013 USA

SN 0940-6719

J9 EUR SPINE J

JI Eur. Spine J.

PD JUL

PY 2011

VL 20

IS 7

BP 1127

EP 1136

DI 10.1007/s00586-011-1791-9

PG 10

WC Clinical Neurology; Orthopedics

WE Science Citation Index Expanded (SCI-EXPANDED)

SC Neurosciences & Neurology; Orthopedics

GA 792LN

UT WOS:000292746500016

PM 21479852

OA Green Published

DA 2023-08-10

ER

PT J

AU Zhang, JT

Wang, DY

Chen, ZQ

Gao, J

Yu, XC

Sun, HN

Li, M

AF Zhang, Jingtao

Wang, Dayi

Chen, Ziqiang

Gao, Juan

Yu, Xiuchun

Sun, Haining

Li, Ming

TI Decrease of Self-Concept in Adolescent Patients With Mild to Moderate

Scoliosis After Conservative Treatment

SO SPINE

LA English

DT Article

DE conservative treatment; mental health; mild to moderate scoliosis;

self-concept scale

ID VALIDITY

AB Study Design. Comparative study.

Objective. To analyze the effect of conservative treatment on self-concept in patients with mild to moderate scoliosis.

Summary of Background Data. The choice of surgery or conservative treatment in adolescent idiopathic scoliosis is usually done on the basis of the magnitude of the initial Cobb angle in these patients. However, mental effect of the therapy choice should be considered.

Methods. Between August 2006 and December 2008, 65 patients with adolescent idiopathic scoliosis were selected for this study. Twenty-two patients with Cobb angles between 20 degrees and 40 degrees received conservative treatment, 18 patients with Cobb angles between 40 degrees and 50 degrees received conservative treatment, and 25 patients with Cobb angles between 40 degrees and 50 degrees received surgical treatment. All subjects were required to fill the Children's Self-Concept Scale at the beginning of the study and at the follow-up visit 1 year later. Scores on this scale were compared among the three different groups, and between the initial visit and the follow-up visit in each group.

Results. At the initial test, the total self-concept score was significantly higher in the group of patients with Cobb angles between 20 degrees and 40 degrees than in the two groups with Cobb angles between 40 degrees and 50 degrees. At the follow-up visit, the total self-concept score had increased significantly in the surgically treated group (Cobb angle between 40 degrees and 50 degrees), however, it had decreased in the two conservatively treated groups. No significant difference was seen between the two conservatively treated groups in the amount by which the self-concept score had decreased at follow-up.

Conclusion. In terms of mental health, conservative treatment is not ideal for patients with mild to moderate scoliosis, and in particular, it is not conducive to mental health in patients with Cobb angles between 40 degrees and 50 degrees.

C1 [Chen, Ziqiang; Li, Ming] Second Mil Med Univ, Dept Orthopaed Surg, Affiliated Changhai Hosp, Shanghai, china.

[Zhang, Jingtao; Yu, Xiuchun; Sun, Haining] Gen Hosp Jinan Mil Commanding Reg, Dept Orthopaed Surg, Jinan, china.

[Wang, Dayi] Affiliated Hosp, Yuyang Med Sch, Dept Spinal Surg, Shiyan, Hubei, china.

[Gao, Juan] S China Normal Univ, Dept Psychol, Guangzhou, Guangdong, china.

C3 Naval Medical University; South China Normal University

RP Li, M (通讯作者)， Changhai Hosp, Dept Orthopaed Surg, 174 Changhai Rd, Shanghai 200433, china.

EM buyning@yahoo.com.cn

RI Zhang, Jing/HII-4294-2022; Zhang, Jing/GWZ-7332-2022

CR Alexopoulos DS, 2002, PSYCHOL REP, V91, P827, DOI 10.2466/PR0.91.7.827-838

[Anonymous], 1996, PERSONALITY ANAL INT

[Anonymous], FDN SOCIAL BEHAV

HAN FL, 2004, SICHUAN MENT HLTH, V17, P129

Huebner ES, 1999, SOC INDIC RES, V46, P1, DOI 10.1023/A:1006821510832

Liu Huijun, 2000, CHIN J CLIN PSYCHOL, V8, P48

Marsh HW, 2004, PSYCHOL ASSESSMENT, V16, P27, DOI 10.1037/1040-3590.16.1.27

Piers E. V., 2002, PIERS HARRIS CHILDRE

SPONSELLE PD, 2002, SHANDONG MED J, V42, P50

SU LY, 2002, CHIN MENT HLTH J, V1, P31

WANG HM, 2005, MATERN CHILD HLTH CA, V20, P2681

[王弘 Wang Hong], 2003, [中国生物化学与分子生物学报, Chinese Journal of Biochemistry and Molecular Biology], V19, P77

ZHOU K, 2003, CHIN J SCH HLTH, V24, P204

海涌, 2003, [中国脊柱脊髓杂志, Chinese Journal of Spine and Spinal Cord], V13, P312

NR 14

TC 4

Z9 6

U1 0

U2 9

PU LIPPINCOTT WILLIAMS & WILKINS

PI PHILADELPHIA

PA TWO COMMERCE SQ, 2001 MARKET ST, PHILADELPHIA, PA 19103 USA

SN 0362-2436

EI 1528-1159

J9 SPINE

JI SPINE

PD JUL 1

PY 2011

VL 36

IS 15

BP E1004

EP E1008

DI 10.1097/BRS.0b013e3181fef60f

PG 5

WC Clinical Neurology; Orthopedics

WE Science Citation Index Expanded (SCI-EXPANDED); Social Science Citation Index (SSCI)

SC Neurosciences & Neurology; Orthopedics

GA 780JS

UT WOS:000291852500004

PM 21289553

OA Green Accepted

DA 2023-08-10

ER

PT J

AU Canavese, F

Kaelin, A

AF Canavese, Federico

Kaelin, Andre

TI Adolescent idiopathic scoliosis: Indications and efficacy of

nonoperative treatment

SO INDIAN JOURNAL OF ORTHOPAEDICS

LA English

DT Review

DE Brace; conservative treatment; scoliosis; adolescents

ID CHARLESTON BENDING BRACE; QUALITY-OF-LIFE; BOSTON BRACE; CONSERVATIVE

TREATMENT; MILWAUKEE BRACE; FOLLOW-UP; CURVE PROGRESSION;

RESEARCH-SOCIETY; BACK PAIN; ORTHOSIS

AB The strategy for the treatment of idiopathic scoliosis depends essentially upon the magnitude and pattern of the deformity, and its potential for progression. Treatment options include observation, bracing and/or surgery. During the past decade, several studies have demonstrated that the natural history of adolescent idiopathic scoliosis can be positively affected by nonoperative treatment, especially bracing. Other forms of conservative treatment, such as chiropractic or osteopathic manipulation, acupuncture, exercise or other manual treatments, or diet and nutrition, have not yet been proven to be effective in controlling spinal deformity progression, and those with a natural history that is favorable at the completion of growth. Observation is appropriate treatment for small curves, curves that are at low risk of progression, and those with a natural history that is favorable at the completion of growth. Indications for brace treatment are a growing child presenting with a curve of 25 degrees-40 degrees or a curve less than 25 with documented progression. Curves of 20 degrees-25 degrees in patients with pronounced skeletal immaturity should also be treated. The purpose of this review is to provide information about conservative treatment of adolescent idiopathic scoliosis. Indications for conservative treatment, hours daily wear and complications of brace treatment as well as brace types are discussed.

C1 [Canavese, Federico] Univ Hosp Geneva, CH-1211 Geneva 14, Switzerland.

Fac Med, Pediat Orthopaed Serv, Dept Child & Adolescent, CH-1211 Geneva, Switzerland.

C3 University of Geneva; University of Geneva

RP Canavese, F (通讯作者)， Univ Hosp Geneva, Rue Willy Donze 6, CH-1211 Geneva 14, Switzerland.

EM canavese_federico@yahoo.fr

RI Kaelin, Andre/AAU-2577-2021

OI CANAVESE, Federico/0000-0002-6114-5372

CR Allington NJ, 1996, J BONE JOINT SURG AM, V78A, P1056, DOI 10.2106/00004623-199607000-00010

Asher MA, 2006, SCOLIOSIS SPINAL DIS, V1, DOI 10.1186/1748-7161-1-2

Barrios C, 2005, SPINE, V30, P1610, DOI 10.1097/01.brs.0000169447.55556.01

BETZ RR, 1987, J BONE JOINT SURG AM, V69A, P90, DOI 10.2106/00004623-198769010-00015

Bunge EM, 2010, SPINE, V35, P57, DOI 10.1097/BRS.0b013e3181bdeaa6

CARR WA, 1980, J BONE JOINT SURG AM, V62, P599, DOI 10.2106/00004623-198062040-00015

CASSELLA MC, 1991, PHYS THER, V71, P897, DOI 10.1093/ptj/71.12.897

CHENEAU J, 1990, ORTHESE SCOLIOSE

CHONG KC, 1981, J PEDIATR ORTHOPED, V1, P251, DOI 10.1097/01241398-198111000-00002

Coillard C, 2003, EUR SPINE J, V12, P141, DOI 10.1007/s00586-002-0467-x

Coillard C, 2007, J PEDIATR ORTHOPED, V27, P375, DOI 10.1097/01.bpb.0000271330.64234.db

d'Amato CR, 2001, SPINE, V26, P2006

Danielsson AJ, 2001, SPINE, V26, P1449, DOI 10.1097/00007632-200107010-00015

Dolan LA, 2007, SPINE, V32, pS91, DOI 10.1097/BRS.0b013e318134ead9

EMANS JB, 1986, SPINE, V11, P792, DOI 10.1097/00007632-198610000-00009

Fayssoux RS, 2010, CLIN ORTHOP RELAT R, V468, P654, DOI 10.1007/s11999-009-0888-5

FEDERICO DJ, 1990, SPINE, V15, P886, DOI 10.1097/00007632-199009000-00009

FERNANDEZFELIBERTI R, 1995, J PEDIATR ORTHOPED, V15, P176

Gepstein R, 2002, J PEDIATR ORTHOPED, V22, P84, DOI 10.1097/00004694-200201000-00018

Gotze C, 2002, SPINE, V27, P1456, DOI 10.1097/00007632-200207010-00016

GREEN NE, 1986, J BONE JOINT SURG AM, V68A, P738, DOI 10.2106/00004623-198668050-00016

HIDALGO AH, 1993, THESIS U SEVILLA SEV

Howard A, 1998, SPINE, V23, P2404, DOI 10.1097/00007632-199811150-00009

JACKSON RP, 1983, SPINE, V8, P749, DOI 10.1097/00007632-198310000-00011

Janicki JA, 2007, J PEDIATR ORTHOPED, V27, P369, DOI 10.1097/01.bpb.0000271331.71857.9a

Katz DE, 1997, SPINE, V22, P1302, DOI 10.1097/00007632-199706150-00005

Landauer Franz, 2003, Pediatr Rehabil, V6, P201

Lange JE, 2009, SCOLIOSIS SPINAL DIS, V4, DOI 10.1186/1748-7161-4-17

LONSTEIN JE, 1984, J BONE JOINT SURG AM, V66A, P1061, DOI 10.2106/00004623-198466070-00013

LONSTEIN JE, 1994, J BONE JOINT SURG AM, V76A, P1207, DOI 10.2106/00004623-199408000-00011

Maruyama T, 2008, DISABIL REHABIL, V30, P786, DOI 10.1080/09638280801889782

Matsunaga S, 2005, SPINE, V30, P547, DOI 10.1097/01.brs.0000154648.53535.52

NACHEMSON AL, 1995, J BONE JOINT SURG AM, V77A, P815, DOI 10.2106/00004623-199506000-00001

Negrini S, 2007, Eura Medicophys, V43, P171

Negrini S, 2006, STUD HEALTH TECHNOL, V123, P245

Noonan KJ, 1997, J PEDIATR ORTHOPED, V17, P712, DOI 10.1097/00004694-199711000-00004

Noonan KJ, 1996, J BONE JOINT SURG AM, V78A, P557, DOI 10.2106/00004623-199604000-00009

O'Neill PJ, 2005, J BONE JOINT SURG AM, V87A, P1069, DOI 10.2106/JBJS.C.01707

OLAFSSON Y, 1995, J PEDIATR ORTHOPED, V15, P524, DOI 10.1097/01241398-199507000-00023

Pehrsson K, 2001, THORAX, V56, P388, DOI 10.1136/thorax.56.5.388

PELTONEN J, 1988, ACTA ORTHOP SCAND, V59, P487, DOI 10.3109/17453678809148769

PRICE CT, 1990, SPINE, V15, P1294, DOI 10.1097/00007632-199012000-00011

Price CT, 1997, J PEDIATR ORTHOPED, V17, P703, DOI 10.1097/00004694-199711000-00002

Rigo M, 2003, Pediatr Rehabil, V6, P209

RIGO M, 2004, PEDIAT REHABILITATIO, V7, P63

Rowe DE, 1997, J BONE JOINT SURG AM, V79A, P664, DOI 10.2106/00004623-199705000-00005

STAGNARA P, 1977, ACTUALITES REEDUCATI

Stagnara P, 1960, REV CHIR ORTHOP, V46, P562

Tones M, 2006, SPINE, V31, P3027, DOI 10.1097/01.brs.0000249555.87601.fc

Trivedi JM, 2001, J PEDIATR ORTHOPED, V21, P277, DOI 10.1097/00004694-200105000-00002

WATTS HG, 1977, CLIN ORTHOP RELAT R, P87

Weiss Hans-Rudolf, 2003, Pediatr Rehabil, V6, P183

Weiss Hans-Rudolf, 2003, Pediatr Rehabil, V6, P111, DOI 10.1080/13638490310001593446

Weiss HR, 2008, SCOLIOSIS SPINAL DIS, V3, DOI 10.1186/1748-7161-3-4

Weiss HR, 2003, ORTHOPADE, V32, P146, DOI 10.1007/s00132-002-0430-x

Wiley JW, 2000, SPINE, V25, P2326, DOI 10.1097/00007632-200009150-00010

Wong MS, 2008, SPINE, V33, P1360, DOI 10.1097/BRS.0b013e31817329d9

Yrjonen T, 2006, EUR SPINE J, V15, P1139, DOI 10.1007/s00586-005-0049-9

NR 58

TC 24

Z9 26

U1 0

U2 45

PU MEDKNOW PUBLICATIONS

PI MUMBAI

PA B-9, KANARA BUSINESS CENTRE, OFF LINK RD, GHAKTOPAR-E, MUMBAI, 400075, INDIA

SN 0019-5413

J9 INDIAN J ORTHOP

JI Indian J. Orthop.

PD JAN-FEB

PY 2011

VL 45

IS 1

BP 7

EP 14

DI 10.4103/0019-5413.73655

PG 8

WC Orthopedics

WE Science Citation Index Expanded (SCI-EXPANDED)

SC Orthopedics

GA 692KK

UT WOS:000285151500005

PM 21221217

OA Green Published

DA 2023-08-10

ER

PT J

AU Martinez-Llorens, J

Ramirez, M

Colomina, MJ

Bago, J

Molina, A

Caceres, E

Gea, J

AF Martinez-Llorens, J.

Ramirez, M.

Colomina, M. J.

Bago, J.

Molina, A.

Caceres, E.

Gea, J.

TI Muscle dysfunction and exercise limitation in adolescent idiopathic

scoliosis

SO EUROPEAN RESPIRATORY JOURNAL

LA English

DT Article

DE Lung function in disease; respiratory muscle function; skeletal muscle

dysfunction; spinal deformity

ID OBSTRUCTIVE PULMONARY-DISEASE; THORACIC SCOLIOSIS; PREDICTION EQUATIONS;

ADULTS; STRENGTH; VALUES; DYNAMOMETRY; ENDURANCE; CAPACITY; SYSTEM

AB Adolescent idiopathic scoliosis (AIS) can lead to ventilatory restriction, respiratory muscle weakness and exercise limitation. However, both the causes and the extent of muscle dysfunction remain unclear. The aim of our study is to describe muscle weakness and its relationship to lung function and tolerance to exercise in AIS patients.

Lung and muscle function, together with exercise capacity, were assessed in 60 patients with pronounced spinal deformity (>40 degrees) and in 25 healthy volunteers.

Patients with AIS had only mild to moderate abnormal ventilatory patterns, the most frequent of which were restrictive abnormalities. The function of respiratory and limb muscles and exercise capacity were below normal limits in AIS patients, and were significantly lower than in controls. Exercise capacity was found to correlate with the function of inspiratory, expiratory, upper limb and lower limb muscles which, in addition, were reciprocally interrelated. Multivariate analysis showed that lower limb muscle function is the main contributor to exercise intolerance. There appeared to be no connection between spinal deformity and lung function, muscle function or exercise capacity.

We conclude that AIS patients show generalised muscle dysfunction which contributes to the reduction in their exercise capacity, even in the absence of severe ventilatory impairment.

C1 [Martinez-Llorens, J.; Gea, J.] Univ Pompeu Fabra, Serv Pneumol, Hosp Mar,ISC 3, URMAR,IMIM,CIBER Enfermedades Resp CIBERES, Barcelona 08003, Spain.

[Ramirez, M.; Molina, A.; Caceres, E.] Univ Autonoma Barcelona, Hosp Mar, Serv COT, IMIM, E-08193 Barcelona, Spain.

[Bago, J.] Univ Autonoma Barcelona, Hosp Vall Hebron, Serv COT, E-08193 Barcelona, Spain.

[Colomina, M. J.] Univ Autonoma Barcelona, Hosp Vall Hebron, Serv Anestesia, E-08193 Barcelona, Spain.

C3 CIBER - Centro de Investigacion Biomedica en Red; CIBERES; Institut

Hospital del Mar d'Investigacions Mediques (IMIM); Hospital del Mar;

Pompeu Fabra University; Autonomous University of Barcelona; Institut

Hospital del Mar d'Investigacions Mediques (IMIM); Hospital del Mar;

Autonomous University of Barcelona; Hospital Universitari Vall d'Hebron;

Autonomous University of Barcelona; Hospital Universitari Vall d'Hebron

RP Gea, J (通讯作者)， Univ Pompeu Fabra, Serv Pneumol, Hosp Mar,ISC 3, URMAR,IMIM,CIBER Enfermedades Resp CIBERES, Pg Maritim 25-27, Barcelona 08003, Spain.

EM jgea@imim.es

RI Bago, Joan/C-4222-2014; Bagó, Joan/AAD-5072-2022; Gea, Joaquim/E-4841-2014; Colomina, Maria J/C-4207-2014; Molina, A/G-5246-2014

OI Bago, Joan/0000-0001-9359-8088; Bagó, Joan/0000-0001-9359-8088; Gea, Joaquim/0000-0001-8718-7346; Colomina, Maria J/0000-0003-0106-0956; Molina, A/0000-0002-7570-4569; Caceres, Enric/0000-0001-7372-557X; Ramirez, Manuel/0000-0003-2628-0074

FU Fondo de Invstigacions Sanitarias Proyecto de Investigacion [070194]; Sociedad Espanola de Neumologia y Cirugia Toracica (SEPAR); Societat Catalana de Pneumologia (SOCAP); Institut Municipal d'Investigacio Medica; CIBERES (ISC III); FIS, Spain [PI 07-0194]

FX J. Martinez-Llorens was funded by grants from Fondo de Invstigacions Sanitarias Proyecto de Investigacion 070194, Sociedad Espanola de Neumologia y Cirugia Toracica (SEPAR), Societat Catalana de Pneumologia (SOCAP) and the Institut Municipal d'Investigacio Medica (Ajuts per Residents (Research Fellowhsips for Residents) 2002 and 2003). The study was funded in part by CIBERES (ISC III) and FIS PI 07-0194, Spain.

CR Alotaibi Saad, 2008, J Med Case Rep, V2, P171, DOI 10.1186/1752-1947-2-171

[Anonymous], 1988, CLIN EXERCISE TESTIN

Barrios C, 2005, SPINE, V30, P1610, DOI 10.1097/01.brs.0000169447.55556.01

BLACK LF, 1969, AM REV RESPIR DIS, V99, P696

Bohannon RW, 1997, ARCH PHYS MED REHAB, V78, P26, DOI 10.1016/S0003-9993(97)90005-8

Boyer J, 1996, CHEST, V109, P1532, DOI 10.1378/chest.109.6.1532

BYLUND P, 1987, CLIN ORTHOP RELAT R, P222

COBB JR, 1948, INSTRUCTIONAL COURSE, V5, P61

Coronell C, 2004, EUR RESPIR J, V24, P129, DOI 10.1183/09031936.04.00079603

DICKSON JH, 1995, J BONE JOINT SURG AM, V77A, P513, DOI 10.2106/00004623-199504000-00003

DOSSANTOS L, 2006, CHEST, V130, P500

Estenne M, 1998, AM J RESP CRIT CARE, V158, P452, DOI 10.1164/ajrccm.158.2.9710116

GEA J, 1988, EUR RESPIR J, V1, P109

GEA J, 2006, SKELETAL MUSCLE PLAS, P315

Giordano A, 1997, NUCL MED COMMUN, V18, P105, DOI 10.1097/00006231-199702000-00003

HEPPER NNG, 1965, AM REV RESPIR DIS, V91, P365

HERMAN R, 1985, SPINE, V10, P1, DOI 10.1097/00007632-198501000-00001

KEARON C, 1993, AM REV RESPIR DIS, V148, P295, DOI 10.1164/ajrccm/148.2.295

KEARON C, 1993, AM REV RESPIR DIS, V148, P288, DOI 10.1164/ajrccm/148.2.288

Kim YJ, 2007, SPINE, V32, P2685, DOI 10.1097/BRS.0b013e31815a7b17

Koumbourlis AC, 2006, PAEDIATR RESPIR REV, V7, P152, DOI 10.1016/j.prrv.2006.04.009

Laghi F, 2003, AM J RESP CRIT CARE, V168, P10, DOI 10.1164/rccm.2206020

LISBOA C, 1985, AM REV RESPIR DIS, V132, P48

LOW WD, 1983, CLIN ORTHOP RELAT R, P217

Lowe TG, 2000, J BONE JOINT SURG AM, V82A, P1157, DOI 10.2106/00004623-200008000-00014

Luna-Heredia E, 2005, CLIN NUTR, V24, P250, DOI 10.1016/j.clnu.2004.10.007

Machida M, 1996, SPINE, V21, P1147, DOI 10.1097/00007632-199605150-00005

Mannion A F, 1998, Eur Spine J, V7, P289, DOI 10.1007/s005860050077

Mantovani G, 2001, DRUGS, V61, P499, DOI 10.2165/00003495-200161040-00004

MARTINEZLLORENS JM, 2005, EUR RESP J S49, V29, pS686

MATHIOWETZ V, 1985, ARCH PHYS MED REHAB, V66, P69

Meier MP, 1997, SPINE, V22, P2357, DOI 10.1097/00007632-199710150-00008

Morales P, 1997, Arch Bronconeumol, V33, P213

Negrini S, 2008, J REHABIL MED, V40, P451, DOI 10.2340/16501977-0195

Newton PO, 2005, J BONE JOINT SURG AM, V87A, P1937, DOI 10.2106/JBJS.D.02209

Parent Stefan, 2005, Instr Course Lect, V54, P529

Ramirez-Sarmiento A, 2002, AM J RESP CRIT CARE, V166, P1491, DOI 10.1164/rccm.200202-075OC

Ramirez-Sarmiento A, 2002, THORAX, V57, P132, DOI 10.1136/thorax.57.2.132

Reamy BV, 2001, AM FAM PHYSICIAN, V64, P111

Roca J, 1998, RESP MED, V92, P454, DOI 10.1016/S0954-6111(98)90291-8

Roca J, 1998, EUR RESPIR J, V11, P1354, DOI 10.1183/09031936.98.11061354

ROCA J, 1990, AM REV RESPIR DIS, V141, P1026, DOI 10.1164/ajrccm/141.4_Pt_1.1026

SAHGAL V, 1983, ACTA ORTHOP SCAND, V54, P242, DOI 10.3109/17453678308996564

Shelton Yvonne A, 2007, Adolesc Med State Art Rev, V18, P121

SMYTH RJ, 1984, THORAX, V39, P901, DOI 10.1136/thx.39.12.901

STONE B, 1979, PHYS THER, V59, P759, DOI 10.1093/ptj/59.6.759

Swallow EB, 2009, EUR RESPIR J, V34, P1429, DOI 10.1183/09031936.00074008

Takahashi S, 2007, SPINE, V32, P106, DOI 10.1097/01.brs.0000251005.31255.25

TRONTELJ JV, 1988, MUSCLE NERVE, V11, P297, DOI 10.1002/mus.880110404

Upadhyay S S, 1995, Eur Spine J, V4, P274, DOI 10.1007/BF00301033

UPADHYAY SS, 1995, SPINE, V20, P2415, DOI 10.1097/00007632-199511001-00008

WARREN MP, 1986, NEW ENGL J MED, V314, P1348, DOI 10.1056/NEJM198605223142104

Wasserman K, 1999, PRINCIPLES EXERCISE, P63

Weinstein SL, 2008, LANCET, V371, P1527, DOI 10.1016/S0140-6736(08)60658-3

Weiss HR, 2009, STUD HEALTH TECHNOL, V135, P164

WINTER RB, 1986, NEW ENGL J MED, V314, P1379, DOI 10.1056/NEJM198605223142108

World Medical Association General Assembly, 2004, J Int Bioethique, V15, P124

NR 57

TC 67

Z9 75

U1 2

U2 9

PU EUROPEAN RESPIRATORY SOC JOURNALS LTD

PI SHEFFIELD

PA 442 GLOSSOP RD, SHEFFIELD S10 2PX, united kingdom

SN 0903-1936

EI 1399-3003

J9 EUR RESPIR J

JI Eur. Resp. J.

PD AUG

PY 2010

VL 36

IS 2

BP 393

EP 400

DI 10.1183/09031936.00025509

PG 8

WC Respiratory System

WE Science Citation Index Expanded (SCI-EXPANDED)

SC Respiratory System

GA 647EY

UT WOS:000281601800025

PM 20032022

OA Bronze

DA 2023-08-10

ER

PT J

AU Schmid, AB

Dyer, L

Boni, T

Held, U

Brunner, F

AF Schmid, Annina B.

Dyer, Linda

Boeni, Thomas

Held, Ulrike

Brunner, Florian

TI Paraspinal Muscle Activity During Symmetrical and Asymmetrical Weight

Training in Idiopathic Scoliosis

SO JOURNAL OF SPORT REHABILITATION

LA English

DT Article

DE exercise therapy; resistance training; low back pain; physical therapy

ID ELECTROMYOGRAPHY; CURVE; SIZE

AB Context: Various studies report decreased muscle activation in the concavity of the curve in patients with scoliosis. Such decreased muscle-performance capacity could lead to sustained postural deficits. Objective: To investigate whether specific asymmetrical sports therapy exercises rather than symmetrical back strengthening can increase EMG amplitudes of paraspinal muscles in the concavity of the curve. Design: Cross-sectional. Setting: Laboratory. Participants: 16 patients with idiopathic

scoliosis. Interventions: Patients performed 4 back-strengthening exercises (front press, lat pull-down, roman chair, bent-over barbell row) during 1 test session. Each exercise was performed in a symmetrical and asymmetrical variant and repeated 3 times. Main Outcome Measure: EMG amplitudes of the paraspinal muscles were recorded in the thoracic and lumbar apexes of the scoliotic curve during each exercise. Ratios of convex-to concave-side EMG activity were calculated. Results: Statistical analysis revealed that the asymmetrical variants of front press at the lumbar level (P = .002) and roman chair and bent-over barbell row at the thoracic level (P < .0001, .001 respectively) were superior in increasing EMG amplitudes in the concavity of the scoliotic curve. Conclusions: Specific asymmetrical exercises increase EMG amplitudes of paraspinal muscles in the concavity. If confirmed in longitudinal studies measuring improvements of postural deficits, these exercises may advance care of patients with scoliosis.

C1 [Schmid, Annina B.; Dyer, Linda] Balgrist Univ Hosp, Dept Physiotherapy, Zurich, Switzerland.

[Boeni, Thomas] Balgrist Univ Hosp, Dept Prosthesis & Orthot, Zurich, Switzerland.

[Brunner, Florian] Balgrist Univ Hosp, Dept Rheumatol, Zurich, Switzerland.

[Held, Ulrike] Univ Zurich, Horten Ctr Patient Oriented Res, Zurich, Switzerland.

C3 University of Zurich; University of Zurich; University of Zurich;

University of Zurich

RP Schmid, AB (通讯作者)， Balgrist Univ Hosp, Dept Physiotherapy, Zurich, Switzerland.

RI Held, Ulrike/O-8328-2019; Schmid, Annina B/F-3070-2010; Held,

Ulrike/D-3666-2013

OI Held, Ulrike/0000-0003-3105-5840; Schmid, Annina B/0000-0001-7759-0211;

FU Swiss Physiotherapy Association

FX We wish to thank the Swiss Physiotherapy Association for funding this

research project. The technical assistance and advice of Hubertus van

Hedel from the Spinal Cord Injury Center, Balgrist University Hospital

Zurich, Switzerland is greatly appreciated.

CR ALEXANDER MA, 1978, ARCH PHYS MED REHAB, V59, P314

Avikainen VJ, 1999, J SPINAL DISORD, V12, P61

Chan YL, 1999, PEDIATR RADIOL, V29, P360, DOI 10.1007/s002470050607

Cheung J, 2006, SPINE, V31, P322, DOI 10.1097/01.brs.0000197155.68983.d8

Cheung J, 2005, EUR SPINE J, V14, P130, DOI 10.1007/s00586-004-0780-7

Drake JDM, 2006, J ELECTROMYOGR KINES, V16, P175, DOI 10.1016/j.jelekin.2005.07.003

Gaudreault N, 2005, BMC MUSCULOSKEL DIS, V6, DOI 10.1186/1471-2474-6-14

Gram MC, 1999, SPINE, V24, P169, DOI 10.1097/00007632-199901150-00019

KENNELLY KP, 1993, SPINE, V18, P913, DOI 10.1097/00007632-199306000-00017

LEHNERTSCHROTH CH, 2000, DREIDIMENSIONALE SKO

Mannion A F, 1998, Eur Spine J, V7, P289, DOI 10.1007/s005860050077

Mannion AF, 1997, J ANAT, V190, P505, DOI 10.1046/j.1469-7580.1997.19040505.x

McIntire KL, 2008, J SPINAL DISORD TECH, V21, P349, DOI 10.1097/BSD.0b013e318145b7e9

Mooney V, 2000, J SPINAL DISORD, V13, P102, DOI 10.1097/00002517-200004000-00002

Odermatt D, 2003, J ORTHOP RES, V21, P931, DOI 10.1016/S0736-0266(03)00038-X

Reamy BV, 2001, AM FAM PHYSICIAN, V64, P111

REDFERN MS, 1993, CLIN BIOMECH, V8, P44, DOI 10.1016/S0268-0033(05)80009-9

REDFORD J B, 1969, Archives of Physical Medicine and Rehabilitation, V50, P433

Schiller JR, 2008, SPORTS MED ARTHROSC, V16, P26, DOI 10.1097/JSA.0b013e3181629aa8

VALENTINO B, 1985, ANAT CLIN, V7, P55, DOI 10.1007/BF01654630

Weiss H R, 1993, Eur Spine J, V1, P240, DOI 10.1007/BF00298367

WEISS HR, 2006, BEFUNDGERECHTE PHYSI

ZETTERBERG C, 1983, SPINE, V8, P447

NR 23

TC 18

Z9 19

U1 4

U2 26

PU HUMAN KINETICS PUBL INC

PI CHAMPAIGN

PA 1607 N MARKET ST, PO BOX 5076, CHAMPAIGN, IL 61820-2200 USA

SN 1056-6716

J9 J SPORT REHABIL

JI J. Sport Rehabil.

PD AUG

PY 2010

VL 19

IS 3

BP 315

EP 327

DI 10.1123/jsr.19.3.315

PG 13

WC Rehabilitation; Sport Sciences

WE Science Citation Index Expanded (SCI-EXPANDED)

SC Rehabilitation; Sport Sciences

GA 636YH

UT WOS:000280780500006

PM 20811080

DA 2023-08-10

ER

PT J

AU Bruneau, A

AF Bruneau, A.

TI Trauma gravity and psychosocial pain in athlete in emergency unit

SO SCIENCE & SPORTS

LA French

DT Article

DE Athletic injuries; Psychology; Health status indicator; Abbreviated

injury scale; Epidemiology

ID ZEALAND RUGBY INJURY; QUALITY-OF-LIFE; PHYSICAL-ACTIVITY; HEALTH

PROFILE; PERFORMANCE PROJECT; RUNNING INJURIES; RISK-FACTORS;

EPIDEMIOLOGY; PREVENTION; DISORDERS

AB Subject. - We aimed to explore a correlation between psychosocial. or psychological factors and athletic injury gravity.

Method. - Prospective case control study by questionnaire collecting sociodemographic data, Duke scores, and psychosocial. items in 205 patients aged 27,3 (+/-9,5), admitted for athletic injury in emergency unit of the hospital of Cholet. Analysis has compared psychosocial factors between two groups composed of lower gravity trauma and higher gravity trauma made by the abbreviated injury scale (AIS).

Results. - The proportion of patients with severe alteration of social Duke score (under 55) was higher (P < 0,05) in the group with lower gravity trauma (AIS = 1) than in the group with higher gravity (AIS >= 2). This relation with mental Duke score was not found. Psychosocial difficulties seem to increase benign injury. (C) 2009 Elsevier Masson SAS. All rights reserved.

C1 CHU Angers, Lab Explorat Fonct Effort, Ctr Reg Med Sport, F-49100 Angers, France.

C3 Universite d'Angers; Centre Hospitalier Universitaire d'Angers

RP Bruneau, A (通讯作者)， CHU Angers, Lab Explorat Fonct Effort, Ctr Reg Med Sport, 4 Rue Larrey, F-49100 Angers, France.

EM antoine.bruneau@etud.univ-angers.fr

CR Alla F, 2002, EUR J HEART FAIL, V4, P337, DOI 10.1016/S1388-9842(02)00006-5

Arnason A, 2004, AM J SPORT MED, V32, p5S, DOI 10.1177/0363546503258912

*ASS ADV AUT MED C, 1998, ABBR INJ SCAL

Baum AL, 2005, CLIN SPORT MED, V24, P853, DOI 10.1016/j.csm.2005.06.006

Bertrais S, 2005, OBES RES, V13, P936, DOI 10.1038/oby.2005.108

Bird YN, 1998, BRIT J SPORT MED, V32, P319, DOI 10.1136/bjsm.32.4.319

BOURDREAUX ED, 2008, GEN HOSP PSYCHIAT, V30, P4

Briancon S, 1997, ARCH MAL COEUR VAISS, V90, P1577

Brooks JHM, 2008, CLIN SPORT MED, V27, P51, DOI 10.1016/j.csm.2007.09.001

Burns J, 2003, J ORTHOP SPORT PHYS, V33, P177, DOI 10.2519/jospt.2003.33.4.177

CAMACHO TC, 1991, AM J EPIDEMIOL, V134, P220, DOI 10.1093/oxfordjournals.aje.a116074

Colburn NT, 2003, INJURY, V34, P207, DOI 10.1016/S0020-1383(02)00039-6

*CONST ORG MOND SA, 2006, DOC FOND S

Ekstrand J, 2008, SCI SPORT, V23, P73, DOI 10.1016/j.scispo.2007.10.012

Filaire E, 2008, SCI SPORT, V23, P49, DOI 10.1016/j.scispo.2007.10.009

Finch C, 1998, BRIT J SPORT MED, V32, P220, DOI 10.1136/bjsm.32.3.220

Galambos SA, 2005, BRIT J SPORT MED, V39, P351, DOI 10.1136/bjsm.2005.018440

Grossbard JR, 2007, J STUD ALCOHOL DRUGS, V68, P566, DOI 10.15288/jsad.2007.68.566

Guillemin F, 2001, JOINT BONE SPINE, V68, P499, DOI 10.1016/S1297-319X(01)00314-1

Guillemin F, 1997, Sante Publique, V9, P35

Hercberg S, 2008, INT J PUBLIC HEALTH, V53, P68, DOI 10.1007/s00038-008-7016-2

HOEBERIGS JH, 1992, SPORTS MED, V13, P408, DOI 10.2165/00007256-199213060-00004

Huang JH, 2007, J ADOLESCENT HEALTH, V40, P390, DOI 10.1016/j.jadohealth.2006.11.146

HUNT SM, 1981, SOC SCI MED-MED SOC, V15, P221, DOI 10.1016/0271-7123(81)90005-5

Kim PTW, 2006, J TRAUMA, V60, P312, DOI 10.1097/01.ta.0000202714.31780.5f

Kofotolis ND, 2007, AM J SPORT MED, V35, P458, DOI 10.1177/0363546506294857

Made C, 2001, KNEE SURG SPORT TR A, V9, P386, DOI 10.1007/s001670100229

Neuner Bruno, 2005, Health Qual Life Outcomes, V3, P77, DOI 10.1186/1477-7525-3-77

PAFFENBARGER RS, 1994, ACTA PSYCHIAT SCAND, V89, P16, DOI 10.1111/j.1600-0447.1994.tb05796.x

PARKERSON GR, 1990, MED CARE, V28, P1056, DOI 10.1097/00005650-199011000-00007

PARKERSON GR, 1981, MED CARE, V19, P806, DOI 10.1097/00005650-198108000-00002

PATE RR, 1995, JAMA-J AM MED ASSOC, V273, P402, DOI 10.1001/jama.273.5.402

Peluso Marco Aurélio Monteiro, 2005, Clinics, V60, P61, DOI 10.1590/S1807-59322005000100012

Quarrie KL, 2001, BRIT J SPORT MED, V35, P157, DOI 10.1136/bjsm.35.3.157

Saliou V, 2005, GEN HOSP PSYCHIAT, V27, P263, DOI 10.1016/j.genhosppsych.2005.03.009

Scully D, 1998, BRIT J SPORT MED, V32, P111, DOI 10.1136/bjsm.32.2.111

Taunton JE, 2003, BRIT J SPORT MED, V37, P239, DOI 10.1136/bjsm.37.3.239

Torjussen J, 2006, BRIT J SPORT MED, V40, P230, DOI 10.1136/bjsm.2005.021329

Ueland O, 1998, BRIT J SPORT MED, V32, P299, DOI 10.1136/bjsm.32.4.299

Ytterstad B, 1996, BRIT J SPORT MED, V30, P64, DOI 10.1136/bjsm.30.1.64

NR 40

TC 0

Z9 1

U1 0

U2 2

PU ELSEVIER FRANCE-EDITIONS SCIENTIFIQUES MEDICALES ELSEVIER

PI ISSY-LES-MOULINEAUX

PA 65 RUE CAMILLE DESMOULINS, CS50083, 92442 ISSY-LES-MOULINEAUX, FRANCE

SN 0765-1597

J9 SCI SPORT

JI Sci. Sports

PD NOV

PY 2009

VL 24

IS 5

BP 238

EP 245

DI 10.1016/j.scispo.2009.01.009

PG 8

WC Sport Sciences

WE Science Citation Index Expanded (SCI-EXPANDED); Social Science Citation Index (SSCI)

SC Sport Sciences

GA 540VE

UT WOS:000273367300003

DA 2023-08-10

ER

PT J

AU Kenanidis, E

Potoupnis, ME

Papavasiliou, KA

Sayegh, FE

Kapetanos, GA

AF Kenanidis, Eustathios

Potoupnis, Michael E.

Papavasiliou, Kyriakos A.

Sayegh, Fares E.

Kapetanos, George A.

TI Adolescent idiopathic scoliosis and exercising - Is there truly a

liaison?

SO SPINE

LA English

DT Article

DE adolescent idiopathic scoliosis; exercising; adolescent idiopathic

scoliosis and exercising; sports

ID BACK-PAIN; PREVALENCE

AB Study Design. Cross-sectional observational study. Objective. Evaluation and comparison of the prevalence of adolescent idiopathic scoliosis (AIS) among 2 groups of patients (athletes and nonathletes) to determine whether athletic activities are related to the development of AIS.

Summary of Background Data. The potential association between AIS and exercising remains uncertain. The latter has often been considered as a therapeutic means and a causative factor of the former.

Methods. A group of 2387 adolescents (boys: 1177, girls: 1210, mean age: 13.4 years) was evaluated. All completed a questionnaire concerning personal, somatometric, and secondary sex characteristics, type, duration and character of daily-performed physical activities, and existing cases of AIS among relatives. Patients were classified into 2 groups according to their answers; "athletes" and "nonathletes." The groups were comparable as far as age, height, weight, onset of menstruation, family history of scoliosis, and side of handedness were concerned. Children underwent physical examination by 3 orthopedic surgeons who were unaware of their level of athletic activities. Children considered, by all, to be suspicious of suffering from scoliosis, underwent further radiographic evaluation.

Results. In 99 cases (athletes: 48, nonathletes: 51), AIS was radiographically confirmed (Cobb angle > 10 degrees). No statistically significant difference was found between athlete and nonathlete adolescents ($P = 0.842$), athlete and nonathlete boys ($P = 0.757$), and athlete and nonathlete girls ($P = 0.705$), as far as the prevalence of AIS was concerned. The mean value of the Cobb angle of the main scoliotic curve was not statistically different between male athletes and nonathletes ($P = 0.45$) and female athletes and nonathletes ($P = 0.707$). With the Cobb threshold reset at 20, no statistically significant differences were detected either.

Conclusion. Our results demonstrate that systematic exercising is probably not associated with the development of AIS. Actively participating in sports activities doesn't seem to affect the degree of the main scoliotic curve either.

C1 Aristotle Univ Thessaloniki, Sch Med, Dept Orthopaed, Papageorgiou Gen Hosp, GR-54006 Thessaloniki, Greece.

C3 Aristotle University of Thessaloniki; Papageorgiou Hospital

RP Kenanidis, E (通讯作者)，7 Anoikseos Str, Thessaloniki 57010, Greece.

EM Kena76@otenet.gr

RI Kenanidis, Eustathios/AAS-3030-2021

OI Kenanidis, Eustathios/0000-0001-9517-1600; Papavasiliou,

Kyriakos/0000-0001-7341-2469

CR [Anonymous], INT RES SOC SPIN DEF

Beausejour M, 2007, SPINE, V32, P1349, DOI 10.1097/BRS.0b013e318059b5f7

BECKER TJ, 1986, CLIN SPORT MED, V5, P149

Bono CM, 2004, J BONE JOINT SURG AM, V86A, P382, DOI 10.2106/00004623-200402000-00027

CARMAN D, 1985, J PEDIATR ORTHOPED, V5, P65, DOI 10.1097/01241398-198501000-00011

DRUMMOND DS, 1980, SPINE, V5, P507, DOI 10.1097/00007632-198011000-00004

Grivas Theodoros B, 2002, Stud Health Technol Inform, V91, P71

HELLSTROM M, 1990, ACTA RADIOL, V31, P127

Holschen JC, 2004, SOUTH MED J, V97, P852, DOI 10.1097/01.SMJ.0000140124.83000.40

Kapetanos G, 2002, Stud Health Technol Inform, V91, P7

Karachalios T, 1999, SPINE, V24, P2318, DOI 10.1097/00007632-199911150-00006

Klentrou P, 2003, BRIT J SPORT MED, V37, P490, DOI 10.1136/bjsm.37.6.490

Kocher MS, 2006, J BONE JOINT SURG AM, V88A, P1412, DOI 10.2106/JBJS.F.00442

Kono K, 2000, J Orthop Surg (Hong Kong), V8, P19

Machida M, 1999, SPINE, V24, P2576, DOI 10.1097/00007632-199912150-00004

McMaster M., 2006, J BONE JOINT SURG BR, V88, P225

Omey ML, 2000, CLIN ORTHOP RELAT R, P74

Potoupnis M, 2002, Stud Health Technol Inform, V91, P10

Prapas N, 1989, Acta Eur Fertil, V20, P315

Ramirez N, 1997, J BONE JOINT SURG AM, V79A, P364, DOI 10.2106/00004623-199703000-00007

Smith FM, 2002, J BONE JOINT SURG BR, V84B, P392, DOI 10.1302/0301-620X.84B3.12619

Soucacos PN, 1997, J BONE JOINT SURG AM, V79A, P1498, DOI 10.2106/00004623-199710000-00006

Tanchev PI, 2000, SPINE, V25, P1367, DOI 10.1097/00007632-200006010-00008

van Rhijn L W, 2001, Spine (Phila Pa 1976), V26, pE373, DOI 10.1097/00007632-200108150-00021

van Rhijn LW, 2001, ACTA ORTHOP SCAND, V72, P621, DOI 10.1080/000164701317269058

WARREN MP, 1986, NEW ENGL J MED, V314, P1348, DOI 10.1056/NEJM198605223142104

Wong HK, 2005, SPINE, V30, P1188, DOI 10.1097/01.brs.0000162280.95076.bb

NR 27

TC 25

Z9 27

U1 2

U2 22

PU LIPPINCOTT WILLIAMS & WILKINS

PI PHILADELPHIA

PA TWO COMMERCE SQ, 2001 MARKET ST, PHILADELPHIA, PA 19103 USA

SN 0362-2436

EI 1528-1159

J9 SPINE

JI SPINE

PD SEP 15

PY 2008

VL 33

IS 20

BP 2160

EP 2165

DI 10.1097/BRS.0b013e31817d6db3

PG 6

WC Clinical Neurology; Orthopedics

WE Science Citation Index Expanded (SCI-EXPANDED)

SC Neurosciences & Neurology; Orthopedics

GA 351CP

UT WOS:000259402400005

PM 18794756

DA 2023-08-10

ER

PT J

AU McIntire, KL

Asher, MA

Burton, DC

Liu, W

AF McIntire, Kevin L.

Asher, Marc A.

Burton, Douglas C.

Liu, Wen

TI Treatment of adolescent idiopathic scoliosis with quantified trunk

rotational strength training - A pilot study

SO JOURNAL OF SPINAL DISORDERS & TECHNIQUES

LA English

DT Article

DE adolescent idiopathic scoliosis; strength training; trunk rotational

strength; strength asymmetry

ID LEAN BODY-WEIGHT; BACK MUSCLES; CURVE PROGRESSION; ANTHROPOMETRIC ESTIMATION; PARAVERTEBRAL MUSCLES; PLATELET CALMODULIN; MULTIFIDUS MUSCLE; LUMBAR FUNCTION; ASYMMETRY; BRACE

AB Study Design: Prospective clinical trial.

Objectives: To test the hypothesis that quantified trunk rotational strength training will equalize any strength asymmetry, increase strength overall, and stabilize adolescent idiopathic scoliosis.

Summary of Background Data: Bracing, the only generally accepted form of adolescent idiopathic scoliosis nonoperative therapy, has many shortcomings. Paraspinal muscle abnormalities, which have been extensively documented in these patients, are generally considered to be secondary. A normal female's trunk strength in flexion and extension decreases from her juvenile to adolescent years, whereas a male's increases.

Methods: Patients received a 4-month supervised followed by a 4-month home trunk rotational strength training program. Trunk rotational strength was measured in both directions at 5 positions at baseline, 4 months, and 8 months. The patients were followed clinically.

Results: Fifteen patients (12 females and 3 males), with an average age of 13.9 years and an average main Cobb of 33 degrees were enrolled. At baseline there was no significant asymmetry. After 4 months of supervised strength training, involving an average of 32 training sessions, each lasting about 25 minutes, their strength had significantly increased by 28%, to 50% ($P < 0.005$ to $P < 0.001$). After 4 months of unsupervised home strength training their strengths were unchanged. The 3 patients with baseline curves of 50 to 60 degrees all had main or compensatory curve progression and 2 had surgery. For patients with 20 to 40-degree curves, survivorship from main curve progression of >= 6 degrees was 100%, at 8 months, but decreased to 64% at 24 months.

Conclusions: Quantified trunk rotational strength training significantly increased strength. It was not effective for curves measuring 50 to 60 degrees. It appeared to help stabilize curves in the 20 to 40-degree ranges for 8 months, but not for 24 months. Periodic additional supervised strength training may help the technique to remain effective, although additional experimentation will be necessary to determine this.

C1 [Asher, Marc A.; Burton, Douglas C.] Univ Kansas, Med Ctr, Dept Orthoped Surg, Kansas City, KS 66160 USA.

[McIntire, Kevin L.; Liu, Wen] Univ Kansas, Med Ctr, Dept Phys Therapy & Rehabil Sci, Kansas City, KS 66160 USA.

C3 University of Kansas; University of Kansas Medical Center; University of Kansas; University of Kansas Medical Center

RP Asher, MA (通讯作者)， Univ Kansas, Med Ctr, Dept Orthoped Surg, 3901 Rainbow Blvd,Mail Stop 3017, Kansas City, KS 66160 USA.

EM masher@kume.edu

CR Andersson EA, 2002, SPINE, V27, pE152, DOI 10.1097/00007632-200203150-00014

Avikainen VJ, 1999, J SPINAL DISORD, V12, P61

BANKOFF AD, 2000, CLIN NEUROPHYSIOL, V40, P345

Barton E, 2003, J GERONTOL A-BIOL, V58, P923

Burwell R G, 1992, Acta Orthop Belg, V58 Suppl 1, P33

BYLUND P, 1987, CLIN ORTHOP RELAT R, V214, P222

CASSELLA MC, 1991, PHYS THER, V71, P897, DOI 10.1093/ptj/71.12.897

Chan YL, 1999, PEDIATR RADIOL, V29, P360, DOI 10.1007/s002470050607

Cheung J, 2006, SPINE, V31, P322, DOI 10.1097/01.brs.0000197155.68983.d8

Cobb JR., 1948, INSTR COURSE LECT, V5, P261

Davis JR, 2000, SPINE, V25, P180, DOI 10.1097/00007632-200001150-00007

Dickson RA, 1999, J BONE JOINT SURG BR, V81B, P193, DOI 10.1302/0301-620X.81B2.9630

FIDLER MW, 1976, J BONE JOINT SURG BR, V58, P200, DOI 10.1302/0301-620X.58B2.932082

Fidler MW, 1974, SCOLIOSIS MUSCLE, P184

FORD DM, 1988, SPINE, V13, P461, DOI 10.1097/00007632-198805000-00004

Goldberg CJ, 2001, SPINE, V26, P42, DOI 10.1097/00007632-200101010-00009

Hansen L, 2006, SPINE, V31, P1888, DOI 10.1097/01.brs.0000229232.66090.58

Jonsson B, 1970, Electromyography, V10, P5

KAPLAN EL, 1958, J AM STAT ASSOC, V53, P457, DOI 10.2307/2281868

KENNELLY KP, 1993, SPINE, V18, P913, DOI 10.1097/00007632-199306000-00017

KHOSLA S, 1980, J NEUROL SCI, V46, P13, DOI 10.1016/0022-510X(80)90040-4

KINDSFATER K, 1994, J BONE JOINT SURG AM, V76A, P1186, DOI 10.2106/00004623-199408000-00009

LeBlanc R, 1998, SPINE, V23, P1109, DOI 10.1097/00007632-199805150-00007

LeBlanc R, 1997, SPINE, V22, P2532, DOI 10.1097/00007632-199711010-00013

Lee LJ, 2005, SPINE, V30, P870, DOI 10.1097/01.brs.0000158956.77897.ec

LEXELL J, 1993, CAN J APPL PHYSIOL, V18, P2, DOI 10.1139/h93-002

LINDH M, 1978, Spine, V3, P313, DOI 10.1097/00007632-197812000-00003

Little DG, 2000, J BONE JOINT SURG AM, V82A, P685, DOI 10.2106/00004623-200005000-00009

Loeb GE, 2000, PRINCIPLES NEURAL SC, V380, P674

LONSTEIN JE, 1984, J BONE JOINT SURG AM, V66A, P1061, DOI 10.2106/00004623-198466070-00013

LONSTEIN JE, 1994, J BONE JOINT SURG AM, V76A, P1207, DOI 10.2106/00004623-199408000-00011

Lowe T, 2002, SPINE, V27, P768, DOI 10.1097/00007632-200204010-00016

MACINTOSH JE, 1993, AUST NZ J SURG, V63, P205, DOI 10.1111/j.1445-2197.1993.tb00520.x

Mannion A F, 1998, Eur Spine J, V7, P289, DOI 10.1007/s005860050077

MARRAS WS, 1995, SPINE, V20, P1440, DOI 10.1097/00007632-199507000-00002

MAYER TG, 1985, SPINE, V10, P912, DOI 10.1097/00007632-198512000-00009

MCGILL SM, 1992, SPINE, V17, P1187, DOI 10.1097/00007632-199210000-00010

MCINTIRE K, 2007, ISOKINET EX IN PRESS

McIntire KL, 2007, SCOLIOSIS SPINAL DIS, V2, DOI 10.1186/1748-7161-2-9

Meier MP, 1997, SPINE, V22, P2357, DOI 10.1097/00007632-199710150-00008

Mooney V, 2000, J SPINAL DISORD, V13, P102, DOI 10.1097/00002517-200004000-00002

Mooney V, 2003, ORTHOPEDICS, V26, P167

Moseley G Lorimer, 2002, Spine (Phila Pa 1976), V27, pE29, DOI 10.1097/00007632-200201150-00013

Musacchia X J, 1988, Exerc Sport Sci Rev, V16, P61

NACHEMSON AL, 1995, J BONE JOINT SURG AM, V77A, P815, DOI 10.2106/00004623-199506000-00001

Negrini Stefano, 2003, Pediatr Rehabil, V6, P227, DOI: 10.1080/13638490310001636781

Ng JKF, 2001, J ORTHOPAED RES, V19, P463, DOI 10.1016/S0736-0266(00)90027-5

OGILVIE JW, 1994, MOES TXB SCOLIOSIS O, P95

POPE MH, 1987, SPINE, V12, P1041, DOI 10.1097/00007632-198712000-00016

PORTILLO D, 1982, SPINE, V7, P551, DOI 10.1097/00007632-198211000-00007

ROGALA EJ, 1978, J BONE JOINT SURG AM, V60, P173, DOI 10.2106/00004623-197860020-00005

Rowe DE, 1997, J BONE JOINT SURG AM, V79A, P664, DOI 10.2106/00004623-199705000-00005

Shandsjr AR, 1941, J BONE JOINT SURG AM, V23, P963

Shimode M, 2003, SPINE, V28, P2535, DOI 10.1097/01.BRS.0000092375.61922.D1

SLAGER UT, 1987, J PEDIATR ORTHOPED, V7, P301, DOI 10.1097/01241398-198705000-00011

SMITH SS, 1985, SPINE, V10, P757, DOI 10.1097/00007632-198510000-00011

SPENCER GSG, 1976, J NEUROL SCI, V30, P137, DOI 10.1016/0022-510X(76)90261-6

Stokes IAF, 2003, J ELECTROMYOGR KINES, V13, P397, DOI 10.1016/S1050-6411(03)00046-4

Stokes IAF, 1996, SPINE, V21, P1162, DOI 10.1097/00007632-199605150-00007

SUNNEGARDH J, 1988, EUR J APPL PHYSIOL O, V58, P291, DOI 10.1007/BF00417265

The Terminology Committee of the Scoliosis Research Society has compiled the present publication, 1976, SPINE, V1, P57

THOMASON DB, 1990, J APPL PHYSIOL, V68, P1, DOI 10.1152/jappl.1990.68.1.1

Toren A, 2001, APPL ERGON, V32, P583, DOI 10.1016/S0003-6870(01)00040-0

Weiss H R, 1997, Pediatr Rehabil, V1, P35

WILMORE JH, 1969, J APPL PHYSIOL, V27, P25, DOI 10.1007/BF00695015

WILMORE JH, 1970, AM J CLIN NUTR, V23, P267

YAROM R, 1979, Spine, V4, P12, DOI 10.1097/00007632-197901000-00003

YAROM R, 1979, ISRAEL J MED SCI, V15, P917

ZETTERBERG C, 1984, ACTA ORTHOP SCAND, V55, P304, DOI 10.3109/17453678408992362

ZETTERBERG C, 1983, SPINE, V8, P457, DOI 10.1097/00007632-198307000-00003

NR 70

TC 20

Z9 22

U1 3

U2 17

PU LIPPINCOTT WILLIAMS & WILKINS

PI PHILADELPHIA

PA TWO COMMERCE SQ, 2001 MARKET ST, PHILADELPHIA, PA 19103 USA

SN 1536-0652

EI 1539-2465

J9 J SPINAL DISORD TECH

JI J. Spinal Disord. Tech.

PD JUL

PY 2008

VL 21

IS 5

BP 349

EP 358

DI 10.1097/BSD.0b013e318145b7e9

PG 10

WC Clinical Neurology; Orthopedics

WE Science Citation Index Expanded (SCI-EXPANDED)

SC Neurosciences & Neurology; Orthopedics

GA 324UC

UT WOS:000257543900010

PM 18600146

DA 2023-08-10

ER

PT J

AU Negrini, S

Atanasio, S

Zaina, F

Romano, M

AF Negrini, S.

Atanasio, S.

Zaina, F.

Romano, M.

TI Rehabilitation of adolescent idiopathic scoliosis: results of exercises

and bracing from a series of clinical studies - Europa

Medicophysica-SIMFER 2007 Award Winner

SO EUROPEAN JOURNAL OF PHYSICAL AND REHABILITATION MEDICINE

LA English

DT Article

DE rehabilitation; scoliosis; adolescent; exercise

ID SOSORT CONSENSUS PAPER; SEAS.02 EXERCISES; PROGRESSION; EFFICACY; SPORT

AB Aim. Rehabilitation of adolescent idiopathic scoliosis (AIS) requires a careful choice from among the possible treatments, such as bracing and exercises, according to the patient's needs. According to the literature, there is little evidence regarding the efficacy of these rehabilitation

instruments. During the past few years, a full series of studies has been carried out to investigate their efficacy. The aim of this paper was to summarize all these results.

Methods. Three systematic reviews (two on exercises and one on manual therapy), and four cohort prospective studies were performed. The prospective studies included two trials with a prospective control group on exercises (one to avoid bracing and one in preparation to bracing) and two trials with retrospective control group on a new brace developed by the Authors (Sforzesco brace and SPoRT concept of correction versus Lyon brace and Risser cast).

Results. Results show that in literature there is proof of level 1b on exercises but no studies on manual therapy. High quality exercises like Scientific Exercises Approach to Scoliosis (SEAS) have more efficacy than usual physiotherapy, significantly reducing brace prescription in one year from 25% of cases to 6%. Moreover, such exercises help to obtain the best results in bracing first correction. The Sforzesco brace has proved to have more efficacy than the Lyon brace, whereas it has the same efficacy - but reduced side effects and impact on quality of life - than the Risser brace.

Conclusion. With an efficient management of data collection, it is possible to develop a set of studies aimed at verifying the efficacy of clinical daily rehabilitation approaches.

C1 [Negrini, S.; Atanasio, S.; Zaina, F.; Romano, M.] ISICO, Italian Sci Spine Inst, I-20141 Milan, Italy.

RP Negrini, S (通讯作者)， ISICO, Italian Sci Spine Inst, Via Bellarmino 13-1, I-20141 Milan, Italy.

EM stefano.negrini@isico.it

RI Zaina, Fabio/H-3261-2013; Negrini, Stefano/B-6667-2013

OI Zaina, Fabio/0000-0002-1256-5362; Negrini, Stefano/0000-0002-1878-2747

CR BROOKS VB, 1983, PHYS THER, V63, P664, DOI 10.1093/ptj/63.5.664

Goldberg CJ, 2001, SPINE, V26, P42, DOI 10.1097/00007632-200101010-00009

Grivas TB, 2007, SCOLIOSIS SPINAL DIS, V2, DOI 10.1186/1748-7161-2-17

HENATSCH HD, 1985, INT J SPORTS MED, V6, P2, DOI 10.1055/s-2008-1025805

IOFE ME, 2004, NEUROSCI BEHAV PHYSL, V34, P5

NEGRINI A, 1989, JOURN GROUP KIN TRAV

NEGRINI A, 1992, RIABILITAZIONE OGGI, V9, P11

NEGRINI A, 1987, GINNASTICA MED, V1, P58

NEGRINI A, 2006, 3 INT C CONS MAN SPI

Negrini S, 2007, Eura Medicophys, V43, P171

Negrini S, 2005, Eura Medicophys, V41, P183

NEGRINI S, 2007, 4 INT C CONS MAN SPI

NEGRINI S, 2008, DISABIL RAH IN PRESS

NEGRINI S, 2008, DISABIL REH IN PRESS

NEGRINI S, 2007, EURA MEDICOPHYS, V43, P183

NEGRINI S, 1996, 2 M INT SOC STUD RES, P68

Negrini S., 2007, EVIDENCE BASED ISICO

Negrini Stefano, 2007, J Surg Orthop Adv, V16, P98

Negrini S, 2006, ST HEAL T, V123, P523

Negrini S, 2006, ST HEAL T, V123, P519

Negrini S, 2006, STUD HEALTH TECHNOL, V123, P245

Negrini S, 2006, SCOLIOSIS SPINAL DIS, V1, DOI 10.1186/1748-7161-1-4

Negrini Stefano, 2003, Pediatr Rehabil, V6, P227, DOI: 10.1080/13638490310001636781

Rigo M, 2006, SCOLIOSIS SPINAL DIS, V1, DOI 10.1186/1748-7161-1-11

ROMANO M, 2006, 3 INT C CONS MAN SPI

ROMANO M, 2007, 4 INT C CONS MAN SPI

ROMANO M, 2006, SOSORT SOC SCOL ORTH

Romano M, 2006, SCOLIOSIS SPINAL DIS, V1, DOI 10.1186/1748-7161-1-12

Shindle Michael K, 2006, J Surg Orthop Adv, V15, P43

SIBILLA P, 2001, DEFORMITA VERTEBRALI, V2, P20

SIBILLA P, 2002, RACHIDE RIABILITAZIO, V1, P73

Weiss HR, 2006, SCOLIOSIS SPINAL DIS, V1, DOI [10.1186/1748-7161-1-5, 10.1186/1748-7161-1-1]

Weiss HR, 2006, SCOLIOSIS SPINAL DIS, V1, DOI 10.1186/1748-7161-1-6

WINTER RB, 2006, JSOA, V15, P43

Winter Robert B, 2006, J Surg Orthop Adv, V15, P184

NR 35

TC 18

Z9 20

U1 0

U2 10

PU EDIZIONI MINERVA MEDICA

PI TURIN

PA CORSO BRAMANTE 83-85 INT JOURNALS DEPT., 10126 TURIN, ITALY

SN 1973-9087

EI 1973-9095

J9 EUR J PHYS REHAB MED

JI Eur. J. Phys. Rehabil. Med.

PD JUN

PY 2008

VL 44

IS 2

BP 169

EP 176

PG 8

WC Rehabilitation

WE Science Citation Index Expanded (SCI-EXPANDED)

SC Rehabilitation

GA 314EJ

UT WOS:000256791900010

PM 18418337

DA 2023-08-10

ER

PT J

AU Negrini, S

Zaina, F

Romano, M

Negrini, A

Parzini, S

AF Negrini, Stefano

Zaina, Fabio

Romano, Michele

Negrini, Alessandra

Parzini, Silvana

TI Specific exercises reduce brace prescription in adolescent idiopathic

scoliosis: A prospective controlled cohort study with worst-case

analysis

SO JOURNAL OF REHABILITATION MEDICINE

LA English

DT Article

DE idiopathic scoliosis; physical exercise; physical therapy; brace

ID PROGRAM

AB Objective: To compare the effect of Scientific Exercises Approach to Scoliosis (SEAS) exercises with "usual care" rehabilitation programmes in terms of the avoidance of brace prescription and prevention of curve progression in adolescent idiopathic scoliosis.

Design: Prospective controlled cohort study observational study.

Patients: Seventy-four consecutive outpatients with adolescent idiopathic scoliosis, mean 15 degrees (standard deviation 6) Cobb angle, 12.4 (standard deviation 2.2) years old, at risk of bracing who had not been treated previously.

Methods: Thirty-five patients were included in the SEAS exercises group and 39 in the usual physiotherapy group. The primary outcome included the number of braced patients, Cobb angle and the angle of trunk rotation.

Results: There were 6.1% braced patients in the SEAS exercises group vs 25.0% in the usual physiotherapy group. Failures of treatment in the worst-case analysis were 11.5% and 30.8%, respectively. In both cases the differences were statistically significant. Cobb angle improved in the SEAS exercises group, but worsened in the usual physiotherapy group. In the SEAS exercises group, 23.5% of patients improved and 11.8% worsened, while in the usual physiotherapy group 11.1% improved and 13.9% worsened.

Conclusion: These data confirm the effectiveness of exercises in patients with scoliosis who are at high risk of progression. Compared with non-adapted exercises, a specific and personalized treatment (SEAS) appears to be more effective.

C1 [Negrini, Stefano; Zaina, Fabio; Romano, Michele] ISICO, Italian Sci Spine Inst, IT-20141 Milan, Italy.

[Negrini, Alessandra; Parzini, Silvana] ISICO, Ctr Negrini, Vigevano, PV, Italy.

RP Negrini, S (通讯作者)，ISICO, Italian Sci Spine Inst, Via Roberto Bellarmino 13-1, IT-20141 Milan, Italy.

EM stefano.negrini@isico.it

RI Zaina, Fabio/H-3261-2013; Negrini, Stefano/B-6667-2013

OI Zaina, Fabio/0000-0002-1256-5362; Negrini, Stefano/0000-0002-1878-2747

CR Andersen MO, 2002, J PEDIATR ORTHOP B, V11, P96, DOI 10.1097/00009957-200204000-00002

[Anonymous], P INT C PREV SCOL SC

Athanasopoulos S, 1999, SCAND J MED SCI SPOR, V9, P36

Dolan LA, 2007, SPINE, V32, pS91, DOI 10.1097/BRS.0b013e318134ead9

Ducong P, 2002, RESONANCE EUROPEENNE, V10, P1229

EDGAR M, 2003, BRACE WEAR COMPLIANC

Ferraro C., 1998, EUR MEDICOPHYS, V34, P25

Freidel K, 2002, SPINE, V27, pE87, DOI 10.1097/00007632-200202150-00013

Grosso C, 2002, Stud Health Technol Inform, V91, P123

HAWES MC, 2007, 4 INT C CONS MAN SPI

Lenssinck MLB, 2005, PHYS THER, V85, P1329, DOI 10.1093/ptj/85.12.1329

Lindeman M, 1999, J PEDIATR ORTHOPED, V19, P493, DOI 10.1097/00004694-199907000-00013

Mollon G, 1986, KINESITHERAPIE SCI, V244, P47

MOLLON G, ENCY MED CHIR

Mooney V, 2000, J SPINAL DISORD, V13, P102, DOI 10.1097/00002517-200004000-00002

NEGRINI A, 1992, RIABILITAZIONE OGGI, V9, P11

NEGRINI A, 2006, 3 INT C CONS MAN SPI

NEGRINI S, 1995, STUD HEALTH TECHNOL, V15, P209

Negrini S, 2005, Eura Medicophys, V41, P183

Negrini S., 2007, EVIDENCE BASED ISICO

Negrini S, 2004, PEDIAT REHABIL, V7, P52

Negrini S, 2006, ST HEAL T, V123, P523

Negrini S, 2006, SCOLIOSIS SPINAL DIS, V1, DOI 10.1186/1748-7161-1-14

Negrini S, 2007, PHYS THER, V87, P112, DOI 10.2522/ptj.2007.87.1.112

Negrini Stefano, 2003, Pediatr Rehabil, V6, P227, DOI: 10.1080/13638490310001636781

Nicholson G P, 2002, Stud Health Technol Inform, V91, P372

Reichel Dagmar, 2003, Pediatr Rehabil, V6, P221

Richards BS, 2005, SPINE, V30, P2068, DOI 10.1097/01.brs.0000178819.90239.d0

ROMANO M, 2004, 1 INT C CONS MAN SPI

SIBILLA P, 2001, DEFORMITA VERTEBRALI, P20

*SRS, 2004, DEF SCOL TERM

STAGNARA P, 1990, REEDUCATION SCOLIOSE

STONE B, 1979, PHYS THER, V59, P759, DOI 10.1093/ptj/59.6.759

Weiss HR, 2006, SCOLIOSIS SPINAL DIS, V1, DOI [10.1186/1748-7161-1-5, 10.1186/1748-7161-1-1]

Weiss Hans-Rudolf, 2003, Pediatr Rehabil, V6, P23, DOI 10.1080/1363849031000095288

WEISS HR, 1991, SPINE, V16, P88, DOI 10.1097/00007632-199101000-00016

Wong MS, 2001, PROSTHET ORTHOT INT, V25, P60, DOI 10.1080/03093640108726570

ZAINA F, 2007, 4 INT C CONS MAN SPI

Zmurko Matthew G, 2003, J Surg Orthop Adv, V12, P208

NR 39

TC 65

Z9 70

U1 1

U2 35

PU FOUNDATION REHABILITATION INFORMATION

PI UPPSALA

PA TRADGARDSGATAN 14, UPPSALA, SE-753 09, SWEDEN

SN 1650-1977

EI 1651-2081

J9 J REHABIL MED

JI J. Rehabil. Med.

PD JUN

PY 2008

VL 40

IS 6

BP 451

EP 455

DI 10.2340/16501977-0195

PG 5

WC Rehabilitation; Sport Sciences

WE Science Citation Index Expanded (SCI-EXPANDED)

SC Rehabilitation; Sport Sciences

GA 310MH

UT WOS:000256533200008

PM 18509560

OA gold, Green Published

DA 2023-08-10

ER

PT J

AU Weiss, HR

Goodall, D

AF Weiss, H. -R.

Goodall, D.

TI The treatment of adolescent idiopathic scoliosis (AIS) according to

present evidence - A systematic review

SO EUROPEAN JOURNAL OF PHYSICAL AND REHABILITATION MEDICINE

LA English

DT Review

DE scoliosis; adolescent; natural history; therapeutics

ID QUALITY-OF-LIFE; CHARLESTON BENDING BRACE; TERM-FOLLOW-UP;

COTREL-DUBOUSSET INSTRUMENTATION; SELECTIVE THORACIC FUSION; POSTERIOR

SPINAL-FUSION; SOSORT CONSENSUS PAPER; LEAST 20 YEARS; RESEARCH-SOCIETY;

NATURAL-HISTORY

AB Traditionally, the treatment options for adolescent idiopathic scoliosis (AIS), the most common form of scoliosis, are exercises; in-patient rehabilitation; braces and surgery. The outcomes of treatments are usually compared with the natural history or observation (nonintervention). The aim of this paper was to provide a synopsis of all treatment options in the light of evidence based practice (EBP). A systematic review was carried out using the most encompassing databases available. Literature has been searched for the outcome parameter "rate of progression" and only prospective controlled studies that have considered the treatment versus the natural history have been included. The search strategy included the following terms: "adolescent idiopathic scoliosis"; "idiopathic scoliosis"; "natural history"; "observation"; "physiotherapy"; "physical therapy"; "rehabilitation"; "bracing"; "orthotics" and"surgery". Prospective short-term studies have been found to support outpatient physiotherapy. One prospective controlled study was found to support scoliosis in-patient rehabilitation (SIR). One prospective multi-centre study, a long-term prospective controlled study and a metaanalysis have been found to support bracing. No controlled study, neither short, mid nor long-term, was found to reveal any substantial evidence to support surgery as a treatment for this condition. There is some evidence supporting the conservative treatment for AIS. No substantial evidence has been found in terms of prospective controlled studies to support surgical intervention. In light of the unknown long-term effects of surgery, a randomised controlled trial (RCT) seems necessary. Due to the presence of evidence to support conservative treatments, a plan to compose a RCT for conservative treatment options seems unethical. But it is also important to conclude that the evidence for conservative treatments is weak in number and length.

C1 [Weiss, H. -R.] Asklepios Katharina Schroth Spinal Deform Rehabil, D-55566 Bad Sobernheim, Germany.

[Goodall, D.] Ealing PCT London, Clayponds Hosp, London, united kingdom.

RP Weiss, HR (通讯作者)， Asklepios Katharina Schroth Spinal Deform Rehabil, Korczakstr 2, D-55566 Bad Sobernheim, Germany.

EM hr.weiss@asklepios.com

CR Abelson R., 2006, NY TIMES 1230

Ali RM, 2003, SPINE, V28, P1163, DOI 10.1097/00007632-200306010-00015

Andersen MO, 2006, SPINE, V31, P350, DOI 10.1097/01.brs.0000197649.29712.de

[Anonymous], P 11 INT C WORLD CON

[Anonymous], BERGEYS MANUAL SYSTE

ASCANI E, 1986, SPINE, V11, P784, DOI 10.1097/00007632-198610000-00007

Asher M, 2004, SPINE, V29, P2013, DOI 10.1097/01.brs.0000138275.49220.81

Asher M, 2003, SPINE, V28, P63, DOI 10.1097/00007632-200301010-00015

Asher MA, 2006, SCOLIOSIS SPINAL DIS, V1, DOI 10.1186/1748-7161-1-2

Aubin CE, 2007, EUR SPINE J, V16, P57, DOI 10.1007/s00586-006-0063-6

Barrios C, 2005, SPINE, V30, P1610, DOI 10.1097/01.brs.0000169447.55556.01

Benli IT, 2007, EUR SPINE J, V16, P381, DOI 10.1007/s00586-006-0147-3

BETZ RR, 1987, J BONE JOINT SURG AM, V69A, P90, DOI 10.2106/00004623-198769010-00015

Bjerkreim I, 2007, SPINE, V32, P2103, DOI 10.1097/BRS.0b013e318145a54a

BRANTHWAITE MA, 1986, BRIT J DIS CHEST, V80, P360, DOI 10.1016/0007-0971(86)90089-6

Bullmann V, 2004, Z ORTHOP GRENZGEB, V142, P403, DOI 10.1055/s-2004-822843

Bullmann V, 2003, SPINE, V28, P1306, DOI 10.1097/00007632-200306150-00016

Bunge EM, 2007, EUR SPINE J, V16, P83, DOI 10.1007/s00586-006-0097-9

Castro Frank P Jr, 2003, Spine J, V3, P180, DOI 10.1016/S1529-9430(02)00557-0

Chang JH, 2006, J SPINAL DISORD TECH, V19, P222, DOI 10.1097/01.bsd.0000168323.58576.2f

Cheng I, 2005, SPINE, V30, P2104, DOI 10.1097/01.brs.0000179261.70845.b7

Cobb JR., 1948, INSTR COURSE LECT, V5, P261

Coillard C, 2007, J PEDIATR ORTHOPED, V27, P375, DOI 10.1097/01.bpb.0000271330.64234.db

COLLIS DK, 1969, J BONE JOINT SURG AM, VA 51, P425, DOI 10.2106/00004623-196951030-00001

Crigger NJ, 2007, J NURS SCHOLARSHIP, V39, P177, DOI 10.1111/j.1547-5069.2007.00164.x

d'Amato CR, 2001, SPINE, V26, P2006

Danielsson AJ, 2007, SPINE, V32, P2198, DOI 10.1097/BRS.0b013e31814b851f

Danielsson AJ, 2007, SPINE, V32, pS101, DOI 10.1097/BRS.0b013e318134ed0e

Danielsson AJ, 2001, SPINE, V26, P516

Danielsson AJ, 2003, SPINE, V28, pE373, DOI 10.1097/01.BRS.0000084267.41183.75

Danielsson AJ, 2001, EUR SPINE J, V10, P278, DOI 10.1007/s005860100309

Danielsson AJ, 2001, SPINE, V26, P1449, DOI 10.1097/00007632-200107010-00015

Danielsson AJ, 2006, SPINE, V31, P275, DOI 10.1097/01.brs.0000197652.52890.71

Danielsson AJ, 2001, ACTA RADIOL, V42, P187, DOI 10.1034/j.1600-0455.2001.042002187.x

den Boer WA, 1999, EUR SPINE J, V8, P406, DOI 10.1007/s005860050195

DICKSON JH, 1990, J BONE JOINT SURG AM, V72A, P678, DOI 10.2106/00004623-199072050-00006

DICKSON JH, 1995, J BONE JOINT SURG AM, V77A, P513, DOI 10.2106/00004623-199504000-00003

DICKSON RA, 1978, ACTA ORTHOP SCAND, V49, P46, DOI 10.3109/17453677809005722

Dobbs MB, 2006, SPINE, V31, P2386, DOI 10.1097/01.brs.0000238965.81013.c5

Dobbs MB, 2006, SPINE, V31, P2400, DOI 10.1097/01.brs.0000240212.31241.8e

Dobosiewlcz K, 2006, STUD HEALTH TECHNOL, V123, P267

Dolan LA, 2007, J PEDIATR ORTHOPED, V27, P270, DOI 10.1097/01.bpb.0000248579.11864.47

Dolan LA, 2007, SPINE, V32, pS91, DOI 10.1097/BRS.0b013e318134ead9

Edelmann P, 1992, Acta Orthop Belg, V58 Suppl 1, P85

EILEEN M, HEALTHCARE PURCHASIN

EMANS JB, 1986, SPINE, V11, P792, DOI 10.1097/00007632-198610000-00009

Fatyga Marek, 2005, Ortop Traumatol Rehabil, V7, P254

FEDER BJ, 2006, NY TIMES 0318

FERNANDEZFELIBERTI R, 1995, J PEDIATR ORTHOPED, V15, P176

Ferraro C., 1998, EUR MEDICOPHYS, V34, P25

FILLER AG, IS THERE ETHICAL CRI

Freidel K, 2002, ST HEAL T, V88, P24

Gepstein R, 2002, J PEDIATR ORTHOPED, V22, P84, DOI 10.1097/00004694-200201000-00018

Glowacki Maciej, 2005, Ortop Traumatol Rehabil, V7, P273

Goldberg CJ, 2008, PEDIATR SURG INT, V24, P129, DOI 10.1007/s00383-007-2016-5

Goldberg CJ, 2001, SPINE, V26, P42, DOI 10.1097/00007632-200101010-00009

GOLDBERG CJ, 1993, SPINE, V18, P902, DOI 10.1097/00007632-199306000-00015

GOLDBERG MS, 1994, SPINE, V19, P1562, DOI 10.1097/00007632-199407001-00004

GOLDBERG MS, 1994, SPINE, V19, P1551, DOI 10.1097/00007632-199407001-00003

Gotze C, 2002, Z ORTHOP GRENZGEB, V140, P492, DOI 10.1055/s-2002-34007

Gotze C, 2002, SPINE, V27, P1456, DOI 10.1097/00007632-200207010-00016

GRIVAS TB, 2005, P IMAST 7 9 JUL 2005

Grivas Theodoros B, 2003, Pediatr Rehabil, V6, P237

Haefeli M, 2006, SPINE, V31, P355, DOI 10.1097/01.brs.0000197664.02098.09

HAHER TR, 1995, SPINE, V20, P1575, DOI 10.1097/00007632-199507150-00005

Haher TR, 1999, SPINE, V24, P1435, DOI 10.1097/00007632-199907150-00008

Halm H, 2000, ORTHOPADE, V29, P563, DOI 10.1007/s001320050494

Hawes Martha C, 2003, Pediatr Rehabil, V6, P171

HAWES NI, 2006, PEDIAT REHABIL, V9, P318

HEINE J, 1985, Z ORTHOP GRENZGEB, V123, P323, DOI 10.1055/s-2008-1045158

Helenius I, 2003, J BONE JOINT SURG AM, V85A, P2303, DOI 10.2106/00004623-200312000-00006

Helenius I, 2002, SPINE, V27, P176, DOI 10.1097/00007632-200201150-00010

Hill D, 2002, Stud Health Technol Inform, V91, P477

HOPF C, 1989, ROFO FORTSCHR RONTG, V151, P311, DOI 10.1055/s-2008-1047184

HOPF C, 1985, Z ORTHOP GRENZGEB, V123, P312, DOI 10.1055/s-2008-1045157

Hurford RK, 2006, SPINE, V31, P2322, DOI 10.1097/01.brs.0000238966.75175.2b

JACKSON RP, 1983, SPINE, V8, P749, DOI 10.1097/00007632-198310000-00011

JAMES JIP, 1954, J BONE JOINT SURG BR, V36, P36, DOI 10.1302/0301-620X.36B1.36

JENG CL, 1993, SPINE, V18, P1584, DOI 10.1097/00007632-199309000-00003

KANE WJ, 1977, CLIN ORTHOP RELAT R, P43

Karol LA, 2001, SPINE, V26, P2001, DOI 10.1097/00007632-200109150-00013

Katz DE, 2001, SPINE, V26, P2354, DOI 10.1097/00007632-200111010-00012

Katz DE, 1997, SPINE, V22, P1302, DOI 10.1097/00007632-199706150-00005

Kim YJ, 2006, SPINE, V31, P291, DOI 10.1097/01.brs.0000197865.20803.d4

Kitayama S, 2004, PSYCHOL SCI, V15, P527, DOI 10.1111/j.0956-7976.2004.00714.x

Kleinberg S, 1922, ARCH SURG-CHICAGO, V5, P631, DOI 10.1001/archsurg.1922.01110150184008

KLISIC P, 1982, JOURN INT PREV SCOL

Koch KD, 2001, SPINE, V26, P2119, DOI 10.1097/00007632-200110010-00015

Kolban Maciej, 2005, Ortop Traumatol Rehabil, V7, P260

KOLINDSORENSEN V, 1973, ACTA ORTHOP SCAND, V44, P98

Kotani T, 2004, SPINE, V29, P298, DOI 10.1097/01.BRS.0000106490.82936.89

Kotwicki T, 2006, EUR SPINE J, V15, P972, DOI 10.1007/s00586-005-0991-6

Kuklo TR, 2007, SPINE, V32, P2258, DOI 10.1097/BRS.0b013e31814b1ba6

Kuklo TR, 2005, SPINE, V30, P2113, DOI 10.1097/01.brs.0000179260.73267.f4

Kunakornsawat Sombat, 2007, Journal of the Medical Association of Thailand, V90, P1443

Landauer Franz, 2003, Pediatr Rehabil, V6, P201

Landry C, 1998, ANN CHIR, V52, P784

Lenssinck MLB, 2005, PHYS THER, V85, P1329, DOI 10.1093/ptj/85.12.1329

Lonner BS, 2006, J BONE JOINT SURG AM, V88A, P1022, DOI 10.2106/JBJS.E.00001

LONSTEIN JE, 1984, J BONE JOINT SURG AM, V66A, P1061, DOI 10.2106/00004623-198466070-00013

LONSTEIN JE, 1994, J BONE JOINT SURG AM, V76A, P1207, DOI 10.2106/00004623-199408000-00011

Luhmann SJ, 2005, SPINE, V30, P2061, DOI 10.1097/01.brs.0000179299.78791.96

Machida M, 2004, J PEDIATR ORTHOPED, V24, P576, DOI 10.1097/01241398-200409000-00021

Mamyama Toni, 2002, Stud Health Technol Inform, V91, P361

MANKIN HJ, 1964, J BONE JOINT SURG AM, V46, P53, DOI 10.2106/00004623-196446010-00005

Mariconda M, 2005, EUR SPINE J, V14, P854, DOI 10.1007/s00586-004-0853-7

Maruyama T, 2006, SPINE, V31, P2310, DOI 10.1097/01.brs.0000238971.05671.d5

Maruyama Toru, 2003, Pediatr Rehabil, V6, P215

Maurtua M, 2005, J CLIN ANESTH, V17, P213, DOI 10.1016/j.jclinane.2004.06.006

MAYO NE, 1994, SPINE, V19, P1573, DOI 10.1097/00007632-199407001-00005

MCMASTER MJ, 1983, J BONE JOINT SURG BR, V65, P612, DOI 10.1302/0301-620X.65B5.6643567

MEHTA M H, 1972, Journal of Bone and Joint Surgery British Volume, V54, P230

Merola AA, 2002, SPINE, V27, P2046, DOI 10.1097/00007632-200209150-00015

MILLER NH, 2000, SPINE STATE ART REV, V14, P313

Min K, 2007, EUR SPINE J, V16, P65, DOI 10.1007/s00586-006-0075-2

Moen KY, 1999, SPINE, V24, P2570, DOI 10.1097/00007632-199912150-00003

Mollon G, 1986, KINESITHERAPIE SCI, V244, P47

Montgomery F, 1997, SPINE, V22, P772, DOI 10.1097/00007632-199704010-00012

Morningstar MW, 2004, BMC MUSCULOSKEL DIS, V5, DOI 10.1186/1471-2474-5-32

MOSES S, 1984, AESTHET PLAST SURG, V8, P213, DOI 10.1007/BF01570705

MOTULSKY AG, 1978, NEW ENGL J MED, V298, P1196, DOI 10.1056/NEJM197805252982111

Muschik MT, 2006, EUR SPINE J, V15, P1128, DOI 10.1007/s00586-005-0034-3

NACHEMSON A, 1979, SPINE, V4, P513, DOI 10.1097/00007632-197911000-00011

NACHEMSON A, 2000, ACTA ORTHOP SCAND, V39, P466

NACHEMSON AL, 1995, J BONE JOINT SURG AM, V77A, P815, DOI 10.2106/00004623-199506000-00001

Negrini S, 2007, Eura Medicophys, V43, P171

Negrini S, 2006, ST HEAL T, V123, P523

Negrini S, 2006, ST HEAL T, V123, P519

Negrini Stefano, 2003, Pediatr Rehabil, V6, P227, DOI: 10.1080/13638490310001636781

Niemeyer T, 2005, INT ORTHOP, V29, P47, DOI 10.1007/s00264-004-0599-1

Niemeyer T, 1999, Z ORTHOP GRENZGEB, V137, P430, DOI 10.1055/s-2008-1037386

NILSONNE U, 1968, ACTA ORTHOP SCAND, V39, P456, DOI 10.3109/17453676808989663

NISSINEN M, 1993, ACTA PAEDIATR, V82, P77, DOI 10.1111/j.1651-2227.1993.tb12521.x

Noonan KJ, 1996, J BONE JOINT SURG AM, V78A, P557, DOI 10.2106/00004623-199604000-00009

OLAFSSON Y, 1995, J PEDIATR ORTHOPED, V15, P524, DOI 10.1097/01241398-199507000-00023

OLMAN S, 2005, SAUDI MED J, V26, P1429

*OXF CTR EV BAS ME, LEV EV

Padua R, 2002, ST HEAL T, V88, P404

Padua R, 2001, SPINE, V26, P1268, DOI 10.1097/00007632-200106010-00019

Parsch D, 2001, J BONE JOINT SURG BR, V83B, P1133, DOI 10.1302/0301-620X.83B8.12079

PEHRSSON K, 1992, SPINE, V17, P1091, DOI 10.1097/00007632-199209000-00014

Pehrsson K, 2001, THORAX, V56, P388, DOI 10.1136/thorax.56.5.388

PEHRSSON K, 1998, THORAX, V46, P476

Pelosi L, 2002, CLIN NEUROPHYSIOL, V113, P1082, DOI 10.1016/S1388-2457(02)00027-5

Pham V-M, 2007, Annales de Readaptation et de Medecine Physique, V50, P125, DOI 10.1016/j.annrmp.2006.11.003

POITRAS B, 1994, SPINE, V19, P1582, DOI 10.1097/00007632-199407001-00006

POLLACK A, 2005, NY TIMES 0423

PONSETI IV, 1950, J BONE JOINT SURG AM, V32-A, P381, DOI 10.2106/00004623-195032020-00017

Potter BK, 2006, SPINE, V31, P67, DOI 10.1097/01.brs.0000192721.51511.fe

Pratt RK, 2002, SPINE, V27, P1543, DOI 10.1097/00007632-200207150-00012

Price CT, 1997, J PEDIATR ORTHOPED, V17, P703, DOI 10.1097/00004694-199711000-00002

Pucher Andrzej, 2005, Ortop Traumatol Rehabil, V7, P243

Rahman T, 2005, J PEDIATR ORTHOPED, V25, P420, DOI 10.1097/01.bpo.0000161097.61586.bb

Rathjen K, 2007, SPINE, V32, P2184, DOI 10.1097/BRS.0b013e31814b88a5

Remes V, 2004, SPINE, V29, P2024, DOI 10.1097/01.brs.0000138408.64907.dc

Rigo M, 2006, SCOLIOSIS SPINAL DIS, V1, DOI 10.1186/1748-7161-1-11

Rigo M, 2003, Pediatr Rehabil, V6, P209

Rigo M, 2002, STUD HEALTH TECHNOL, V88, P241

Rinella A, 2004, SPINE, V29, P318, DOI 10.1097/01.BRS.0000111838.98892.01

Rinella A, 2004, SPINE, V29, P303, DOI 10.1097/01.BRS.0000106489.03355.C5

RISEBOROUGH EJ, 1973, J BONE JOINT SURG AM, VA 55, P974, DOI 10.2106/00004623-197355050-00006

Robin GC, 1990, ETIOLOGY IDIOPATHIC, P43

Robinson CM, 1996, J BONE JOINT SURG AM, V78A, P1140, DOI 10.2106/00004623-199608000-00003

ROGALA EJ, 1978, J BONE JOINT SURG AM, V60, P173, DOI 10.2106/00004623-197860020-00005

Roush TF, 2001, SPINE, V26, P448, DOI 10.1097/00007632-200102150-00024

Rowe DE, 1997, J BONE JOINT SURG AM, V79A, P664, DOI 10.2106/00004623-199705000-00005

Schulte TL, 2006, SPINE, V31, P315, DOI 10.1097/01.brs.0000197409.03396.24

Shapiro F, 2004, EUR SPINE J, V13, pS6, DOI 10.1007/s00586-004-0760-y

Simmons VN, 2004, ADDICT BEHAV, V29, P1129, DOI 10.1016/j.addbeh.2004.03.005

Smith PL, 2006, SPINE, V31, P2367, DOI 10.1097/01.brs.0000240204.98960.dd

SPONSELLER PD, 1987, J BONE JOINT SURG AM, V69A, P667, DOI 10.2106/00004623-198769050-00005

Stasikelis PJ, 1998, J PEDIATR ORTHOP B, V7, P111, DOI 10.1097/01202412-199804000-00004

STOKES IAF, 1994, SPINE, V19, P236, DOI 10.1097/00007632-199401001-00020

Stokes IAF, 2000, SPINE STATE ART REV, V14, P299

STONE B, 1979, PHYS THER, V59, P759, DOI 10.1093/ptj/59.6.759

Stone J, 2003, PERS SOC PSYCHOL B, V29, P846, DOI 10.1177/0146167203029007004

Suk SI, 2005, SPINE, V30, P1602, DOI 10.1097/01.brs.0000169452.50705.61

Sweet FA, 2001, SPINE, V26, P1956, DOI 10.1097/00007632-200109150-00005

Takahashi S, 2002, SPINE, V27, P1742, DOI 10.1097/00007632-200208150-00011

TAYLOR TKF, 2000, SPINE STATE ART REV, V14, P305

Trivedi JM, 2001, J PEDIATR ORTHOPED, V21, P277, DOI 10.1097/00004694-200105000-00002

UPADHYAY SS, 1995, SPINE, V20, P537, DOI 10.1097/00007632-199503010-00006

Vitale MG, 2007, SPINE J, V7, P292, DOI 10.1016/j.spinee.2006.04.004

Watanabe K, 2005, SPINE, V30, P1197, DOI 10.1097/01.brs.0000162284.38214.53

Watanabe K, 2007, SPINE, V32, P550, DOI 10.1097/01.brs.0000256474.68580.f2

Weigert KP, 2006, EUR SPINE J, V15, P1108, DOI 10.1007/s00586-005-0014-7

WEINSTEIN SL, 1983, J BONE JOINT SURG AM, V65, P447, DOI 10.2106/00004623-198365040-00004

WEINSTEIN SL, 1981, J BONE JOINT SURG AM, V63, P702, DOI 10.2106/00004623-198163050-00003

Weinstein SL, 2003, JAMA-J AM MED ASSOC, V289, P559, DOI 10.1001/jama.289.5.559

Weiss H R, 1994, Rehabilitation (Stuttg), V33, P31

Weiss H R, 1992, Ital J Orthop Traumatol, V18, P395

Weiss H R, 1997, Pediatr Rehabil, V1, P35

Weiss H.-R., 1993, EUR J PHYS MED REHAB, V3, P91

Weiss H-R, 2006, Pediatr Rehabil, V9, P190, DOI 10.1080/13638490500079583

Weiss HR, 2007, SCOLIOSIS SPINAL DIS, V2, DOI 10.1186/1748-7161-2-2

Weiss HR, 2006, SCOLIOSIS SPINAL DIS, V1, DOI 10.1186/1748-7161-1-6

Weiss HR, 2006, ST HEAL T, V123, P594

Weiss Hans-Rudolf, 2005, Pediatr Rehabil, V8, P199

Weiss Hans-Rudolf, 2003, Pediatr Rehabil, V6, P183

Weiss Hans-Rudolf, 2003, Pediatr Rehabil, V6, P111, DOI 10.1080/13638490310001593446

Weiss Hans-Rudolf, 2003, Pediatr Rehabil, V6, P23, DOI 10.1080/1363849031000095288

Weiss HR, 2002, ST HEAL T, V88, P304

Weiss HR, 2002, ST HEAL T, V88, P250

WEISS HR, 1995, Z ORTHOP GRENZGEB, V133, P114, DOI 10.1055/s-2008-1039421

WEISS HR, 1991, SPINE, V16, P88, DOI 10.1097/00007632-199101000-00016

WEISS HR, 1995, MED ORTH TECH, V5, P323

Weiss HR, 1996, ORTHOP PRAX, V32, P450

White SF, 1999, SPINE, V24, P1693, DOI 10.1097/00007632-199908150-00011

WILLERS U, 1993, SPINE, V18, P432

Wilson PL, 2002, SPINE, V27, P2036, DOI 10.1097/00007632-200209150-00013

Winter R B, 2003, J Orthop Surg (Hong Kong), V11, P202

Winter RB, 2003, SPINE, V28, P948

Wong MS, 2003, PROSTHET ORTHOT INT, V27, P121, DOI 10.1080/03093640308726668

Yrjonen T, 2007, EUR SPINE J, V16, P393, DOI 10.1007/s00586-006-0167-z

Yrjonen T, 2006, EUR SPINE J, V15, P1139, DOI 10.1007/s00586-005-0049-9

Zarzycki Daniel, 2005, Ortop Traumatol Rehabil, V7, P137

NR 216

TC 72

Z9 82

U1 0

U2 40

PU EDIZIONI MINERVA MEDICA

PI TURIN

PA CORSO BRAMANTE 83-85 INT JOURNALS DEPT., 10126 TURIN, ITALY

SN 1973-9087

EI 1973-9095

J9 EUR J PHYS REHAB MED

JI Eur. J. Phys. Rehabil. Med.

PD JUN

PY 2008

VL 44

IS 2

BP 177

EP 193

PG 17

WC Rehabilitation

WE Science Citation Index Expanded (SCI-EXPANDED)

SC Rehabilitation

GA 314EJ

UT WOS:000256791900011

PM 18418338

DA 2023-08-10

ER

PT J

AU Weinstein, SL

Dolan, LA

Cheng, JCY

Danielsson, A

Morcuende, JA

AF Weinstein, Stuart L.

Dolan, Lori A.

Cheng, Jack C. Y.

Danielsson, Aina

Morcuende, Jose A.

TI Adolescent idiopathic scoliosis

SO LANCET

LA English

DT Review

ID TERM FOLLOW-UP; CHARLESTON BENDING BRACE; POSTERIOR SPINAL-FUSION;

THORACIC PEDICLE SCREWS; NON-TREATED SCOLIOSIS; QUALITY-OF-LIFE;

PULMONARY-FUNCTION; CURVE PROGRESSION; WILMINGTON BRACE; BOSTON BRACE

AB Adolescent idiopathic scoliosis (AIS) affects 1-3% of children in the at-risk population of those aged 10-16 years. The aetiopathogensis of this disorder remains unknown, with misinformation about its natural history. Non-surgical treatments are aimed to reduce the number of operations by preventing curve progression. Although bracing and physiotherapy are common treatments in much of the world, their effectiveness has never been rigorously assessed. Technological advances have much improved the ability of surgeons to safely correct the deformity while maintaining sagittal and coronal balance. However, we do not have long-term results of these changing surgical treatments. Much has yet to be learned about the general health, quality of life, and self-image of both treated and untreated patients with AIS.

C1 [Weinstein, Stuart L.; Dolan, Lori A.; Morcuende, Jose A.] Univ Iowa, Dept Orthopaed Surg & Rehabil, Iowa City, IA USA.

[Cheng, Jack C. Y.] Chinese Univ Hong Kong, Dept Orthopaed & Traumatol, Hong Kong, china.

[Danielsson, Aina] Univ Gothenburg, Sahlgrens Univ Hosp, Dept Orthopaed, Gothenburg, Sweden.

C3 University of Iowa; Chinese University of Hong Kong; Sahlgrenska

University Hospital; University of Gothenburg

RP Weinstein, SL (通讯作者)， Univ Iowa Hosp & Clin, Dept Orthopaed Surg & Rehabil, 200 Hawkins Dr, Iowa City, IA 52242 USA.

EM stuart-weinstein@uiowa.edu

RI Cheng, Jack/J-3878-2018

OI Cheng, Jack/0000-0001-8153-364X; Dolan, Lori/0000-0003-2455-2187;

Weinstein, Stuart/0000-0002-3676-6687

CR AGADIR M, 1989, J ORTHOPAED RES, V7, P690, DOI 10.1002/jor.1100070509

Al-Sayyad MJ, 2005, CLIN ORTHOP RELAT R, P61, DOI 10.1097/01.blo.0000163244.77486.47

Allington NJ, 1996, J BONE JOINT SURG AM, V78A, P1056, DOI 10.2106/00004623-199607000-00010

ASCANI E, 1986, SPINE, V11, P784, DOI 10.1097/00007632-198610000-00007

Axenovich TI, 1999, AM J MED GENET, V86, P389

Bagnall KM, 2001, SPINE, V26, P1022, DOI 10.1097/00007632-200105010-00007

Bashiardes S, 2004, HUM GENET, V115, P81, DOI 10.1007/s00439-004-1121-y

BASSETT GS, 1987, CLIN ORTHOP RELAT R, P164

BASSETT GS, 1986, J BONE JOINT SURG AM, V68A, P602, DOI 10.2106/00004623-198668040-00019

Belmont PJ, 2001, SPINE, V26, P2340, DOI 10.1097/00007632-200111010-00010

Benli IT, 2006, SPINE, V31, P1828, DOI 10.1097/01.brs.0000227256.15525.9b

Betz RR, 2005, CLIN ORTHOP RELAT R, P55, DOI 10.1097/01.blo.0000163472.46511.a8

Betz RR, 1999, SPINE, V24, P225, DOI 10.1097/00007632-199902010-00007

Beuerlein M, 2001, SPINE, V26, P237, DOI 10.1097/00007632-200102010-00007

BJURE J, 1973, CLIN ORTHOP RELAT R, P44

BLOUNT WP, 1973, ISRAEL J MED SCI, V9, P745

BRANTHWAITE MA, 1986, BRIT J DIS CHEST, V80, P360, DOI 10.1016/0007-0971(86)90089-6

Braun JT, 2006, SPINE, V31, P1314, DOI 10.1097/01.brs.0000218662.78165.b1

Bridwell KH, 2007, J BONE JOINT SURG AM, V89A, P1654, DOI 10.2106/JBJS.G.00425

Bullmann V, 2003, SPINE, V28, P1306, DOI 10.1097/00007632-200306150-00016

BUNNELL WP, 1986, SPINE, V11, P773, DOI 10.1097/00007632-198610000-00003

Burton DC, 2005, SPINE, V30, P1979, DOI 10.1097/01.brs.0000176196.94565.d6

Burwell R G, 2003, Pediatr Rehabil, V6, P137

CARR AJ, 1992, CLIN ORTHOP RELAT R, P305

Chan V, 2002, AM J HUM GENET, V71, P401, DOI 10.1086/341607

Cheung KMC, 2003, SPINE, V28, P1941, DOI 10.1097/01.BRS.0000083140.80750.93

Chu WCW, 2006, SPINE, V31, pE19, DOI 10.1097/01.brs.0000193892.20764.51

CLAYSON D, 1987, SPINE, V12, P983, DOI 10.1097/00007632-198712000-00007

Coe JD, 2006, SPINE, V31, P345, DOI 10.1097/01.brs.0000197188.76369.13

Coillard C, 2003, EUR SPINE J, V12, P141, DOI 10.1007/s00586-002-0467-x

COLLIS DK, 1969, J BONE JOINT SURG AM, VA 51, P425, DOI 10.2106/00004623-196951030-00001

COTREL Y, 1988, CLIN ORTHOP RELAT R, P10

d'Amato CR, 2001, SPINE, V26, P2006

Danielsson AJ, 2003, SPINE, V28, pE373, DOI 10.1097/01.BRS.0000084267.41183.75

Danielsson AJ, 2001, EUR SPINE J, V10, P278, DOI 10.1007/s005860100309

Danielsson AJ, 2003, SPINE, V28, P2078

DEKONING HJ, 2007, EFFECTIVENESS BRACIN

Deyo RA, 2006, SPINE, V31, P2724, DOI 10.1097/01.brs.0000244618.06877.cd

DICKSON JH, 1995, J BONE JOINT SURG AM, V77A, P513, DOI 10.2106/00004623-199504000-00003

Dickson Robert A., 1994, P421

DWYER AF, 1974, J BONE JOINT SURG BR, VB 56, P218, DOI 10.1302/0301-620X.56B2.218

Early SD, 2002, SPINE, V27, P2368, DOI 10.1097/00007632-200211010-00011

EDGAR MA, 1988, J BONE JOINT SURG BR, V70, P712, DOI 10.1302/0301-620X.70B5.3192566

EMANS JB, 1986, SPINE, V11, P792, DOI 10.1097/00007632-198610000-00009

Emery E, 1997, Eur Spine J, V6, P158, DOI 10.1007/BF01301429

FALLSTROM K, 1986, SPINE, V11, P756

FERNANDEZFELIBERTI R, 1995, J PEDIATR ORTHOPED, V15, P176

FOWLES JV, 1978, CLIN ORTHOP RELAT R, P212

Gao XC, 2007, AM J HUM GENET, V80, P957, DOI 10.1086/513571

GAVIN TM, 2001, PEDIAT SPINE PRINCIP

Goldberg CJ, 2001, SPINE, V26, P42, DOI 10.1097/00007632-200101010-00009

Goldbloom RB, 1994, CANADIAN TASK FORCE, P346

Graham EJ, 2000, SPINE, V25, P2319, DOI 10.1097/00007632-200009150-00009

GRIMARD G, 2002, SCOL RES SOC ANN M, P112

Guo X, 2003, J BONE JOINT SURG BR, V85B, P1026, DOI 10.1302/0301-620X.85B7.14046

Hadley Miller N, 2000, Spine (Phila Pa 1976), V25, P2416

HAMMERBERG KW, 1988, ORTHOPEDICS, V11, P1365

Harrington PR, 2002, J BONE JOINT SURG AM, V84A, P316, DOI 10.2106/00004623-200202000-00020

Huynh AM, 2007, EUR SPINE J, V16, P523, DOI 10.1007/s00586-006-0235-4

Inoue M, 2005, SPINE, V30, P108, DOI 10.1097/01.brs.0000149075.96242.0e

Inoue Masatoshi, 2002, Stud Health Technol Inform, V91, P90

Justice CM, 2003, SPINE, V28, P589, DOI 10.1097/00007632-200303150-00014

KAFER ER, 1977, B EUR PHYSIOPATH RES, V13, P299

KAHANOVITZ N, 1989, SPINE, V14, P483, DOI 10.1097/00007632-198905000-00001

Kaneda K, 1996, SPINE, V21, P1250, DOI 10.1097/00007632-199605150-00021

Katz DE, 1997, SPINE, V22, P1302, DOI 10.1097/00007632-199706150-00005

KEARON C, 1993, AM REV RESPIR DIS, V148, P288, DOI 10.1164/ajrccm/148.2.288

Kesling KL, 1997, SPINE, V22, P2009, DOI 10.1097/00007632-199709010-00014

Kim YJ, 2006, SPINE, V31, P291, DOI 10.1097/01.brs.0000197865.20803.d4

Kim YJ, 2004, SPINE, V29, P333, DOI 10.1097/01.BRS.0000109983.12113.9B

KOLINDSORENSEN V, 1973, ACTA ORTHOP SCAND, V44, P98

Korovessis P, 2000, SPINE, V25, P2064, DOI 10.1097/00007632-200008150-00010

Kuklo TR, 2005, SPINE, V30, P222, DOI 10.1097/01.brs.0000150482.26918.d8

Lenke LG, 2004, SPINE, V29, P2055, DOI 10.1097/01.brs.0000138274.09504.38

Lenke LG, 2004, J PEDIATR ORTHOPED, V24, P329, DOI 10.1097/01241398-200405000-00017

Lenke LG, 2003, SPINE, V28, pS36, DOI 10.1097/00007632-200308011-00007

Lin MC, 2001, ARCH PHYS MED REHAB, V82, P335, DOI 10.1053/apmr.2001.21528

Little DG, 2000, J BONE JOINT SURG AM, V82A, P685, DOI 10.2106/00004623-200005000-00009

Lonner BS, 2006, J BONE JOINT SURG AM, V88A, P1022, DOI 10.2106/JBJS.E.00001

LONSTEIN JE, 1984, J BONE JOINT SURG AM, V66A, P1061, DOI 10.2106/00004623-198466070-00013

Lowe T, 2002, SPINE, V27, P768, DOI 10.1097/00007632-200204010-00016

Lowe TG, 2000, J BONE JOINT SURG AM, V82A, P1157, DOI 10.2106/00004623-200008000-00014

Lowe TG, 2003, SPINE, V28, pS208, DOI 10.1097/01.BRS.0000092483.10776.2A

Maiocco B, 1997, SPINE, V22, P2537, DOI 10.1097/00007632-199711010-00014

MAYO NE, 1994, SPINE, V19, P1573, DOI 10.1097/00007632-199407001-00005

MEROLA AA, 2003, SCOL RES SOC M 2003

MILLER JAA, 1984, SPINE, V9, P632, DOI 10.1097/00007632-198409000-00015

Miller NH, 1996, J ORTHOP RES, V14, P994, DOI 10.1002/jor.1100140621

MILLER NH, 2003, 38 ANN M SCOL RES SO

MOE JH, 1983, CLIN ORTHOP RELAT R, P133

Morcuende JA, 2003, SPINE, V28, P2025, DOI 10.1097/01.BRS.0000083235.74593.49

Muschik MT, 2006, EUR SPINE J, V15, P1128, DOI 10.1007/s00586-005-0034-3

NACHEMSON A, 1968, ACTA ORTHOP SCAND, V39, P466, DOI 10.3109/17453676808989664

NACHEMSON AL, 1995, J BONE JOINT SURG AM, V77A, P815, DOI 10.2106/00004623-199506000-00001

NACHEMSON AL, 1982, REPORT PREVALENCE NA

Negrini A, 2001, EUR MED PHYS, V37, P181

Negrini S, 2005, Eura Medicophys, V41, P183

NILSONNE U, 1968, ACTA ORTHOP SCAND, V39, P456, DOI 10.3109/17453676808989663

O'Kelly C, 1999, SPINE, V24, P35, DOI 10.1097/00007632-199901010-00009

O'Neill PJ, 2005, J BONE JOINT SURG AM, V87A, P1069, DOI 10.2106/JBJS.C.01707

OLAFSSON Y, 1995, J PEDIATR ORTHOPED, V15, P524, DOI 10.1097/01241398-199507000-00023

Olafsson Y, 1999, EUR SPINE J, V8, P402, DOI 10.1007/s005860050194

Parent S, 2004, SPINE, V29, P239, DOI 10.1097/01.BRS.0000109995.64028.FE

Parent Stefan, 2005, Instr Course Lect, V54, P529

PEHRSSON K, 1991, THORAX, V46, P474, DOI 10.1136/thx.46.7.474

Pehrsson K, 2001, THORAX, V56, P388, DOI 10.1136/thorax.56.5.388

PETERSON LE, 1995, J BONE JOINT SURG AM, V77A, P823, DOI 10.2106/00004623-199506000-00002

PIAZZA MR, 1990, J PEDIATR ORTHOPED, V10, P39

PICAULT C, 1981, SPINE, V11, P77

PONSETI IV, 1950, J BONE JOINT SURG AM, V52, P131

Poon AMS, 2006, SPINE, V31, P2043, DOI 10.1097/01.brs.0000231796.49827.39

PRICE CT, 1990, SPINE, V15, P1294, DOI 10.1097/00007632-199012000-00011

Price CT, 1997, J PEDIATR ORTHOPED, V17, P703, DOI 10.1097/00004694-199711000-00002

Rahman T, 2005, J PEDIATR ORTHOPED, V25, P420, DOI 10.1097/01.bpo.0000161097.61586.bb

Rajwani T, 2004, SPINE, V29, pE145, DOI 10.1097/01.BRS.0000120507.36611.8D

RICHARDS BS, 1994, SPINE, V19, P1598, DOI 10.1097/00007632-199407001-00008

Rigo M, 2006, SCOLIOSIS SPINAL DIS, V1, DOI 10.1186/1748-7161-1-11

Rowe DE, 1997, J BONE JOINT SURG AM, V79A, P664, DOI 10.2106/00004623-199705000-00005

Salehi LB, 2002, HUM GENET, V111, P401, DOI 10.1007/s00439-002-0785-4

SCHATZINGER LH, 1979, AM J NURS, V79, P1608, DOI 10.2307/3424697

Sevastik JA, 2006, STUD HEALTH TECHNOL, V123, P552

Spoonamore MJ, 2004, SPINE, V29, P1458, DOI 10.1097/01.BRS.0000128756.89367.9E

Sucato DJ, 2004, SPINE, V29, P554, DOI 10.1097/01.BRS.0000106495.91477.92

Suk SI, 2005, SPINE, V30, P1602, DOI 10.1097/01.brs.0000169452.50705.61

Suk SI, 2001, SPINE, V26, P2049, DOI 10.1097/00007632-200109150-00022

Szappanos L, 1997, Acta Chir Hung, V36, P343

Tang NLS, 2006, SPINE, V31, P2463, DOI 10.1097/01.brs.0000239179.81596.2b

Tones M, 2006, SPINE, V31, P3027, DOI 10.1097/01.brs.0000249555.87601.fc

Trivedi JM, 2001, J PEDIATR ORTHOPED, V21, P277, DOI 10.1097/00004694-200105000-00002

TURI M, 1993, SPINE, V18, P417

*US PREV SERV TASK, 2004, SCREEN ID SCOL AD RE

Veldhuizen AG, 2002, MED ENG PHYS, V24, P209, DOI 10.1016/S1350-4533(02)00008-5

Vijvermans V, 2004, J PEDIATR ORTHOP B, V13, P143, DOI 10.1097/00009957-200405000-00001

Villemure I, 2004, EUR SPINE J, V13, P83, DOI 10.1007/s00586-003-0565-4

WEINSTEIN SL, 1983, J BONE JOINT SURG AM, V65, P447, DOI 10.2106/00004623-198365040-00004

WEINSTEIN SL, 1981, J BONE JOINT SURG AM, V63, P702, DOI 10.2106/00004623-198163050-00003

Weinstein SL, 2003, JAMA-J AM MED ASSOC, V289, P559, DOI 10.1001/jama.289.5.559

WEINSTEIN SL, 2007, BRAIST BRACING ADOLE

Weiss HR, 2006, SCOLIOSIS SPINAL DIS, V1, DOI [10.1186/1748-7161-1-5, 10.1186/1748-7161-1-1]

Weiss Hans-Rudolf, 2003, Pediatr Rehabil, V6, P183

Weiss Hans-Rudolf, 2002, Stud Health Technol Inform, V91, P342

Wiley JW, 2000, SPINE, V25, P2326, DOI 10.1097/00007632-200009150-00010

Wise CA, 2000, SPINE, V25, P2372, DOI 10.1097/00007632-200009150-00017

Wu J, 2006, SPINE, V31, P1131, DOI 10.1097/01.brs.0000216603.91330.6f

NR 144

TC 759

Z9 844

U1 11

U2 194

PU ELSEVIER SCIENCE INC

PI NEW YORK

PA 360 PARK AVE SOUTH, NEW YORK, NY 10010-1710 USA

SN 0140-6736

EI 1474-547X

J9 LANCET

JI Lancet

PD MAY 3

PY 2008

VL 371

IS 9623

BP 1527

EP 1537

DI 10.1016/S0140-6736(08)60658-3

PG 11

WC Medicine, General & Internal

WE Science Citation Index Expanded (SCI-EXPANDED)

SC General & Internal Medicine

GA 298ED

UT WOS:000255668300030

PM 18456103

DA 2023-08-10

ER

PT J

AU Negrini, S

Fusco, C

Minozzi, S

Atanasio, S

Zaina, F

Romano, M

AF Negrini, S.

Fusco, C.

Minozzi, S.

Atanasio, S.

Zaina, F.

Romano, M.

TI Exercises reduce the progression rate of adolescent idiopathic

scoliosis: Results of a comprehensive systematic review of the

literature

SO DISABILITY AND REHABILITATION

LA English

DT Article

DE physical exercises; adolescent idiopathic scoliosis; conservative

treatment; physiotherapy; rehabilitation

ID SOSORT CONSENSUS PAPER; SEAS.02 EXERCISES; BRACE TREATMENT; EFFICACY;

THERAPY; PROGRAM

AB Background. A previously published systematic review (Ped. Rehab. 2003 - DARE 2004) documented the existence of the evidence of level 2a (Oxford EBM Centre) on the efficacy of specific exercises to reduce the progression of AIS (Adolescent Idiopathic Scoliosis).

Aim. To confirm whether the indication for treatment with specific exercises for AIS has changed in recent years.

Study design. Systematic review.

Methods. A bibliographic search with strict inclusion criteria (patients treated exclusively with exercises, outcome Cobb degrees, all study designs) was performed on the main electronic databases and through extensive manual searching. We retrieved 19 studies, including one RCT and eight controlled studies; 12 studies were prospective. A methodological and clinical evaluation was performed.

Results. The 19 papers considered included 1654 treated patients and 688 controls. The highest-quality study (RCT) compared two groups of 40 patients, showing an improvement of curvature in all treated patients after six months. We found three papers on Scoliosis Intensive Rehabilitation (Schroth), five on extrinsic autocorrection-based methods (Schroth, side-shift), four on intrinsic autocorrection-based approaches (Lyon and SEAS) and five with no autocorrection (three asymmetric, two symmetric exercises). Apart from one (no autocorrection, symmetric exercises, very low methodological quality), all studies confirmed the efficacy of exercises in reducing the progression rate (mainly in early puberty) and/or improving the Cobb angles (around the end of growth). Exercises were also shown to be effective in reducing brace prescription.

Conclusion. In five years, eight more papers have been published to the indexed literature coming from throughout the world (Asia, the US, Eastern Europe) and proving that interest in exercises is not

exclusive to Western Europe. This systematic review confirms and strengthens the previous ones. The actual evidence on exercises for AIS is of level 1b.

C1 [Negrini, S.; Fusco, C.; Minozzi, S.; Atanasio, S.; Zaina, F.; Romano, M.] ISICO Italian Sci Spine Inst, Milan, Italy.

RP Negrini, S (通讯作者)， Sci ISICO Ist Sci Italiano Colonna Vertebrale, Milan, Italy.

EM stefano.negrini@isico.it

RI Minozzi, Silvia/ABG-1115-2020; Negrini, Stefano/B-6667-2013; Zaina,

Fabio/H-3261-2013

OI Minozzi, Silvia/0000-0003-0471-8581; Negrini,

Stefano/0000-0002-1878-2747; Zaina, Fabio/0000-0002-1256-5362

CR [Anonymous], P 11 INT C WORLD CON

[Anonymous], 2005, ZHONGGUO LINCHUANG K

[Anonymous], 1939, GENUINE WORKS HIPPOC

[Anonymous], P INT C PREV SCOL SC

[Anonymous], 2001, OXFORD CTR EVIDENCE

[Anonymous], KINESITHERAPIE SCI

Athanasopoulos S, 1999, SCAND J MED SCI SPOR, V9, P36

den Boer WA, 1999, EUR SPINE J, V8, P406, DOI 10.1007/s005860050195

Dobosiewicz Krystyna, 2002, Stud Health Technol Inform, V91, P336

Ducong P, 2002, RESONANCE EUROPEENNE, V10, P1229

Durmala Jacek, 2003, Ortop Traumatol Rehabil, V5, P80

FERRARO C, 1998, EUROPA MEDICOPHYSICA, P25

Lehnert-Schroth C., 2007, 3 DIMENSIONAL TREATM, V7th ed.

Lenssinck MLB, 2005, PHYS THER, V85, P1329, DOI 10.1093/ptj/85.12.1329

Mamyama Toni, 2002, Stud Health Technol Inform, V91, P361

MARUYAMA T, 2003, J BONE JOINT SURG B, V85

Mcintire K, 2006, ST HEAL T, V123, P273

Moen KY, 1999, SPINE, V24, P2570, DOI 10.1097/00007632-199912150-00003

MOLLON G, ENCY MED CHIR

Mooney V, 2000, J SPINAL DISORD, V13, P102, DOI 10.1097/00002517-200004000-00002

Negrini A, 2001, EURA MEDICOPHYS, V37, P181

Negrini S, 2006, Eura Medicophys, V42, P173

Negrini S, 2007, Eura Medicophys, V43, P381

Negrini S, 2007, Eura Medicophys, V43, P171

Negrini S, 2005, Eura Medicophys, V41, P183

NEGRINI S, 2007, EURA MEDICOPHYS, V43, P183

Negrini S., 2007, EVIDENCE BASED ISICO

Negrini S, 2006, ST HEAL T, V123, P523

Negrini S, 2006, ST HEAL T, V123, P519

Negrini S, 2007, PHYS THER, V87, P112, DOI 10.2522/ptj.2007.87.1.112

Negrini S, 2006, SCOLIOSIS SPINAL DIS, V1, DOI 10.1186/1748-7161-1-4

Negrini Stefano, 2003, Pediatr Rehabil, V6, P227, DOI: 10.1080/13638490310001636781

Otman S, 2005, SAUDI MED J, V26, P1429

PIROLA V, 1999, CHINESITERAPIA NELLA

REILLY RP, 1989, AM J PHYS MED REHAB, V68, P196, DOI 10.1097/00002060-198908000-00009

Rigo M, 2006, SCOLIOSIS SPINAL DIS, V1, DOI 10.1186/1748-7161-1-11

Roach JW, 1997, PEDIAT SPINE PRINCIP, P497

Rowe DE, 1997, J BONE JOINT SURG AM, V79A, P664, DOI 10.2106/00004623-199705000-00005

Section of Physical and Rehabilitation Medicine Union Europeenne des Medecins Specialistes (UEMS), 2006, Eura Medicophys, V42, P292

SOUCHARD PE, 2002, SCOLIOSI TRATTAMENTO

SOUCHARD PE, 1982, POSTURE MEZIERESED

STAGNARA P, 1990, REEDUCATION SCOLIOSE

STONE B, 1979, PHYS THER, V59, P759, DOI 10.1093/ptj/59.6.759

WEIGL M, 2007, EUR MEDICOPHYS 1109

Weiss H R, 1992, Ital J Orthop Traumatol, V18, P395

Weiss H R, 1997, Pediatr Rehabil, V1, P35

Weiss HR, 2006, SCOLIOSIS SPINAL DIS, V1, DOI 10.1186/1748-7161-1-6

Weiss Hans-Rudolf, 2003, Pediatr Rehabil, V6, P23, DOI 10.1080/1363849031000095288

WEISS HR, 1991, SPINE, V16, P88, DOI 10.1097/00007632-199101000-00016

Wong MS, 2001, PROSTHET ORTHOT INT, V25, P60, DOI 10.1080/03093640108726570

NR 50

TC 86

Z9 93

U1 0

U2 63

PU TAYLOR & FRANCIS LTD

PI ABINGDON

PA 2-4 PARK SQUARE, MILTON PARK, ABINGDON OR14 4RN, OXON, united kingdom

SN 0963-8288

EI 1464-5165

J9 DISABIL REHABIL

JI Disabil. Rehabil.

PY 2008

VL 30

IS 10

BP 772

EP 785

DI 10.1080/09638280801889568

PG 14

WC Rehabilitation

WE Science Citation Index Expanded (SCI-EXPANDED); Social Science Citation Index (SSCI)

SC Rehabilitation

GA 308JS

UT WOS:000256386300005

PM 18432435

DA 2023-08-10

ER

PT J

AU Negrini, S

AF Negrini, Stefano

TI Approach to scoliosis changed due to causes other than evidence:
Patients call for conservative (rehabilitation) experts to join in team
orthopedic surgeons

SO DISABILITY AND REHABILITATION

LA English

DT Article

DE adolescent idiopathic scoliosis; rehabilitation; bracing; surgery;
surgery; exercises

ID ADOLESCENT IDIOPATHIC SCOLIOSIS; TERM FOLLOW-UP; SURGICAL-TREATMENT;
MILWAUKEE-BRACE; QUESTIONNAIRE; VALIDITY; MEDICINE

AB Purpose. To look critically at the present reality of AIS (Adolescent Idiopathic Scoliosis) treatment and verify the hypothesis that the current prevalence of a single medical specialty could be creating distortions in patient care and/or cure.

Method. This is a multifaceted study comprising a review of the evidence on AIS, a bibliometric study of the general and orthopedic literature since Medline start, and two case reports.

Results. Evidence exists to support the efficacy of exercises, bracing and fusion (grade B, B and C recommendations, respectively), but in clinics exercises are generally ignored; braces are used with some criticism, while fusion is generally considered the only reliable treatment. The literature on AIS treatment prevails in journals of orthopedic surgery, and therapy papers focused on surgery have increased from 34 to 55% over the past two decades. The two clinical cases show how an incorrect psychological approach to the patient and family, as well as inappropriate conservative treatments can have disastrous consequences for patients.

Conclusions. Our results seem to confirm the initial hypothesis: The interest of the AIS treatment community (composed almost exclusively by orthopedic surgeons) has shifted toward fusion whereas research has increased, while conservative treatment is suffering a decrease in professional interest (and diminished research). AIS requires expert, committed evidence-based care, but other specialists totally devoted to conservative treatment, particularly (but not exclusively) Physical and Rehabilitation Medicine specialists, should enter the field to create better treating teams.

C1 Sci ISICO Italian Sci Spine Inst, Milan, Italy.

RP Negrini, S (通讯作者)，Sci ISICO Italian Sci Spine Inst, Milan, Italy.

EM stefano.negrini@isico.it

RI Negrini, Stefano/B-6667-2013

OI Negrini, Stefano/0000-0002-1878-2747

CR [Anonymous], 2001, OXFORD CTR EVIDENCE

[Anonymous], 2006, EURA MEDICOPHYS, V42, P292

Asher M, 2003, SPINE, V28, P74, DOI 10.1097/00007632-200301010-00017

Asher M, 2003, SPINE, V28, P63, DOI 10.1097/00007632-200301010-00015

Asher M, 2003, SPINE, V28, P70, DOI 10.1097/00007632-200301010-00016

BLOUNT WP, 1957, J BONE JOINT SURG AM, V39, P693

Botens-Helmus C, 2006, SCOLIOSIS SPINAL DIS, V1, DOI 10.1186/1748-7161-1-22

COBB J, 1948, INSTRUCTIONAL COURSE, V5, P241

Dickson RA, 1999, J BONE JOINT SURG BR, V81B, P193, DOI 10.1302/0301-620X.81B2.9630

Dolan LA, 2007, J PEDIATR ORTHOPED, V27, P270, DOI 10.1097/01.bpb.0000248579.11864.47

Dolan LA, 2007, SPINE, V32, pS91, DOI 10.1097/BRS.0b013e318134ead9

Ford S, 2003, SOC SCI MED, V56, P589, DOI 10.1016/S0277-9536(02)00056-4

Giovannoni S, 2006, Eura Medicophys, V42, P177

Goldberg CJ, 2001, SPINE, V26, P42, DOI 10.1097/00007632-200101010-00009

Grivas TB, 2007, SCOLIOSIS SPINAL DIS, V2, DOI 10.1186/1748-7161-2-17

Grivas TB, 2008, DISABIL REHABIL, V30, P752, DOI 10.1080/09638280802041086

Hawes Martha, 2006, Pediatr Rehabil, V9, P318, DOI 10.1080/13638490500402264

Hawes MC, 2006, SCOLIOSIS SPINAL DIS, V1, DOI 10.1186/1748-7161-1-3

Hawes MC, 2008, DISABIL REHABIL, V30, P808, DOI 10.1080/09638280801889972

Hawes MC, 2003, SCOLIOSIS HUMAN SPIN

*ICF, 2001, CLASS INT FUNZ DIS S

Kotwicki T, 2008, DISABIL REHABIL, V30, P792, DOI 10.1080/09638280801889584

Kotwicki T, 2008, DISABIL REHABIL, V30, P742, DOI 10.1080/09638280801889519

Lenssinck MLB, 2005, PHYS THER, V85, P1329, DOI 10.1093/ptj/85.12.1329

Maruyama T, 2008, DISABIL REHABIL, V30, P786, DOI 10.1080/09638280801889782

Maruyama Toru, 2003, Pediatr Rehabil, V6, P215

MOE JH, 1970, J BONE JOINT SURG AM, VA 52, P1509, DOI 10.2106/00004623-197052080-00001

Monticone M, 2004, Eura Medicophys, V40, P191

NACHEMSON A, 1968, ACTA ORTHOP SCAND, V39, P466, DOI 10.3109/17453676808989664

NACHEMSON AL, 1995, J BONE JOINT SURG AM, V77A, P815, DOI 10.2106/00004623-199506000-00001

Negrini S, 2007, Eura Medicophys, V43, P75

Negrini S, 2004, Eura Medicophys, V40, P1

Negrini S, 2006, Eura Medicophys, V42, P151

Negrini S, 2006, Eura Medicophys, V42, P173

Negrini S, 2007, Eura Medicophys, V43, P381

Negrini S, 2007, Eura Medicophys, V43, P171

Negrini S, 2008, DISABIL REHABIL, V30, P772, DOI 10.1080/09638280801889568

Negrini S, 2005, Eura Medicophys, V41, P183

NEGRINI S, 2007, 4 INT C CONS MAN SPI

NEGRINI S, 2007, J SURG ORTHOP ADV, V16, P99

Negrini S, 2004, PEDIAT REHABIL, V7, P52

Negrini Stefano, 2007, J Surg Orthop Adv, V16, P98

Negrini S, 2006, SCOLIOSIS SPINAL DIS, V1, DOI 10.1186/1748-7161-1-14

Negrini S, 2006, SCOLIOSIS SPINAL DIS, V1, DOI 10.1186/1748-7161-1-4

Negrini Stefano, 2003, Pediatr Rehabil, V6, P227, DOI: 10.1080/13638490310001636781

Pineda S, 2006, SCOLIOSIS SPINAL DIS, V1, DOI 10.1186/1748-7161-1-18

PONSETI IV, 1950, J BONE JOINT SURG AM, V32-A, P381, DOI 10.2106/00004623-195032020-00017

Rigo M, 2006, SCOLIOSIS SPINAL DIS, V1, DOI 10.1186/1748-7161-1-11

Rigo M, 2003, Pediatr Rehabil, V6, P209

RISSER JC, 1976, CLIN ORTHOP RELAT R, P86

Rowe DE, 1997, J BONE JOINT SURG AM, V79A, P664, DOI 10.2106/00004623-199705000-00005

Sackett DL, 1995, J ROY SOC MED, V88, P620

Shindle Michael K, 2006, J Surg Orthop Adv, V15, P43

SIBILLA P, 2001, DEFORMITA VERTEBRALI, P20

SIBILLA P, 2002, RACHIDE RIABILITAZIO, P73

Smania N, 2008, DISABIL REHABIL, V30, P763, DOI 10.1080/17483100801921311

Sponseller PD, 2003, JAMA-J AM MED ASSOC, V289, P608, DOI 10.1001/jama.289.5.608

SPONSELLER PD, 1987, J BONE JOINT SURG AM, V69A, P667, DOI 10.2106/00004623-198769050-00005

STAGNARA P, 1976, DEFORMATIONS RACHISE

Stucki G, 2003, DISABIL REHABIL, V25, P628, DOI 10.1080/09638280110070221

Vasiliadis E, 2006, SCOLIOSIS SPINAL DIS, V1, DOI 10.1186/1748-7161-1-7

Waljee JF, 2007, J CLIN ONCOL, V25, P3694, DOI 10.1200/JCO.2007.10.9272

WEIGL M, 2007, EURA MEDICOPHYS 1109

Weiss HR, 2008, DISABIL REHABIL, V30, P799, DOI 10.1080/09638280801889717

Weiss HR, 2006, SCOLIOSIS SPINAL DIS, V1, DOI [10.1186/1748-7161-1-5, 10.1186/1748-7161-1-1]

Weiss HR, 2006, SCOLIOSIS SPINAL DIS, V1, DOI 10.1186/1748-7161-1-6

Weiss Hans-Rudolf, 2003, Pediatr Rehabil, V6, P111, DOI 10.1080/13638490310001593446

WEISS HR, 2004, METAANALYSIS PREVALE, P416

Winter R B, 1983, Instr Course Lect, V32, P170

NR 69

TC 34

Z9 38

U1 0

U2 12

PU TAYLOR & FRANCIS LTD

PI ABINGDON

PA 4 PARK SQUARE, MILTON PARK, ABINGDON OX14 4RN, OXON, united kingdom

SN 0963-8288

J9 DISABIL REHABIL

JI Disabil. Rehabil.

PY 2008

VL 30

IS 10

BP 731

EP 741

DI 10.1080/09638280801889485

PG 11

WC Rehabilitation

WE Science Citation Index Expanded (SCI-EXPANDED); Social Science Citation Index (SSCI)

SC Rehabilitation

GA 308JS

UT WOS:000256386300001

PM 18432431

DA 2023-08-10

ER

PT J

AU Schiller, JR

Eberson, CP

AF Schiller, Jonathan R.

Eberson, Craig P.

TI Spinal deformity and athletics

SO SPORTS MEDICINE AND ARTHROSCOPY REVIEW

LA English

DT Review

DE scoliosis; kyphosis; deformity; bracing; fusion; athletics

ID ADOLESCENT IDIOPATHIC SCOLIOSIS; CHARLESTON BENDING BRACE; TERM

FOLLOW-UP; BACK-PAIN; MECHANICAL MODULATION; SCHEUERMANN KYPHOSIS;

FUSIONLESS TREATMENT; THORACOLUMBAR SPINE; BOSTON BRACE; GROWTH

AB Exercise and athletic competition for the young individual has become increasingly more important in society. Scoliosis and Scheurmann kyphosis are spinal deformities prevalent in up to 2% to 3% and 7% of the population respectively, requiring nonoperative and occasionally operative treatment. Curve progression and patient physiologic age dictate treatment regimens. Bracing and physical therapy is the mainstay for nonoperative treatment, whereas soft tissue releases and fusion with instrumentation are used for operative correction. Athletic activity and sports participation is usually allowed for patients undergoing nonoperative treatment. Return to sport after surgical correction is variable, often decided by the treating surgeon, and based on the level of fusion and sporting activity. Although most treating surgeons promote some form of activity regardless of treatment modality chosen, caution should be taken when deciding on participation in collision activities such as football and wrestling.

C1 [Schiller, Jonathan R.; Eberson, Craig P.] Brown Univ, Warren Alpert Sch Med, Dept Orthopaed, Providence, RI 02905 USA.

C3 Brown University

RP Eberson, CP (通讯作者)， Brown Univ, Warren Alpert Sch Med, Dept Orthopaed, 2 Dudley St,Suite 200, Providence, RI 02905 USA.

EM Ceberson@lifespan.org

CR Andersen MO, 2006, SPINE, V31, P350, DOI 10.1097/01.brs.0000197649.29712.de

Betz RR, 2005, CLIN ORTHOP RELAT R, P55, DOI 10.1097/01.blo.0000163472.46511.a8

BRADFORD DS, 1975, J BONE JOINT SURG AM, VA 57, P439, DOI 10.2106/00004623-197557040-00001

Braun JT, 2006, SPINE, V31, P1776, DOI 10.1097/01.brs.0000227263.43060.50

Braun JT, 2006, SPINE, V31, P1314, DOI 10.1097/01.brs.0000218662.78165.b1

Braun JT, 2005, SPINE, V30, pS35, DOI 10.1097/01.brs.0000175187.61474.9a

CRAWFORD A, 2007, AAOS ANN M INSTR COU

d'Amato CR, 2001, SPINE, V26, P2006

Danielsson AJ, 2006, SPINE, V31, P275, DOI 10.1097/01.brs.0000197652.52890.71

DIMEGLIO A, 2007, PEDIAT ORTHOPAEDIC S

Engsberg JB, 2002, SPINE, V27, P1346, DOI 10.1097/00007632-200206150-00018

Facanha FAM, 2001, J BONE JOINT SURG AM, V83A, P42, DOI 10.2106/00004623-200101000-00006

Haefeli M, 2006, SPINE, V31, P355, DOI 10.1097/01.brs.0000197664.02098.09

HALAL F, 1978, AM J DIS CHILD, V132, P1105, DOI 10.1001/archpedi.1978.02120360061011

HELLSTROM M, 1990, ACTA RADIOL, V31, P127

Katz DE, 2001, SPINE, V26, P2354, DOI 10.1097/00007632-200111010-00012

Katz DE, 1997, SPINE, V22, P1302, DOI 10.1097/00007632-199706150-00005

LEE MC, 2006, P PED ORTH SOC N AM

Lee SS, 2006, SPINE, V31, P2316, DOI 10.1097/01.brs.0000238977.36165.b8

Lenke Lawrence G, 2005, Instr Course Lect, V54, P537

LONSTEIN JE, 1984, J BONE JOINT SURG AM, V66A, P1061, DOI 10.2106/00004623-198466070-00013

Lowe TG, 2005, SPINE, V30, pS69, DOI 10.1097/01.brs.0000175175.41471.d4

LOWE TG, 1990, J BONE JOINT SURG AM, V72A, P940, DOI 10.2106/00004623-199072060-00026

Masso PD, 2002, J PEDIATR ORTHOPED, V22, P279, DOI 10.1097/00004694-200205000-00002

OHLEN G, 1989, SPINE, V14, P847

Omey ML, 2000, CLIN ORTHOP RELAT R, P74

Parsch D, 2002, CLIN J SPORT MED, V12, P95, DOI 10.1097/00042752-200203000-00005

Pizzutillo P D, 1993, Instr Course Lect, V42, P463

Price CT, 1997, J PEDIATR ORTHOPED, V17, P703, DOI 10.1097/00004694-199711000-00002

Ramirez N, 1997, J BONE JOINT SURG AM, V79A, P364, DOI 10.2106/00004623-199703000-00007

Rubery PT, 2002, SPINE, V27, P423, DOI 10.1097/00007632-200202150-00019

SACHS B, 1987, J BONE JOINT SURG AM, V69A, P50, DOI 10.2106/00004623-198769010-00009

Stokes IAF, 1996, SPINE, V21, P1162, DOI 10.1097/00007632-199605150-00007

SWARD L, 1990, SPINE, V15, P124

Tosi LL, 2000, CLIN ORTHOP RELAT R, P17

Tribus C B, 1998, J Am Acad Orthop Surg, V6, P36

Vijvermans V, 2004, J PEDIATR ORTHOP B, V13, P143, DOI 10.1097/00009957-200405000-00001

Wall EJ, 2005, SPINE, V30, P1148, DOI 10.1097/01.brs.0000162278.68000.91

Weinstein S L, 1989, Instr Course Lect, V38, P115

Wojtys EM, 2000, AM J SPORT MED, V28, P490, DOI 10.1177/03635465000280040801

Wood KB, 2002, CLIN SPORT MED, V21, P77, DOI 10.1016/S0278-5919(03)00058-9

NR 41

TC 15

Z9 17

U1 0

U2 8

PU LIPPINCOTT WILLIAMS & WILKINS

PI PHILADELPHIA

PA TWO COMMERCE SQ, 2001 MARKET ST, PHILADELPHIA, PA 19103 USA

SN 1062-8592

EI 1538-1951

J9 SPORTS MED ARTHROSC

JI Sports Med. Arthrosc. Rev.

PY 2008

VL 16

IS 1

BP 26

EP 31

DI 10.1097/JSA.0b013e3181629aa8

PG 6

WC Sport Sciences

WE Science Citation Index Expanded (SCI-EXPANDED)

SC Sport Sciences

GA 264HJ

UT WOS:000253278100005

PM 18277259

DA 2023-08-10

ER

PT J

AU Smania, N

Picelli, A

Romano, M

Negrini, S

AF Smania, Nicola

Picelli, Alessandro

Romano, Michele

Negrini, Stefano

TI Neurophysiological basis of rehabilitation of adolescent idiopathic scoliosis

SO DISABILITY AND REHABILITATION

LA English

DT Article

DE body schema; bracing; neurophysiology; rehabilitation; scoliosis

ID LOW-BACK-PAIN; UPPER-LIMB; POSTURAL ADJUSTMENTS; TRANSVERSUS ABDOMINIS;

TRUNK; RESPONSES; MOVEMENTS; BODY; MUSCLES; SPACE

AB Background. Knowledge on mechanisms of neurophysiological control of trunk movement and posture could help in the development of rehabilitation programs and brace treatment in adolescent idiopathic scoliosis (AIS).

Aims. Reviewing up-to-date research on neurophysiology of movement and posture control with the aim of providing basis for new researches in the field of AIS rehabilitation and background understanding for clinicians engaged in management of AIS.

Methods. Review of literature.

Results. We considered several neurophysiological issues relevant for AIS rehabilitation, namely, the peculiar organization of patterns of trunk muscle recruitment, the structure of the neural hardware subserving axial and arm muscle control, and the relevance of cognitive systems allowing mapping of spatial coordinates and building of body schema.

Discussion and conclusion. We made clear the reason why trunk control is generally carried out by means of very fast, feedforward or feedback driven patterns of muscle activation which are deeply rooted in our neural control system and very difficult to modify by training. We hypothesized that augmented sensory feedback and strength exercises could be an important stage in a rehabilitation program aimed at hindering, or possibly reversing, scoliosis progression. In this context we considered bracing not only as a corrective biomechanical device but also as a tool for continuous sensory stimulation that could help awareness of body misalignment. Future research aimed at developing strategies of trunk postural control learning is essential in the rehabilitation of adolescent idiopathic scoliosis.

C1 [Smania, Nicola] GB Rossi Univ Hosp, Rehabil Unit, I-37134 Verona, Italy.

[Smania, Nicola; Picelli, Alessandro] Univ Verona, Dept Neurol & Vis Sci, Neurorehabil Sect, I-37100 Verona, Italy.

[Romano, Michele; Negrini, Stefano] ISICO Italian Sci Spine Inst, Milan, Italy.

C3 University of Verona; Azienda Ospedaliera Universitaria Integrata

Verona; University of Verona

RP Smania, N (通讯作者)，GB Rossi Univ Hosp, Rehabil Unit, Via LA Scuro 10, I-37134 Verona, Italy.

EM nicola.smania@univr.it

RI Picelli, Alessandro/K-5610-2016; Negrini, Stefano/B-6667-2013

OI Picelli, Alessandro/0000-0002-3558-8276; Negrini,

Stefano/0000-0002-1878-2747; smania, nicola/0000-0001-7630-1887

CR Aglioti S, 1997, BEHAV NEUROSCI, V111, P867, DOI 10.1037/0735-7044.111.5.867

Aglioti S, 1996, NEUROREPORT, V8, P293, DOI 10.1097/00001756-199612200-00058

Alexandrov A, 1998, EXP BRAIN RES, V118, P210, DOI 10.1007/s002210050274

Allum JHJ, 1998, EXP BRAIN RES, V121, P478, DOI 10.1007/s002210050484

[Anonymous], KINESITHERAPIE SCI

[Anonymous], PRINCIPLES NEURAL SC

ARUIN AS, 1995, EXP BRAIN RES, V103, P323

Asher MA, 2006, SCOLIOSIS SPINAL DIS, V1, DOI 10.1186/1748-7161-1-2

Avikainen VJ, 1999, J SPINAL DISORD, V12, P61

Bagesteiro LB, 2006, EXP BRAIN RES, V171, P358, DOI 10.1007/s00221-005-0272-y

Bagnall KM, 1996, SPINE, V21, P1974, DOI 10.1097/00007632-199609010-00006

Bazzarelli Michael, 2002, Stud Health Technol Inform, V91, P383

BELEN'KII V. E., 1967, BIOFIZIKA, V12, P135

Berlucchi G, 1997, TRENDS NEUROSCI, V20, P560, DOI 10.1016/S0166-2236(97)01136-3

Blagoveshchenskii E D, 2005, Neurosci Behav Physiol, V35, P299, DOI 10.1007/s11055-005-0008-2

Bogduk N, 1997, CLIN ANA TOMY LUMBAR, V3rd, P177

Bradl I, 2005, Pathophysiology, V12, P275, DOI 10.1016/j.pathophys.2005.09.002

BURKE D, 1992, J PHYSIOL-LONDON, V449, P655, DOI 10.1113/jphysiol.1992.sp019107

Burwell RG, 2006, STUD HEALTH TECHNOL, V123, P72

Cheung J, 2005, EUR SPINE J, V14, P130, DOI 10.1007/s00586-004-0780-7

Critchley M., 1979, DIVINE BANQUET BRAIN

Danielsson AJ, 2001, SPINE, V26, P1449, DOI 10.1097/00007632-200107010-00015

DELWAIDE PJ, 1977, J NEUROL NEUROSUR PS, V40, P616, DOI 10.1136/jnnp.40.6.616

Fogassi L, 1996, J NEUROPHYSIOL, V76, P141, DOI 10.1152/jn.1996.76.1.141

FRIEDLI WG, 1988, J NEUROL NEUROSUR PS, V51, P232, DOI 10.1136/jnnp.51.2.232

GARDNERMORSE M, 1995, J ORTHOP RES, V13, P802, DOI 10.1002/jor.1100130521

Granata KP, 2001, CLIN BIOMECH, V16, P650, DOI 10.1016/S0268-0033(01)00064-X

Graziano MSA, 1998, CURR OPIN NEUROBIOL, V8, P195, DOI 10.1016/S0959-4388(98)80140-2

GRAZIANO MSA, 1994, SCIENCE, V266, P1054, DOI 10.1126/science.7973661

Grivas TB, 2007, SCOLIOSIS SPINAL DIS, V2, DOI 10.1186/1748-7161-2-6

Hawes Martha, 2006, Pediatr Rehabil, V9, P318, DOI 10.1080/13638490500402264

Hedberg A, 2004, EXP BRAIN RES, V157, P10, DOI 10.1007/s00221-003-1811-z

Henry SM, 1998, J NEUROPHYSIOL, V80, P1939, DOI 10.1152/jn.1998.80.4.1939

HERMAN R, 1985, SPINE, V10, P1, DOI 10.1097/00007632-198501000-00001

HIRSCHFELD H, 1994, EXP BRAIN RES, V97, P528

Hodges P W, 2000, J Sci Med Sport, V3, P243, DOI 10.1016/S1440-2440(00)80033-X

Hodges PW, 2000, GAIT POSTURE, V11, P92, DOI 10.1016/S0966-6362(99)00055-7

Hodges PW, 1998, J SPINAL DISORD, V11, P46

Hodges PW, 1997, EXP BRAIN RES, V114, P362, DOI 10.1007/PL00005644

Hodges PW, 1996, SPINE, V21, P2640, DOI 10.1097/00007632-199611150-00014

Hodges PW, 2001, EXP BRAIN RES, V138, P243, DOI 10.1007/s002210100693

Horak FB, 2006, AGE AGEING, V35, P7, DOI 10.1093/ageing/afl077

HORAK FB, 1986, J NEUROPHYSIOL, V55, P1369, DOI 10.1152/jn.1986.55.6.1369

Hsu WL, 2007, J NEUROPHYSIOL, V97, P3024, DOI 10.1152/jn.01142.2006

JOHANSSON H, 1991, CLIN ORTHOP RELAT R, P161

Jones G.M., 2000, PRINCIPLES NEURAL SC, P816

Karnath HO, 2003, PHYS THER, V83, P1119, DOI 10.1093/ptj/83.12.1119

Karnath HO, 2000, NEUROLOGY, V55, P1298, DOI 10.1212/WNL.55.9.1298

Karnath HO, 2000, P NATL ACAD SCI USA, V97, P13931, DOI 10.1073/pnas.240279997

KESHNER EA, 1988, EXP BRAIN RES, V71, P455, DOI 10.1007/BF00248739

Kuypers H.G.J.M., 1985, SCI BASIS CLIN NEURO, P3

LACKNER JR, 1988, BRAIN, V111, P281, DOI 10.1093/brain/111.2.281

Ladavas E, 2002, TRENDS COGN SCI, V6, P17, DOI 10.1016/S1364-6613(00)01814-3

Lateiner JE, 2003, EXP BRAIN RES, V151, P446, DOI 10.1007/s00221-003-1503-8

Lee LJ, 2009, J ELECTROMYOGR KINES, V19, P46, DOI 10.1016/j.jelekin.2007.06.015

LEHNERTSCHROTH CH, 2000, DREIDIMENSIONALE SKO

Lou E, 2002, Stud Health Technol Inform, V91, P401

Lowe TG, 2000, J BONE JOINT SURG AM, V82A, P1157, DOI 10.2106/00004623-200008000-00014

Mannion A F, 1998, Eur Spine J, V7, P289, DOI 10.1007/s005860050077

Maravita A, 2004, TRENDS COGN SCI, V8, P79, DOI 10.1016/j.tics.2003.12.008

McGill SM, 2003, J ELECTROMYOGR KINES, V13, P353, DOI 10.1016/S1050-6411(03)00043-9

Miller NH, 1996, J ORTHOP RES, V14, P994, DOI 10.1002/jor.1100140621

MOLLON G, ENCY MED CHIR

Mooney V, 2000, J SPINAL DISORD, V13, P102, DOI 10.1097/00002517-200004000-00002

Moseley G Lorimer, 2002, Spine (Phila Pa 1976), V27, pE29, DOI 10.1097/00007632-200201150-00013

Moseley GL, 2003, J PHYSIOL-LONDON, V547, P581, DOI 10.1113/jphysiol.2002.024950

NASHNER LM, 1976, EXP BRAIN RES, V26, P59

NEGRINI A, 2006, 3 INT C CONS MAN SPI

Negrini S, 2005, Eura Medicophys, V41, P183

Negrini S, 2006, SCOLIOSIS SPINAL DIS, V1, DOI 10.1186/1748-7161-1-4

NITZ AJ, 1986, AM SURGEON, V52, P273

Oddsson L I, 1990, Acta Physiol Scand Suppl, V595, P1

Perennou D, 2006, RESTOR NEUROL NEUROS, V24, P319

Rizzolatti G, 1997, SCIENCE, V277, P190, DOI 10.1126/science.277.5323.190

Simoneau M, 2006, BMC NEUROSCI, V7, DOI 10.1186/1471-2202-7-68

SINGER KP, 2000, CLIN ANATOMY MANAGEM, P1

STAGNARA P, 1990, REEDUCATION SCOLIOSE

Stokes IAF, 2006, SCOLIOSIS SPINAL DIS, V1, DOI 10.1186/1748-7161-1-16

Tsao H, 2008, J ELECTROMYOGR KINES, V18, P559, DOI 10.1016/j.jelekin.2006.10.012

van der Fits IBM, 1998, EXP BRAIN RES, V120, P202, DOI 10.1007/s002210050394

Veldhuizen AG, 2000, EUR SPINE J, V9, P178, DOI 10.1007/s005860000142

Weiss HR, 2007, SCOLIOSIS SPINAL DIS, V2, DOI 10.1186/1748-7161-2-2

Weiss HR, 2006, SCOLIOSIS SPINAL DIS, V1, DOI [10.1186/1748-7161-1-5, 10.1186/1748-7161-1-1]

Weiss HR, 2006, SCOLIOSIS SPINAL DIS, V1, DOI 10.1186/1748-7161-1-6

Wong MS, 2002, PROSTHET ORTHOT INT, V26, P139, DOI 10.1080/03093640208726637

ZATTARA M, 1988, J NEUROL NEUROSUR PS, V51, P956, DOI 10.1136/jnnp.51.7.956

2007, EVIDENCE BASED ISICO

NR 87

TC 44

Z9 47

U1 2

U2 24

PU TAYLOR & FRANCIS LTD

PI ABINGDON

PA 2-4 PARK SQUARE, MILTON PARK, ABINGDON OR14 4RN, OXON, united kingdom

SN 0963-8288

EI 1464-5165

J9 DISABIL REHABIL

JI Disabil. Rehabil.

PY 2008

VL 30

IS 10

BP 763

EP 771

DI 10.1080/17483100801921311

PG 9

WC Rehabilitation

WE Science Citation Index Expanded (SCI-EXPANDED); Social Science Citation Index (SSCI)

SC Rehabilitation

GA 308JS

UT WOS:000256386300004

PM 18432434

DA 2023-08-10

ER

PT J

AU Arlet, V

Reddi, V

AF Arlet, Vincent

Reddi, Vasantha

TI Adolescent idiopathic scoliosis

SO NEUROSURGERY CLINICS OF NORTH AMERICA

LA English

DT Article

ID NATURAL-HISTORY; RESEARCH-SOCIETY; DECOMPENSATION; COMMITTEE; CURVE;

BRACE

AB Conservative treatment with a brace continues to remain controversial. A recent study on the efficacy of brace treatment by the Scoliosis Research Society Study Group found it to be efficient, however, and recommends it. Surgical treatment of adolescent idiopathic scoliosis must be tailored to each individual need, type of curve, and surgeon's expertise. A straighter curve does not necessarily mean better for each curve. Recent advances in pedicle screw instrumentation, although powerful in the rate of correction achieved, carry a definitive increase in neurologic risk and must be used only when justified and by experienced teams.

C1 Univ Virginia, Div Scoliosis & Spine Surg, Charlottesville, VA 22903 USA.

Univ Virginia, Dept Orthoped Surg, Charlottesville, VA 22903 USA.

C3 University of Virginia; University of Virginia

RP Arlet, V (通讯作者)， Univ Virginia, Div Scoliosis & Spine Surg, 400 Ray C Hunt Dr,Suite 330, Charlottesville, VA 22903 USA.

EM va3e@hcsmail.mcc.virginia.edu

CR Arlet V, 2000, EUR SPINE J, V9, P156, DOI 10.1007/s005860050227

ARLET V, ADOLESCENT IDIOPATHI

ARLET V, 2006, EUR SPINE J, V15, P8

Benli I T, 1996, Eur Spine J, V5, P380, DOI 10.1007/BF00301965

Bridwell KH, 2000, SPINE, V25, P2392, DOI 10.1097/00007632-200009150-00020

Coe JD, 2006, SPINE, V31, P345, DOI 10.1097/01.brs.0000197188.76369.13

LONSTEIN JE, 1984, J BONE JOINT SURG AM, V66A, P1061, DOI 10.2106/00004623-198466070-00013

Miller NH, 1999, ORTHOP CLIN N AM, V30, P343, DOI 10.1016/S0030-5898(05)70091-2

Moreland Morey S, 2002, Stud Health Technol Inform, V91, P492

NACHEMSON AL, 1995, J BONE JOINT SURG AM, V77A, P815, DOI 10.2106/00004623-199506000-00001

Richards BS, 2005, SPINE, V30, P2068, DOI 10.1097/01.brs.0000178819.90239.d0

Roach JW, 1999, ORTHOP CLIN N AM, V30, P353, DOI 10.1016/S0030-5898(05)70092-4

ROWE DE, 1995, J BONE JOINT SURG AM, V77, P815

THOMPSON JP, 1990, SPINE, V15, P927, DOI 10.1097/00007632-199009000-00017

Weinstein SL, 2003, JAMA-J AM MED ASSOC, V289, P559, DOI 10.1001/jama.289.5.559

WEINSTEIN SL, 1986, SPINE, V11, P780, DOI 10.1097/00007632-198610000-00006

NR 16

TC 10

Z9 14

U1 0

U2 0

PU W B SAUNDERS CO-ELSEVIER INC

PI PHILADELPHIA

PA 1600 JOHN F KENNEDY BOULEVARD, STE 1800, PHILADELPHIA, PA 19103-2899 USA

SN 1042-3680

EI 1558-1349

J9 NEUROSURG CLIN N AM

JI Neurosurg. Clin. N. Am.

PD APR

PY 2007

VL 18

IS 2

BP 255

EP +

DI 10.1016/j.nec.2007.02.002

PG 6

WC Clinical Neurology; Surgery

WE Science Citation Index Expanded (SCI-EXPANDED)

SC Neurosciences & Neurology; Surgery

GA 184ZH

UT WOS:000247680600009

PM 17556126

DA 2023-08-10

ER

PT J

AU Mallau, S

Bollini, G

Jouve, JL

Assaiante, C

AF Mallau, Sophie

Bollini, Gerard

Jouve, Jean-Luc

Assaiante, Christine

TI Locomotor skills and balance strategies in adolescents idiopathic

scoliosis

SO SPINE

LA English

DT Article

DE locomotion; balance control; idiopathic scoliosis; kinematic analysis;

adolescent

ID SEGMENTAL MOVEMENTS; HEAD STABILIZATION; CHILDREN; WALKING; GAIT;

HUMANS; SYSTEM; TASKS

AB Study Design. Locomotor balance control assessment was performed to study the effect of idiopathic scoliosis on head-trunk coordination in 17 patients with adolescent idiopathic scoliosis (AIS) and 16 control subjects.

Objective. The aim of this study was to explore the functional effects of structural spinal deformations like idiopathic scoliosis on the balance strategies used during locomotion.

Summary of Background Data. Up to now, the repercussion of the idiopathic scoliosis on head-trunk coordination and balance strategies during locomotion is relatively unknown.

Methods. Seventeen patients with AIS (mean age 14 years 3 months, 10 degrees < Cobb angle > 30 degrees) and 16 control subjects (mean age 14 years 1 month) were tested during various locomotor tasks: walking on the ground, walking on a line, and walking on a beam. Balance control was examined in terms of rotation about the vertical axis (yaw) and on a frontal plane (roll). Kinematics of foot, pelvis, trunk, shoulder, and head rotations were measured with an automatic optical TV image processor in order to calculate angular dispersions and segmental stabilizations.

Results. Decreasing the walking speed is the main adaptive strategy used in response to balance problems in control subjects as well as patients with AIS. However, patients with AIS performed walking tasks more slowly than normal subjects (around 15%). Moreover, the pelvic stabilization is preserved, despite the structural changes affecting the spine. Lastly, the biomechanical defect resulting from idiopathic scoliosis mainly affects the yaw head stabilization during locomotion.

Conclusions. Patients with AIS show substantial similarities with control subjects in adaptive strategies relative to locomotor velocity as well as balance control based on segmental stabilization. In contrast, the loss of the yaw head stabilization strategies, mainly based on the use of vestibular information, probably reflects the presence of vestibular deficits in the patients with AIS.

C1 CNRS, DPA, UMR 6196, F-13402 Marseille 20, France.

Univ Timone, Ctr Hosp, Serv Chirurg Orthoped Infantile, Marseille, France.

C3 Centre National de la Recherche Scientifique (CNRS); UDICE-French

Research Universities; Aix-Marseille Universite; Assistance

Publique-Hopitaux de Marseille

RP Assaiante, C (通讯作者)，CNRS, DPA, UMR 6196, 31 Chemin Joseph Auguier, F-13402 Marseille 20, France.

EM assaiant@dpm.cnrs-mrs.fr

CR Assaiante C, 1998, NEUROSCI BIOBEHAV R, V22, P527, DOI 10.1016/S0149-7634(97)00040-7

ASSAIANTE C, 1995, HUM MOVEMENT SCI, V14, P13, DOI 10.1016/0167-9457(94)00048-J

ASSAIANTE C, 1993, EXP BRAIN RES, V93, P499

Chen PQ, 1998, CLIN BIOMECH, V13, pS52, DOI 10.1016/S0268-0033(97)00075-2

Crosbie J, 1997, GAIT POSTURE, V5, P6, DOI 10.1016/S0966-6362(96)01066-1

FERRIGNO G, 1985, IEEE T BIO-MED ENG, V32, P943, DOI 10.1109/TBME.1985.325627

Frigo C, 2003, CLIN BIOMECH, V18, P419, DOI 10.1016/S0268-0033(03)00028-7

Gauchard GC, 2001, SPINE, V26, P1052, DOI 10.1097/00007632-200105010-00014

Grasso R, 1998, NEUROSCI BIOBEHAV R, V22, P533, DOI 10.1016/S0149-7634(97)00041-9

GROSSMAN GE, 1989, J NEUROPHYSIOL, V62, P264, DOI 10.1152/jn.1989.62.1.264

HERMAN R, 1985, SPINE, V10, P1, DOI 10.1097/00007632-198501000-00001

HERMAN R, 1979, IDIOPATHIC SCOLIOSIS, P61

Kramers-de Quervain IA, 2004, EUR SPINE J, V13, P449, DOI 10.1007/s00586-003-0588-x

MALLAU S, 2006, THESIS U BOURGOGNE D

Mesure S, 1999, EXP BRAIN RES, V129, P573, DOI 10.1007/s002210050927

MORRIS NM, 1980, J YOUTH ADOLESCENCE, V9, P271, DOI 10.1007/BF02088471

Nadeau S, 2003, GAIT POSTURE, V18, P134, DOI 10.1016/S0966-6362(02)00070-X

PEDOTTI A, 1988, INT CONGR SER, V812, P167

POZZO T, 1990, EXP BRAIN RES, V82, P97

Rogol AD, 2002, J ADOLESCENT HEALTH, V31, P192, DOI 10.1016/S1054-139X(02)00485-8

ROUSIE D, 2005, GAIT POSTURE, V21, pS87

SAHLSTRAND T, 1979, ACTA ORTHOP SCAND, V50, P759, DOI 10.3109/17453677908991307

Syczewska M, 1999, CLIN BIOMECH, V14, P384, DOI 10.1016/S0268-0033(99)00003-0

THOMACHOT B, 1995, ACTUALITES REEDUCATI, P151

Wiener-Vacher SR, 1998, J PEDIATR-US, V132, P1028, DOI 10.1016/S0022-3476(98)70403-2

YAMAMOTO H, 1982, J PEDIATR ORTHOPED, V2, P521, DOI 10.1097/01241398-198212000-00011

NR 26

TC 47

Z9 50

U1 0

U2 15

PU LIPPINCOTT WILLIAMS & WILKINS

PI PHILADELPHIA

PA TWO COMMERCE SQ, 2001 MARKET ST, PHILADELPHIA, PA 19103 USA

SN 0362-2436

EI 1528-1159

J9 SPINE

JI SPINE

PD JAN 1

PY 2007

VL 32

IS 1

BP E14

EP E22

DI 10.1097/01.brs.0000251069.58498.eb

PG 9

WC Clinical Neurology; Orthopedics

WE Science Citation Index Expanded (SCI-EXPANDED)

SC Neurosciences & Neurology; Orthopedics

GA 123EI

UT WOS:000243278700025

PM 17202875

DA 2023-08-10

ER

PT J

AU Koumbourlis, AC

AF Koumbourlis, Anastassios C.

TI Scoliosis and the respiratory system

SO PAEDIATRIC RESPIRATORY REVIEWS

LA English

DT Review

ID ADOLESCENT IDIOPATHIC SCOLIOSIS; PULMONARY-FUNCTION; THORACIC SCOLIOSIS;

CONSECUTIVE SERIES; SPINAL-FUSION; ANTERIOR; CHILDREN; INSTRUMENTATION;

KYPHOSCOLIOSIS; OBSTRUCTION

AB Scoliosis is caused by the lateral displacement and rotation of the vertebral bodies. It is most common during periods of rapid somatic growth. Scoliosis impedes on the movement of the ribs, places the respiratory muscles at a mechanical disadvantage and displaces the various organs of the thoracic cavity. Scoliosis decreases the chest wall compliance directly and the lung compliance indirectly (due to progressive atelectasis and air-trapping), causing a significant increase in the work of breathing that, because of the associated respiratory muscle weakness may lead to chronic respiratory failure. Progressive pulmonary hypertension also constitutes a leading cause for morbidity and mortality. Scoliosis is not reversible, but it can be controlled. Routine screening should start early and continue until the child reaches skeletal maturation. Pulmonary function testing can provide an easy and reliable means for the evaluation and follow-up of the condition. (C) 2006 Elsevier Ltd. All rights reserved.

C1 Schneider Childrens Hosp, Albert Einstein Coll Med, New Hyde Pk, NY 11040 USA.

Schneider Childrens Hosp, Div Pulm Med, New Hyde Pk, NY 11040 USA.

C3 Northwell Health; North Shore University Hospital; Steven & Alexandra

Cohen Children's Medical Center of New York; Yeshiva University;

Northwell Health; North Shore University Hospital; Steven & Alexandra

Cohen Children's Medical Center of New York

RP Koumbourlis, AC (通讯作者)， Schneider Childrens Hosp, Albert Einstein Coll Med, New Hyde Pk, NY 11040 USA.

EM akoumbou@lij.edu

RI Koumbourlis, Anastassios Constantin/AAD-2038-2020

OI Koumbourlis, Anastassios C./0000-0002-4400-4885

CR Benson ER, 1998, SPINE, V23, P2308, DOI 10.1097/00007632-199811010-00012

Betz RR, 1999, SPINE, V24, P225, DOI 10.1097/00007632-199902010-00007

Borowitz D, 2001, PEDIATR PULM, V31, P86, DOI 10.1002/1099-0496(200101)31:1<86::AID-PPUL1012>3.0.CO;2-3

Boyer J, 1996, CHEST, V109, P1532, DOI 10.1378/chest.109.6.1532

Buyse B, 2003, EUR RESPIR J, V22, P525, DOI 10.1183/09031936.03.00076103

COOPER DM, 1984, AM REV RESPIR DIS, V130, P16

DAY GA, 1994, SPINE, V19, P1027, DOI 10.1097/00007632-199405000-00004

Dhuper S, 1997, PEDIATR CARDIOL, V18, P425, DOI 10.1007/s002469900220

Edwards Bryan T, 2003, J Long Term Eff Med Implants, V13, P437, DOI 10.1615/JLongTermEffMedImplants.v13.i6.10

Graham EJ, 2000, SPINE, V25, P2319, DOI 10.1097/00007632-200009150-00009

KAFER ER, 1977, B EUR PHYSIOPATH RES, V13, P299

Katsaris G, 1999, EUR SPINE J, V8, P2, DOI 10.1007/s005860050119

KEARON C, 1993, AM REV RESPIR DIS, V148, P295, DOI 10.1164/ajrccm/148.2.295

KEARON C, 1993, AM REV RESPIR DIS, V148, P288, DOI 10.1164/ajrccm/148.2.288

KINNEAR WJM, 1993, SPINE, V18, P1556

Korovessis P, 1996, SPINE, V21, P1979, DOI 10.1097/00007632-199609010-00008

Koukourakis I, 1997, J SPINAL DISORD, V10, P527

LISBOA C, 1985, AM REV RESPIR DIS, V132, P48

LONSTEIN JE, 1994, LANCET, V344, P1407

Miller NH, 2000, SPINE, V25, P2416

MURRAY J, 1986, NORMAL LUNG, P121

Newton PO, 2005, SPINE, V30, P392, DOI 10.1097/01.brs.0000153404.62017.75

Stirling AJ, 1996, J BONE JOINT SURG AM, V78A, P1330, DOI 10.2106/00004623-199609000-00006

TREVOR S, 1980, DEV MED CHILD NEUROL, V22, P675

Vedantam R, 2000, SPINE, V25, P82, DOI 10.1097/00007632-200001010-00015

NR 25

TC 129

Z9 157

U1 1

U2 17

PU ELSEVIER SCI LTD

PI OXFORD

PA THE BOULEVARD, LANGFORD LANE, KIDLINGTON, OXFORD OX5 1GB, OXON, united kingdom

SN 1526-0542

EI 1526-0550

J9 PAEDIATR RESPIR REV

JI Paediatr. Respir. Rev.

PD JUN

PY 2006

VL 7

IS 2

BP 152

EP 160

DI 10.1016/j.prrv.2006.04.009

PG 9

WC Pediatrics; Respiratory System

WE Science Citation Index Expanded (SCI-EXPANDED)

SC Pediatrics; Respiratory System

GA 116MN

UT WOS:000242807100013

PM 16765303

DA 2023-08-10

ER

PT J

AU Haefeli, M

Elfering, A

Kilian, R

Min, K

Boos, N

AF Haefeli, M

Elfering, A

Kilian, R

Min, K

Boos, N

TI Nonoperative treatment for adolescent idiopathic scoliosis - A 10-to 60-year follow-up with special reference to health-related quality of life

SO SPINE

LA English

DT Article

DE adolescent idiopathic scoliosis; nonoperative treatment; health-related

quality of life; long-term follow-up; disability; pain; psychological

well-being

ID SAGITTAL SPINAL ALIGNMENT; NATURAL-HISTORY; VERTEBRAL ROTATION;

SURGICAL-TREATMENT; SKELETAL MATURITY; CURVE PROGRESSION; BACK-PAIN;

VOLUNTEERS; PLANE; BRACE

AB Study Design. Retrospective study on patients 10 to 60 years of age after nonoperative treatment for adolescent idiopathic scoliosis (AIS).

Objectives. To investigate long-term outcome with regard to pain, disability, psychological disturbance, and health-related quality of life (HRQOL) in nonoperatively treated patients with AIS.

Summary of Background Data. Only little is known on the long-term quality of life and disability in patients nonoperatively treated for AIS. A detailed knowledge of the nonoperative treatment results is important when advising patients for surgery.

Methods. A total of 135 nonoperatively treated AIS patients with a minimum follow-up of 10 years were included in this investigation, 121 of whom responded to a questionnaire containing questions on pain, disability (Oswestry Disability Index [ODI], Hannover Functional Ability Questionnaire [HFAQ], psychological general well-being [PGWB], and health-related quality of life [WHOQOL(BREF)]). Eighty-one patients participated in a clinical/radiologic follow-up examination. Nonoperative treatment consisted of bracing (n = 60), physiotherapy (n = 59), and electrical stimulation (n = 2). The overall follow-up rate was 89.6%. The mean age at follow-up was 38.0 years (range, 20-73 years.).

Results. In general, patients achieved a satisfactory outcome 10 to 60 years (mean, 23 years) after nonoperative treatment with regard to pain, disability, and HRQOL. The average curve at first diagnosis measured 29.5 (range, 15-59) for the thoracic spine, 21.3 degrees (range, 15 degrees-28 degrees) for the thoracolumbar spine, and 26.8 degrees (10 degrees-44 degrees) for the lumbar spine. Thirteen patients showed a substantial change in curve size (+/- 10 degrees) between first diagnosis and end of growth: 11 curves progressed more than 10 showing an average increase of 19.0 degrees (range, 12 degrees-30 degrees) and 2 patients presented with less severe curves at follow-up (-10 degrees and -13 degrees). After end of growth, 7 patients showed a substantial average increase of 16.3 degrees (range, 10 degrees-31 degrees). Five of eight patients with thoracic curves greater than 80 degrees had restrictive pulmonary disease. Patients with curves greater than 45 degrees reported significantly higher pain levels than those with smaller curves. Patients only showed a minimal absolute disability (Oswestry and HFAQ), and no significant correlation was found between curve size and curve type, respectively. Compared with a healthy control group that was matched for age and gender, no significant differences were found in terms of HRQOL as assessed by the

WHOQOL(BREF) questionnaire. No significant differences in pain, disability, or HRQOL were found between patients with and without brace treatment.

Conclusions. Although pain, disability, HRQOL, and psychological general well-being are quite satisfactory on an absolute level, curve size was found to be a significant predictor for pain in a long-term follow-up.

C1 Univ Zurich, Ctr Spinal Surg, CH-8008 Zurich, Switzerland.

Univ Bern, Dept Psychol, Bern, Switzerland.

Univ Ulm, Dept Psychiat 2, Bezirkskrankenhaus Gunzburg, Ulm, Germany.

C3 University of Zurich; University of Bern; Ulm University

RP Boos, N (通讯作者)， Univ Zurich, Univ Hosp Balgrist, Ctr Spinal Surg, Forchstr 340, CH-8008 Zurich, Switzerland.

EM Norbert.Boos@Balgrist.ch

CR ANGERMEYER C, 2000, HDB DEUTSCHSPARCHIGE

[Anonymous], 2000, SRS TERMINOLOGY COMM

[Anonymous], 1990, Z F R DIFFERENTIELLE

[Anonymous], 1948, AM ACAD ORTHOP SURG

ASCANI E, 1986, SPINE, V11, P784, DOI 10.1097/00007632-198610000-00007

Asher MA, 2000, SPINE, V25, P2381, DOI 10.1097/00007632-200009150-00018

BARSANTI CM, 1990, J PEDIATR ORTHOPED, V10, P527

Bridwell KH, 1999, SPINE, V24, P2607, DOI 10.1097/00007632-199912150-00008

BUNNELL WP, 1986, SPINE, V11, P773, DOI 10.1097/00007632-198610000-00003

CARMAN DL, 1990, J BONE JOINT SURG AM, V72A, P328, DOI 10.2106/00004623-199072030-00003

COLLIS DK, 1969, J BONE JOINT SURG AM, VA 51, P425, DOI 10.2106/00004623-196951030-00001

Danielsson AJ, 2001, SPINE, V26, P516

Danielsson AJ, 2001, EUR SPINE J, V10, P278, DOI 10.1007/s005860100309

Danielsson AJ, 2003, SPINE, V28, P2078

Danielsson AJ, 2003, SPINE, V28, P86

de Jonge T, 2002, SPINE, V27, P754, DOI 10.1097/00007632-200204010-00013

Dupuy H. J., 1984, PSYCHOL GEN WELL BEI, P170

DURIEZ J, 1967, ACTA ORTHOP BELG, V33, P547

EDGAR MA, 1988, J BONE JOINT SURG BR, V70, P712, DOI 10.1302/0301-620X.70B5.3192566

Fairbank J C, 1980, Physiotherapy, V66, P271

FALLSTROM K, 1986, SPINE, V11, P756

GELB DE, 1995, SPINE, V20, P1351, DOI 10.1097/00007632-199506000-00005

Guigui P, 2003, REV CHIR ORTHOP, V89, P496

Haase I, 2001, Rehabilitation (Stuttg), V40, P40

Haher TR, 1999, SPINE, V24, P1435, DOI 10.1097/00007632-199907150-00008

Harper A, 1998, PSYCHOL MED, V28, P551, DOI 10.1017/S0033291798006667

JACKSON RP, 1994, SPINE, V19, P1611, DOI 10.1097/00007632-199407001-00010

KING HA, 1983, J BONE JOINT SURG AM, V65, P1302, DOI 10.2106/00004623-198365090-00012

Kohlmann T, 1996, Rehabilitation (Stuttg), V35, pI

Lautenschlager J, 1997, Z RHEUMATOL, V56, P144, DOI 10.1007/s003930050030

MACLEAN WE, 1989, J PEDIATR ORTHOPED, V9, P257

MOE JH, 1978, SCOLIOSIS OTHER SPIN, P429

MOE JH, 1978, SCOLIOSIS OTHER SPIN, P325

NASH CL, 1969, J BONE JOINT SURG AM, VA 51, P223, DOI 10.2106/00004623-196951020-00002

Noonan KJ, 1997, J PEDIATR ORTHOPED, V17, P712, DOI 10.1097/00004694-199711000-00004

Payne WK, 1997, SPINE, V22, P1380, DOI 10.1097/00007632-199706150-00017

PERDRIOLLE R, 1981, REV CHIR ORTHOP, V67, P25

PERDRIOLLE R, 1985, SPINE, V10, P785, DOI 10.1097/00007632-198511000-00001

PERDRIOLLE R, 1979, PARIS MALOCHE

PONSETI IV, 1950, J BONE JOINT SURG AM, V32-A, P381, DOI 10.2106/00004623-195032020-00017

Roese I, 1996, Rehabilitation (Stuttg), V35, P103

STAGNARA P, 1982, SPINE, V7, P335, DOI 10.1097/00007632-198207000-00003

Vedantam R, 1998, SPINE, V23, P211, DOI 10.1097/00007632-199801150-00012

WEINSTEIN SL, 1983, J BONE JOINT SURG AM, V65, P447, DOI 10.2106/00004623-198365040-00004

WEINSTEIN SL, 1981, J BONE JOINT SURG AM, V63, P702, DOI 10.2106/00004623-198163050-00003

Weinstein SL, 2003, JAMA-J AM MED ASSOC, V289, P559, DOI 10.1001/jama.289.5.559

NR 46

TC 52

Z9 54

U1 0

U2 16

PU LIPPINCOTT WILLIAMS & WILKINS

PI PHILADELPHIA

PA TWO COMMERCE SQ, 2001 MARKET ST, PHILADELPHIA, PA 19103 USA

SN 0362-2436

EI 1528-1159

J9 SPINE

JI SPINE

PD FEB 1

PY 2006

VL 31

IS 3

BP 355

EP 366

DI 10.1097/01.brs.0000197664.02098.09

PG 12

WC Clinical Neurology; Orthopedics

WE Science Citation Index Expanded (SCI-EXPANDED)

SC Neurosciences & Neurology; Orthopedics

GA 008AE

UT WOS:000235011800016

PM 16449911

DA 2023-08-10

ER

PT J

AU Lenssinck, MLB

Frijlink, AC

Berger, MY

Bierma-Zeinstra, SMA

Verkerk, K

Verhagen, AP

AF Lenssinck, MLB

Frijlink, AC

Berger, MY

Bierma-Zeinstra, SMA

Verkerk, K

Verhagen, AP

TI Effect of bracing and other conservative interventions in the treatment

of idiopathic scoliosis in adolescents: A systematic review of clinical

trials

SO PHYSICAL THERAPY

LA English

DT Article

DE back pain; bracing; evidence-based practice; exercise movement

techniques; idiopathic scoliosis; immobilization; pediatrics;

rehabilitation; scoliosis; spinal curvatures; systematic review;

treatment outcome

ID MILWAUKEE BRACE; QUALITY ASSESSMENT; BOSTON BRACE; FOLLOW-UP; EXERCISES;

IMPACT; IMAGE; GIRLS

AB Background and Purpose. Many conservative treatments are available for adolescents with idiopathic scoliosis, but the evidence for their accepted use is still unclear. The purpose of this study was to evaluate the effectiveness of braces and other conservative treatments of idiopathic scoliosis in adolescents by systematically reviewing the literature.

Methods. The literature was searched in the PubMed, CINAHL, Cochrane, and PEDro databases. Studies were selected if the design was a randomized clinical trial or a controlled clinical trial, if all patients had an idiopathic scollosis, if all patients were less than 18 years of age during the intervention, and if the type of intervention was a conservative one. Two reviewers independently assessed the methodological quality using the Delphi list and performed data extraction. Analysis was based on the levels of evidence.

Results. Thirteen studies met the final inclusion criteria, showing a wide range of interventions such as bracing, electrical surface stimulation, and exercises.

Discussion and Conclusion. The authors conclude that the effectiveness of bracing and exercises is not yet established, but might be promising. They found no evidence of the effectiveness of electrical stimulation. [Lenssinck M-LB, Frijlink AC, Berger MY, et al. Effect of bracing and other conservative interventions in the treatment of idiopathic scoliosis in adolescents: a systematic review of clinical trials.

C1 Erasmus MC, Dept Gen Practice, NL-3000 DR Rotterdam, Netherlands.

Hogesch Rotterdam, Dept Physiotherapy, Rotterdam, Netherlands.

C3 Erasmus University Rotterdam; Erasmus MC

RP Lenssinck, MLB (通讯作者)， Erasmus MC, Dept Gen Practice, POB 1738, NL-3000 DR Rotterdam, Netherlands.

RI Bierma-Zeinstra, Sita/AAF-2122-2019

OI Verhagen, Arianne/0000-0002-6195-0128

CR Athanasopoulos S, 1999, SCAND J MED SCI SPOR, V9, P36

BIRBAUMER N, 1994, J PSYCHOSOM RES, V38, P623, DOI 10.1016/0022-3999(94)90060-4

BURNS YR, 1996, PHYSIOTHERAPY GROWIN, P332

CARMAN D, 1985, J PEDIATR ORTHOPED, V5, P65, DOI 10.1097/01241398-198501000-00011

CHALMERS TC, 1981, CONTROL CLIN TRIALS, V2, P31, DOI 10.1016/0197-2456(81)90056-8

Climent JM, 1999, SPINE, V24, P1903, DOI 10.1097/00007632-199909150-00007

den Boer WA, 1999, EUR SPINE J, V8, P406, DOI 10.1007/s005860050195

Dickson RA, 1999, J BONE JOINT SURG BR, V81B, P193, DOI 10.1302/0301-620X.81B2.9630

DICKSON RA, 1978, ACTA ORTHOP SCAND, V49, P46, DOI 10.3109/17453677809005722

el-Sayyad M, 1994, Int J Rehabil Res, V17, P70, DOI 10.1097/00004356-199403000-00008

FALLSTROM K, 1986, SPINE, V11, P756

FERNANDEZFELIBERTI R, 1995, J PEDIATR ORTHOPED, V15, P176

Ferraro C., 1998, EUR MEDICOPHYS, V34, P25

FIORE N, 1988, REV CHIR ORTHOP, V74, P569

Gepstein R, 2002, J PEDIATR ORTHOPED, V22, P84, DOI 10.1097/00004694-200201000-00018

Goldberg CJ, 2001, SPINE, V26, P42, DOI 10.1097/00007632-200101010-00009

Haasbeek JF, 1997, POSTGRAD MED, V101, P207, DOI 10.3810/pgm.1997.06.230

Jadad AR, 1998, ARCH PEDIAT ADOL MED, V152, P812

MINAMI S, 1982, Journal of the Japanese Orthopaedic Association, V56, P471

Moher D, 1996, INT J TECHNOL ASSESS, V12, P195, DOI 10.1017/S0266462300009570

MULCAHY T, 1973, CLIN ORTHOP RELAT R, V93, P53

NACHEMSON AL, 1995, J BONE JOINT SURG AM, V77A, P815, DOI 10.2106/00004623-199506000-00001

Noonan KJ, 1996, J BONE JOINT SURG AM, V78A, P557, DOI 10.2106/00004623-199604000-00009

Olafsson Y, 1999, EUR SPINE J, V8, P402, DOI 10.1007/s005860050194

Payne WK, 1997, SPINE, V22, P1380, DOI 10.1097/00007632-199706150-00017

PEHRSSON K, 1992, SPINE, V17, P1091, DOI 10.1097/00007632-199209000-00014

Reamy BV, 2001, AM FAM PHYSICIAN, V64, P111

Robinson KA, 2002, INT J EPIDEMIOL, V31, P150, DOI 10.1093/ije/31.1.150

Rowe DE, 1997, J BONE JOINT SURG AM, V79A, P664, DOI 10.2106/00004623-199705000-00005

Sapountzi-Krepia DS, 2001, J ADV NURS, V35, P683, DOI 10.1046/j.1365-2648.2001.01900.x

SCHLENZKA D, 1990, Beitraege zur Orthopaedie und Traumatologie, V37, P373

Skaggs DL, 1996, AM FAM PHYSICIAN, V53, P2327

Smidt N, 2002, PAIN, V96, P23, DOI 10.1016/S0304-3959(01)00388-8

van Tulder M, 2003, SPINE, V28, P1290, DOI 10.1097/00007632-200306150-00014

Vandal S, 1999, Issues Compr Pediatr Nurs, V22, P59

Verhagen AP, 2001, J CLIN EPIDEMIOL, V54, P651, DOI 10.1016/S0895-4356(00)00360-7

Verhagen AP, 1998, J CLIN EPIDEMIOL, V51, P1235, DOI 10.1016/S0895-4356(98)00131-0

VONDEIMLING U, 1995, Z ORTHOP GRENZGEB, V133, P270, DOI 10.1055/s-2008-1039447

Weiss Hans-Rudolf, 2003, Pediatr Rehabil, V6, P23, DOI 10.1080/13638490310000095288

NR 39

TC 107

Z9 112

U1 1

U2 21

PU OXFORD UNIV PRESS INC

PI CARY

PA JOURNALS DEPT, 2001 EVANS RD, CARY, NC 27513 USA

SN 0031-9023

EI 1538-6724

J9 PHYS THER

JI Phys. Ther.

PD DEC

PY 2005

VL 85

IS 12

BP 1329

EP 1339

DI 10.1093/ptj/85.12.1329

PG 11

WC Orthopedics; Rehabilitation

WE Science Citation Index Expanded (SCI-EXPANDED)

SC Orthopedics; Rehabilitation

GA 997RG

UT WOS:000234264300006

PM 16305271

DA 2023-08-10

ER

PT J

AU Otman, S

Kose, N

Yakut, Y

AF Otman, S

Kose, N

Yakut, Y

TI The efficacy of Schroth's 3-dimensional exercise therapy in the

treatment of adolescent idiopathic scoliosis in Turkey

SO SAUDI MEDICAL JOURNAL

LA English

DT Article

AB Objective: To determine the effectiveness of 3-dimensional therapy in the treatment of adolescent idiopathic scoliosis.

Methods: We carried out this Study with 50 patients whose average age was 14.15 +/- 1.69 years at the Physical Therapy and Rehabilitation School, Hacettepe University, Ankara, Turkey, from 1999 to 2004. We treated them as outpatients, 5 days a week, in a 4-hour program for the first 6 weeks. After that, they continued with the same program at home. We evaluated the Cobb angle, vital capacity and muscle strength of the patients before treatment, and after 6 weeks, 6 months and one year, and compared all the results.

Results: The average Cobb angle, which was 26.10 degrees on average before treatment, was 23.45 degrees after 6 weeks, 19.25 degrees after 6 months and 17.85 degrees after one year ($p < 0.01$). The vital capacities, which were on average 2795 ml before treatment, reached 2956 ml after 6 weeks, 3125 ml after 6 months and 3215 ml after one year ($p < 0.01$). Similarly, according to the results of evaluations after 6 weeks, 6 months and one year, we observed an increase in muscle strength and recovery of the postural defects in all patients ($p < 0.01$).

Conclusion: Schroth's technique positively influenced the Cobb angle, vital capacity, strength and postural defects in Outpatient adolescents.

C1 Univ Hacettepe, Sch Phys Therapy & Rehabil, TR-06100 Ankara, Turkey.

C3 Hacettepe University

RP Otman, S (通讯作者)，Univ Hacettepe, Sch Phys Therapy & Rehabil, TR-06100 Ankara, Turkey.

EM sotman@hacettepe.edu.tr

CR [Anonymous], P 11 INT C WORLD CON
[Anonymous], 1992, PHYSIOTHERAPY
Burgoyne W, 2001, CURRENT PAEDIAT, V11, P323
Ebenbichler G, 1994, Wien Med Wochenschr, V144, P593
HANSEN S, 1985, 3 DIMENSIONAL THERAP
Hawes Martha C, 2003, Pediatr Rehabil, V6, P171
Kendall F.P., 1993, MUSCLE TESTING FUNCT, V4
LaMontagne LL, 2004, APPL NURS RES, V17, P168, DOI 10.1016/j.apnr.2004.06.007
LEHNERTSCHROTH C, 1992, PHYSIOTHERAPY, V78, P810
Lin MC, 2001, ARCH PHYS MED REHAB, V82, P335, DOI 10.1053/apmr.2001.21528
Maruyama Toru, 2003, Pediatr Rehabil, V6, P215
Negrini Stefano, 2003, Pediatr Rehabil, V6, P227, DOI: 10.1080/13638490310001636781
Reamy BV, 2001, AM FAM PHYSICIAN, V64, P111
Stehbens WE, 2003, EXP MOL PATHOL, V74, P326, DOI 10.1016/S0014-4800(02)00014-X
Weiss H R, 1992, Ital J Orthop Traumatol, V18, P395
Weiss Hans-Rudolf, 2003, Pediatr Rehabil, V6, P183
Weiss Hans-Rudolf, 2002, Stud Health Technol Inform, V91, P352
Weiss Hans-Rudolf, 2002, Stud Health Technol Inform, V91, P342
Weiss Hans-Rudolf, 2003, Pediatr Rehabil, V6, P111, DOI 10.1080/13638490310001593446
Weiss HR, 2003, ORTHOPADE, V32, P146, DOI 10.1007/s00132-002-0430-x
WEISS HR, 1991, SPINE, V16, P88, DOI 10.1097/00007632-199101000-00016
WIESS HR, 2003, PEDIAT REHABIL, V6, P23

NR 22

TC 50

Z9 54

U1 5

U2 33

PU SAUDI MED J

PI RIYADH

PA ARMED FORCES HOSPITAL, PO BOX 7897,, RIYADH 11159, SAUDI ARABIA

SN 0379-5284

J9 SAUDI MED J

JI Saudi Med. J.

PD SEP

PY 2005

VL 26

IS 9

BP 1429

EP 1435

PG 7

WC Medicine, General & Internal

WE Science Citation Index Expanded (SCI-EXPANDED)

SC General & Internal Medicine

GA 974BM

UT WOS:000232566300020

PM 16155663

DA 2023-08-10

ER

PT J

AU Barrios, C

Perez-Encinas, C

Maruenda, JI

Laguia, M

AF Barrios, C

Perez-Encinas, C

Maruenda, JI

Laguia, M

TI Significant ventilatory functional restriction in adolescents with mild

or moderate scoliosis during maximal exercise tolerance test

SO SPINE

LA English

DT Article

DE pulmonary function; ventilatory efficiency; exercise tolerance test;

maximal oxygen uptake; adolescent idiopathic scoliosis

ID PULMONARY-FUNCTION; IDIOPATHIC SCOLIOSIS; CAPACITY; BRACE

AB Study Design. A prospective evaluation of cardiopulmonary tolerance to maximal exercise in adolescent idiopathic scoliosis.

Objectives. To evaluate ventilatory functional restrictions during a maximal exercise tolerance test in idiopathic scoliosis patients with mild and moderate curves and to compare them with the results obtained in healthy adolescents matched in age undergoing similar test.

Summary of Background Data. Adolescents with idiopathic scoliosis with mild curves do not exhibit significant restrictions in ventilatory parameters measured by conventional static spirometry. Few reports have dealt with cardiorespiratory response to maximal exercise in adolescent idiopathic scoliosis with mild to moderate curves. Although results seem to show a reduced exercise tolerance in these patients, the frequency and signification of the restricted work capacity is uncertain because of important design limitations in previous studies.

Methods. Thirty-seven girls diagnosed with adolescent idiopathic scoliosis with a mean age of 13 years (range, 11-16) and an average scoliotic curve of 32.8 degrees Cobb (range, 20-45 degrees) were studied by basal spirometry and dynamic ventilatory parameters during a maximal exercise tolerance test. Similar studies were performed in a control group of 10 healthy girls matched in age. Exercise test consisted of a ramp protocol on treadmill starting at a speed of 0.75 m/second (2.7 km/hour) with increments of 0.2 m/second (0.72 km/hour) per minute. All subjects completed the

test to exhaustion to determine maximal oxygen uptake (VO2 (max)) and ventilatory efficiency parameters.

Results. There were no differences between scoliotic and healthy girls in basal ventilatory parameters (FVC, FEV1). However, adolescents with idiopathic scoliosis showed worse tolerance to exercise test with lower maximal speed average (9.4 km/hour versus 11.5 km/hour, P < 0.005), lower ventilatory efficiency at maximal exercise (VE: 68.9 L/minute versus 82.3 L/minute, P < 0.01), early anaerobic threshold, and a lower aerobic power expressed by 23% decreased body weight normalized VO2 (max) (38.6 mL/kg/minute versus 49.0 mL/kg/minute, P < 0.001). VEmax values were correlated to the severity of the scoliotic curve. Patients with more severe curves had greater limitation of ventilatory capacity (r = -0.374, P < 0.05). Maximal breath frequency was higher in scoliotic girls (54 versus 47, P < 0.05) suggesting a compensatory mechanism adopted in response to the lower ventilatory capacity during demanding exercise. When ventilatory efficiency was considered by the VE/VO2 ratio, scoliotic girls disclosed higher values than control (average 35.2 versus 29.6, P < 0.001) indicating an inefficiency in their ventilation. Patients wearing a brace at the time of ventilatory functional assessment did not exhibit any difference in the parameters investigated both at basal spirometry and during exercise tolerance test.

Conclusion. Although patients with mild or moderate scoliosis do not exhibit cardiopulmonary restrictions in basal static conditions, they do show a significant lower tolerance to maximal exercise. Respiratory inefficiency together with lower ventilation capacity and lower VO2 (max) may be responsible for reduced exercise tolerance in adolescents with idiopathic scoliosis. Exercise deconditioning in scoliotic patients cannot be attributed to brace treatment.

C1 Univ Valencia, Sch Med, Dept Surg, Orthopaed & Trauma Unit, Valencia 46010, Spain.

Univ Valencia, Clin Hosp, Inst Sports Med, Valencia, Spain.

Univ Valencia, Clin Hosp, Dept Orthopaed, Valencia, Spain.

C3 University of Valencia; University of Valencia; University of Valencia

RP Barrios, C (通讯作者)， Univ Valencia, Sch Med, Dept Surg, Orthopaed & Trauma Unit, Avda Blasco Ibanez 17, Valencia 46010, Spain.

EM cbarrios@uv.es

RI Colomina, Maria J/C-4207-2014

OI Colomina, Maria J/0000-0003-0106-0956; Barrios,

Carlos/0000-0002-6657-7246

CR [Anonymous], 1986, TXB WORK PHYSL PHYSL

CHONG KC, 1981, J PEDIATR ORTHOPED, V1, P251, DOI 10.1097/01241398-198111000-00002

DIROCCO PJ, 1988, ARCH PHYS MED REHAB, V69, P198

Graham EJ, 2000, SPINE, V25, P2319, DOI 10.1097/00007632-200009150-00009

KAFER ER, 1980, ANESTHESIOLOGY, V52, P339, DOI 10.1097/00000542-198004000-00012

Katsaris G, 1999, EUR SPINE J, V8, P2, DOI 10.1007/s005860050119

KEARON C, 1993, AM REV RESPIR DIS, V148, P295, DOI 10.1164/ajrccm/148.2.295

KESTEN S, 1991, CHEST, V99, P663, DOI 10.1378/chest.99.3.663

Korovessis P, 1996, SPINE, V21, P1979, DOI 10.1097/00007632-199609010-00008

LEECH JA, 1985, J PEDIATR-US, V106, P143, DOI 10.1016/S0022-3476(85)80487-X

Lenke LG, 2002, SPINE, V27, P2041, DOI 10.1097/00007632-200209150-00014

Leong JCY, 1999, SPINE, V24, P1310, DOI 10.1097/00007632-199907010-00007

Pehrsson K, 2001, THORAX, V56, P388, DOI 10.1136/thorax.56.5.388

SHNEERSON JM, 1980, THORAX, V35, P347, DOI 10.1136/thx.35.5.347

SMYTH RJ, 1986, PEDIATRICS, V77, P692

Vedantam R, 1997, SPINE, V22, P2731, DOI 10.1097/00007632-199712010-00006

VENDANTAM R, 2000, SPINE, V25, P82

WEBER B, 1975, AM REV RESPIR DIS, V111, P389

Weise K, 2004, HASTINGS CENT REP, V34, P8, DOI 10.2307/3528683

WONG CA, 1966, THORAX, V51, P543

NR 20

TC 64

Z9 71

U1 0

U2 10

PU LIPPINCOTT WILLIAMS & WILKINS

PI PHILADELPHIA

PA TWO COMMERCE SQ, 2001 MARKET ST, PHILADELPHIA, PA 19103 USA

SN 0362-2436

EI 1528-1159

J9 SPINE

JI SPINE

PD JUL 15

PY 2005

VL 30

IS 14

BP 1610

EP 1615

DI 10.1097/01.brs.0000169447.55556.01

PG 6

WC Clinical Neurology; Orthopedics

WE Science Citation Index Expanded (SCI-EXPANDED)

SC Neurosciences & Neurology; Orthopedics

GA 945QR

UT WOS:000230517700007

PM 16025029

DA 2023-08-10

ER

PT J

AU Bayar, B

Uygur, F

Bayar, K

Bek, N

Yakut, Y

AF Bayar, B

Uygur, F

Bayar, K

Bek, N

Yakut, Y

TI The short term effects of an exercise programme as an adjunct to an

orthosis in neuromuscular scoliosis

SO PROSTHETICS AND ORTHOTICS INTERNATIONAL

LA English

DT Article

ID DUCHENNE MUSCULAR-DYSTROPHY; SPINAL-FUSION; DISEASE; DEFORMITY; BRACE

AB The purpose of this study was to investigate the effects of a 4 week physiotherapy programme on patients who were given a spinal orthosis for neuromuscular scoliosis. This study was planned as a single group pre- and post-intervention repeated measures design. All patients were given a polyethylene spinal orthosis with an anterior opening. Fifteen (15) patients with neuromuscular diseases and a mean age of 12.46 years were evaluated. An exercises programme consisting of postural training, muscle strengthening and stretching exercises with special emphasis on respiratory exercises was given as an adjunct to orthotic treatment. The degree of impairment in forced vital capacity was 17.56% upon wearing an orthosis, it decreased to 9.28% following therapy ($p<0.05$). There was also a statistically significant increase in muscle strength, balance duration and a significant decrease in limitation of range of motion. The results of the study imply that the conservative treatment of neuromuscular scoliosis should include an exercise programme as an adjunct to an orthosis, both to reduce the compromising effect of an orthosis on respiratory function and to support the patient's physical capacities.

C1 Univ Hacettepe, Sch Phys Therapy & Rehabil, Unit Orthot & Biomech, TR-06100 Ankara, Turkey.

C3 Hacettepe University

RP Bayar, B (通讯作者)， Univ Hacettepe, Sch Phys Therapy & Rehabil, Unit Orthot & Biomech, TR-06100 Ankara, Turkey.

EM bkurklu@hacettepe.edu.tr

RI YAKUT, YAVUZ/J-1174-2013; BAYAR, KILICHAN/ITT-9243-2023; BAYAR, Banu/ITT-9151-2023

CR Benson ER, 1998, SPINE, V23, P2308, DOI 10.1097/00007632-199811010-00012

BRADFORD DS, 1987, MOES TXB SCOLIOSIS O

Cambach W, 1999, ARCH PHYS MED REHAB, V80, P103, DOI 10.1016/S0003-9993(99)90316-7

CAMBRIDGE W, 1987, J PEDIATR ORTHOPED, V7, P436, DOI 10.1097/01241398-198707000-00011

Daniels L, 1972, MUSCLE TESTING TECHN

GALASKO CSB, 1992, J BONE JOINT SURG BR, V74, P210, DOI 10.1302/0301-620X.74B2.1544954

HENSINGER RN, 1976, J BONE JOINT SURG AM, V58, P13, DOI 10.2106/00004623-197658010-00003

LETTS M, 1992, J PEDIATR ORTHOPED, V12, P470, DOI 10.1097/01241398-199207000-00010

MALONEY WJ, 1990, J PEDIATR ORTHOPED, V10, P742, DOI 10.1097/01241398-199011000-00007

Miller A, 1996, J PEDIATR ORTHOPED, V16, P332, DOI 10.1097/01241398-199605000-00007

NOBLEJAMIESON CM, 1986, ARCH DIS CHILD, V61, P178, DOI 10.1136/adc.61.2.178

Olafsson Y, 1999, J PEDIATR ORTHOPED, V19, P376, DOI 10.1097/00004694-199905000-00017

Priftis KN, 2003, PEDIATR PULM, V35, P83, DOI 10.1002/ppul.10220

Ramirez N, 1997, J PEDIATR ORTHOPED, V17, P109, DOI 10.1097/00004694-199701000-00023

RIDEAU Y, 1984, MUSCLE NERVE, V7, P281, DOI 10.1002/mus.880070405

WINTER S, 1994, ORTHOP CLIN N AM, V25, P239

YOUNG A, 1984, DEV MED CHILD NEUROL, V26, P808

Zeller R, 2000, ORTHOPADE, V29, P518, DOI 10.1007/s001320050490

NR 18

TC 5

Z9 5

U1 5

U2 15

PU SAGE PUBLICATIONS LTD

PI LONDON

PA 1 OLIVERS YARD, 55 CITY ROAD, LONDON EC1Y 1SP, united kingdom

SN 0309-3646

EI 1746-1553

J9 PROSTHET ORTHOT INT

JI Prosthet. Orthot. Int.

PD DEC

PY 2004

VL 28

IS 3

BP 273

EP 277

PG 5

WC Orthopedics; Rehabilitation

WE Science Citation Index Expanded (SCI-EXPANDED)

SC Orthopedics; Rehabilitation

GA 883SX

UT WOS:000226037100010

PM 15658640

DA 2023-08-10

ER

PT J

AU Bennett, BC

Abel, MF

Granata, KP

AF Bennett, BC

Abel, MF

Granata, KP

TI Seated postural control in adolescents with idiopathic scoliosis

SO SPINE

LA English

DT Article

DE idiopathic scoliosis; sway; center of pressure; postural control

ID HEALTHY-INDIVIDUALS; LUMBAR SPINE; MUSCLE; BALANCE; STABILITY; MOBILITY;

PATTERN

AB Study Design. The center of pressure (COP) path in 14 adolescents with idiopathic scoliosis and 12 age-matched able-bodied adolescents was compared using traditional measures and a two-level decomposition.

Objectives. To investigate whether asymmetries in the spines of children with idiopathic scoliosis are reflected in altered sway patterns in quiet sitting.

Summary of Background Data. Previous studies have studied the sway of children with scoliosis while standing. However, the standing posture is typically controlled at the ankle joint. To date, there are no studies with this population of sitting sway, where the movement is controlled by the trunk muscles.

Methods. Traditional measures of the COP of the trunk were analyzed. The COP was also decomposed into an approximation of the center of mass path and deviations around this path.

Results. COP movement in sitting, reflecting the postural control of the spine, was decreased in adolescents with idiopathic scoliosis. Children with scoliosis had symmetric sitting COP trajectories and most measures were similar between the two groups.

Conclusions. The results suggest a control strategy for maintaining a sitting posture that does not change with the development of scoliosis but does adapt by decreasing movement to maintain the trunk in a region where the it can remain "passively" stable.

C1 Univ Virginia, Charlottesville, VA USA.

Virginia Tech, Blacksburg, VA USA.

C3 University of Virginia; Virginia Polytechnic Institute & State

University

RP Bennett, BC (通讯作者)，Kluge Childrens Rehab & Res Inst, 2270 Ivy Rd, Charlottesville, VA 22903 USA.

EM bcb3a@virginia.edu

RI Bennett, Bradford/GQA-5646-2022

CR BOUISSET S, 1994, NEUROREPORT, V5, P957, DOI 10.1097/00001756-199404000-00026

Chen PQ, 1998, CLIN BIOMECH, V13, pS52, DOI 10.1016/S0268-0033(97)00075-2

Cholewicki J, 1997, SPINE, V22, P2207, DOI 10.1097/00007632-199710010-00003

COLLINS JJ, 1993, EXP BRAIN RES, V95, P308, DOI 10.1007/BF00229788

ELLIPSE DM, 2002, MATLAB SOFTWARE WEB

FIDLER MW, 1976, J BONE JOINT SURG BR, V58, P200, DOI 10.1302/0301-620X.58B2.932082

Gauchard GC, 2001, SPINE, V26, P1052, DOI 10.1097/00007632-200105010-00014

Granata KP, 2001, CLIN BIOMECH, V16, P650, DOI 10.1016/S0268-0033(01)00064-X

GUAN J, 1999, J SPORT EXERC PSYC S, V21, P48

Hodges PW, 2002, EXP BRAIN RES, V144, P293, DOI 10.1007/s00221-002-1040-x

Kantor E, 2001, NEUROSCI LETT, V308, P128, DOI 10.1016/S0304-3940(01)01986-3

KENNELLY KP, 1993, SPINE, V18, P913, DOI 10.1097/00007632-199306000-00017

King DL, 1997, GAIT POSTURE, V6, P27, DOI 10.1016/S0966-6362(96)01101-0

KING HA, 1983, J BONE JOINT SURG AM, V65, P1302, DOI 10.2106/00004623-198365090-00012

Leong JCY, 1999, SPINE, V24, P1310, DOI 10.1097/00007632-199907010-00007

Mannion A F, 1998, Eur Spine J, V7, P289, DOI 10.1007/s005860050077

Meier MP, 1997, SPINE, V22, P2357, DOI 10.1097/00007632-199710150-00008

Nault ML, 2002, SPINE, V27, P1911, DOI 10.1097/00007632-200209010-00018

Radebold A, 2000, SPINE, V25, P947, DOI 10.1097/00007632-200004150-00009

Radebold A, 2001, SPINE, V26, P724, DOI 10.1097/00007632-200104010-00004

Silfies SP, 2003, HUM MOVEMENT SCI, V22, P237, DOI 10.1016/S0167-9457(03)00046-0

Winter DA, 1998, J NEUROPHYSIOL, V80, P1211, DOI 10.1152/jn.1998.80.3.1211

Winter DA, 1996, J NEUROPHYSIOL, V75, P2334, DOI 10.1152/jn.1996.75.6.2334

Zatsiorsky VM, 1998, J BIOMECH, V31, P161, DOI 10.1016/S0021-9290(97)00116-4

Zatsiorsky VM, 1999, MOTOR CONTROL, V3, P28, DOI 10.1123/mcj.3.1.28

Zatsiorsky VM, 2000, MOTOR CONTROL, V4, P185, DOI 10.1123/mcj.4.2.185

Zatsiorsky VM, 2002, J SPORT EXERCISE PSY, V24, P9

NR 27

TC 22

Z9 23

U1 0

U2 11

PU LIPPINCOTT WILLIAMS & WILKINS

PI PHILADELPHIA

PA TWO COMMERCE SQ, 2001 MARKET ST, PHILADELPHIA, PA 19103 USA

SN 0362-2436

EI 1528-1159

J9 SPINE

JI SPINE

PD OCT 15

PY 2004

VL 29

IS 20

BP E449

EP E454

DI 10.1097/01.brs.0000142005.21714.32

PG 6

WC Clinical Neurology; Orthopedics

WE Science Citation Index Expanded (SCI-EXPANDED)

SC Neurosciences & Neurology; Orthopedics

GA 862MM

UT WOS:000224495000030

PM 15480122

DA 2023-08-10

ER

PT J

AU Bullmann, V

Halm, HF

Lerner, T

Lepsien, U

Hackenberg, L

Liljenqvist, U

AF Bullmann, V

Halm, HF

Lerner, T

Lepsien, U

Hackenberg, L

Liljenqvist, U

TI Prospective evaluation of brace treatment in idiopathic scoliosis

SO ZEITSCHRIFT FUR ORTHOPADIE UND IHRE GRENZGEBIETE

LA German

DT Article

DE idiopathic scoliosis; brace treatment; flexibility; skeletal age

ID NATURAL-HISTORY; CONSERVATIVE TREATMENT; SPINAL DEFORMITIES; MILWAUKEE

BRACE; BOSTON BRACE; LARGE CURVES; GIRLS; ORTHOSIS; THERAPY

AB Aim: To prospectively evaluate the results of brace treatment in idiopathic scoliosis and to define risk factors of treatment failure. Method: Fifty-two patients with a Cobb angle of between 25 and 40degrees were included in the study. Prior to initiation of brace treatment with the Cheneau-Toulouse-Muenster orthesis, skeletal age and flexibility of the curve (bending films) were evaluated. The average follow-up after weaning of the brace was 42 months (36-78 months). Results: An average initial Cobb angle of 31degrees was corrected to 18degrees (43%) under brace treatment with a flexibility to 6degrees Cobb angle on bending films. Three years after weaning there was an overall increase of the Cobb angle to 37degrees on average. The apical vertebral rotation was corrected from 16degrees to 11degrees (31%) and increased to 20degrees during follow-up. Thoracic kyphosis changed from 24degrees to 18degrees during treatment. At the latest follow-up kyphosis had returned to the pre-treatment angle again. Twenty-two patients had a curve progression during or after brace treatment of more than 5degrees. In 14 patients surgical correction and fusion have been indicated. There was a positive correlation between flexibility and Cobb angle correction during brace treatment and a negative correlation between Cobb angle correction during brace treatment and curve progression ($p < 0.05$). Conclusions: Curve progression was prevented in 58%. Prognostic risk factors are a young age at initiation of brace treatment, a thoracic curve, unsatisfactory curve correction in the brace and a male gender.

C1 Univ Klinikum Munster, Klin & Poliklin Allgemeine Orthopad, D-48149 Munster, Germany.

C3 University of Munster

RP Bullmann, V (通讯作者)， Univ Klinikum Munster, Klin & Poliklin Allgemeine Orthopad, Albert Schweitzer Str 33, D-48149 Munster, Germany.

EM bullmanv@uni-muenster.de

CR [Anonymous], 1971, RADIOGRAPHIC ATLAS S

Boni T, 2002, ORTHOPADE, V31, P11, DOI 10.1007/s132-002-8270-4

BUNNELL WP, 1986, SPINE, V11, P773, DOI 10.1097/00007632-198610000-00003

CHENEAU J, 1986, ORTHOPADIE TECHNIK, V8, P443

Dickson RA, 1999, J BONE JOINT SURG BR, V81B, P193, DOI 10.1302/0301-620X.81B2.9630

DICKSON RA, 1985, J BONE JOINT SURG BR, V67, P176, DOI 10.1302/0301-620X.67B2.3872301

Frobin W, 1983, J Biol Photogr, V51, P11

GOLDBERG CJ, 1993, SPINE, V18, P902, DOI 10.1097/00007632-199306000-00015

Howard A, 1998, SPINE, V23, P2404, DOI 10.1097/00007632-199811150-00009

Karol LA, 2001, SPINE, V26, P2001, DOI 10.1097/00007632-200109150-00013

Katz DE, 2001, SPINE, V26, P2354, DOI 10.1097/00007632-200111010-00012

Korovessis P, 2000, SPINE, V25, P2064, DOI 10.1097/00007632-200008150-00010

Liljenqvist U, 1998, Z ORTHOP GRENZGEB, V136, P57, DOI 10.1055/s-2008-1044652

LONSTEIN JE, 1984, J BONE JOINT SURG AM, V66A, P1061, DOI 10.2106/00004623-198466070-00013

LONSTEIN JE, 1994, J BONE JOINT SURG AM, V76A, P1207, DOI 10.2106/00004623-199408000-00011

Matussek J, 2000, ORTHOPADE, V29, P490, DOI 10.1007/s001320050487

MILLER JAA, 1984, SPINE, V9, P632, DOI 10.1097/00007632-198409000-00015

NACHEMSON AL, 1995, J BONE JOINT SURG AM, V77A, P815, DOI 10.2106/00004623-199506000-00001

Nicholson GP, 2003, SPINE, V28, P2243, DOI 10.1097/01.BRS.0000085098.69522.52

Noonan KJ, 1996, J BONE JOINT SURG AM, V78A, P557, DOI 10.2106/00004623-199604000-00009

PICAULT C, 1986, SPINE, V11, P777, DOI 10.1097/00007632-198610000-00004

Schmitz A, 2002, Z ORTHOP GRENZGEB, V140, P347, DOI 10.1055/s-2002-32480

van Rhijn LW, 2002, ACTA ORTHOP SCAND, V73, P277

Vandal S, 1999, Issues Compr Pediatr Nurs, V22, P59

VONDEIMLING U, 1995, Z ORTHOP GRENZGEB, V133, P270, DOI 10.1055/s-2008-1039447

WEINSTEIN SL, 1986, SPINE, V11, P780, DOI 10.1097/00007632-198610000-00006

Weiss HR, 2003, ORTHOPADE, V32, P146, DOI 10.1007/s00132-002-0430-x

Wiley JW, 2000, SPINE, V25, P2326, DOI 10.1097/00007632-200009150-00010

WILLERS U, 1993, SPINE, V18, P432

Winter RB, 1997, SPINE, V22, P1283, DOI 10.1097/00007632-199706150-00001

WINTER RB, 1994, ORTHOP CLIN N AM, V25, P195

NR 31

TC 22

Z9 23

U1 0

U2 3

PU GEORG THIEME VERLAG KG

PI STUTTGART

PA RUDIGERSTR 14, D-70469 STUTTGART, GERMANY

SN 0044-3220

J9 Z ORTHOP GRENZGEB

JI Z. Orthop. Grenzg.

PD JUL-AUG

PY 2004

VL 142

IS 4

BP 403

EP 409

DI 10.1055/s-2004-822843

PG 7

WC Orthopedics

WE Science Citation Index Expanded (SCI-EXPANDED)

SC Orthopedics

GA 854ZM

UT WOS:000223942600003

PM 15346300

DA 2023-08-10

ER

PT J

AU Weiss, HR

AF Weiss, HR

TI Conservative treatment of idiopathic scoliosis with physical therapy and

orthoses

SO ORTHOPADE

LA German

DT Article

DE scoliosis; conservative treatment; physiotherapy; rehabilitation;

bracing

ID FOLLOW-UP; ADULT SCOLIOSIS; BRACE

AB Opinions differ in the international literature about the efficacy of conservative approaches to scoliosis treatment. Because this divergence of opinion corresponds to a great discrepancy in the standards applied to conservative treatment methods, it is not astonishing that the results of conservative treatment as described in the literature also differ. Scoliosis normally does not have such dramatic effects that immediate surgery would be indicated. Moreover, it is clear from the published literature that it is the functional and physiological impairments of scoliosis patients-including pain, torso deformity, psychological disturbance, and pulmonary dysfunction - which require therapeutic intervention.

In Germany the triad of outpatient physiotherapy, intensive inpatient rehabilitation, and bracing has proven effective in conservative scoliosis treatment. Indication, content, and results of the individual treatment procedures are described and discussed.

The positive outcomes of this practice validate a policy of offering conservative scoliosis treatment as an alternative to patients, including those for whom surgery is indicated.

C1 Asklepios Katharina Schroth Klin, D-55566 Bad Sobernheim, Germany.

RP Weiss, HR (通讯作者)， Asklepios Katharina Schroth Klin, Korczakstr 2, D-55566 Bad Sobernheim, Germany.

CR [Anonymous], P 11 INT C WORLD CON

APPELGREN G, 1990, SPINE, V15, P71, DOI 10.1097/00007632-199002000-00004

BAUER R, 2001, PRAXIS ORTHOPADIE, V1, P498

BERGER M, 2001, DTSCH ARZTEBLATT, V6, P294

BETTANY J, 1995, ST HEAL T, V15, P321

BETTANY J, 1994, P EUR SPIN DEF SOC M

BLOUNT W, 1980, MILWAUKEE BRACE, P1

Bradford DS, 1999, SPINE, V24, P2617, DOI 10.1097/00007632-199912150-00009

COCHRAN T, 1983, SPINE, V8, P576, DOI 10.1097/00007632-198309000-00003

CONNOLLY PJ, 1995, J BONE JOINT SURG AM, V77A, P1210, DOI 10.2106/00004623-199508000-00011

Danielsson AJ, 2001, SPINE, V26, P516

Danielsson AJ, 2001, EUR SPINE J, V10, P278, DOI 10.1007/s005860100309

Dickson RA, 1999, SPINE, V24, P2601, DOI 10.1097/00007632-199912150-00007

EMANS JB, 1986, SPINE, V11, P792, DOI 10.1097/00007632-198610000-00009

Ferraro C., 1998, EUR MEDICOPHYS, V34, P25

FREIDEL K, 1999, THESIS U BREMEN

FREIDEL K, 1999, STATIONARE INTENSIVR

Goldberg CJ, 2001, SPINE, V26, P42, DOI 10.1097/00007632-200101010-00009

GOLDBERG CJ, 1993, SPINE, V18, P902, DOI 10.1097/00007632-199306000-00015

GOLDBERG MS, 1988, SPINE, V13, P1371, DOI 10.1097/00007632-198812000-00008

GRILL F, 1997, DTSCH OST ORTH 17 BI

HANKE P, 1983, SKOL ENTW GRUNDL ANL

HANKS GA, 1988, SPINE, V13, P626

HEFTI F, 1997, KINDERORTHOPADIE PRA, P80

HEINE J, 1985, Z ORTHOP GRENZGEB, V123, P323, DOI 10.1055/s-2008-1045158

HOFFA A, 2002, LEHRBUCH ORTHOPADISC

HOPF C, 1985, Z ORTHOP GRENZGEB, V123, P312, DOI 10.1055/s-2008-1045157

Hopf C, 2000, ORTHOPADE, V29, P500, DOI 10.1007/s001320050488

Howard A, 1998, SPINE, V23, P2404, DOI 10.1097/00007632-199811150-00009

KLAPP R, 1905, FUNKTIONELLE BEHANDL

KLISIC P, 1991, WIRBELSAULENDEFORMIT, V1, P1

Landauer F, 1999, FORTBILDUNG ORTHOPAD, P31

LEHNERTSCHROTH C, 1981, Z ORTHOP PRAX, V27, P255

LEHNERTSCHROTH CH, 2000, DREIDIMENSIONALE SKO

LOVETT RW, 1907, LATERAL CURVATURE SP

Matussek J, 2000, ORTHOPADE, V29, P490, DOI 10.1007/s001320050487

McCance SE, 1998, SPINE, V23, P2063, DOI 10.1097/00007632-199810010-00005

MELLEROWICZ H, 1994, VORTR 42 JAHR VER SU

Moen KY, 1999, SPINE, V24, P2570, DOI 10.1097/00007632-199912150-00003

Mollon G, 1986, KINESITHERAPIE SCI, V244, P47

NACHEMSON A, 1979, SPINE, V4, P513, DOI 10.1097/00007632-197911000-00011

NACHEMSON AL, 1995, J BONE JOINT SURG AM, V77A, P815, DOI 10.2106/00004623-199506000-00001

NOKER M, 1998, LEHRBUCH KLIN KINDER, P517

OLAFSSON Y, 1995, J PEDIATR ORTHOPED, V15, P524, DOI 10.1097/01241398-199507000-00023

OLDEVIG J, 1910, NEUES GERAT NEUE UBU

Pehrsson K, 2001, THORAX, V56, P388, DOI 10.1136/thorax.56.5.388

PETERMANN F, 1996, PRAV REHEHAB, V4, P140

Rigo M, 1999, ST HEAL T, V59, P362

Rowe DE, 1997, J BONE JOINT SURG AM, V79A, P664, DOI 10.2106/00004623-199705000-00005

SCHANZ A, 2001, STAT BELASTUNGSDEFOR

SCHARLL M, 1958, Z KRANKENGYM, V12, P193

SCHONING N, 2001, VORTR 8 BAD SOB SKOL

SCHROTH K, 1924, ATMUNGSKUR

Shandsjr AR, 1941, J BONE JOINT SURG AM, V23, P963

THULBOURNE T, 1976, J BONE JOINT SURG BR, V58, P64, DOI 10.1302/0301-620X.58B1.1270497

Vojta V., 1992, VOJTA PRINZIP

VONNIEDERHOFFER L, 1942, BEHANDLUNG RUCKGRATV

Weber M, 1999, Z ORTHOP GRENZGEB, V137, P173, DOI 10.1055/s-2008-1039354

Weiss H R, 1997, Pediatr Rehabil, V1, P35

WEISS HR, 1995, Z ORTHOP GRENZGEB, V133, P114, DOI 10.1055/s-2008-1039421

WEISS HR, 2001, BEFUNDGERECHTE PHYSI

WEISS HR, 2002, P INT RES SOC SPIN D, P84

WEISS HR, 2000, PRAXIS CHENEAU KORSE

WEISS HR, 2001, WIRBELSAULE, P56

Weiss HR, 1994, WIRBELSAULENDEFORMIT, V3, P47

WEISS HR, 1995, MED ORTHOP TECH, V115, P323

WEISS HR, 2002, P INT RES SOC SPIN D, P86

WEISS HR, 2000, MED ORTHOP TECHN, V120, P106

WEISS HR, 2000, SKOLIOSEREHABILITATI

WEISS M, 1991, ADV METHODS PHARMACO, V1, P89

[No title captured]

NR 71

TC 9

Z9 9

U1 0

U2 14

PU SPRINGER

PI NEW YORK

PA ONE NEW YORK PLAZA, SUITE 4600, NEW YORK, NY, UNITED STATES

SN 0085-4530

EI 1433-0431

J9 ORTHOPADE

JI Orthopade

PD FEB

PY 2003

VL 32

IS 2

BP 146

EP +

DI 10.1007/s00132-002-0430-x

PG 11

WC Orthopedics

WE Science Citation Index Expanded (SCI-EXPANDED)

SC Orthopedics

GA 662JY

UT WOS:000181944600007

PM 12607080

DA 2023-08-10

ER

PT J

AU Lenke, LG

White, DK

Kemp, JS

Bridwell, KH

Blanke, KM

Engsberg, JR

AF Lenke, LG

White, DK

Kemp, JS

Bridwell, KH

Blanke, KM

Engsberg, JR

TI Evaluation of ventilatory efficiency during exercise in patients with

idiopathic scoliosis undergoing spinal fusion

SO SPINE

LA English

DT Article; Proceedings Paper

CT 36th Annual Meeting of the Scoliosis-Research-Society

CY SEP 19-22, 2001

CL CLEVELAND, OH

SP Scoliosis Res Soc

DE pulmonary function; ventilatory efficiency; adolescent idiopathic

scoliosis; maximal oxygen uptake; spinal fusion

ID PULMONARY-FUNCTION; THORACIC SCOLIOSIS; ADOLESCENT; INSTRUMENTATION;

METAANALYSIS; SURGERY; BRACE

AB Study Design. A prospective evaluation of ventilatory function following spinal fusion in adolescent idiopathic scoliosis.

Objectives. To prospectively evaluate pulmonary function, maximal oxygen uptake, and ventilatory efficiency during exercise in patients with adolescent idiopathic scoliosis before surgery and a minimum of 2 years postoperation.

Summary of Background Data. For reasons that are unclear, patients with untreated adolescent idiopathic scoliosis tend to avoid aerobic exercise. Their reluctance may be the result of low ventilatory efficiency, as they often approach their ventilatory ceiling at maximum oxygen uptake despite forced vital capacities that are near normal. This inefficiency of ventilation with exercise may explain the reluctance of patients with scoliosis to pursue aerobic fitness. No study has evaluated the effect spinal fusion has on the ventilatory function of patients with scoliosis during exercises.

Methods. Forty-two patients with adolescent idiopathic scoliosis (36 female and 6 male) at an average age of 14 +/- 3 years (range 10-18 years) underwent spinal fusion. Twenty patients underwent a posterior spinal fusion alone, 20 an anterior spinal fusion alone, and 2 an anterior spinal fusion and posterior spinal fusion. The average Cobb measurement was 55degrees (range 40-85degrees). Pulmonary function values (forced vital capacity, total lung capacity, maximum voluntary ventilation), maximum oxygen uptake ((V) over dot O(2)max), and ventilatory efficiency were obtained before surgery and a minimum of 2 years postoperation.

Results. For all patients, forced vital capacity percent predicted decreased from 88.1% to 81.4% (P<0.0001). Total lung capacity also declined from 90.5% to 88.5% but was not statistically significant (P=0.189). Percent predicted maximum oxygen uptake ((V) over dot O(2)max) declined from 93.6% to 85.1% (P = 0.00029). Ventilatory efficiency, as measured by V(E)max/maximum voluntary ventilation, improved from 0.76 to 0.68 (P = 0.005), whereas measured by V(E)max/FEV1 x 40 was unchanged from 0.69 to 0.70 (P = 0.172) postoperation. The choice of operative approach [anterior (n = 20) versus posterior (n = 20)] or whether rib graft was harvested (n = 33) versus iliac crest graft (n = 7) did not change these results.

Conclusion. Improvement in ventilatory efficiency during exercise dies no occur in the majority of patients with adolescent idiopathic scoliosis following spinal fusion and thus cannot be relied on to foster increases in aerobic activity.

C1 Washington Univ, Sch Med, Dept Orthopaed Surg, St Louis, MO 63110 USA.

St Louis Childrens Hosp, Pulm Funct Lab, St Louis, MO 63178 USA.

St Louis Univ, Dept Pediat Pulm Med, St Louis, MO 63103 USA.

Barnes Jewish Hosp, Human Performance Lab, St Louis, MO 63110 USA.

C3 Washington University (WUSTL); St. Louis Children's Hospital; Washington

University (WUSTL); Saint Louis University; Barnes-Jewish Hospital;

Washington University (WUSTL)

RP Lenke, LG (通讯作者)， Washington Univ, Sch Med, Dept Orthopaed Surg, 1 Barnes Jewish Hosp Plaza,Suite 11300, St Louis, MO 63110 USA.

EM lenkel@msnotes.wustl.edu

CR Athanasopoulos S, 1999, SCAND J MED SCI SPOR, V9, P36

Giordano A, 1997, NUCL MED COMMUN, V18, P105, DOI 10.1097/00006231-199702000-00003

Graham EJ, 2000, SPINE, V25, P2319, DOI 10.1097/00007632-200009150-00009

HAHER TR, 1995, SPINE, V20, P1575, DOI 10.1097/00007632-199507150-00005

KEARON C, 1993, AM REV RESPIR DIS, V148, P295, DOI 10.1164/ajrccm/148.2.295

KEARON C, 1993, AM REV RESPIR DIS, V148, P288, DOI 10.1164/ajrccm/148.2.288

KINNEAR WJM, 1993, SPINE, V18, P1556

Korovessis P, 1996, SPINE, V21, P1979, DOI 10.1097/00007632-199609010-00008

LENKE LG, 1995, SPINE, V20, P1343, DOI 10.1097/00007632-199506000-00004

LENKE LG, 1992, J SPINAL DISORD, V5, P16, DOI 10.1097/00002517-199203000-00003

Pehrsson K, 2001, THORAX, V56, P388, DOI 10.1136/thorax.56.5.388

Upadhyay S S, 1995, Eur Spine J, V4, P274, DOI 10.1007/BF00301033

UPADHYAY SS, 1995, SPINE, V20, P2415, DOI 10.1097/00007632-199511001-00008

Vedantam R, 1997, SPINE, V22, P2731, DOI 10.1097/00007632-199712010-00006

Vedantam R, 2000, SPINE, V25, P82, DOI 10.1097/00007632-200001010-00015

Wong CA, 1996, THORAX, V51, P534, DOI 10.1136/thx.51.5.534

Wood KB, 1996, SPINE, V21, P718, DOI 10.1097/00007632-199603150-00012

NR 17

TC 34

Z9 38

U1 0

U2 4

PU LIPPINCOTT WILLIAMS & WILKINS

PI PHILADELPHIA

PA TWO COMMERCE SQ, 2001 MARKET ST, PHILADELPHIA, PA 19103 USA

SN 0362-2436

EI 1528-1159

J9 SPINE

JI SPINE

PD SEP 15

PY 2002

VL 27

IS 18

BP 2041

EP 2045

DI 10.1097/00007632-200209150-00014

PG 5

WC Clinical Neurology; Orthopedics

WE Science Citation Index Expanded (SCI-EXPANDED); Conference Proceedings Citation Index - Science (CPCI-S)

SC Neurosciences & Neurology; Orthopedics

GA 594ZJ

UT WOS:000178082100013

PM 12634566

DA 2023-08-10

ER

PT J

AU Parsch, D

Gartner, V

Brocai, DRC

Carstens, C

Schmitt, H

AF Parsch, D

Gartner, V

Brocai, DRC

Carstens, C

Schmitt, H

TI Sports activity of patients with idiopathic scoliosis at long-term

follow-up

SO CLINICAL JOURNAL OF SPORT MEDICINE

LA English

DT Article

DE idiopathic scoliosis; sports activity, long-term; spinal fusion

ID ADOLESCENT SCOLIOSIS; FUSION

AB Objective: The aim of the study was to assess long-term the sports activities of operatively and nonoperatively treated patients with idiopathic scoliosis and compare these activities with those of controls.

Study Design: Cross-sectional case-control study, performed at The Orthopaedic University Hospital Heidelberg.

Patients and Methods: The study enrolled 59 patients (53 female, 6 male; mean age 43 years) with idiopathic scoliosis and a minimum follow-up of 5 years (mean 22 years) since treatment (28 nonoperative, 31 operative). Mean Cobb angle at the time of the study was 54degrees. An age-adjusted control group ($n = 33$) with no history of spinal disorder was evaluated at the same time. All participants in the study ($n = 92$) completed a questionnaire assessing spinal function (Spine Score) and sporting activity (Sport Score). In addition, the scoliosis patients underwent radiographic evaluation of their spine. The groups were compared by analysis of variance. In order to assess the relationship between two variables, Spearman's correlation coefficient was calculated.

Results: Both groups of scoliosis patients attained a lower Sport Score than the controls ($p < 0.015$ and $p < 0.006$, respectively). There was no difference between the two scoliosis groups. Reduced spinal function correlated with reduced sports activity ($p < 0.001$). In both scoliosis groups, the subscales "back pain" and "physical activity" correlated with sporting activity ($p < 0.03$ and $p < 0.02$, respectively), In the surgically treated patients, Cobb angle correlated with reduced sports activity ($p < 0.03$). The extent of the spinal arthrodesis (number of segments) in surgically treated patients had no effect on their sports activity.

Conclusions: Over the long term, patients with idiopathic scoliosis suffer impairment of their sports activities compared with age-matched controls. The main reasons for this are functional impairment and the frequency of back pain. Sports activity is not more restricted after extended spinal fusion than it is after nonoperative treatment.

C1 Orthopaed Univ Hosp Heidelberg, D-69118 Heidelberg, Germany.

C3 Ruprecht Karls University Heidelberg

RP Parsch, D (通讯作者)， Orthopaed Univ Hosp Heidelberg, Schlierbacher Landstr 200, D-69118 Heidelberg, Germany.

OI Schmitt, Holger/0000-0001-5765-7036

CR Barber-Westin SD, 1999, SPORTS MED, V28, P1, DOI 10.2165/00007256-199928010-00001

Bridwell KH, 2000, SPINE, V25, P2392, DOI 10.1097/00007632-200009150-00020

Bridwell KH, 1999, SPINE, V24, P2607, DOI 10.1097/00007632-199912150-00008

COCHRAN T, 1983, SPINE, V8, P576, DOI 10.1097/00007632-198309000-00003

CONNOLLY PJ, 1995, J BONE JOINT SURG AM, V77A, P1210, DOI 10.2106/00004623-199508000-00011

DICKSON JH, 1990, J BONE JOINT SURG AM, V72A, P678, DOI 10.2106/00004623-199072050-00006

Dickson RA, 1999, SPINE, V24, P2601, DOI 10.1097/00007632-199912150-00007

HOPF C, 1991, Z ORTHOP GRENZGEB, V129, P204, DOI 10.1055/s-2008-1040184

MOSKOWITZ A, 1980, J BONE JOINT SURG AM, V62, P364, DOI 10.2106/00004623-198062030-00006

NOYES FR, 1989, CLIN ORTHOP RELAT R, P238

Parsch D, 2001, J BONE JOINT SURG BR, V83B, P1133, DOI 10.1302/0301-620X.83B8.12079

SENGHAS RE, 1992, J BONE JOINT SURG AM, V74A, P319, DOI 10.2106/00004623-199274030-00001

White SF, 1999, SPINE, V24, P1693, DOI 10.1097/00007632-199908150-00011

NR 13

TC 27

Z9 29

U1 0

U2 8

PU LIPPINCOTT WILLIAMS & WILKINS

PI PHILADELPHIA

PA 530 WALNUT ST, PHILADELPHIA, PA 19106-3621 USA

SN 1050-642X

J9 CLIN J SPORT MED

JI Clin. J. Sport Med.

PD MAR

PY 2002

VL 12

IS 2

BP 95

EP 98

DI 10.1097/00042752-200203000-00005

PG 4

WC Orthopedics; Physiology; Sport Sciences

WE Science Citation Index Expanded (SCI-EXPANDED)

SC Orthopedics; Physiology; Sport Sciences

GA 540WM

UT WOS:000174953900004

PM 11953555

DA 2023-08-10

ER

PT J

AU Schmitt, H

Carstens, C

AF Schmitt, H

Carstens, C

TI Physical limits of deformities of the spine in children

SO DEUTSCHE ZEITSCHRIFT FUR SPORTMEDIZIN

LA German

DT Review

DE sports; scoliosis; kyphosis; spondylolysis

ID NATURAL-HISTORY; DEGENERATIVE SPONDYLOLISTHESIS; LUMBAR SPONDYLOLYSIS;

FOLLOW-UP; SCOLIOSIS; PROGRESSION; ADOLESCENTS; INJURIES; ETIOLOGY;

DEFECTS

AB Spinal deformities can reduce physical resilience. Recommendations are made with regard to the degree of deformity and the expected growth. Children with scoliosis more than 20degrees (Cobb) should take part in sports, because no studies exist which show that special disciplines increase the deformity of the spine. Recommendations after spinal surgery are made individually with regard to the length of fusions. Physical activity seems to influence the degree of kyphosis. Children with M. Scheuermann and acute backache shouldn't take part in sports, in adulthood recommendations are made with regard to the degree of deformity and the resilience of the anterior parts of the spine. Children with spondylolysis and acute backache are not able to do sports, clinical and radiological checks are required. Children with spondylolysis or spondylolisthesis shouldn't take part in sports with hyperextensive or rotatory loads. There exist only a few scientific examinations about sports and spinal deformities, recommendations essentially base on empirical data.

C1 Heidelberg Univ, Stiftung Orthopad Klin, Heidelberg, Germany.

C3 4EU+; Ruprecht Karls University Heidelberg

RP Schmitt, H (通讯作者)，Heidelberg Univ, Stiftung Orthopad Klin, Heidelberg, Germany.

EM holger.schmitt@ok.uni-heidelberg.de

OI Schmitt, Holger/0000-0001-5765-7036

CR AMATO M, 1984, RADIOLOGY, V153, P627, DOI 10.1148/radiology.153.3.6494460

Berlemann U, 1999, J SPINAL DISORD, V12, P68

BRADFORD D, 1985, PEDIAT SPINE

BUNNELL WP, 1988, CLIN ORTHOP RELAT R, V229, P20

Cinotti G, 1997, INT ORTHOP, V21, P337, DOI 10.1007/s002640050180

CYRON BM, 1978, J BONE JOINT SURG BR, V60, P234, DOI 10.1302/0301-620X.60B2.659472

Engelhardt M, 1997, ORTHOPADE, V26, P755, DOI 10.1007/PL00003437

FREDRICKSON BE, 1984, J BONE JOINT SURG AM, V66A, P699, DOI 10.2106/00004623-198466050-00008

GOLDSTEIN JD, 1991, AM J SPORT MED, V19, P463, DOI 10.1177/036354659101900507

Graf R, 1998, ORTHOPADE, V27, P51, DOI 10.1007/s001320050202

GREEN TP, 1994, SPINE, V19, P2683, DOI 10.1097/00007632-199412010-00016

HEFTI F, 1994, ORTHOPADE, V23, P220

HENSINGER RN, 1989, J BONE JOINT SURG AM, V71A, P1098, DOI 10.2106/00004623-198971070-00020

HOPF C, 1991, Z ORTHOP GRENZGEB, V129, P204, DOI 10.1055/s-2008-1040184

HOPF C, 1989, ROFO FORTSCHR RONTG, V151, P311, DOI 10.1055/s-2008-1047184

JACKSON DW, 1976, CLIN ORTHOP RELAT R, P68

Konermann W, 1992, Sportverletz Sportschaden, V6, P156, DOI 10.1055/s-2007-993543

Kotani PT., 1971, BRIT J SPORT MED, V6, P4

LAFOND G, 1962, Clin Orthop, V22, P175

LONSTEIN JE, 1984, J BONE JOINT SURG AM, V66A, P1061, DOI 10.2106/00004623-198466070-00013

MURRAY PM, 1993, J BONE JOINT SURG AM, V75A, P236, DOI 10.2106/00004623-199302000-00011

Muschik M, 1996, J PEDIATR ORTHOPED, V16, P364, DOI 10.1097/01241398-199605000-00014

Niethard FU, 1997, ORTHOPADE, V26, P750, DOI 10.1007/PL00003436

NIETHARD FU, 1997, KINDERORTHOPADIE, P180

OMEY ML, 2000, CLIN ORTHOP RELAT R, V372, P74

ONEILL DB, 1989, AM J SPORTS MED, V17, P196

PARSCH D, IN PRESS CLIN J SPOR

PFEIL J, 1987, Z ORTHOP GRENZGEB, V125, P526, DOI 10.1055/s-2008-1044750

PFEIL J, 1985, ORTHOP PRAX, V21, P214

POLLAEHNE W, 1991, Deutsche Zeitschrift fuer Sportmedizin, V42, P292

PORTER RW, 1984, SPINE, V9, P755, DOI 10.1097/00007632-198410000-00018

Recknagel S, 1996, Z ORTHOP GRENZGEB, V134, P214, DOI 10.1055/s-2008-1039751

RIEL K-A, 1991, Deutsche Zeitschrift fuer Sportmedizin, V42, P14

ROGALA EJ, 1978, J BONE JOINT SURG AM, V60, P173, DOI 10.2106/00004623-197860020-00005

ROMPE G, 1972, Z ORTHOP GRENZGEB, V110, P745

ROSSI F, 1990, J SPORT MED PHYS FIT, V30, P450

SARASTE H, 1987, J PEDIATR ORTHOPED, V7, P631

SARASTE H, 1984, INT ORTHOP, V8, P163, DOI 10.1007/BF00269912

Schlenzka D, 1997, ORTHOPADE, V26, P760, DOI 10.1007/PL00003438

SCHLOZ M, 1993, ORTHOP PRAX, V8, P544

Schmitt H, 2001, J BONE JOINT SURG BR, V83B, P324, DOI 10.1302/0301-620X.83B3.11386

SEITSALO S, 1991, SPINE, V16, P417, DOI 10.1097/00007632-199104000-00004

SEMON RL, 1981, SPINE, V6, P172, DOI 10.1097/00007632-198103000-00012

Soler T, 2000, AM J SPORT MED, V28, P57, DOI 10.1177/03635465000280012101

SPENCER GW, 1983, CLIN SPORTS MED, V2, P191

SWARD L, 1992, SPORTS MED, V13, P357

SWARD L, 1993, AM J SPORT MED, V21, P841, DOI 10.1177/036354659302100614

THEISS F, 1980, DTSCH Z SPORTMED, V31, P161

TRUOUP JDG, 1976, CLIN ORTHOP RELAT R, V147, P59

vanTulder MW, 1997, SPINE, V22, P427, DOI 10.1097/00007632-199702150-00015

WARREN MP, 1986, NEW ENGL J MED, V314, P1348, DOI 10.1056/NEJM198605223142104

Watson AWS, 1995, J SPORT MED PHYS FIT, V35, P289

WEINSTEIN SL, 1986, SPINE, V11, P780, DOI 10.1097/00007632-198610000-00006

WILTSE LL, 1975, J BONE JOINT SURG AM, VA 57, P17, DOI 10.2106/00004623-197557010-00003

WILTSE LL, 1976, CLIN ORTHOPAEDICS, V35, P116

Wismach J, 1988, Sportverletz Sportschaden, V2, P95, DOI 10.1055/s-2007-993672

Wojtys EM, 2000, AM J SPORT MED, V28, P490, DOI 10.1177/03635465000280040801

YANCEY RA, 1994, THORACOLUMBAR SPINE, P162

NR 58

TC 11

Z9 11

U1 0

U2 0

PU W W F VERLAGSGESELLSCHAFT GMBH

PI GREVEN

PA AM EGGENKAMP 37-39, 48268 GREVEN, GERMANY

SN 0344-5925

J9 DEUT Z SPORTMED

JI Dtsch. Z. Sportmed.

PD JAN

PY 2002

VL 53

IS 1

BP 6

EP 11

PG 6

WC Sport Sciences

WE Science Citation Index Expanded (SCI-EXPANDED)

SC Sport Sciences

GA 530EX

UT WOS:000174344400001

DA 2023-08-10

ER

PT J

AU Wong, MS

Mak, AFT

Luk, KDK

Evans, JH

Brown, B

AF Wong, MS

Mak, AFT

Luk, KDK

Evans, JH

Brown, B

TI Effectiveness of audio-biofeedback in postural training for adolescent

idiopathic scoliosis patients

SO PROSTHETICS AND ORTHOTICS INTERNATIONAL

LA English

DT Article; Proceedings Paper

CT 10th Triennial World Congress of the

International-Society-for-Prosthetics-and-Orthotics

CY JUL 01-06, 2001

CL GLASGOW, SCOTLAND

SP Int Soc Prosthet & Orthot

ID MILWAUKEE BRACE

AB The possibility of using learned physiological responses in control of progressive adolescent idiopathic scoliosis (AIS) was investigated, Sixteen (16) AIS patients with progressing or high-risk curves (Cobb's angle between 25 degrees and 35 degrees at start and reducible by lateral bending) were fitted with a device with tone alarm for poor posture. In the first Is months of application, 3 patients defaulted and 4 showed curve progression >10 degrees (2 changed to rigid spinal orthoses and 2 underwent surgery). The curves for the other 9 patients were kept under control (within +/-5 degrees of Cobb's angle) and 5 of them have reached skeletal maturity and terminated the application, The remaining 4 patients were still using the devices until skeletal maturity or curve progression, The

curve control rate was 69%. A long-lasting active spinal control could be achieved through the patient's own spinal muscles, Nevertheless, before the postural training device could become treatment modality, a long-term study for more AIS patients was necessary. This project is ongoing in the Duchess of Kent Children's Hospital. Sandy Bay, Hong Kong.

C1 Hong Kong Polytech Univ, Rehabil Engn Ctr, Kowloon, Hong Kong, china.

C3 Hong Kong Polytechnic University

RP Wong, MS (通讯作者)， Hong Kong Polytech Univ, Rehabil Engn Ctr, Kowloon, Hong Kong, china.

EM rcmswong@polyu.edu.hk

RI Evans, J. Henry/E-5778-2011

OI WONG, MS/0000-0002-4157-9528

CR [Anonymous], 1971, RADIOGRAPHIC ATLAS S

[Anonymous], 1990, PEDIAT ORTHOPEDICS

AZRIN N, 1968, J APPL BEHAV ANAL, V1, P99, DOI 10.1901/jaba.1968.1-99

BERGER N, 1983, SPINAL ORTHOTICS PRO

BERTRAND SL, 1992, CLIN ORTHOP RELAT R, P176

BLOUNT WP, 1957, J BONE JOINT SURG AM, V39, P693

BUNNELL WP, 1984, J BONE JOINT SURG AM, V66A, P1381, DOI 10.2106/00004623-198466090-00010

CARR WA, 1980, J BONE JOINT SURG AM, V62, P599, DOI 10.2106/00004623-198062040-00015

Cobb JR., 1948, INSTR COURSE LECT, V5, P261

DANSEREAU J, 1995, ORTHOP T, V19, P591

DURHAM JW, 1990, SPINE, V15, P888, DOI 10.1097/00007632-199009000-00010

DWORKIN B, 1985, P NATL ACAD SCI USA, V82, P2493, DOI 10.1073/pnas.82.8.2493

Dworkin B. R., 1982, HEALTH PSYCHOL, V1, P45, DOI [10.1037/0278-6133.1.1.45, DOI 10.1037/0278-6133.1.1.45]

EDMONSON AS, 1977, CLIN ORTHOP RELAT R, V126, P58

FALLSTROM K, 1984, ORTHOP T, V8, P150

KEISER RP, 1976, CLIN ORTHOP RELAT R, P19

LONSTEIN JE, 1994, J BONE JOINT SURG AM, V76A, P1207, DOI 10.2106/00004623-199408000-00011

MYERS BA, 1970, AM J DIS CHILD, V120, P175, DOI 10.1001/archpedi.1970.02100080059001

PERDRIOLLE R, 1985, SPINE, V10, P785, DOI 10.1097/00007632-198511000-00001

RISSER J C, 1958, Clin Orthop, V11, P111

ROGALA EJ, 1978, J BONE JOINT SURG AM, V60, P173, DOI 10.2106/00004623-197860020-00005

RUDICEL S, 1983, SPINE, V8, P385, DOI 10.1097/00007632-198305000-00008

WICKERS FC, 1977, CLIN ORTHOP RELAT R, P62

Wong MS, 2000, PROSTHET ORTHOT INT, V24, P148, DOI 10.1080/03093640008726538

WONG MS, 1995, THESIS HONG KONG POL

NR 25

TC 25

Z9 26

U1 0

U2 5

PU SAGE PUBLICATIONS LTD

PI LONDON

PA 1 OLIVERS YARD, 55 CITY ROAD, LONDON EC1Y 1SP, united kingdom

SN 0309-3646

EI 1746-1553

J9 PROSTHET ORTHOT INT

JI Prosthet. Orthot. Int.

PD APR

PY 2001

VL 25

IS 1

BP 60

EP 70

DI 10.1080/03093640108726570

PG 11

WC Orthopedics; Rehabilitation

WE Science Citation Index Expanded (SCI-EXPANDED); Conference Proceedings Citation Index - Science (CPCI-S)

SC Orthopedics; Rehabilitation

GA 432TW

UT WOS:000168712600010

PM 11411007

DA 2023-08-10

ER

PT J

AU den Boer, WA

Anderson, PG

von Limbeek, J

Kooijman, MAP

AF den Boer, WA

Anderson, PG

von Limbeek, J

Kooijman, MAP

TI Treatment of idiopathic scoliosis with side-shift therapy: an initial

comparison with a brace treatment historical cohort

SO EUROPEAN SPINE JOURNAL

LA English

DT Article

DE side-shift; idiopathic scoliosis; treatment; posture training

ID ADOLESCENT SCOLIOSIS; STIMULATION

AB A group of 44 patients with idiopathic scoliosis (mean age 13.6 years) with an initial Cobb angle between 20 degrees and 32 degrees received side-shift therapy (mean-treatment duration 2.2 years). A group of 120 brace patients (mean age 13.6 years) with an initial Cobb angle in the same range (mean brace treatment 3.0 years) was the historical reference group. Failure was defined as an increase of Cobb angle greater than 5 degrees within 4 months or a Cobb angle greater than 35 degrees or a total increase of Cobb angle greater than 10 degrees. The chance of success was not significantly different between the side-shift and the brace groups, whether tested for efficiency (66% vs 68%) or efficacy (85% vs 90%). The difference in the mean progression of the Cobb angle for the respective groups is small (for efficiency: 3 degrees vs -2 degrees, for efficacy: 2 degrees vs -1 degrees). Side-shift therapy appears to be a promising additional treatment for idiopathic scoliosis in adolescents with an inital Cobb angle between 20 degrees and 32 degrees.

C1 Sint Maartensklin, Dept Orthoped Res, NL-6500 GM Nijmegen, Netherlands.

C3 Sint Maartens Clinic

RP Anderson, PG (通讯作者)， Sint Maartensklin, Dept Orthoped Res, POB 9011, NL-6500 GM Nijmegen, Netherlands.

CR AXELGAARD J, 1983, SPINE, V8, P242, DOI 10.1097/00007632-198304000-00004

AXELGAARD J, 1983, SPINE, V8, P463, DOI 10.1097/00007632-198307000-00004

BIRBAUMER N, 1994, J PSYCHOSOM RES, V38, P623, DOI 10.1016/0022-3999(94)90060-4

BRADFORD DS, 1983, SPINE, V8, P757, DOI 10.1097/00007632-198310000-00012

EDMONSON AS, 1977, CLIN ORTHOP RELAT R, P58

ELIASON MJ, 1984, J DEV BEHAV PEDIATR, V5, P169

HANKS GA, 1988, SPINE, V13, P626

KEHL DK, 1988, CLIN ORTHOP RELAT R, P34

MACLEAN WE, 1989, J PEDIATR ORTHOPED, V9, P257

Mehta MH., 1985, SCOLIOSIS PREVENTION, P126

NACHEMSON A, 1982, 17 ANN M SCOL RES SO

NACHEMSON AL, 1995, J BONE JOINT SURG AM, V77A, P815, DOI 10.2106/00004623-199506000-00001

ODONNELL CS, 1988, CLIN ORTHOP RELAT R, P107

Rowe DE, 1997, J BONE JOINT SURG AM, V79A, P664, DOI 10.2106/00004623-199705000-00005

SHIRADO O, 1995, ARCH PHYS MED REHAB, V76, P621, DOI 10.1016/S0003-9993(95)80630-X

Slot G H, 1982, Ned Tijdschr Geneeskd, V126, P325

STYBLO K, 1991, THESIS BRENDA

SULLIVAN JA, 1986, SPINE, V11, P903, DOI 10.1097/00007632-198611000-00010

WATTS HG, 1977, CLIN ORTHOP RELAT R, P87

WINTER RB, 1986, SPINE, V11, P700

NR 20

TC 29

Z9 31

U1 0

U2 5

PU SPRINGER VERLAG

PI NEW YORK

PA 175 FIFTH AVE, NEW YORK, NY 10010 USA

SN 0940-6719

J9 EUR SPINE J

JI Eur. Spine J.

PD OCT

PY 1999

VL 8

IS 5

BP 406

EP 410

DI 10.1007/s005860050195

PG 5

WC Clinical Neurology; Orthopedics

WE Science Citation Index Expanded (SCI-EXPANDED)

SC Neurosciences & Neurology; Orthopedics

GA 254PW

UT WOS:000083622200012

PM 10552325

OA Green Published

DA 2023-08-10

ER

PT J

AU Kunimune, Y

Harada, Y

Kabuto, Y

Takeuchi, K

Senda, M

Inoue, H

AF Kunimune, Y

Harada, Y

Kabuto, Y

Takeuchi, K

Senda, M

Inoue, H

TI Recovery from exercise-induced desaturation in the paraspinal muscles in

idiopathic scoliosis

SO SPINE

LA English

DT Article

DE idiopathic scoliosis; near-infrared light spectroscopy; paraspinal

muscle; tissue oxymeter

ID SPECTROSCOPY; OXYGENATION; FOREARM

AB Study Design. A study using near-infrared light spectroscopy to measure recovery from exercise-induced desaturation in the paraspinal muscles of patients with idiopathic scoliosis.

Objectives. To measure oxygenation of the paraspinal muscles and obtain differences between the convex and concave sides.

Summary of Background Data. Authors of previous studies have repotted that some patients experience pain on the convex side of the paraspinal muscles. The muscles on the convex side are more stretched and stressed than those on the concave side. The current authors investigated the degree of stress by measuring oxygenation and blood volume changes.

Methods. Paraspinal muscle spectral properties at L3 were investigated using near-infrared light spectroscopy. Thirty-six patients (8 men and 28 women) underwent this procedure. To assess a level of peripheral adaptations to exercise, the half-time of Oxyhemoglobin/Myoglobin recovery was measured, which indicates the recovery from energy deficit after exercise.

Results. The average half-time recovery on the convex side was 3.38 seconds (range, 1.5-5.5 seconds), whereas that on the concave side was 1.51 seconds (range, 0.7-4.0 seconds). The average difference between the convex side and the concave side was 1.87 seconds (range, 0.9-3.5 seconds).

Conclusions. Half-time recovery on the convex side in the patients with idiopathic scoliosis was slower than ih at in healthy adults ($P < 0.05$). Half-time recovery on the concave side in such patients was faster than that on the convex side ($P < 0.01$). The authors consider half-time recovery an indication of back muscle stress.

C1 Okayama Univ, Sch Med, Dept Orthopaed Surg, Okayama 7008558, Japan.

C3 Okayama University

RP Kunimune, Y (通讯作者)，Okayama Univ, Sch Med, Dept Orthopaed Surg, 2-5-1 Shikata Cho, Okayama 7008558, Japan.

CR ASCANI E, 1986, SPINE, V11, P784, DOI 10.1097/00007632-198610000-00007

Breit GA, 1997, J BONE JOINT SURG AM, V79A, P838, DOI 10.2106/00004623-199706000-00006

CHANCE B, 1990, P SOC PHOTO-OPT INS, V1204, P481, DOI 10.1117/12.17711

CHANCE B, 1992, AM J PHYSIOL-CELL PH, V262, P766

COBB JR, 1948, AAOS INSTR COURSE, V261, P261

COLLIS DK, 1969, J BONE JOINT SURG AM, VA 51, P425, DOI 10.2106/00004623-196951030-00001

EDGER MA, 1987, ORTHOPAEDICS, V10, P931

FERRARI M, 1992, J PHOTOCH PHOTOBIO B, V16, P141, DOI 10.1016/1011-1344(92)80005-G

FIGUEIREDO UM, 1981, J BONE JOINT SURG BR, V63, P61, DOI 10.1302/0301-620X.63B1.7204475

HAMAOKA T, 1992, MED SPORT SCI, V37, P421

HAMAOKA T, 1993, J TOKYO MED COLL, V51, P437

HAMPSON NB, 1988, J APPL PHYSIOL, V64, P2449, DOI 10.1152/jappl.1988.64.6.2449

JOBSIS FF, 1974, AM REV RESPIR DIS, V110, P58

KAWAKAMI N, 1991, ORTHOPAEDICS, V44, P7

KING HA, 1983, J BONE JOINT SURG AM, V65, P1302, DOI 10.2106/00004623-198365090-00012

LONSTEIN JE, 1995, MOES TXB SCOLIOSIS O, P221

NACHEMSON A, 1968, ACTA ORTHOP SCAND, V39, P466, DOI 10.3109/17453676808989664

NILSONNE U, 1968, ACTA ORTHOP SCAND, V39, P456, DOI 10.3109/17453676808989663

OZAWA T, 1995, BIOCHEM BIOPH RES CO, V213, P432, DOI 10.1006/bbrc.1995.2150

PATTERSON MS, 1989, APPL OPTICS, V28, P2331, DOI 10.1364/AO.28.002331

SASAO Y, 1997, J JPN SCOLIOSIS SOC, V12, P16

SHIGA T, 1995, MED BIOL ENG COMPUT, V33, P622, DOI 10.1007/BF02522525

TOLO VT, 1978, J BONE JOINT SURG BR, V60, P181, DOI 10.1302/0301-620X.60B2.659460

TREMPER KK, 1989, ADV ANESTHESIA, V6, P97

WANG DJ, 1990, 9TH SOC MAGN RES MED, V1, P175

ZATINA MA, 1986, J VASC SURG, V3, P411

NR 26

TC 8

Z9 9

U1 0

U2 3

PU LIPPINCOTT WILLIAMS & WILKINS

PI PHILADELPHIA

PA 530 WALNUT ST, PHILADELPHIA, PA 19106-3621 USA

SN 0362-2436

J9 SPINE

JI SPINE

PD OCT 1

PY 1999

VL 24

IS 19

BP 2019

EP 2024

DI 10.1097/00007632-199910010-00010

PG 6

WC Clinical Neurology; Orthopedics

WE Science Citation Index Expanded (SCI-EXPANDED)

SC Neurosciences & Neurology; Orthopedics

GA 244EJ

UT WOS:000083038600013

PM 10528378

DA 2023-08-10

ER

PT J

AU Athanasopoulos, S

Paxinos, T

Tsafantakis, E

Zachariou, K

Chatziconstantinou, S

AF Athanasopoulos, S

Paxinos, T

Tsafantakis, E

Zachariou, K

Chatziconstantinou, S

TI The effect of aerobic training in girls with idiopathic scoliosis

SO SCANDINAVIAN JOURNAL OF MEDICINE & SCIENCE IN SPORTS

LA English

DT Article

DE scoliosis; brace; aerobic training; aerobic capacity; respiratory

function

ID CAPACITY

AB The purpose of the present study was to investigate the effect of aerobic training in girls with idiopathic scoliosis, in selective respiratory parameters (VC, FVC, FEV1, FEV1/VC% FEV1/FVC) as well as the ability to perform aerobic work (PWC170) The training group consisted of 20 young girls with a mean age of 13.5 +/- 0.16 years. They wore a Boston-type brace for a mean period of 0.30 +/- 0.04 years and suffered from a scoliotic curve (27.4 +/- 1.9 degrees). They followed a two-month training program on the cycloergometer. Each training session lasted 30 min and was performed four times a week. The control group consisted of 20 girls with a mean age of 13.6 +/- 0.18 years and they wore the same type of brace for a mean period of 0.24 +/- 0.04 years. They also suffered from a scoliosis (29.5 +/- 1.8 degrees). The results of the study revealed that aerobic training sustained (VC, FEV1, FEV,NC) or improved significantly (FVC, FEV1/FVC) the parameters of pulmonary function, while the respective parameters (VC, FVC) for the control group were reduced during the two-month period. The ability to perform aerobic work increased 48.1% (P < 0.01) in the training group, while it decreased 9.2% (P < 0.01) in the control group.

C1 Univ Athens, Dept Phys Educ & Sport Sci, Athens, Greece.

Hellen Army Acad, Athens, Greece.

KAT Hosp, Athens, Greece.

C3 National & Kapodistrian University of Athens

RP Paxinos, T (通讯作者)， 4 Ainou St,Nea Smyrni, Athens 17124, Greece.

CR BERGOFSKY EH, 1959, MEDICINE, V38, P263, DOI 10.1097/00005792-195909000-00004

BJURE J, 1969, ACTA ORTHOP SCAND, V40, P325, DOI 10.3109/17453676908989511

CHONG KC, 1981, J PEDIATR ORTHOPED, V1, P251, DOI 10.1097/01241398-198111000-00002

CLUKER T, 1962, JBJS, V3, pA44

DIROCCO PJ, 1988, ARCH PHYS MED REHAB, V69, P198

DURNIN JVG, 1967, BRIT J NUTR, V21, P681, DOI 10.1079/BJN19670070

EVANS BJ, 1981, SPINE, V11, P792

GRINTON S, 1992, MED SCI SPORT EXER, V24, P551

HUSSAIN SNA, 1985, J APPL PHYSIOL, V58, P2020, DOI 10.1152/jappl.1985.58.6.2020

KENNEDY JD, 1989, THORAX, V44, P548, DOI 10.1136/thx.44.7.548

KENNEDY JD, 1987, THORAX, V42, P959, DOI 10.1136/thx.42.12.959

LOW WD, 1983, CLIN ORTHOP RELAT R, P217

NOBLEJAMIESON CM, 1986, ARCH DIS CHILD, V61, P178, DOI 10.1136/adc.61.2.178

Quanjer PH., 1983, STANDARDIZED LUNG FU

SEVASTIKOGLOU JA, 1976, ACTA ORTHOP SCAND, V47, P540, DOI 10.3109/17453677608988734

SHNEERSON JM, 1979, ACTA ORTHOP SCAND, V50, P303, DOI 10.3109/17453677908989771

SHNEERSON JM, 1978, THORAX, V33, P747, DOI 10.1136/thx.33.6.747

WEBER B, 1975, AM REV RESPIR DIS, V111, P389

NR 18

TC 20

Z9 22

U1 1

U2 7

PU MUNKSGAARD INT PUBL LTD

PI COPENHAGEN

PA 35 NORRE SOGADE, PO BOX 2148, DK-1016 COPENHAGEN, DENMARK

SN 0905-7188

J9 SCAND J MED SCI SPOR

JI Scand. J. Med. Sci. Sports

PD FEB

PY 1999

VL 9

IS 1

BP 36

EP 40

PG 5

WC Sport Sciences

WE Science Citation Index Expanded (SCI-EXPANDED)

SC Sport Sciences

GA 160GE

UT WOS:000078221400005

PM 9974195

DA 2023-08-10

ER

PT J

AU Fayada, P

Morin, C

Plais, PY

Leonard, JC

AF Fayada, P

Morin, C

Plais, PY

Leonard, JC

TI Scoliosis, kyphosis and sports.

SO SCIENCE & SPORTS

LA French

DT Article

DE sports; scoliosis; kyphosis

AB Sport or any other form of physical activity is or should be a necessity far children, even if they present with spine abnormalities such as scoliosis or kyphosis. Any contra-indication to exercising should be the exception rather than the rule. Insofar as possible if should be temporary even in the

case of fused spine. Precise and careful orthopedic and neurological examination should be the prelude to any decision. The opinion of a specialist is often necessary. Curved and painful rachis should lead the practitioner to first eliminate the potential existence of either inflammatory or tumoral pathology, as idiopathic scoliosis is rarely painful. Except during painful episodes, the majority of sports is accessible to patients with kyphosis and/or scoliosis, even if they need to take off their corset for a short while. The objective of this article was therefore to show that a multidisciplinary medical team confronted daily with a wide variety of spine abnormalities is quite permissive in regard to classic and reasonable practice of sport (C) 1999 Elsevier, Paris.

C1 Inst Calot, Serv Chirurg Rachis, F-62600 Berck Sur Mer, France.

Inst Calot, Serv Chirurg Orthoped & Reparatrice Enfant, F-62600 Berck Sur Mer, France.

Inst Calot, Serv Readaptat Fonct & Med Phys, F-62600 Berck Sur Mer, France.

RP Leonard, JC (通讯作者)， Inst Calot, Serv Chirurg Rachis, F-62600 Berck Sur Mer, France.

OI morin, christian/0000-0002-0917-9890

NR 0

TC 2

Z9 2

U1 1

U2 5

PU EDITIONS SCIENTIFIQUES MEDICALES ELSEVIER

PI PARIS CEDEX 15

PA 23 RUE LINOIS, 75724 PARIS CEDEX 15, FRANCE

SN 0765-1597

J9 SCI SPORT

JI Sci. Sports

PY 1999

VL 14

IS 1

BP 28

EP 32

DI 10.1016/S0765-1597(99)80025-2

PG 5

WC Sport Sciences

WE Science Citation Index Expanded (SCI-EXPANDED)

SC Sport Sciences

GA 192MJ

UT WOS:000080082700009

DA 2023-08-10

ER

EF
